# Leveraging Mechanistic Insights into Stereoretentive ROMP for Precision Synthesis of Poly(*p*‑phenylene vinylene)s

**DOI:** 10.1021/jacs.6c02915

**Published:** 2026-05-05

**Authors:** Jake L. Nicholson, Samuel J. Kempel, Ángel Rentería-Gómez, Ting-Wei Hsu, Antoine C. Gravet, Caroline E. Gallo, Osvaldo Gutierrez, Quentin Michaudel

**Affiliations:** † Department of Chemistry, 14736 Texas A&M University, College Station, Texas 77843, United States; ‡ Department of Materials Science and Engineering, Texas A&M University, College Station, Texas 77843, United States; § Department of Chemistry and Biochemistry, University of California, Los Angeles, Los Angeles, California 90095, United States

## Abstract

Poly­(*p*-phenylene vinylene) (PPV) is an important class of conjugated polymers whose properties are largely dictated by structural parameters such as backbone regioregularity, defect content, alkene geometry, side-chain structure, and the sequence of repeat units. Despite decades of development and applications spanning optoelectronic devices to biomedical imaging, a controlled, defect-free polymerization method that affords predictable command over all structural attributes of PPVs has remained elusive. We report herein the synthesis of a variety of regioregular and stereodefined PPVs through stereoretentive ring-opening metathesis polymerization (ROMP) of electronically and sterically diverse [2.2]­paracyclophane-1,9-dienes (PCDs). Computational studies were combined with experimental findings to uncover structural design principles underlying controlled propagation as well as regio- and stereoselectivity. These findings were harnessed to access well-defined gradient and one-shot block PPV copolymers, as well as unprecedented stereoblocks with *cis* and *trans* block geometries through *in situ* photoisomerization. The influence of monomer composition and alkene geometry on the optical properties of the synthesized PPVs was also investigated. The exceptional structural control achieved through stereoretentive ROMP of PCDs establishes a unique platform for the precise synthesis of PPVs with tailored properties.

## Graphical abstract

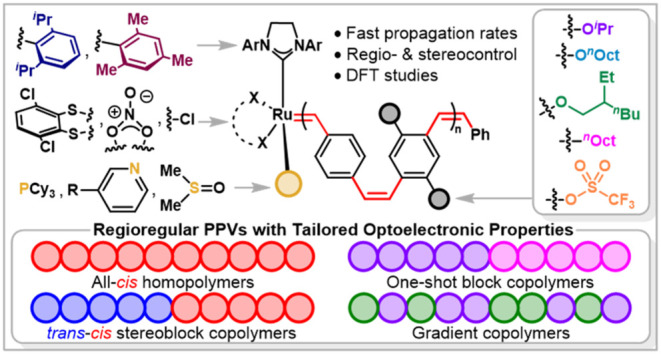

## Introduction

Poly­(*p*-phenylene vinylene) (PPV) represents a prominent class of conjugated polymers whose electroluminescence efficiency and narrow bandgap have enabled widespread application in optoelectronic devices, including heterojunction solar cells^1,2^ and organic light-emitting diodes.^3–6^ Recently, Samuel and co-workers demonstrated that PPV can also serve as an effective light-amplifying medium in an all-organic semiconductor laser driven by electrical pumping.^7^ Beyond optoelectronic devices, amphiphilic PPV copolymers have also been synthesized to form photostable, biocompatible, and noncytotoxic fluorescent micelles and polymer nanoparticles, extending the utility of PPV materials to bioimaging and drug delivery.^8,9^

The broad appeal of PPVs stems from their structural versatility, which enables precise control over physical properties including charge mobility, optoelectronic bandgap, and processability. For example, conjugated polymers with high regioregularity are well-known to have superior charge mobilities arising from enhanced crystallinity and self-organization in films.^5,10–15^ While *trans*-rich PPVs often have more desirable optoelectronic properties, *cis*-rich PPVs possess enhanced solubility due to their coil-like structure reducing aggregation caused by increased π–π stacking in the more coplanar *trans* conformers.^16,17^ All-*trans* PPVs can also be readily accessed from PPVs containing *cis* alkenes through one-way photoisomerization,^18^ enabling further application in photopatterning^19^ and crystallization-driven self-assembly.^20^ Furthermore, the optoelectronic bandgap and film crystallinity can be modulated through side-chain design and copolymer structure.^6^ Lastly, accessing PPVs with low defect content is essential for improving device performance and lifetime.^21^

Despite this large body of work, typical synthetic routes to PPVs lack full control over all structural parameters, often improving one feature at the expense of another. Polycondensation and precursor routes typically exhibit poor control over molar mass (*M*_n_), dispersity (*Đ*), and *cis*/*trans* stereochemistry of the repeating alkenes.^22–26^ While stereodefined all-*cis* PPVs were initially accessed through stereoretentive Suzuki-Miyaura polymerization^20,27^ and *syn*-reduction of poly­(phenylene ethynylene),^28^ both strategies relied on step-growth processes, which typically do not allow for precise control over molar mass distribution and chain-ends. Ring-opening metathesis polymerization (ROMP) of highly strained unsaturated cyclophane monomers using dichloro Grubbs catalysts has enabled a controlled PPV chain-growth process, but affords *cis*/*trans* mixtures.^29–35^ Recently, we achieved PPVs combining perfect *cis*-selectivity and living characteristics via the stereoretentive ROMP of [2.2]­paracyclophane-1,9-diene (PCD)^36,37^ using a dithiolate Ru catalyst initially reported by Hoveyda.^38,39^ This strategy delivered all-*cis* PPVs with narrow *Đ*’s and predictable *M*_n_’s up to 108.8 kg/mol.^40^ Importantly, *cis*-to-*trans* photoisomerization provides access to all-*cis*, all-*trans*, and intermediate PPV mixtures, facilitating direct study of stereochemical effects on complex optical properties including two-photon absorption of quantum light.^41^

Beyond delivering stereodefined PPV materials, our early studies uncovered puzzling differences in reactivity between dichloro Ru catalysts (e.g., Grubbs second generation) and dithiolate Ru catalysts with PCD monomers. Herein, we report a dual experimental and computational investigation of the mechanism of the stereoretentive ROMP of PCDs rationalizing these phenomena. Key mechanistic insights enabled the synthesis of PPVs with unparalleled structural control and complexity, including regioregular copolymers with tunable monomer sequences and *cis*/*trans* stereoblocks (Figure 1). The study of the photophysics of the synthesized homo- and copolymers revealed stark differences in their absorption and emission properties, which highlight the importance of fine-tuning the structure of PPVs through precision synthesis.

**Figure 1:**
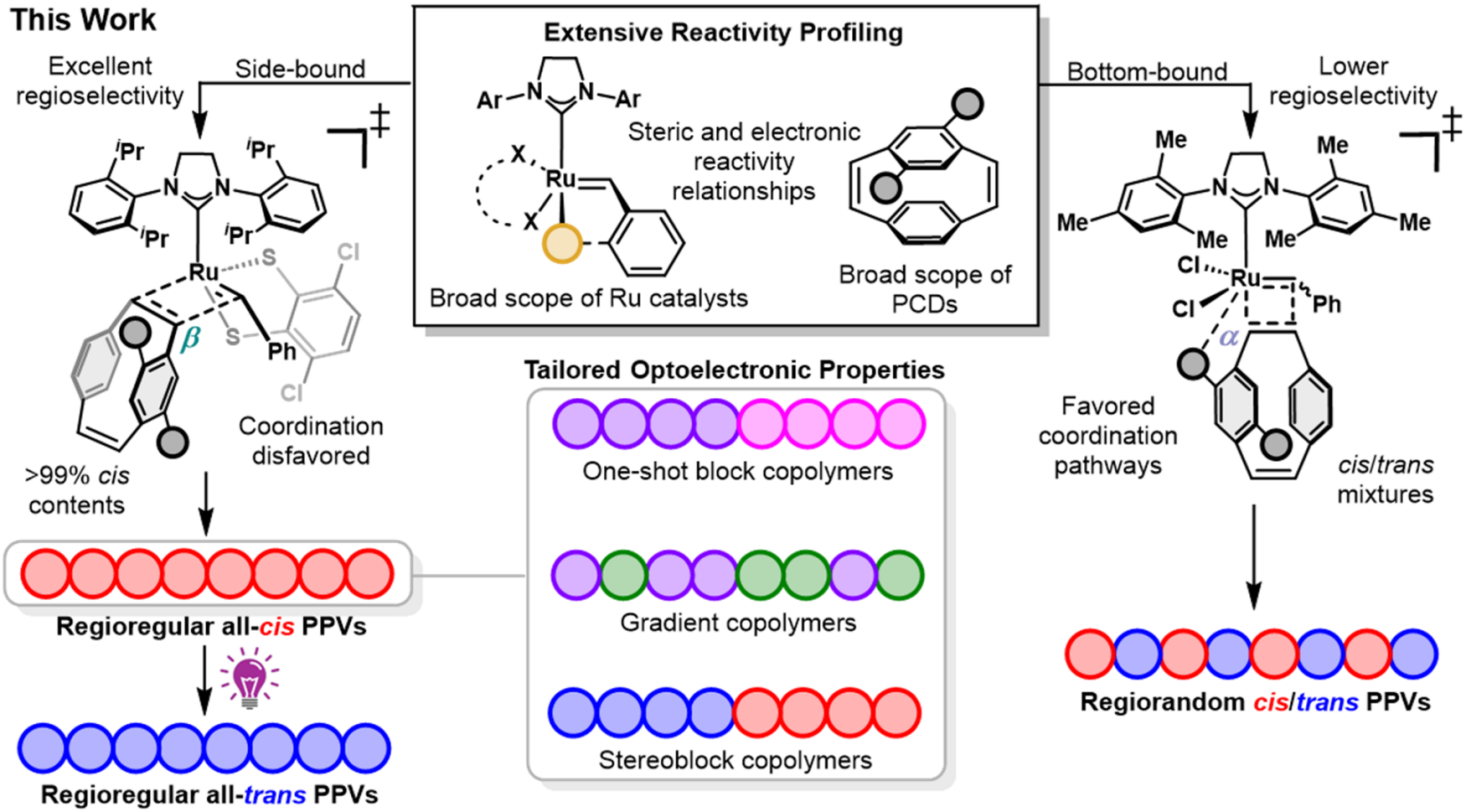
Overview of this mechanistic investigation into the stereoretentive ROMP of PCDs to access regio- and stereodefined PPVs with increasing structural complexity.

## Results and Discussion

### Kinetics of the Stereoretentive ROMP of Various PCDs

In our prior report,^40^ electron-rich alkoxy PCDs were found to polymerize not only with high *cis*-selectivity, but also fast rates of propagation using dithiolate catalyst **Ru-1a**, in stark contrast with the sluggish rates obtained for the same monomers and dichloro Grubbs second (**GII**) and third (**GIII**) generation catalysts.^32,42–44^ Slower rates for alkoxy-substituted PCDs have been ascribed to the formation of a 16-electron complex through oxygen coordination to the dichloro Ru center.^45^ This lack of reactivity is a major limitation in the broad adoption of ROMP for the synthesis of PPVs toward optoelectronic devices, since alkoxy substituents are commonly introduced to redshift the absorbance and increase charge mobilities.^2,5,46–49^

To experimentally decipher the influence of electronic and steric effects on the *cis*-selective ROMP of PCDs, five monomers (**M1**–**M5**) were prepared (Figure 2a). **M2**–**M4** were successfully synthesized on gram-scale, which represents a promising step toward the larger-scale production of PPVs using this method. The steric hindrance of the alkoxy side chains was increased from **M1**–**M3**, while **M4** bears alkyl side chains without coordinating oxygen. Bistriflate monomer **M5** was also prepared to minimize the dativity of the oxygens through inductive and resonance effects. Of note, **M1** and **M5** have not been reported prior to this study. A variety of stereoselective Ru catalysts^50^ previously used by our group in the synthesis of *cis*-rich polyalkenamers were also investigated.^51–53^ Dithiolate catalysts **Ru-1a**, **Ru-1b**, and **Ru-2** ^54^ were employed to interrogate the impact of the N-heterocyclic carbene (NHC) ligand and Hoveyda isopropoxy chelate on the polymerization kinetics as well as regio- and stereoselectivity of the ROMP process (Figure 2b). Cyclometalated nitrato^55^ catalysts **Ru-3a** and **Ru-3b** are also known to favor a side-bound approach^56,57^ of the strained alkene and were therefore added to the screening despite typically exhibiting slower initiation rates in ROMP of strained norbornene derivatives.^55,58–60^

**Figure 2:**
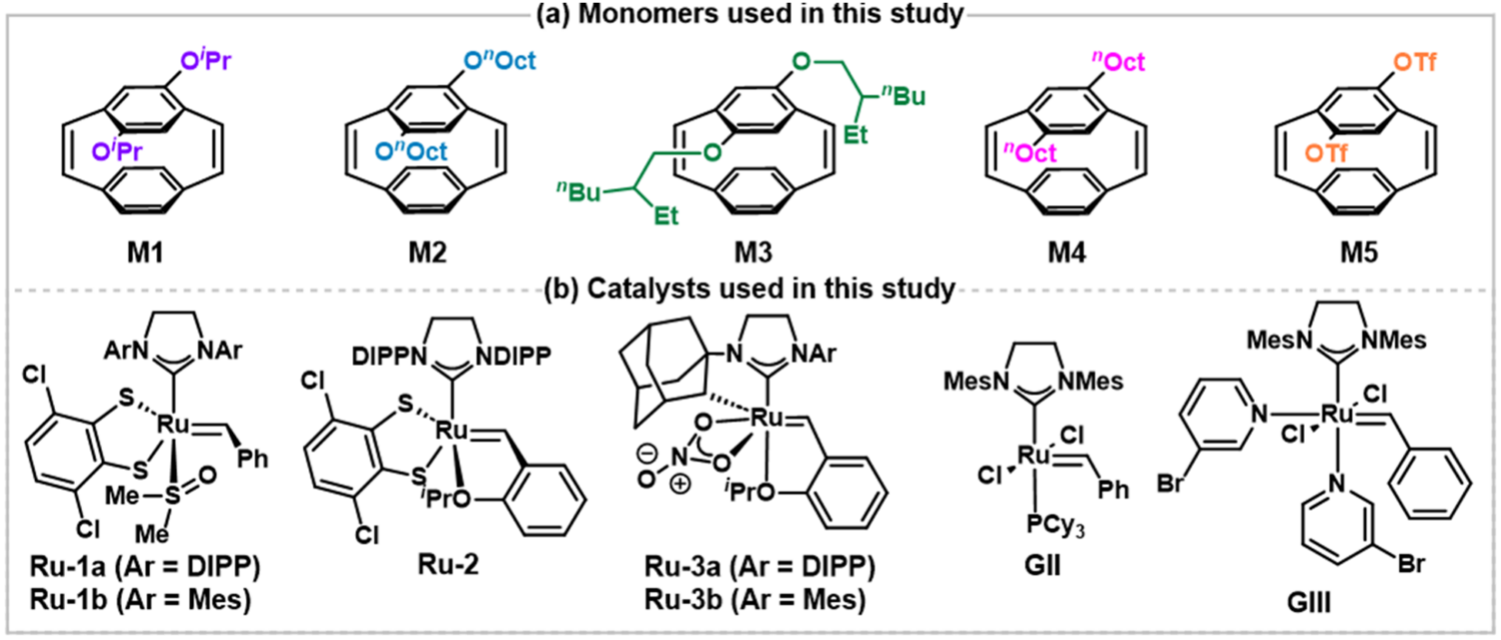
(a) Scope of PCDs and (b) catalysts used (DIPP = 2,6-diisopropylphenyl; Mes = 2,4,6-trimethylphenyl).

The kinetics of the stereoretentive ROMP were monitored in C_6_D_6_ first using **Ru-1a** (5 mol %, Table 1). While alkoxy monomers **M1**–**M3** readily polymerized at room temperature, 40 °C was required to polymerize **M4** and **M5**. Size exclusion chromatography (SEC) analysis supported well-controlled polymerizations for **M1**–**M4**, with the corresponding polymers showing narrow *Đ*’s and excellent agreement with theoretical *M*_n_ values (Table 1, Entries 1–4). However, the polymerization of **M5** resulted in slow conversion (83% after 7 days), a broader *Đ*, and significant differences between the theoretical and experimental *M*_n_ (Table 1, Entry 5). In agreement with these observations, fast initiation (*k*_i_ ≫ *k*_p_) and first-order kinetics with respect to monomer concentration were observed for all monomers except **M5** (Figure 3a).

**Table 1:** Summary of Monomer and Catalyst Screening

Entry	Monomer	Ru	*T* (°C)	Time (h)	Conv. (%)	*M*_n_^theo^(kg/mol)	*M*_n_^exp^ (kg/mol)^a^	*Đ*	*cis* (%)^b^
1	**M1**	**Ru-1a**	rt	0.5	97	6.1	6.0	1.25	>99
2	**M2**	**Ru-1a**	rt	9	>99	9.3	9.9	1.16	>99
3	**M3**	**Ru-1a**	rt	20	>99	9.3	10.1	1.06	>99
4	**M4**	**Ru-1a**	40	50	91	7.8	9.1	1.12	>99
5	**M5**	**Ru-1a**	40	168	83^c^	8.6	14.9	1.42	>99
									
6	**M3**	**Ru-1b**	40	106	97	8.9	12.2	1.21	98
7	**M3**	**Ru-2**	rt	0.1	>99	9.3	28.8	1.46	>99
8	**M3**	**Ru-2** ^d^	rt	21	>99	9.3	11.4	1.06	>99
9	**M3**	**Ru-3a**	70	44	10^c^	0.9	4.8	1.48	68
10	**M3**	**Ru-3b**	40	293	87	7.9	10.1	1.81	65
11	**M3**	**GII**	rt	149	98	9.3	7.2	1.38	50
12	**M3**	**GIII**	rt	49	>99	9.3	8.8	1.31	50

a Determined by SEC analysis in THF against polystyrene standards.

b Measured by ^1^H NMR spectroscopy.

c Full catalyst decomposition was observed.

d 2 equiv of 3-ClPy used as an additive.

**Figure 3:**
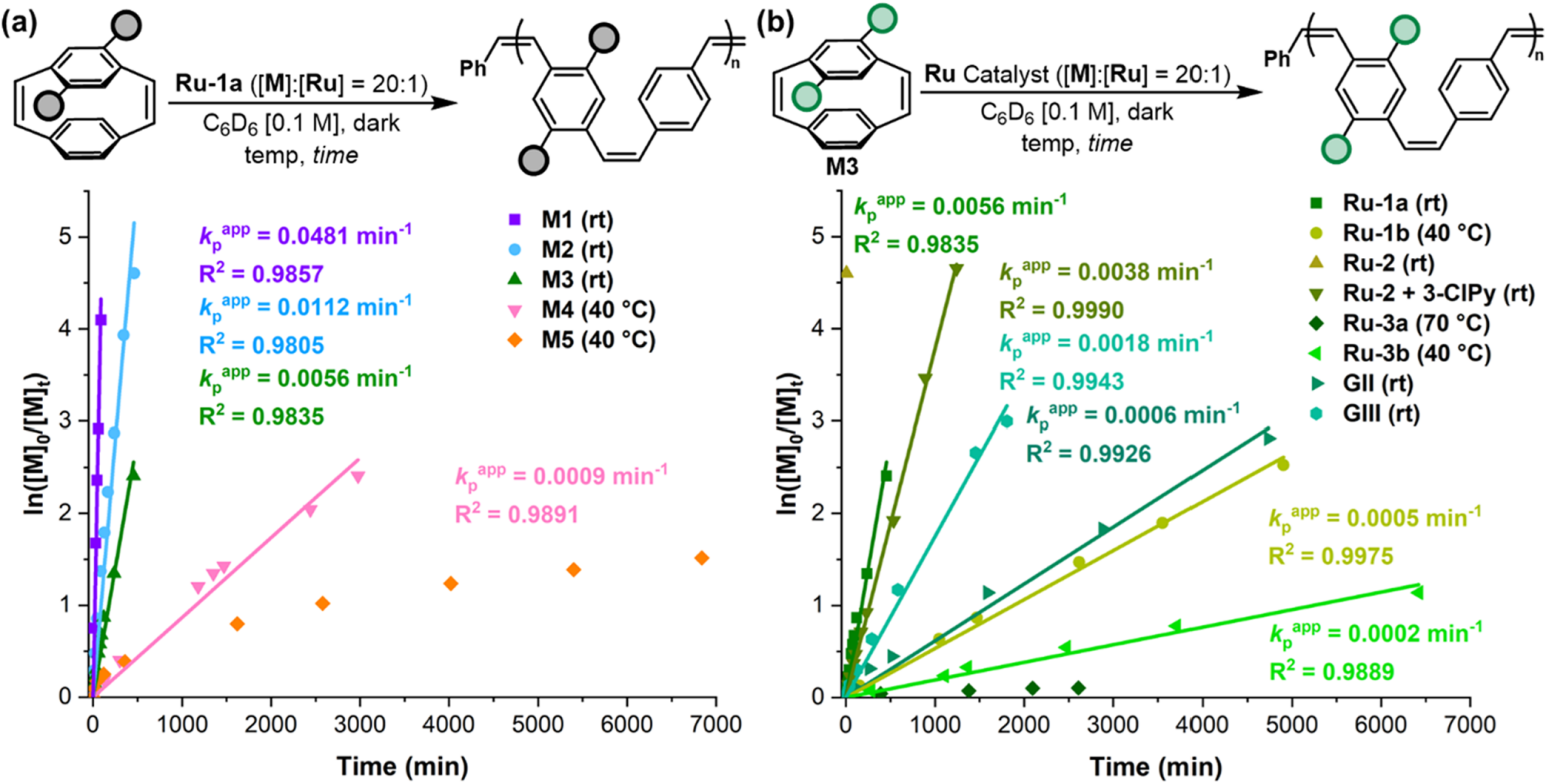
(a) Monomer propagation kinetics using **Ru-1a** and (b) **M3** propagation kinetics using different Ru catalysts.

To further examine the influence of catalyst structure on the stereoretentive ROMP of PCDs, **M3** was polymerized using initiators depicted in Figure 2b. Polymerization using dithiolate **Ru-1b**, the mesityl-NHC variant of **Ru-1a**, required slightly higher temperature (40 °C) and a significantly longer reaction time (Figure 3b and Table 1, Entry 6). Hoveyda-chelate **Ru-2** yielded full monomer conversion within minutes but showed poor control over the polymerization (Table 1, Entry 7). Interestingly, addition of 3-chloropyridine (3-ClPy, 2 equiv) to **Ru-2** restored the first-order kinetics (Table 1, Entry 8). In contrast, cyclometalated initiators **Ru-3a** and **Ru-3b** displayed lower *cis* selectivities, conversions, and polymerization rates even at higher temperatures (Table 1, Entries 9 and 10). Importantly, **Ru-1a** also resulted in a substantially faster propagation rate and narrower *Đ* than **GII** and **GIII** with electron-rich **M3** (Table 1, Entries 11 and 12).

The role of the labile ancillary ligand in stabilizing the active dithiolate catalyst species was further probed both experimentally and computationally. Monitoring the ROMP of **M2** and **M3** with Hoveyda-chelate **Ru-2** revealed incomplete initiation caused by extremely fast propagation (*k*_i_ ≪ *k*_p_, Figure S39). This high *k*_p_ was ascribed to the propagation of an untamed 14-electron species^61,62^ generated in the initiation step between **M2** or **M3** and **Ru-2** resulting in the transfer of the chelating isopropoxy group to the α-chain-end. Addition of 3-ClPy rescued first-order kinetics as well as good control over *M*_n_^exp^ and *Đ* with **Ru-2** and **M1**–**M4** (Figure S36, and Table S1, Entries 6–10), thereby confirming the importance of the labile ancillary ligand for stabilizing the propagating chain-end of the dithiolate species.^54,62,63^ Similar to studies with **GIII**,^64,65^ an inverse first-order dependency on propagation rates with respect to the concentration of 3-ClPy was also observed (Figure S6), with 2 equiv providing robust and reproducible stereoretentive ROMP with **Ru-2**. Interestingly, competition experiments using **Ru-1a** and 3-ClPy suggest that DMSO binds preferentially over 3-ClPy as shown by ^1^H NMR of the respective alkylidene signals (Figure S7). This observation was corroborated by computing the stabilization energies of the Ru carbene provided by different coordinating species compared to the unbound Ru carbene including DMSO (Δ*G* = −17.3 kcal/mol), 3-ClPy (Δ*G* = −11.8 kcal/mol), and diOMe-PCD (**M6**) used as a computational model (Δ*G* = 0.2 kcal/mol, Figure 4).

**Figure 4:**
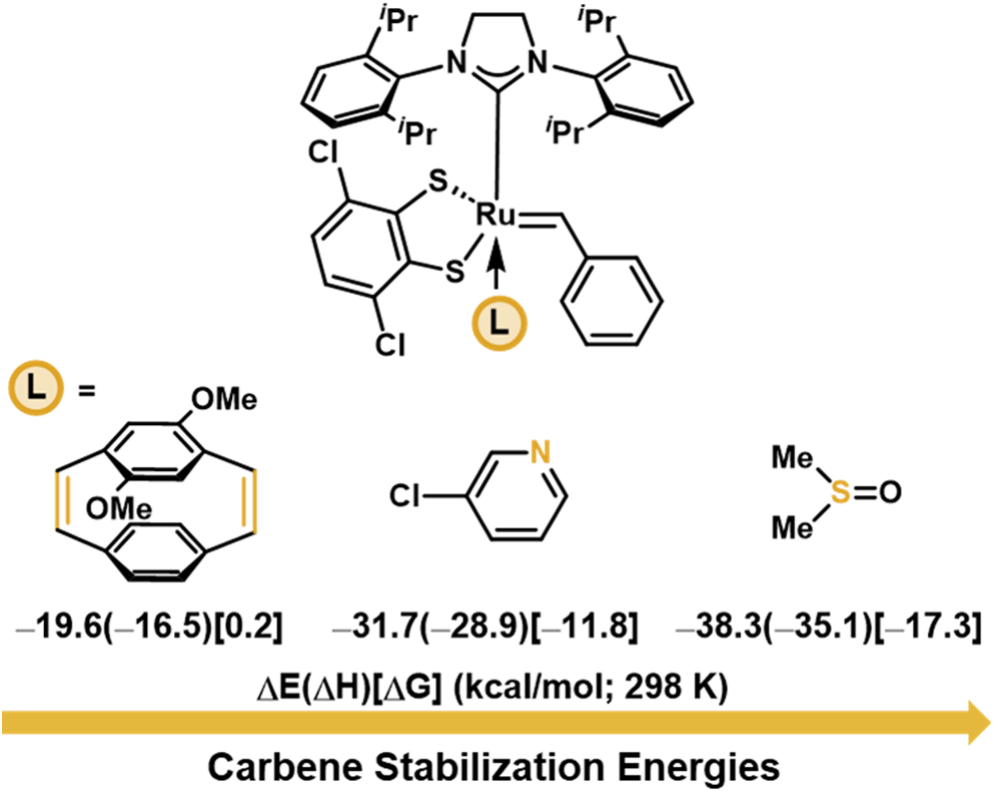
Stabilization energies are calculated at B3LYP-D3/def2svp-SDD­(Ru)-CPCM­(benzene) level of theory and relative to the unbound Ru carbene.

### Experimental Determination of Catalyst Regioselectivity

In addition to marked changes in polymerization rates and *cis*-stereoselectivity, *in*-*situ* ^1^H NMR monitoring of the polymerization of **M3** using **GII, Ru-1b**, and **Ru-1a** revealed major differences in the nature of the propagating alkylidene species (Figure 5a,5b). While multiple carbene signals were observed with **GII** and **Ru-1b**, a single downfield peak was present with **Ru-1a**. These distinct signals were ascribed to the different modes of ring-opening of **M3** putatively accessible due to the unsymmetrical substitution pattern of the PCD. The substituted aryl groups can indeed be placed either proximal or distal to the Ru center (α or β position) during formation of the metallacyclobutane. In agreement with this hypothesis, Turner and co-workers have already characterized the different regioisomers arising from opening of **M3** with **GII** and assigned the most upfield peak to an O-chelate 16-electron complex.^42,66^ By analogy, we suggest the similar formation of α or β regioisomers with **Ru-1b**, as well as a chelated α isomer. The formation of the chelated complex was further probed by addition of DMSO (5 equiv) toward the end of the ROMP leading to the disappearance of the signals at 14.20 and 14.16 ppm and the concomitant increase of the peak at 15.41 ppm (Figure S41). Finally, the exclusive formation of the β isomer with **Ru-1a** was confirmed by NOESY NMR using **M1** that exhibited through-space coupling between the alkylidene proton and those on the unsubstituted aryl group (Figures S42–S44). This remarkable regioselectivity obtained with **Ru-1a** was consistent across the scope of monomers.

**Figure 5:**
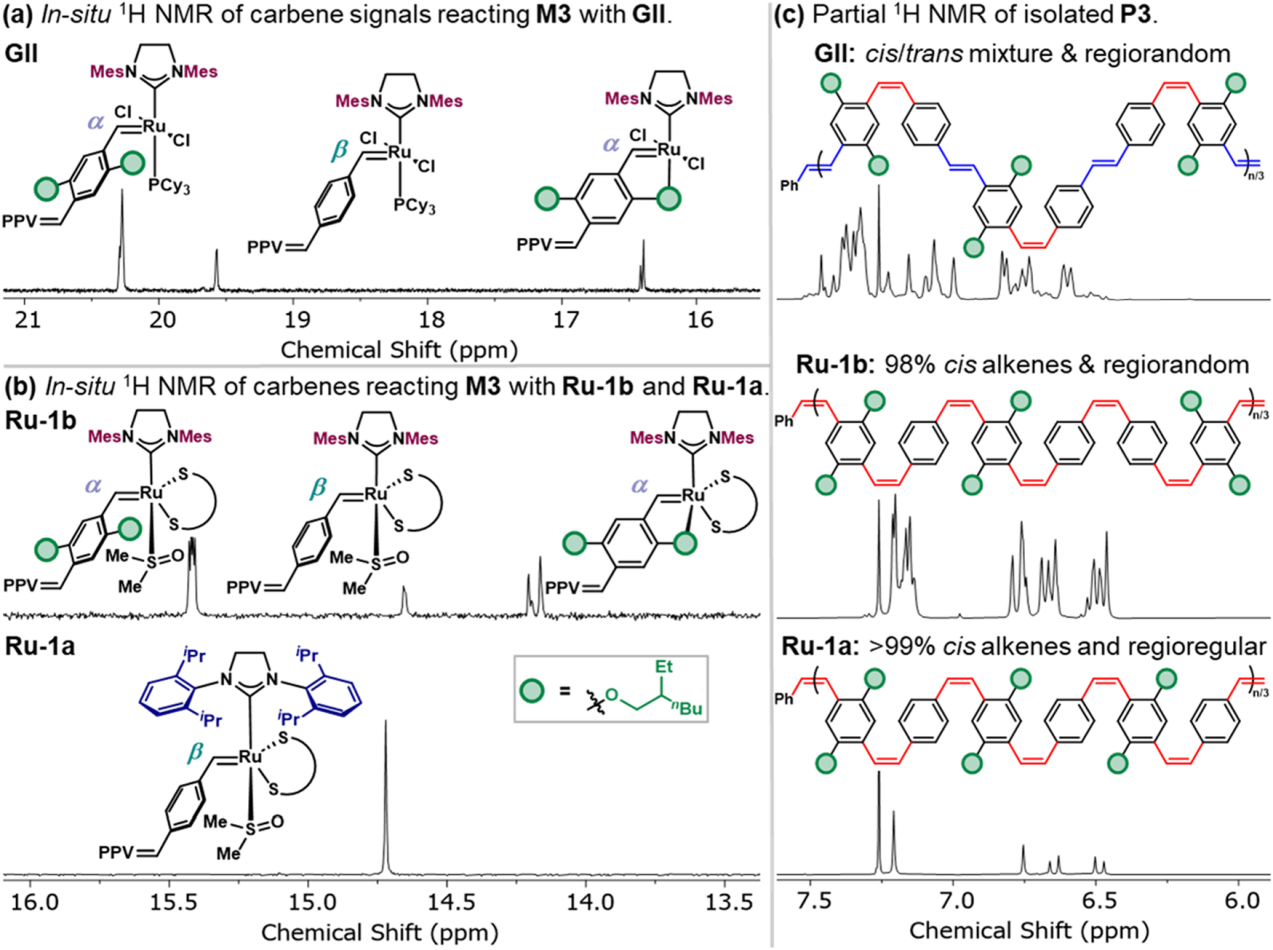
(a) Propagating catalyst species observed when polymerizing **M3** using **GII** as well as (b) **Ru-1b** and **Ru-1a** in C_6_D_6_. (c) ^1^H NMR of aromatic region for the final corresponding polymer products in CDCl_3_.

Importantly, the uniformity of the propagating chain-end arising from **Ru-1a** led to a highly regio- and stereoregular PPV (**P3**, Figure 5c bottom) in contrast to the PPVs produced with **GII** and **Ru-1b** (Figure 5c top and middle). While **P3** made from **Ru-1b** exhibited high *cis* content, the presence of head-to-tail, tail-to-head, and head-to-head linkages likely explains the broader and more complex NMR patterns. Finally, **GII** delivered **P3** with both poor regio- and stereoselectivity. The high regioregularity obtained using **Ru-1a** was consistent across NMR analysis of all isolated PPVs. These findings demonstrate the ability of dithiolate Ru carbenes to control both the regio- and the stereochemical outcomes of each monomer addition and the importance of the bulky NHC ligand (DIPP vs mesityl) to impart high selectivity. Controlling regioselectivity in PPVs has been shown to dramatically improve material performance—for example, regioregular PPVs can exhibit up to a 30-fold increase in charge mobility and greater structural order in thin films compared to regiorandom analogs.^67,68^

### Computational Investigation of Catalyst Regioselectivity and Reactivity Rate Implications

To further investigate the striking selectivity dictated by **Ru-1a**, a series of dispersion-corrected DFT calculations were conducted. Consistent with previous studies by Hoveyda^38^ as well as Houk and Grubbs^69^ with dithiolate catalysts and *cis*-alkenes, a side-bound approach of the monomer appears energetically favorable to avoid a large distortion of the ligand sphere required in the bottom-bound approach (Figure S86). In addition, calculations reveal that stereoinversion is unfavorable due to the direct steric collision of the benzylidene with the aryl substituents on the NHC, which rationalizes the exclusive formation of all-*cis* PPV observed (Figure S87). Interestingly, unlike other strained monomers such as norbornene, calculations using PCD did not lead to the formation of a discrete metallacyclobutane intermediate. Presumably the high ring-strain energy and electron repulsion in the transition states of the two parallel aromatic systems in PCD leads to a barrierless collapse of the metallacyclobutane intermediate (Figures S88–S90). Overall, calculations are consistent with the [2+2] cycloaddition as the rate-determining step.

The planar chirality of disubstituted PCDs and the stereogenicity at Ru center of dithiolate **Ru-1a** ^70,71^ result in four distinct possible side-bound transition states that were computed using **M2** as an example monomer (Figure 6a). Overall, **TS1-** ***β*** **1**, which minimizes steric interactions with the catalyst during metallacyclobutane formation, was found to have the lowest energy compared to the other transition states. To gain further insight into the energy difference between **TS1-** ***α*** **1** and **TS1-** ***β*** **1**, a distortion-interaction analysis was conducted. This approach partitions the activation barrier into the distortion energy required to deform the ground-state fragments (**Ru-1a** and **M2**) into their transition-state geometries and the interaction energy between these distorted fragments in the transition state.^72^ Although **TS1-** ***α*** **1** has a similar *interaction energy* with respect to **TS1-** ***β*** **1** (∼72.0 kcal/mol), this TS exhibits higher *distortion energy*, resulting in an uphill relative energy of 1.9 kcal/mol (Table S2). In addition, **TS1-** ***β*** **2** was computed to be significantly higher in energy (ΔΔ*G* ^‡^ = 7.4 kcal/mol), consistent with steric interactions between the monomer substituent and benzylidene initiator ligand. Furthermore, **TS1-** ***α*** **2**, which situates the monomer substituent beneath the 2,6-diisopropylphenyl group of the NHC, has the highest relative energy (ΔΔ*G* ^‡^ = 7.9 kcal/mol). Analysis of the computed Gibbs free energy surface for the ROMP of **M2** catalyzed by **Ru-1a** using a higher-level DFT method (Figure 6b) shows that the initiation step favors the formation of **Int1-** ***β*** **1** over **Int1-** ***α*** **1** (Δ*G* ^‡^ = 12.2 vs 15.6 kcal/mol). Additionally, in the propagation step, the **β1** pathway via **TS2-** ***β*** **1** is preferred over the **α1** pathway, with similar differences in energy barriers (Δ*G* ^‡^ = 12.8 vs 16.4 kcal/mol) to the initiation step. Given that a ΔΔ*G* ^‡^ value of ∼3.0 kcal/mol corresponds to a predicted selectivity greater than 100:1 at 298 K, the calculated energy difference is in excellent agreement with the experimentally observed regioselectivity (*vide infra*). Importantly, the preference for the β pathway was found to be consistent throughout the ROMP process, including both initiation and propagation, for monomers bearing either alkoxy or alkyl side chains (**M2** vs **M4**), thereby supporting the high regioselectivity of **Ru-1a** irrespective of the electronic character of the monomer side chains.

**Figure 6:**
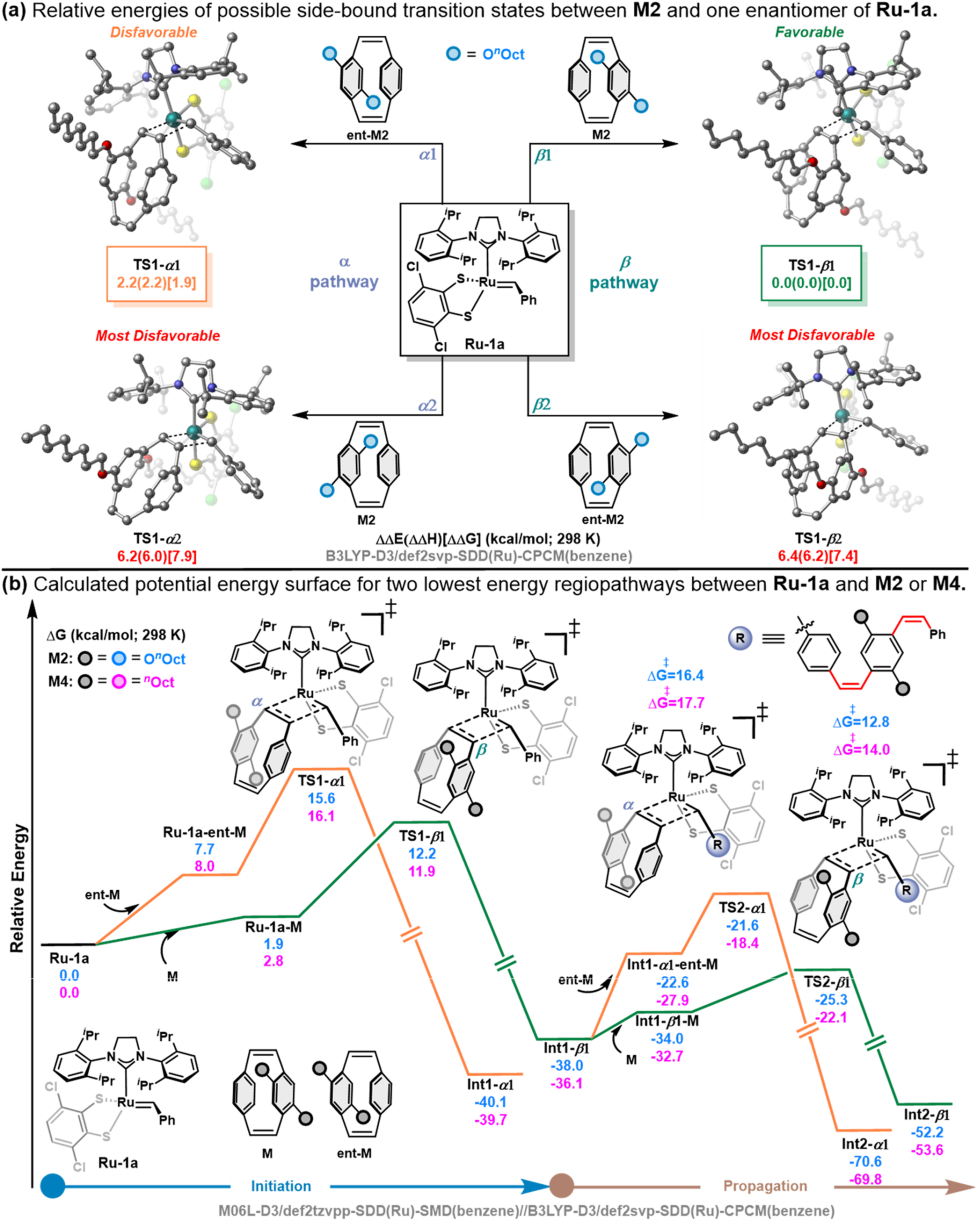
(a) Possible regioselective pathways during stereoretentive ROMP considering enantiomers of **M2** with **Ru-1a** and their relative energies. (b) Calculated potential energy surface for stereoretentive ROMP of **M2** and **M4** with **Ru-1a** considering two lowest energy regioselective pathways (α1 and β1).

Our model also rationalizes the observed differences in regioselectivity between **Ru-1a** and **Ru-1b** (Table 2). Specifically, **Ru-1b** displayed a smaller energy difference between **TS1-** ***α*** **1** and **TS1-** ***β*** **1** (ΔΔ*G* ^‡^ = 0.4 kcal/mol) with a slight preference for **TS1-** ***α*** **1**, while **Ru-1a** exhibited a larger difference between the two pathways in favor of **TS1-** ***β*** **1** (ΔΔ*G* ^‡^ = 1.6 kcal/mol). These calculations correlate well with the ^1^H NMR data pertaining to the distinct propagating carbene species shown in Figure 5b and the experimentally observed lack of regioselectivity with **Ru-1b**. Further distortion–interaction analysis suggested that this may arise from a more favorable interaction energy for **TS1-α1** with **Ru-1b**. However, in the case of **Ru-1a**, which exhibits similar interaction energies, the selectivity is attributed to a higher distortion energy for **TS1-α1** (Table 2).

**Table 2:**
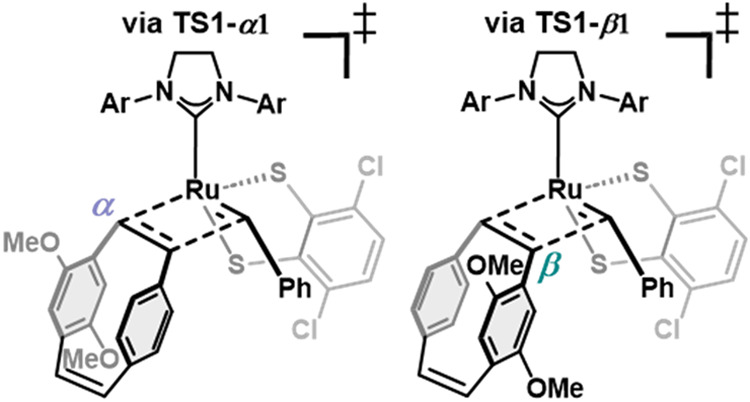

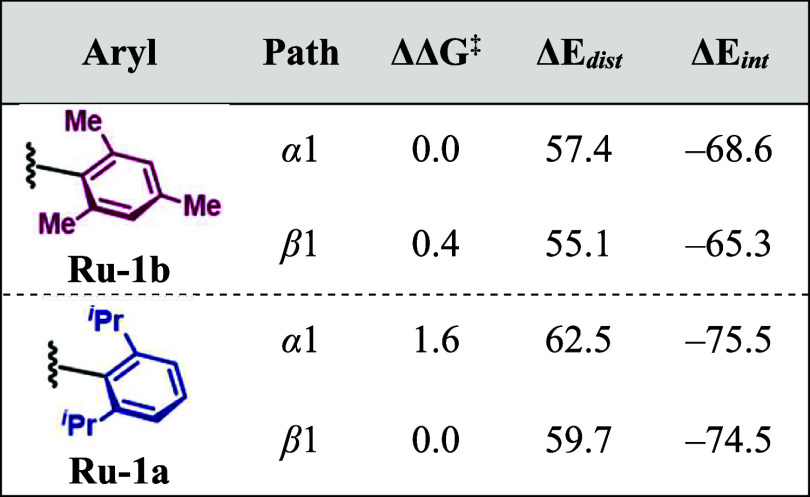
Computed Regioselective Pathway Transition State Energies (kcal/mol) using Ru-1b and Ru-1a with M6 as a Model Monomer^a^

a Calculations performed at B3LYP-D3/def2svp-SDD­(Ru)-CPCM­(benzene) level of theory.

Finally, this computational investigation provides insights into the pronounced differences in polymerization rates observed for electron-rich, alkoxy monomers using dithiolate **Ru-1a** relative to dichloro catalysts **GII** and **GIII**. The propensity of **Ru-1a** to favor formation of the β-ruthenacyclobutane likely precludes O-coordination in the resulting ring-opened adduct, which is prevalent in the α-selective pathway of **GII** and **GIII** (Δ*G*°_rxn_ computed as 10.2 kcal/mol) and has been posited to decrease the propagation rates of alkoxy PCDs with dichloro catalysts (Figure 7a).^42,44^ In contrast, these electron-rich alkoxy side chains may facilitate the formation of the productive ruthenacycle with **Ru-1a** through improved energy-level alignment between the π orbital of the alkene with the empty π* and d orbitals of the Ru=C bond,^73^ thereby increasing propagation rates. Similar phenomena have been discussed in the ROMP of *exo*-norbornene derivatives in the synthesis of linear polymers^74^ and bottlebrush copolymers^75,76^ and described as anchoring effects by Matson and co-workers. In these studies, the reactivity of monomers containing various anchor groups was correlated with HOMO energy levels. In line with this anchoring effect, the computed HOMO energy of **M2** was found to be higher than that of **M4** by 13 kcal/mol, consistent with the higher rates of polymerization for **M2** with **Ru-1a** (Figure 7b). Overall, these results underscore the importance of understanding the subtle effects of coordinating atoms present in ROMP monomers,^64,74,77,78^ as their impact on propagation rates depends strongly on ruthenacycle geometry.

**Figure 7:**
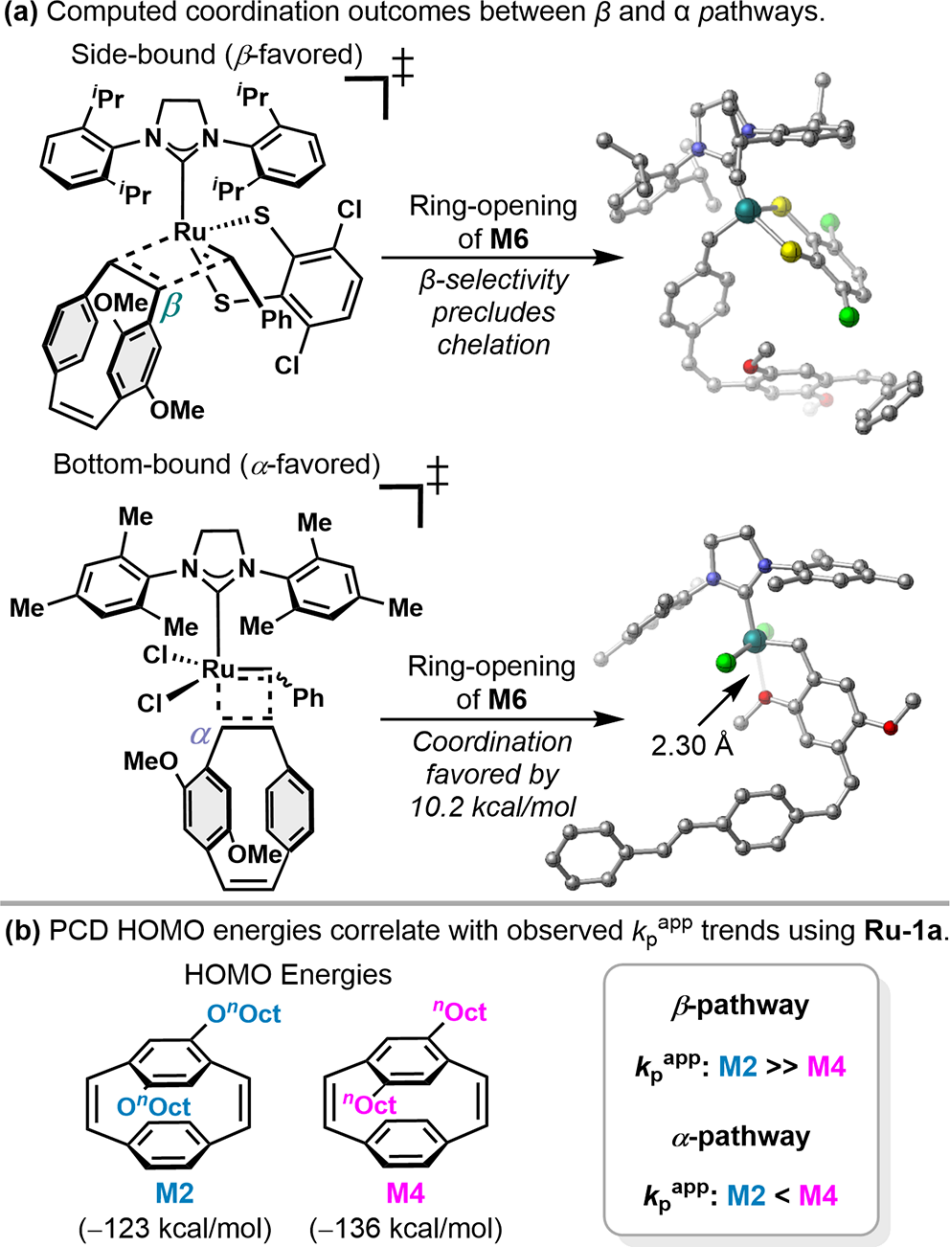
(a) Coordination outcomes between β- and α-selective pathways using **M6** as a model monomer calculated at B3LYP-D3/def2svp-SDD­(Ru)-CPCM­(benzene) level of theory. (b) Calculations suggest electron-rich alkoxy side chains increase *k*_p_^app^ in the β pathway consistent with PCD HOMO energies, yet lower *k*_p_^app^ in the α pathway consistent with coordination to Ru.

### Synthesis of Well-Defined PPV Copolymers to Further Tune Optoelectronic Properties

The exquisite levels of regio- and stereoselectivity and unique monomer preferences offered by dithiolate initiator **Ru-1a** prompted us to harness these mechanistic insights to access PPV copolymers with precise structural control. Leveraging the significant reactivity differences between alkoxy- and alkyl-substituted PCDs, we first pursued a one-shot synthesis of regioregular block copolymer *cis*-**P1**-*b*-*cis*-**P4** by combining **M1** and **M4** with **Ru-1a** at room temperature (Figure 8a). As expected from our investigation of the kinetics of the stereoretentive ROMP, ^1^H NMR monitoring revealed full consumption of **M1** after 80 min (Figure 8b), with almost no conversion of **M4** (∼3%). Heating to 40 °C then activated polymerization of **M4**, yielding the desired block copolymer *cis*-**P1**-*b*-*cis*-**P4** in one-shot. SEC analysis showed a unimodal and narrow *Đ* of 1.24 with good agreement between experimental and theoretical *M*_n_ values (*M*_n_^exp^ = 16.7 kg/mol and *M*_n_^theo^ = 15.0 kg/mol). Complete isomerization was then performed in DCM using UV light (350 nm) for 16 h. Comparison of the optical profiles of *cis*-**P1**-*b*-*cis*-**P4** and the photoisomerized product *trans*-**P1**-*b*-*trans*-**P4** revealed significant differences in optoelectronic behavior (Figure 8c). Intriguingly, while the emission profile of *cis*-**P1**-*b*-*cis*-**P4** was nearly identical to that of the more electron-rich homopolymer *cis*-**P1**, *trans*-**P1**-*b*-*trans*-**P4** showed a composite emission profile of *trans*-**P1** and *trans*-**P4**, exemplifying the stark influence of stereochemistry on material properties.^79–82^

**Figure 8:**
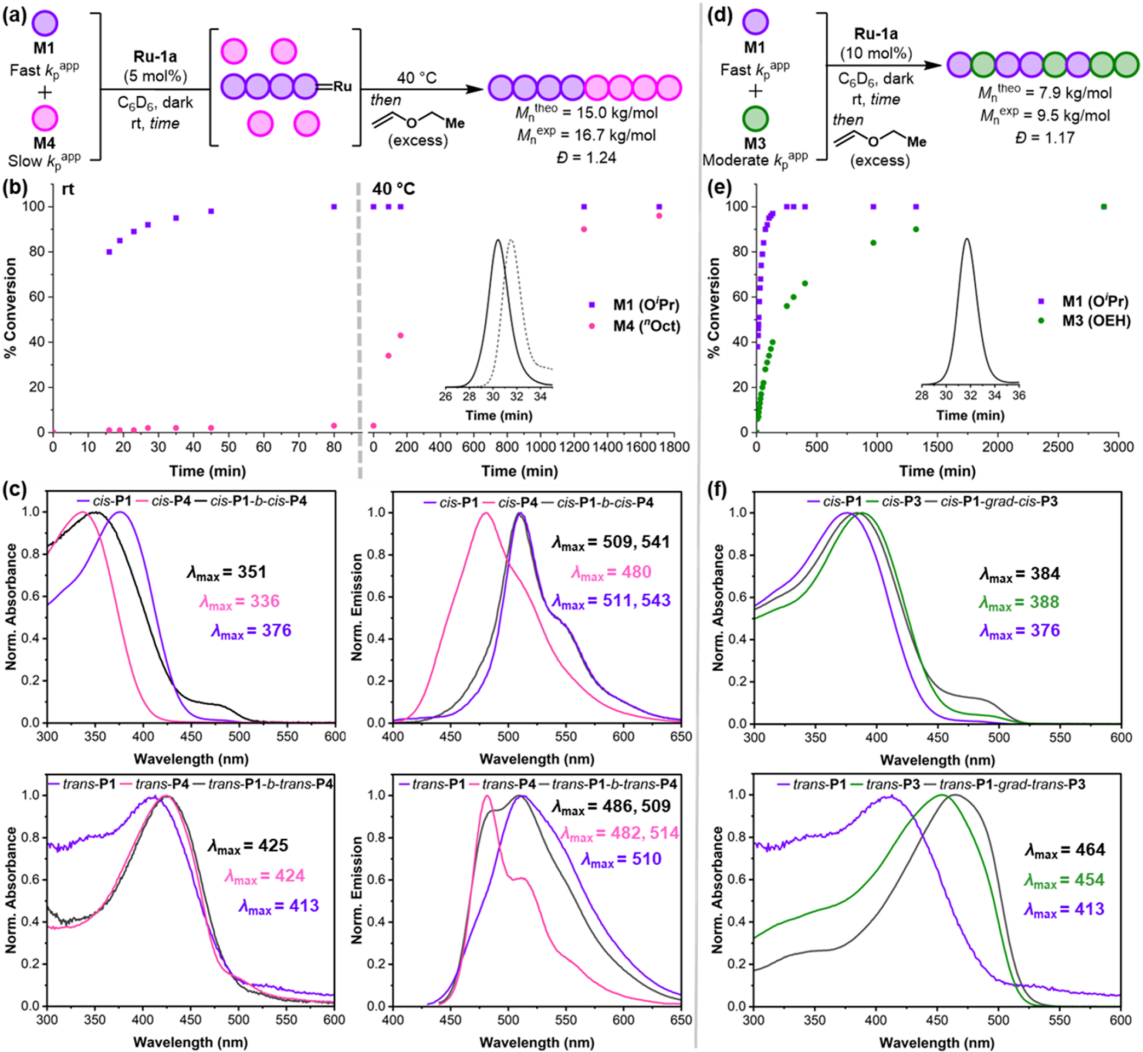
(a) One-shot synthesis of *cis*-**P1**-*b*-*cis*-**P4**. (b) Conversion plot over time (inset shows unimodal SEC trace supporting successful chain extension). (c) Absorbance and emission spectra of *cis*-**P1**-*b*-*cis*-**P4**, *trans*-**P1**-*b*-*trans*-**P4**, and their corresponding homopolymers in CHCl_3_. (d) Synthesis of *cis*-**P1**-*grad*-*cis*-**P3**. (e) Conversion plot over time (inset shows unimodal SEC trace; OEH = 2-ethylhexyloxy). (f) Absorbance spectra of *cis*-**P1**-*grad*-*cis*-**P3**, *trans*-**P1**-*grad*-*trans*-**P3**, and their corresponding homopolymers in CHCl_3_.

Capitalizing on the differences in polymerization rates of **M1** and **M3** with **Ru-1a** (*k*_p_^app^ = 0.0481 and 0.0056 min^–1^, respectively), a gradient copolymerization was also targeted (Figure 8d). ^1^H NMR monitoring and SEC analysis were consistent with a gradient structure enabled by the considerably faster polymerization of **M1** and slower incorporation of **M3** over time (Figure 8e). Only the two propagating carbene signals corresponding to the β-regioisomers of **M1** and **M3** were observed (Figure S49), thereby confirming the formation of a regioregular copolymer *cis*-**P1**-*grad*-*cis*-**P3**. SEC analysis showed a unimodal trace with a narrow *Đ* of 1.17 and good agreement between the experimental and theoretical *M*_n_’s (*M*_n_^exp^ = 9.5 kg/mol and *M*_n_^theo^ = 7.9 kg/mol), indicating a well-controlled copolymerization. Photoisomerization in DCM using UV light (350 nm) delivered *trans*-**P1**-*grad*-*trans*-**P3** after 60 min. Interestingly, while *cis*-**P1**-*grad*-*cis*-**P3** had a λ_max_^abs^ (384 nm) centered near the middle of corresponding all-*cis* homopolymers *cis*-**P1** (376 nm) and *cis*-**P3** (388 nm), all-*trans* congener *trans*-**P1**-*grad*-*trans*-**P3** displayed a bathochromic shift in λ_max_^abs^ (464 nm) compared to all-*trans* homopolymer analogues *trans*-**P1** (413 nm) and *trans*-**P3** (454 nm) (Figure 8f). The narrowing of the optical bandgap energy exhibited by the gradient copolymer is consistent with a previous report by Choi^83^ and further showcases the importance of sequence control for fine-tuning optoelectronic properties.

Finally, we hypothesized that regioregular stereoblock copolymers comprising all-*cis* and all-*trans* segments could be accessed by photoisomerization prior to chain extension, provided that the dithiolate chain-end remains intact during the photoisomerization process. Copolymers exhibiting blocks of *cis* and *trans* stereochemistry are synthetically challenging to access,^84,85^ and to the best of our knowledge, no examples have been reported in the PPV family. Following ROMP of **M3** with **Ru-1a** in THF-*d*_8_ at 40 °C, living all*-cis* **P3** (*M*_n_^theo^ = 2.4 kg/mol, *M*_n_^exp^ = 4.7 kg/mol, *Đ* = 1.28) was irradiated with UV light (350 nm) for 21 h to ensure complete photoisomerization (Figure 9a). Impressively, ^1^H NMR monitoring confirmed the stability of the dithiolate carbene chain-ends (Figure S52), allowing for successful chain extension after the addition of another batch of **M3** (15 equiv) in the dark (Figure 9b). Termination with an excess of ethyl vinyl ether delivered the desired block copolymer *trans*-**P3**-*b*-*cis*-**P3** (*M*_n_^theo^ = 9.3 kg/mol, *M*_n_^exp^ = 11.9 kg/mol, and *Đ* = 1.20). Investigation of the optoelectronic properties showed a wider absorbance profile representing a composite of the two homopolymers, thereby further confirming the isolation of the desired stereoblock copolymer (Figure 9c). In addition to the utility of a broader absorbance window for optimizing energy harvesting in devices, the rod–coil backbone structure^17^ of the PPV stereoblock copolymer may also open the door to further self-assembly applications.^20,86,87^

**Figure 9:**
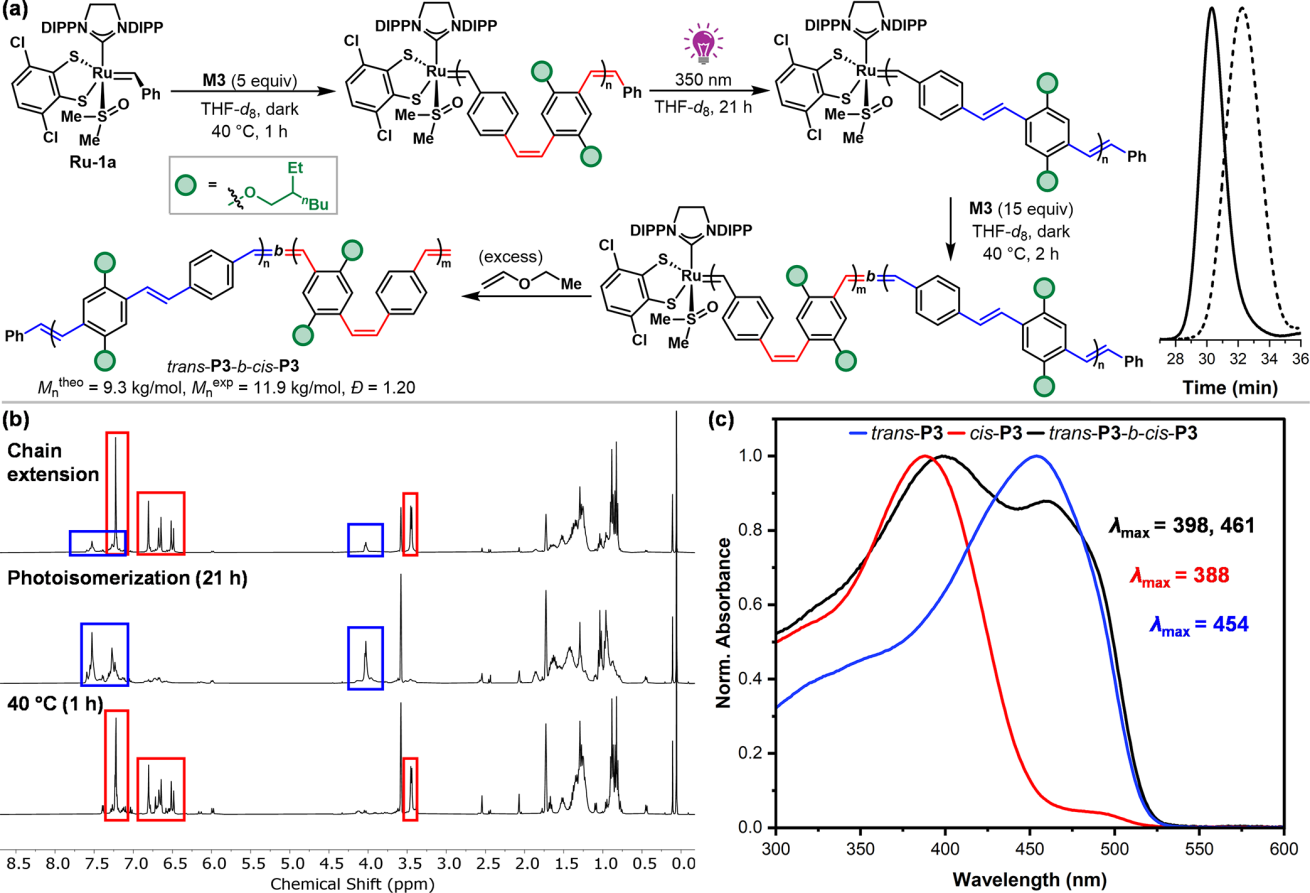
(a) Synthesis of stereoblock copolymer *trans*-**P3**-*b*-*cis*-**P3** and corresponding SEC traces. (b) *In*-*situ* ^1^H NMR monitoring in THF-*d*_8_. (c) Absorbance spectra of *trans*-**P3**, *cis*-**P3**, and *trans*-**P3**-*b*-*cis*-**P3** in CHCl_3_.

Overall, this mechanistically driven investigation delivered five highly controlled regio- and stereodefined PPV copolymers with diverse composition, sequence, and stereochemistry in addition to ten PPV homopolymers. Access to the all-*cis* variants also enabled full characterization of derivatives that were otherwise insoluble for SEC and ^1^H NMR analysis after photoisomerization, including unprecedented *trans*-**P1**, *trans*-**P5**, and *trans*-**P1**-*b*-*trans*-**P4**. The wavelengths corresponding to the absorption and emission maxima of all synthesized PPVs are summarized in Table 3 and the corresponding spectra are included in the Supporting Information. Importantly, the scope of synthesized PPVs exhibited a broad range of λ_max_^abs^ ranging from the UV (336 nm) to visible light (464 nm) regions of the electromagnetic spectrum, in addition to λ_max_^em^ from blue (475 nm) to green (517 nm). This careful modulation of optical bandgaps through precision synthesis further illustrates the potential of controlling all structural features of the polymer to fine-tune the optoelectronic properties in conjugated materials. Furthermore, this exquisite control should facilitate future structure–property relationship studies regarding polymer electronic performance with a variety of side chains in flexible organic electronic devices.

**Table 3:**
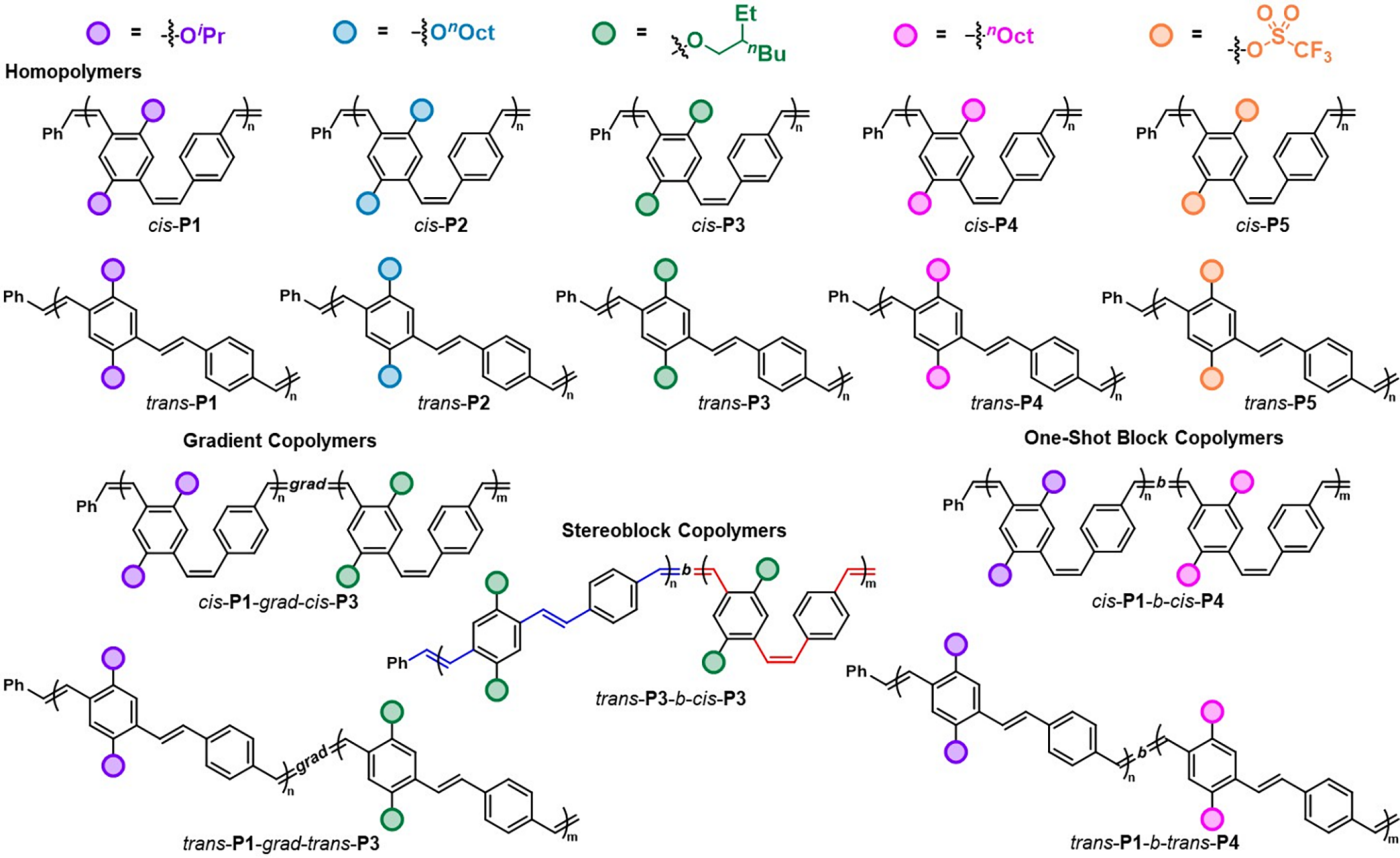
Summary of Absorbance and Emission Data for Synthesized PPVs in CHCl_3_

Entry	Polymer	*M*_n_^exp^ (kg/mol)	*Đ*	λ_max_^abs^ (nm)	λ_max_^em^ (nm)
1	*cis*-**P1**	6.8	1.09	376	511, 543
2	*trans*-**P1**	—^a^	—	413	510
3	*cis*-**P2**	9.9	1.09	387	516, 555
4	*trans*-**P2**	—^a^	—	433	513, 551
5	*cis*-**P3**	8.5	1.08	388	517, 555
6	*trans*-**P3**	20.7	1.18	454	514, 553
7	*cis*-**P4**	8.7	1.16	336	480
8	*trans*-**P4**	—^a^	—	424	482, 514
9	*cis*-**P5**	12.1	1.41	348	475, 507
10	*trans*-**P5**	—^a^	—	407	475, 507
11	*cis*-**P1**-*grad*-*cis*-**P3**	9.5	1.17	384	514, 555
12	*trans*-**P1**-*grad*-*trans*-**P3**	17.1	1.37	464	514, 555
13	*cis*-**P1**-*b*-*cis*-**P4**	16.7	1.24	351	509, 541
14	*trans*-**P1**-*b*-*trans*-**P4**	—^a^	—	425	486, 509
15	*trans*-**P3**-*b*-*cis*-**P3**	11.9	1.20	398, 461	517, 555

a Polymer insolubility in THF prevented SEC analysis.

## Conclusion

In summary, mechanistic investigation of the stereoretentive ROMP of PCDs identified key design principles for accessing regioregular, stereodefined PPV homo- and copolymers. Comprehensive structure–property relationships revealed how structural features of both PCD monomers and dithiolate Ru carbenes govern propagation rates, regio- and stereoselectivity, as well as overall control over the polymerization process. Importantly, the presence of electron-rich alkoxy side chains was shown to drastically increase the rate of propagation with **Ru-1a** and favor high regio- and stereoselectivity, which is promising for the synthesis of precise electron-donor material in optoelectronic devices. Additionally, the rates of polymerization were shown to be influenced by the steric hindrance of the side chain. *In-situ* ^1^H NMR analysis combined with DFT calculations helped rationalize these findings. In particular, the high β-selectivity of **Ru-1a** was found to be crucial to avoid deleterious O-to-Ru coordination from the alkoxy side chains. Instead, these electron-donating motifs increased the rates of propagation, potentially by increasing stabilizing π-interactions with the Ru carbene in the transition state, as suggested by the higher computed HOMO energy. Additionally, well-controlled polymerizations and first-order rate kinetics were observed using **Ru-1a** with most monomers at lower temperatures and shorter reactions times than the other stereoselective catalysts. Finally, the control and selectivity of **Ru-1a** was leveraged to synthesize regio- and stereodefined gradient and one-shot block copolymers, as well as an unprecedented stereoblock PPV through *in*-*situ* photoisomerization followed by chain extension. Overall, this mechanistically guided strategy affords unparalleled control over PPV structure for precise materials design and, more broadly, establishes catalyst design principles for stereoretentive ROMP.

## Supplementary Material



## Notes

Experimental procedures, spectroscopic characterization, and details of computational studies with Cartesian coordinates for the calculated structures (PDF)

## References

[1] Halls J. J. M., Walsh C. A., Greenham N. C., Marseglia E. A., Friend R. H., Moratti S. C., Holmes A. B. (1995). Efficient photodiodes
from interpenetrating polymer networks. Nature.

[2] Yu G., Gao J., Hummelen J. C., Wudl F., Heeger A. J. (1995). Polymer Photovoltaic
Cells: Enhanced Efficiencies via a Network of Internal Donor-Acceptor
Heterojunctions. Science.

[3] Burroughes J. H., Bradley D. D. C., Brown A. R., Marks R. N., Mackay K., Friend R. H., Burns P. L., Holmes A. B. (1990). Light-emitting diodes
based on conjugated polymers. Nature.

[4] Greenham N. C., Moratti S. C., Bradley D. D. C., Friend R. H., Holmes A. B. (1993). Efficient
light-emitting diodes based on polymers with high electron affinities. Nature.

[5] Kulkarni A. P., Tonzola C. J., Babel A., Jenekhe S. A. (2004). Electron Transport
Materials for Organic Light-Emitting Diodes. Chem. Mater..

[6] Grimsdale A.
C., Leok Chan K., Martin R. E., Jokisz P. G., Holmes A. B. (2009). Synthesis
of Light-Emitting Conjugated Polymers for Applications in Electroluminescent
Devices. Chem. Rev..

[7] Yoshida K., Gong J., Kanibolotsky A. L., Skabara P. J., Turnbull G. A., Samuel I. D. W. (2023). Electrically
driven organic laser using integrated
OLED pumping. Nature.

[8] Peters M., Zaquen N., D’Olieslaeger L., Bové H., Vanderzande D., Hellings N., Junkers T., Ethirajan A. (2016). PPV-Based
Conjugated Polymer Nanoparticles as a Versatile Bioimaging Probe:
A Closer Look at the Inherent Optical Properties and Nanoparticle–Cell
Interactions. Biomacromolecules.

[9] Zaquen N., Lu H., Chang T., Mamdooh R., Lutsen L., Vanderzande D., Stenzel M., Junkers T. (2016). Profluorescent PPV-Based Micellar
System as a Versatile Probe for Bioimaging and Drug Delivery. Biomacromolecules.

[10] Kim Y., Cook S., Tuladhar S. M., Choulis S. A., Nelson J., Durrant J. R., Bradley D. D. C., Giles M., McCulloch I., Ha C.-S., Ree M. (2006). A strong regioregularity effect in
self-organizing conjugated polymer films and high-efficiency polythiophene:fullerene
solar cells. Nat. Mater..

[11] Sirringhaus H., Brown P. J., Friend R. H., Nielsen M. M., Bechgaard K., Langeveld-Voss B. M. W., Spiering A. J. H., Janssen R. A. J., Meijer E. W., Herwig P., de Leeuw D. M. (1999). Two-dimensional charge transport
in self-organized, high-mobility conjugated polymers. Nature.

[12] Zaumseil J., Sirringhaus H. (2007). Electron and Ambipolar Transport in Organic Field-Effect
Transistors. Chem. Rev..

[13] Mozer A. J., Denk P., Scharber M. C., Neugebauer H., Sariciftci N. S., Wagner P., Lutsen L., Vanderzande D. (2004). Novel Regiospecific
MDMO–PPV Copolymer with Improved Charge Transport for Bulk
Heterojunction Solar Cells. J. Phys. Chem. B.

[14] Jiang X., Patil R., Harima Y., Ohshita J., Kunai A. (2005). Influences
of Self-Assembled Structure on Mobilities of Charge Carriers in π-Conjugated
Polymers. J. Phys. Chem. B.

[15] Wang G., Swensen J., Moses D., Heeger A. J. (2003). Increased mobility
from regioregular poly­(3-hexylthiophene) field-effect transistors. J. Appl. Phys..

[16] Wang F., He F., Xie Z. Q., Li Y. P., Hanif M., Li M., Ma Y. (2008). Poly­(*p*-phenylene vinylene) Derivatives with Different
Contents of *cis*-Olefins and their Effect on the Optical
Properties. Macromol. Chem. Phys..

[17] Wang F., He F., Xie Z., Li M., Hanif M., Gu X., Yang B., Zhang H., Lu P., Ma Y. (2008). A solution-processible
poly­(*p*-phenylene vinylene) without alkyl substitution:
Introducing the *cis*-vinylene segments in polymer
chain for improved solubility, blue emission, and high efficiency. J. Polym. Sci., Part A:Polym. Chem..

[18] Arai T., Tokumaru K. (1993). Photochemical one-way
adiabatic isomerization of aromatic
olefins. Chem. Rev..

[19] Katayama H., Nagao M., Nishimura T., Matsui Y., Umeda K., Akamatsu K., Tsuruoka T., Nawafune H., Ozawa F. (2005). Stereocontrolled
Synthesis and Optical Properties of All-cis Poly­(phenylene vinylenes)
(PPVs): A Method for Direct Patterning of PPVs. J. Am. Chem. Soc..

[20] Shin S., Menk F., Kim Y., Lim J., Char K., Zentel R., Choi T.-L. (2018). Living Light-Induced Crystallization-Driven
Self-Assembly for Rapid Preparation of Semiconducting Nanofibers. J. Am. Chem. Soc..

[21] Becker H., Spreitzer H., Ibrom K., Kreuder W. (1999). New Insights into the
Microstructure of GILCH-Polymerized PPVs. Macromolecules.

[22] Blayney A. J., Perepichka I. F., Wudl F., Perepichka D. F. (2014). Advances
and Challenges in the Synthesis of Poly­(p-phenylene vinylene)-Based
Polymers. Isr. J. Chem..

[23] Morin J.-F., Drolet N., Tao Y., Leclerc M. (2004). Syntheses and Characterization
of Electroactive and Photoactive 2,7-Carbazolenevinylene-Based Conjugated
Oligomers and Polymers. Chem. Mater..

[24] Pfeiffer S., Hörhold H.-H. (1999). Investigation
of poly­(arylene vinylene)­s, 41. Synthesis
of soluble dialkoxy-substituted poly­(phenylene alkenylidene)­s by applying
the Horner-reaction for condensation polymerization. Macromol. Chem. Phys..

[25] Yang Z., Hu B., Karasz F. E. (1995). Polymer
Electroluminescence Using ac or Reverse dc
Biasing. Macromolecules.

[26] Junkers T., Vandenbergh J., Adriaensens P., Lutsen L., Vanderzande D. (2012). Synthesis
of poly­(*p*-phenylene vinylene) materials *via* the precursor routes. Polym. Chem..

[27] Katayama H., Nagao M., Nishimura T., Matsui Y., Fukuse Y., Wakioka M., Ozawa F. (2006). Stereocontrolled
Synthesis and Characterization
of *cis*-Poly­(arylenevinylene)­s. Macromolecules.

[28] Moslin R. M., Espino C. G., Swager T. M. (2009). Synthesis of Conjugated Polymers
Containing *cis*-Phenylenevinylenes by Titanium-Mediated
Reductions. Macromolecules.

[29] Bunz U. H. F., Mäker D., Porz M. (2012). Alkene Metathesis – A Tool
for the Synthesis of Conjugated Polymers. Macromol.
Rapid Commun..

[30] Mann A., Hannigan M. D., Weck M. (2023). Cyclophanediene and Cyclophanetriene-Based
Conjugated Polymers. Macromol. Chem. Phys..

[31] Zaquen N., Lutsen L., Vanderzande D., Junkers T. (2016). Controlled/living polymerization
towards functional poly­(*p*-phenylene vinylene) materials. Polym. Chem..

[32] Yu C.-Y., Turner M. L. (2006). Soluble Poly­(*p*-phenylenevinylene)­s
through Ring-Opening Metathesis Polymerization. Angew. Chem., Int. Ed..

[33] Elacqua E., Weck M. (2015). Fabrication of Supramolecular Semiconductor Block Copolymers by Ring-Opening
Metathesis Polymerization. Chem. - Eur. J..

[34] Elacqua E., Geberth G. T., Vanden Bout D. A., Weck M. (2019). Synthesis and folding
behaviour of poly­(*p*-phenylene vinylene)-based β-sheet
polychromophores. Chem. Sci..

[35] Mann A., Wang C., Dumlao B. L., Weck M. (2024). Functionalized [2.2]­Paracyclophanedienes
as Monomers for Poly­(*p*-phenylenevinylene)­s. ACS Macro Lett..

[36] Hsu T.-W., Kim C., Michaudel Q. (2020). Stereoretentive
Ring-Opening Metathesis Polymerization
to Access All-*cis* Poly­(*p*-phenylenevinylene)­s
with Living Characteristics. J. Am. Chem. Soc..

[37] Kempel S. J., Hsu T.-W., Michaudel Q. (2021). Stereoretentive
Olefin Metathesis:
A New Avenue for the Synthesis of All-*cis* Poly­(*p*-phenylene vinylene)­s and Stereodefined Polyalkenamers. Synlett.

[38] Khan R. K. M., Torker S., Hoveyda A. H. (2013). Readily Accessible and Easily Modifiable
Ru-Based Catalysts for Efficient and *Z*-Selective
Ring-Opening Metathesis Polymerization and Ring-Opening/Cross-Metathesis. J. Am. Chem. Soc..

[39] Müller D. S., Baslé O., Mauduit M. (2018). A tutorial review of stereoretentive
olefin metathesis based on ruthenium dithiolate catalysts. Beilstein J. Org. Chem..

[40] Hsu T.-W., Kempel S. J., Michaudel Q. (2022). All-*cis* poly­(*p*-phenylene vinylene)­s with high molar masses and fast photoisomerization
rates obtained through stereoretentive ring-opening metathesis polymerization
of [2,2]­paracyclophane dienes with various aryl substituents. J. Polym. Sci..

[41] Mandal H., Ogunyemi O. J., Nicholson J. L., Orr M. E., Lalisse R. F., Rentería-Gómez Á., Gogoi A. R., Gutierrez O., Michaudel Q., Goodson T. III. (2024). Linear and Nonlinear Optical Properties
of All-*cis* and All-*trans* Poly­(*p*-phenylenevinylene). J. Phys. Chem.
C.

[42] Kumar D. R., Lidster B. J., Adams R. W., Turner M. L. (2017). Mechanistic investigation
of the ring opening metathesis polymerisation of alkoxy and alkyl
substituted paracyclophanedienes. Polym. Chem..

[43] Lidster B. J., Kumar D. R., Spring A. M., Yu C.-Y., Turner M. L. (2016). Alkyl substituted
poly­(*p*-phenylene vinylene)­s by ring opening metathesis
polymerisation. Polym. Chem..

[44] Menk F., Mondeshki M., Dudenko D., Shin S., Schollmeyer D., Ceyhun O., Choi T.-L., Zentel R. (2015). Reactivity Studies
of Alkoxy-Substituted [2.2]­Paracyclophane-1,9-dienes and Specific
Coordination of the Monomer Repeating Unit during ROMP. Macromolecules.

[45] Haigh D. M., Kenwright A. M., Khosravi E. (2005). Nature of the Propagating
Species
in Ring-Opening Metathesis Polymerizations of Oxygen-Containing Monomers
Using Well-Defined Ruthenium Initiators. Macromolecules.

[46] Jung I.-S., Lee Y. J., Jeong D., Graf R., Choi T.-L., Son W.-J., Bulliard X., Spiess H. W. (2016). Conformational Analysis
of Oxygen-Induced Higher Ordered Structure of A, B-Alternating Poly­(arylene
vinylene) Copolymers by Solid-State NMR and Molecular Dynamics Simulations. Macromolecules.

[47] Choi T.-L., Han K.-M., Park J.-I., Kim D. H., Park J.-M., Lee S. (2010). High Performance Organic Thin-Film
Transistor based on Amorphous
A,B-Alternating Poly­(arylenevinylene) Copolymers. Macromolecules.

[48] Sariciftci N. S., Smilowitz L., Heeger A. J., Wudl F. (1992). Photoinduced Electron
Transfer from a Conducting Polymer to Buckminsterfullerene. Science.

[49] Sakanoue T., Fujiwara E., Yamada R., Tada H. (2004). Visible light
emission
from polymer-based field-effect transistors. Appl. Phys. Lett..

[50] Michaudel, Q. ; Kempel, S. J. ; Hsu, T.-W. ; deGruyter, J. N. 13.07 – *E* vs *Z* Selectivity in Olefin Metathesis Through Catalyst Design. In Comprehensive Organometallic Chemistry IV; Parkin, G. ; Meyer, K. ; O’hare, D. , Eds.; Elsevier, 2022; pp 265–338.

[51] Hsu T.-W., Kempel S. J., Felix
Thayne A. P., Michaudel Q. (2023). Stereocontrolled
acyclic diene metathesis polymerization. Nat.
Chem..

[52] Kempel S. J., Hsu T.-W., Nicholson J. L., Michaudel Q. (2023). *cis*-Selective Acyclic Diene Metathesis Polymerization of *α*,*ω*-Dienes. J. Am. Chem.
Soc..

[53] Nicholson J. L., Gravet A. C., Michaudel Q. (2026). Telechelic all-*cis* polycyclooctene *via* catalytic stereoretentive ROMP
for the synthesis of polylactide-based ABA triblock copolymers. Faraday Discuss..

[54] Song J.-A., Peterson G. I., Bang K.-T., Ahmed T. S., Sung J.-C., Grubbs R. H., Choi T.-L. (2020). Ru-Catalyzed, *cis*-Selective Living Ring-Opening Metathesis Polymerization of Various
Monomers, Including a Dendronized Macromonomer, and Implications to
Enhanced Shear Stability. J. Am. Chem. Soc..

[55] Keitz B. K., Endo K., Patel P. R., Herbert M. B., Grubbs R. H. (2012). Improved
Ruthenium Catalysts for *Z*-Selective Olefin Metathesis. J. Am. Chem. Soc..

[56] Liu P., Xu X., Dong X., Keitz B. K., Herbert M. B., Grubbs R. H., Houk K. N. (2012). Z-Selectivity in Olefin Metathesis with Chelated Ru
Catalysts: Computational Studies of Mechanism and Selectivity. J. Am. Chem. Soc..

[57] Liu, P. ; Taylor, B. L. H. ; Garcia-Lopez, J. ; Houk, K. N. Computational Studies of Ruthenium-Catalyzed Olefin Metathesis. In Handbook of Metathesis; Wiley, 2015; pp 199–252 10.1002/9783527674107.ch7.

[58] Rosebrugh L. E., Ahmed T. S., Marx V. M., Hartung J., Liu P., López J. G., Houk K. N., Grubbs R. H. (2016). Probing Stereoselectivity
in Ring-Opening Metathesis Polymerization Mediated by Cyclometalated
Ruthenium-Based Catalysts: A Combined Experimental and Computational
Study. J. Am. Chem. Soc..

[59] Rosebrugh L. E., Marx V. M., Keitz B. K., Grubbs R. H. (2013). Synthesis of Highly
Cis, Syndiotactic Polymers via Ring-Opening Metathesis Polymerization
Using Ruthenium Metathesis Catalysts. J. Am.
Chem. Soc..

[60] Mandal I., Kilbinger A. F. M. (2024). Mechanistic
Insights into the *cis*-Selective
Catalytic Ring-Opening Metathesis Polymerization. J. Am. Chem. Soc..

[61] Song J.-A., Park B., Kim S., Kang C., Lee D., Baik M.-H., Grubbs R. H., Choi T.-L. (2019). Living Polymerization
Caught in the Act: Direct Observation of an Arrested Intermediate
in Metathesis Polymerization. J. Am. Chem. Soc..

[62] Jung K., Ahmed T. S., Lee J., Sung J.-C., Keum H., Grubbs R. H., Choi T.-L. (2019). Living
β-selective cyclopolymerization
using Ru dithiolate catalysts. Chem. Sci..

[63] Jung K., Kim K., Sung J.-C., Ahmed T. S., Hong S. H., Grubbs R. H., Choi T.-L. (2018). Toward
Perfect Regiocontrol for β-Selective Cyclopolymerization
Using a Ru-Based Olefin Metathesis Catalyst. Macromolecules.

[64] Hyatt M. G., Walsh D. J., Lord R. L., Andino
Martinez J. G., Guironnet D. (2019). Mechanistic and Kinetic Studies of
the Ring Opening
Metathesis Polymerization of Norbornenyl Monomers by a Grubbs Third
Generation Catalyst. J. Am. Chem. Soc..

[65] Walsh D. J., Lau S. H., Hyatt M. G., Guironnet D. (2017). Kinetic Study
of Living Ring-Opening Metathesis Polymerization with Third-Generation
Grubbs Catalysts. J. Am. Chem. Soc..

[66] Kumar D. R., Lidster B. J., Adams R. W., Turner M. L. (2018). Understanding the
Microstructure of Poly­(*p*-phenylenevinylene)­s Prepared
by Ring-Opening Metathesis Polymerization Using ^13^C-Labeled
Paracyclophanediene Monomers. Macromolecules.

[67] Sato S., Tajima K., Hashimoto K. (2009). Synthesis
and Characterization of
Regioregular Cyano-Substituted Poly­(*p*-phenylenevinylene). Macromolecules.

[68] Suzuki Y., Hashimoto K., Tajima K. (2007). Synthesis of Regioregular Poly­(*p*-phenylenevinylene)­s by Horner Reaction and Their Regioregularity
Characterization. Macromolecules.

[69] Grandner J. M., Shao H., Grubbs R. H., Liu P., Houk K. N. (2017). Origins
of the Stereoretentive Mechanism of Olefin Metathesis with Ru-Dithiolate
Catalysts. J. Org. Chem..

[70] Torker S., Khan R. K. M., Hoveyda A. H. (2014). The Influence
of Anionic Ligands
on Stereoisomerism of Ru Carbenes and Their Importance to Efficiency
and Selectivity of Catalytic Olefin Metathesis Reactions. J. Am. Chem. Soc..

[71] Mikus M. S., Torker S., Hoveyda A. H. (2016). Controllable ROMP Tacticity by Harnessing
the Fluxionality of Stereogenic-at-Ruthenium Complexes. Angew. Chem., Int. Ed..

[72] Bickelhaupt F. M., Houk K. N. (2017). Analyzing Reaction Rates with the Distortion/Interaction-Activation
Strain Model. Angew. Chem., Int. Ed..

[73] Suresh C. H., Koga N. (2004). Orbital Interactions
in the Ruthenium Olefin Metathesis Catalysts. Organometallics.

[74] Scannelli S. J., Paripati A., Weaver J. R., Vu C., Alaboalirat M., Troya D., Matson J. B. (2023). Influence of the Norbornene Anchor
Group in Ru-Mediated Ring-Opening Metathesis Polymerization: Synthesis
of Linear Polymers. Macromolecules.

[75] Radzinski S. C., Foster J. C., Chapleski R. C., Troya D., Matson J. B. (2016). Bottlebrush Polymer
Synthesis by Ring-Opening Metathesis
Polymerization: The Significance of the Anchor Group. J. Am. Chem. Soc..

[76] Scannelli S. J., Alaboalirat M., Troya D., Matson J. B. (2023). Influence of the
Norbornene Anchor Group in Ru-Mediated Ring-Opening Metathesis Polymerization:
Synthesis of Bottlebrush Polymers. Macromolecules.

[77] Wolf W. J., Lin T.-P., Grubbs R. H. (2019). Examining
the Effects of Monomer
and Catalyst Structure on the Mechanism of Ruthenium-Catalyzed Ring-Opening
Metathesis Polymerization. J. Am. Chem. Soc..

[78] Hancock S. N., Yuntawattana N., Valdez S. M., Michaudel Q. (2022). Expedient
synthesis and ring-opening metathesis polymerization of pyridinonorbornenes. Polym. Chem..

[79] Worch J. C., Prydderch H., Jimaja S., Bexis P., Becker M. L., Dove A. P. (2019). Stereochemical enhancement of polymer properties. Nat. Rev. Chem..

[80] Rylski A. K., Cater H. L., Mason K. S., Allen M. J., Arrowood A. J., Freeman B. D., Sanoja G. E., Page Z. A. (2022). Polymeric
multimaterials
by photochemical patterning of crystallinity. Science.

[81] Commisso A. J., Nagel E. M., Kiker M. T., Recker E. A., Bischoff A., Holzmann M. J., Fowler H. E., Pham M. N., Nguyen C. P. H., Baca E. (2026). Lithographic crystallinity regulation in additive
fabrication of thermoplastics (CRAFT). Science.

[82] Nicholson J. L., Michaudel Q. (2026). Controlling
plastic behavior with light. Science.

[83] Lee J., Kim H., Park H., Kim T., Hwang S.-H., Seo D., Chung T. D., Choi T.-L. (2021). Universal
Suzuki–Miyaura Catalyst-Transfer
Polymerization for Precision Synthesis of Strong Donor/Acceptor-Based
Conjugated Polymers and Their Sequence Engineering. J. Am. Chem. Soc..

[84] McGuire T. M., Pérale C., Castaing R., Kociok-Köhn G., Buchard A. (2019). Divergent Catalytic Strategies for the *Cis*/*Trans* Stereoselective Ring-Opening Polymerization
of a Dual Cyclic Carbonate/Olefin Monomer. J.
Am. Chem. Soc..

[85] Ricci G., Pampaloni G., Sommazzi A., Masi F. (2021). Dienes Polymerization:
Where We Are and What Lies Ahead. Macromolecules.

[86] Yang S., Kang S.-Y., Choi T.-L. (2021). Semi-conducting
2D rectangles with
tunable length via uniaxial living crystallization-driven self-assembly
of homopolymer. Nat. Commun..

[87] Yang S., Choi T.-L. (2020). Rapid formation and real-time observation
of micron-sized
conjugated nanofibers with tunable lengths and widths in 20 minutes
by living crystallization-driven self-assembly. Chem. Sci..

