# Supplementary material for: Leveraging Mechanistic Insights into Stereoretentive ROMP for Precision Synthesis of Poly(p‑phenylene vinylene)s
Source: J Am Chem Soc. 2026 May 5;148(19):19943–55. doi: 10.1021/jacs.6c02915 (PMC13195656; doi:10.1021/jacs.6c02915)
Supplement: Supplementary file 1 [file ja6c02915_si_001.pdf]

Supporting Information

Leveraging Mechanistic Insights into Stereoretentive ROMP for Precision Synthesis of Poly(*p*-phenylene vinylene)s

Jake L. Nicholson,[1] Samuel J. Kempel,[1]‡ Ángel Rentería-Gómez,[3]‡ Ting-Wei Hsu,[1] Antoine C. Gravet,[1] Caroline E. Gallo,[1] Osvaldo Gutierrez,[3*] Quentin Michaudel[1,2*]

[1] Department of Chemistry, Texas A&M University, College Station, Texas 77843, United States.

[2] Department of Materials Science and Engineering, Texas A&M University, College Station, Texas, 77843, United States.

[3] Department of Chemistry and Biochemistry, University of California, Los Angeles, Los Angeles, California 90095, United States.

‡S.J.K. and Á.R.-G. contributed equally to this study.

Experimental Procedures

General Reagent Information S5

General Analytical Information S5

Monomer Synthesis S6

Sequence for Monomer **M1** S6

Monomers **M2**–**M4** S11

Monomer **M5** S12

Polymerization Kinetics Experiments S13

General Kinetics Procedure A using Ru Catalysts S13

General Kinetics Procedure B using **Ru-2** and **3-ClPy** S14

Determination of Propagation and Initiation Kinetics S14

^{1}H NMR Kinetic Studies for **P1** S15

Figure S1. ^{1}H NMR spectra using **Ru-1a** and **M1** S15

Figure S2. ^{1}H NMR spectrum using **Ru-2** and **M1** S16

Figure S3. ^{1}H NMR spectra using **Ru-2 + 3-ClPy** (1:1.2) and **M1** S16

Figure S4. ^{1}H NMR spectra using **Ru-2 + 3-ClPy** (1:2.7) and **M1** S17

Figure S5. ^{1}H NMR spectra using **Ru-2 + 3-ClPy** (1:4.8) and **M1** S17

Figure S6. ln([**3-ClPy**]) vs. ln(k_p^{app}) at constant Ru loading S18

Figure S7. ^{1}H NMR spectrum of **M1** polymerization 1:1 **Ru-1a**-to-**3-ClPy** S18

Figure S8. ^{1}H NMR spectra using **Ru-3a** and **M1** S19
Figure S9. Alkylidene decomposition using **Ru-3a** and **M1** S19
^{1}H NMR Kinetic Studies for **P2** S20
Figure S10. ^{1}H NMR spectra using **Ru-1a** and **M2** S20
Figure S11. ^{1}H NMR spectrum using **Ru-2** and **M2** S21
Figure S12. ^{1}H NMR spectra using **Ru-2 + 3-ClPy** and **M2** S21
Figure S13. ^{1}H NMR spectra using **Ru-3a** and **M2** S22
Figure S14. Alkylidene decomposition using **Ru-3a** and **M2** S22
^{1}H NMR Kinetic Studies for **P3** S23
Figure S15. ^{1}H NMR spectra using **Ru-1a** and **M3** S23
Figure S16. ^{1}H NMR spectra using **Ru-1b** and **M3** S24
Figure S17. ^{1}H NMR spectrum using **Ru-2** and **M3** S24
Figure S18. ^{1}H NMR spectrum using **Ru-2** and **M3** in THF-d_8 .... S25
Figure S19. ^{1}H NMR spectra using **Ru-2 + 3-ClPy** and **M3** S25
Figure S20. ^{1}H NMR spectra using **Ru-3a** and **M3** S26
Figure S21. Alkylidene decomposition using **Ru-3a** and **M3** S26
Figure S22. ^{1}H NMR spectra using **Ru-3b** and **M3** S27
Figure S23. ^{1}H NMR spectra using **GII** and **M3** S27
Figure S24. ^{1}H NMR spectra using **GIII** and **M3** S28
^{1}H NMR Kinetic Studies for **P4** S29
Figure S25. ^{1}H NMR spectra using **Ru-1a** and **M4** S29
Figure S26. ^{1}H NMR spectra for initiation monitoring using **Ru-1a** and **M4** S30
Figure S27. Initiation kinetics plot of **Ru-1a** and **M4** S30
Figure S28. ^{1}H NMR spectra using **Ru-2** and **M4** S31
Figure S29. ^{1}H NMR spectra using **Ru-2 + 3-ClPy** and **M4** S31
^{1}H NMR Kinetic Studies for **P5** S32
Figure S30. ^{1}H NMR spectra using **Ru-1a** and **M5** S32
Figure S31. Alkylidene region using **Ru-1a** and **M5** S33
Figure S32. ^{1}H NMR spectra using **Ru-2** and **M5** S33
Figure S33. Alkylidene region using **Ru-2** and **M5** S34
Figure S34. ^{1}H NMR spectra using **Ru-2 + 3-ClPy** and **M5** S34
Summary of the Reaction Kinetics Performed using *in-situ* ^{1}H NMR Spectroscopy S35
Figure S35. Overlayed Conversion Plots of **M1–M5** using **Ru-2** S35
Figure S36. Overlayed Kinetics Plots of **M1–M5** using **Ru-2 + 3-ClPy** S35

Figure S37. Overlayed Conversion Plots of **M1**–**M3** using **Ru-3a** S36

Table S1. Polymerization data using **Ru-2**, **Ru-2 + 3-ClPy**, and **Ru-3a** S36

Diagnostic ^{1}H NMR Alkylidene Signals S37

Figure S38. Alkylidene signals using **Ru-1a** with **M1**–**M4** S37

Figure S39. Alkylidene signals using **Ru-2** with **M2** and **M3** S38

Figure S40. Alkylidene signals using **Ru-2 + 3-ClPy** with **M1**–**M4** S39

Figure S41. Alkylidene signals using **Ru-1b** with **M3** S40

Ring-Opening Metathesis Procedures S41

Determination of Regioselectivity through Ring-Opening Metathesis of **M1** with **Ru-1a** S41

Figure S42. Full NOESY spectrum using 1 equiv. of **Ru-1a** with **M1** S41

Figure S43. Zoomed in NOESY spectrum using 1 equiv. of **Ru-1a** with **M1** S42

Figure S44. ^{1}H NMR spectrum using 1 equiv. of **Ru-1a** with **M1** S42

Copolymer Synthesis S43

One-Shot Synthesis of *cis*-**P1**-*b*-*cis*-**P4** S43

Figure S45. ^{1}H NMR spectra of polymer conversion over time S44

Figure S46. SEC traces showing chain extension after heating S45

Figure S47. *cis*-**P1**, *cis*-**P4**, and *cis*-**P1**-*b*-*cis*-**P4** ^{1}H NMR spectra S45

Synthesis of *cis*-**P1**-*grad*-*cis*-**P3** S46

Figure S48. ^{1}H NMR spectra of polymer conversion over time S47

Figure S49. ^{1}H NMR spectra of alkylidene signal shifts over time S47

Figure S50. *cis*-**P1**, *cis*-**P3**, and *cis*-**P1**-*grad*-*cis*-**P3** ^{1}H NMR spectra S48

Stereodefined Synthesis of *trans*-**P3**-*b*-*cis*-**P3** S49

Figure S51. Photoisomerization monitoring in THF-d_8 .... S50

Figure S52. Alkylidene stability during photoisomerization in THF-d_8 .... S51

Figure S53. *trans*-**P3**, *cis*-**P3**, and *trans*-**P3**-*b*-*cis*-**P3** ^{1}H NMR spectra S52

General Photoisomerization Procedure to Access All-*trans* PPV S52

Absorption and Emission Spectra S53

Figure S54. Absorbance/emission traces for *cis*-**P1** and *trans*-**P1** S53

Figure S55. Absorbance/emission traces for *cis*-**P2** and *trans*-**P2** S53

Figure S56. Absorbance/emission traces for *cis*-**P3** and *trans*-**P3** S54

Figure S57. Absorbance/emission traces for *cis*-**P4** and *trans*-**P4** S54

Figure S58. Absorbance/emission traces for *cis*-**P5** and *trans*-**P5** S55

Figure S59. Absorbance/emission traces for *cis*-**P1**-*grad*-*cis*-**P3** and *trans*-**P1**-*grad*-*trans*-**P3**. S55

Figure S60. Absorbance/emission traces for *cis*-**P1**-*b*-*cis*-**P4** and *trans*-**P1**-*b*-*trans*-**P4** S56

Figure S61. Absorbance/emission traces for *trans*-**P3**-*b*-*cis*-**P3** S56
Supporting NMR Spectra S57
Computational Details S69
Figure S86. Possible transition states for reactions of **M0** with **Ru-1a** S70
Figure S87. Side-bound vs. bottom-bound for reactions of **M0** with **Ru-1a** S70
Figure S88. Calculated potential energy surface using **M0** with **Ru-1a** S71
Figure S89. Calculated potential energy surface using **N0** with **Ru-1a** S71
Figure S90. NCI analysis of **TS1** S72
Table S2. Single point energies for distortion/interaction analysis of **Ru-1a**/**M2** S72
Figure S91. Favorable transition state energies for **Ru-1b** and **Ru-1a** with **M**-OMe S73
Table S3. Single point energies for distortion/interaction analysis of **Ru-1b**/**M**-OMe S73
Table S4. Single point energies for distortion/interaction analysis of **Ru-1a**/**M**-OMe S73
Figure S92. Conformational search of truncated **M2**-O^{n}Oct with **Ru-1a** S74
Figure S93. Calculated potential energy surface using **Ru-1b**/**M**-OMe S75
Figure S94. Calculated potential energy surface using **Ru-1a**/**M**-OMe S75
Figure S95. Possible transition states for reactions of **M**-OMe with **GII/III** S76
Figure S96. Calculated potential energy surface using **M**-OMe with **GII/III** S76
Table S5. Cartesian coordinates and single-point energies S77
References S307

General Reagent Information: All reactions were carried out under an inert nitrogen atmosphere with dry solvents under anhydrous conditions unless otherwise stated. Dry dichloromethane (DCM), diethyl ether (Et_2O), tetrahydrofuran (THF), and toluene (PhMe) were obtained by passing the previously degassed solvents through activated alumina columns. Anhydrous C_6D_6 and THF-d_8 were degassed via "freeze-pump-thaw" before being brought into a nitrogen-filled glove box. Synthesis of polymers using stereoselective ruthenium catalysts were carried out in a nitrogen-filled glove box (SG1800/750TS-F, VIGOR). Reagents were purchased at the highest commercial quality and used without further purification, unless otherwise stated. Ruthenium catalysts were generously donated by Umicore. Yields refer to chromatographically and spectroscopically (^{1}H NMR) homogeneous material, unless otherwise stated. Reactions were monitored by thin layer chromatography (TLC) carried out on 250 µm SiliCycle SiliaPlate™ silica plates (F254), using UV light as the visualizing agent. Flash silica gel chromatography was performed using SiliCycle SiliaFlash® Irregular Silica Gel (60 Å, particle size 40–63 µm). The polymer was isolated after precipitation using an Eppendorf 5804 centrifuge.

General Analytical Information: All polymer samples were analyzed using a Tosoh EcoSec HLC 8320GPC system with a TSKgel SuperHM-M column and a TSKgel SuperH-RC column at flow rates of 0.10 and 0.40 mL/min at 40 °C. THF stabilized with BHT was used as the eluent and the number-average molecular weight (M_n), weight-average molecular weight (M_w), and dispersity ($Đ$) for the polymer were calculated from an RI chromatogram against a TSKgel polystyrene standard. NMR spectra were recorded on Bruker Avance Neo 400 and Bruker Avance 500 instruments and were calibrated using residual undeuterated solvent as an internal reference ($CHCl_3$ @ 7.26 ppm ^{1}H NMR, 77.16 ppm ^{13}C NMR). The following abbreviations were used to explain NMR peak multiplicities: s = singlet, d = doublet, dd = doublet of doublets, t = triplet, q = quartet, quin. = quintet, hep. = heptet m = multiplet, br = broad. High-resolution mass spectra (HRMS) were recorded on an Agilent LC/MSD TOF mass spectrometer by Atmospheric pressure chemical ionization (APCI) or electrospray ionization (ESI). The absorption and emission measurements were carried out in dilute chloroform solutions. Absorption spectra were collected on a Shimadzu UV-2600 UV-visible spectrophotometer using 1.0 cm path length quartz cuvettes. Fluorescence measurements were collected on a Horiba FluoroMax 4 spectrofluorometer with entrance and exit slits set to 5.0 nm and an integration time of 0.1 s.

Monomer Synthesis:

Sequence for Monomer **M1**

Compound **S1a**

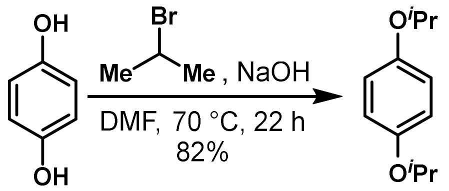

S1a

Hydroquinone (6.6 g, 60 mmol, 1 equiv) and freshly crushed NaOH (7.2 g, 180 mmol, 3 equiv) were weighed into a flame-dried round bottom flask equipped with a stir bar and addition funnel. The apparatus was placed under vacuum and backfilled with N_2 three times before anhydrous DMF* (60 mL) was added under N_2. Then, 2-bromopropane (22 g, 17 mL, 180 mmol, 3 equiv) was added dropwise via addition funnel to the stirring hydroquinone solution. After complete addition, the round bottom flask was lowered into an oil bath preheated to 70 °C and stirred at this temperature for 22 h. Next, the reaction was allowed to cool to room temperature (rt) and H_2O (100 mL) was added. The mixture was transferred to a separatory funnel where the organics were extracted with EtOAc (150 mL). Upon separating the organic layer, it was washed with H_2O (3x 50 mL), saturated NH_4Cl (aq.) (50 mL), and brine (50 mL). The aqueous layers were combined and washed once more with EtOAc (50 mL). The organic layers were then combined and dried over $MgSO_4$. The $MgSO_4$ was filtered off and the solvent was removed under reduced pressure. The residue was then purified through column chromatography (SiO_2; 9:1 Hexanes:EtOAc) to give the product as a clear oil (9.5 g, 82%).

The spectroscopic data for this compound were identical to those reported in the literature.[1]

^{1}H NMR (400 MHz, $CDCl_3$) δ 6.81 (s, 4 H), 4.47–4.35 (hep, J = 6.1 Hz, 2 H), 1.33–1.28 (d, J = 6.1 Hz, 12 H) ppm.

*NOTE: The reaction can be performed with no precautions for removing air and water with a small drop in yield (71% instead of 82%).

Compound **S1b**

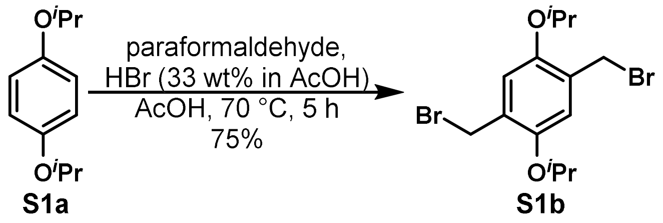

Compound **S1a** (8.00 g, 41 mmol, 1 equiv) and paraformaldehyde (4.94 g, 165 mmol, 4 equiv) were weighed under air into a 500 mL round bottom flask equipped with a stir bar. Glacial acetic acid (165 mL) was added to the flask, followed quickly by the addition of HBr (25 mL, 33 wt% in AcOH). The flask was fitted with a waterless condenser and Ar balloon and stirred for 5 h at 70 °C before cooling to rt. Upon cooling, a white solid precipitated in the reaction mixture. The contents were poured into 200 mL of ice water where more white solid precipitated. The solid was filtered using a frit funnel and further washed with cold MeOH (3 x 20 mL washes). The solid was then dried under reduced pressure to give the product **S1b** as a white solid (11.7 g, 75%).

^{1}H NMR (400 MHz, $CDCl_3$) δ 6.87 (s, 2 H), 4.51 (s, 4 H), 4.57–4.47 (hep, J = 6.0 Hz, 2 H), 2.12–2.01 (d, J = 6.0 Hz, 12 H) ppm.

^{13}C NMR (101 MHz, $CDCl_3$) δ 149.6, 128.8, 116.8, 71.5, 29.0, 22.4 ppm.

HRMS-(+)ESI: calc'd. for $C_{14}H_{28}O_2Br_2$ $[M + NH_4]^+$ 398.0148, found 398.0143

Compound **S1c**

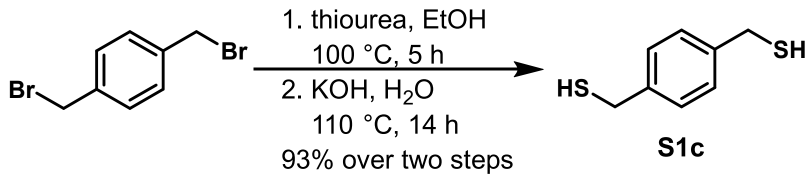

Compound **S1c** was synthesized following literature procedure reported by Turner and co-workers and the spectroscopic data for this compound were identical to those in the literature.[2]

^{1}H NMR (400 MHz, $CDCl_3$) δ 7.28 (s, 4 H), 3.73 (d, J = 7.5 Hz, 4 H), 1.75 (t, J = 7.5 Hz, 2 H) ppm.

Compound **S1d**

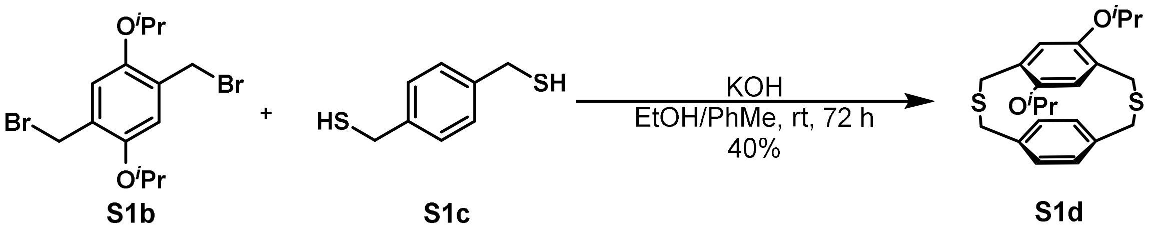

KOH (85 wt%, 3.96 g, 60 mmol, 3 equiv) was weighed into a 2L round bottom flask equipped with a stir bar and subsequently dissolved in EtOH (950 mL). Meanwhile, compound **S1b** (7.60 g, 20 mmol, 1 equiv) was dissolved in PhMe (190 mL) and compound **S1c** (3.40 g, 20 mmol, 1 equiv) was also dissolved in PhMe (190 mL). Using a syringe pump, each PhMe solution was added over three days at rt to the reaction flask, followed by an additional 6 h of stirring after complete addition. The solvent was removed under reduced pressure, and the resulting residue was dissolved in DCM (200 mL) and washed with water (2 x 50 mL) followed by brine (40 mL). The organic layer was separated and dried over $MgSO_4$. The solvent was then removed under reduced pressure and the resulting yellow oil was purified through column chromatography (SiO_2; Hexanes to 3:2 Hexanes to DCM) to give the product **S1d** as a white solid (3.09 g, 40%).

^{1}H NMR (500 MHz, $CDCl_3$) δ 7.02–6.96 (m, 2 H), 6.95–6.89 (m, 2 H), 6.42 (s, 2 H), 4.34–4.26 (m, 2 H), 4.24 (d, J = 14.6 Hz, 2 H), 3.86–3.78 (m, 2 H), 3.78–3.71 (m, 2 H), 3.31 (d, J = 14.8 Hz, 2 H), 1.47 (d, J = 5.9 Hz, 6 H), 1.23 (d, J = 6.0 Hz, 6 H) ppm.

^{13}C NMR (126 MHz, $CDCl_3$) δ 148.9, 135.7, 129.0, 128.2, 126.1, 116.7, 70.7, 38.1, 31.4, 23.0, 22.2 ppm.

HRMS-(+)ESI: calc'd. for $C_{22}H_{28}O_2S_2$ $[M+H]^+$ 389.1603, found 389.1596

Compound **S1e**

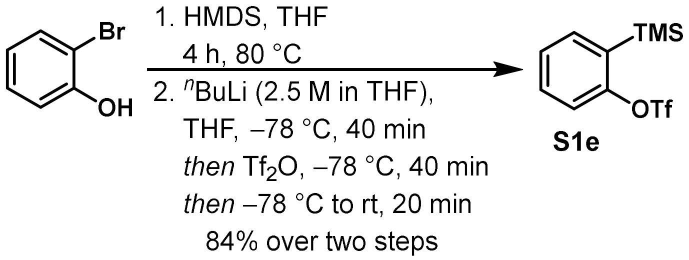

Compound **S1e** was synthesized following literature procedure reported by Turner and co-workers and the spectroscopic data were identical to those in the literature.[2]

^{1}H NMR (400 MHz, $CDCl_3$) δ 7.54 (dd, *J* = 7.5, 1.9 Hz, 1 H), 7.44 (ddd, *J* = 8.3, 7.3, 1.9 Hz, 1 H), 7.38–7.31 (m, 2 H), 0.37 (s, 9 H) ppm.

Compound **S1f**

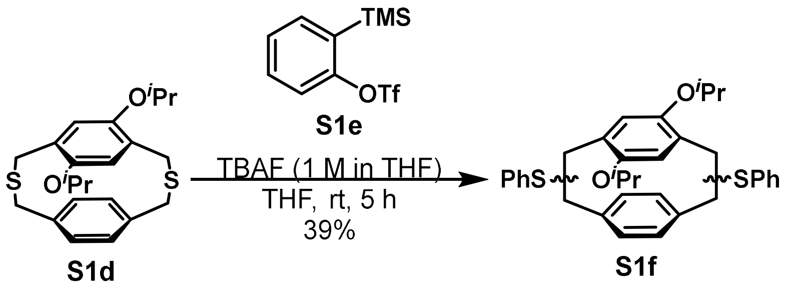

Compound **S1d** (2.5 g, 6.4 mmol, 1 equiv) and compound **S1e** (4.2 g, 14.2 mmol, 2.2 equiv) were weighed into a flame-dried round-bottom flask equipped with a stir bar, followed by three nitrogen/vacuum cycles. Anhydrous THF (100 mL) was added to dissolve the contents. Then, TBAF (1 M in THF, 19.3 mL, 3 equiv) was added to the solution over the period of 4 h at rt with the aid of a syringe pump. After complete addition of TBAF, the reaction was allowed to stir for an additional hour. The organic solvent was then removed *in vacuo* and the oil was purified through column chromatography (SiO_2; Hexanes to 7:3 Hexanes:DCM) to give the product **S1f** as a mixture of stereo- and regioisomers as a yellow oil (1.39 g, 39%).

^{1}H NMR (400 MHz, $CDCl_3$) characteristic peaks: δ 7.40–7.30 (m, 2), 7.17–7.07 (m), 6.99–6.87 (m), 6.88–6.30 (m), 6.44–6.25 (m), 6.20–6.17 (m), 6.12–6.02 (m), 5.90–5.85 (m), 5.81 (s), 5.74(s), 5.73–5.64 (m), 5.47 (s), 5.23 (dd, *J* = 4.5, 5.2 Hz), 4.77 (q, *J* = 8.4 Hz), 4.54 (dd, *J* =10.2, 5.2 Hz), 4.20–4.00 (m), 3.87–3.70 (m), 3.69–3.60 (m), 3.34–3.12 (m), 3.11–3.01 (m), 2.66 (dd, *J* = 13.7, 4.6 Hz), 2.50 (tt, *J* = 12.0, 6.6 Hz), 2.30–2.12 (m), 1.50–1.45 (m), 1.42–1.27 (m), 1.23–1.09 (m), 1.07 (d, *J* = 6.0 Hz) ppm.

HRMS-(+)ESI: calc'd. for $C_{34}H_{36}O_2S_2$ $[M+H]^+$ 541.2229, found 541.2221.

Monomer **M1**

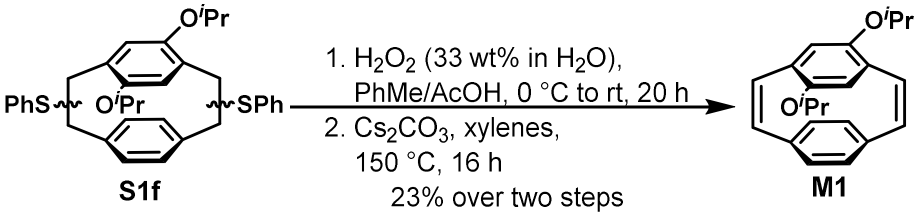

Compound **S1f** (1.3 g, 2.4 mmol, 1.0 equiv) was transferred with the aid of PhMe (28 mL) under air into a round bottom flask equipped with a stir bar. AcOH (14 mL) was added, and the solution was cooled to 0 °C using an ice/water bath. Next, an H_2O_2 solution (33 wt% in water, 0.80 mL) was added dropwise over 30 min using a syringe pump. The reaction was allowed to warm to rt and stirred for 20 h. DCM (80 mL) was added, and the mixture was transferred to a separatory funnel. Brine (40 mL) was added and the organic layer was separated. The organic layer was subsequently washed with two more portions of brine (40 mL) before drying over $MgSO_4$. The mixture was filtered, and the pale-yellow solution was concentrated under reduced pressure and further dried under high vacuum for 14 h to give a pale yellow solid. Cs_2CO_3 (3.16 g, 9.7 mmol, 4.3 equiv) was quickly added into a flame-dried round bottom flask equipped with a stir bar and a waterless condenser, followed by three nitrogen/vacuum cycles. Xylenes (49 mL) dried over activated 4 Å molecular sieves for 24 h were then used to dissolve the oxidized intermediate (1.29 g, 2.26 mmol, 1 equiv). The solution was transferred to the flask containing Cs_2CO_3 and the mixture was heated at 150 °C for 16 h to give a bright yellow solution. The reaction was cooled to rt before removing solvent under reduced pressure. The mixture was dissolved in DCM (100 mL) and transferred to a separatory funnel. The yellow solution was washed carefully with 2 x 30 mL portions of 1 M HCl. The organic layer was then washed with water (40 mL) and brine (40 mL). The organic layer was separated and dried over $MgSO_4$. The solvent was removed under reduced pressure to give a yellow-orange oil that was further purified through column chromatography (SiO_2; 3:2 Hexanes:DCM) to give the product **M1** as a yellow semisolid (168 mg, 23%).

^{1}H NMR (500 MHz, $CDCl_3$) δ 7.11 (d, J = 10.2 Hz, 2 H), 6.89 (dd, J = 7.9, 1.8 Hz, 2 H), 6.85 (d, J = 10.1 Hz, 2 H), 5.79 (s, 2 H), 4.16 (hep, J = 6.1 Hz, 2 H), 1.26 (dd, J = 12.6, 6.1 Hz, 12 H) ppm.

^{13}C NMR (126 MHz, $CDCl_3$) δ 152.2, 138.3, 135.7, 134.3, 131.5, 128.9, 127.5, 123.4, 72.7, 22.7, 22.6 ppm.

HRMS-(+)ESI: calc'd. for $C_{22}H_{24}O_2$ $[M+H]^+$ 321.1849, found 321.1844.

Monomers **M2–M4**

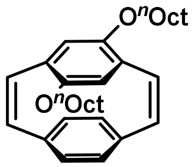

M2

Monomer **M2** and its precursors were synthesized following literature procedures reported by Michaudel and co-workers and the spectroscopic data were identical to those in the literature.[3]

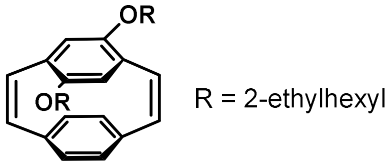

M3

Monomer **M3** and its precursors were synthesized following literature procedures reported by Goodson III, Michaudel, and co-workers and the spectroscopic data were identical to those in the literature.[4]

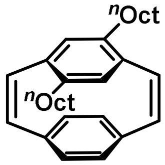

M4

Monomer **M4** and its precursors were synthesized following literature procedures reported by Michaudel and co-workers and the spectroscopic data were identical to those in the literature.[3]

Monomer **M5**

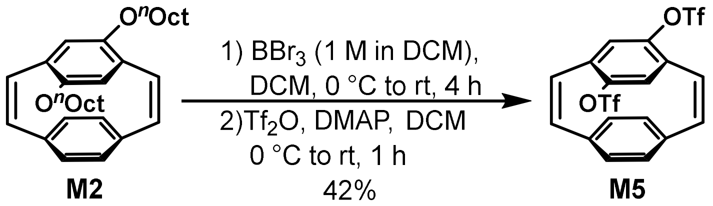

To a flame-dried 50 mL round-bottom flask equipped with a stir bar, **M2** (370 mg, 0.8 mmol, 1.0 equiv) was quickly added followed by three nitrogen/vacuum cycles. Following this, anhydrous DCM (8.0 mL) was added, and the solution was cooled to 0 °C in an ice water bath. 1 M BBr_3 in DCM (6.4 mL, 6.4 mmol, 8.0 equiv) was then added to the solution under nitrogen. The reaction flask was removed from the ice water bath and left to react at room temperature for 4 hours. The reaction was quenched with water, and the mixture was extracted using DCM (50 mL). The organic layer was dried with Na_2SO_4 and concentrated under reduced pressure. The resulting intermediate was then quickly added to a flame-dried 50 mL round bottom flask containing a stir bar and DMAP (668 mg, 5.5 mmol, 6.0 equiv). After three nitrogen/vacuum cycles, anhydrous DCM (10.0 mL) was added, and the solution was cooled to 0 °C in an ice water bath. Tf_2O (0.6 mL, 3.6 mmol, 4.0 equiv) was then added dropwise under nitrogen. The reaction flask was removed from the ice bath and left to react at rt for 1 hour. The crude product was filtered and dissolved using DCM (50 mL). The organic layer was washed with water, and the aqueous layer was reextracted using DCM (50 mL). The combined organic layers were dried with Na_2SO_4 and concentrated under reduced pressure. Purification via column chromatography (SiO_2; Hexanes, 2:1 Hexanes:DCM) afforded the desired monomer **M5** as a white solid (169 mg, 42%).

^{1}H NMR (500 MHz, $CDCl_3$) δ 7.39 (d, J = 10.4 Hz, 2 H), 6.93 (dd, J = 8.1 Hz, 1.3 Hz, 2 H); 6.86 (d, J = 10.4 Hz, 2 H), 6.58 (dd, J = 8.1 Hz, 1.3 Hz, 2 H), 6.38 (s, 2 H) ppm.

^{13}C NMR (126 MHz, $CDCl_3$) δ 146.4, 139.7, 137.6, 134.6, 131.6, 130.5, 129.1, 127.5, 122.6, 120.0, 117.5, 114.9 ppm.

^{19}F NMR (376 MHz, $CDCl_3$) δ –72.83 ppm.

HRMS-APCI: calc'd. for $C_{18}H_{11}F_6O_6S_2$ $[M+H]^+$ 500.9896, found 500.9879.

Polymerization Kinetics Experiments

General Kinetics Procedure A using Ru Catalysts

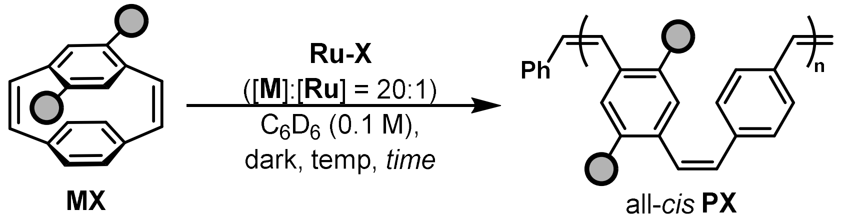

Inside a nitrogen-filled glovebox, 5 mol% of **Ru-1a** (2.2 mg, 0.0025 mmol), **Ru-1b** (2.0 mg, 0.0025 mmol), **Ru-2** (2.1 mg, 0.0025 mmol), **Ru-3a** (1.7 mg, 0.0025 mmol), **Ru-3b** (1.6 mg, 0.0025 mmol), **GII** (2.2 mg, 0.0025 mmol), or **GIII** (2.2 mg, 0.0025 mmol) was weighed into a vial. Next, monomer **M1** (16.0 mg, 0.05 mmol), **M2** (23.1 mg, 0.05 mmol), **M3** (23.1 mg, 0.05 mmol), **M4** (21.4 mg, 0.05 mmol), or **M5** (25.0 mg, 0.05 mmol) was dissolved in C_6D_6 (0.5 mL) and quickly transferred to the vial containing the measured catalyst. For catalyst screening, a hexamethyldisilane stock solution (2.2 mg dissolved in 0.6 mL of C_6D_6) was prepared, from which a measured amount (0.1 mL, 0.0025 mmol, 1.0 equiv) was combined with C_6D_6 (0.4 mL) for a consistent reaction volume (0.5 mL). After quickly mixing, the reaction solutions were transferred into a J-Young tube and sealed while inside the glovebox. All reactions were monitored *in-situ* by ^{1}H NMR while running at either rt (**Ru-1a** with **M1–M3**, **Ru-2**, **GII**, and **GIII**), 40 °C (**Ru-1a** with **M4–M5**, **Ru-1b**, and **Ru-3b**) or 70 °C (**Ru-3a**) in the dark. The reactions were quenched inside a nitrogen-filled glovebox using excess ethyl vinyl ether (0.1 mL). After leaving to sit for 30 minutes at rt, two cycles of precipation with addition of methanol (~10 mL), centrifugation, and decantation afforded polymers **P1**–**P5** with residual solvent. All polymer vials were wrapped in aluminum foil, dried under reduced pressure followed by high vacuum, and stored in the dark under a nitrogen atmosphere at –20 °C to prevent any undesired photoisomerization. Polymerization using THF-d_8 (0.5 mL) instead of C_6D_6 was performed following the same procedure.

General Kinetics Procedure B using **Ru-2** and **3-ClPy**

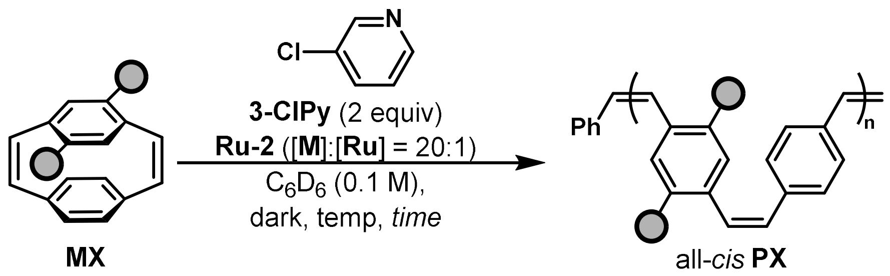

Inside a nitrogen-filled glovebox, 5 mol% of **Ru-2** (2.1 mg, 0.0025 mmol) was weighed into a vial. A stock solution of **3-ClPy** (28 mg) in C_6D_6 (1.0 mL) was prepared. Next, monomer **M1** (16.0 mg, 0.05 mmol), **M2** (23.1 mg, 0.05 mmol), **M3** (23.1 mg, 0.05 mmol), **M4** (21.4 mg, 0.05 mmol), or **M5** (25.0 mg, 0.05 mmol) was dissolved in C_6D_6 (0.48 mL) and **3-ClPy** stock solution (20 μL) was added with a microsyringe. The solution was transferred to the vial containing the measured catalyst. After quickly mixing, the solution was transferred into a J-Young tube and sealed while inside the glovebox. The reactions were monitored *in-situ* by ^{1}H NMR while running at rt (**M1**–**M3**) or 40 °C (**M4**–**M5**) in the dark. The reactions were quenched inside a nitrogen-filled glovebox using excess ethyl vinyl ether (0.1 mL). After leaving to sit for 30 minutes at rt, two cycles of precipation with addition of methanol (~10 mL), centrifugation, and decantation afforded polymers **P1**–**P5** with residual solvent. All polymer vials were wrapped in aluminum foil, dried under reduced pressure followed by high vacuum, and stored in the dark under a nitrogen atmosphere at –20 °C to prevent any undesired photoisomerization.

Determination of Propagation and Initiation Rates

k_p^{app} and k_i^{app} values were determined from the linear fit of $\ln([M]_0/[M]_t)$ and $\ln([I]_0/[I]_t)$, respectively, plotted as a function of time based on the following equations

$$-\frac{d[\mathrm{M}]}{dt} = k_\mathrm{p}^\mathrm{app}[\mathrm{M}]_\mathrm{t} \qquad (1)$$

$$k_\mathrm{p}^\mathrm{app} = k_\mathrm{p}[\mathrm{C}]_\mathrm{t} \qquad (2)$$

$$-\frac{d[\mathrm{I}]}{dt} = k_\mathrm{i}^\mathrm{app}[\mathrm{I}]_\mathrm{t} \qquad (3)$$

$$k_\mathrm{i}^\mathrm{app} = k_\mathrm{i}[\mathrm{C}]_\mathrm{t} \qquad (4)$$

where k_p^{app} and k_i^{app} are the apparent propagation and initiation rate constants, respectively, $[M]_t$ and $[I]_t$ are the concentration of monomer and uninitiated catalyst at time t, k_p and k_i are the propagation and initiation rate constants, and $[C]_t$ is the catalyst concentration at time t.

^{1}H NMR Kinetic Studies for **P1**

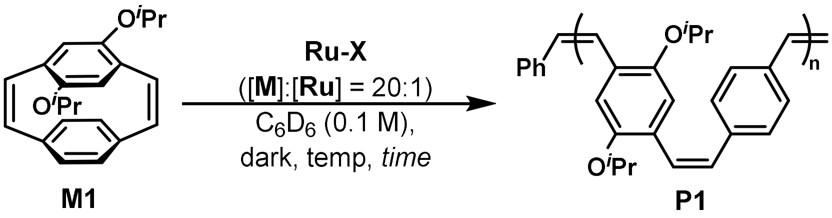

All-*cis* **P1**

^{1}H NMR (500 MHz, $CDCl_3$) δ 7.18 (s, 4 H), 6.75 (s, 2 H), 6.62 (d, *J* = 12.3 Hz, 2 H), 6.49 (d, *J* = 12.3 Hz, 2 H), 4.01 (hep., *J* = 6.1 Hz, 2 H), 1.10 (d, *J* = 6.0 Hz, 12 H) ppm.

^{13}C NMR (126 MHz, $CDCl_3$) δ 149.6, 136.3, 129.5, 128.9, 128.0, 126.1, 117.1, 72.0, 22.3 ppm.

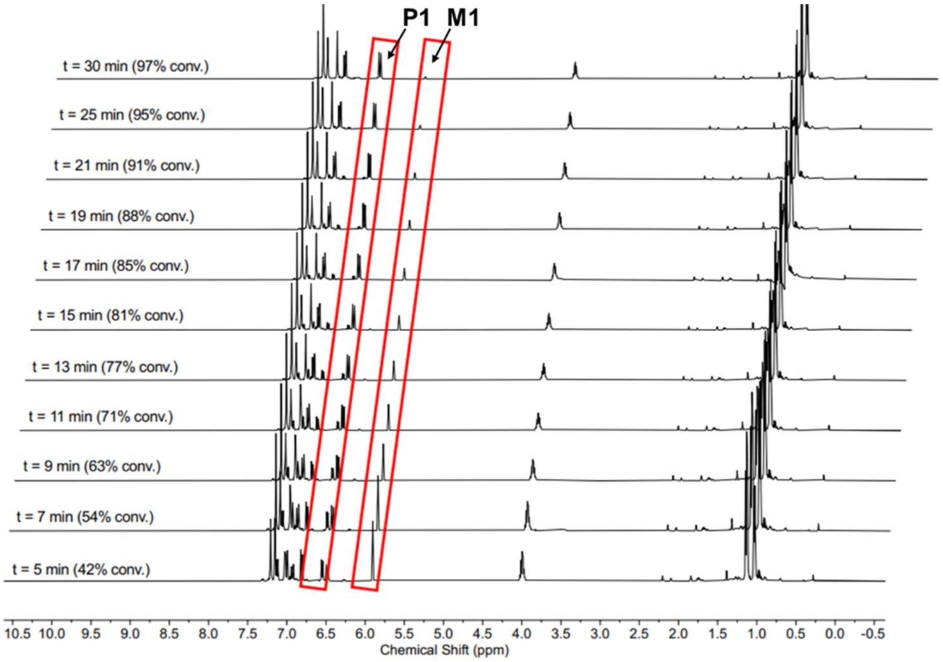

Figure S1. ^{1}H NMR spectra using general kinetics procedure A with **Ru-1a** and **M1** in C_6D_6. Conversion determined through comparing changes in integration of **P1** alkene signals (6.48 ppm, 2 H) and **M1** aromatic signal (5.91 ppm, 2 H) over time.

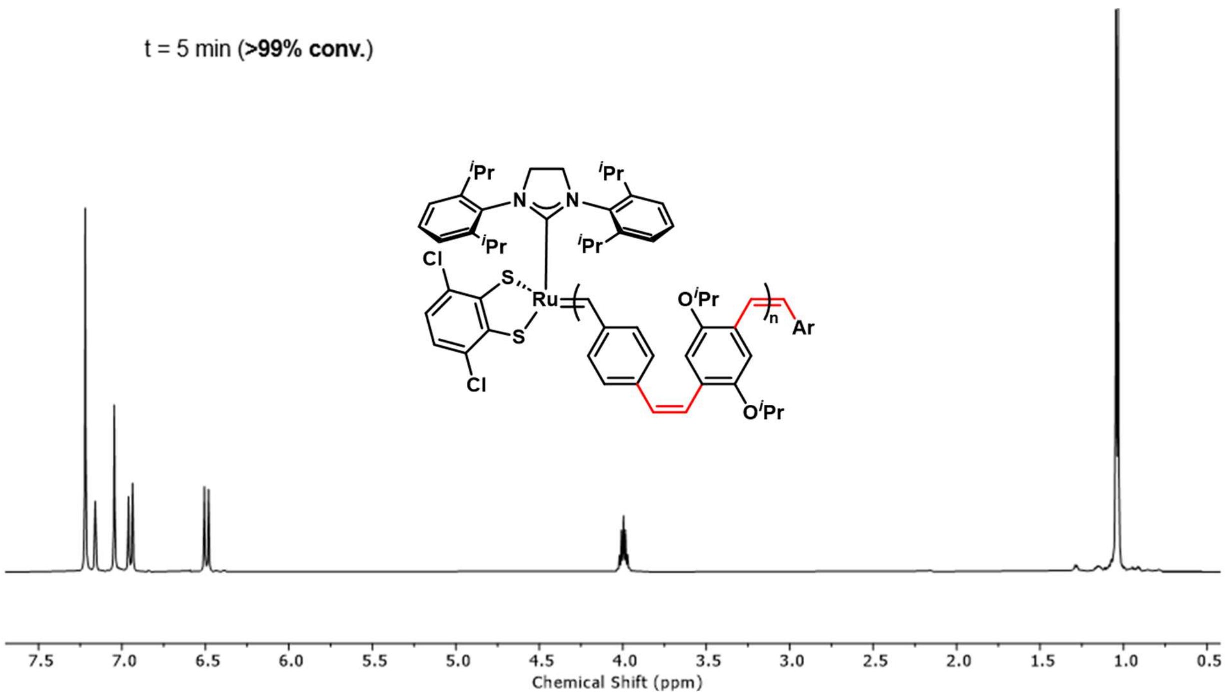

Figure S2. ^{1}H NMR spectrum using general kinetics procedure A with **Ru-2** and **M1** in C_6D_6. Full conversion observed at time of first ^{1}H NMR measurement (5 minutes).

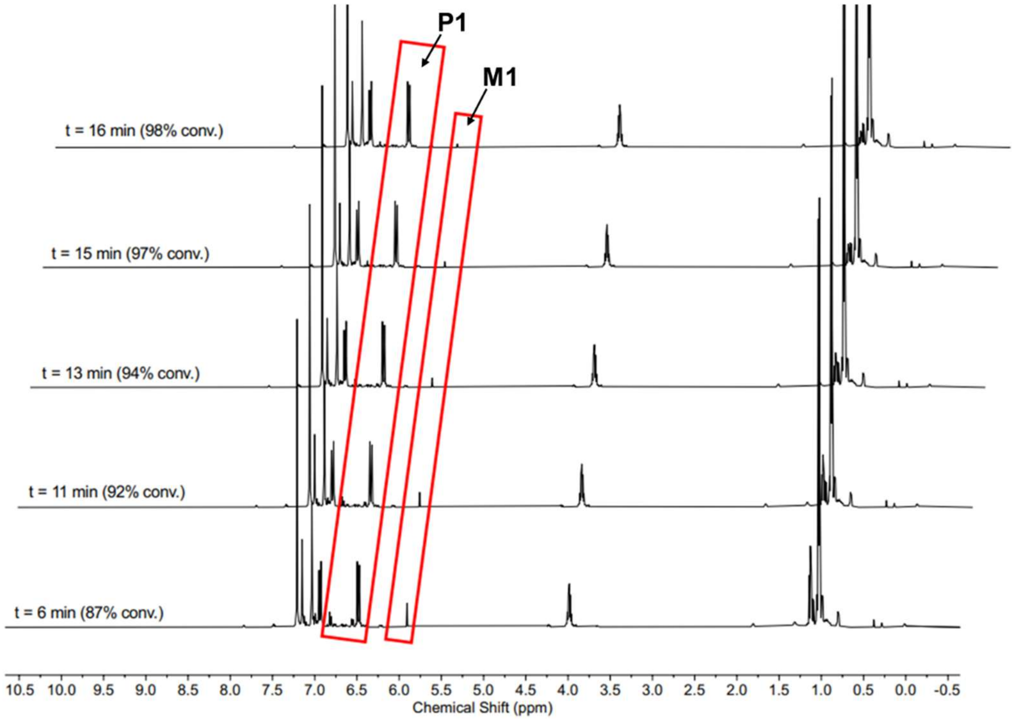

Figure S3. ^{1}H NMR spectra using general kinetics procedure B with **Ru-2 + 3-ClPy** (1:1.2) and **M1**. Conversion determined through comparing changes in integration of **P1** alkene signals (6.48 ppm, 2 H) and **M1** aromatic signal (5.91 ppm, 2 H) over time.

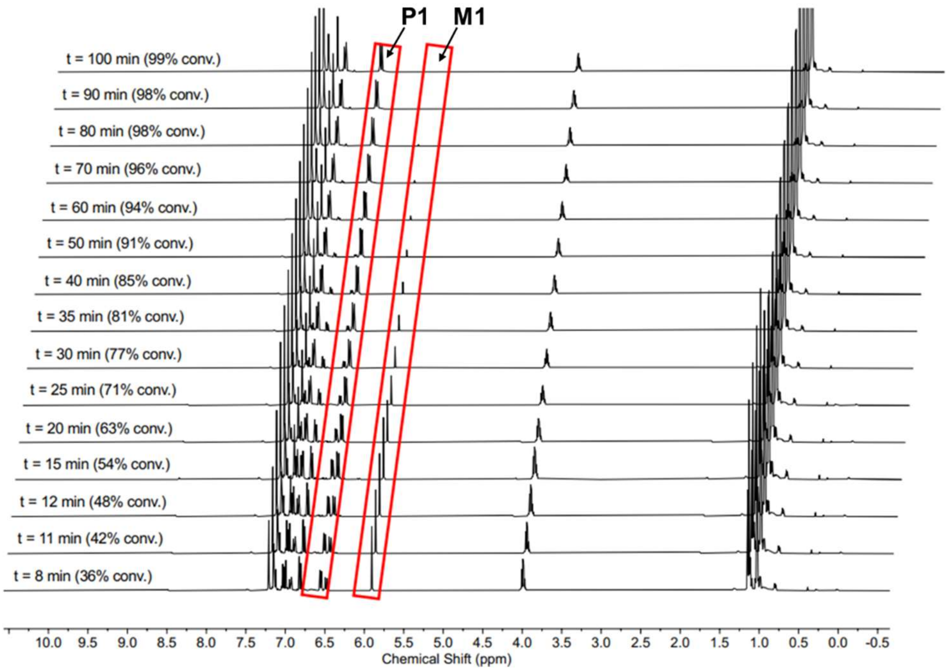

Figure S4. ^{1}H NMR spectra using general kinetics procedure B with **Ru-2 + 3-ClPy** (1:2.7) and **M1**. Conversion determined through comparing changes in integration of **P1** alkene signals (6.48 ppm, 2 H) and **M1** aromatic signal (5.91 ppm, 2 H) over time.

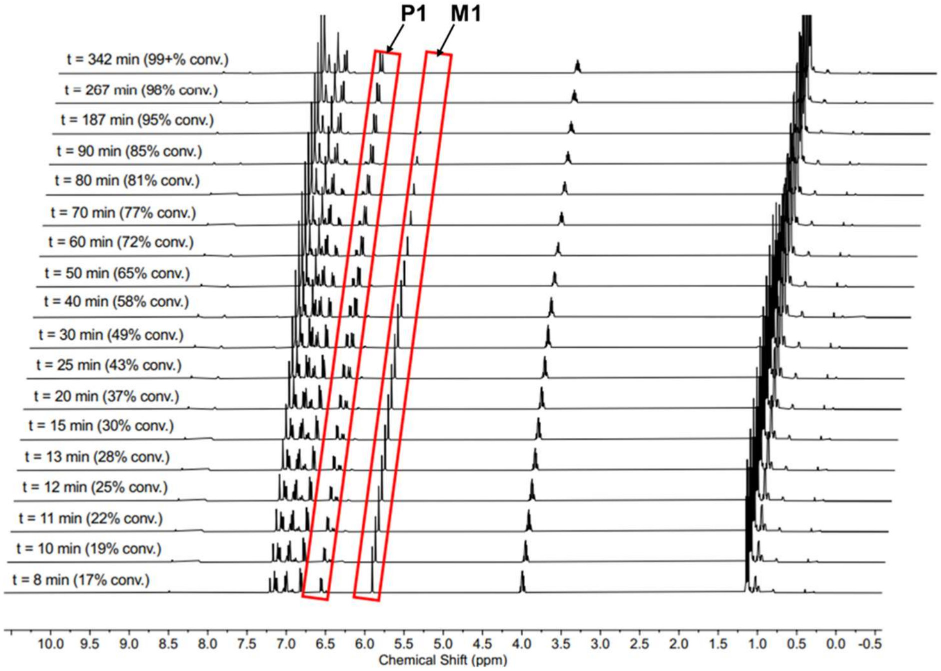

Figure S5. ^{1}H NMR spectra using general kinetics procedure B with **Ru-2 + 3-ClPy** (1:4.8) and **M1**. Conversion determined through comparing changes in integration of **P1** alkene signals (6.48 ppm, 2 H) and **M1** aromatic signal (5.91 ppm, 2 H) over time.

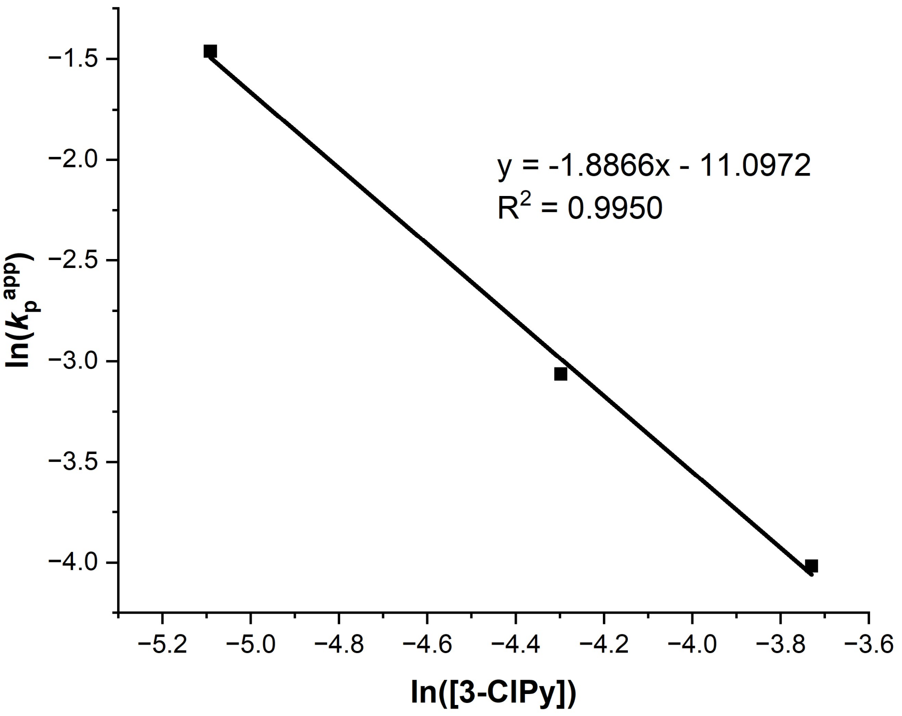

Figure S6. ln([**3-ClPy**]) vs. ln(k_p^{app}) at constant **Ru-2** loading when polymerizing **M1**.

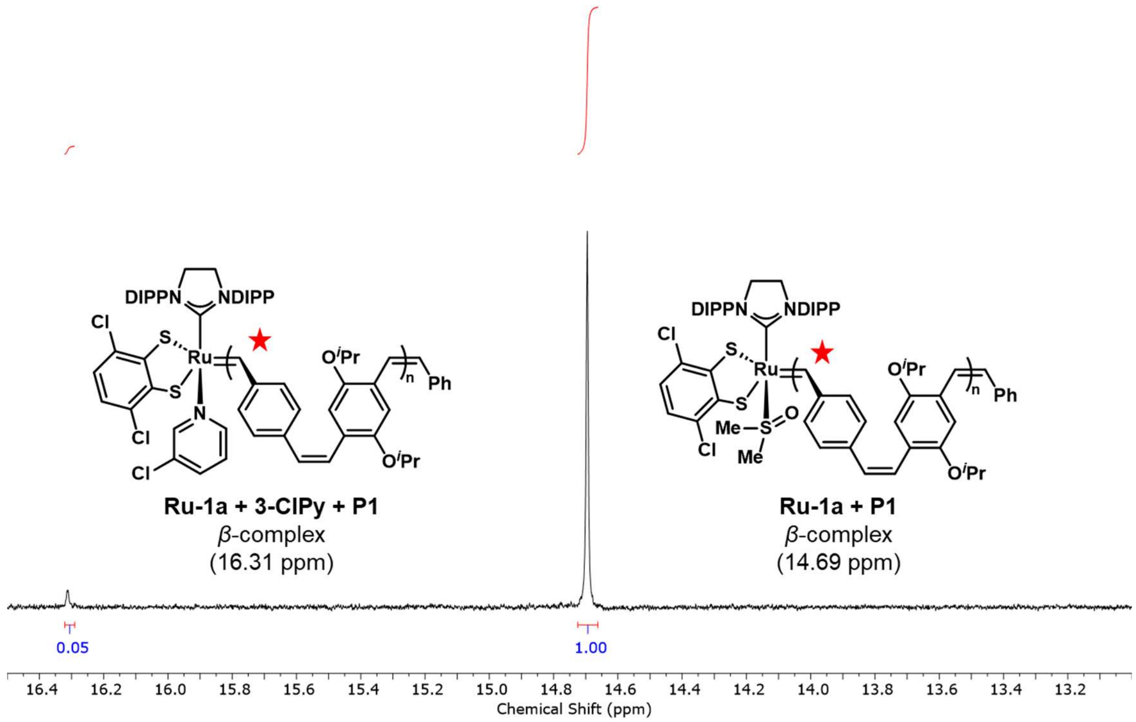

Figure S7. ^{1}H NMR spectrum of alkylidene region at 99% conversion of **M1** (20 equiv) using 1 equiv of **Ru-1a** and **3-ClPy** at rt in C_6D_6.

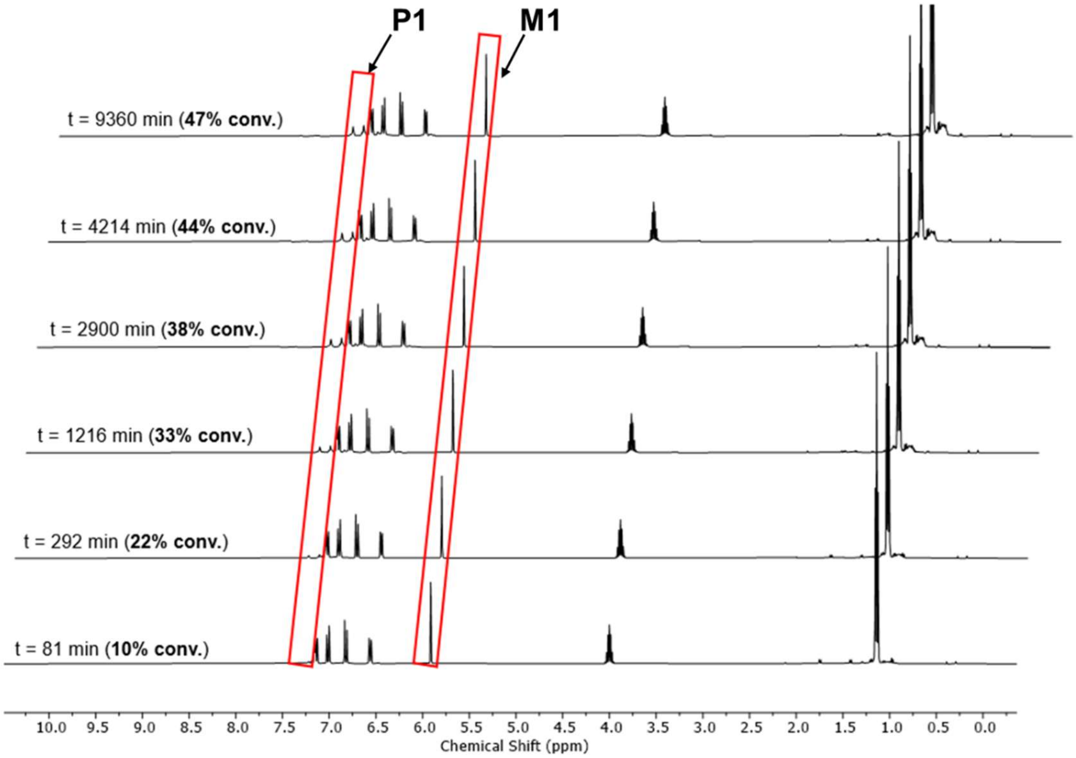

Figure S8. ^{1}H NMR spectra using general kinetics procedure A with **Ru-3a** and **M1** in C_6D_6. Conversion determined through comparing changes in integration of **P1** alkene signals (7.34–7.23 ppm, 4 H) and **M1** aromatic signal (5.91 ppm, 2 H) over time.

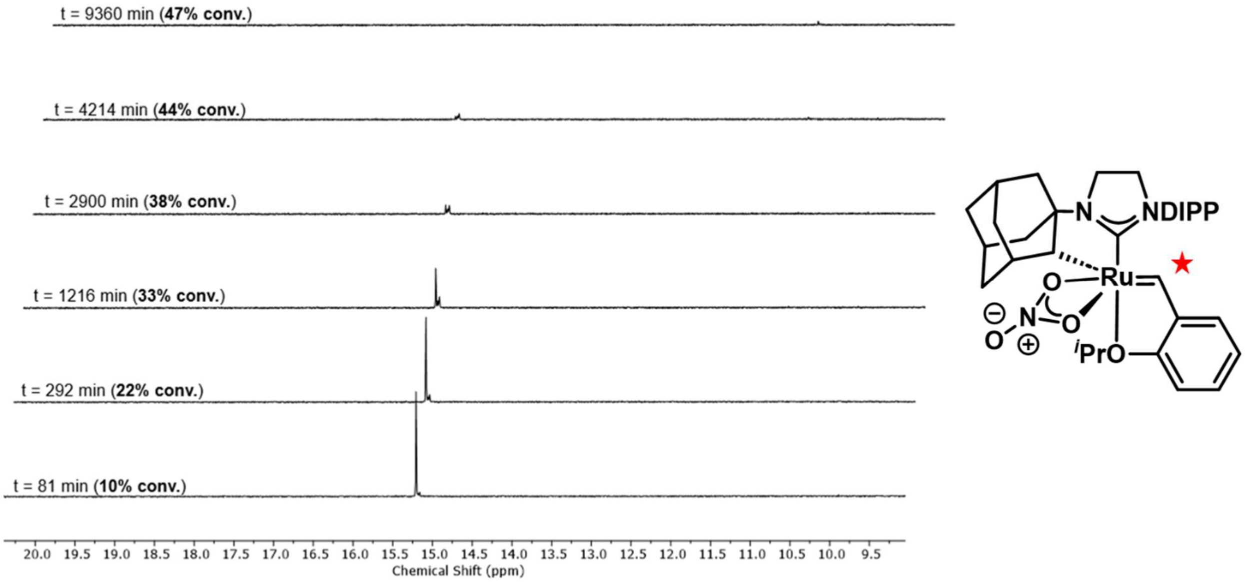

Figure S9. ^{1}H NMR spectra showing alkylidene decomposition with **Ru-3a** and **M1** in C_6D_6 at 70 °C.

^{1}H NMR Kinetic Studies for **P2**

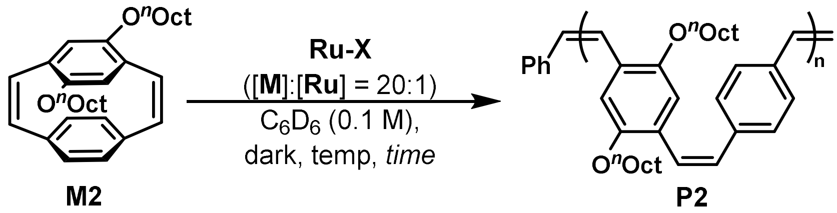

Spectroscopic data for all-*cis* **P2** was identical to those reported in the literature.[4]

^{1}H NMR (400 MHz, $CDCl_3$) δ 7.20 (s, 4 H), 6.76 (s, 2 H), 6.64 (d, *J* = 12.3 Hz, 2 H), 6.50 (d, *J* = 12.3 Hz, 2 H), 3.53 (t, *J* = 6.5 Hz, 4 H), 1.33–1.20 (m, 24 H), 0.87 (t, *J* = 6.6 Hz, 6 H) ppm.

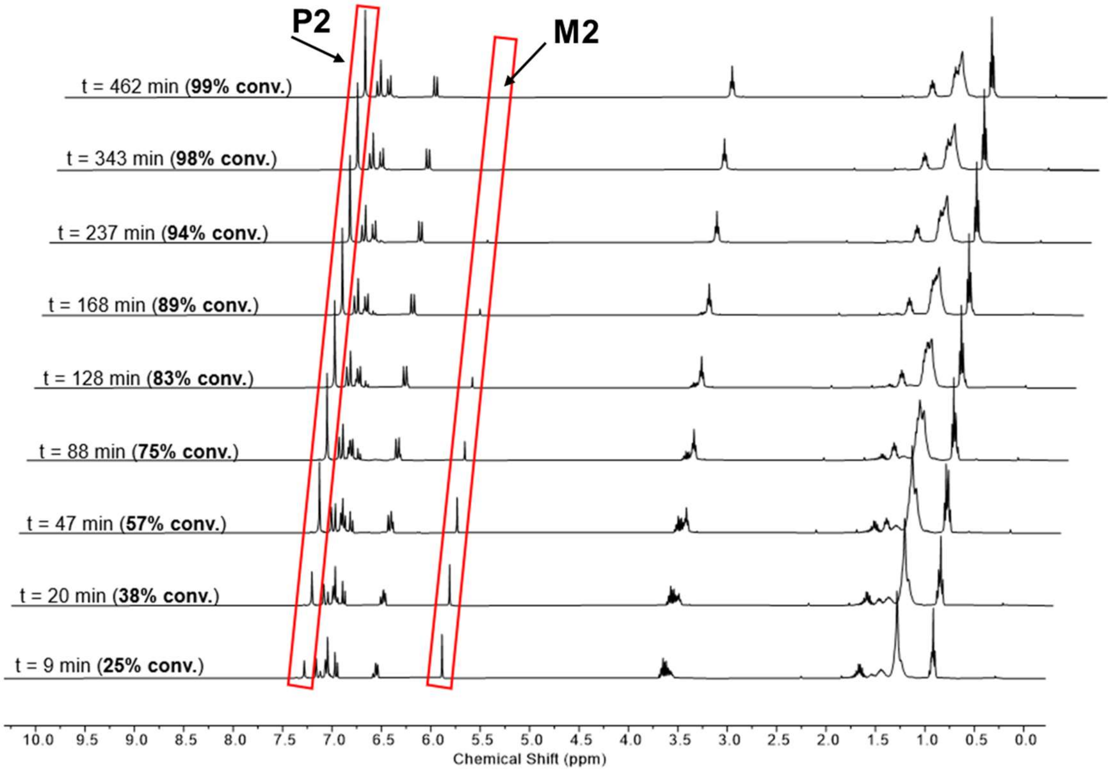

Figure S10. ^{1}H NMR spectra using general kinetics procedure A with **Ru-1a** and **M2** in C_6D_6. Conversion determined through comparing changes in integration of a *cis*-**P2** aromatic signal (7.29 ppm, 4 H) and **M2** aromatic signal (5.88 ppm, 2 H) over time.

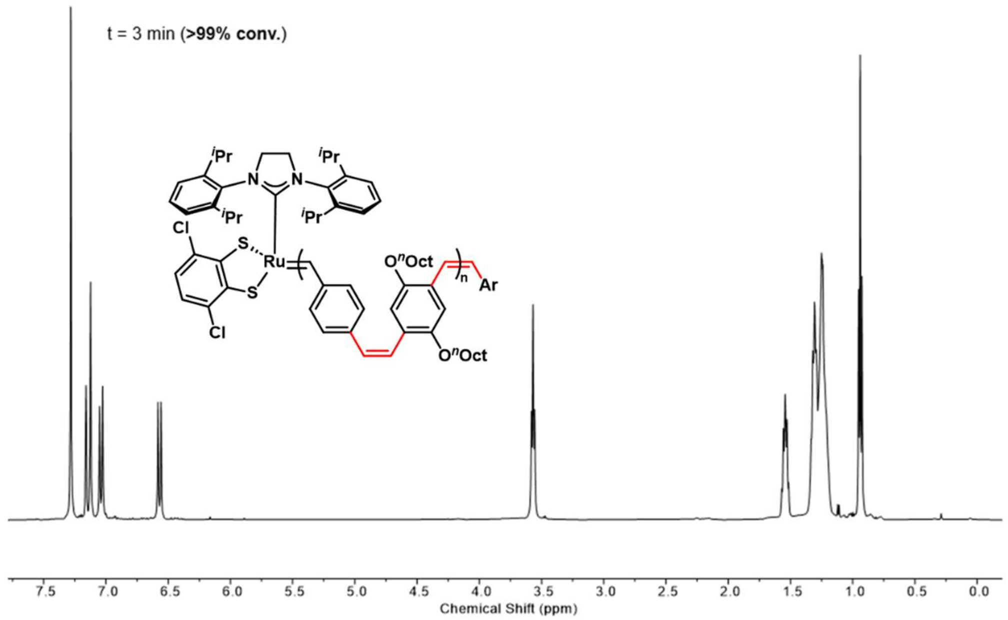

Figure S11. ^{1}H NMR spectrum using general kinetics procedure A with **Ru-2** and **M2** in C_6D_6. Full conversion observed at time of first ^{1}H NMR measurement (3 minutes).

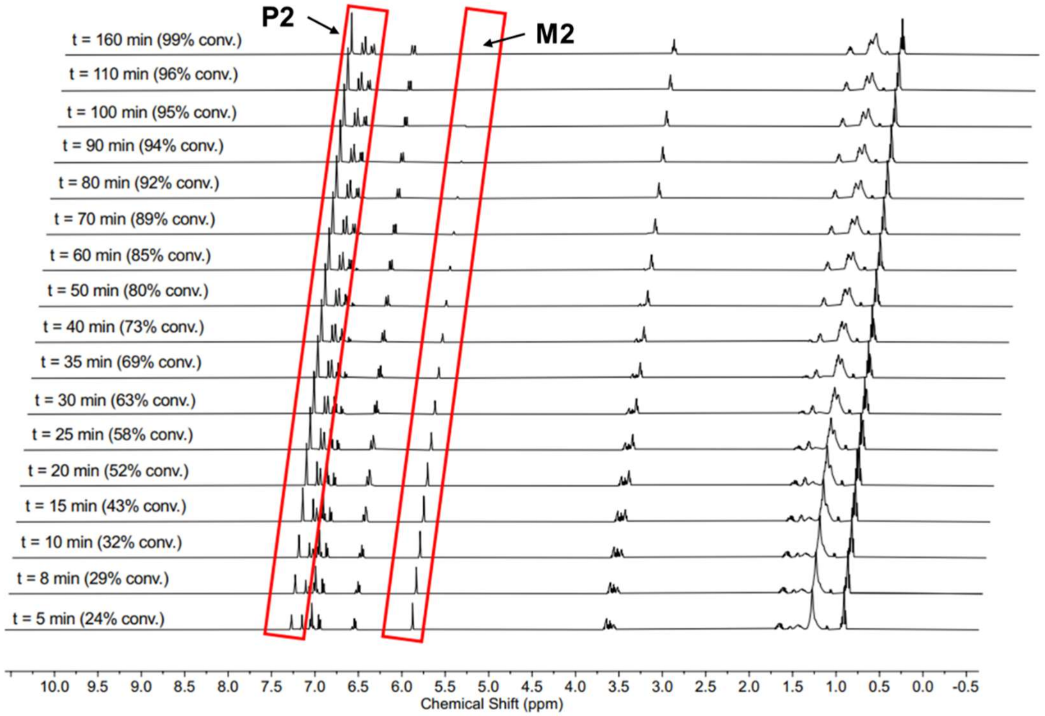

Figure S12. ^{1}H NMR spectra using general kinetics procedure B with **Ru-2 + 3-ClPy** (1:2) and **M2** in C_6D_6. Conversion determined through comparing changes in integration of **P2** aromatic signals (7.40 ppm, 4 H and 7.29 ppm, 4 H) and **M2** aromatic signal (5.88 ppm, 2 H) over time.

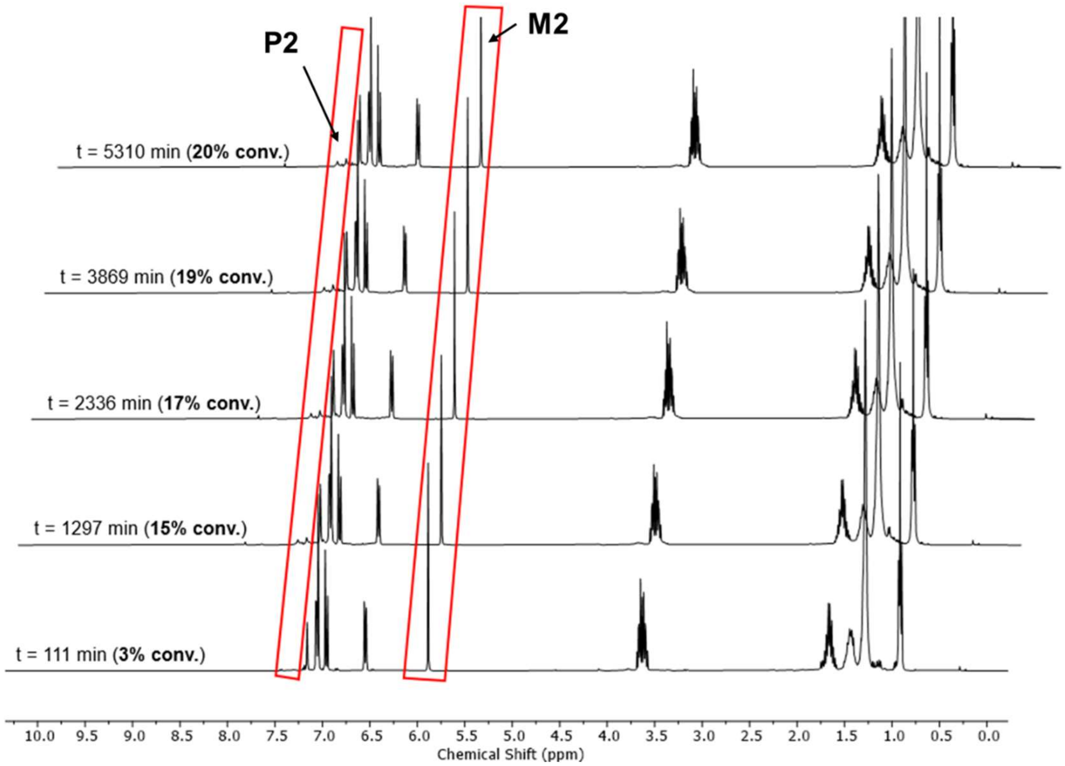

Figure S13. ^{1}H NMR spectra using general kinetics procedure A with **Ru-3a** and **M2** in C_6D_6. Conversion determined through comparing changes in integration of **P2** aromatic signals (7.40–7.29 ppm, 4 H) and **M2** aromatic signal (5.88 ppm, 2 H) over time.

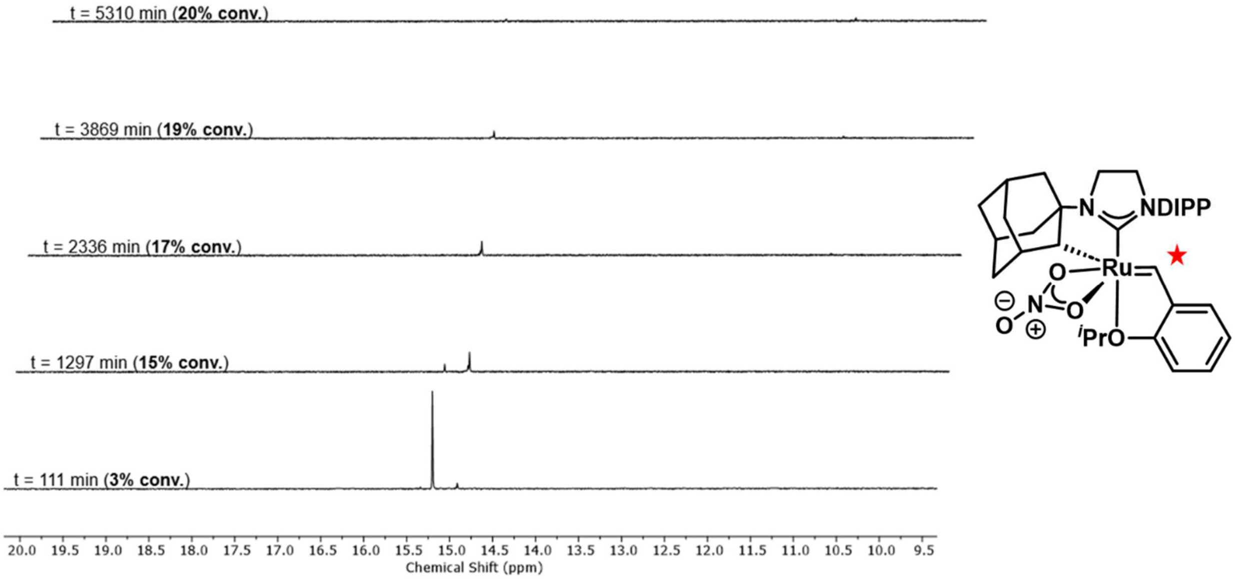

Figure S14. ^{1}H NMR spectra showing alkylidene decomposition with **Ru-3a** and **M2** in C_6D_6 at 70 °C.

^{1}H NMR Kinetic Studies for **P3**

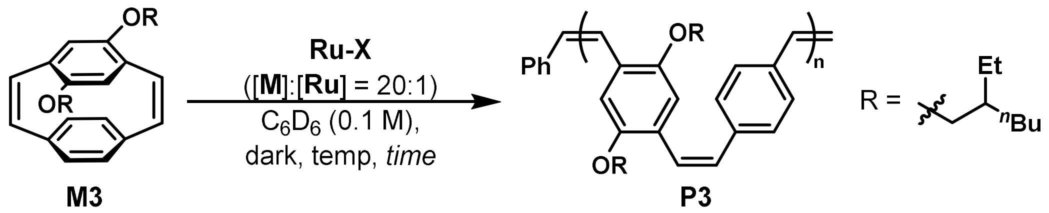

Spectroscopic data for all-*cis* **P3** was identical to those reported in the literature.[5]

^{1}H NMR (400 MHz, $CDCl_3$) δ 7.21 (s, 4 H), 6.75 (s, 2 H), 6.65 (d, *J* = 12.3 Hz, 2 H), 6.49 (d, *J* = 12.3 Hz, 2 H), 3.40 (d, *J* = 5.7 Hz, 4 H), 1.58–1.13 (m, 18 H), 0.86 (t, *J* = 7.2 Hz, 6 H), 0.80 (t, *J*= 7.2 Hz, 6 H) ppm.

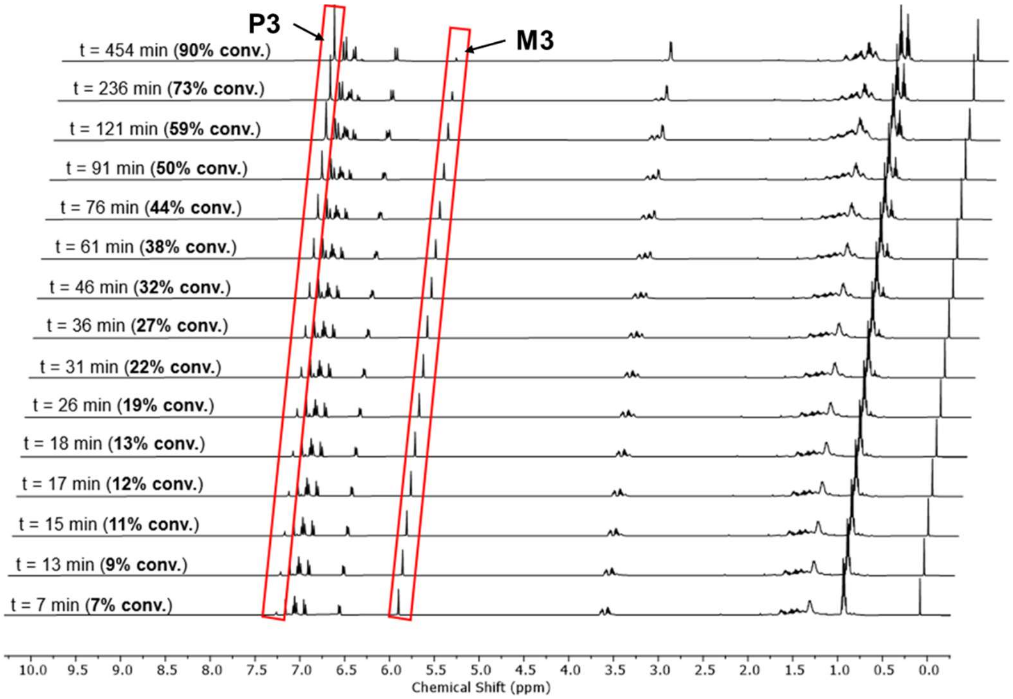

Figure S15. Overlayed ^{1}H NMR spectra using general kinetics procedure A with **Ru-1a** and **M3** in C_6D_6. Conversion determined through comparing changes in integration of a *cis*-**P3** aromatic signal (7.26 ppm, 4 H) and **M3** aromatic signal (5.90 ppm, 2 H) over time.

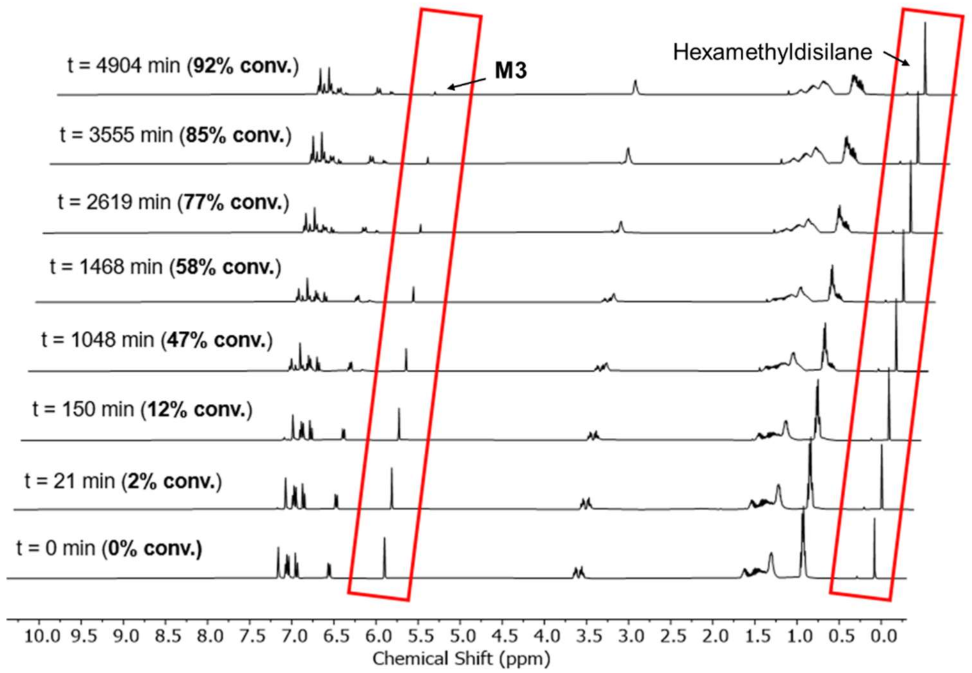

Figure S16. ^{1}H NMR spectra using general kinetics procedure A with **Ru-1b** and **M3** in C_6D_6. Conversion determined by comparing difference in integration of a **M3** peak (5.90 ppm) relative to a hexamethyldisilane internal standard (0.08 ppm) over time.

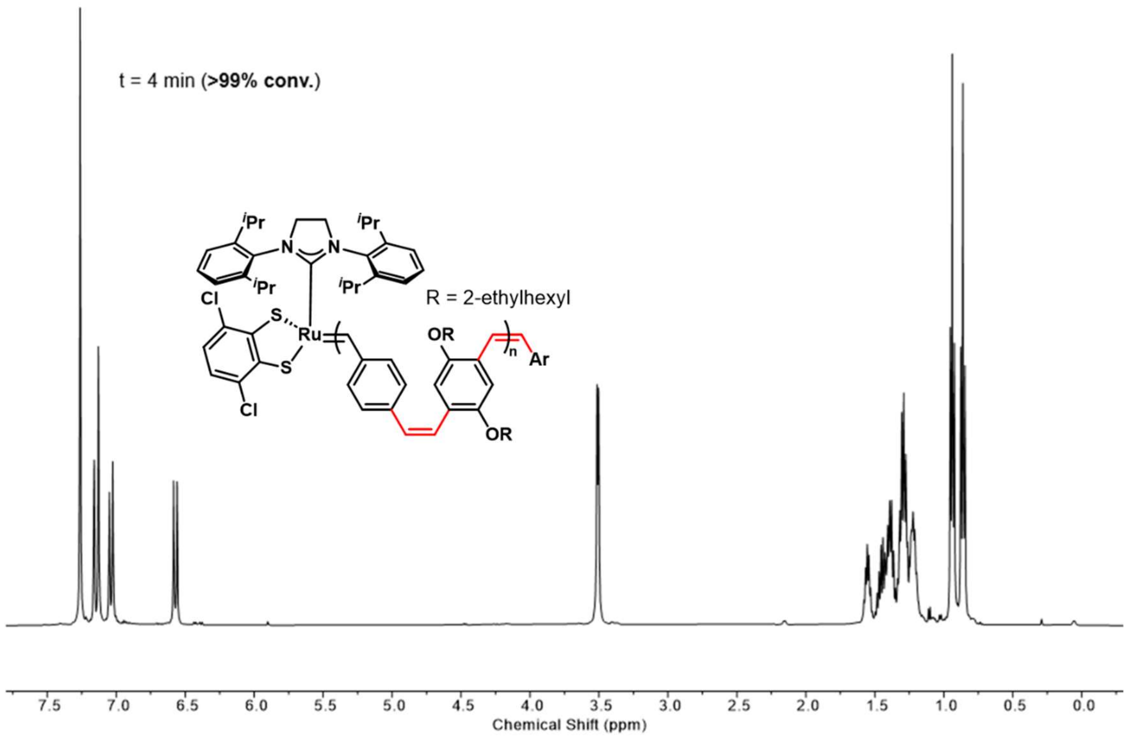

Figure S17. ^{1}H NMR spectrum using general kinetics procedure A with **Ru-2** and **M3** in C_6D_6. Full conversion observed at time of first ^{1}H NMR measurement (4 minutes).

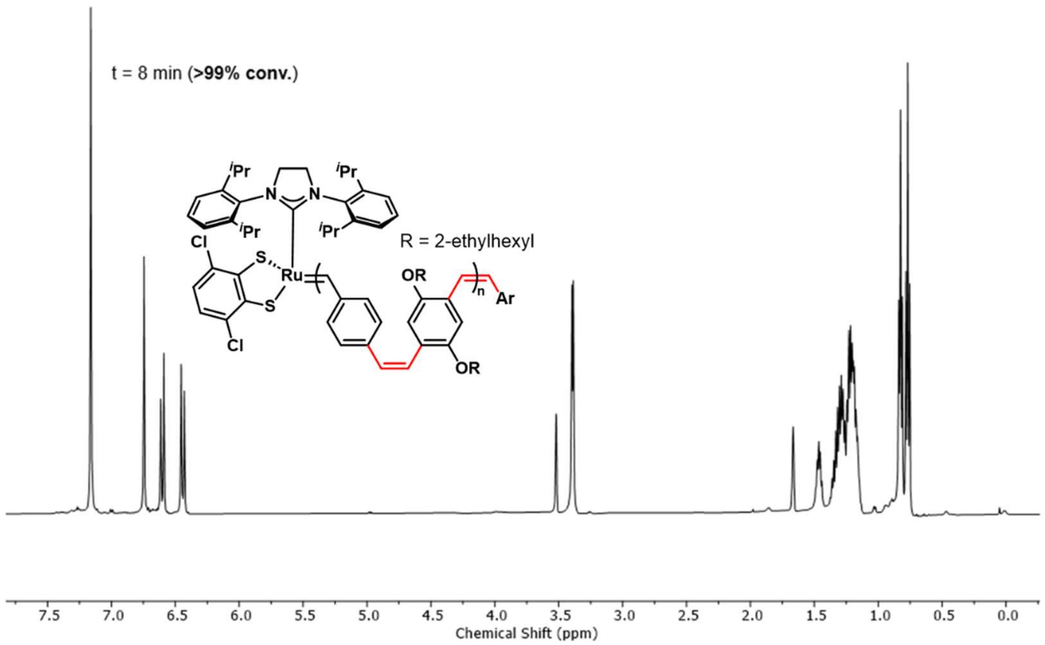

Figure S18. ^{1}H NMR spectrum using general kinetics procedure A with **Ru-2** and **M3** in THF-d_8. Full conversion observed at time of first ^{1}H NMR measurement (8 minutes).

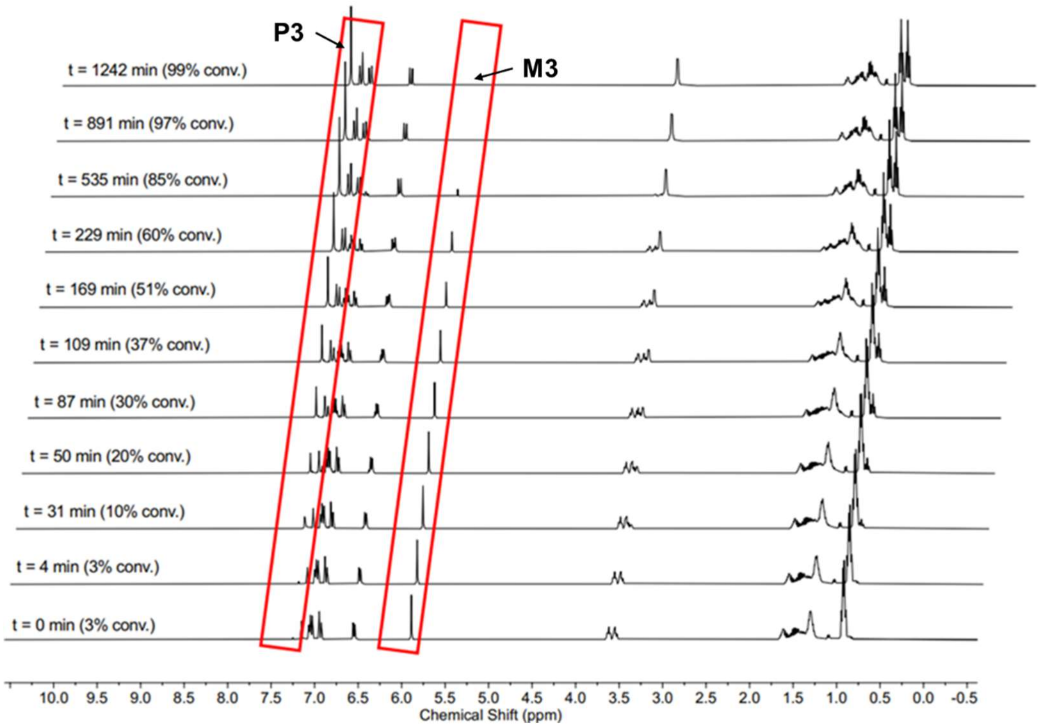

Figure S19. ^{1}H NMR spectra using general kinetics procedure B with **Ru-2 + 3-ClPy** (1:2) and **M3** in C_6D_6. Conversion determined through comparing changes in integration of a *cis*-**P3** aromatic signal (7.26 ppm, 4 H) and **M3** aromatic signal (5.90 ppm, 2 H) over time.

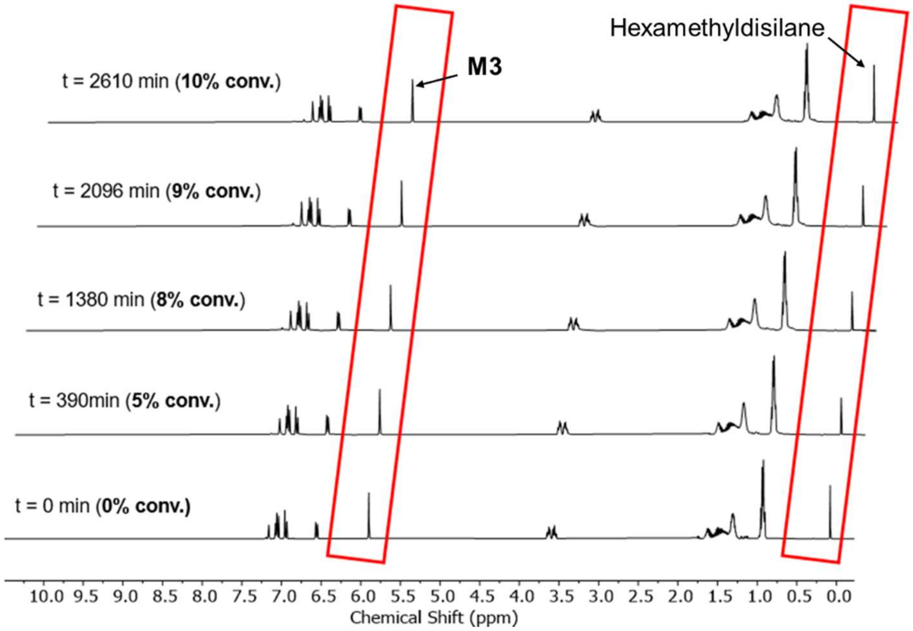

Figure S20. ^{1}H NMR spectra using general kinetics procedure A with **Ru-3a** and **M3** in C_6D_6. Conversion determined by comparing difference in integration of a **M3** peak (5.90 ppm) relative to a hexamethyldisilane internal standard (0.08 ppm) over time.

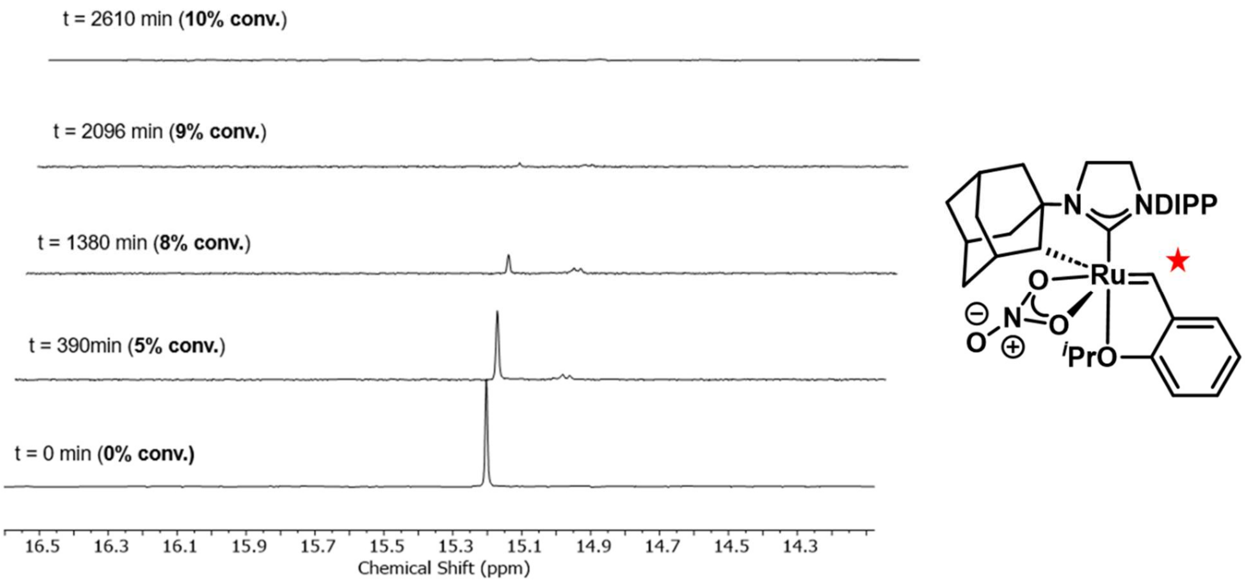

Figure S21. ^{1}H NMR spectra showing alkylidene decomposition with **Ru-3a** and **M3** in C_6D_6 at 70 °C.

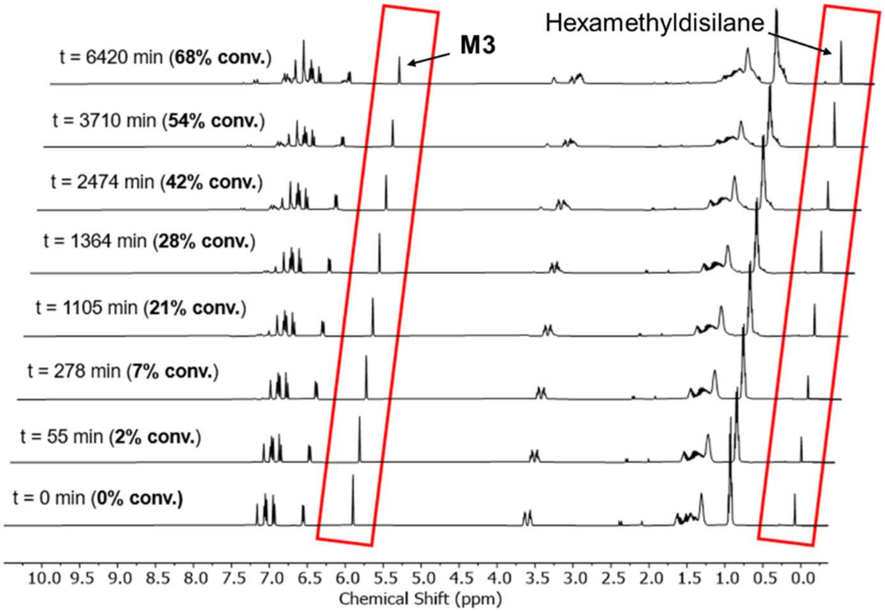

Figure S22. Overlayed ^{1}H NMR spectra using general kinetics procedure A with **Ru-3b** and **M3** in C_6D_6. Conversion determined by comparing difference in integration of a **M3** peak (5.90 ppm) relative to a hexamethyldisilane internal standard (0.08 ppm) over time.

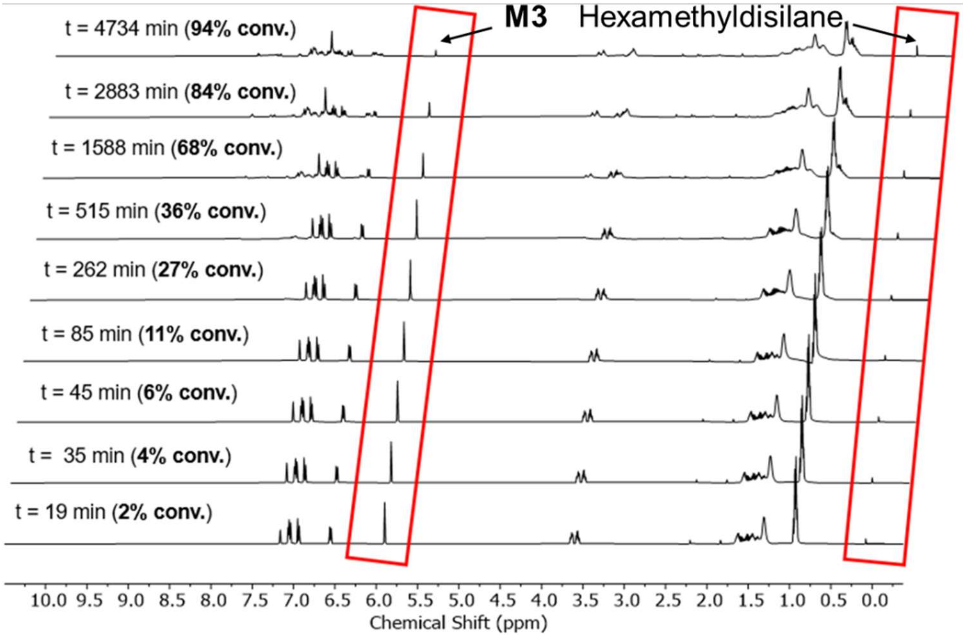

Figure S23. Overlayed ^{1}H NMR spectra using general kinetics procedure A with **GII** and **M3** in C_6D_6. Conversion determined by comparing difference in integration of a **M3** peak (5.90 ppm) relative to a hexamethyldisilane internal standard (0.08 ppm) over time.

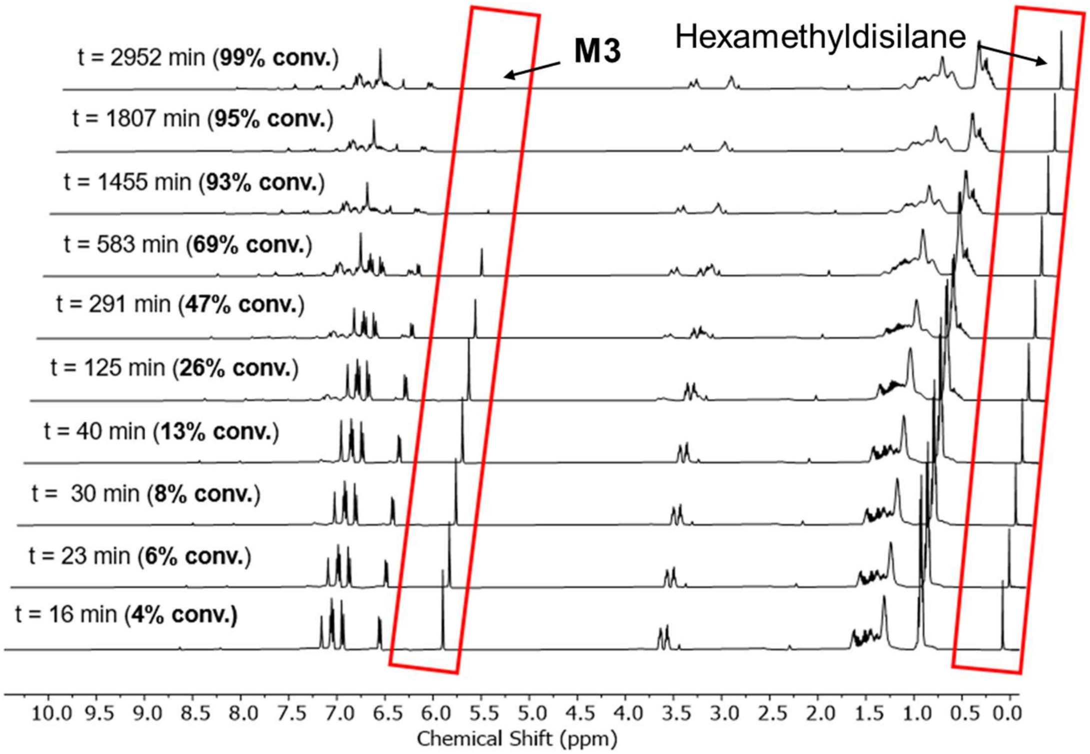

Figure S24. Overlayed ^{1}H NMR spectra using general kinetics procedure A with **GIII** and **M3** in C_6D_6. Conversion determined by comparing difference in integration of a **M3** peak (5.90 ppm) relative to a hexamethyldisilane internal standard (0.08 ppm) over time.

^{1}H NMR Kinetic Studies for **P4**

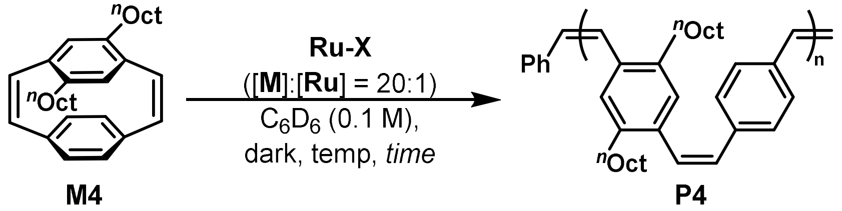

Spectroscopic data for all-*cis* **P4** was identical to those reported in the literature.[5]

^{1}H NMR (500 MHz, $CDCl_3$) δ 7.00–6.93 (m, 6 H), 6.63 (d, *J* = 12.2 Hz, 2 H), 6.48 (d, *J* = 12.2 Hz, 2 H), 2.48–2.38 (m, 4 H), 1.44–1.34 (m, 4 H), 1.32–1.11 (m, 20 H), 0.85 (t, *J* = 6.9 Hz, 6 H) ppm.

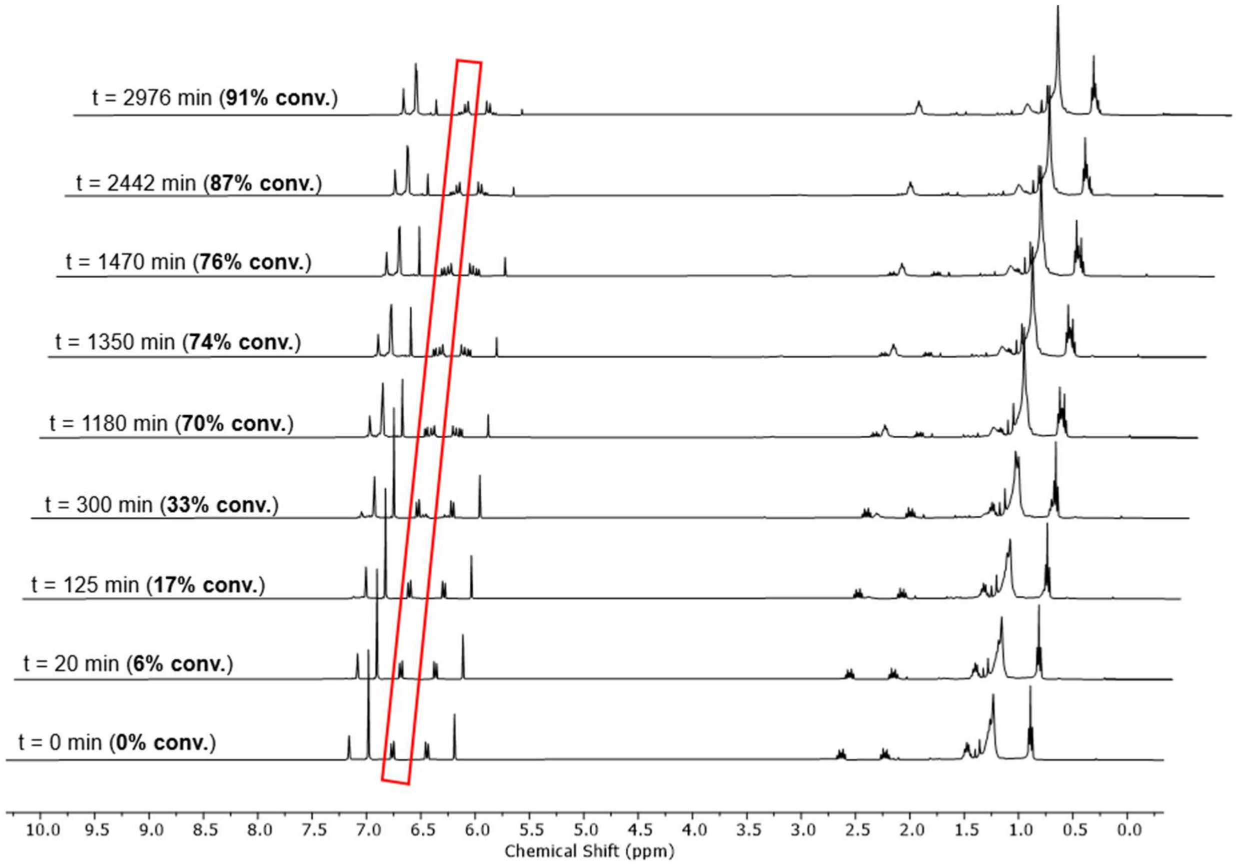

Figure S25. ^{1}H NMR spectra using general kinetics procedure A with **Ru-1a** and **M4** in C_6D_6. Conversion determined through comparing changes in integration of a *cis*-**P4** alkene signal (6.76 ppm, 2 H) and **M4** alkene signal (6.70 ppm, 2 H) over time.

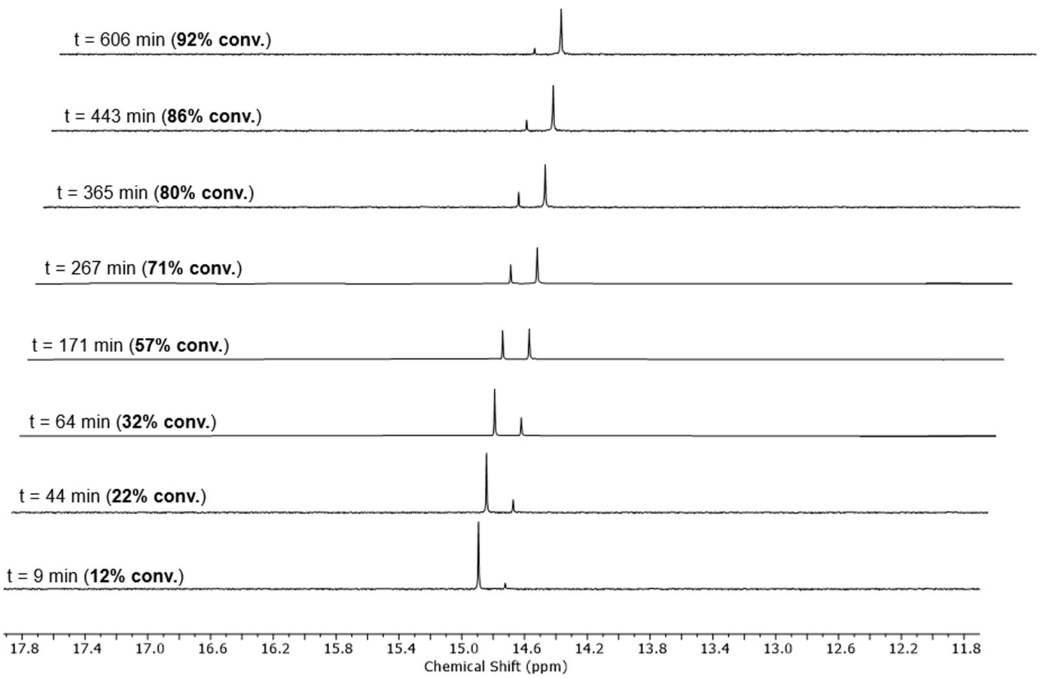

Figure S26. ^{1}H NMR spectra showing initiation in alkylidene region over time with **Ru-1a** and **M4** at rt in C_6D_6. Conversion determined by comparing integration of uninitiated alkylidene signal (14.90 ppm, 1 H) and propagating alkylidene signal (14.73 ppm, 1 H) over time.

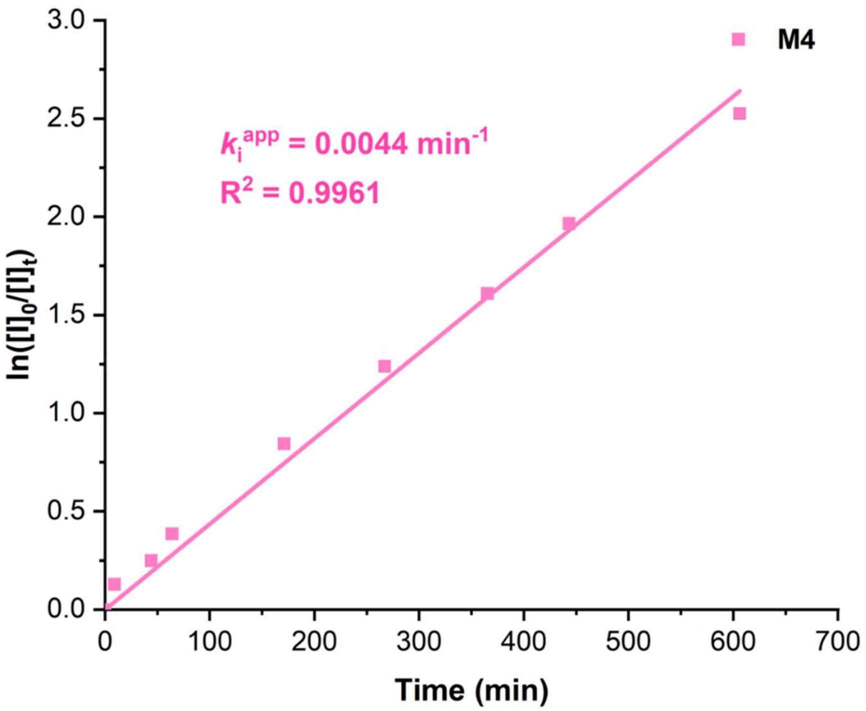

Figure S27. Initiation kinetics with **Ru-1a** (2.2 mg, 0.0025 mmol) and **M4** (21.4 mg, 0.05 mmol) at rt in C_6D_6 (0.5 mL).

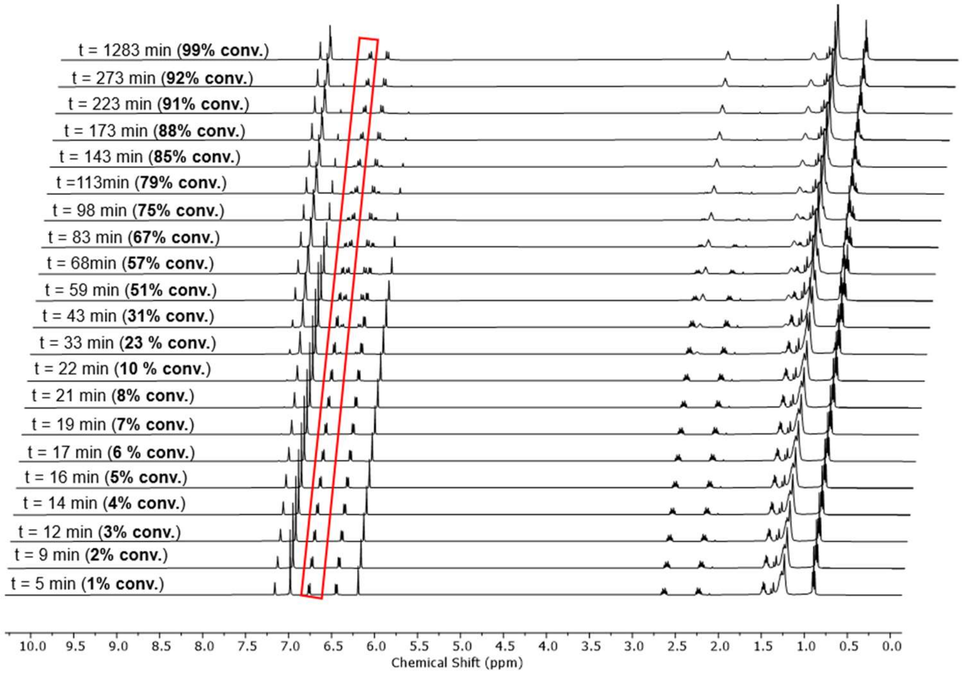

Figure S28. ^{1}H NMR spectra using general kinetics procedure A with **Ru-2** and **M4** in C_6D_6. Conversion determined through comparing changes in integration of a *cis*-**P4** alkene signal (6.76 ppm, 2 H) and **M4** alkene signal (6.70 ppm, 2 H) over time.

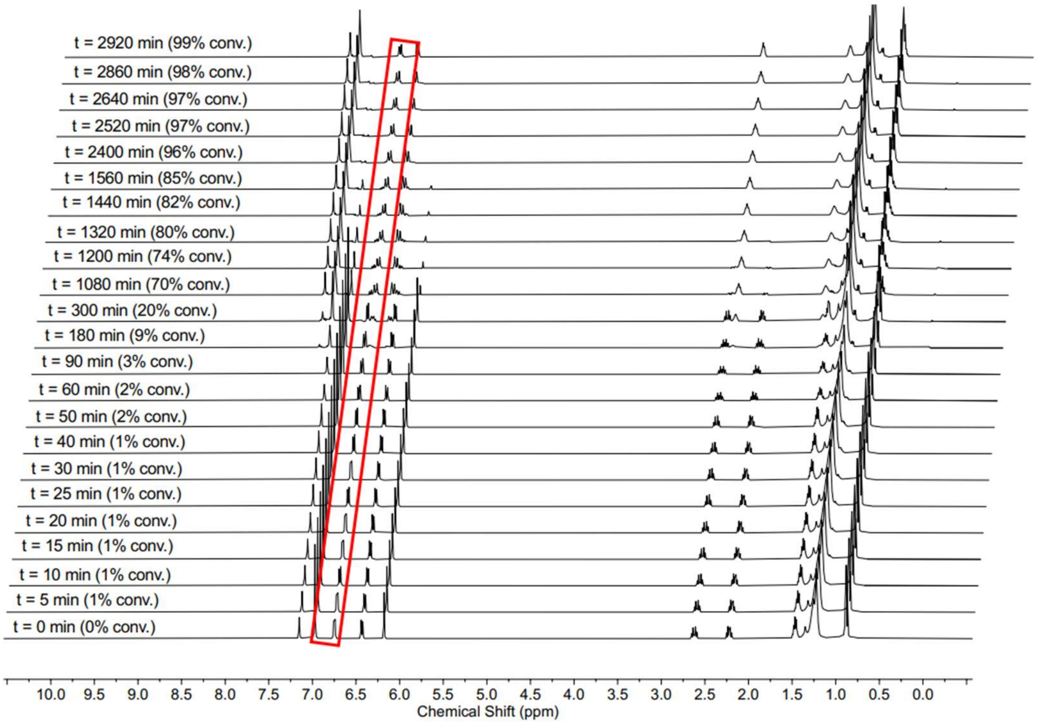

Figure S29. Overlayed ^{1}H NMR spectra using general kinetics procedure B with **Ru-2 + 3-ClPy** (1:2) and **M4** in C_6D_6. Conversion determined through comparing changes in integration of a *cis*-**P4** alkene signal (6.76 ppm, 2 H) and **M4** alkene signal (6.70 ppm, 2 H) over time.

^{1}H NMR Kinetic Studies for **P5**

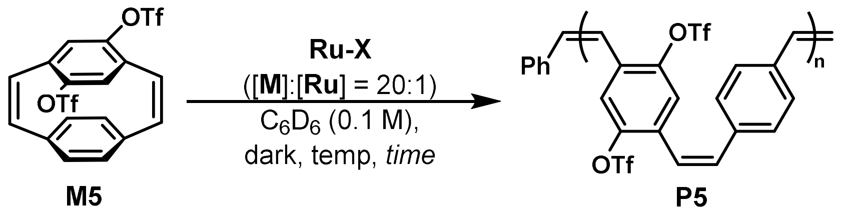

All-*cis* **P5**

^{1}H NMR (500 MHz, $CDCl_3$) δ 7.14–6.90 (m, 6 H), 6.81–6.64 (m, 2 H), 6.47–6.32 (m, 2 H) ppm.

^{13}C NMR (126 MHz, $CDCl_3$) δ 146.6, 145.9, 145.8, 135.5, 135.2, 132.5, 129.2, 128.8, 124.4, 122.3, 121.2, 119.8, 117.2, 114.7 ppm.

^{19}F NMR (470 MHz, $CDCl_3$) δ –73.42, –73.51, – (73.67–73.85) (m) ppm.

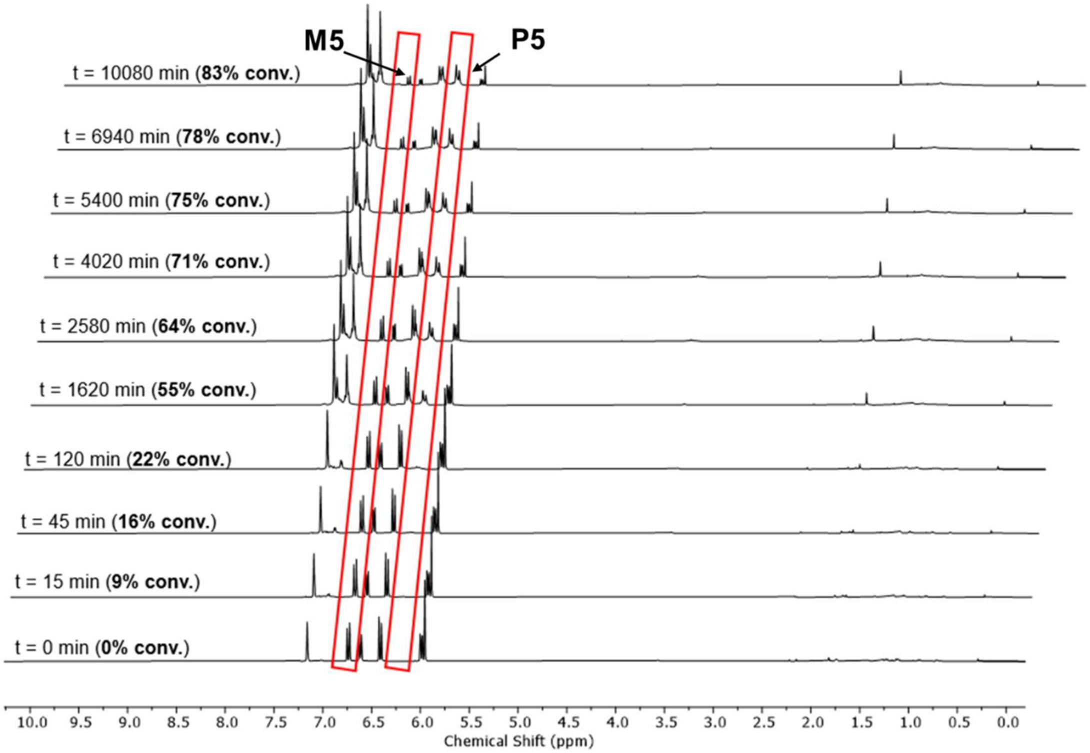

Figure S30. ^{1}H NMR spectra using general kinetics procedure A with **Ru-1a** and **M5** in C_6D_6. Conversion determined through comparing changes in integration of a **M5** alkene signal (6.74 ppm, 2 H) and *cis*-**P5** alkene signal (6.24 ppm, 2 H) over time.

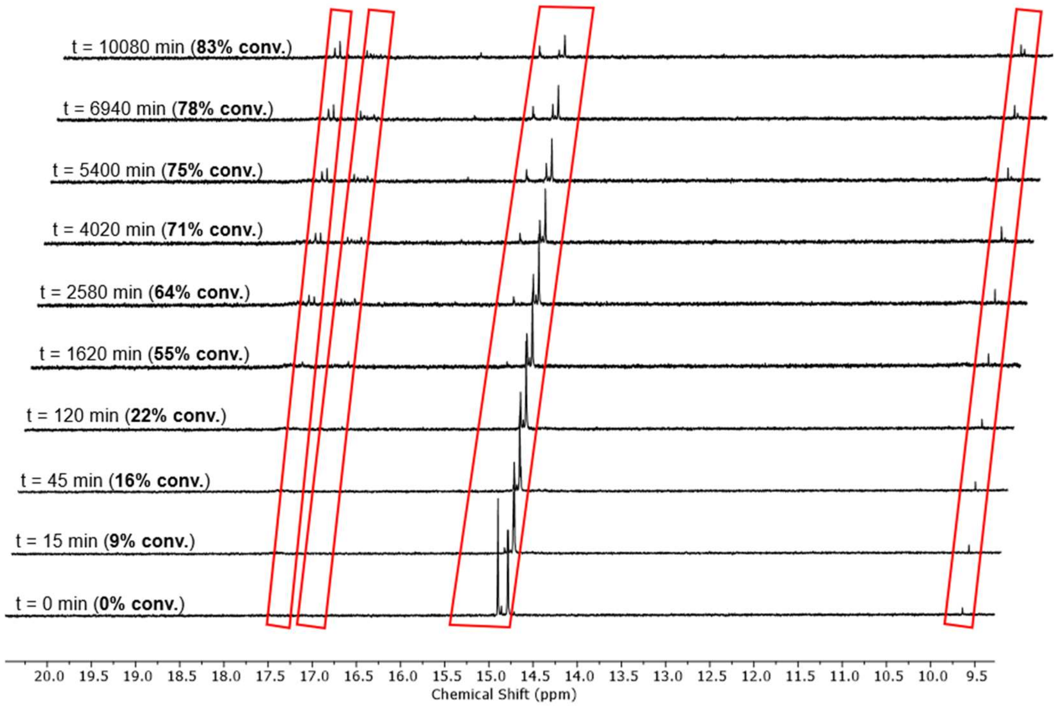

Figure S31. ^{1}H NMR spectra showing alkylidene region over time with **Ru-1a** and **M5** in C_6D_6.

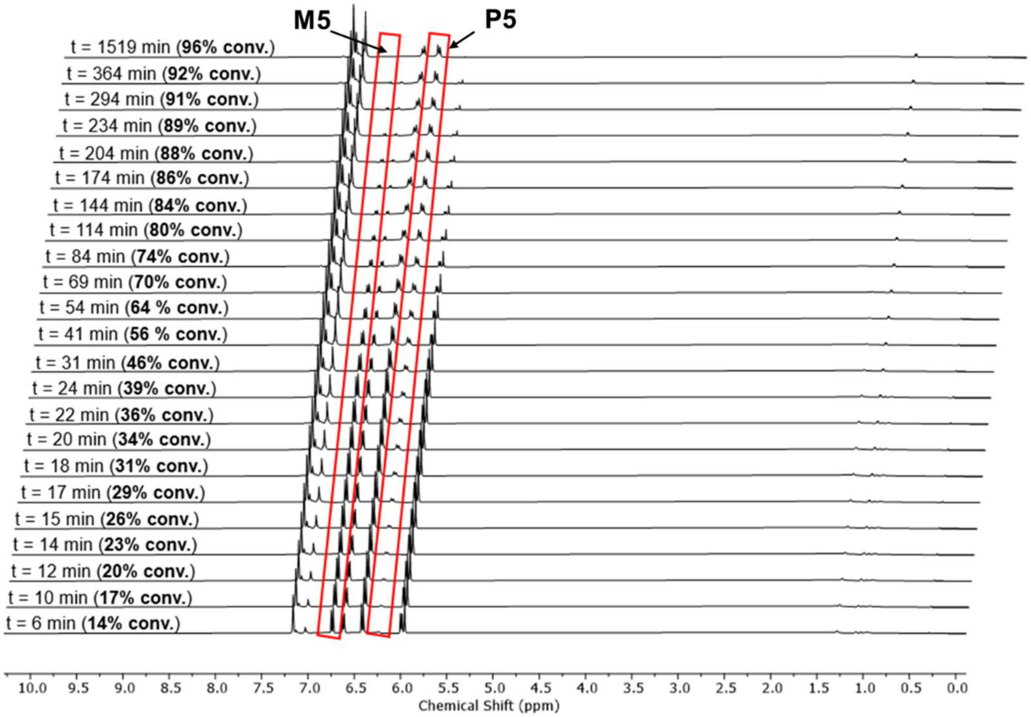

Figure S32. ^{1}H NMR spectra using general kinetics procedure A with **Ru-2** and **M5** in C_6D_6. Conversion determined through comparing changes in integration of a **M5** alkene signal (6.74 ppm, 2 H) and *cis*-**P5** alkene signal (6.24 ppm, 2 H) over time.

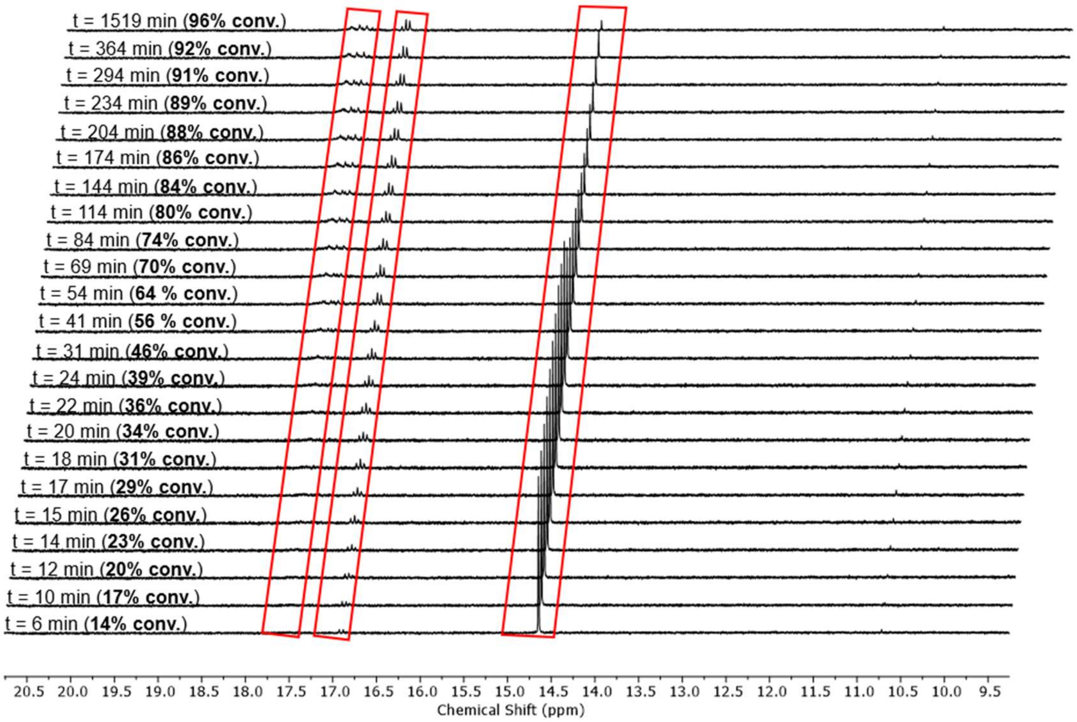

Figure S33. ^{1}H NMR spectra showing alkylidene region over time with **Ru-2** and **M5** in C_6D_6.

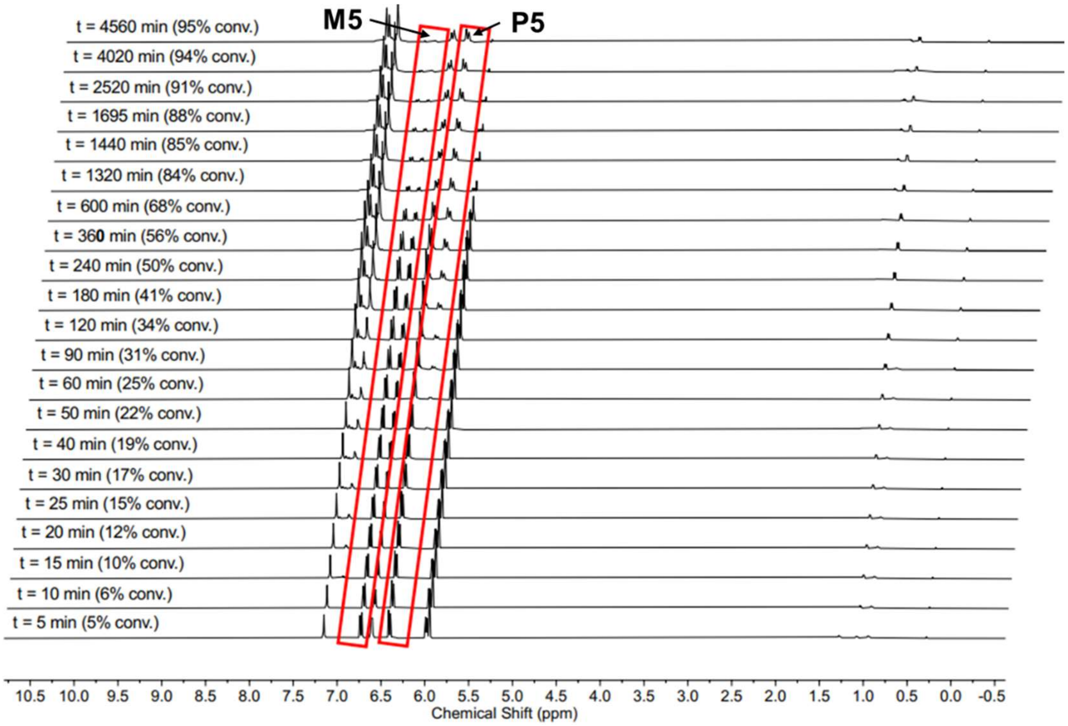

Figure S34. ^{1}H NMR spectra using general kinetics procedure B with **Ru-2 + 3-ClPy** (1:2) and **M5** in C_6D_6. Conversion determined through comparing changes in integration of **M5** alkene signal (6.74 ppm, 2 H) and *cis*-**P5** alkene signal (6.24 ppm, 2 H) over time.

Summary of the Reaction Kinetics Performed using *in-situ* ^{1}H NMR Spectroscopy

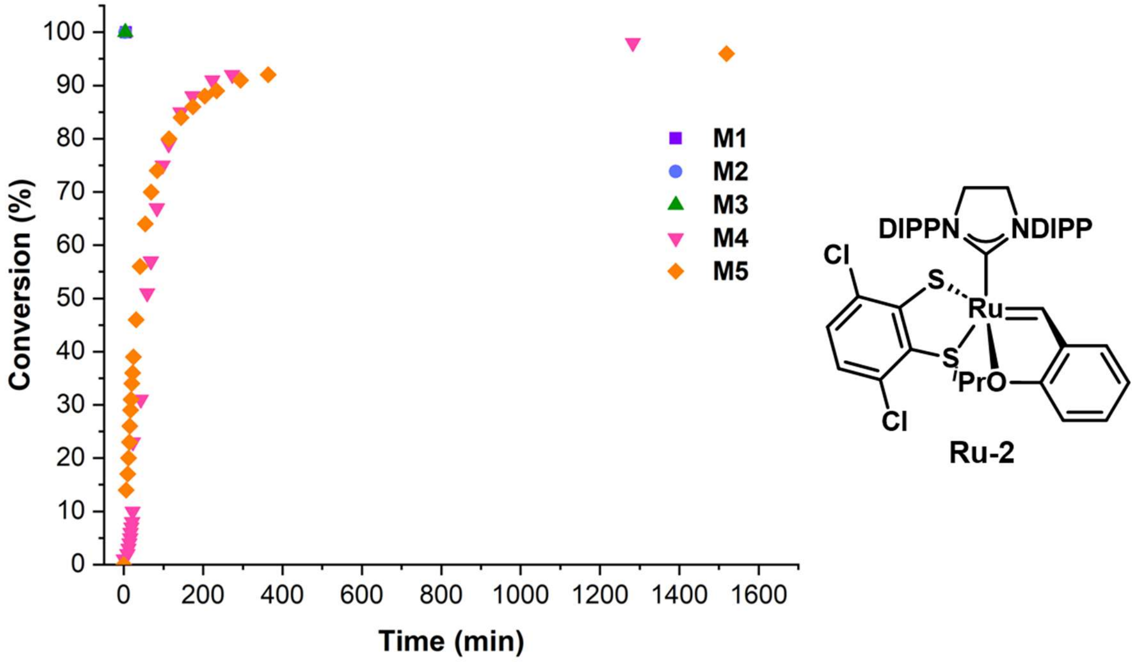

Figure S35. Overlayed conversion plots of **M1–M5** polymerized with **Ru-2** in C_6D_6 at rt.

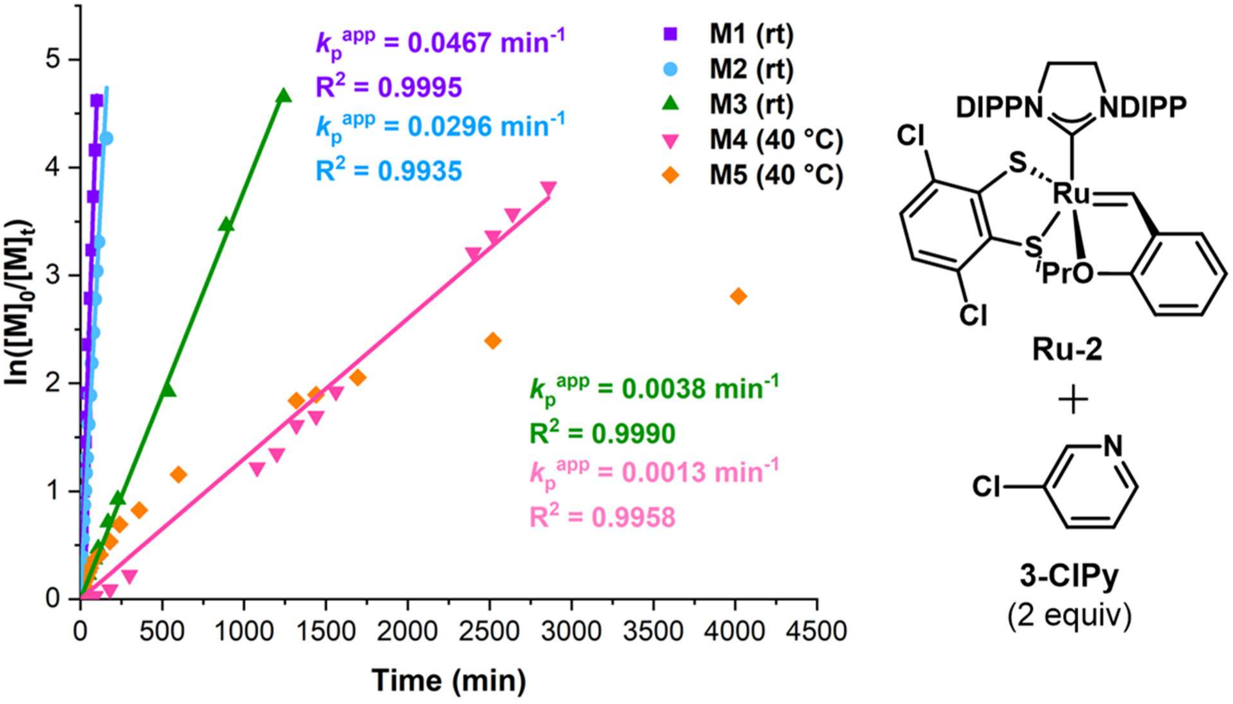

Figure S36. Overlayed kinetics plots of **M1–M5** polymerized with **Ru-2 + 3-ClPy** in C_6D_6.

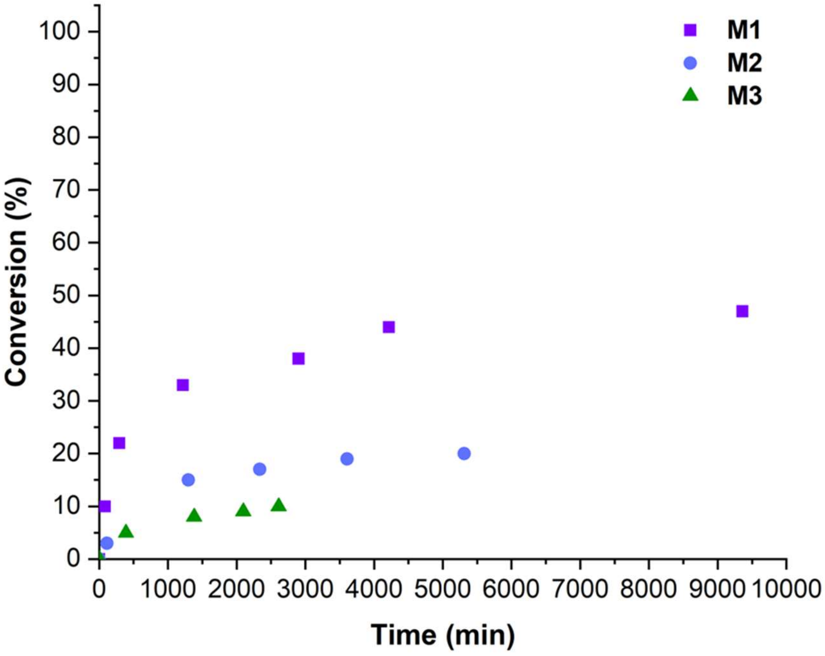

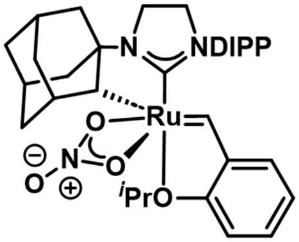

Ru-3a

Figure S37. Overlayed conversion plots of **M1–M3** polymerized with **Ru-3a** in C_6D_6 at 70 °C.

Table S1. Summary of Polymerization Data using **Ru-2**, **Ru-2 + 3-ClPy**, and **Ru-3a** in C_6D_6

Entry	Monomer	Ru	*T* (°C)	Time (min)	Conv. (%)	M_n^{theo} (kg/mol)	M_n^{exp} (kg/mol)[a]	*Đ*	*cis* (%)[b]
1	**M1**	**Ru-2**	rt	5	>99	6.5	45.7	1.50	>99
2	**M2**	**Ru-2**	rt	3	>99	9.3	18.5[c]	–	>99
3	**M3**	**Ru-2**	rt	4	>99	9.3	28.8	1.46	>99
4	**M4**	**Ru-2**	rt	1283	>99	8.7	32.5	1.52	>99
5	**M5**	**Ru-2**	rt	1519	96	9.6	16.7	1.70	>99
6	**M1**	**Ru-2**[d]	rt	100	>99	6.5	6.9	1.08	>99
7	**M2**	**Ru-2**[d]	rt	160	>99	9.3	6.7	1.09	>99
8	**M3**	**Ru-2**[d]	rt	1242	>99	9.3	11.4	1.06	>99
9	**M4**	**Ru-2**[d]	40	2920	>99	8.7	8.7	1.16	>99
10	**M5**	**Ru-2**[d]	40	4560	95[e]	9.6	12.1	1.41	>99
11	**M1**	**Ru-3a**	70	9360	47[e]	3.3	3.1	1.33	70
12	**M2**	**Ru-3a**	70	5310	20[e]	1.7	2.7	1.33	72
13	**M3**	**Ru-3a**	70	2610	10[e]	0.9	4.8	1.48	68

[a]Determined by SEC analysis in THF against polystyrene standards. [b]Measured by ^{1}H NMR spectroscopy. [c]Determined by ^{1}H NMR chain-end analysis due to polymer insolubility in THF. [d]2 equiv of 3-ClPy used as an additive. [e]Full catalyst decomposition observed.

Diagnostic ¹H NMR Alkylidene Signals

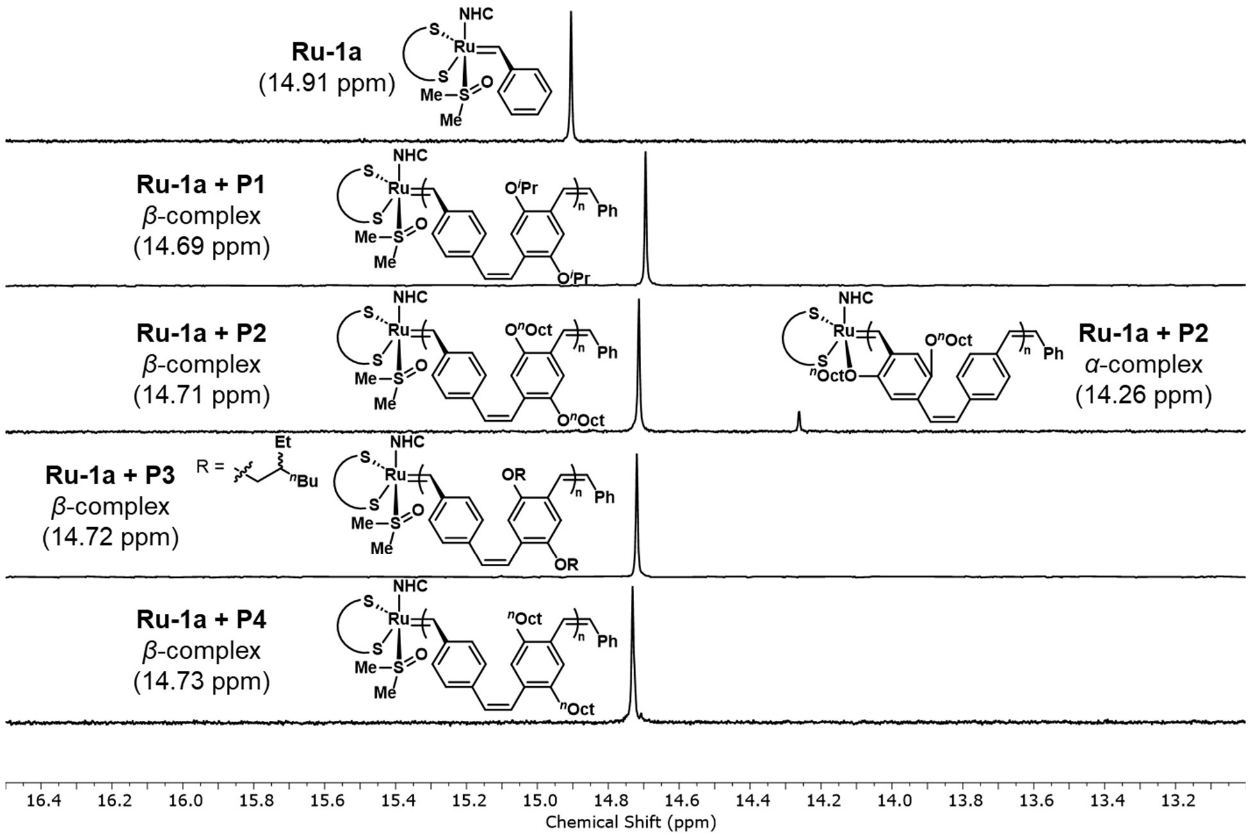

Figure S38. ¹H NMR spectra showing propagating **Ru-1a** alkylidene signals in C_6D_6.

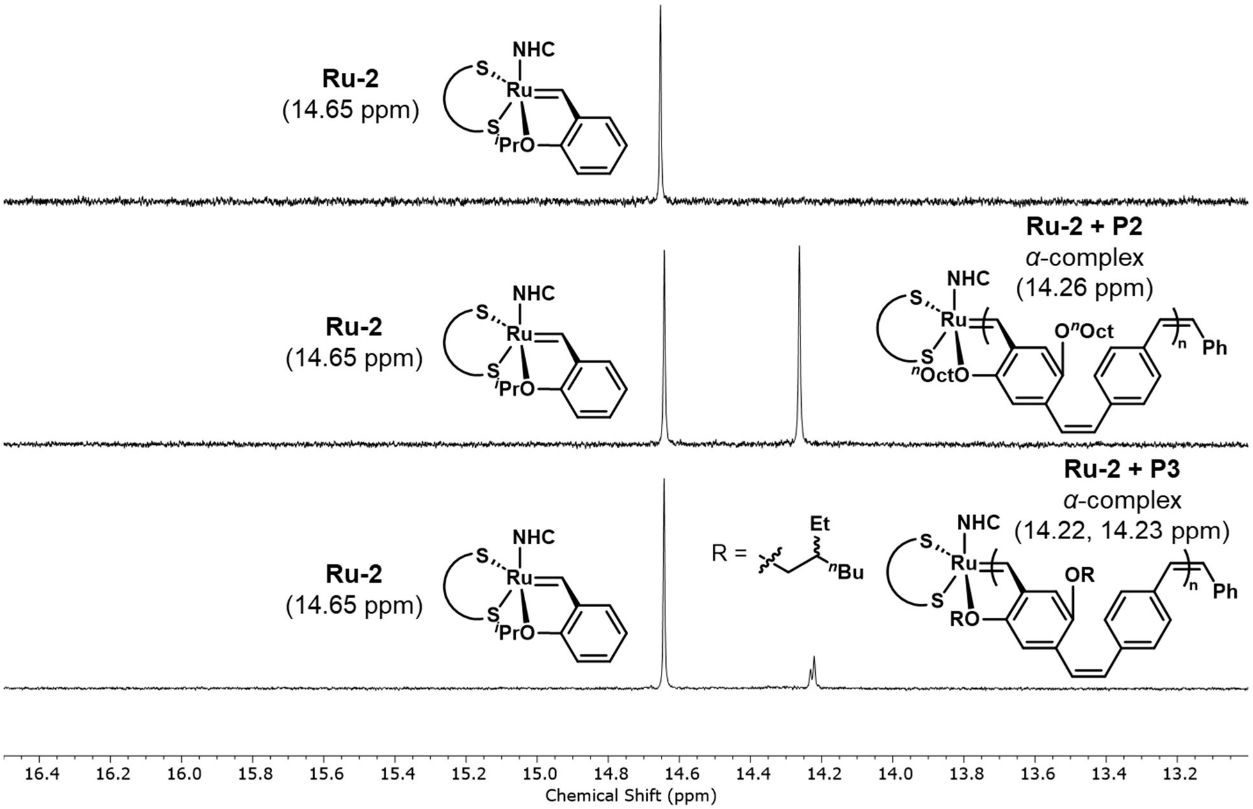

Figure S39. ^{1}H NMR spectra showing differences in **Ru-2** alkylidene signal shifts between **M2** and **M3** in C_6D_6 after full monomer conversion. The two signals for **Ru-2 + P3** are postulated to result from the chirality of the 2-ethylhexyloxy sidechain.

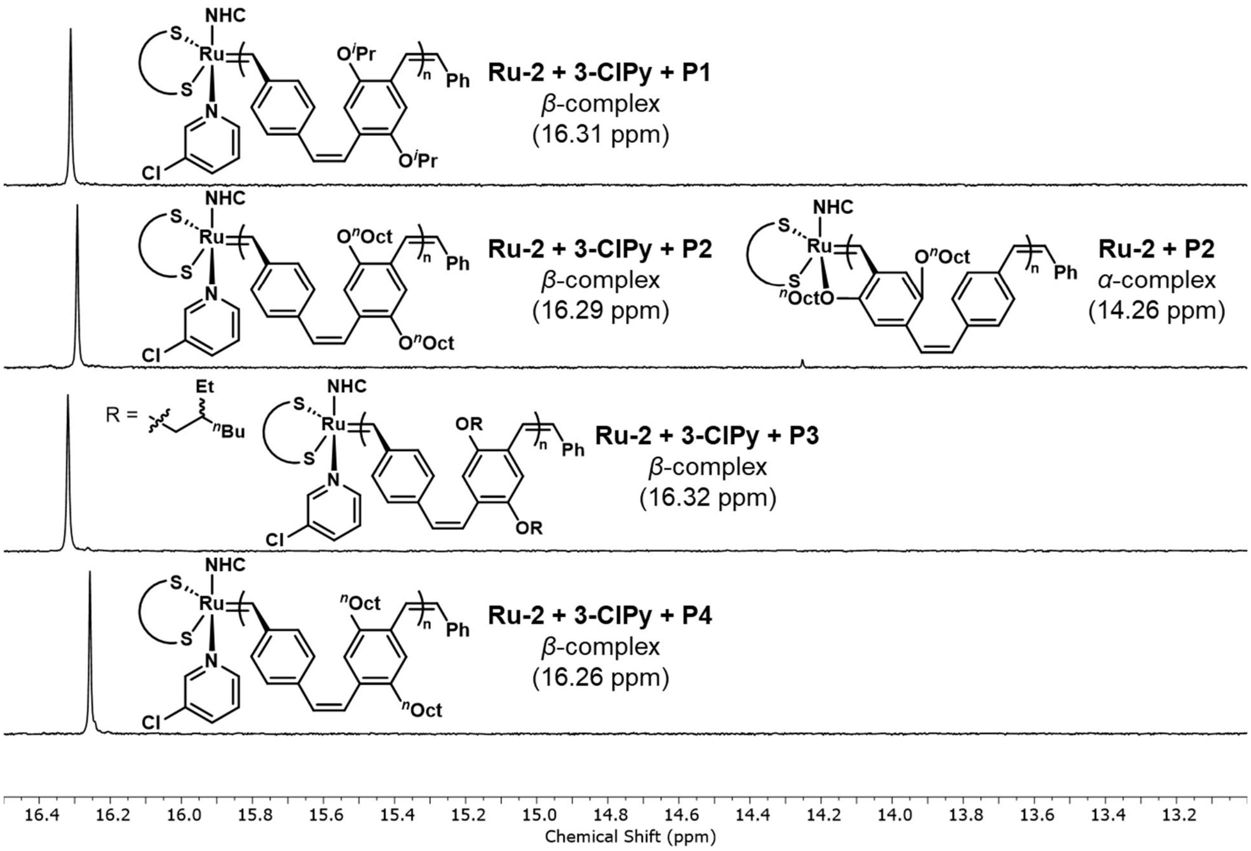

Figure S40. ^{1}H NMR spectra showing **Ru-2 + 3-ClPy** propagating alkylidene shifts in C_6D_6.

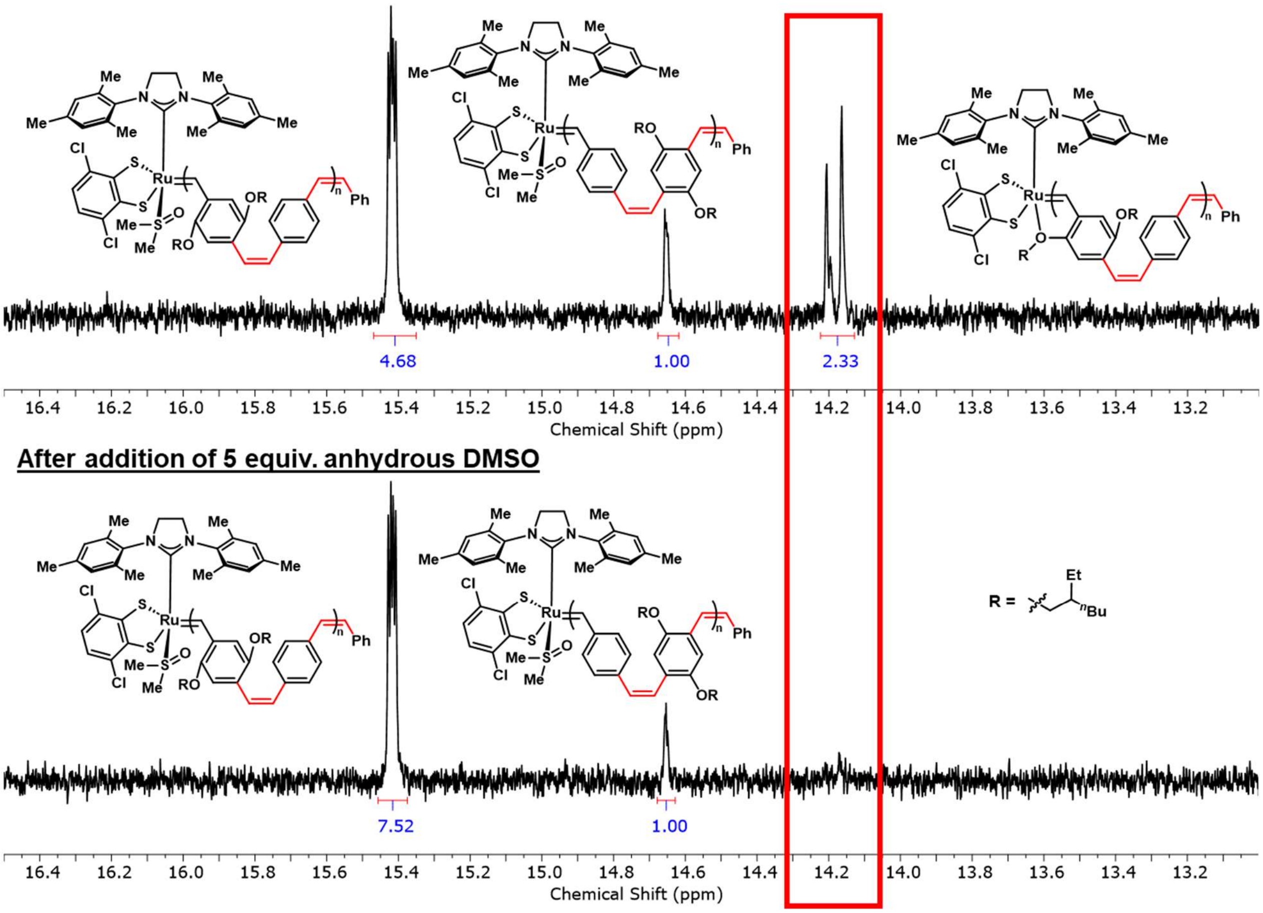

Figure S41. Overlayed ^{1}H NMR spectra showing disappearance of coordination after addition of 5 equivalence of anhydrous DMSO to the polymerization of **M3** with **Ru-1b** at 97% conversion in sealed in a J-Young tube under nitrogen in C_6D_6.

Ring-Opening Metathesis Procedures

Determination of Regioselectivity through Ring-Opening Metathesis of **M1** with **Ru-1a**

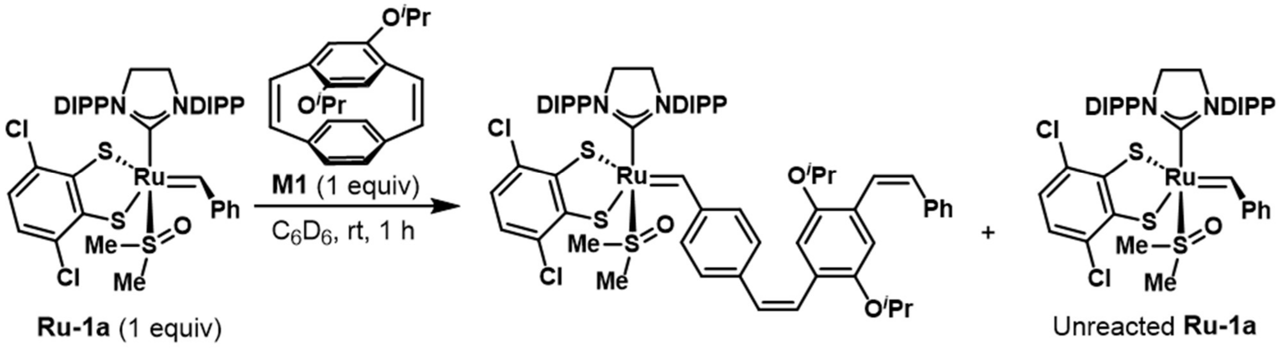

Inside a nitrogen-filled glovebox, **Ru-1a** (0.01 mmol, 8.7 mg, 1 equiv) was added to a small vial, followed by **M1** (0.01 mmol, 3.2 mg, 1 equiv) dissolved in C_6D_6 (0.5 mL). After quickly mixing, the solution was then added to a J-young tube and sealed. The reaction was left for 1 hour in the dark at rt before taking ^{1}H NMR and NOESY.

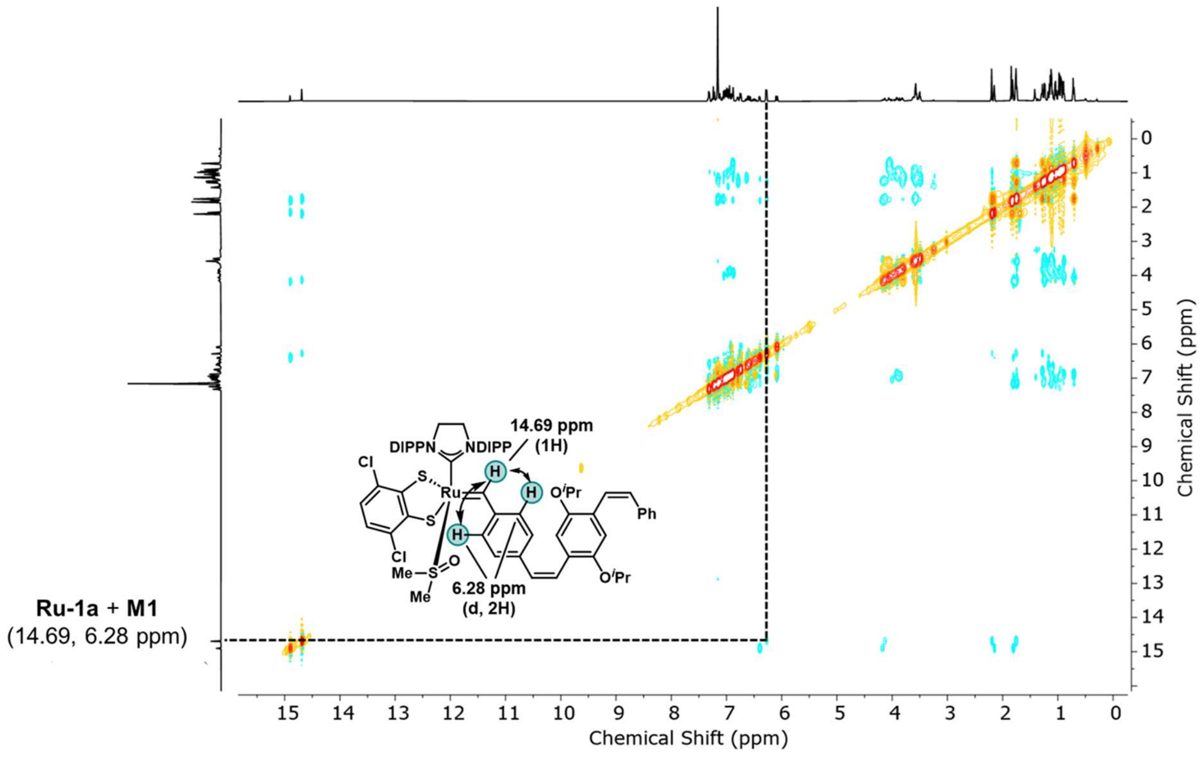

Figure S42. Full NOESY spectrum using **Ru-1a** and **M1** (1 equiv) in C_6D_6.

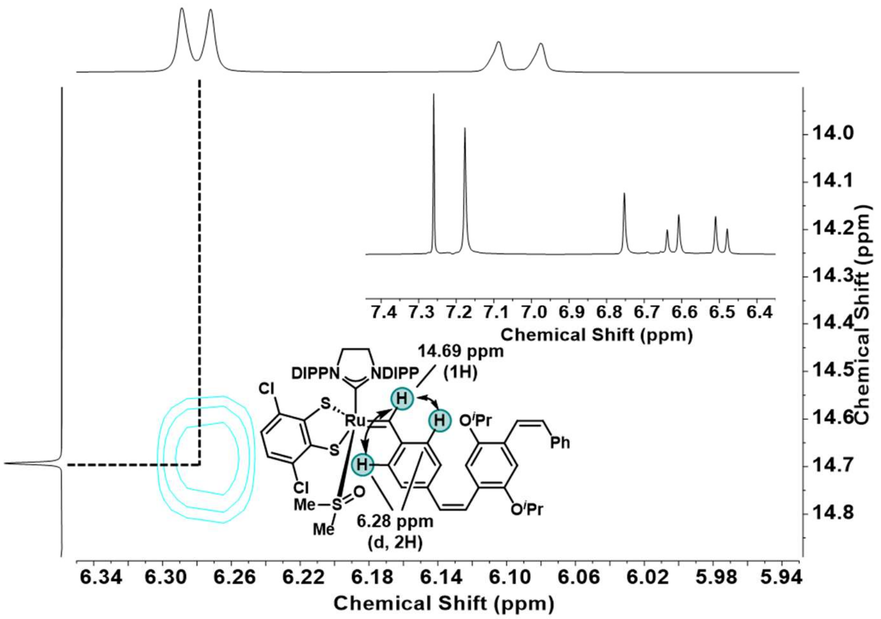

Figure S43. Zoomed in NOESY spectrum of alkylidene and aromatic region using **Ru-1a** and **M1** (1 equiv) in C_6D_6. Inset shows ^1H NMR of isolated *cis*-**P1** aromatic region.

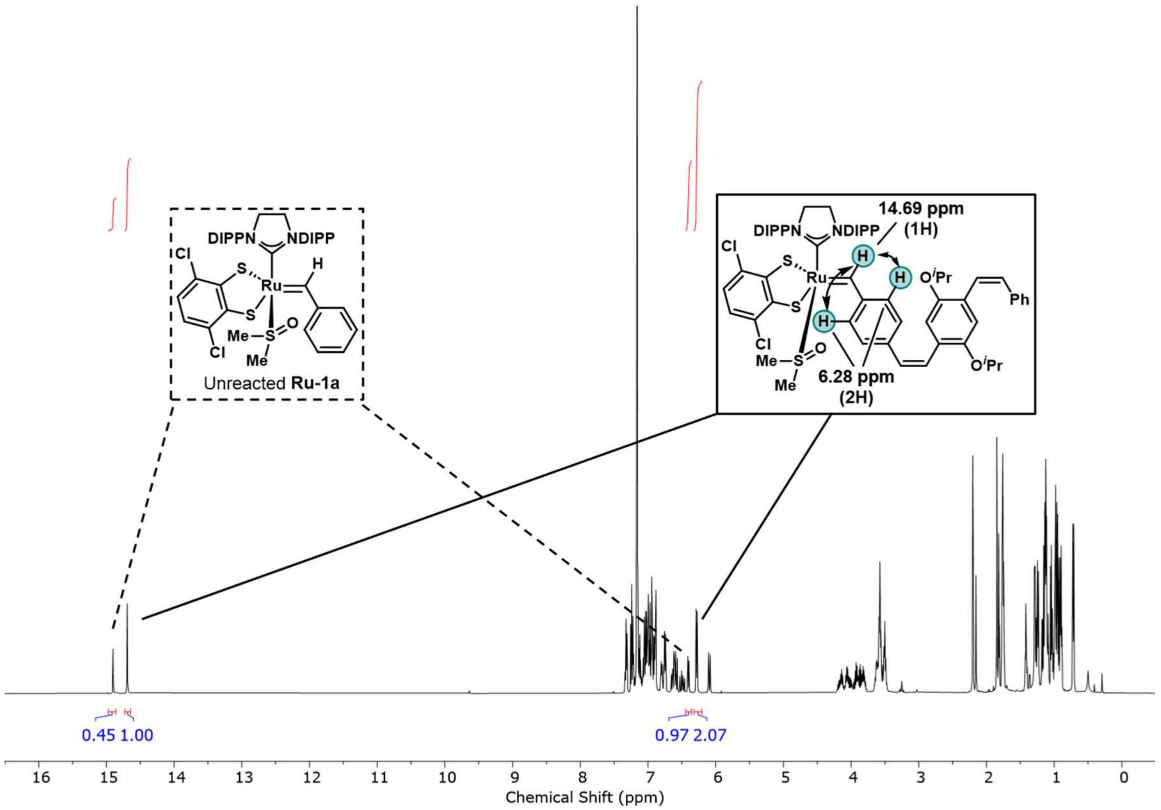

Figure S44. ^1H NMR spectrum of ring-opening metathesis using **Ru-1a** and **M1** (1 equiv) in C_6D_6. Relative integral ratios between corresponding alkylidene and aromatic signals found by NOESY support the predicted ratios.

Copolymer Synthesis

One-Shot Synthesis of *cis*-**P1**-*b*-*cis*-**P4**

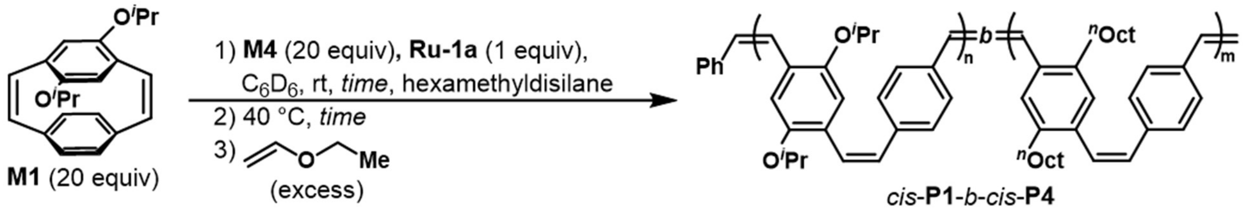

Inside a nitrogen-filled glovebox, **Ru-1a** (2.2 mg, 0.0025 mmol, 1.0 equiv) was weighed into a vial. A hexamethyldisilane stock solution (2.2 mg dissolved in 0.6 mL of C_6D_6) was prepared, from which a measured amount (0.1 mL, 0.0025 mmol, 1.0 equiv) was added to the catalyst vial. Next, monomers **M1** (16.0 mg, 0.050 mmol, 20.0 equiv) and **M4** (21.4 mg, 0.050 mmol, 20.0 equiv) were each separately dissolved in C_6D_6 (0.25 mL) and mixed before transferring to the reaction vial. After quickly mixing, the solution was transferred into a J-Young tube and sealed while inside the glovebox. The reaction was monitored *in-situ* by ^{1}H NMR at rt in the dark. After full conversion of **M1**, the reaction was brought back inside a nitrogen-filled glovebox where an aliquot (0.1 mL) of the sample was removed and quenched using excess ethyl vinyl ether (0.1 mL) for SEC analysis. The remainder of the reaction was monitored *in-situ* by ^{1}H NMR at 40 °C in the dark. The reaction was quenched inside a nitrogen-filled glovebox using excess ethyl vinyl ether (0.1 mL). After leaving to sit for 30 minutes at rt, two cycles of precipation with addition of methanol (~10 mL), centrifugation, and decantation afforded the polymer *cis*-**P1**-*b*-*cis*-**P4** with residual solvent. The polymer vial was wrapped in aluminum foil, dried under reduced pressure followed by high vacuum, and stored in the dark under a nitrogen atmosphere at –20 °C to prevent any undesired photoisomerization.

First Block: M_n^{theo} = 6.5 kg/mol, M_n^{exp} = 5.8 kg/mol, $Đ$ = 1.07

Full Polymer: M_n^{theo} = 15.0 kg/mol, M_n^{exp} = 16.7 kg/mol, $Đ$ = 1.24

^{1}H NMR (500 MHz, $CDCl_3$) δ 7.20–7.15 (s, 4 H), 7.00–6.91 (m, 6 H), 6.76–6.72 (s, 2 H), 6.67–6.58 (m, 4 H), 6.53–6.44 (m, 4 H), 4.07–3.94 (m, 2 H), 2.49–2.36 (m, 4 H), 1.46–1.36 (m, 4 H), 1.29–1.16 (m, 24 H), 1.23–1.07 (d, J = Hz, 12 H) 0.89–0.80 (t, *J* = Hz, 6 H) ppm.

^{13}C NMR (126 MHz, $CDCl_3$) δ 149.7, 138.4, 136.4, 135.9, 135.8, 130.4, 130.2, 129.6, 128.9, 128.1, 126.1, 117.2, 72.0, 33.2, 32.1, 30.9, 29.9, 29.7, 29.4, 22.8, 22.3, 14.3 ppm.

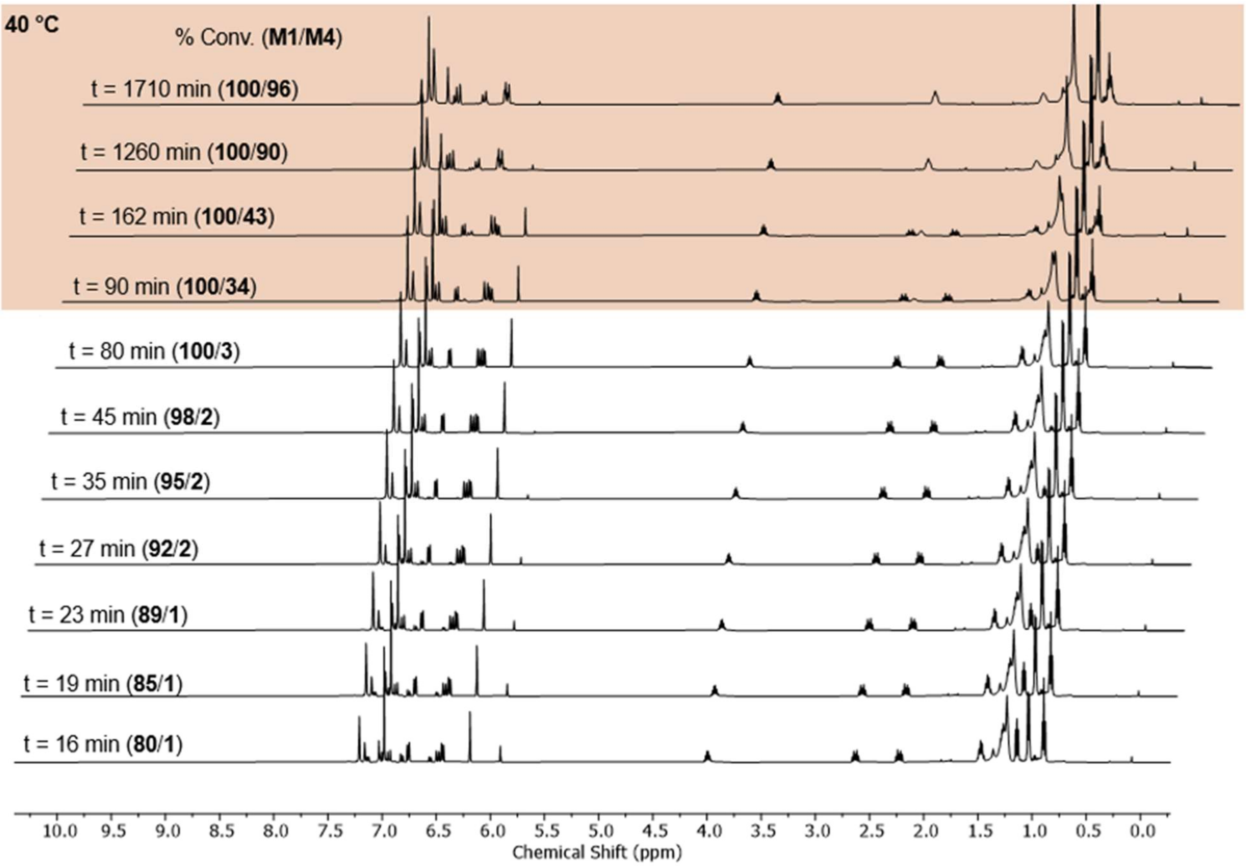

Figure S45. ^{1}H NMR spectra showing conversion of **M1** and **M4** over time in C_6D_6. Conversion of **M1** was determined through comparing changes in integration of **P1** aromatic signal (7.21 ppm, 4 H) and **M1** aromatic signal (5.91 ppm, 2 H) over time. Conversion of **M4** was determined through comparing changes in integration of **P4** aromatic signal (7.28 ppm, 2 H) and **M4** aromatic signal (6.19 ppm, 2 H) over time.

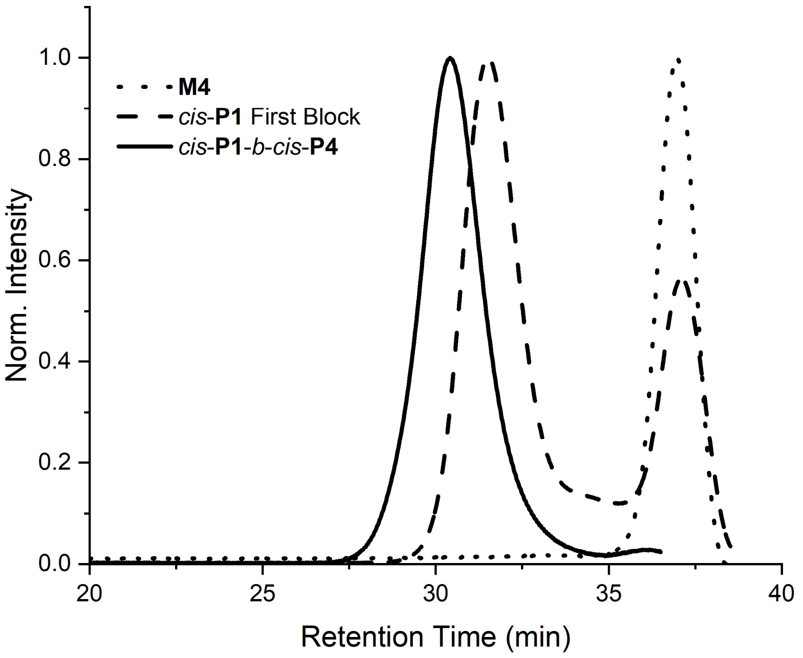

Figure S46. SEC traces showing chain extension after heating to 40 °C.

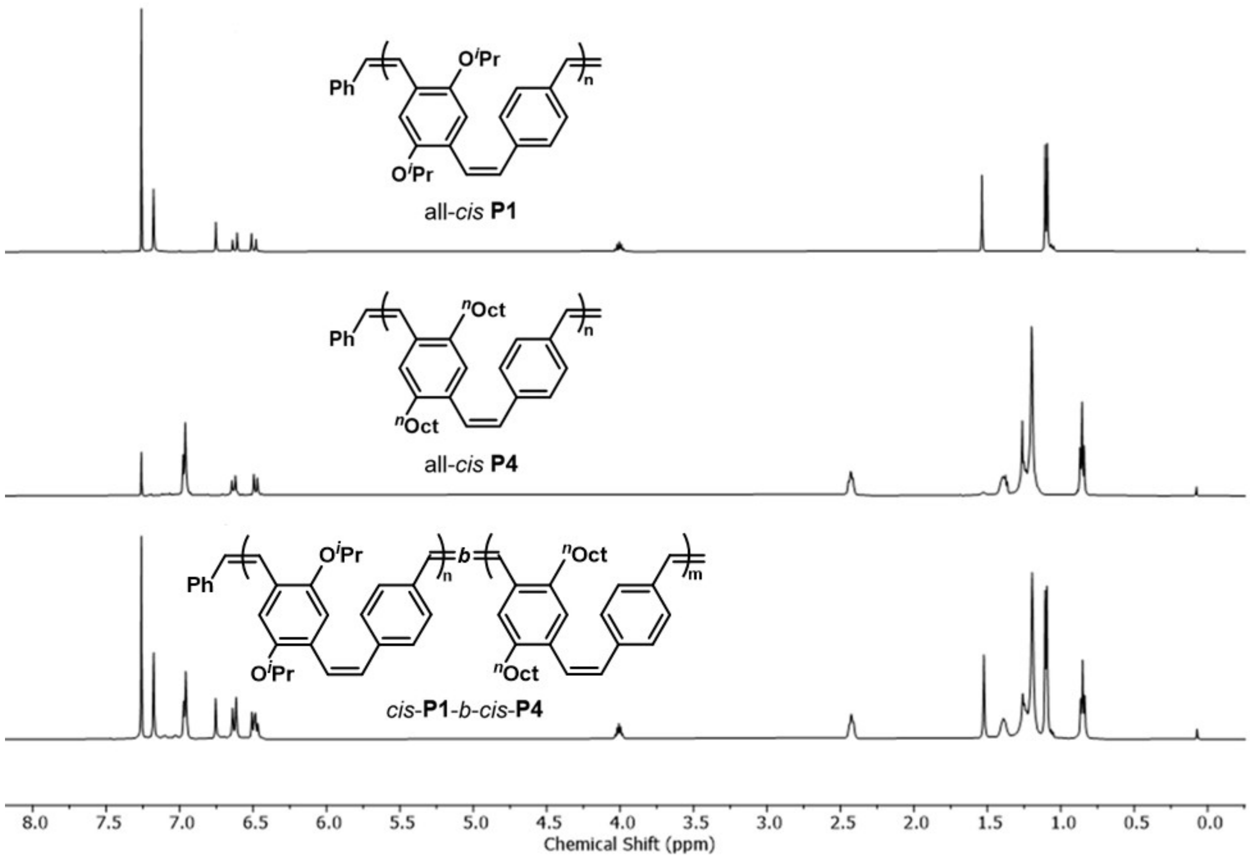

Figure S47. Overlayed ^{1}H NMR spectra comparison of homopolymers all-*cis* **P1** (top) and all-*cis* **P4** (middle), and isolated copolymer *cis*-**P1**-*b*-*cis*-**P4** (bottom) in $CDCl_3$.

Synthesis of *cis*-**P1**-*grad*-*cis*-**P3**

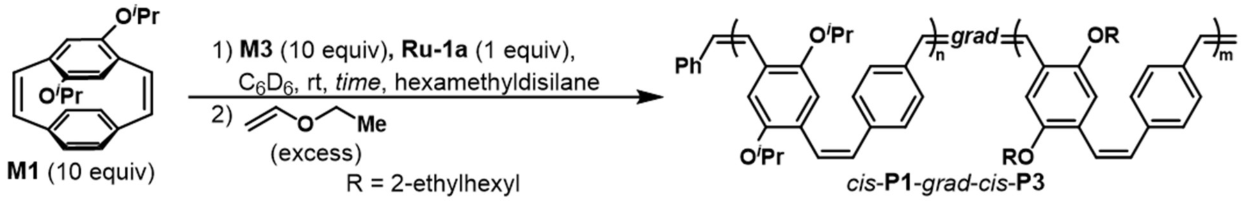

Inside a nitrogen-filled glovebox, **Ru-1a** (2.2 mg, 0.0025 mmol, 1.0 equiv) was weighed into a vial. A hexamethyldisilane stock solution (2.2 mg dissolved in 0.6 mL of C_6D_6) was prepared, from which a measured amount (0.1 mL, 0.0025 mmol, 1.0 equiv) was added to the catalyst vial. Next, monomers **M1** (8.0 mg, 0.025 mmol, 10.0 equiv) and **M3** (11.6 mg, 0.025 mmol, 10.0 equiv) were each seperately dissolved in C_6D_6 (0.2 mL) and mixed before transferring to the reaction vial. After quickly mixing, the solution was transferred into a J-Young tube and sealed while inside the glovebox. The reaction was monitored *in-situ* by ^{1}H NMR at rt in the dark. The reaction was quenched inside a nitrogen-filled glovebox using excess ethyl vinyl ether (0.1 mL). After leaving to sit for 30 minutes at rt, two cycles of precipation with addition of methanol (~10 mL), centrifugation, and decantation afforded the polymer *cis*-**P1**-*grad*-*cis*-**P3** with residual solvent. The polymer vial was wrapped in aluminum foil, dried under reduced pressure followed by high vacuum, and stored in the dark under a nitrogen atmosphere at –20 °C to prevent any undesired photoisomerization.

M_n^{theo} = 7.9 kg/mol, M_n^{exp} = 9.5 kg/mol, $Đ$ = 1.17

^{1}H NMR (500 MHz, $CDCl_3$) δ 7.24–7.14 (m, 8 H), 6.78–6.73 (m, 4 H), 6.68–6.59 (m, 4 H), 6.54–6.45 (m, 4 H), 4.06–3.91 (m, 2 H), 3.46–3.35 (m, 4 H), 1.59–1.46 (m, 4 H), 1.39–1.14 (m, 16 H), 1.13–1.02 (m, 10 H), 0.89–0.84 (t, J = 7.1 Hz, 6 H), 0.83–0.75 (m, 6 H) ppm.

^{13}C NMR (126 MHz, $CDCl_3$) δ 150.5, 150.5, 149.7, 136.5, 136.5, 136.4, 129.5, 129.4, 129.3, 129.0, 128.9, 128.8, 128.4, 128.1, 128.0, 126.3, 126.3, 126.2, 126.1, 125.5, 117.2, 117.2, 114.0, 114.0, 72.0, 72.0, 71.5, 71.4, 39.4, 39.4, 30.7, 29.1, 24.0, 23.2, 22.3, 14.2, 11.2, 11.2 ppm.

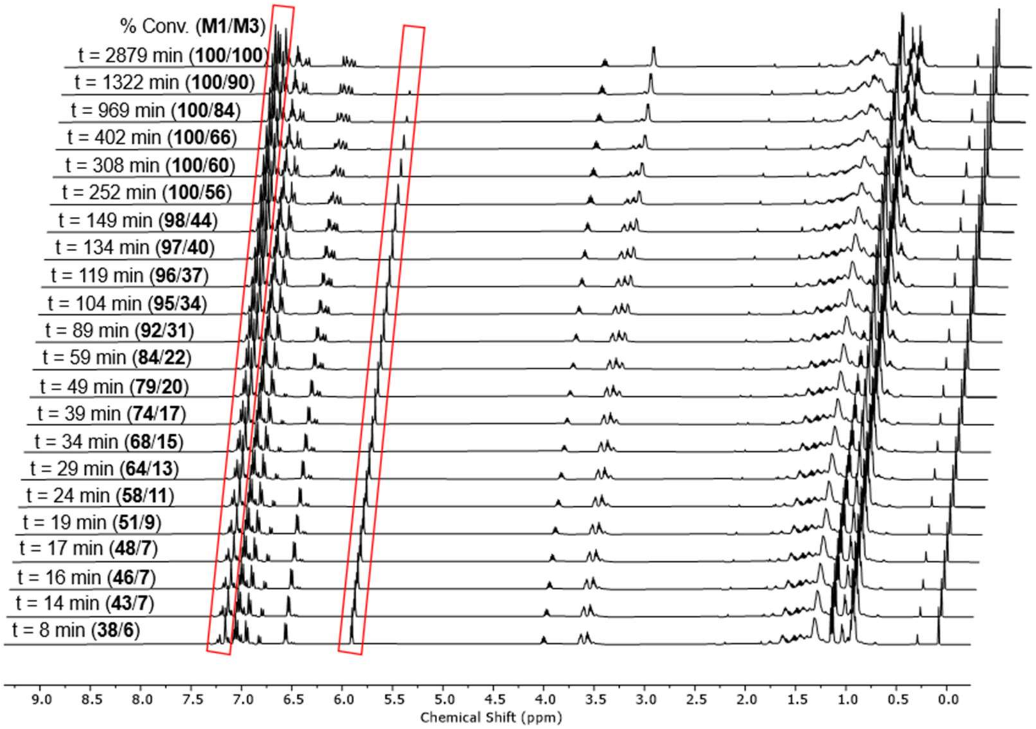

Figure S48. ^{1}H NMR spectra showing conversion of **M1** and **M3** over time in C_6D_6. Conversion determined through comparing changes in integration (assisted by peak deconvolution) of a **M1** aromatic signal (5.91 ppm, 2 H), **M3** aromatic signal (5.90 ppm, 2 H), and *cis*-**P1**-*grad*-*cis*-**P3** aromatic signals (7.27 ppm, 4 H; 7.24 ppm; 4 H; 7.22 ppm, 4 H).

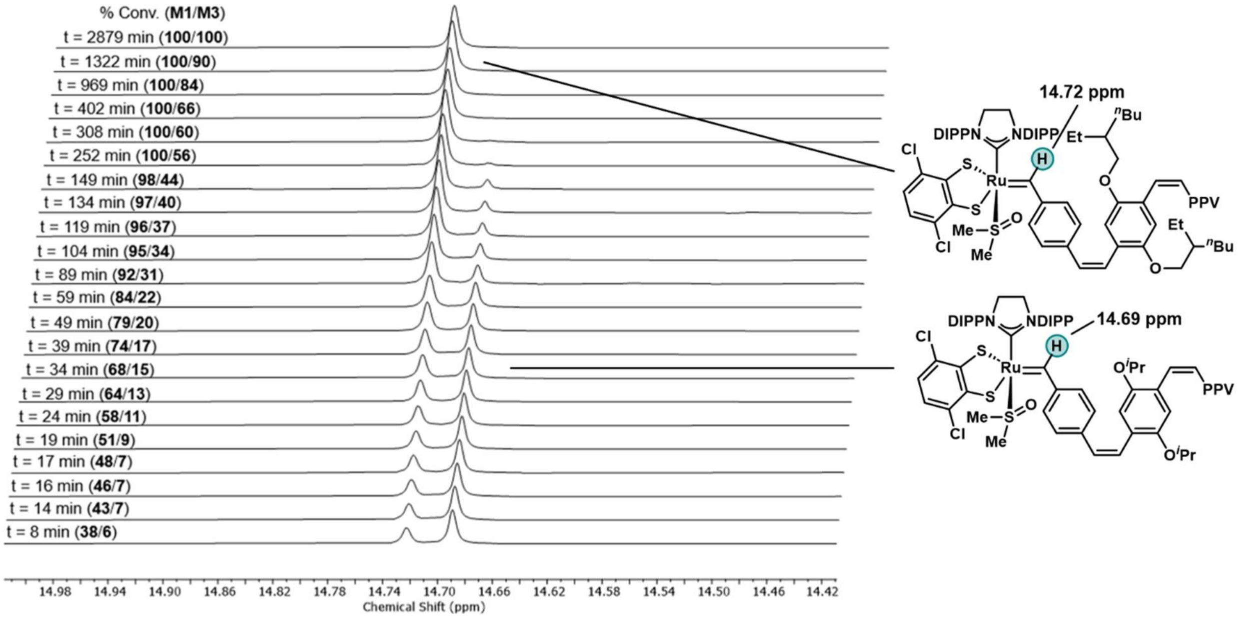

Figure S49. ^{1}H NMR spectra showing change in alkylidene signals over time in C_6D_6.

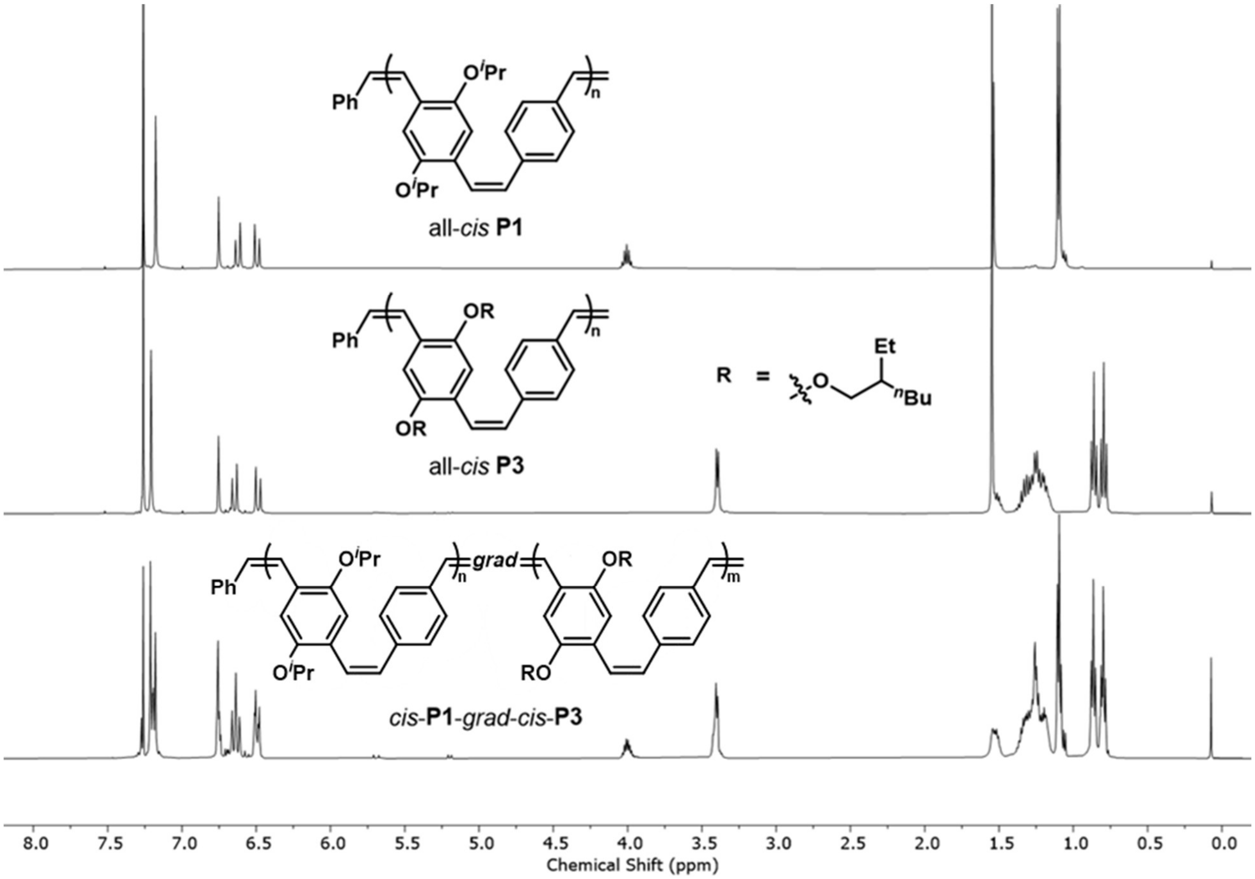

Figure S50. Overlayed ^{1}H NMR spectra of homopolymers all-*cis* **P1** (top) and all-*cis* **P3** (middle), and isolated copolymer *cis*-**P1**-*grad*-*cis*-**P3** (bottom) in $CDCl_3$.

Stereoblock Synthesis of *trans*-**P3**-*b*-*cis*-**P3**

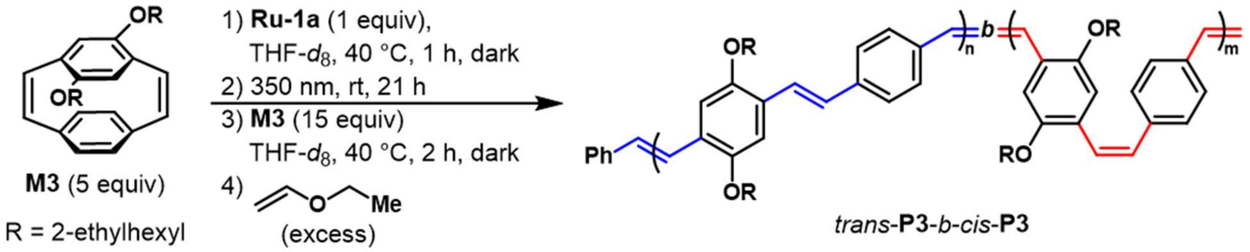

Inside a nitrogen-filled glovebox, **Ru-1a** (2.2 mg, 0.0025 mmol, 1.0 equiv) was weighed into a vial and dissolved in THF-d_8 (0.3 mL). A hexamethyldisilane stock solution (2.2 mg dissolved in 0.6 mL of THF-d_8) was prepared, from which a measured amount (0.1 mL, 0.0025 mmol, 1.0 equiv) was added to the catalyst solution. Next, a stock solution of monomer **M3** (23.1 mg, 0.050 mmol) in THF-d_8 (0.4 mL) was prepared, from which a measured amount (0.1 mL, 5.0 equiv) was transferred to the reaction solution. The solution was then quickly mixed before transfering into a J-Young tube and sealing inside the glovebox. The reaction was left in the dark for 1 hour at 40 °C. Photoisomerization at rt using two 350 nm UV lamps, each placed 1.0 cm on either side of the J-young tube, was monitored *in-situ* by ^{1}H NMR. After 21 hours of irradiation, the reaction was brought back inside a nitrogen-filled glovebox where an aliquot (0.1 mL) of the sample was removed and quenched using excess ethyl vinyl ether (0.1 mL) for SEC analysis. Then, more of the stock solution containing **M3** in THF-d_8 (0.24 mL, now 15.0 equiv) was mixed with the reaction solution. The reaction was left at 40 °C for 2 hours in the dark. The reaction was then quenched inside a nitrogen-filled glovebox using excess ethyl vinyl ether (0.1 mL). After leaving to sit for 30 minutes at rt, two cycles of precipation with addition of methanol (~10 mL), centrifugation, and decantation afforded the polymer *trans*-**P3**-*b*-*cis*-**P3** with residual solvent. The polymer vial was wrapped in aluminum foil, dried under reduced pressure followed by high vacuum, and stored in the dark under a nitrogen atmosphere at –20 °C to prevent any undesired photoisomerization.

First block: M_n^{theo} = 2.4 kg/mol, M_n^{exp} = 4.7 kg/mol, $Đ$ = 1.28

Full polymer: M_n^{theo} = 9.3 kg/mol, M_n^{exp} = 11.9 kg/mol, $Đ$ = 1.20

^{1}H NMR (500 MHz, $CDCl_3$) δ 7.59–7.34 (m, 6 H), 7.24–7.19 (s, 12 H), 7.18–7.06 (m, 4 H), 6.79–6.73 (s, 6 H), 6.68–6.62 (d, *J* =12.3 Hz, 6 H), 6.53–6.45 (d, *J* = 12.3 Hz, 6 H), 4.04–3.85 (m, 4 H), 3.44–3.36 (m, 12 H), 1.91–1.77 (m, 2 H), 1.67–1.56 (m, 6 H), 1.44–1.14 (m, 74 H), 1.05–0.70 (m, 62 H) ppm.

^{13}C NMR (125 MHz, $CDCl_3$) δ 151.5, 150.5, 137.4, 136.5, 129.4, 128.9, 128.5, 127.1, 127.0, 126.2, 125.5, 123.5, 114.0, 110.5, 72.1, 71.4, 40.0, 39.4, 31.2, 30.7, 29.5, 29.1, 24.5, 24.0, 23.3, 23.2, 14.3, 14.2, 11.5, 11.2 ppm.

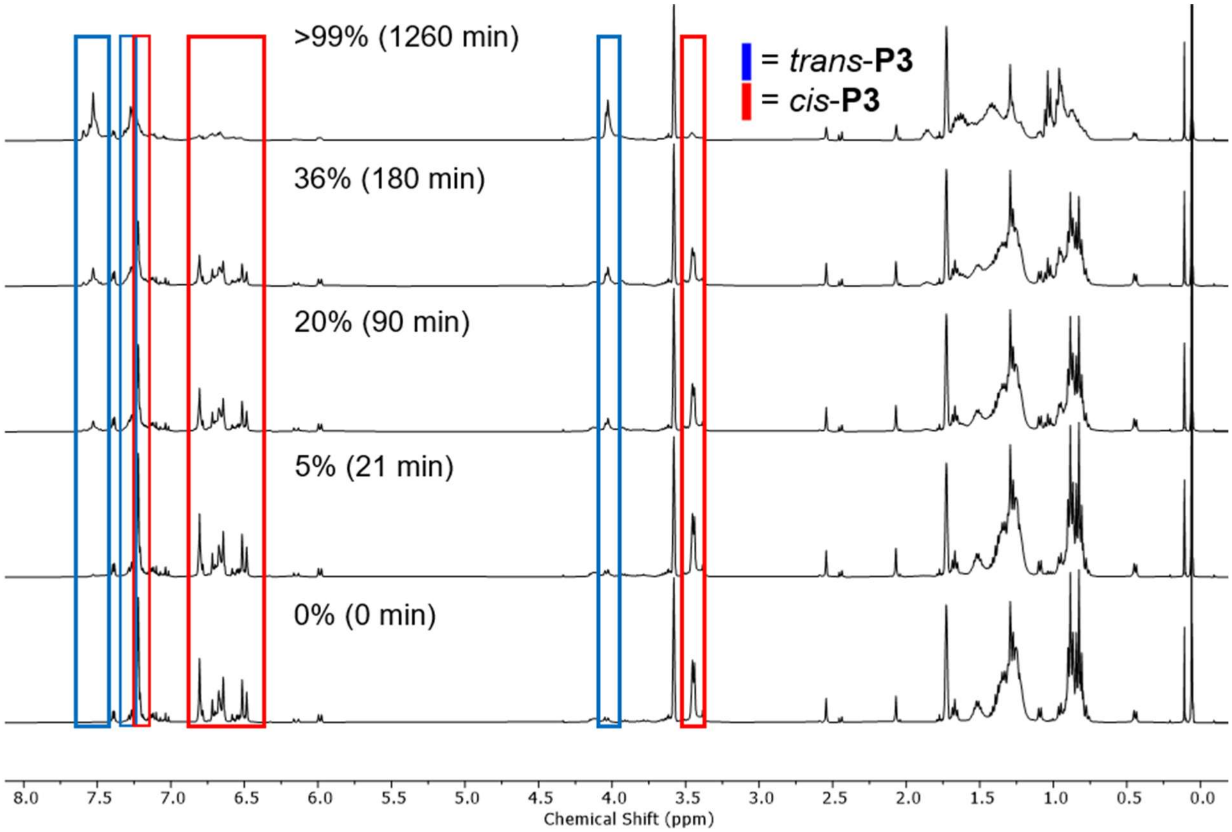

Figure S51. ^{1}H NMR monitoring of *in-situ* photoisomerization in THF-d_8. Conversion determined through comparing changes in integration of a *cis*-**P3** aromatic signal (6.80 ppm, 2 H) and the hexamethyldisilane internal standard signal (0.06 ppm, 18 H) over time.

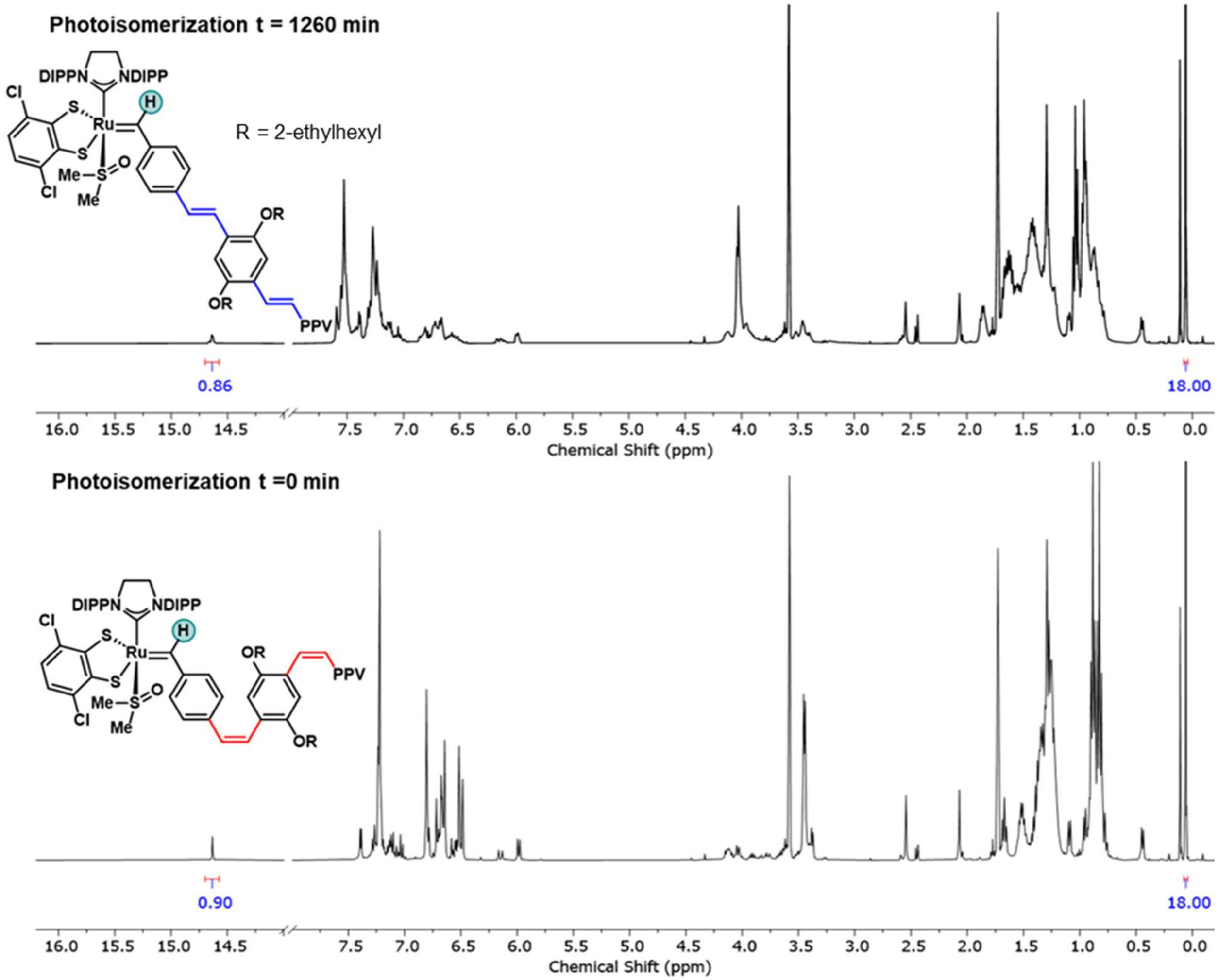

Figure S52. ^{1}H NMR monitoring of alkylidene stability during *in-situ* photoisomerization in THF-d_8. The alkylidene signal (14.63 ppm, 1 H) intensity was compared to the hexamethyldisilane internal standard signal (0.06 ppm, 18 H). Slight broadening of the alkylidene signal was observed and attributed to formation of the *trans* PPV isomer attached to the catalyst.

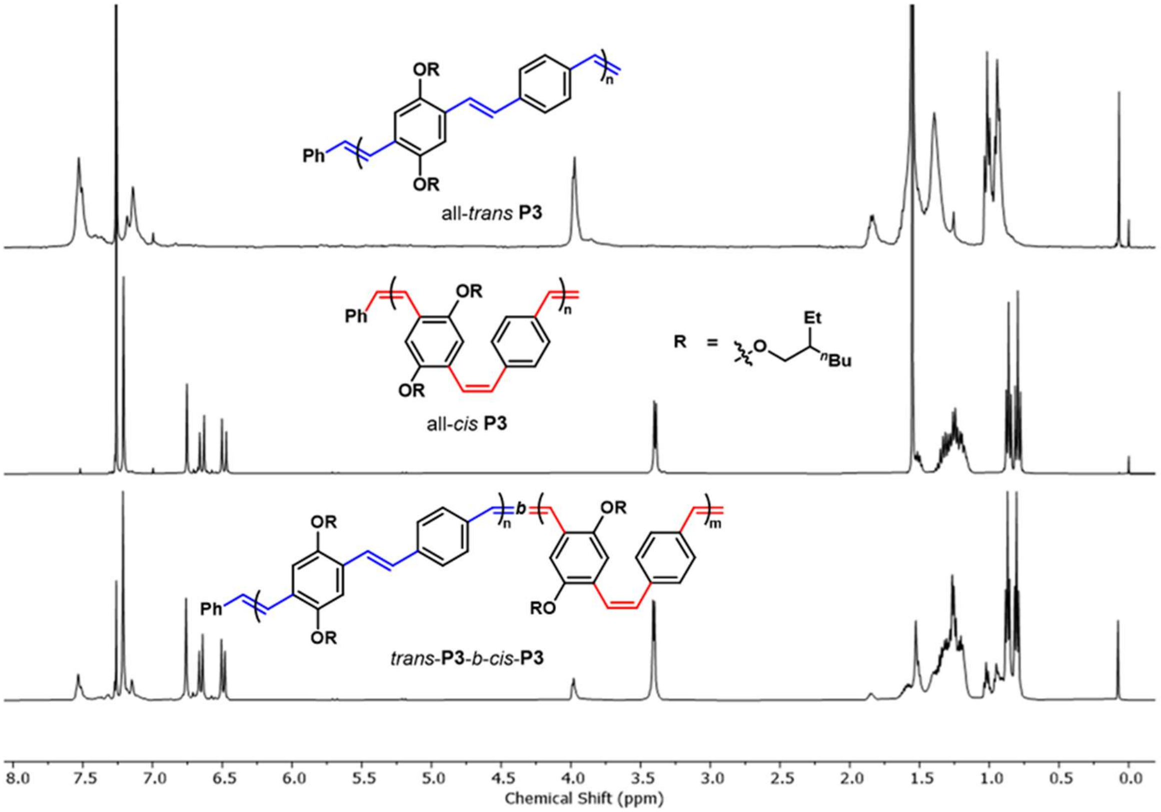

Figure S53. Overlayed ^{1}H NMR spectra comparison of homopolymers all-*trans* **P3** (top) and all-*cis* **P3** (middle), and isolated copolymer *trans*-**P3**-*b*-*cis*-**P3** in $CDCl_3$.

General Photoisomerization Procedure to Access All-*trans* PPV

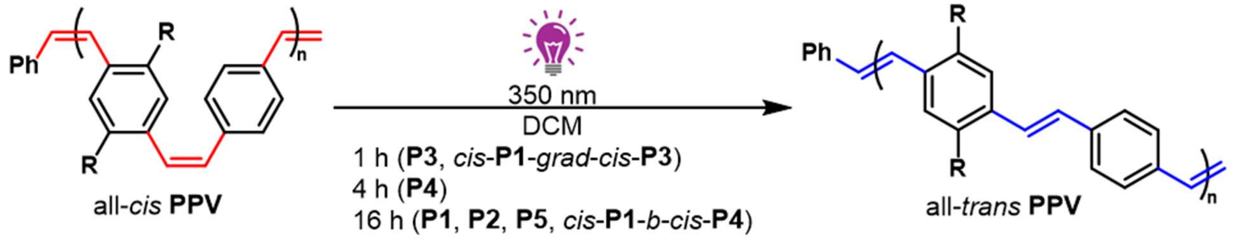

All-*cis* PPV samples were dissolved in DCM in a reaction vial (borosilicate glass) at a concentration of 1 mg/mL. The samples were then irradiated at room temperature using two 350 nm UV lamps placed 1 cm away from the sample (exposure time for each polymer indicated in the scheme above). Following photoisomerization, the samples were wrapped in aluminum foil and concentrated under reduced pressure before further drying under high vacuum. Complete photoisomerization of **P3** and **P4** was confirmed by ^{1}H NMR spectroscopy in $CDCl_3$.[5] Other all-*trans* PPVs could not be characterized using ^{1}H NMR spectroscopy or SEC analysis due to poor solubility in organic solvents and instead photoisomerization was monitored by UV-visible absorption spectroscopy.

Absorption and Emission Spectra

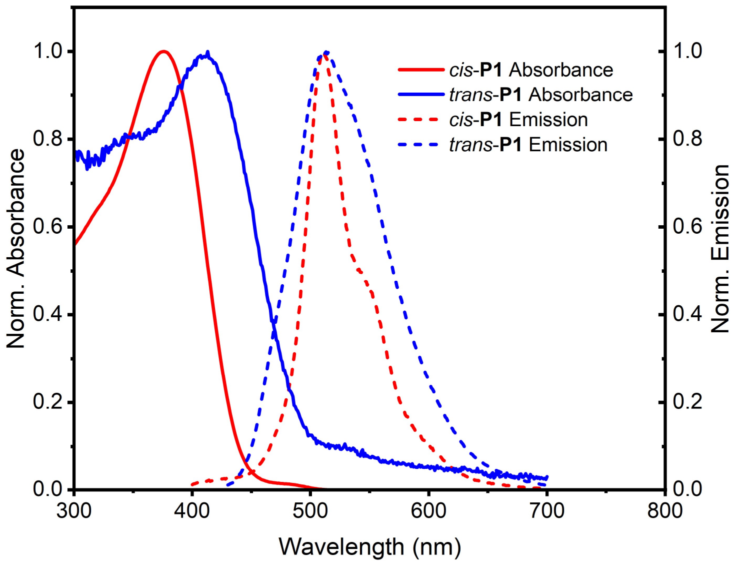

Figure S54. *cis*-**P1** (M_n^{theo} = 6.5 kg/mol, M_n^{exp} = 6.8 kg/mol, *Đ* = 1.09) normalized absorbance (C = 10 μg/mL) and emission (λ_{ex} = 376 nm, C = 0.5 μg/mL) traces in $CHCl_3$. *trans*-**P1** (insolubility prevented SEC analysis in THF) normalized absorbance (C = 0.5 μg/mL) and emission (λ_{ex} = 413 nm, 0.5 μg/mL) traces in $CHCl_3$.

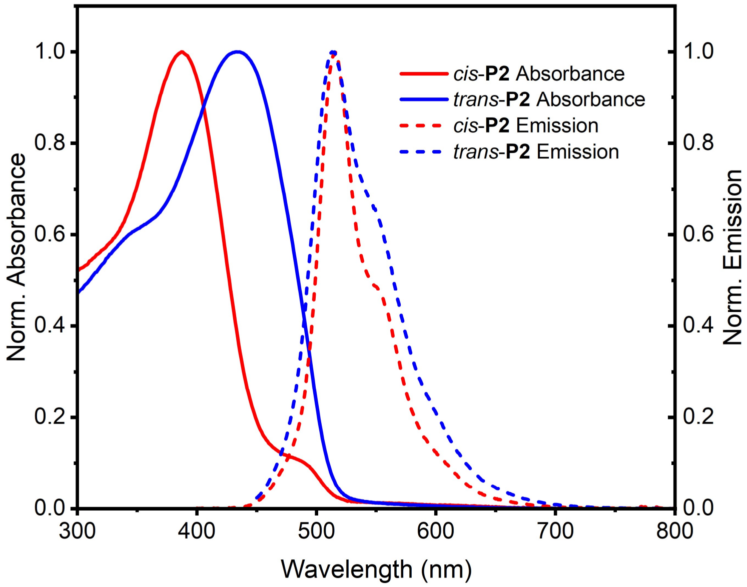

Figure S55. *cis*-**P2** (M_n^{theo} = 9.2 kg/mol, M_n^{exp} = 6.7 kg/mol, *Đ* = 1.09) normalized absorbance (C = 10 μg/mL) and emission (λ_{ex} = 387 nm, C = 0.5 μg/mL) traces in $CHCl_3$. *trans*-**P2** (insolubility prevented SEC analysis in THF) normalized absorbance (C = 10 μg/mL) and emission (λ_{ex} = 433 nm, C = 0.5 μg/mL) traces in $CHCl_3$.

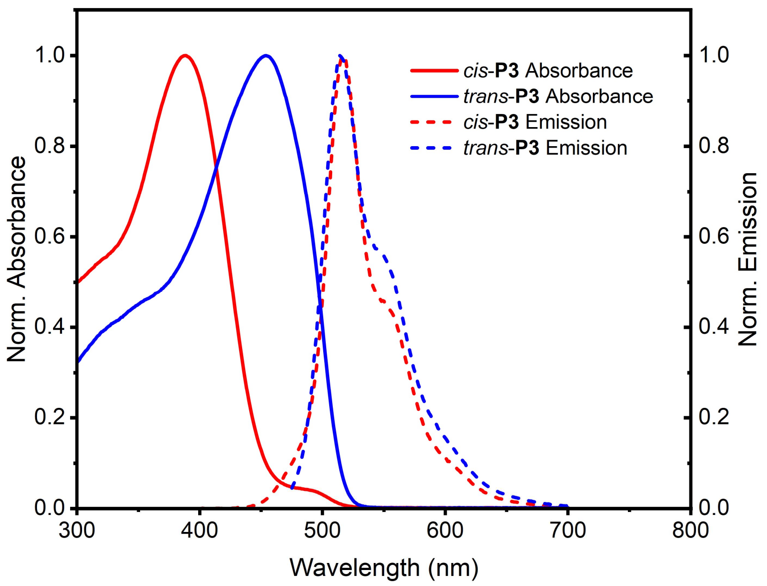

Figure S56. *cis*-**P3** (M_n^{theo} = 9.2 kg/mol, M_n^{exp} = 8.5 kg/mol, $Đ$ = 1.08) normalized absorbance (C = 20 µg/mL) and emission (λ_{ex} = 388 nm, C = 0.5 µg/mL) traces in $CHCl_3$. *trans*-**P3** (M_n^{theo} = 9.2 kg/mol, M_n^{exp} = 20.7 kg/mol, $Đ$ = 1.18) normalized absorbance (C = 10 µg/mL) and emission (λ_{ex} = 454 nm, C = 0.5 µg/mL) traces in $CHCl_3$.

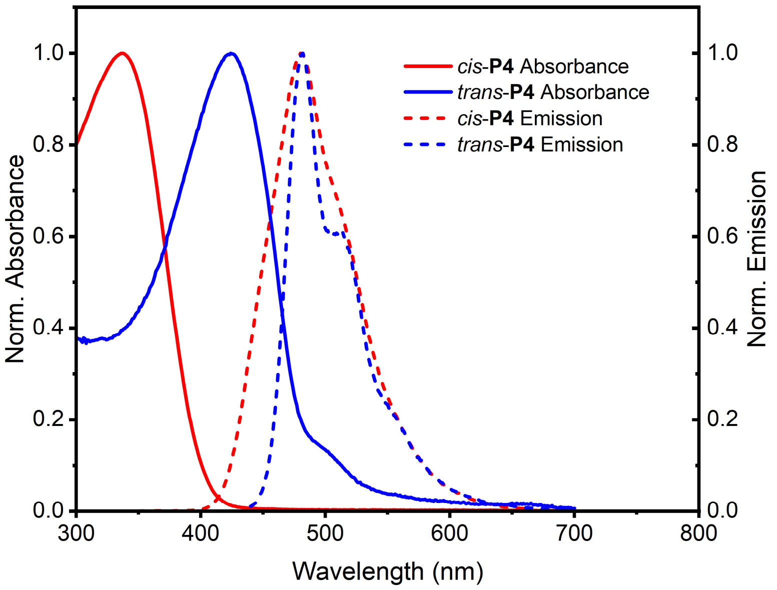

Figure S57. *cis*-**P4** (M_n^{theo} = 8.6 kg/mol, M_n^{exp} = 8.7 kg/mol, $Đ$ = 1.16) normalized absorbance (C = 10 µg/mL) and emission (λ_{ex} = 336 nm, C = 0.5 µg/mL) traces in $CHCl_3$. *trans*-**P4** (insolubility prevented SEC analysis in THF) normalized absorbance (C = 10 µg/mL) and emission (λ_{ex} = 424 nm, C = 0.5 µg/mL) traces in $CHCl_3$.

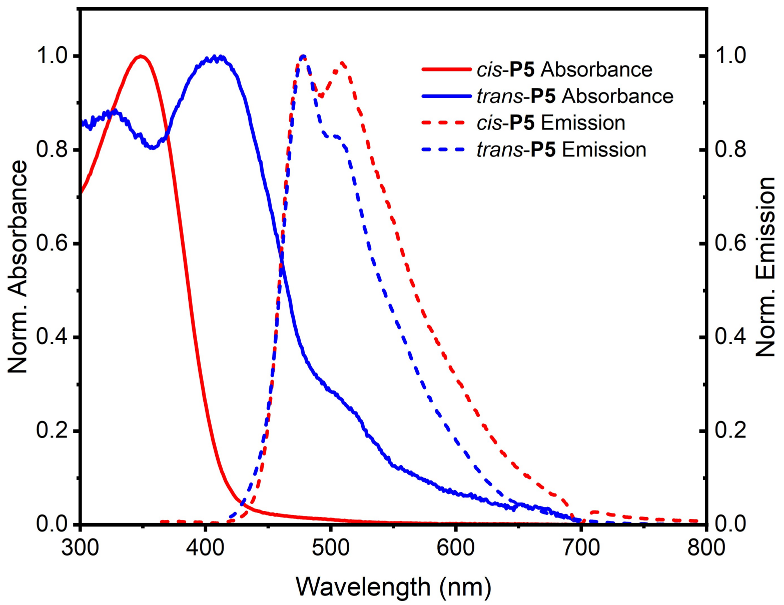

Figure S58. *cis*-**P5** (M_n^{theo} = 10.0 kg/mol, M_n^{exp} = 12.1 kg/mol, $Đ$ = 1.41) normalized absorbance (C = 10 μg/mL) and emission (λ_{ex} = 348 nm, C = 0.5 μg/mL) traces in $CHCl_3$. *trans*-**P5** (insolubility prevented SEC analysis in THF) normalized absorbance (C = 10 μg/mL) and emission (λ_{ex} = 407 nm, C = 0.5 μg/mL) traces in $CHCl_3$.

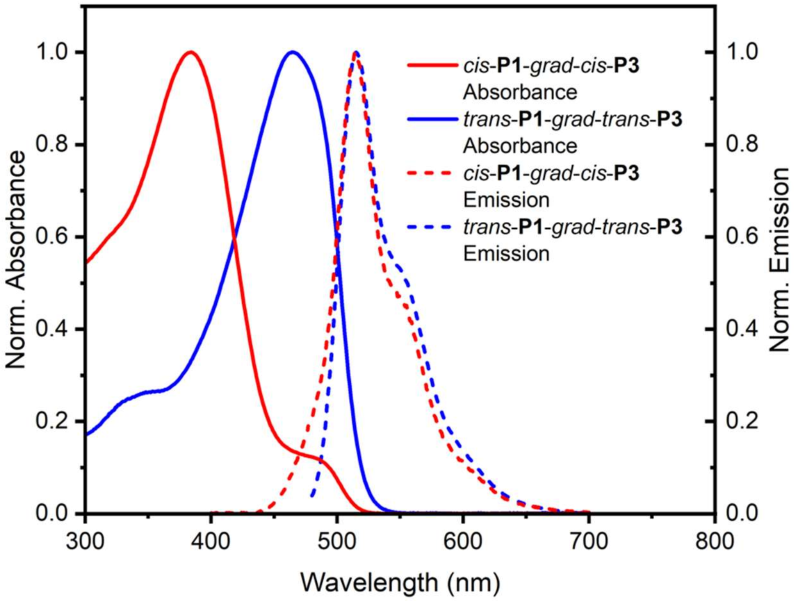

Figure S59. Gradient copolymer *cis*-**P1**-*grad*-*cis*-**P3** (M_n^{theo} = 7.9 kg/mol, M_n^{exp} = 9.2 kg/mol, $Đ$ = 1.08) normalized absorbance (C = 12.5 μg/mL) and emission excited at 384 nm (C = 1.25 μg/mL) trace in $CHCl_3$. *trans*-**P1**-*grad*-*trans*-**P3** (M_n^{theo} = 7.9 kg/mol, M_n^{exp} = 15.4 kg/mol, $Đ$ = 1.23) normalized absorbance (C = 5 μg/mL) and emission excited at 464 nm (C = 0.5 μg/mL) traces in $CHCl_3$.

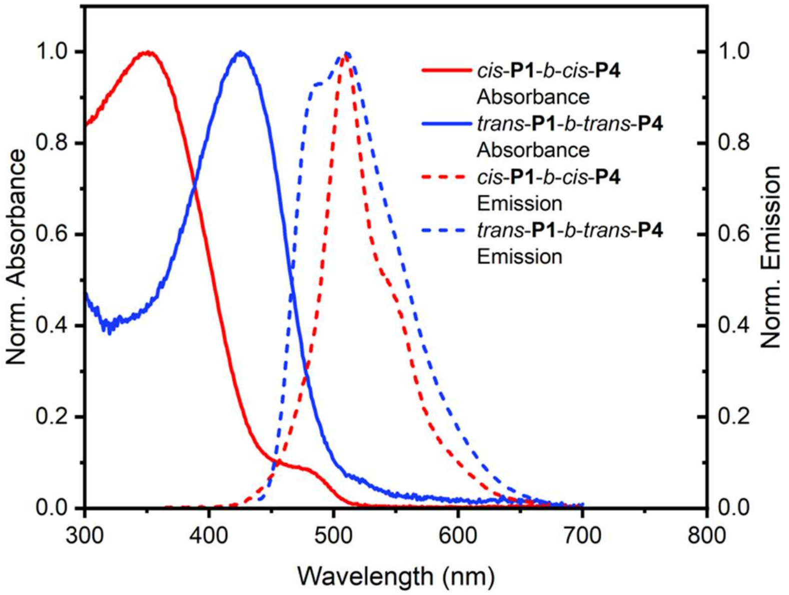

Figure S60. Block copolymer *cis*-**P1**-*b*-*cis*-**P4** (M_n^{theo} = 15.0 kg/mol, M_n^{exp} = 16.7 kg/mol, *Đ* = 1.24) normalized absorbance (C = 10 µg/mL) and emission excited at 351 nm (C = 0.1 µg/mL) traces in $CHCl_3$. *trans*-**P1**-*b*-*trans*-**P4** (insolubility prevented SEC analysis in THF) normalized absorbance (C = 10 µg/mL) and emission excited at 425 nm (C = 0.1 µg/mL) traces in $CHCl_3$.

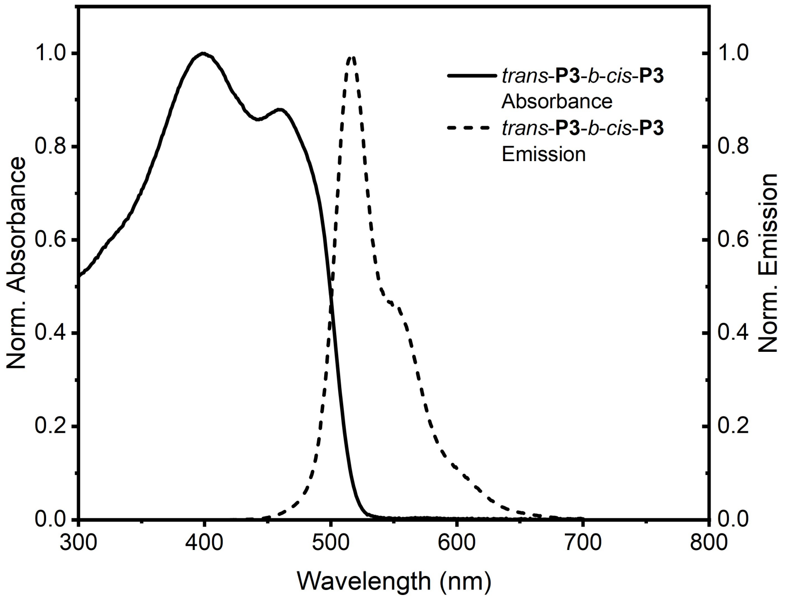

Figure S61. Stereodefined block copolymer *trans*-**P3**-*b*-*cis*-**P3** (M_n^{theo} = 9.3 kg/mol, M_n^{exp} = 11.9 kg/mol, *Đ* = 1.20) normalized absorbance (C = 5 µg/mL) and emission excited at 398 nm (C = 0.5 µg/mL) traces in $CHCl_3$.

Supporting NMR Spectra

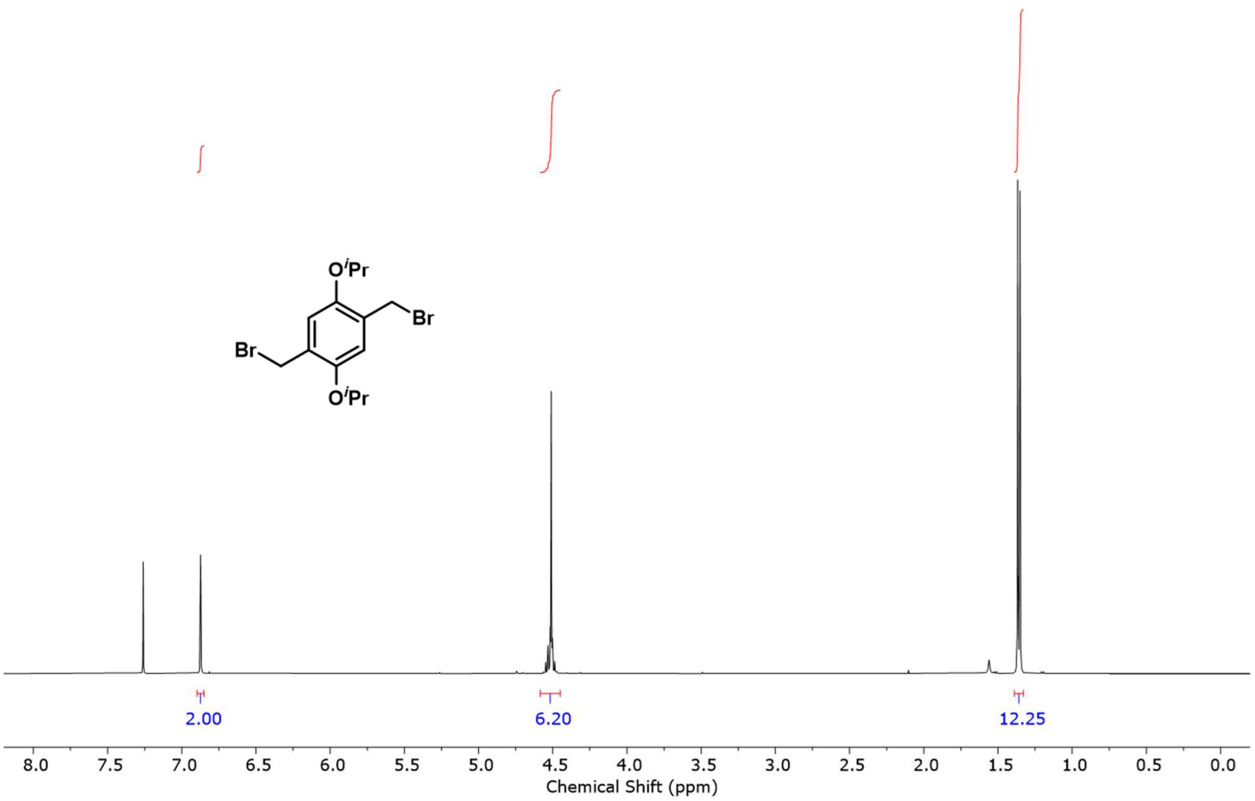

Figure S62. ^{1}H NMR (400 MHz, $CDCl_3$) spectrum of **S1b**.

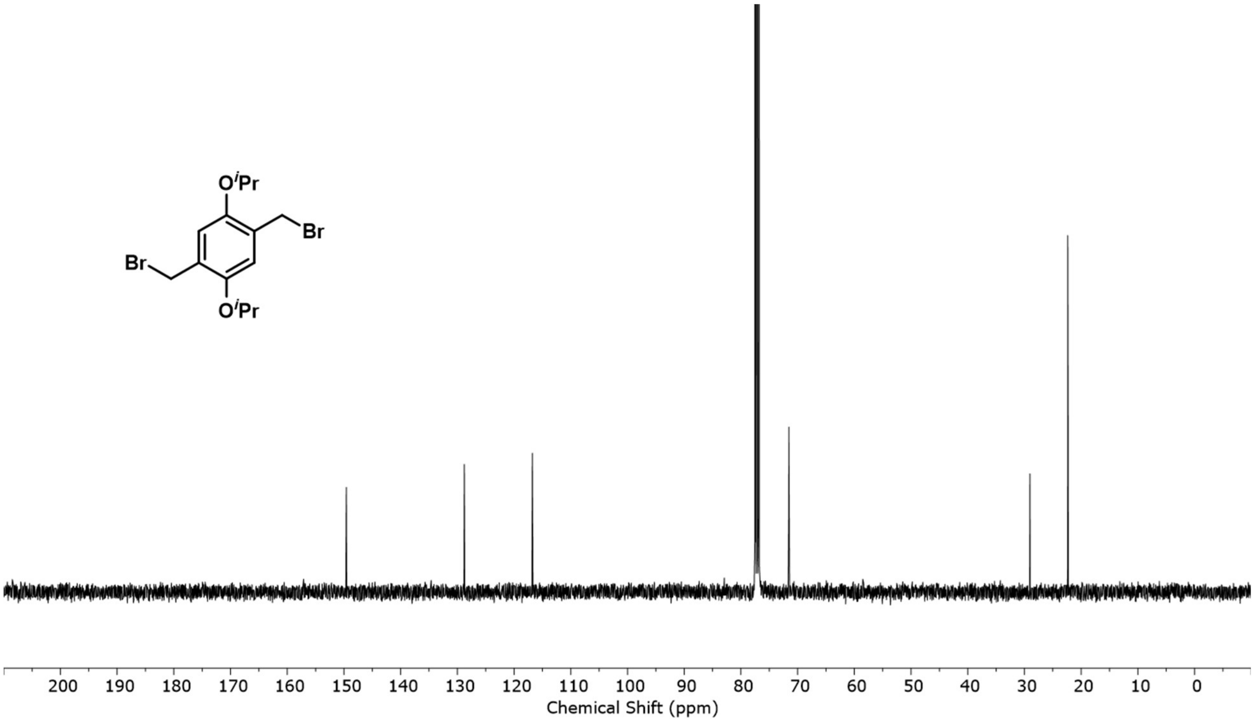

Figure S63. ^{13}C NMR (101 MHz, $CDCl_3$) spectrum of **S1b**.

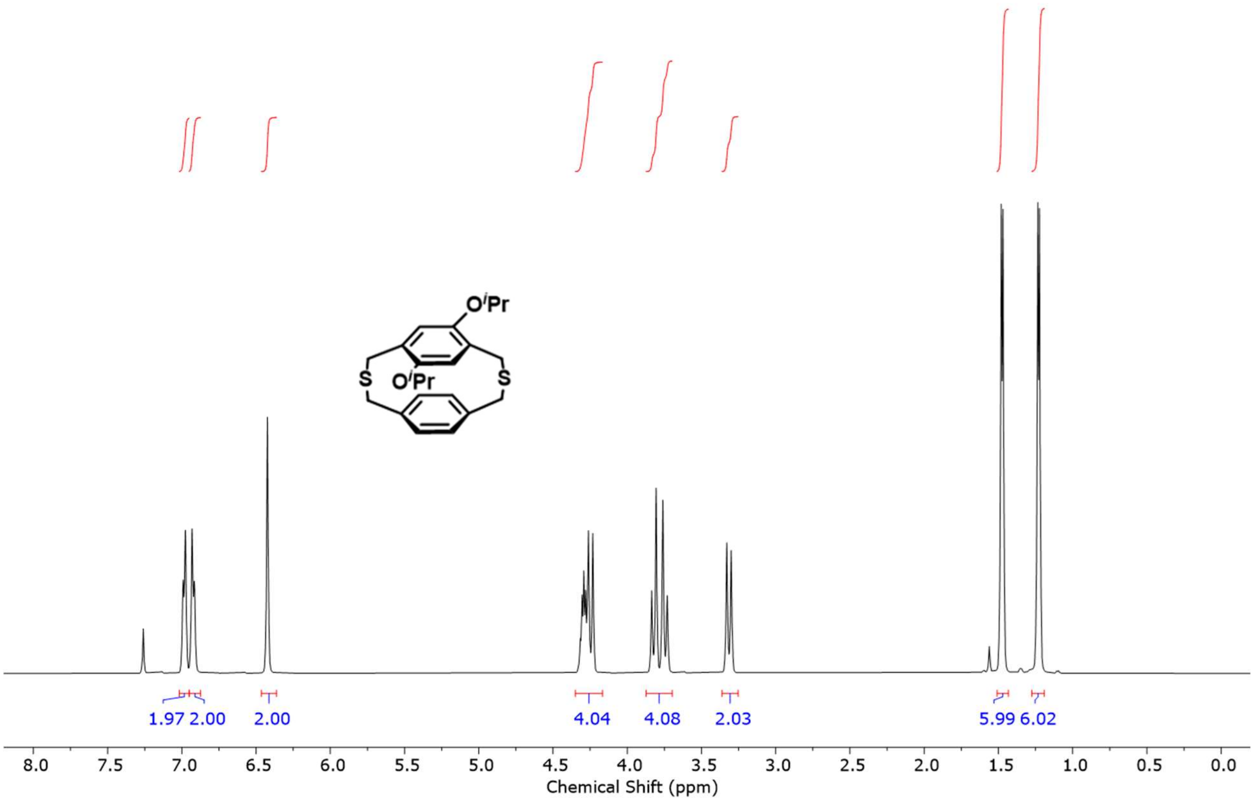

Figure S64. ^{1}H NMR (500 MHz, $CDCl_3$) spectrum of **S1d**.

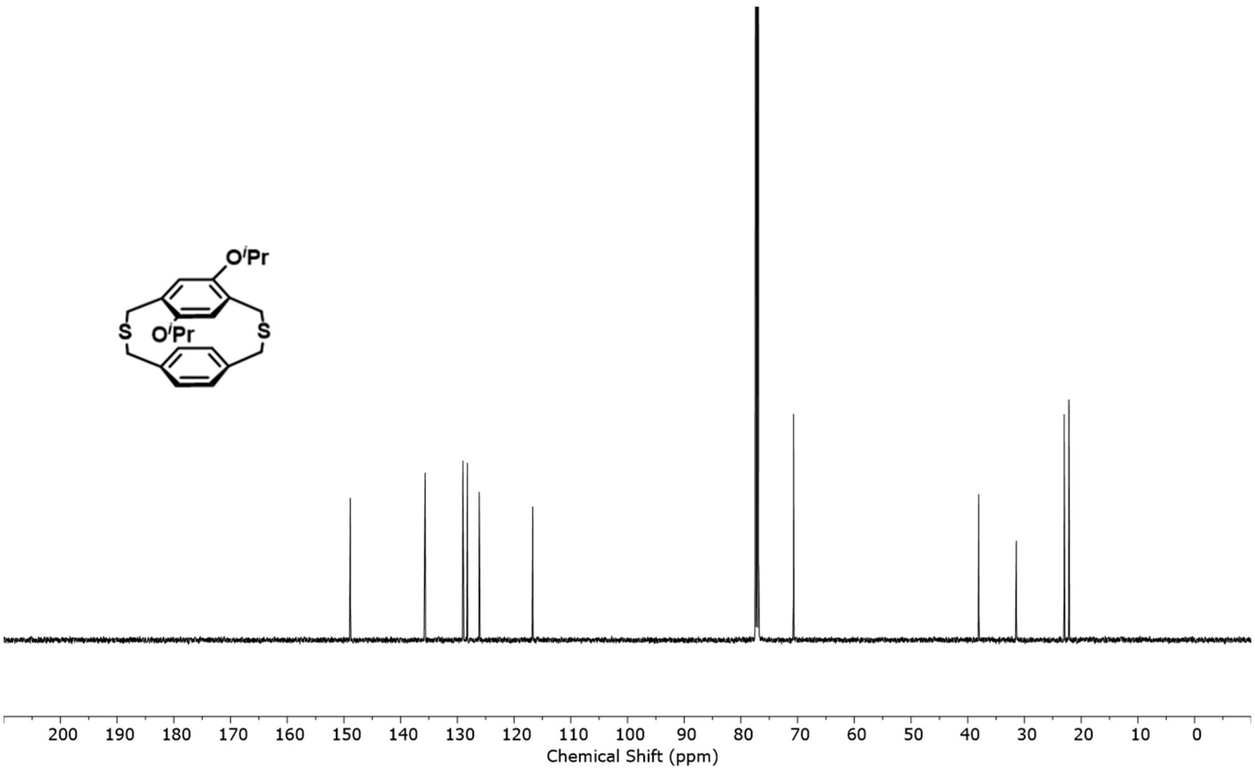

Figure S65. ^{13}C NMR (126 MHz, $CDCl_3$) spectrum of **S1d**.

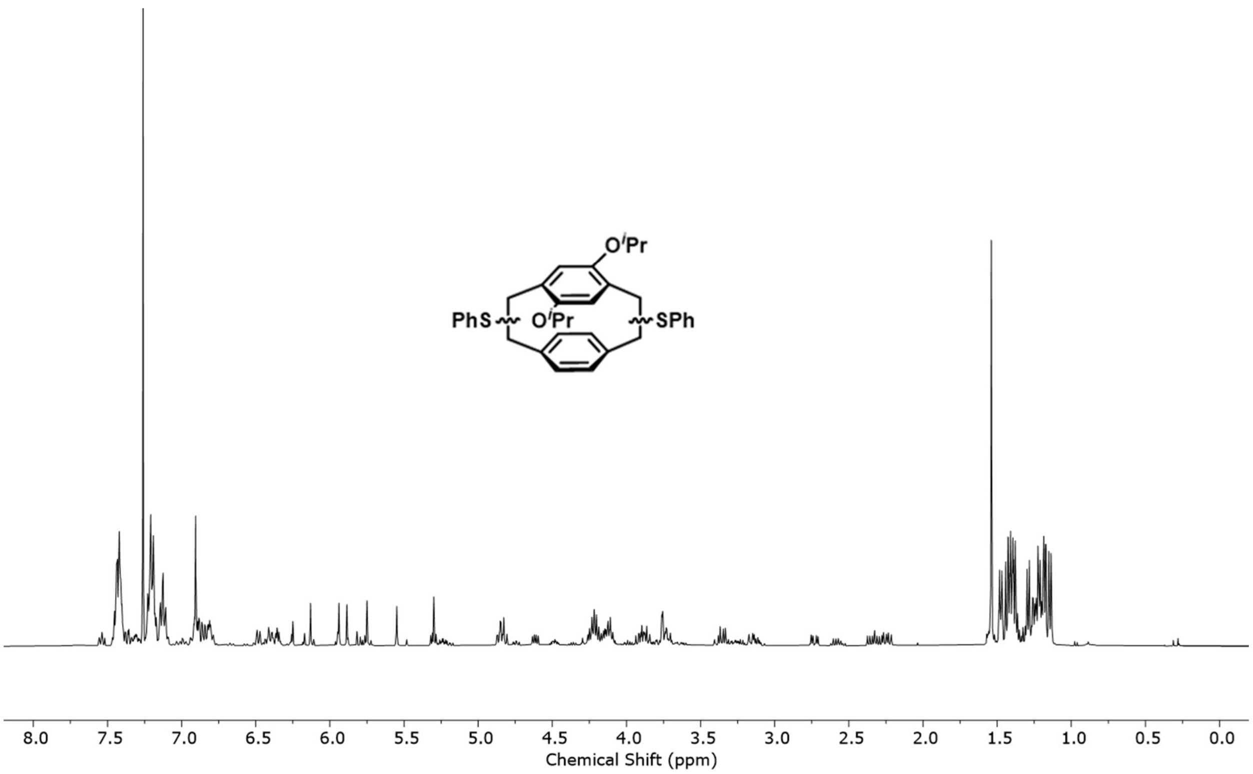

Figure S66. ^{1}H NMR (400 MHz, $CDCl_3$) spectrum of **S1f** as a mixture of stereo- and regioisomers.

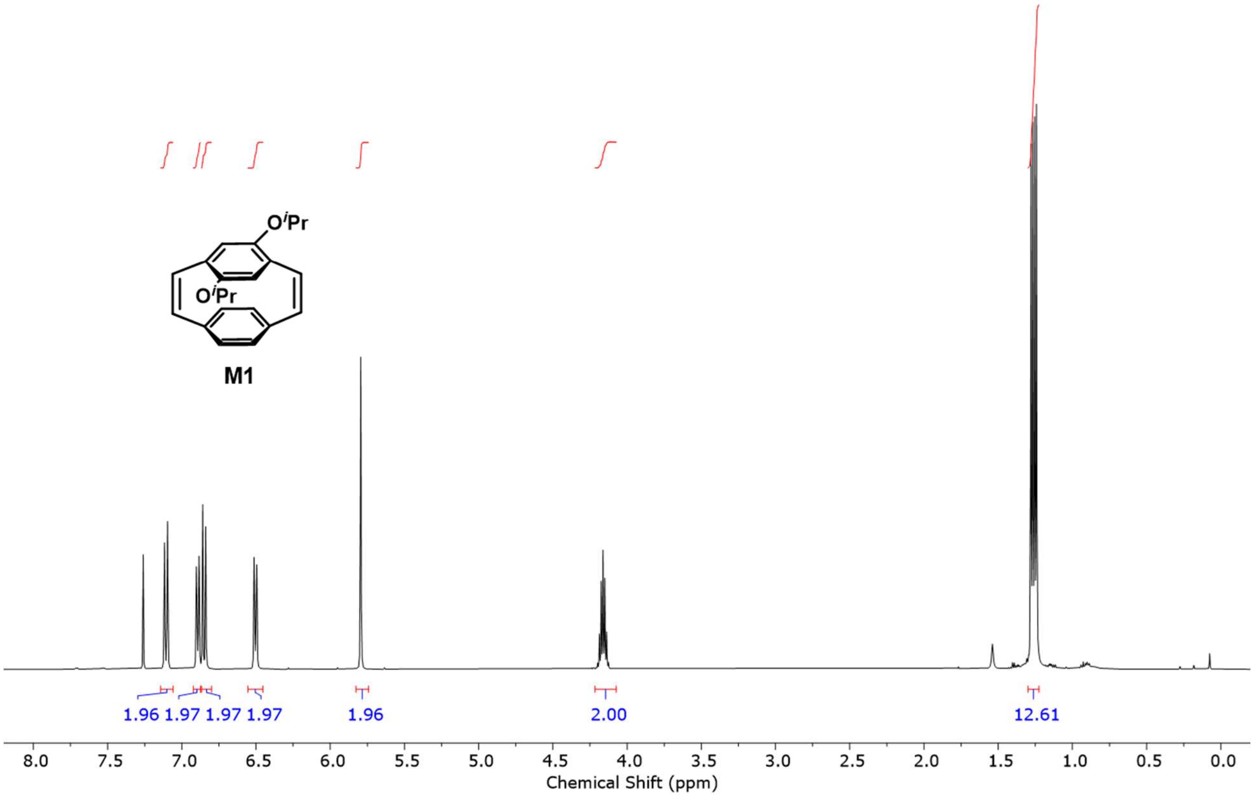

Figure S67. ^{1}H NMR (500 MHz, $CDCl_3$) spectrum of **M1**.

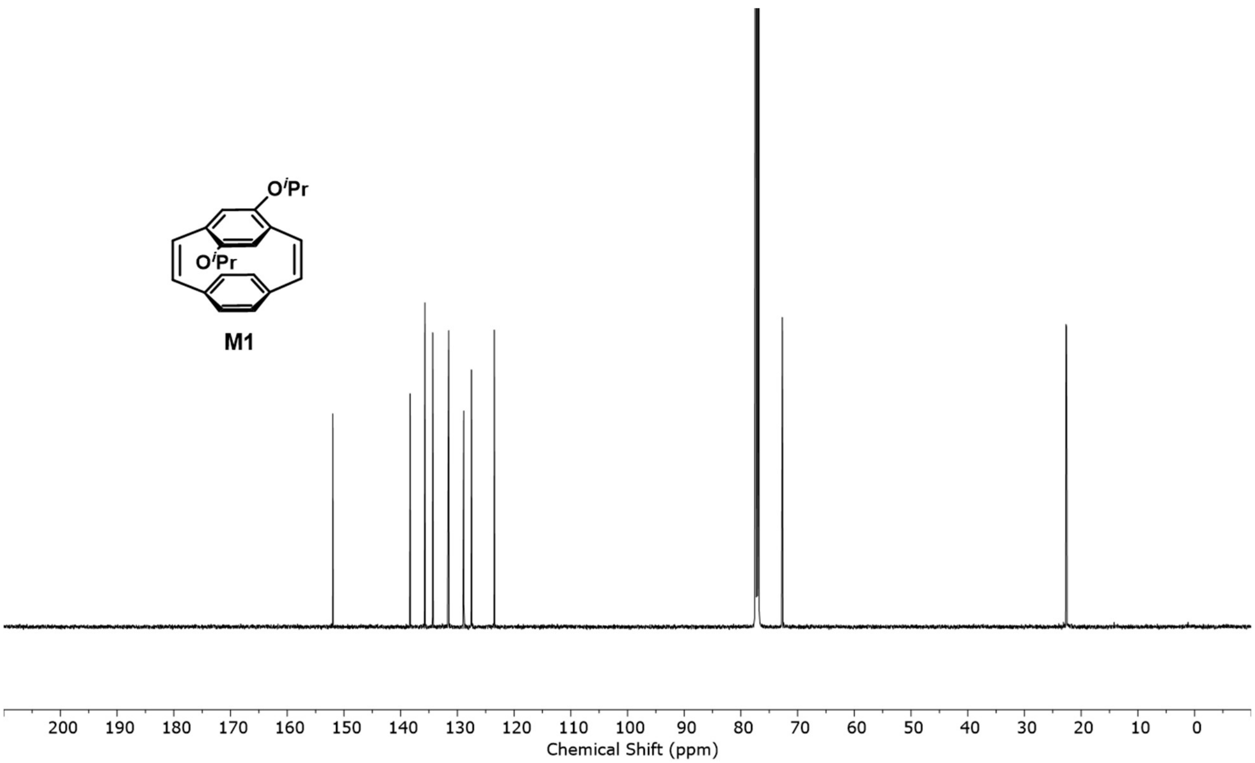

Figure S68. ^{13}C NMR (126 MHz, $CDCl_3$) spectrum of **M1**.

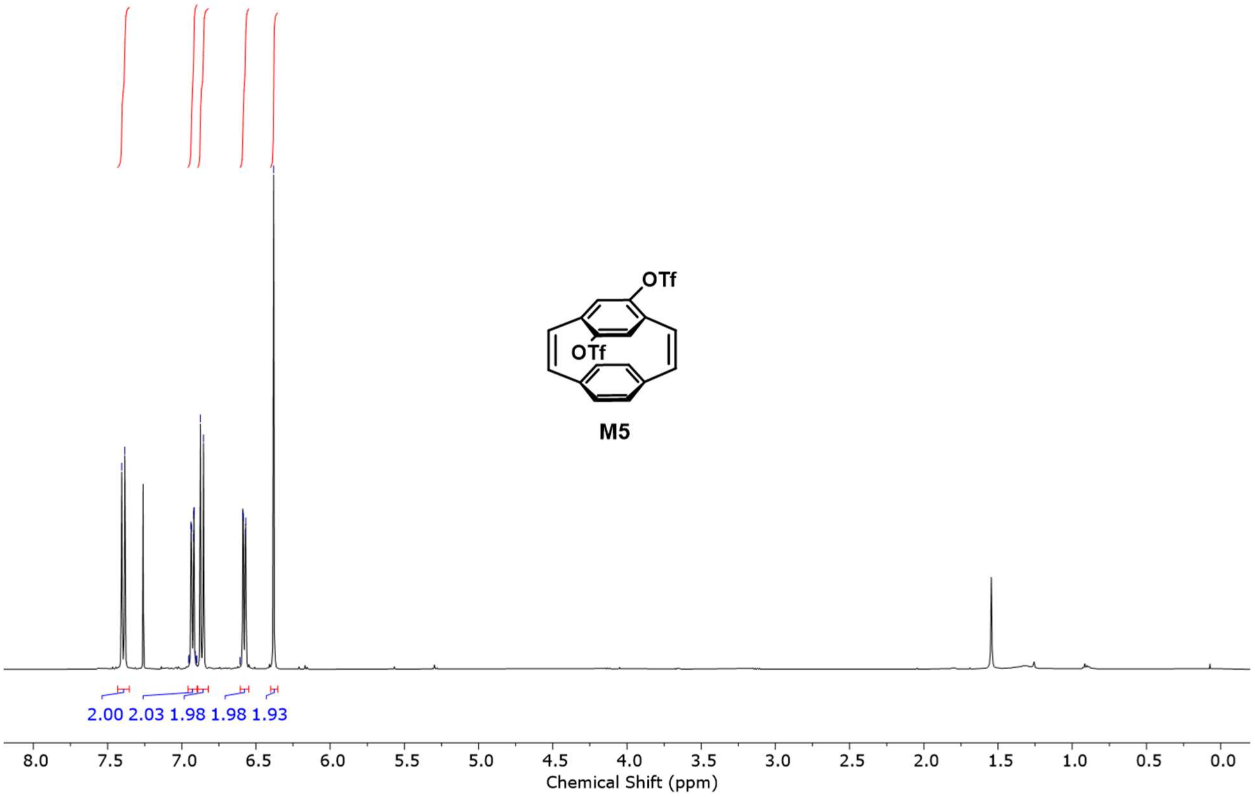

Figure S69. ^{1}H NMR (500 MHz, $CDCl_3$) spectrum of **M5**.

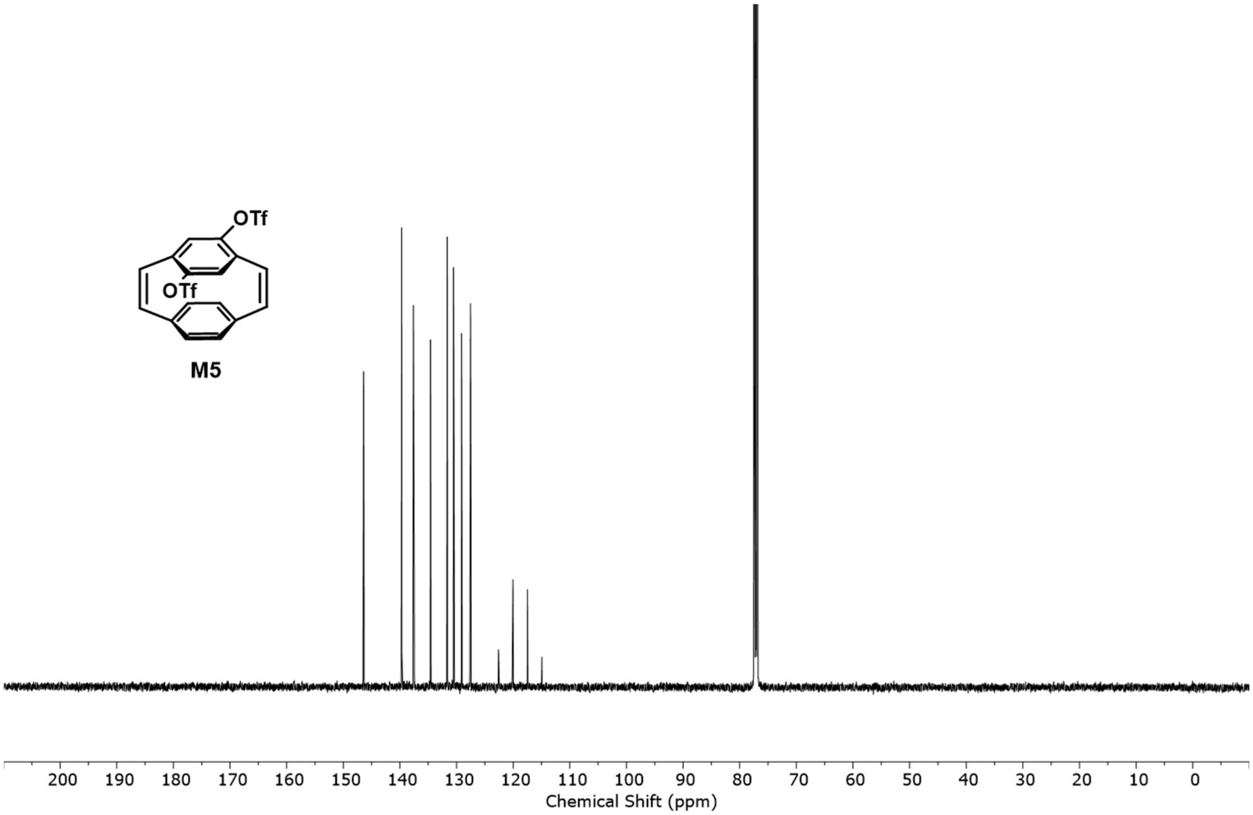

Figure S70. ^{13}C NMR (126 MHz, $CDCl_3$) spectrum of **M5**.

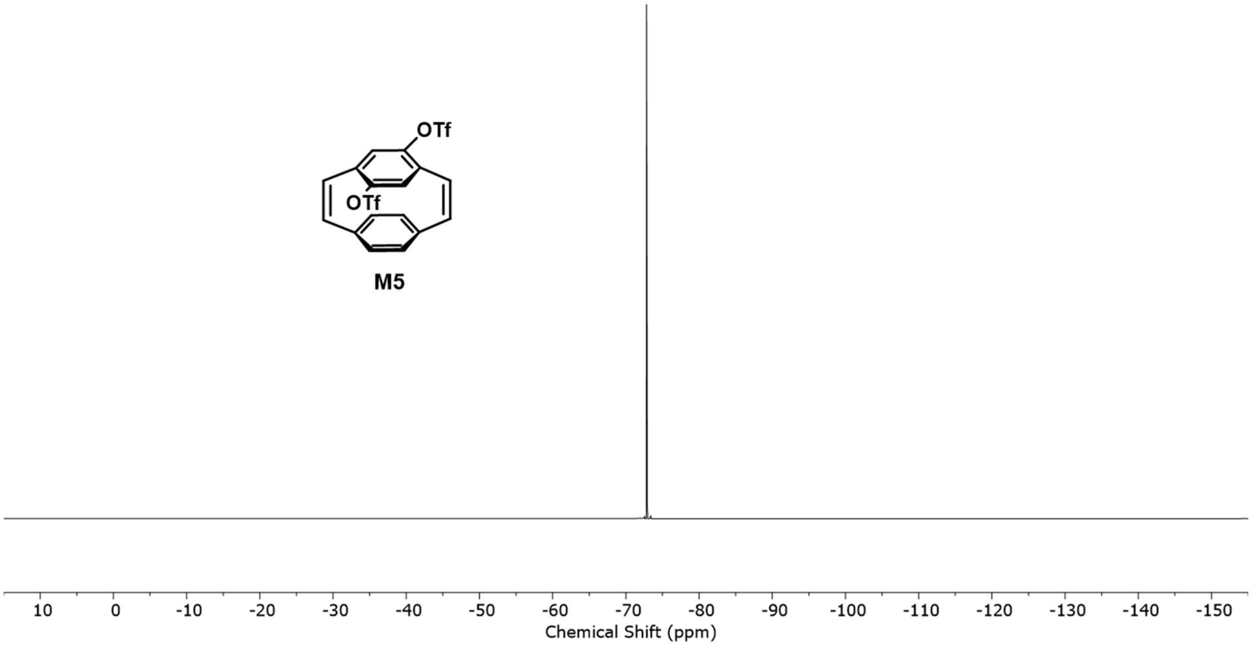

Figure S71. ^{19}F NMR (376 MHz, $CDCl_3$) spectrum of **M5**.

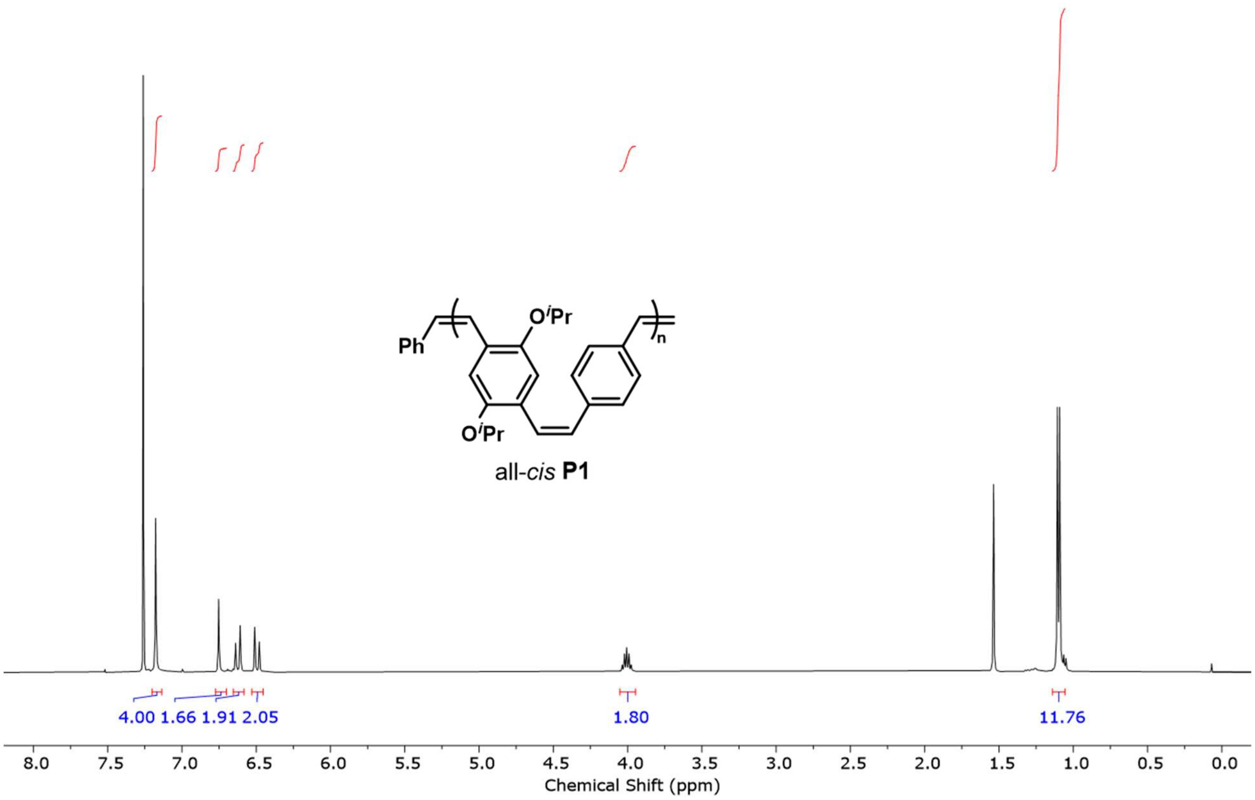

Figure S72. ^{1}H NMR (500 MHz, $CDCl_3$) spectrum of all-*cis* **P1**.

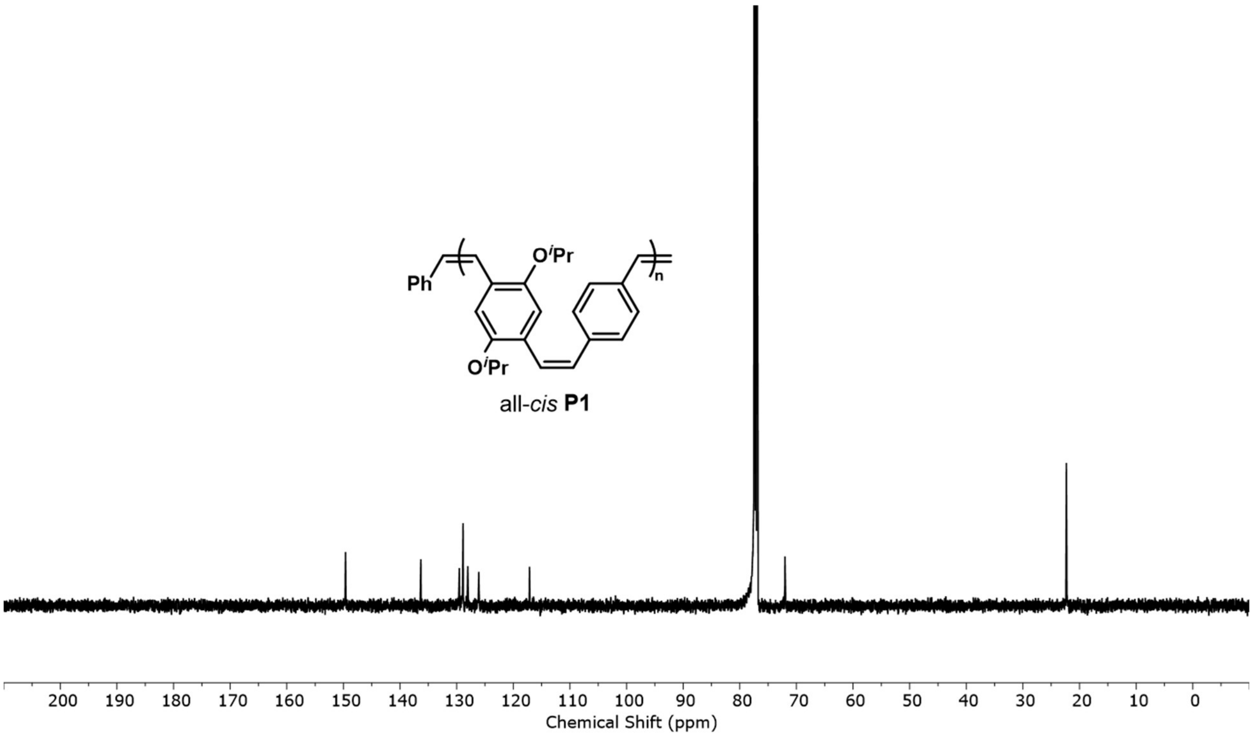

Figure S73. ^{13}C NMR (126 MHz, $CDCl_3$) spectrum of all-*cis* **P1**.

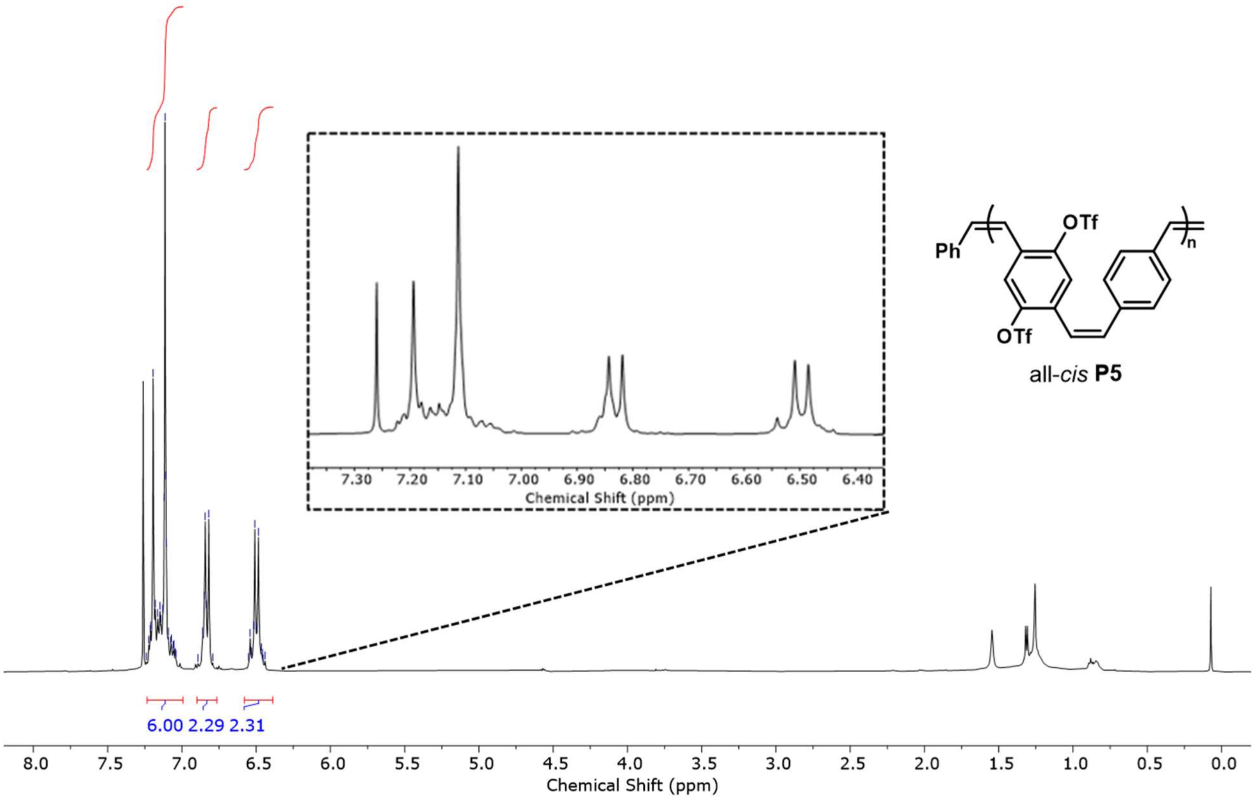

Figure S74. ^{1}H NMR (500 MHz, $CDCl_3$) spectrum of all-*cis* **P5**. Inset shows minor ^{1}H NMR peaks indicating lack of full regioselectivity.

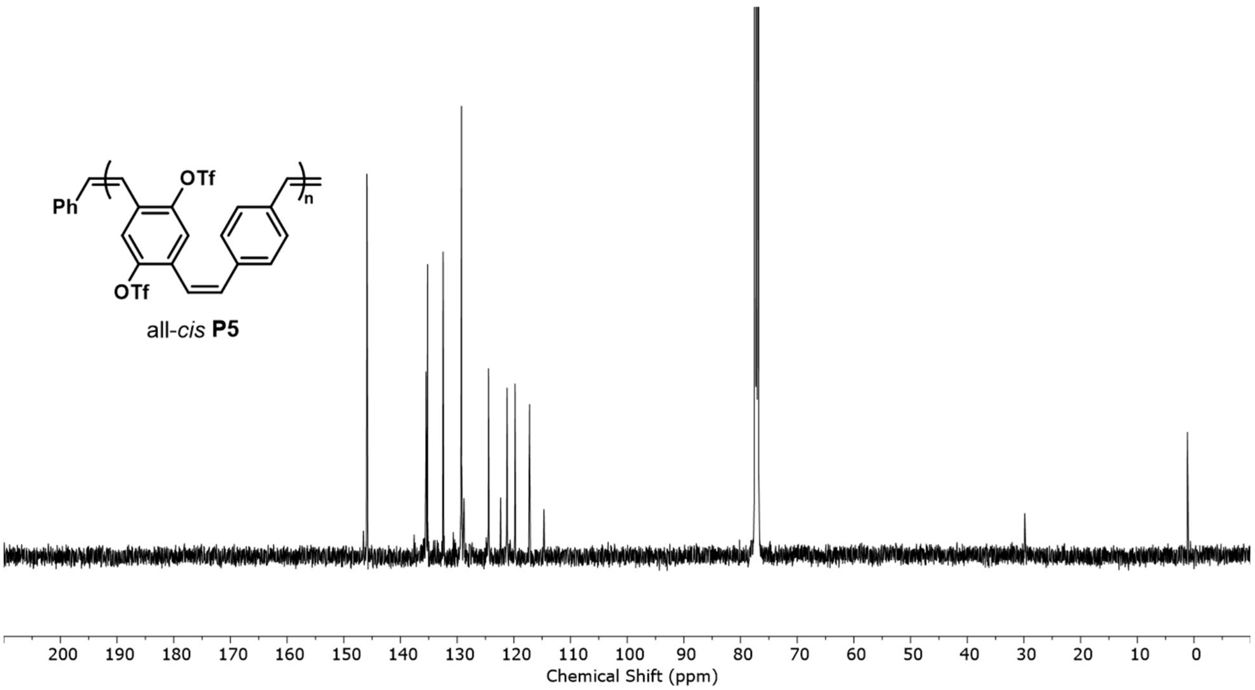

Figure S75. ^{13}C NMR (126 MHz, $CDCl_3$) spectrum of all-*cis* **P5**. Minor peaks in the aromatic region suggest a lack of full regioselectivity.

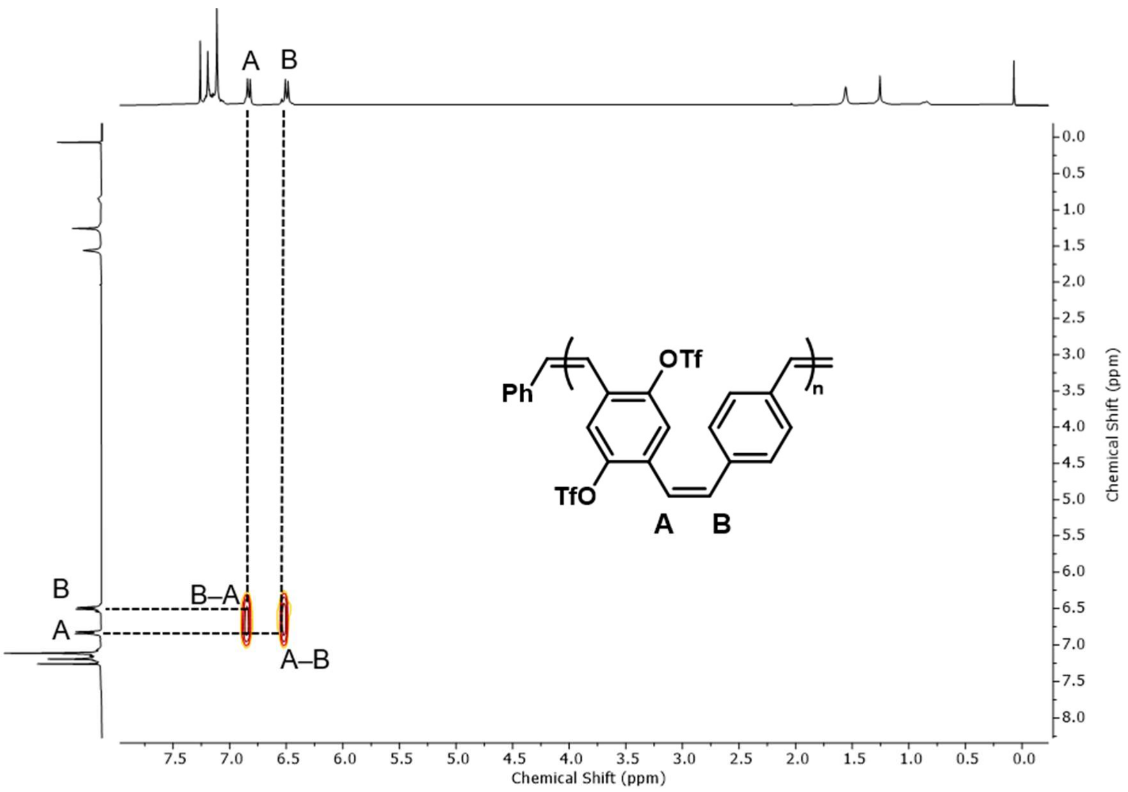

Figure S76. COSY spectrum of all-*cis* **P5**.

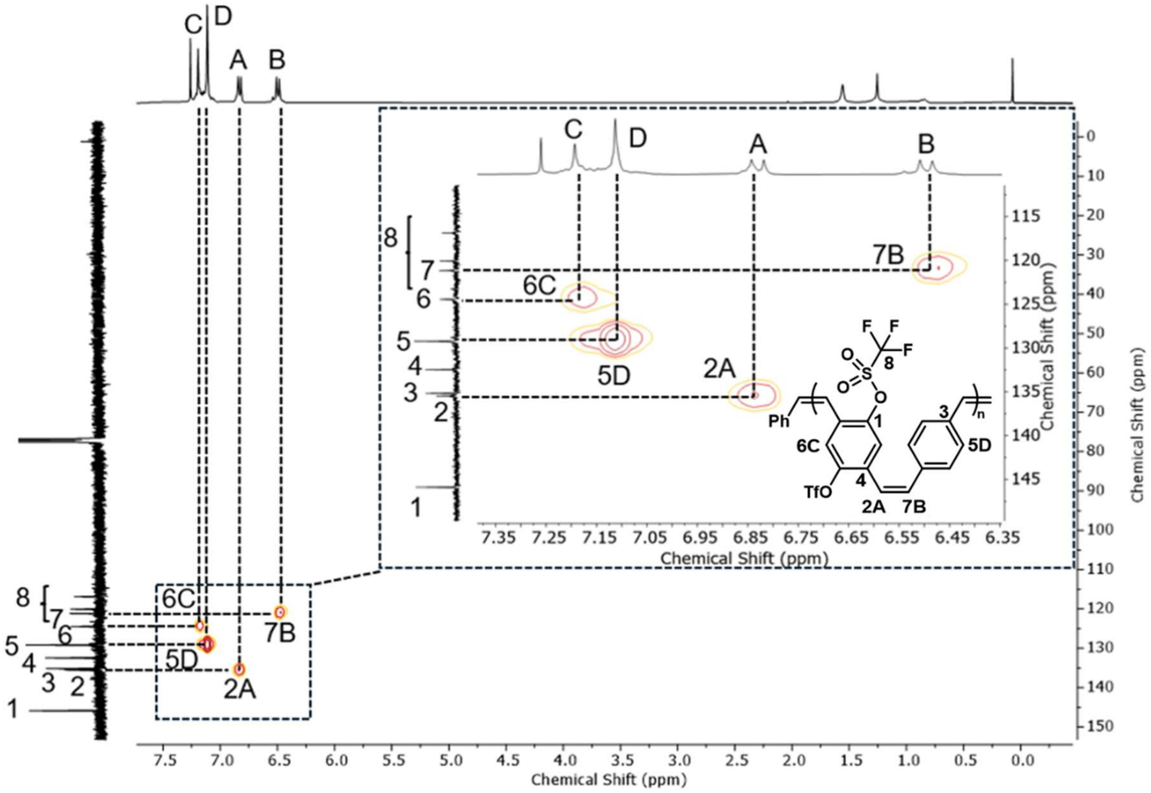

Figure S77. HSQC spectrum of all-*cis* **P5**. Inset shows zoomed in correlation peaks for clarity.

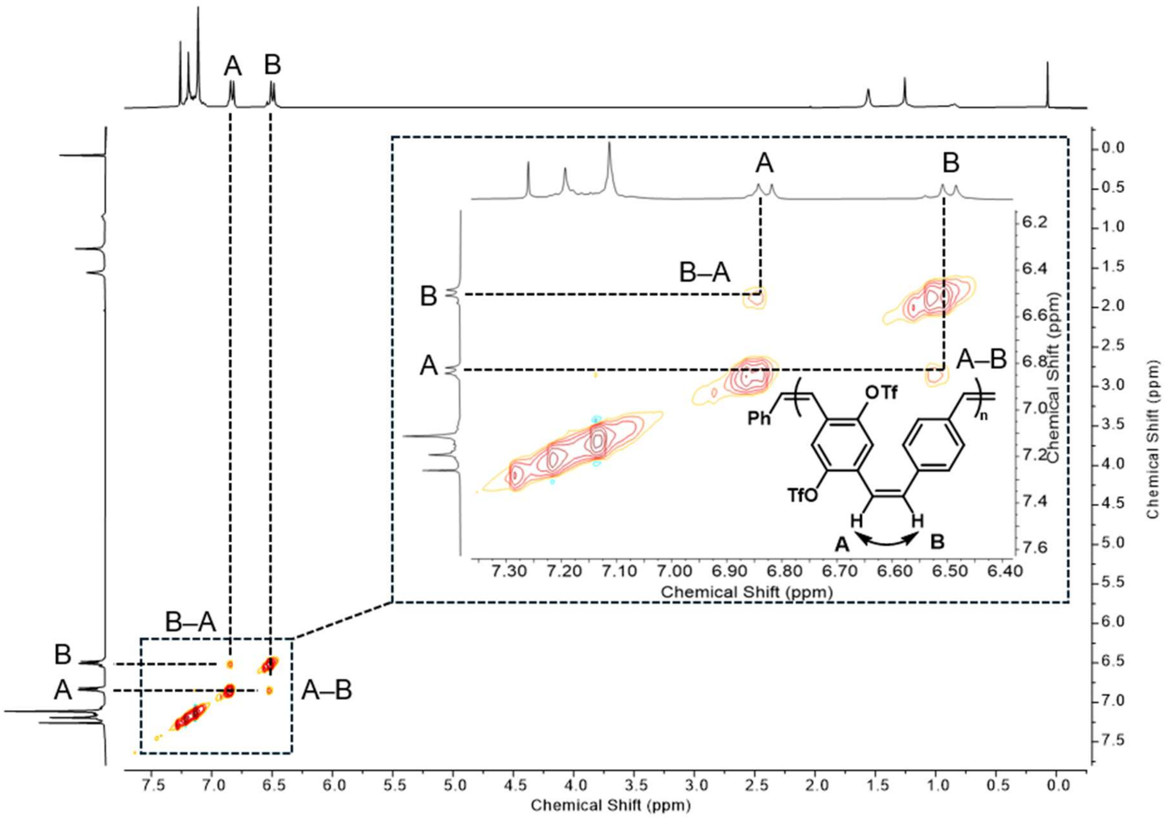

Figure S78. NOESY spectrum of all-*cis* **P5**. Inset shows zoomed in correlation peaks for clarity.

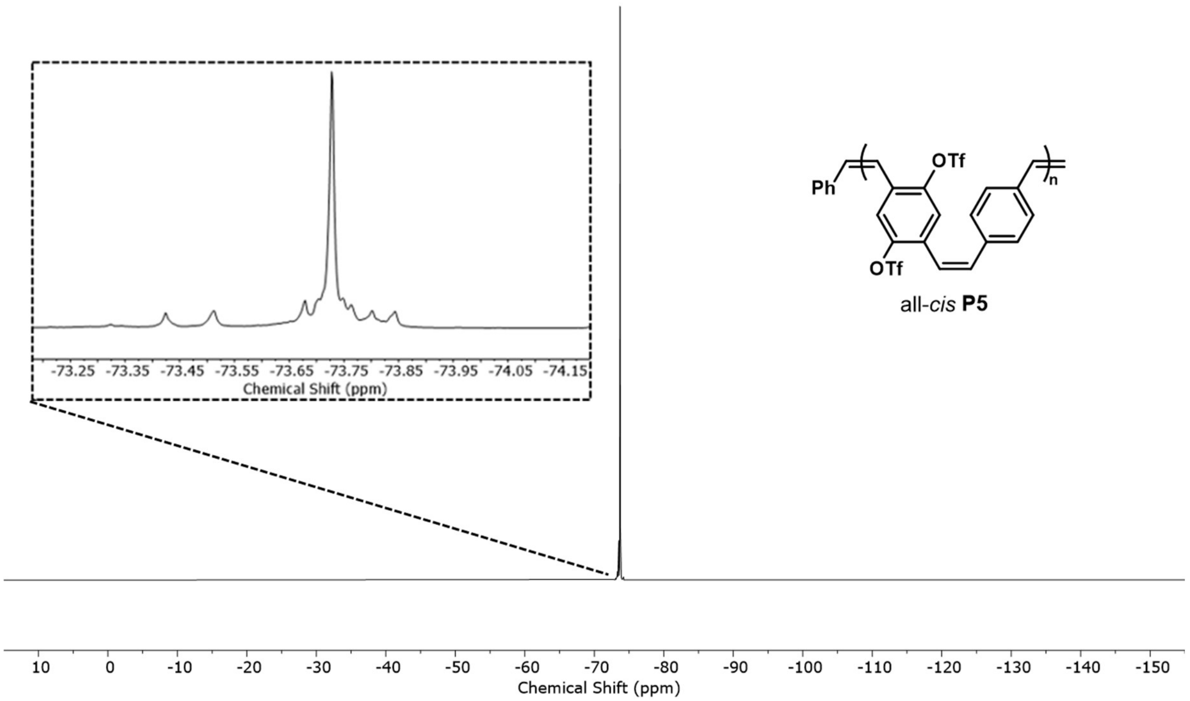

Figure S79. ^{19}F NMR (470 MHz, $CDCl_3$) spectrum of all-*cis* **P5**. Inset shows minor ^{19}F NMR peaks indicating lack of full regioselectivity.

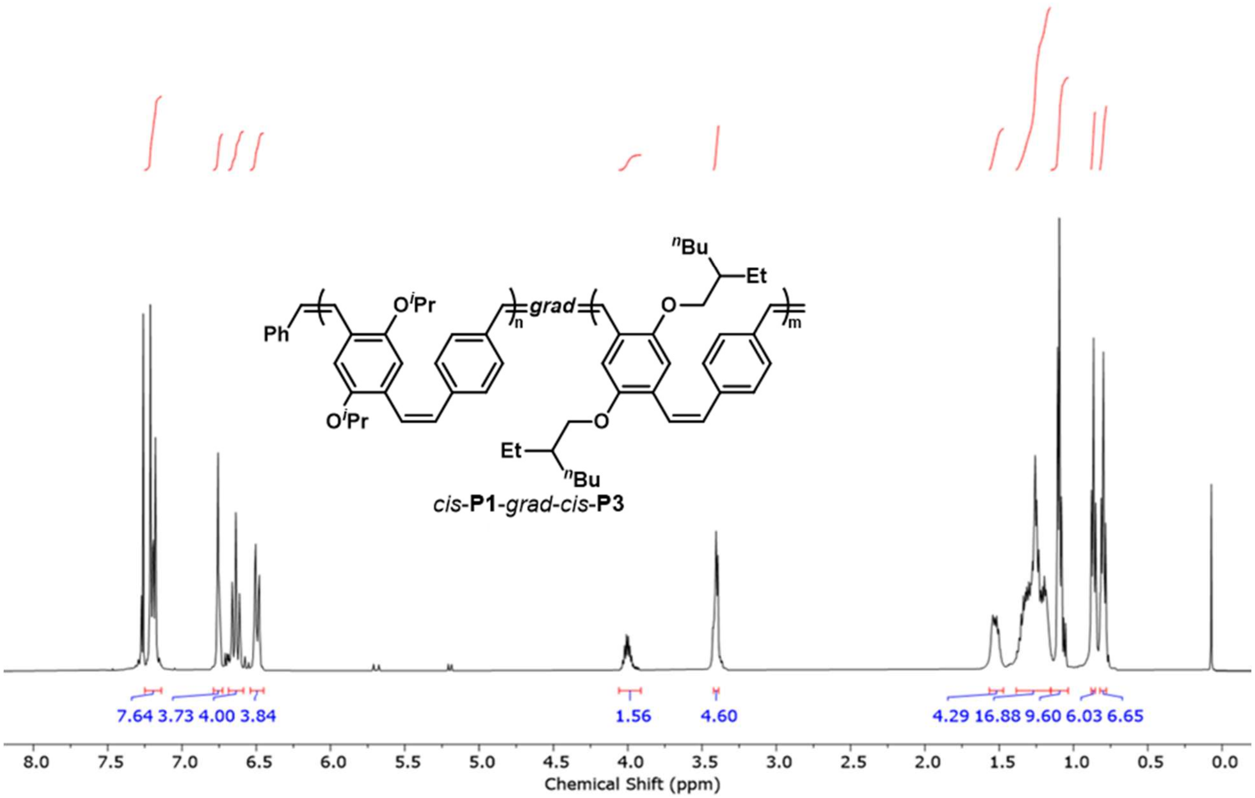

Figure S80. ^{1}H NMR (500 MHz, $CDCl_3$) spectrum of *cis*-**P1**-*grad*-*cis*-**P3**.

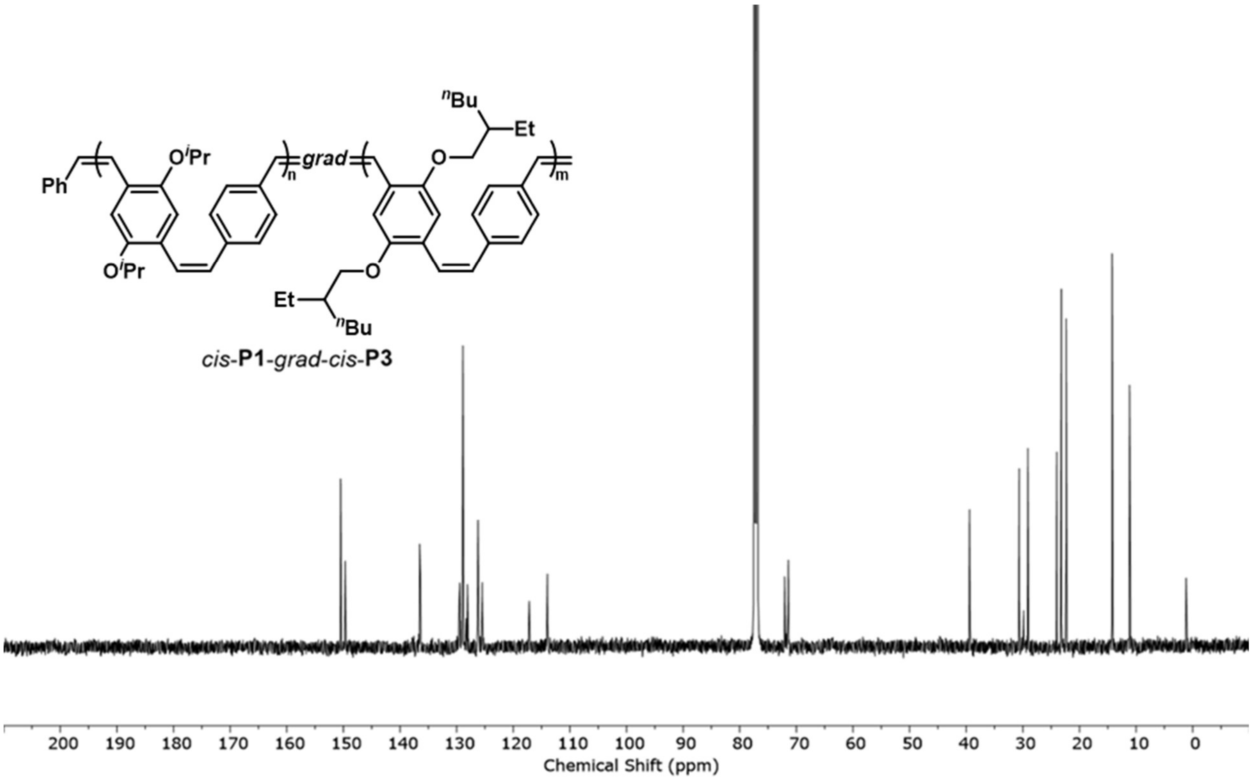

Figure S81. ^{13}C NMR (126 MHz, $CDCl_3$) spectrum of *cis*-**P1**-*grad*-*cis*-**P3**.

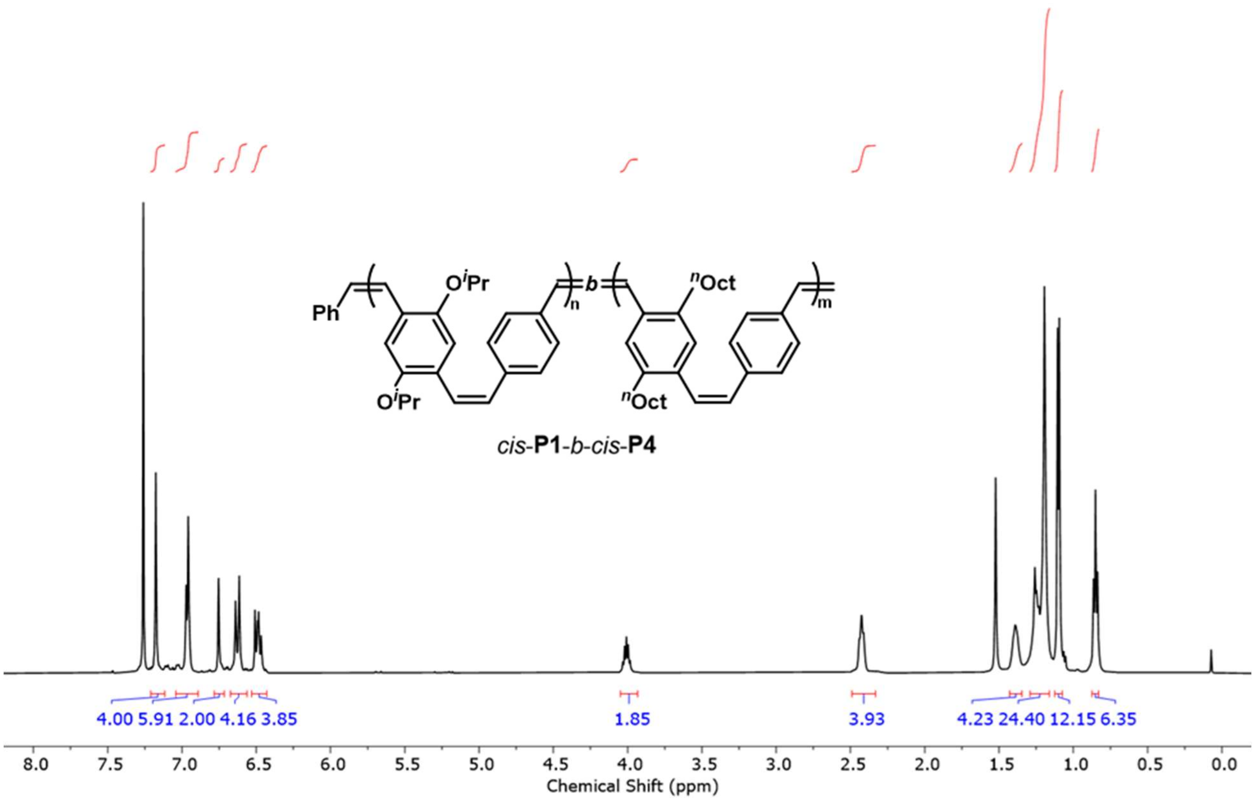

Figure S82. ^{1}H NMR (500 MHz, $CDCl_3$) spectrum of *cis*-**P1**-*b*-*cis*-**P4**.

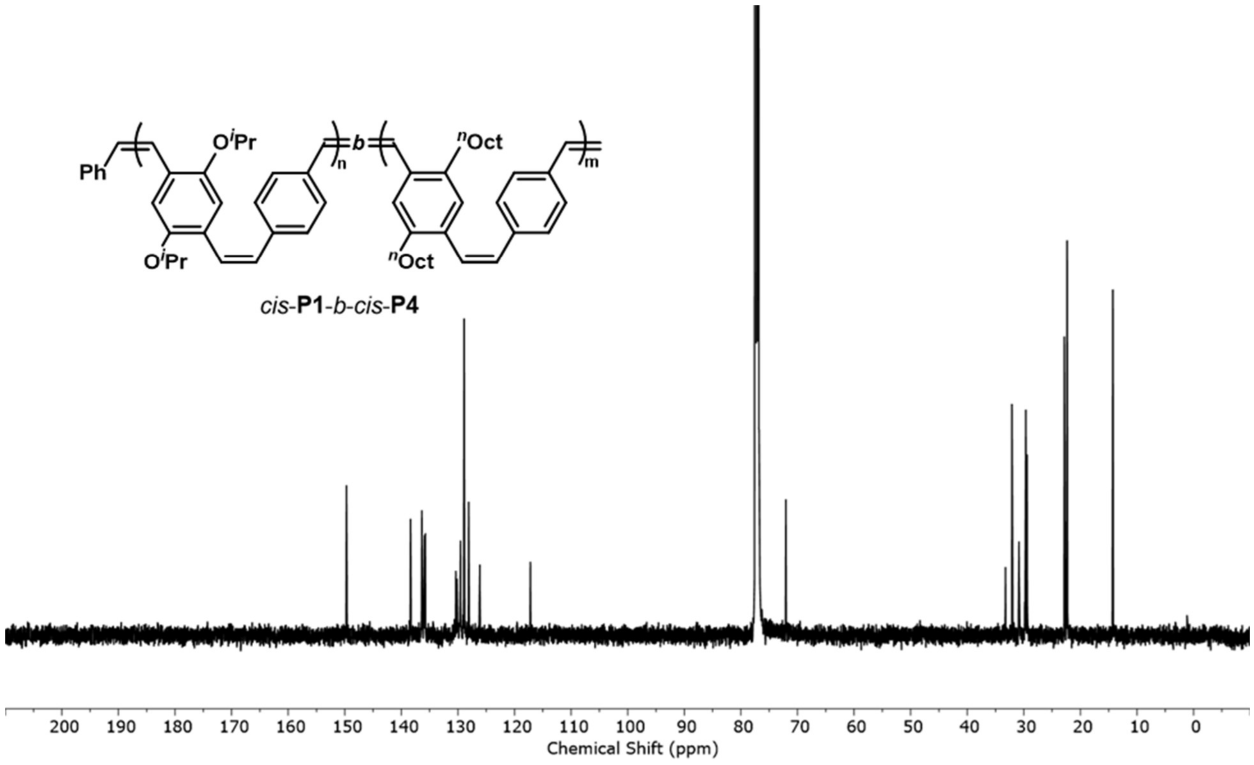

Figure S83. ^{13}C NMR (126 MHz, $CDCl_3$) spectrum of *cis*-**P1**-*b*-*cis*-**P4**.

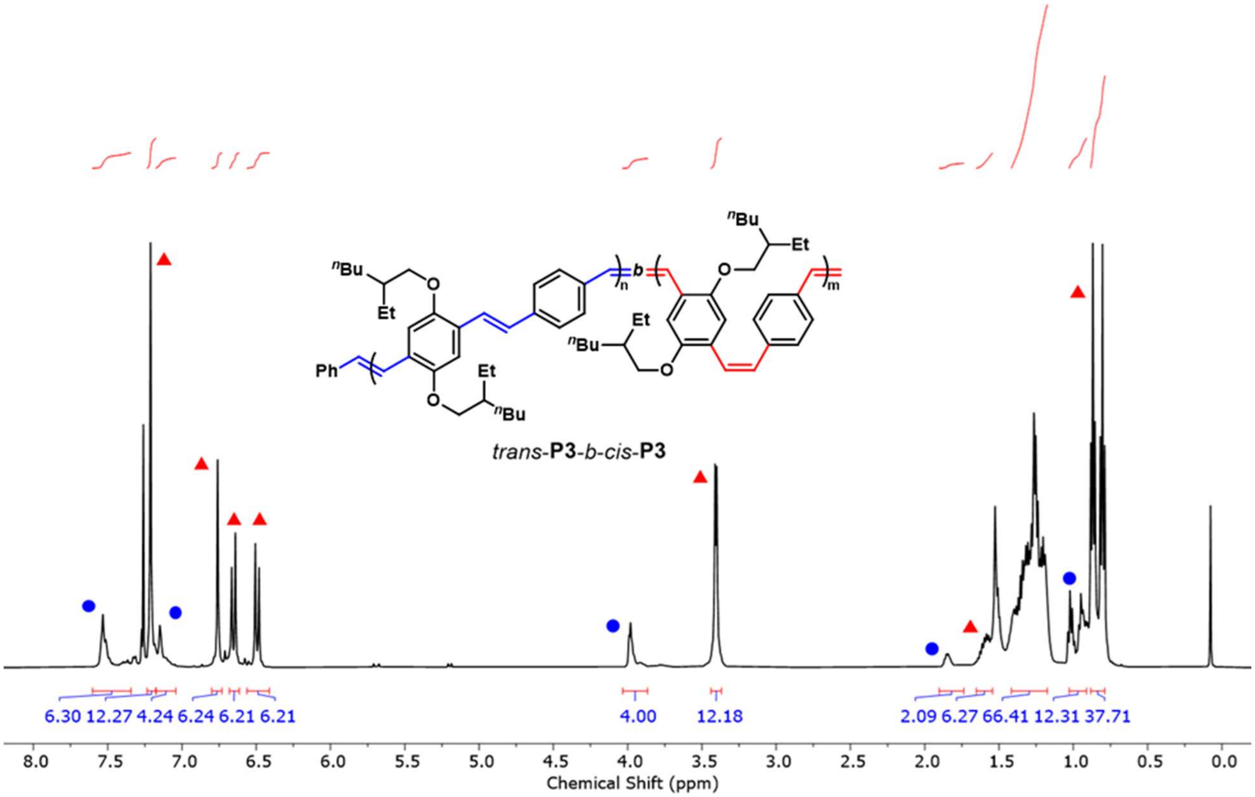

Figure S84. ^{1}H NMR (500 MHz, $CDCl_3$) spectrum of *trans*-**P3**-*b*-*cis*-**P3**.

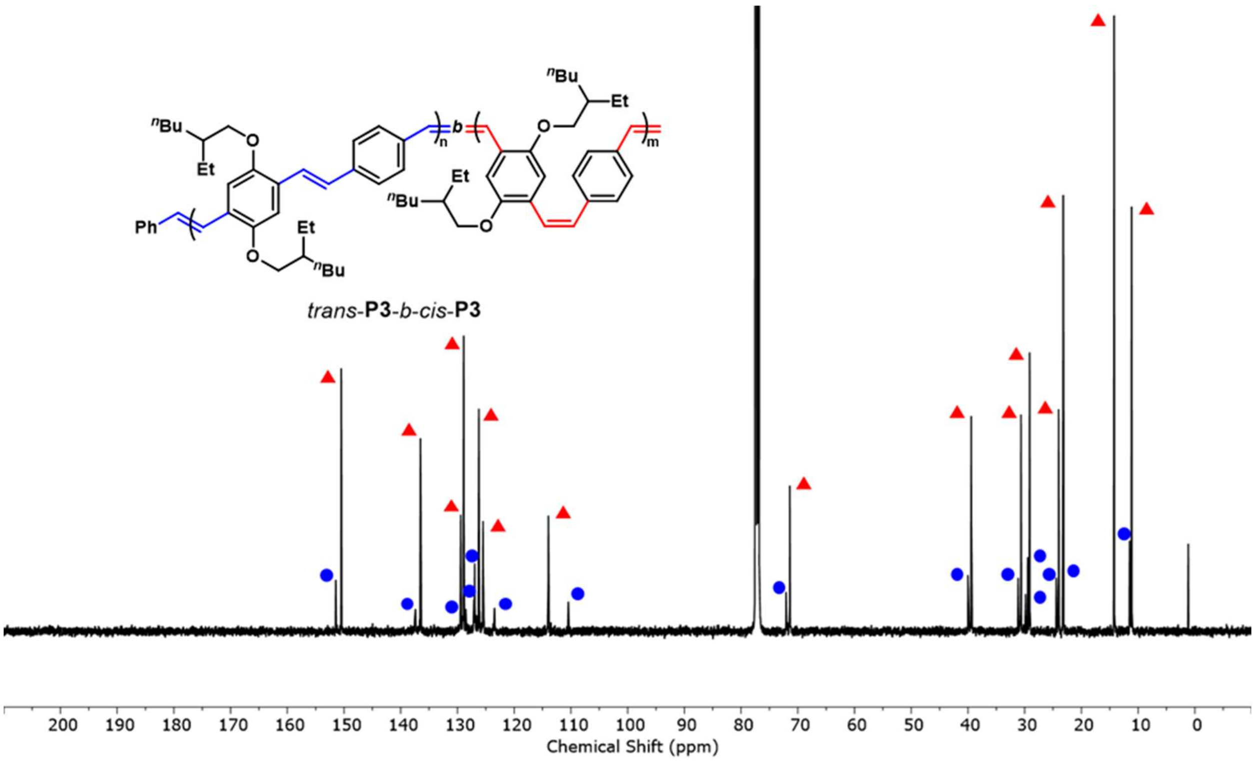

Figure S85. ^{13}C NMR (126 MHz, $CDCl_3$) spectrum of *trans*-**P3**-*b*-*cis*-**P3**.

Computational Details

All geometry optimizations of intermediates and transition states were achieved using B3LYP[6]-D3[7]/def2svp[8]-SDD(Ru)[9] method as implemented in Gaussian16.[10] Moreover, the solvation effects were considered with benzene as the solvent used experimentally using the CPCM solvent model.[11] Frequency calculations were also conducted at the same level of theory to obtain vibrational frequencies to determine the identity of stationary points as intermediates (no imaginary frequencies) or transition states (only one imaginary frequency), as well as obtain thermal correction to enthalpy and free energy at 298 K. Intrinsic Reaction Coordinate (IRC) calculations were done on the transition states to verify the correct transition state associated with the reaction. The endpoint geometries obtained from the IRC calculations were further optimized to verify the authenticity of the transition state. An extensive manual conformational search was conducted for all transition states relevant to selectivity using **M**-OMe as a truncated monomer model in combination with **Ru-1a** (Figure S92). Only the lowest-energy transition states were retained and used to generate the corresponding transition states (**TS-**α and **TS-**β). Intermediates were selected from the corresponding lowest-energy transition states via IRC calculations. Finally, we performed single-point calculations on the optimized geometries using the more robust M06L[12]-D3/def2-TZVPP[8]-SDD(Ru) using the SMD solvent model[13] method to improve our numerical precision. Therefore, the final reported energy was calculated at the M06L-D3/def2tzvpp-SDD(Ru)-SMD(benzene)//B3LYP-D3/def2svp-SDD(Ru)-CPCM(benzene) level of theory.

All structural figures were generated with CYLview.[14] Distances in structural figures are shown in Å and energies are in kcal/mol. Noncovalent interaction (NCI) analysis, also known as reduced density gradient (RDG) method, was performed on Multiwfn to study the possible effect of noncovalent interaction in the relevant transition states.[15] Extension distance of 0 Bohr, medium quality grid (totally about 512000 points) were set by default. Further visualization of the color-filled RDG isosurface was realized by VMD, where RDG isosurface and color range were set as 0.5, and -0.035 to 0.2, respectively.[16]

To further investigate the striking selectivity dictated by **Ru-1a**, a series of dispersion-corrected DFT calculations were conducted using the unsubstituted PCD (**M0**) as a model monomer to reduce the computational cost (Figure S85).[17]

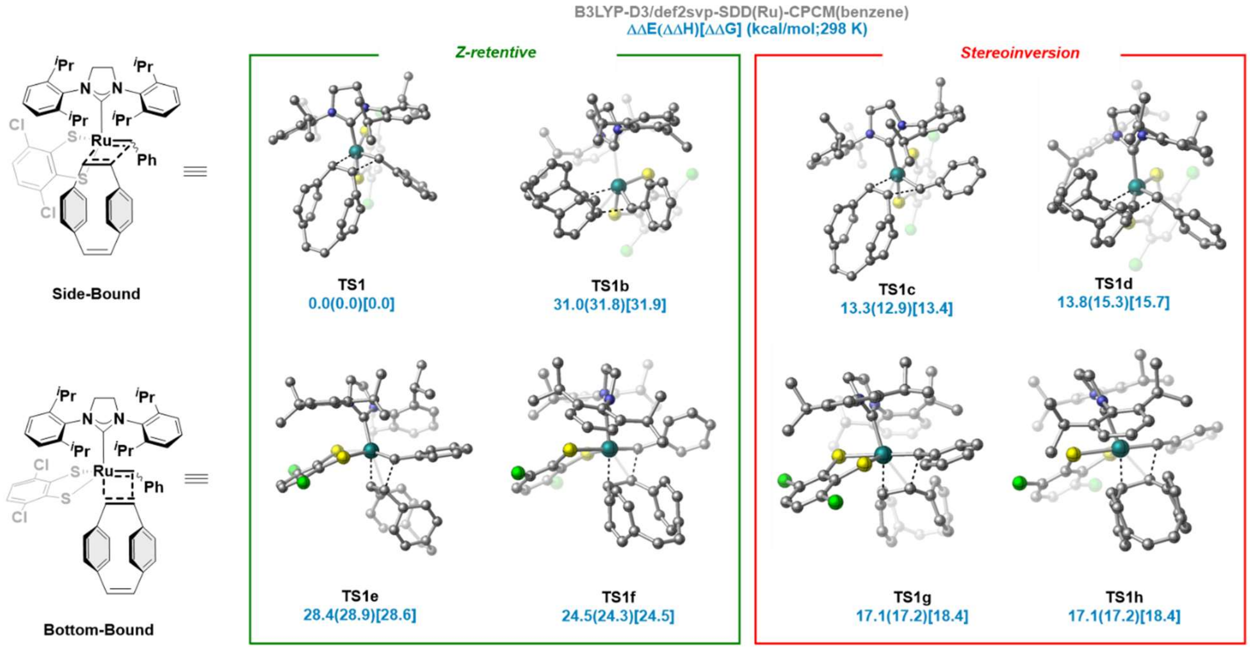

Figure S86. Possible transition states for reactions of **M0** with **Ru-1a**.

The stereoretention leading to the ruthenacycle with substituents pointing away from the NHC is favored by ~13.3 kcal/mol relative to stereoinversion due to the direct steric clash of the benzylidene with the aryl substituents on the NHC, rationalizing the exclusive formation of all-*cis* PPV observed (Figure S86)**.**

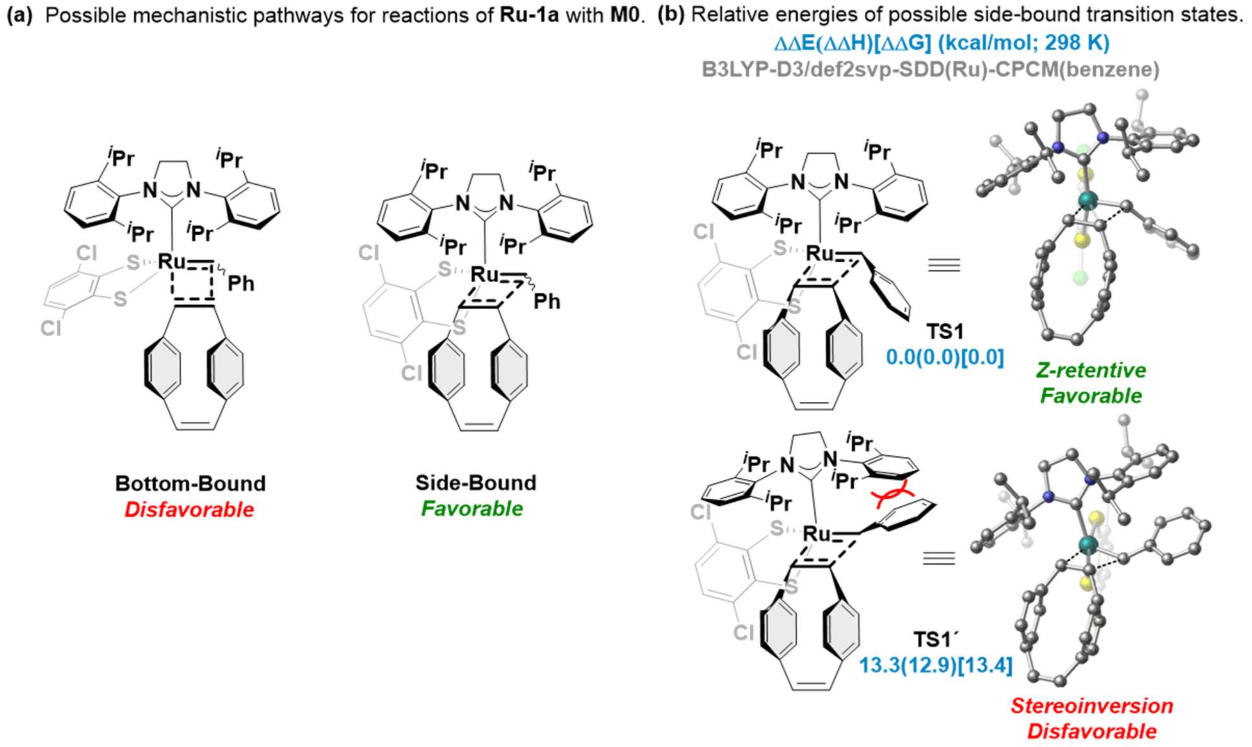

Figure S87. (a) Summary of bottom-bound vs. side-bound pathways polymerizing **M0** with **Ru-1a**. (b) Comparison of *Z*-retentive (TS1) and stereoinversion (TS1') transition state energies.

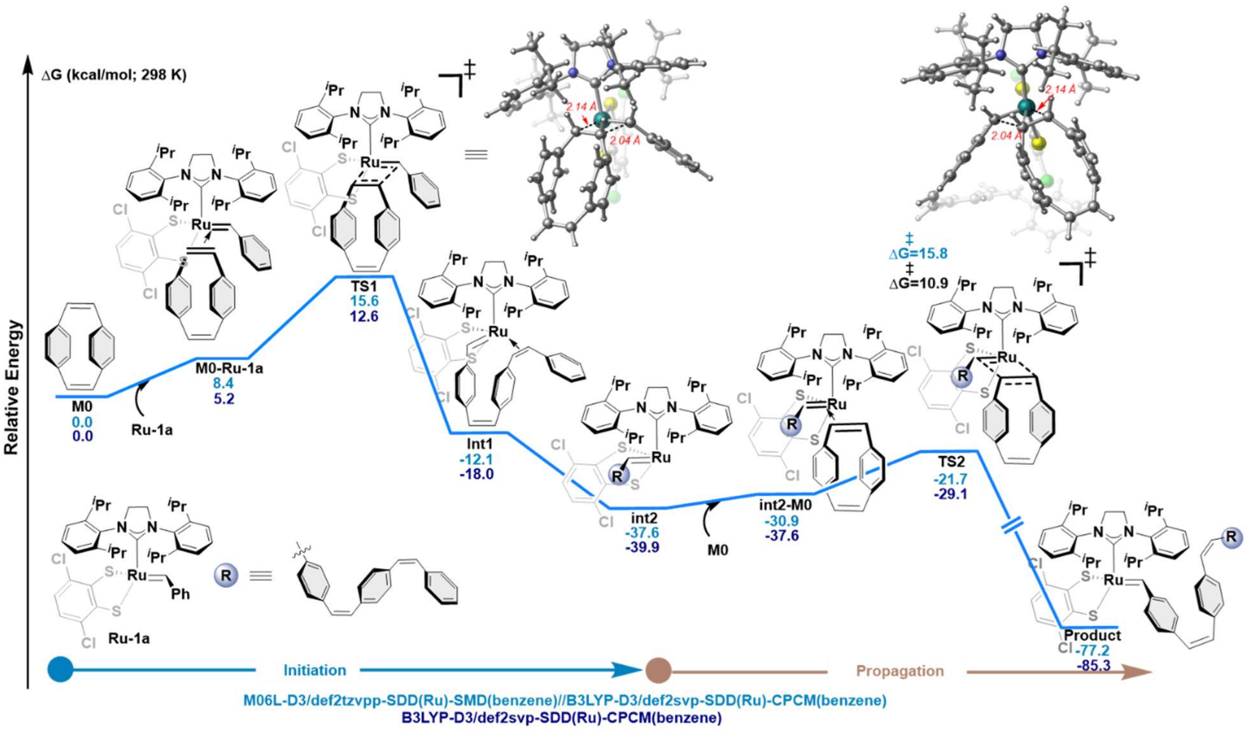

Figure S88. Calculated potential energy surface for ROMP using **M0** with **Ru-1a**.

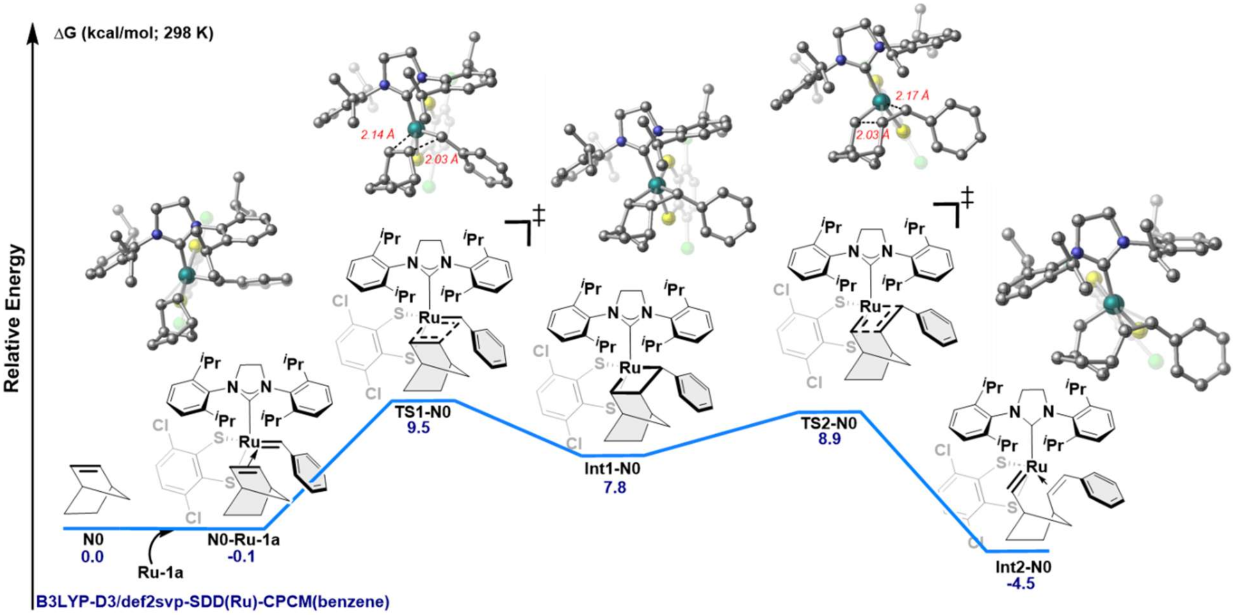

Figure S89. Calculated potential energy surface for ROMP (initiation) using **N0** (Norbornene) with **Ru-1a**.

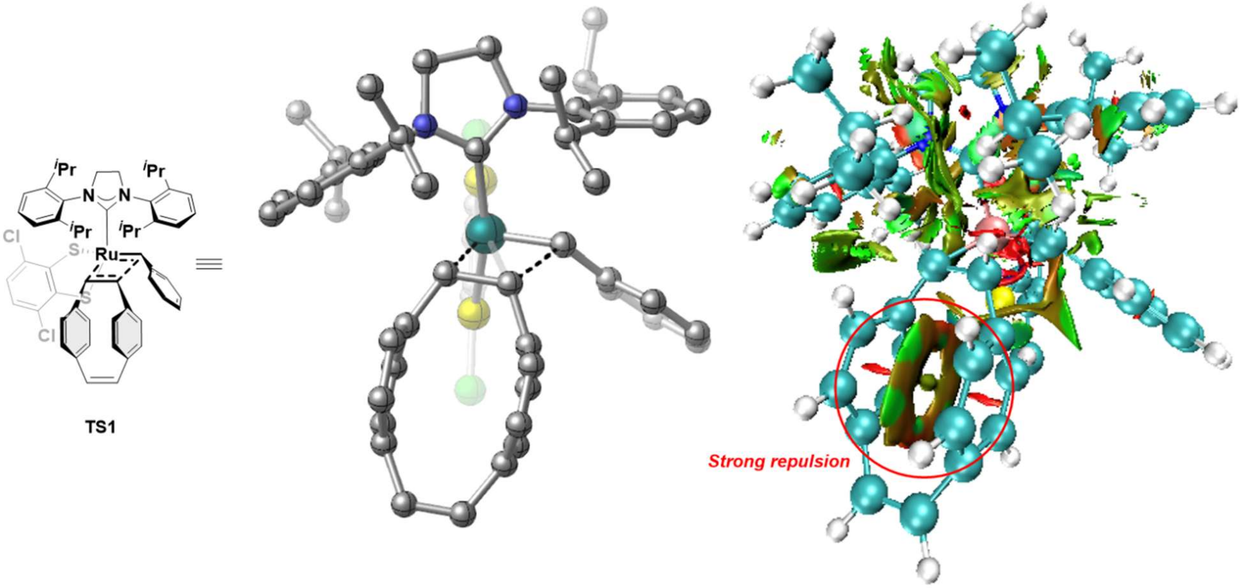

Figure S90. NCI analysis of **TS1**.

To gain further insight into the origins of energy differences for **TS1-*α*1** and **TS1-*β*1** (**Table S1**),[18] we analyzed their distortion and interaction. Single-point energy calculations at the B3LYP-D3/def2svp-SDD(Ru)-CPCM(benzene) level of theory were performed for: (i) the whole structure, (ii) just the **M2**-O^{n}Oct (frag1) and (iii) just the **Ru-1a** fragment (i.e., the remaining fragment after removing the **M2**-O^{n}Oct; frag2). The contributions of distortion and interaction were then calculated as follows:

Potential energy surface (ΔE) = *E* (***TS***) – [*E* (***M2***) + *E*(**Ru-1a**)]
ΔE (distortion) = [*E (fragment 1)* + *E (fragment 2)*] – [*E* (***M2***) + *E*(**Ru-1a**)]
ΔE (interaction) = *ΔE* – *ΔE (distortion)*

Table S2. Single point energies used for the distortion/interaction analysis of **Ru-1a** with **M2** and resulting ΔE values. All energies in the table are in kcal/mol.

	*E(**TS**) a.u.*	*E(***M2***-O^{n}Oct) a.u.*	*E(***Ru-1a***) a.u.*	*E(frag1) a.u.*	*E(frag2) a.u.*	*ΔE*	*$ΔE_{dist}$*	*$ΔE_{int}$*
**TS1-*α*1**	-4867.1230302	-1395.3588018	-3471.7446871	-1395.30572188	-3471.70162093	**-12.3**	**60.3**	**-72.6**
**TS1-*β*1**	-4867.12640713	-1395.3588018	-3471.7446871	-1395.30928126	-3471.70218035	**-14.4**	**57.7**	**-72.1**

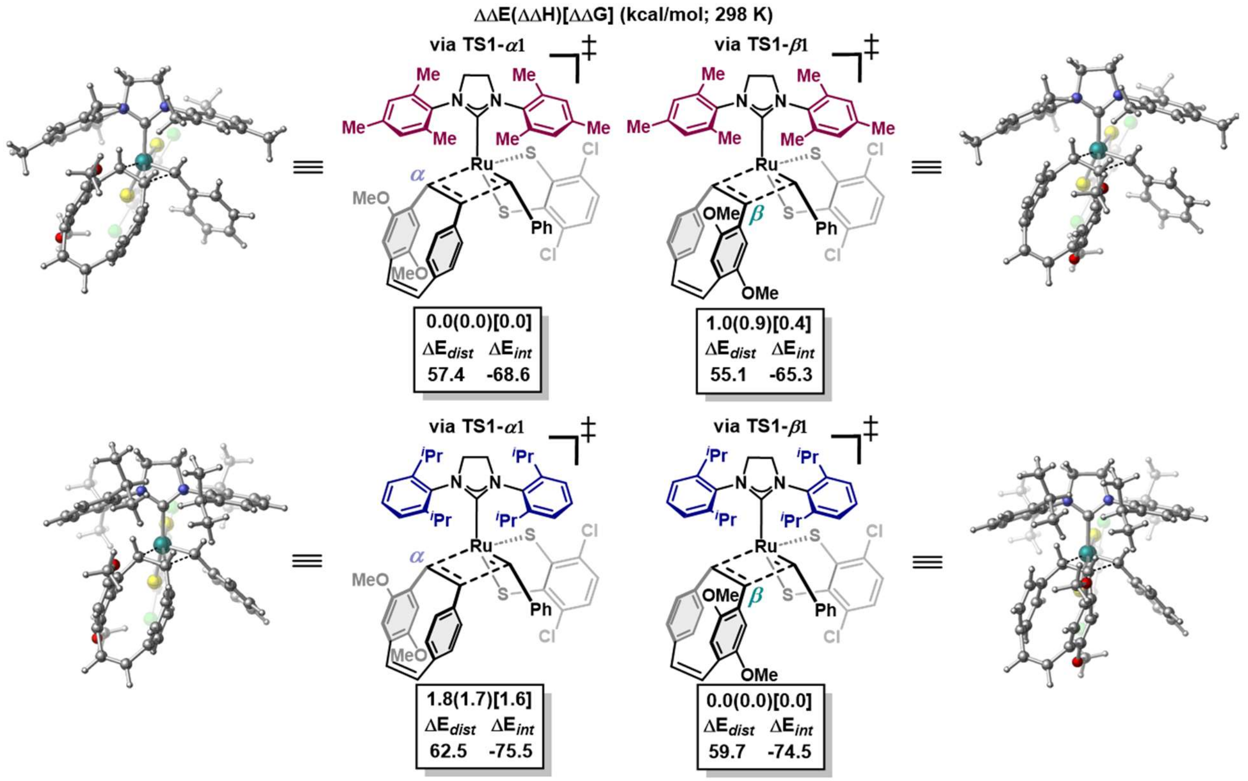

Figure S91. Most favorable regioselective pathway transition state energies (kcal/mol) using **Ru-1b** and **Ru-1a** calculated at B3LYP-D3/def2svp-SDD(Ru)-CPCM(benzene) level of theory.

Table S3. Single point energies used for the distortion/interaction analysis using **Ru-1b** and resulting ΔE values. All energies in the table are in kcal/mol.

	*E(**TS**) a.u.*	*E(**M**-OMe) a.u.*	*E(**Ru-1b**) a.u.*	*E(frag1) a.u.*	*E(frag2) a.u.*	*ΔE*	*ΔE_{dist}*	*ΔE_{int}*
**TS1-*α*1**	-4081.33690156	-845.309882743	-3236.00924284	-845.260161961	-3235.96743719	**-11.2**	**57.4**	**-68.6**
**TS1-*β*1**	-4081.33536713	-845.309882743	-3236.00924284	-845.261834116	-3235.96949573	**-10.2**	**55.1**	**-65.3**

Table S4. Single point energies used for the distortion/interaction analysis using **Ru-1a** and resulting ΔE values. All energies in the table are in kcal/mol.

	*E(**TS**) a.u.*	*E(**M**-OMe) a.u.*	*E(**Ru-1a**) a.u.*	*E(frag1) a.u.*	*E(frag2) a.u.*	*ΔE*	*ΔE_{dist}*	*ΔE_{int}*
**TS1-*α*1**	-4317.06734726	-845.309882743	-3471.68899285	-845.258092189	-3235.96743719	**-13.0**	**62.5**	**-75.5**
**TS1-*β*1**	-4317.07019433	-845.309882743	-3471.69280136	-845.25866205	-3471.70218035	**-14.8**	**59.7**	**-74.5**

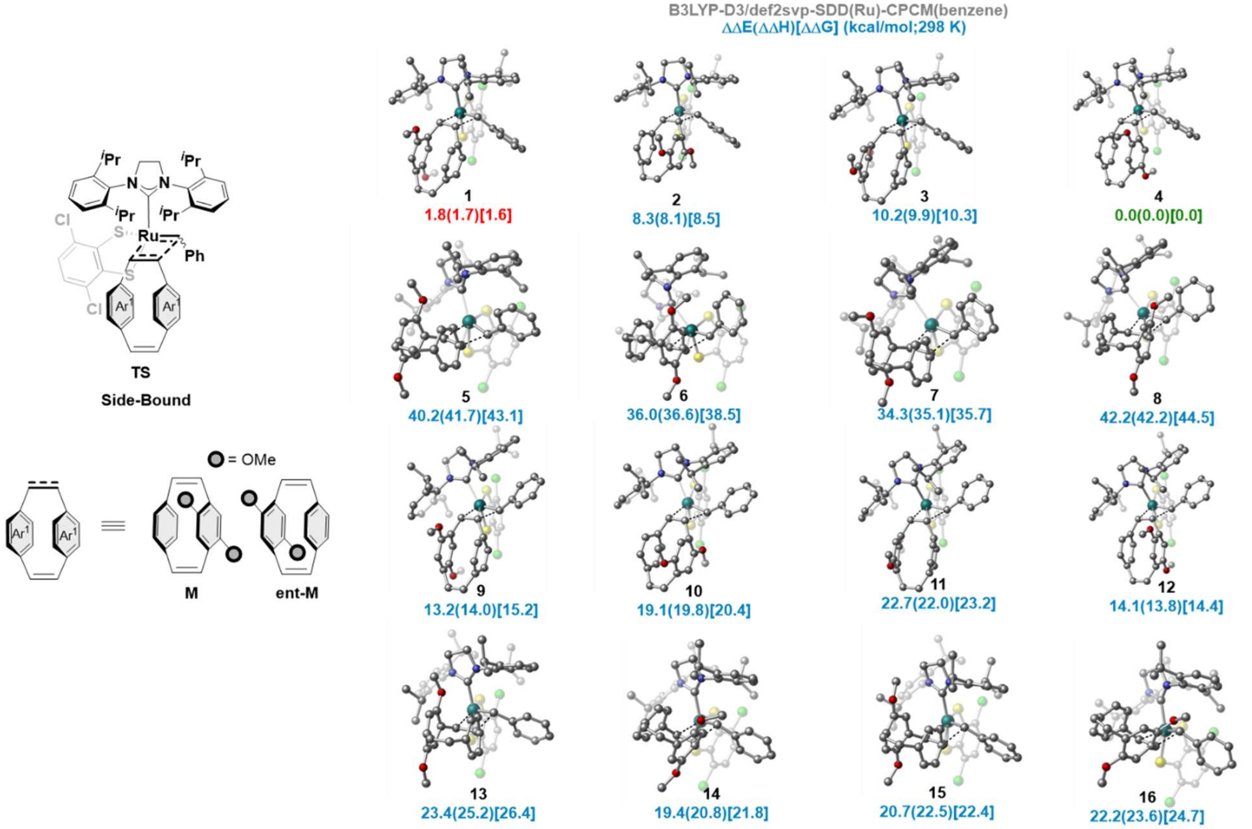

Figure S92. Conformational search for possible transition states in the reactions of truncated **M2**-O^{n}Oct with **Ru-1a**.

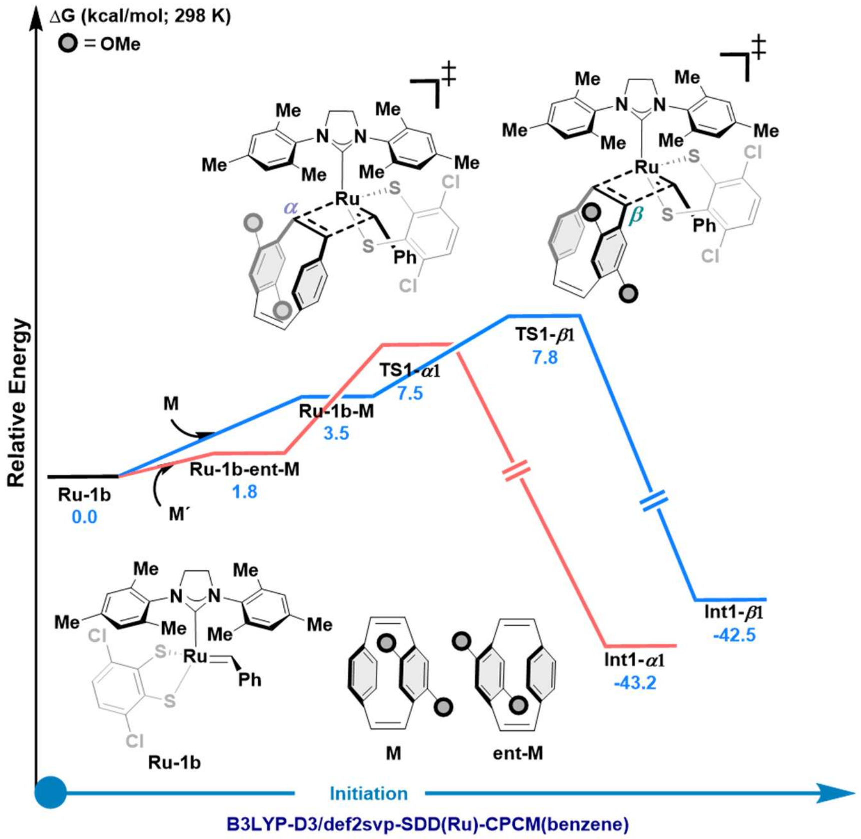

Figure S93. Calculated potential energy surface for ROMP (initiation) using **Ru-1b** and **M**-OMe.

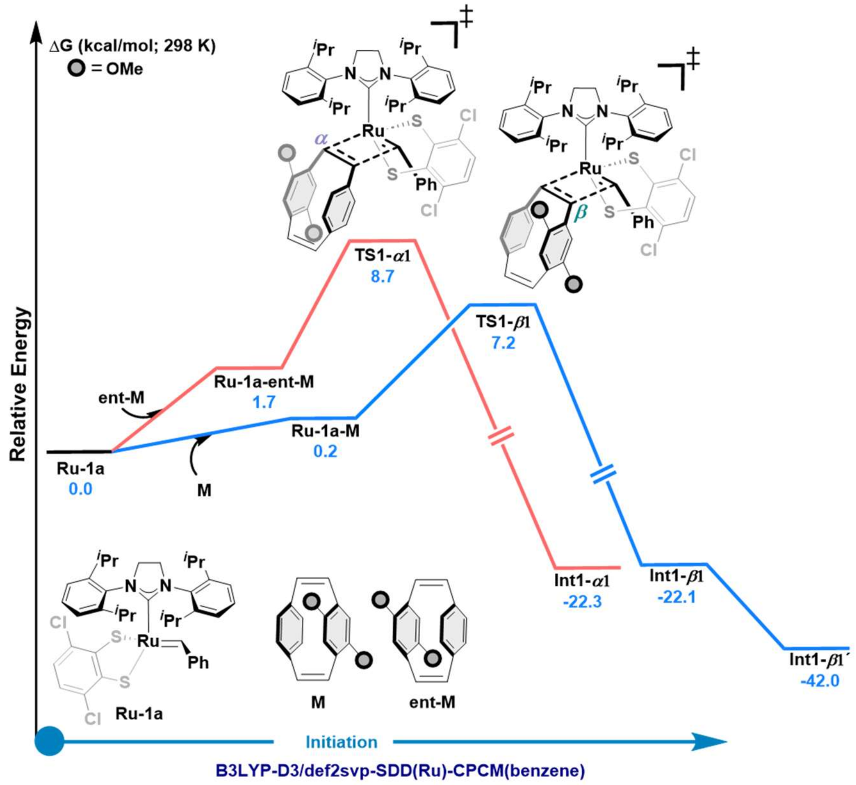

Figure S94. Calculated potential energy surface for ROMP (initiation) using **Ru-1a** and **M**-OMe.

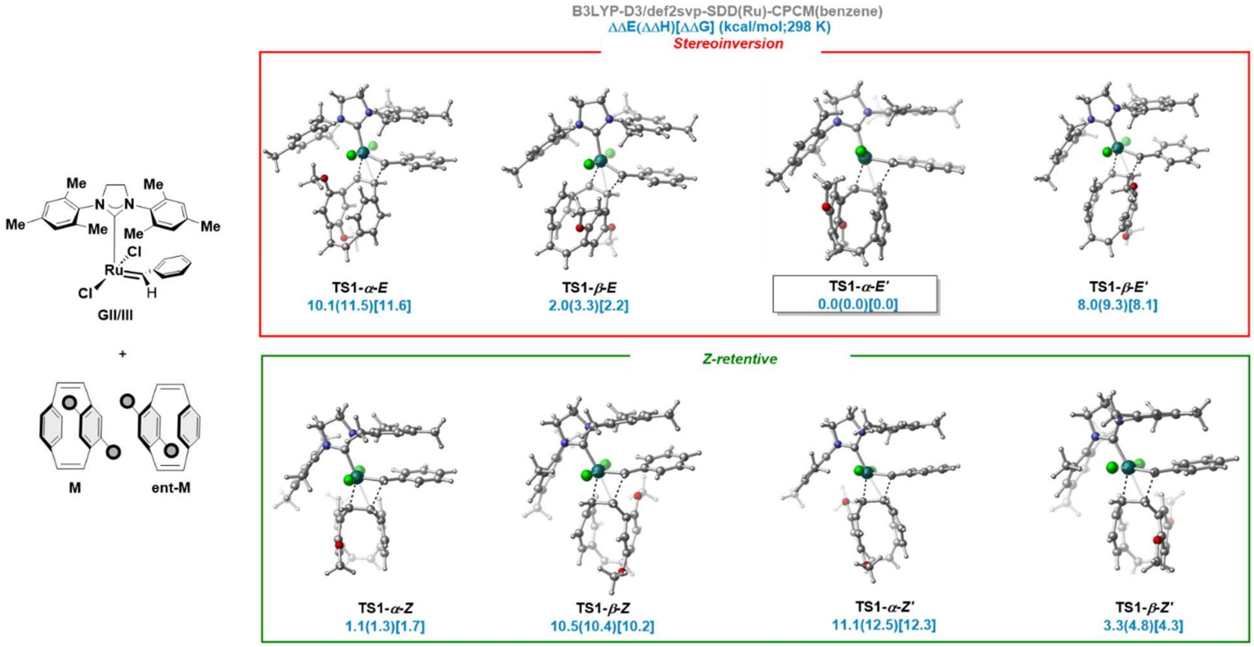

Figure S95. Possible transition states for reactions of **M/ent-M**(-OMe) with **GII/III** show a favorable stereoinverted α-selective pathway.

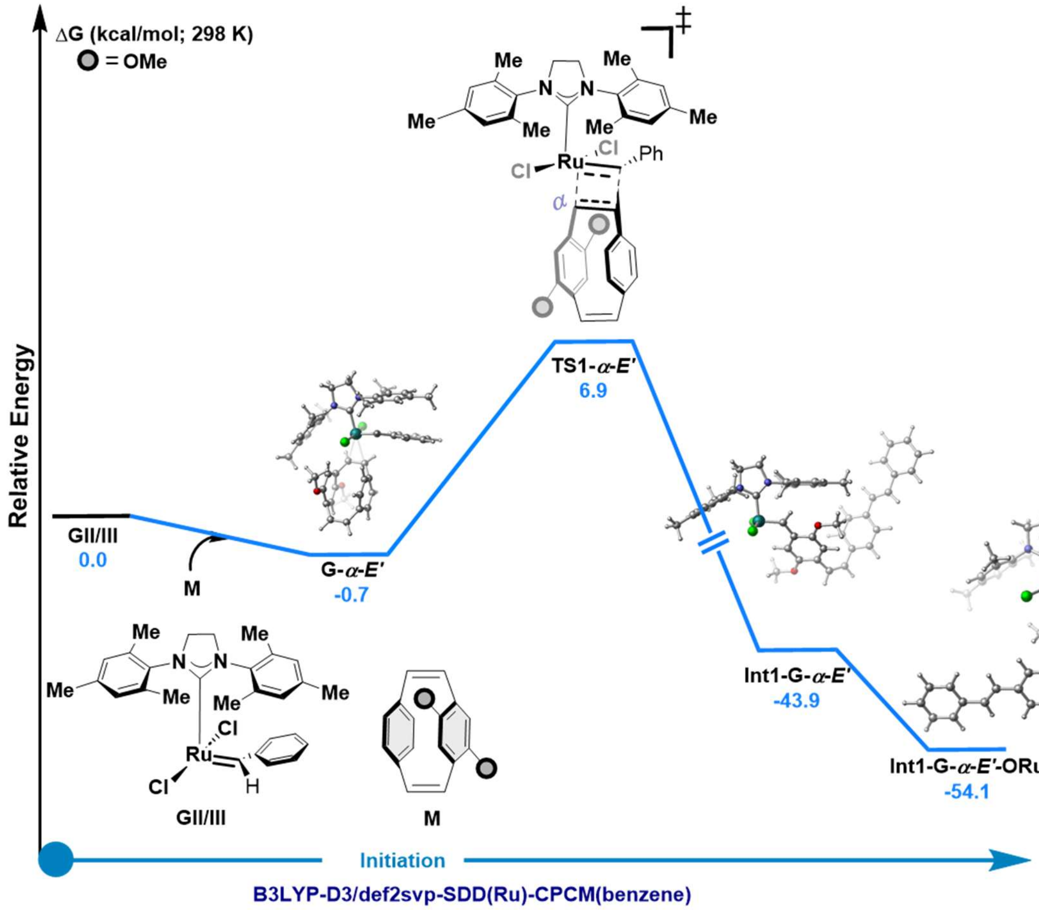

Figure S96. Calculated potential energy surface for stereoinverted ROMP (initiation) using **GII/III** and **M-**OMe.

Table S5. Cartesian coordinates (XYZ format) of all the structures calculated at the B3LYP-D3/def2svp-SDD(Ru)-CPCM(benzene), and single-point energies calculated at M06L-D3/def2tzvpp-SDD(Ru)-SMD(benzene).

TS1

E(scf) = -4088.17224314 a.u.

ν_{min} = -179.98 cm^{-1}

S	-2.220604	0.722673	-0.983774	C	-0.974011	-0.736823	-3.907051
C	-0.984360	-1.769163	-0.354762	H	1.382594	-0.891910	-5.240517
Ru	-0.177874	0.198470	-0.115855	H	3.274194	-3.958493	-2.904666
C	1.814590	-0.546437	-0.351721	H	-3.780089	-2.938087	-0.704718
C	-0.173850	0.137180	1.799781	H	0.412938	-4.207174	-0.475966
S	0.365818	2.498091	-0.534721	H	-1.679782	-0.788946	-3.069297
N	-2.035269	-2.305811	0.322667	C	-4.479801	-0.823800	1.114314
N	-0.855325	-2.470629	-1.511190	H	-4.007277	-0.852432	0.125037
C	-2.351556	-2.151184	1.716703	C	-0.249451	-3.540692	2.282378
C	-2.776637	-3.316343	-0.459710	H	0.129898	-3.099658	1.349776
C	-1.902459	-3.488426	-1.706547	C	-2.274186	2.454742	-1.313962
C	0.253690	-2.442671	-2.427717	C	-3.450851	3.062235	-1.796113
H	-1.455640	-4.491339	-1.786676	C	-1.120802	3.245812	-1.089397
H	-2.448033	-3.286404	-2.640420	C	-3.511751	4.429841	-2.050941
H	-2.898377	-4.242505	0.119669	C	-1.206469	4.633472	-1.353144
C	-3.006953	-1.989015	4.432060	C	-2.379467	5.218845	-1.823247
C	-3.525174	-1.470472	2.107340	H	-4.435514	4.873666	-2.423992
C	-1.505537	-2.767498	2.679425	H	-2.406825	6.292946	-2.012017
C	-1.854368	-2.666082	4.031557	H	1.713048	-1.592323	-0.630851
C	-3.829988	-1.402875	3.477209	C	1.679377	-0.391466	1.138572
H	-1.216731	-3.122151	4.789804	H	1.557315	-1.370271	1.605955
H	-4.731214	-0.875355	3.796133	H	-0.309308	-0.810985	2.324249
C	2.456278	-2.397067	-4.141320	C	-0.301410	1.254478	2.758810
C	1.329502	-3.338705	-2.200823	C	-1.038664	2.421398	2.480414
C	0.238130	-1.580268	-3.544528	C	0.270641	1.109036	4.040698
C	1.365945	-1.565019	-4.380847	C	-1.178742	3.419095	3.447093
C	2.426834	-3.289196	-3.069603	H	-1.524839	2.527009	1.512731
C	1.311823	-4.376830	-1.080743	C	0.155472	2.119517	4.994910

H	0.828940	0.199765	4.276801
C	-0.570327	3.279904	4.699103
H	-1.764589	4.312438	3.217161
H	0.626178	2.000262	5.973973
H	-0.670157	4.069496	5.448317
H	-3.259815	-1.920019	5.492996
H	3.327941	-2.361605	-4.799399
Cl	-4.885731	2.091954	-2.111697
Cl	0.184873	5.670654	-1.088492
C	-5.805953	-1.600445	1.032477
H	-6.343071	-1.575726	1.994962
H	-6.463750	-1.155435	0.268423
H	-5.644225	-2.658474	0.770864
C	-4.727390	0.658709	1.439076
H	-3.776687	1.199534	1.551492
H	-5.286477	1.135587	0.620064
H	-5.308654	0.781975	2.367172
C	-0.580951	-5.016777	1.996456
H	-0.993719	-5.498161	2.897494
H	-1.321789	-5.123511	1.191914
H	0.322644	-5.570388	1.697169
C	0.887133	-3.443909	3.312001
H	0.672009	-4.028271	4.220144
H	1.817064	-3.843709	2.880067
H	1.075082	-2.404440	3.621476
C	1.211093	-5.798881	-1.662587
H	1.097660	-6.540474	-0.855262
H	0.350827	-5.897669	-2.342937
H	2.116809	-6.057629	-2.234352
C	2.512890	-4.267915	-0.127180
H	3.468731	-4.399191	-0.657932
H	2.541807	-3.294103	0.381419
H	2.456968	-5.048399	0.647550

C	-1.680469	-1.317596	-5.145744
H	-2.594060	-0.743078	-5.369612
H	-1.029452	-1.277138	-6.034636
H	-1.967782	-2.370355	-4.991044
C	-0.628521	0.746264	-4.109107
H	-1.550151	1.333079	-4.246199
H	-0.101480	1.156680	-3.235428
H	0.003201	0.900754	-4.998634
C	3.271778	1.346294	-1.399774
C	3.061126	0.025154	-0.979715
C	4.213804	-0.777652	-0.831099
C	5.479050	-0.199533	-0.737370
C	5.633413	1.195830	-0.821889
C	4.543256	1.925131	-1.322488
C	6.724332	1.873513	-0.045405
H	7.655927	2.192060	-0.528953
C	5.094640	0.332815	2.371055
C	5.262384	1.653916	1.919955
C	4.100319	2.423572	1.740840
C	2.841914	1.821173	1.695360
C	2.723615	0.431317	1.828975
C	3.835833	-0.271577	2.330915
C	6.548881	2.087255	1.279511
H	7.341842	2.569413	1.864059
H	6.324542	-0.818104	-0.424449
H	2.416984	1.977803	-1.635597
H	4.103569	-1.840547	-0.605649
H	4.639622	3.002841	-1.480171
H	5.973162	-0.280876	2.587461
H	1.976881	2.413615	1.397959
H	4.190286	3.475133	1.456986
H	3.756703	-1.345983	2.521778

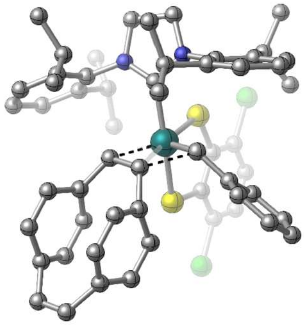

Zero-point correction= 0.995887 (Hartree/Particle)

Thermal correction to Energy= 1.055353

Thermal correction to Enthalpy= 1.056297

Thermal correction to Gibbs Free Energy= 0.903758

Sum of electronic and zero-point Energies= -4087.176356

Sum of electronic and thermal Energies= -4087.116890

Sum of electronic and thermal Enthalpies= -4087.115946

Sum of electronic and thermal Free Energies= -4087.268486

M06L-D3/def2tzvpp-SDD(Ru)-SMD(benzene).

E(scf) = -4090.73293460 a.u.

TS1b

E(scf) = -4088.12283235 a.u.

ν_{min} = -141.47 cm^{-1}

S	-2.484898	0.585657	-0.549851
C	0.166042	1.055737	1.141306
Ru	-0.594584	-0.685499	-0.437358
C	-0.049035	-0.782939	-2.273344
S	-2.013719	-2.549034	-0.781745
N	0.947608	2.167677	1.244563
N	-0.200293	0.716399	2.414160
C	1.368405	3.142926	0.262393
C	0.994087	2.677653	2.642315
C	0.592667	1.448313	3.422286
C	-1.333601	-0.091485	2.815701
H	1.470746	0.843515	3.705087
H	0.008561	1.659137	4.323923
H	1.991168	3.048877	2.896053
C	2.223184	5.323672	-1.296876
C	0.411637	3.950177	-0.407769
C	2.752637	3.433676	0.147376
C	3.151845	4.526999	-0.635563
C	0.869616	5.023055	-1.187551
H	4.214283	4.761036	-0.723856

H	0.140649	5.647957	-1.705962
C	-3.566209	-1.668206	3.408829
C	-2.630698	0.492484	2.782903
C	-1.148159	-1.405436	3.308710
C	-2.285580	-2.184806	3.571084
C	-3.730607	-0.330575	3.056073
H	-2.165576	-3.211496	3.917655
H	-4.738062	0.080159	2.995164
H	0.273257	3.506821	2.746618
C	-3.763826	-0.462533	-1.194798
C	-5.001077	0.068263	-1.609213
C	-3.546800	-1.860154	-1.303290
C	-6.021313	-0.744956	-2.101024
C	-4.593492	-2.667379	-1.799462
C	-5.816123	-2.123543	-2.189368
H	-6.965412	-0.300056	-2.417642
H	-6.600825	-2.778429	-2.570664
Cl	-4.377296	-4.404278	-1.939169
H	-4.440212	-2.297658	3.593973
H	2.553258	6.173866	-1.898783
C	4.286684	-1.125459	1.461704
C	4.936309	-2.365713	1.347745
C	4.128524	-3.516107	1.392035
C	2.747169	-3.416838	1.248801
C	2.130782	-2.168319	1.023729
C	2.901808	-1.027711	1.281918
C	6.322802	-2.482475	0.781553
H	7.199881	-2.569909	1.434268
C	0.866969	-2.213722	0.216036
C	3.106895	-3.356085	-1.994406
C	2.478917	-2.112885	-1.770383
C	3.298806	-0.982331	-1.691618
H	2.852197	0.003236	-1.571394
C	4.679702	-1.125739	-1.556949
C	5.267431	-2.393251	-1.437414
C	4.484553	-3.500221	-1.809701
H	4.917051	-4.504204	-1.794563
C	1.067889	-2.187019	-1.267131
C	6.479401	-2.533076	-0.563014
H	7.480528	-2.668710	-0.989721
H	5.285738	-0.247325	-1.341229
H	2.494821	-4.251734	-2.134182
H	0.250414	-3.070977	0.507938
H	0.500150	-2.986638	-1.751878
C	-2.856671	1.988200	2.577818
H	-2.099721	2.350147	1.872397
C	0.221439	-1.939405	3.697246
H	0.967245	-1.450957	3.057982
C	-1.091773	3.756441	-0.293245
H	-1.276302	2.778070	0.161866
C	3.844964	2.623810	0.835328
H	3.376643	1.737114	1.283122
C	0.371996	-3.456621	3.527546
H	0.052196	-3.791399	2.531119
H	1.423341	-3.747130	3.665602
H	-0.221137	-4.008540	4.273695
C	0.527695	-1.563878	5.163221
H	1.549825	-1.874890	5.434609
H	0.439341	-0.484367	5.348076
H	-0.175872	-2.071324	5.843453
C	-4.233205	2.343744	2.002577
H	-5.037173	2.164056	2.734125
H	-4.456412	1.778356	1.091029
C	-2.667049	2.746637	3.908292
H	-3.420517	2.422697	4.644505
H	-1.678925	2.582531	4.356982
H	-2.789129	3.830484	3.750681
C	4.542923	3.415066	1.957150

H	5.289702	2.783125	2.464358
H	5.067702	4.293905	1.549363
H	3.836466	3.780262	2.717613
C	4.886190	2.143760	-0.188609
H	5.476562	2.984303	-0.584559
H	5.595176	1.440255	0.272926
H	4.401898	1.648783	-1.040592
C	-1.707550	4.828625	0.623019
H	-1.538890	5.838749	0.216032
H	-2.793355	4.676605	0.713294
H	-1.275596	4.799945	1.635463
C	-1.790505	3.740425	-1.661974
H	-2.843684	3.450140	-1.540152
H	-1.763411	4.729126	-2.147533
H	-1.322975	3.008739	-2.336632
H	-0.555377	-1.540735	-2.892158
C	0.670729	0.168491	-3.132643
C	0.929302	-0.185930	-4.473790
C	1.107510	1.423660	-2.676345
C	1.619396	0.682522	-5.320651
H	0.591588	-1.157482	-4.844835
C	1.786343	2.299337	-3.521215
H	0.906262	1.697794	-1.644828
C	2.050222	1.927130	-4.845631
H	1.820028	0.390665	-6.354474
H	2.108788	3.270075	-3.141724
H	2.588461	2.609062	-5.509025
H	-4.267350	3.414564	1.751048
Cl	-5.299972	1.802730	-1.542806
H	2.443396	-0.045899	1.174293
H	4.596762	-4.503593	1.365292
H	4.881375	-0.211113	1.496227
H	2.161212	-4.331846	1.132052

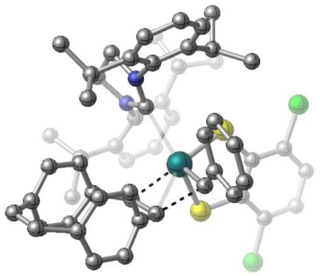

Zero-point correction= 0.997390 (Hartree/Particle)

Thermal correction to Energy= 1.056588

Thermal correction to Enthalpy= 1.057532

Thermal correction to Gibbs Free Energy= 0.905216

Sum of electronic and zero-point Energies= -4087.125442

Sum of electronic and thermal Energies= -4087.066245

Sum of electronic and thermal Enthalpies= -4087.065301

Sum of electronic and thermal Free Energies= -4087.217617

TS1c

E(scf) = -4088.15110919 a.u.

ν_{min} = -79.81 cm^{-1}

S	1.849446	1.651694	0.410029	H	-3.257352	-2.705693	3.949146
C	1.090054	-1.050573	1.068949	H	3.886230	-1.841962	2.395707
Ru	0.003669	0.414258	-0.177050	H	0.050854	-3.366969	2.265308
C	-1.880016	-0.705110	0.226990	H	0.951988	1.274687	2.901287
C	0.120027	0.131014	-2.042757	C	4.933580	-0.374216	0.353987
S	-1.049217	2.499284	-0.676808	H	4.160075	0.003941	1.037249
N	2.327803	-1.596319	0.954656	C	1.169099	-3.765738	-0.678258
N	0.764907	-1.110254	2.393879	H	0.558555	-3.041006	-0.122151
C	3.034083	-2.038713	-0.213668	C	1.487375	3.343103	0.042724
C	2.843382	-2.134684	2.235810	C	2.446743	4.349095	0.275056
C	1.892549	-1.522064	3.247011	C	0.216844	3.704469	-0.475863
C	-0.505146	-0.908683	3.037161	C	2.188927	5.688498	-0.009029
H	1.560854	-2.225631	4.021775	C	-0.022024	5.069714	-0.757152
H	2.319006	-0.634486	3.743505	C	0.944621	6.047721	-0.533622
H	2.798443	-3.235664	2.214719	H	2.953742	6.442354	0.181299
C	4.612193	-3.176161	-2.217707	H	0.720360	7.089881	-0.765212
C	4.335756	-1.530509	-0.444734	H	-1.520571	-1.435537	0.944906
C	2.514176	-3.111178	-0.981355	C	-1.587994	-1.099933	-1.133794
C	3.321560	-3.649536	-1.992411	H	-1.022352	-2.024736	-1.248831
C	5.110390	-2.129255	-1.447710	H	5.227662	-3.620596	-3.003696
H	2.942317	-4.470989	-2.602294	H	-3.959304	-0.467314	4.745426
H	6.119198	-1.756417	-1.638019	Cl	4.011936	3.940483	0.970494
C	-2.978248	-0.596561	4.281910	Cl	-1.576637	5.583239	-1.394677
C	-1.341858	-2.041123	3.206944	C	6.143210	-0.833744	1.189976
C	-0.853568	0.354391	3.560221	H	6.979188	-1.141942	0.541180
C	-2.113825	0.488480	4.165098	H	6.502842	-0.011913	1.829717
C	-2.584738	-1.854257	3.823635	H	5.901701	-1.690793	1.838243
C	-0.907076	-3.447961	2.796272	C	5.336316	0.805550	-0.547521
C	0.125815	1.519230	3.580852	H	4.473618	1.207674	-1.094883
H	-2.414919	1.460640	4.560371	H	5.736381	1.628912	0.062904

H	6.111213	0.514735	-1.275063
C	1.366372	-5.003878	0.216858
H	1.993113	-5.752115	-0.294677
H	1.859554	-4.752205	1.166947
H	0.398760	-5.472747	0.451956
C	0.377209	-4.148064	-1.938294
H	0.839187	-4.992719	-2.473099
H	-0.641957	-4.461185	-1.660978
H	0.304612	-3.306836	-2.641109
C	-0.668523	-4.319834	4.043219
H	-0.258408	-5.301669	3.756259
H	0.037540	-3.844336	4.741798
H	-1.609322	-4.492620	4.590483
C	-1.887785	-4.139571	1.835173
H	-2.900277	-4.206900	2.262842
H	-1.958892	-3.608539	0.874800
H	-1.552892	-5.166716	1.624133
C	0.714065	1.685003	4.995064
H	1.478544	2.478976	5.001331
H	-0.069481	1.962621	5.719498
H	1.183228	0.755044	5.354251
C	-0.483588	2.836820	3.082619
H	0.299675	3.606638	3.002667
H	-0.934887	2.716178	2.087915
H	-1.255127	3.218062	3.771192
C	-3.824159	1.026176	0.404539
C	-3.284204	-0.258949	0.546619
C	-4.203926	-1.319929	0.687910
C	-5.541421	-1.155063	0.338926
C	-6.006659	0.084354	-0.136191
C	-5.174649	1.196562	0.069433
C	-7.108164	0.091108	-1.156350
H	-8.144013	0.320861	-0.878418
C	-4.841450	-1.811495	-2.737767
C	-5.387202	-0.515927	-2.806394
C	-4.483329	0.556187	-2.860972
C	-3.141781	0.378623	-2.525010
C	-2.662409	-0.886415	-2.156379
C	-3.489989	-1.995880	-2.428164
C	-6.814222	-0.232574	-2.437943
H	-7.613945	-0.276770	-3.187067
H	-0.646077	0.633611	-2.647272
C	1.113166	-0.447860	-2.950683
C	2.486265	-0.203707	-2.790139
C	0.666414	-1.110699	-4.116756
C	3.389834	-0.586749	-3.781752
H	2.818025	0.328853	-1.902082
C	1.580323	-1.553174	-5.071155
H	-0.404976	-1.274259	-4.262216
C	2.945325	-1.277966	-4.912347
H	4.449969	-0.359651	-3.657938
H	1.227346	-2.088111	-5.956444
H	3.659915	-1.597921	-5.674927
H	-5.521211	2.192724	-0.218914
H	-3.836153	-2.325753	0.895464
H	-5.504067	-2.680890	-2.760029
H	-2.523221	1.265604	-2.383945
H	-6.184669	-2.036156	0.266021
H	-3.159935	1.889802	0.388744
H	-4.864415	1.573335	-2.977855
H	-3.125771	-3.005864	-2.218370

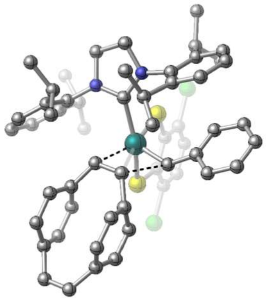

Zero-point correction= 0.995293 (Hartree/Particle)

Thermal correction to Energy= 1.054830

Thermal correction to Enthalpy= 1.055774

Thermal correction to Gibbs Free Energy= 0.903908

Sum of electronic and zero-point Energies= -4087.155816

Sum of electronic and thermal Energies= -4087.096279

Sum of electronic and thermal Enthalpies= -4087.095335

Sum of electronic and thermal Free Energies= -4087.247201

TS1d

E(scf) = -4088.15026766 a.u.

ν_{min} = -282.74 cm^{-1}

S	2.615969	0.222017	0.237832
C	-0.146548	1.585807	-0.190034
Ru	0.506879	-0.458069	-0.249361
C	-0.555336	-1.047734	1.262205
S	1.621316	-2.416187	-1.173847
N	-0.931508	2.339207	0.650567
N	0.243599	2.421844	-1.200570
C	-1.369648	2.013495	1.984029
C	-0.925601	3.770056	0.261668
C	-0.517344	3.680902	-1.190442
C	1.383660	2.310878	-2.080029
H	-1.392591	3.584213	-1.856656
H	0.090214	4.522428	-1.535065
H	-1.902098	4.237064	0.397903
C	-2.247608	1.283554	4.543171
C	-0.415463	1.726603	2.996556
C	-2.754012	2.088890	2.298940
C	-3.166573	1.689579	3.579313
C	-0.886129	1.338309	4.258866
H	-4.227147	1.718120	3.833737
H	-0.168618	1.081624	5.038760
C	3.606501	2.173957	-3.763555
C	2.578540	2.982502	-1.708646
C	1.281535	1.615511	-3.303102
C	2.416049	1.549416	-4.126042
C	3.677675	2.893416	-2.571976
H	2.363641	1.006313	-5.070640
H	4.610744	3.392792	-2.304408

H	-0.192873	4.314821	0.880996
C	3.708487	-1.174164	0.097823
C	5.011411	-1.126784	0.626759
C	3.272427	-2.346410	-0.565941
C	5.892709	-2.199381	0.488130
C	4.180860	-3.416637	-0.704629
C	5.474847	-3.347637	-0.186981
H	6.896173	-2.134961	0.910684
H	6.148350	-4.197555	-0.305133
Cl	3.694313	-4.888667	-1.525690
Cl	5.570141	0.293989	1.500944
H	4.480466	2.108784	-4.416675
H	-2.590988	0.959755	5.528792
C	-0.197089	-1.998202	2.340421
C	0.938160	-2.835048	2.303222
C	-1.015551	-2.039271	3.490042
C	1.238048	-3.678045	3.373401
H	1.587896	-2.812032	1.431096
C	-0.713086	-2.880465	4.561430
H	-1.892049	-1.391393	3.544313
C	0.416647	-3.704391	4.507130
H	2.125543	-4.314030	3.324294
H	-1.359652	-2.891097	5.442694
H	0.658335	-4.362049	5.346122
C	-4.590539	0.318259	-1.743158
C	-5.258843	-0.648164	-2.515143
C	-4.470511	-1.452614	-3.356828
C	-3.085595	-1.501233	-3.186744
C	-2.459555	-0.769872	-2.153063
C	-3.209541	0.253819	-1.551704
C	-6.655296	-1.054973	-2.142657
H	-7.519106	-0.727030	-2.733590
C	-1.257455	-1.379637	-1.532168
C	-3.463279	-3.342596	-0.547246
C	-2.868122	-2.304812	0.200135
C	-3.721891	-1.496077	0.959726
H	-3.323004	-0.723194	1.617408
C	-5.105054	-1.531675	0.737238
C	-5.649776	-2.319690	-0.286906
C	-4.830962	-3.335371	-0.809120
H	-5.232794	-4.025099	-1.555613
C	-1.432650	-2.048832	-0.210286
C	-6.845748	-1.853510	-1.065424
H	-7.862463	-2.151937	-0.783098
H	-5.178489	1.014286	-1.145476
H	-2.513452	-2.258292	-3.729740
H	-0.681140	-1.981770	-2.239522
H	-0.829531	-2.959327	-0.140837
H	-1.385848	-0.409283	1.578326
C	2.704832	3.832677	-0.445519
H	1.862458	3.585019	0.214624
C	-0.030644	1.004302	-3.764785
H	-0.636646	0.819787	-2.870485
C	1.081971	1.944436	2.816386
H	1.312294	1.914213	1.747343
C	-3.786340	2.742655	1.381041
H	-3.393109	2.738107	0.353728
C	0.153122	-0.338511	-4.482678
H	0.757231	-1.031663	-3.877562
H	-0.827491	-0.799697	-4.669308
H	0.644254	-0.218544	-5.461385
C	-0.810271	1.996555	-4.646655
H	-1.793331	1.578271	-4.918262
H	-0.978367	2.953656	-4.128656
H	-0.259107	2.212994	-5.576953
C	3.987896	3.560938	0.355651
H	4.887203	3.877939	-0.195919
H	3.967564	4.134786	1.295922

H	4.094429	2.498018	0.600803	C	1.463455	3.348065	3.327034
C	2.626788	5.335142	-0.785210	H	1.259991	3.442295	4.406350
H	3.496826	5.635439	-1.391407	H	2.536507	3.533621	3.162982
H	1.725889	5.597581	-1.359431	H	0.897953	4.140010	2.811776
H	2.631631	5.940448	0.135854	C	1.950931	0.873271	3.489287
C	-4.003582	4.207219	1.826346	H	3.000259	0.998503	3.184946
H	-4.642528	4.742393	1.105399	H	1.909631	0.942864	4.588154
H	-4.504568	4.230091	2.807438	H	1.639828	-0.136687	3.190840
H	-3.061836	4.764506	1.935134	H	-4.950403	-2.172426	-4.025268
C	-5.144361	2.034208	1.343953	H	-2.736702	0.893569	-0.805195
H	-5.654096	2.063873	2.319135	H	-2.826141	-4.048755	-1.086527
H	-5.808593	2.531358	0.619649	H	-5.742137	-0.803432	1.241060
H	-5.037296	0.988549	1.045992				

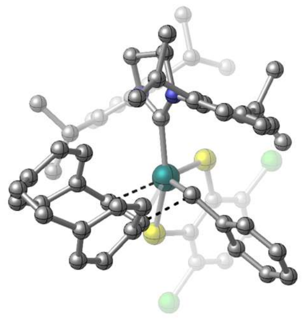

Zero-point correction= 0.998462 (Hartree/Particle)

Thermal correction to Energy= 1.057707

Thermal correction to Enthalpy= 1.058651

Thermal correction to Gibbs Free Energy= 0.906789

Sum of electronic and zero-point Energies= -4087.151805

Sum of electronic and thermal Energies= -4087.092561

Sum of electronic and thermal Enthalpies= -4087.091616

Sum of electronic and thermal Free Energies= -4087.243478

TS1e

E(scf) = -4088.12703007 a.u.

ν_{min} = -302.36 cm^{-1}

Ru	0.023860	0.052316	-0.310011
C	1.360884	1.257328	0.752572
C	2.068405	2.504529	2.614617
C	3.193198	2.458123	1.589797
H	2.269928	1.856526	3.484035
H	4.144672	2.130768	2.017953
C	-0.916687	0.723159	-1.939030
H	1.845017	3.516521	2.975394
H	3.357344	3.425345	1.085833
N	0.944020	1.960040	1.839993
N	2.689865	1.472281	0.609332
C	3.570505	1.100983	-0.463916
C	4.604379	0.168957	-0.213139
C	3.441461	1.749364	-1.714839
C	5.487118	-0.129120	-1.260672
C	4.353471	1.416845	-2.724536
C	5.363661	0.481731	-2.506459
H	6.283140	-0.858197	-1.097892
H	4.271051	1.895798	-3.701979
C	-0.405837	2.354459	2.114590
C	-0.868157	3.573147	1.557972
C	-1.195680	1.593244	3.007545
C	-2.183610	3.962720	1.842411
C	-2.522117	2.000352	3.215799
C	-3.015331	3.166831	2.630132
H	-2.567208	4.897255	1.428417
H	-3.165718	1.415539	3.875436
C	-1.374098	-1.672285	-0.359080
C	-1.718285	-1.145894	-1.719997
S	1.072548	-1.470087	1.576984
C	1.786582	-2.717612	0.559044
C	2.218275	-3.940418	1.120949
C	1.985529	-2.533959	-0.842981
C	2.828927	-4.943502	0.367393
Cl	2.050824	-4.223323	2.849279
S	1.514938	-1.039651	-1.682158
C	2.616473	-3.553883	-1.582829
C	3.035106	-4.749355	-0.997035
H	3.149213	-5.865533	0.854162
Cl	2.913196	-3.337918	-3.304238
H	3.516067	-5.514260	-1.607802
H	6.059349	0.230111	-3.311047
H	-4.047942	3.474147	2.815070
C	2.370386	2.797824	-1.993670
H	1.578566	2.690768	-1.241162
C	4.796973	-0.507835	1.139698
H	3.857198	-0.393582	1.701212
C	0.043307	4.509218	0.767033
H	0.995090	3.991239	0.591628
C	-0.579892	0.489107	3.855140
H	0.347410	0.167785	3.362978
C	1.694816	2.609701	-3.360603
H	1.389931	1.564626	-3.509520
H	0.796747	3.243270	-3.428646
H	2.361767	2.890833	-4.191048
C	2.952344	4.217828	-1.868829
H	3.767779	4.367653	-2.595107
H	2.177988	4.975433	-2.065780
H	3.364012	4.405140	-0.865203
C	5.073756	-2.017109	1.014906
H	4.911256	-2.513132	1.984272
H	4.414538	-2.491496	0.280483
H	6.115178	-2.213335	0.711511
C	5.946930	0.143451	1.934885
H	5.814407	1.225965	2.079923
H	6.040105	-0.323383	2.929019
H	6.904184	0.004668	1.405857
C	-0.192804	1.076858	5.228064

H	-1.090327	1.391681	5.785954
H	0.338065	0.323229	5.831854
H	0.460906	1.957502	5.126900
C	-1.458824	-0.753717	4.025791
H	-1.723614	-1.189878	3.055249
H	-0.910834	-1.518659	4.597324
H	-2.387847	-0.531153	4.575918
C	0.352330	5.768468	1.599870
H	1.093886	6.398981	1.083410
H	-0.556606	6.372699	1.752793
H	0.751165	5.514460	2.594547
C	-0.505318	4.895977	-0.613896
H	0.173952	5.614932	-1.097062
H	-0.600937	4.020575	-1.268903
H	-1.496366	5.369292	-0.545409
C	-3.567034	-2.638320	-2.251998
C	-3.191218	-1.287794	-2.079213
C	-4.235311	-0.362512	-1.953387
H	-4.033036	0.700151	-1.902826
C	-5.541977	-0.789622	-1.682408
C	-5.831646	-2.149988	-1.509584
C	-4.860214	-3.064086	-1.956626
H	-5.066818	-4.136472	-1.911950
C	-6.894216	-2.610543	-0.554406
H	-7.919484	-2.800619	-0.894060
C	-4.712757	-1.209094	1.259075
C	-5.162782	-2.540041	1.188215
C	-4.184327	-3.550332	1.156811
H	-4.488897	-4.600239	1.174841
C	-2.852393	-3.232260	0.876858
C	-2.481363	-1.900847	0.600309
C	-3.390383	-0.892656	0.960543
H	-3.104453	0.154205	0.861749
C	-6.570114	-2.809959	0.745170
H	-7.328624	-3.178868	1.445971
H	-6.290448	-0.037945	-1.417266
H	-2.795775	-3.391565	-2.435826
H	-0.665848	-2.504537	-0.397351
H	-1.050812	-1.588403	-2.468199
H	-0.460802	0.504652	-2.916011
C	-1.925508	1.790740	-2.073787
C	-2.644557	2.304981	-0.974736
C	-2.175533	2.354817	-3.342971
C	-3.598582	3.305376	-1.140377
H	-2.457496	1.906756	0.021828
C	-3.113294	3.376519	-3.508887
H	-1.628473	1.974658	-4.209560
C	-3.839449	3.846963	-2.409426
H	-4.149934	3.671940	-0.272427
H	-3.288431	3.797270	-4.502290
H	-4.585011	4.635398	-2.538816
H	-5.440902	-0.401329	1.364002
H	-2.135546	-4.037591	0.694662

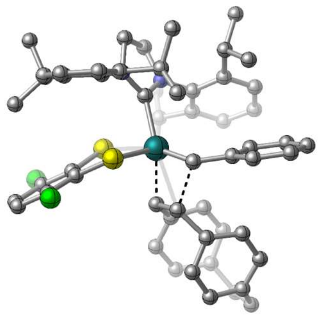

Zero-point correction= 0.996612 (Hartree/Particle)

Thermal correction to Energy= 1.056235

Thermal correction to Enthalpy= 1.057180

Thermal correction to Gibbs Free Energy= 0.904180

Sum of electronic and zero-point Energies= -4087.130418

Sum of electronic and thermal Energies= -4087.070795

Sum of electronic and thermal Enthalpies= -4087.069850

Sum of electronic and thermal Free Energies= -4087.222850

TS1f

E(scf) = -4088.13322072 a.u.

ν_{min} = -226.77 cm^{-1}

Ru	0.131133	0.046060	-0.400106
C	-1.162497	1.047321	0.982954
C	-2.711966	2.533435	1.949011
C	-1.575920	2.295292	2.930925
H	-3.664692	2.090115	2.271551
H	-1.910167	2.039209	3.941675
C	0.670310	1.618513	-1.479484
H	-2.882463	3.596276	1.744559
H	-0.918138	3.175907	2.998547
N	-2.221808	1.845150	0.734416
N	-0.840380	1.174859	2.307289
H	0.173682	1.736149	-2.455305
C	-0.189560	0.180879	3.134448
C	-0.991058	-0.839258	3.713184
C	1.196696	0.267628	3.416686
C	-0.355967	-1.812793	4.497876
C	1.780201	-0.738548	4.199804
C	1.016250	-1.779679	4.723374
H	-0.949968	-2.616003	4.936342
H	2.848666	-0.707472	4.412945
C	-3.046443	1.933526	-0.447028
C	-2.930338	3.068369	-1.281276
C	-4.039142	0.946234	-0.676457
C	-3.799238	3.180699	-2.377453
C	-4.887418	1.115330	-1.778399
C	-4.768657	2.213920	-2.627557
H	-3.719783	4.046307	-3.038050
H	-5.652154	0.364934	-1.983284
C	1.427093	-1.277121	-1.356203
C	1.777550	0.116049	-1.927047
H	0.906855	-1.876477	-2.112002
H	1.442664	0.122446	-2.971223
S	-1.385127	-0.457059	-2.092609
C	-1.607973	-2.212531	-1.946739
C	-2.121446	-2.959148	-3.022362
C	-1.370984	-2.866438	-0.704117
C	-2.423138	-4.317918	-2.904729
Cl	-2.444692	-2.170684	-4.562374
S	-0.746170	-1.976681	0.687015
C	-1.692167	-4.235217	-0.608781

C	-2.208194	-4.959447	-1.686246
H	-2.824873	-4.860572	-3.761371
Cl	-1.462635	-5.086541	0.914177
H	-2.436666	-6.019217	-1.565619
H	1.492236	-2.559033	5.323718
H	-5.438496	2.317656	-3.485073
C	2.032304	1.458235	2.964870
H	1.689924	1.726323	1.957134
C	-2.513479	-0.875795	3.597605
H	-2.804182	-0.250037	2.744707
C	-4.242676	-0.259688	0.235034
H	-3.285089	-0.473174	0.728705
C	-1.938239	4.190899	-1.008422
H	-1.211401	3.819580	-0.274809
C	-3.086851	-2.279123	3.342561
H	-4.171503	-2.210462	3.167238
H	-2.621010	-2.751068	2.468731
H	-2.946023	-2.940056	4.212660
C	-3.160554	-0.287120	4.868778
H	-2.943435	-0.925182	5.740789
H	-2.793540	0.722478	5.106087
H	-4.255026	-0.232641	4.752361
C	1.803994	2.672985	3.886120
H	2.189140	2.458437	4.896311
H	2.338246	3.555634	3.498662
H	0.744513	2.934892	3.989580
C	3.538121	1.184124	2.886102
H	3.763276	0.281810	2.306668
H	4.044345	2.034898	2.404688
H	3.982467	1.067456	3.886965
C	-1.155493	4.613016	-2.262476
H	-0.314592	5.266111	-1.983848
H	-0.745502	3.745833	-2.796937
H	-1.793079	5.170448	-2.967153
C	-2.639924	5.421355	-0.401150
H	-1.902701	6.201983	-0.151727
H	-3.359565	5.850518	-1.117281
H	-3.198768	5.175583	0.514521
C	-5.312301	0.033441	1.306207
H	-6.279228	0.259530	0.827816
H	-5.454536	-0.842230	1.957749
H	-5.056633	0.888068	1.947455
C	-4.638777	-1.539115	-0.520513
H	-4.017993	-1.693142	-1.409686
H	-4.508555	-2.412111	0.137305
H	-5.694865	-1.517725	-0.835346
C	1.416294	2.860852	-1.151843
C	2.188468	3.509410	-2.135761
C	1.326794	3.455322	0.117195
C	2.873726	4.689317	-1.848383
H	2.258671	3.068768	-3.133189
C	1.990011	4.651216	0.403789
H	0.701312	2.987451	0.875027
C	2.775901	5.266225	-0.575473
H	3.479991	5.168152	-2.621284
H	1.891348	5.101474	1.394629
H	3.304141	6.196500	-0.352283
C	5.457588	-0.229849	-2.439164
C	5.925679	-0.075365	-1.122392
C	5.109177	0.640913	-0.232805
C	3.774487	0.901293	-0.540923
C	3.229527	0.476659	-1.757757
C	4.125021	0.051291	-2.755476
C	7.004392	-0.959114	-0.565743
H	8.058062	-0.655708	-0.589951
C	2.981177	-1.844201	0.607028
C	2.525893	-2.049569	-0.706635
C	3.337373	-2.828848	-1.553552

C	4.671736	-3.071334	-1.229651	H	5.335235	-3.500332	-1.985217
C	5.216922	-2.562319	-0.038239	H	2.328547	-1.354923	1.332621
C	4.313614	-2.106382	0.939008	H	5.467760	0.832115	0.780011
C	6.659827	-2.148871	-0.018434	H	3.742760	-0.229109	-3.741207
H	7.436211	-2.798834	0.403167	H	2.975209	-3.088092	-2.552496
H	6.084354	-0.731551	-3.181177	H	4.693157	-1.785517	1.912705
H	3.118259	1.299844	0.230147				

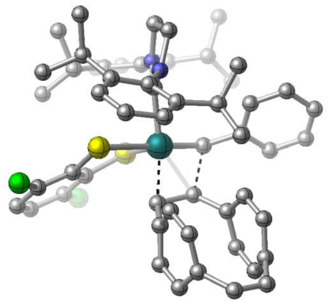

Zero-point correction= 0.995703 (Hartree/Particle)

Thermal correction to Energy= 1.054998

Thermal correction to Enthalpy= 1.055943

Thermal correction to Gibbs Free Energy= 0.903790

Sum of electronic and zero-point Energies= -4087.137517

Sum of electronic and thermal Energies= -4087.078222

Sum of electronic and thermal Enthalpies= -4087.077278

Sum of electronic and thermal Free Energies= -4087.229430

TS1g

E(scf) = -4088.14492901 a.u.

ν_{min} = -237.91 cm^{-1}

Ru	-0.084242	0.149566	-0.274097	H	2.903639	-0.527816	3.468552
C	0.980602	0.561120	1.536443	C	-0.558301	1.918424	-1.026533
C	1.189446	0.746973	3.878997	H	1.090303	1.757319	4.302415
C	2.559829	0.494854	3.262174	H	3.334012	1.197481	3.597443
H	0.942383	0.023542	4.663788	N	0.299126	0.593553	2.712525

N	2.296286	0.648929	1.815756
C	3.380031	0.893065	0.903989
C	4.334383	-0.121375	0.663221
C	3.495472	2.180910	0.319946
C	5.398014	0.167560	-0.203454
C	4.567529	2.405575	-0.554301
C	5.509777	1.411134	-0.816992
H	6.142614	-0.602420	-0.412850
H	4.679554	3.375311	-1.038213
C	-1.129892	0.587130	2.873443
C	-1.866117	1.783938	2.650758
C	-1.770296	-0.591439	3.336925
C	-3.259425	1.739403	2.806730
C	-3.166101	-0.573179	3.481075
C	-3.907990	0.569932	3.199735
H	-3.852138	2.636399	2.625893
H	-3.679086	-1.474351	3.820978
C	-1.269761	-0.944555	-1.615606
C	-1.498439	0.515706	-1.995034
S	0.464438	-2.083993	0.665076
C	1.322107	-2.829962	-0.681957
C	1.557366	-4.217783	-0.721282
C	1.834830	-2.035103	-1.743568
C	2.247891	-4.827640	-1.771718
Cl	1.007912	-5.240992	0.600981
S	1.714712	-0.268187	-1.682238
C	2.518398	-2.666634	-2.798554
C	2.729452	-4.047398	-2.821559
H	2.402944	-5.907262	-1.762054
Cl	3.167630	-1.707119	-4.123656
H	3.267753	-4.499996	-3.655287
H	6.337433	1.611913	-1.502210
H	-4.995591	0.557788	3.305870
C	2.510342	3.306853	0.641428

H	1.498952	2.882675	0.553499
C	4.263858	-1.496274	1.319883
H	3.225628	-1.653063	1.649615
C	-1.188097	3.123050	2.364730
H	-0.271533	2.927400	1.790765
C	-1.005420	-1.837820	3.771313
H	0.016022	-1.766438	3.372577
C	2.579153	4.492806	-0.327026
H	2.483274	4.178739	-1.374248
H	1.756141	5.191205	-0.114640
H	3.522382	5.052590	-0.216724
C	2.686206	3.828975	2.079798
H	3.698635	4.241806	2.219021
H	1.960959	4.631973	2.286802
H	2.535223	3.049976	2.835738
C	4.598570	-2.650395	0.359801
H	4.313637	-3.610258	0.817925
H	4.059471	-2.559167	-0.589382
H	5.677169	-2.698005	0.138621
C	5.208544	-1.565707	2.537857
H	5.030048	-0.759322	3.263767
H	5.097116	-2.529746	3.060775
H	6.257330	-1.479404	2.208963
C	-0.927880	-1.892681	5.311314
H	-1.932315	-2.027460	5.745005
H	-0.303017	-2.740751	5.634423
H	-0.507729	-0.972013	5.744951
C	-1.606681	-3.147406	3.235114
H	-1.716836	-3.125717	2.143663
H	-0.939565	-3.987890	3.482044
H	-2.588453	-3.361584	3.687397
C	-0.786426	3.811438	3.685833
H	-0.263118	4.759159	3.482358
H	-1.683419	4.037312	4.284873

H	-0.126878	3.188127	4.302278
C	-2.053488	4.102948	1.555723
H	-1.437120	4.950519	1.219750
H	-2.512111	3.638330	0.671617
H	-2.869661	4.520271	2.165885
C	-3.740763	0.763417	-3.063793
C	-2.943534	0.950676	-1.919506
C	-3.605183	1.192337	-0.708434
H	-3.038035	1.370859	0.203789
C	-4.977838	0.973414	-0.587879
C	-5.720511	0.477808	-1.669987
C	-5.111375	0.521351	-2.938163
H	-5.674287	0.198349	-3.817995
C	-6.894053	-0.421546	-1.409433
H	-7.923873	-0.057484	-1.507979
C	-4.462217	-1.915819	0.156852
C	-5.247567	-2.195141	-0.974600
C	-4.571426	-2.565038	-2.151556
C	-3.196762	-2.356414	-2.273102
C	-2.470766	-1.747202	-1.230592
C	-3.091415	-1.683262	0.026731
C	-6.665528	-1.709629	-1.059915
H	-7.512897	-2.380474	-0.872811
H	-0.653329	-1.452307	-2.367255
H	-1.020907	0.717952	-2.958292
C	-0.044183	2.919134	-1.983935
C	-0.515670	4.246426	-1.876050
C	0.887847	2.635705	-3.006869
C	-0.057757	5.253461	-2.727152
H	-1.251211	4.489690	-1.108366
C	1.332087	3.637424	-3.868174
H	1.260212	1.618495	-3.125727
C	0.869007	4.952453	-3.729175
H	-0.432996	6.273428	-2.612605
H	2.052297	3.391089	-4.652413
H	1.226053	5.735789	-4.402550
H	-1.355953	2.390245	-0.438816
H	-2.719729	-2.500048	-3.246945
H	-4.945403	-1.684947	1.109675
H	-2.537964	-1.300288	0.882276
H	-5.147132	-2.852003	-3.035707
H	-3.264294	0.631885	-4.039305
H	-5.425751	1.001083	0.408282

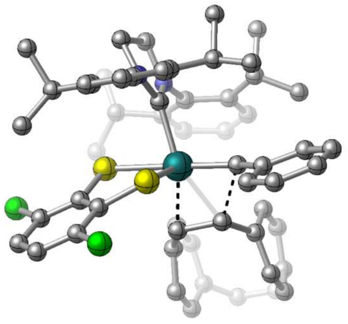

Zero-point correction= 0.996409 (Hartree/Particle)

Thermal correction to Energy= 1.055509

Thermal correction to Enthalpy= 1.056453

Thermal correction to Gibbs Free Energy= 0.905794

Sum of electronic and zero-point Energies= -4087.148520

Sum of electronic and thermal Energies= -4087.089420

Sum of electronic and thermal Enthalpies= -4087.088476

Sum of electronic and thermal Free Energies= -4087.239135

TS1h

E(scf) = -4088.14492906 a.u.

ν_{min} = -237.88 cm^{-1}

Ru	0.084206	0.149579	-0.274041	C	-5.509653	1.410980	-0.817055
C	-0.980652	0.561024	1.536545	H	-4.679427	3.375147	-1.038265
C	-2.559930	0.494907	3.262311	H	-6.142503	-0.602579	-0.412935
C	-1.189414	0.746372	3.879166	C	1.269768	-0.944406	-1.615625
H	-2.904241	-0.527572	3.468756	C	1.498338	0.515873	-1.994985
H	-0.942535	0.022417	4.663535	H	0.653349	-1.452159	-2.367284
C	0.558091	1.918510	-1.026424	H	1.020765	0.718169	-2.958214
H	-3.333796	1.197921	3.597519	S	-1.714762	-0.268215	-1.682152
H	-1.089958	1.756430	4.303196	C	-1.834738	-2.035134	-1.743608
N	-2.296325	0.648784	1.815890	C	-2.518264	-2.666650	-2.798629
N	-0.299150	0.593338	2.712611	C	-1.321932	-2.830016	-0.682056
C	1.129872	0.586970	2.873488	C	-2.729175	-4.047435	-2.821737
C	1.770353	-0.591579	3.336923	Cl	-3.167634	-1.707095	-4.123635
C	1.866034	1.783820	2.650816	S	-0.464356	-2.084042	0.665035
C	3.166157	-0.573242	3.481075	C	-1.557023	-4.217861	-0.721499
C	3.259347	1.739351	2.806769	C	-2.247499	-4.827709	-1.771972
C	3.907981	0.569913	3.199755	H	-3.267445	-4.500024	-3.655488
H	3.679190	-1.474397	3.820951	Cl	-1.007397	-5.241127	0.600651
H	3.852015	2.636377	2.625940	H	-2.402424	-5.907350	-1.762398
C	-3.380055	0.892975	0.904118	H	4.995583	0.557830	3.305885
C	-3.495452	2.180812	0.320051	H	-6.337241	1.611739	-1.502362
C	-4.334374	-0.121479	0.663284	C	1.187943	3.122905	2.364823
C	-4.567434	2.405433	-0.554304	H	0.271435	2.927240	1.790775
C	-5.397937	0.167421	-0.203483	C	1.005585	-1.838060	3.771221

H	-0.015875	-1.766733	3.372525
C	-4.263863	-1.496385	1.319931
H	-3.225663	-1.653142	1.649770
C	-2.510297	3.306752	0.641495
H	-1.498919	2.882608	0.553267
C	1.606932	-3.147552	3.234888
H	0.939767	-3.988075	3.481552
H	1.717269	-3.125679	2.143460
H	2.588627	-3.361813	3.687301
C	0.928102	-1.893067	5.311220
H	0.507791	-0.972512	5.744945
H	0.303406	-2.741280	5.634276
H	1.932574	-2.027698	5.744868
C	0.786133	3.811170	3.685945
H	1.683052	4.036863	4.285165
H	0.262965	4.758974	3.482506
H	0.126398	3.187853	4.302184
C	2.053335	4.102900	1.555937
H	2.511916	3.638392	0.671747
H	1.436977	4.950534	1.220105
H	2.869539	4.520113	2.166131
C	-2.579377	4.492818	-0.326803
H	-1.756441	5.191303	-0.114419
H	-2.483559	4.178896	-1.374075
H	-3.522676	5.052452	-0.216329
C	-2.685825	3.828712	2.079966
H	-1.961053	4.632230	2.286607
H	-3.698475	4.240821	2.219717
H	-2.533855	3.049832	2.835825
C	-5.208678	-1.565869	2.537799
H	-6.257418	-1.479353	2.208814
H	-5.097450	-2.530010	3.060572
H	-5.030130	-0.759626	3.263856
C	-4.598432	-2.650504	0.359785
H	-4.059539	-2.559024	-0.589493
H	-4.313138	-3.610335	0.817748
H	-5.677061	-2.698390	0.138806
C	5.111230	0.521807	-2.938230
C	5.720411	0.478239	-1.670076
C	4.977745	0.973742	-0.587918
C	3.605070	1.192583	-0.708411
C	2.943399	0.950945	-1.919475
C	3.740601	0.763799	-3.063800
C	6.894020	-0.421058	-1.409624
H	7.923813	-0.056923	-1.508187
C	3.091539	-1.683093	0.026616
C	2.470835	-1.747003	-1.230681
C	3.196824	-2.356123	-2.273253
H	2.719757	-2.499734	-3.247084
C	4.571506	-2.564673	-2.151779
C	5.247677	-2.194797	-0.974834
C	4.462360	-1.915576	0.156664
H	4.945575	-1.684716	1.109475
C	6.665599	-1.709179	-1.060185
H	7.513025	-2.379975	-0.873166
H	5.674132	0.198885	-3.818098
H	3.037937	1.371017	0.203839
H	1.355748	2.390370	-0.438749
C	0.043863	2.919209	-1.983776
C	0.515298	4.246524	-1.875901
C	-0.888209	2.635751	-3.006664
C	0.057313	5.253542	-2.726984
H	1.250862	4.489818	-1.108249
C	-1.332517	3.637454	-3.867952
H	-1.260538	1.618526	-3.125506
C	-0.869476	4.952497	-3.728975
H	0.432515	6.273523	-2.612450
H	-2.052754	3.391093	-4.652159

H	-1.226576	5.735820	-4.402336	H	5.425689	1.001378	0.408231
H	2.538111	-1.300194	0.882207	H	3.264106	0.632286	-4.039302
H	5.147188	-2.851558	-3.035972				

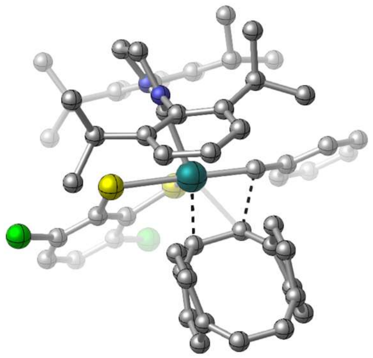

Zero-point correction= 0.996410 (Hartree/Particle)

Thermal correction to Energy= 1.055510

Thermal correction to Enthalpy= 1.056454

Thermal correction to Gibbs Free Energy= 0.905794

Sum of electronic and zero-point Energies= -4087.148519

Sum of electronic and thermal Energies= -4087.089419

Sum of electronic and thermal Enthalpies= -4087.088475

Sum of electronic and thermal Free Energies= -4087.239135

M0

E(scf) = -616.421539968 a.u.

ν_{min} = 76.81 cm^{-1}

C	-2.721799	0.677211	-0.000178	C	1.411409	1.410124	0.000047
H	-3.675968	1.217961	-0.000277	C	0.698750	1.576168	1.200161
C	-2.721786	-0.677234	0.000051	C	2.721798	0.677212	0.000032
H	-3.675922	-1.218034	-0.000081	H	3.675968	1.217961	0.000148
C	-0.698857	1.576404	1.200169	C	0.698750	-1.576578	-1.200134
C	-1.411409	1.410124	-0.000023	C	1.411375	-1.410083	-0.000048
C	-0.698750	1.576456	-1.200096	C	0.698838	-1.576307	1.200152
C	0.698856	1.576692	-1.200104	H	1.229021	-1.510586	2.153996

C	-0.698750	-1.576292	1.200198	H	-1.228855	-1.510498	2.154083
C	-1.411375	-1.410083	0.000071	H	1.228855	-1.511011	-2.154035
C	-0.698837	-1.576594	-1.200089	H	1.228979	1.511169	-2.153983
H	-1.229020	-1.511099	-2.153948	H	-1.228981	1.510652	2.154032
C	2.721787	-0.677233	-0.000197	H	1.228875	1.510544	2.154050
H	3.675922	-1.218033	-0.000048	H	-1.228874	1.511060	-2.154001

Zero-point correction= 0.225698 (Hartree/Particle)

Thermal correction to Energy= 0.236957

Thermal correction to Enthalpy= 0.237901

Thermal correction to Gibbs Free Energy= 0.189362

Sum of electronic and zero-point Energies= -616.195842

Sum of electronic and thermal Energies= -616.184583

Sum of electronic and thermal Enthalpies= -616.183639

Sum of electronic and thermal Free Energies= -616.232178

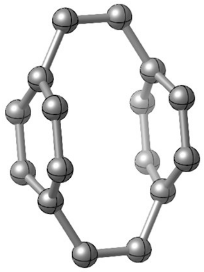

M06L-D3/def2tzvpp-SMD(benzene).

E(scf) = -616.977011457 a.u.

Ru-1-DMSO

E(scf) = -4024.76469585 a.u.

ν_{min} = 20.34 cm^{-1}

S	-1.595636	0.125507	1.035820	C	-0.057133	1.373300	-1.542612
C	1.327445	0.043994	0.714329	S	-1.921899	-1.487293	-1.740929
Ru	-0.130673	-0.312526	-0.742731	N	1.623956	1.205538	1.345945

N	1.827543	-0.971028	1.466527	C	-5.489873	-1.051203	1.127918
C	1.565269	2.551930	0.844557	C	-4.560984	-1.832594	-0.955538
C	2.386792	0.980557	2.593920	C	-5.646886	-1.720060	-0.087611
C	2.185474	-0.511765	2.819893	H	-6.325021	-0.956583	1.823249
C	1.985553	-2.348855	1.087840	H	-6.607321	-2.157147	-0.363375
H	3.072460	-1.029789	3.201815	H	0.884282	1.684909	-2.026372
H	1.345082	-0.711151	3.505901	C	-1.078645	2.407082	-1.667755
H	3.443993	1.251526	2.442072	C	-2.464525	2.180329	-1.498037
C	1.693043	5.252626	0.138471	C	-0.655561	3.721570	-1.981893
C	0.688585	3.473022	1.462821	C	-3.375913	3.229462	-1.601996
C	2.492748	2.966517	-0.148158	H	-2.822554	1.173522	-1.306944
C	2.536482	4.328377	-0.477388	C	-1.566363	4.772628	-2.062242
C	0.771250	4.822469	1.087673	H	0.408426	3.914681	-2.128617
H	3.245089	4.673786	-1.231699	C	-2.931904	4.530662	-1.868347
H	0.098208	5.546819	1.550969	H	-4.442271	3.030172	-1.470291
C	2.382605	-5.035856	0.446463	H	-1.211587	5.783815	-2.276775
C	3.196333	-2.736021	0.450733	H	-3.649722	5.352300	-1.935902
C	0.999982	-3.297358	1.443721	H	1.747222	6.309597	-0.134525
C	1.214625	-4.639624	1.091938	H	2.534915	-6.086335	0.185580
C	3.365842	-4.093140	0.141889	Cl	-4.097377	0.316250	3.005018
C	4.314641	-1.742845	0.144339	Cl	-4.807729	-2.688942	-2.469290
C	-0.227294	-2.926235	2.262100	C	0.011917	3.650877	3.897325
H	0.459690	-5.385943	1.346935	H	-0.035262	4.752158	3.884471
H	4.280067	-4.421431	-0.353516	H	-0.704726	3.293932	4.654594
H	1.986560	1.595417	3.408497	H	1.024843	3.370218	4.227742
H	3.842735	-0.774882	-0.061191	C	-1.760063	3.456326	2.099822
H	-0.316245	-1.833079	2.261130	H	-1.991969	3.087347	1.092149
C	-0.332042	3.063879	2.516510	H	-2.492798	3.009670	2.789456
H	-0.312235	1.968665	2.596496	H	-1.897998	4.549954	2.113352
C	3.441976	1.995800	-0.841992	C	4.795517	1.908224	-0.113888
H	2.984558	0.999264	-0.826170	H	5.298146	2.889358	-0.104177
C	-3.139600	-0.599440	0.590804	H	4.686549	1.577885	0.928419
C	-4.252522	-0.499936	1.453970	H	5.457095	1.189214	-0.622421
C	-3.297519	-1.290482	-0.639584	C	3.672605	2.324764	-2.324769

H	4.300689	3.220207	-2.460076
H	4.175430	1.476389	-2.809761
H	2.723663	2.494862	-2.856786
C	5.261237	-1.579143	1.349415
H	6.051999	-0.848508	1.116079
H	4.743735	-1.228881	2.251965
H	5.745360	-2.538790	1.594436
C	5.138463	-2.108692	-1.098315
H	5.787779	-2.981323	-0.919353
H	4.490476	-2.306740	-1.960806
H	5.795696	-1.265310	-1.363171
C	-0.056279	-3.389933	3.720402
H	-0.912037	-3.057519	4.330269
H	-0.001357	-4.489452	3.783368
H	0.863549	-2.985821	4.172407
C	-1.532482	-3.465905	1.660794
H	-2.395602	-3.056669	2.207655
H	-1.633633	-3.167504	0.608074
H	-1.590014	-4.564960	1.719009
S	1.300046	-1.117955	-2.422784
O	2.760605	-0.734352	-2.446545
C	0.643720	-0.695157	-4.069564
H	-0.396860	-1.038802	-4.142791
H	0.688130	0.398550	-4.147350
H	1.289456	-1.166718	-4.822714
C	1.201623	-2.929617	-2.547809
H	1.857647	-3.240345	-3.372740
H	1.553174	-3.337329	-1.594250
H	0.154572	-3.210426	-2.721086

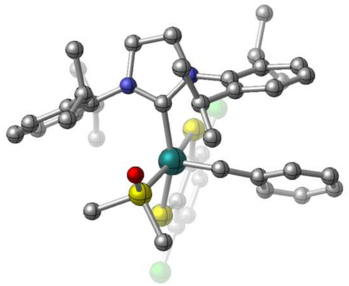

Zero-point correction= 0.849515 (Hartree/Particle)

Thermal correction to Energy= 0.903553

Thermal correction to Enthalpy= 0.904498

Thermal correction to Gibbs Free Energy= 0.764003

Sum of electronic and zero-point Energies= -4023.915181

Sum of electronic and thermal Energies= -4023.861142

Sum of electronic and thermal Enthalpies= -4023.860198

Sum of electronic and thermal Free Energies= -4024.000692

M06L-D3/def2tzvpp-SDD(Ru)-SMD(benzene).

E(scf) = -4027.05268127 a.u.

Ru-1-Pyridine

E(scf) = -4179.36646733 a.u.

ν_{min} = 22.65 cm^{-1}

S	-1.804807	-0.659695	0.951066	H	-0.441579	2.560383	3.392786
C	0.611380	1.071053	0.965143	H	3.247622	1.983400	1.001536
Ru	-0.029513	-0.253964	-0.508597	H	0.116201	-1.145985	2.797639
C	-0.824614	1.004492	-1.609239	C	-2.824076	2.628310	1.837594
S	-0.475319	-2.376102	-1.449986	H	-2.200445	1.778454	2.146344
N	0.062338	2.249463	1.355986	C	1.519573	3.699083	-0.713490
N	1.474848	0.682829	1.937630	H	1.696983	2.624217	-0.561995
C	-0.660327	3.203465	0.564774	C	-2.463177	-2.231323	0.490023
C	0.436665	2.626981	2.734331	C	-3.570647	-2.770922	1.178471
C	1.505255	1.589387	3.096190	C	-1.896837	-2.981829	-0.577646
C	2.440800	-0.375260	1.838455	C	-4.126034	-4.002600	0.837612
H	2.507873	2.028218	3.215619	C	-2.481712	-4.221762	-0.912043
H	1.265718	1.036854	4.016210	C	-3.578895	-4.733191	-0.218596
H	0.804275	3.662547	2.767019	H	-4.981158	-4.385080	1.396604
C	-1.955893	5.306685	-0.739093	H	-3.997276	-5.698999	-0.504569
C	-2.020024	3.458766	0.846737	H	-0.184043	1.808305	-2.016335
C	0.051211	3.969753	-0.396336	C	-2.198506	1.149812	-2.084798
C	-0.621982	5.019348	-1.033967	C	-3.167225	0.120683	-2.036850
C	-2.646192	4.524370	0.180715	C	-2.588462	2.400752	-2.620628
H	-0.098097	5.628218	-1.772280	C	-4.471764	0.348788	-2.472554
H	-3.697304	4.737027	0.386089	H	-2.884917	-0.862857	-1.674323
C	4.350909	-2.399726	1.647832	C	-3.898140	2.632676	-3.036116
C	3.729239	-0.067899	1.333772	H	-1.852628	3.205103	-2.675344
C	2.120355	-1.669819	2.306105	C	-4.848017	1.606574	-2.959438
C	3.092726	-2.674871	2.178691	H	-5.202609	-0.462558	-2.427692
C	4.669144	-1.102820	1.246640	H	-4.180474	3.616253	-3.420410
C	4.141885	1.350895	0.957415	H	-5.875455	1.783529	-3.288215
C	0.804769	-1.974850	3.005160	H	-2.460878	6.133859	-1.244335
H	2.861481	-3.687013	2.515951	H	5.090245	-3.197801	1.549817
H	5.663541	-0.894883	0.846583	Cl	-4.282413	-1.894620	2.530561

Cl	-1.827506	-5.185818	-2.228033
C	-3.185615	3.443679	3.092008
H	-3.849738	4.286412	2.838410
H	-3.712857	2.809838	3.823203
H	-2.293584	3.863857	3.583346
C	-4.085803	2.034881	1.188214
H	-3.837839	1.497828	0.263425
H	-4.557094	1.311756	1.871764
H	-4.826472	2.816652	0.952851
C	2.438972	4.475677	0.247032
H	2.270279	5.559913	0.146068
H	2.259027	4.207075	1.297154
H	3.498253	4.273357	0.025201
C	1.902382	4.006861	-2.168926
H	1.939761	5.089982	-2.366271
H	2.902687	3.600698	-2.385571
H	1.190913	3.563469	-2.882457
C	5.155396	1.904046	1.976874
H	5.374500	2.963782	1.768189
H	4.779174	1.828266	3.009015
H	6.104989	1.346620	1.929738
C	4.696057	1.467201	-0.469642
H	5.581302	0.830052	-0.620973
H	3.944555	1.183769	-1.215505
H	4.998645	2.505865	-0.676970
C	1.017365	-2.048384	4.528805
H	0.055349	-2.209401	5.041958
H	1.688006	-2.882061	4.795833
H	1.465272	-1.123062	4.925343
C	0.131462	-3.251184	2.481712
H	-0.885767	-3.334955	2.893267
H	0.045822	-3.232210	1.386581
H	0.686950	-4.157762	2.772423
N	1.803150	-0.326125	-1.739036
C	2.678697	-1.308141	-1.451751
C	2.014352	0.410480	-2.846568
C	3.780744	-1.577486	-2.266889
H	2.492861	-1.903077	-0.558158
C	3.095452	0.200786	-3.698773
H	1.286658	1.189905	-3.062951
C	4.005796	-0.816770	-3.414047
H	3.215211	0.830774	-4.581623
H	4.863730	-1.019967	-4.056552
Cl	4.866429	-2.868060	-1.836766

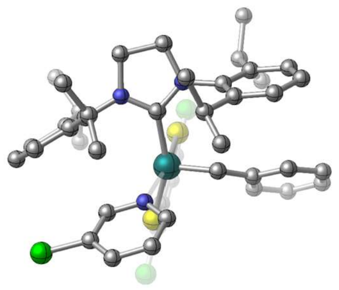

Zero-point correction= 0.848883 (Hartree/Particle)

Thermal correction to Energy= 0.903263

Thermal correction to Enthalpy= 0.904207

Thermal correction to Gibbs Free Energy= 0.761593

Sum of electronic and zero-point Energies= -4178.517584

Sum of electronic and thermal Energies= -4178.463205

Sum of electronic and thermal Enthalpies= -4178.462261

Sum of electronic and thermal Free Energies= -4178.604874

M06L-D3/def2tzvpp-SDD(Ru)-SMD(benzene).

E(scf) = -4181.73666868 a.u.

DMSO

E(scf) = -552.966900345 a.u.

ν_{min} = 168.22 cm^{-1}

S	0.000001	0.231990	-0.452589	H	1.256409	-0.891989	1.278348
O	0.000023	1.490366	0.392560	C	-1.369312	-0.808791	0.187172
C	1.369291	-0.808825	0.187170	H	-1.256418	-0.891988	1.278346
H	1.358537	-1.796762	-0.296869	H	-2.299419	-0.275775	-0.054011
H	2.299410	-0.275841	-0.054040	H	-1.358594	-1.796717	-0.296892

Zero-point correction= 0.078529 (Hartree/Particle)

Thermal correction to Energy= 0.084231

Thermal correction to Enthalpy= 0.085176

Thermal correction to Gibbs Free Energy= 0.050154

Sum of electronic and zero-point Energies= -552.888372

Sum of electronic and thermal Energies= -552.882669

Sum of electronic and thermal Enthalpies= -552.881725

Sum of electronic and thermal Free Energies= -552.916747

M06L-D3/def2tzvpp-SMD(benzene).

E(scf) = -553.265230085 a.u.

2-ClPyridine

E(scf) = -707.579174966 a.u.

ν_{min} = 190.99 cm^{-1}

N	1.538828	-1.248058	0.000126	H	-0.343930	-2.141762	0.000016
C	2.221625	-0.098844	0.000230	C	0.210819	1.222305	-0.000024
C	0.206651	-1.194883	-0.000109	H	2.205823	2.069491	0.000068
C	1.605306	1.157157	0.000060	H	-0.319992	2.176085	-0.000060
H	3.314685	-0.176023	0.000253	Cl	-2.242198	0.010933	-0.000096
C	-0.496237	0.018057	-0.000077				

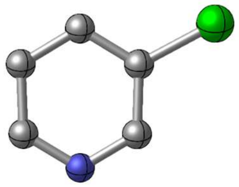

Zero-point correction= 0.079196 (Hartree/Particle)

Thermal correction to Energy= 0.084549

Thermal correction to Enthalpy= 0.085494

Thermal correction to Gibbs Free Energy= 0.049479

Sum of electronic and zero-point Energies= -707.499979

Sum of electronic and thermal Energies= -707.494626

Sum of electronic and thermal Enthalpies= -707.493681

Sum of electronic and thermal Free Energies= -707.529696

M06L-D3/def2tzvpp-SMD(benzene).

E(scf) = -707.952494559 a.u.

Ru-1

E(scf) = -3471.73675298 a.u.

ν_{min} = 7.53 cm^{-1}

S	1.108251	-0.923186	0.879760	N	-0.680872	2.256199	1.081956
C	-1.113688	1.104646	0.523454	C	-3.188365	-0.279756	0.695511
Ru	0.117641	0.188869	-0.844772	C	-2.718720	1.744023	2.111210
C	-1.017701	-1.066437	-1.564311	C	-1.657504	2.850164	2.008045
S	2.154113	0.126612	-1.994149	C	0.452876	2.991012	0.593767
N	-2.311233	0.796538	1.052572	H	-2.054168	3.787612	1.582243

H	-1.190910	3.084057	2.974148
H	-3.740301	2.106011	1.931035
C	-5.065459	-2.263963	0.150446
C	-3.115364	-1.503019	1.396760
C	-4.175339	-0.042303	-0.290118
C	-5.108534	-1.056560	-0.546407
C	-4.073472	-2.484394	1.103984
H	-5.884736	-0.896298	-1.298102
H	-4.038620	-3.439766	1.630985
C	2.650391	4.381460	-0.394188
C	1.652778	2.991038	1.339222
C	0.334257	3.682731	-0.636605
C	1.457097	4.368054	-1.116344
C	2.742798	3.704743	0.820939
H	1.404049	4.890938	-2.073254
H	3.685612	3.718260	1.370699
H	-2.702747	1.234520	3.087129
C	2.749234	-1.290440	0.325534
C	3.630334	-2.011245	1.156534
C	3.203373	-0.852786	-0.945171
C	4.926341	-2.325336	0.754962
C	4.515538	-1.193717	-1.337880
C	5.369670	-1.917078	-0.505092
H	5.581642	-2.885032	1.423707
H	6.379408	-2.153969	-0.842728
Cl	5.130140	-0.692483	-2.904497
Cl	3.102408	-2.521271	2.756226
H	3.517620	4.918724	-0.785988
H	-5.802677	-3.042766	-0.060334
C	-1.019343	-2.526876	-1.546336
C	0.146901	-3.297862	-1.337576
C	-2.243483	-3.206074	-1.740893
C	0.077975	-4.689529	-1.286302
H	1.106646	-2.794331	-1.236112
C	-2.311981	-4.596684	-1.665799
H	-3.151054	-2.624971	-1.916577
C	-1.151204	-5.344497	-1.433822
H	0.991487	-5.268259	-1.127989
H	-3.274310	-5.100074	-1.790115
H	-1.201883	-6.435128	-1.381272
H	-1.831972	-0.640518	-2.186527
C	1.779919	2.244964	2.659910
H	0.959936	1.512585	2.697557
C	-0.945258	3.638808	-1.466568
H	-1.761294	3.292197	-0.816933
C	-2.058863	-1.768284	2.460311
H	-1.306805	-0.969423	2.392872
C	-4.273793	1.282123	-1.038742
H	-3.384075	1.875335	-0.787124
C	-0.825219	2.631411	-2.621607
H	-0.619351	1.599186	-2.261146
H	-1.757684	2.570309	-3.201523
H	0.005049	2.885320	-3.296955
C	-1.363295	5.017939	-1.999894
H	-2.347586	4.952616	-2.490367
H	-1.433154	5.753697	-1.183877
H	-0.650893	5.405184	-2.744992
C	3.094630	1.455246	2.765306
H	3.958404	2.121389	2.922909
H	3.052709	0.758874	3.617056
H	3.276499	0.861952	1.859543
C	1.631082	3.207962	3.852715
H	2.448385	3.947975	3.858318
H	0.682869	3.767015	3.820134
H	1.669457	2.653856	4.804659
C	-5.508739	2.083575	-0.588411
H	-5.535218	3.067806	-1.083599
H	-6.438450	1.550440	-0.845510

H	-5.512740	2.248740	0.500238
C	-4.275294	1.098158	-2.564840
H	-5.172201	0.559725	-2.909493
H	-4.266320	2.078694	-3.067264
H	-3.394039	0.534378	-2.904783
C	-2.672934	-1.718363	3.871456
H	-3.422060	-2.516744	4.001651
H	-1.892260	-1.859295	4.636338
H	-3.175639	-0.758201	4.068591
C	-1.325902	-3.099693	2.229988
H	-0.470640	-3.183021	2.918144
H	-1.987704	-3.962683	2.408673
H	-0.935324	-3.166283	1.206157

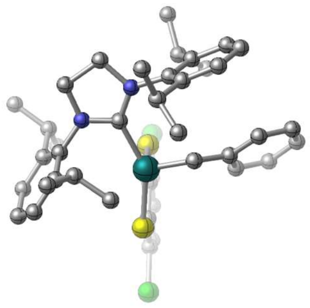

Zero-point correction= 0.764576 (Hartree/Particle)

Thermal correction to Energy= 0.813274

Thermal correction to Enthalpy= 0.814218

Thermal correction to Gibbs Free Energy= 0.680431

Sum of electronic and zero-point Energies= -3470.972177

Sum of electronic and thermal Energies= -3470.923479

Sum of electronic and thermal Enthalpies= -3470.922535

Sum of electronic and thermal Free Energies= -3471.056322

M06L-D3/def2tzvpp-SDD(Ru)-SMD(benzene).

E(scf) = -3473.74683833 a.u.

M0-Ru-1

E(scf) = -4088.18150995 a.u.

ν_{min} = 12.44 cm^{-1}

S	2.241513	0.743787	0.770458
C	0.673910	-1.666385	0.763774
Ru	0.076385	0.260173	0.056803
C	-2.210445	-0.053994	0.539739
C	0.440964	-0.071953	-1.763749
S	-0.279563	2.579884	0.154985

N	1.622947	-2.479132	0.227202
N	0.479507	-2.069491	2.045442
C	1.968527	-2.675926	-1.155061
C	2.148547	-3.471229	1.189360
C	1.428794	-3.103797	2.487446
C	-0.592025	-1.700312	2.927423
H	0.895462	-3.950126	2.942885
H	2.107255	-2.680637	3.245150
H	1.920891	-4.490298	0.842729
C	2.729727	-3.313622	-3.770543
C	3.260693	-2.322078	-1.608474
C	1.060081	-3.370991	-1.998040
C	1.464411	-3.670301	-3.305665
C	3.613055	-2.647885	-2.927204
H	0.782146	-4.201907	-3.971060
H	4.602741	-2.372495	-3.296632
C	-2.726461	-1.006414	4.580717
C	-1.770992	-2.488422	2.903511
C	-0.442069	-0.619095	3.822451
C	-1.539619	-0.278183	4.628561
C	-2.831119	-2.114712	3.739885
C	-1.909537	-3.730780	2.027758
C	0.876644	0.120099	3.987259
H	-1.455590	0.567447	5.314104
H	-3.755683	-2.695963	3.729531
H	3.239360	-3.380407	1.272252
H	-1.029062	-3.785415	1.374101
H	1.531885	-0.176167	3.158876
C	4.297159	-1.644364	-0.722042
H	3.804241	-1.359990	0.216444
C	-0.302284	-3.858059	-1.514715
H	-0.579473	-3.257272	-0.639213
C	2.381223	2.491580	0.900363
C	3.592846	3.103038	1.286947
C	1.260216	3.310704	0.618404
C	3.709261	4.486193	1.399109
C	1.400756	4.711323	0.730279
C	2.605444	5.296316	1.115426
H	4.657916	4.927158	1.708129
H	2.677994	6.381725	1.195025
H	-2.053589	-0.710287	1.393289
C	-1.945224	-0.601394	-0.733467
H	-1.664227	-1.652137	-0.773603
H	-0.069861	-0.906575	-2.260274
C	1.316856	0.598125	-2.714205
C	1.967022	1.834549	-2.493598
C	1.523312	-0.042968	-3.962537
C	2.799345	2.386877	-3.464978
H	1.807757	2.366892	-1.563461
C	2.369922	0.501373	-4.924603
H	1.022713	-0.993339	-4.157148
C	3.014076	1.720214	-4.677476
H	3.289243	3.344215	-3.271318
H	2.527916	-0.022902	-5.870323
H	3.675819	2.153515	-5.431979
H	3.025078	-3.555982	-4.794449
H	-3.570101	-0.719348	5.213198
Cl	4.997648	2.116294	1.671927
Cl	0.041077	5.767362	0.386028
C	5.451959	-2.607129	-0.388094
H	6.011520	-2.885625	-1.296193
H	6.159392	-2.128795	0.308444
H	5.093692	-3.540194	0.075436
C	4.836927	-0.348675	-1.348815
H	4.016623	0.322108	-1.634245
H	5.464263	0.189168	-0.621833
H	5.448157	-0.553222	-2.242953
C	-0.235306	-5.327418	-1.058069

H	0.042041	-5.982238	-1.899820
H	0.506335	-5.478001	-0.260522
H	-1.213638	-5.659216	-0.675129
C	-1.419735	-3.678961	-2.554129
H	-1.302489	-4.367509	-3.405183
H	-2.395623	-3.896549	-2.093460
H	-1.455693	-2.653838	-2.950707
C	-1.921540	-5.006734	2.889429
H	-1.940286	-5.904337	2.250388
H	-1.034827	-5.067686	3.539579
H	-2.811134	-5.035124	3.539186
C	-3.143548	-3.684615	1.112137
H	-4.079942	-3.676257	1.691897
H	-3.137778	-2.793291	0.470847
H	-3.167954	-4.572468	0.460669
C	1.559957	-0.299550	5.301852
H	2.545881	0.184807	5.391686
H	0.956848	-0.005289	6.176787
H	1.708746	-1.390373	5.351968
C	0.723285	1.645391	3.903596
H	1.714781	2.123800	3.901876
H	0.209701	1.942253	2.978111
H	0.158167	2.048805	4.759360
C	-3.480637	2.247477	0.478490
C	-3.406249	0.862716	0.679851
C	-4.631818	0.159827	0.677350
C	-5.782761	0.734302	0.140339
C	-5.750842	2.039933	-0.382951
C	-4.642431	2.829715	-0.044276
C	-6.625059	2.392896	-1.550889
H	-7.554854	2.959109	-1.416743
C	-4.967918	-0.181482	-2.823062
C	-4.982727	1.218972	-2.959161
C	-3.749298	1.885704	-2.871886
C	-2.624001	1.243302	-2.349849
C	-2.712251	-0.085348	-1.907186
C	-3.841361	-0.827948	-2.308989
C	-6.258564	1.991476	-2.790925
H	-6.900122	2.223830	-3.649665
H	-6.658977	0.103902	-0.033478
H	-2.586126	2.859158	0.568354
H	-4.637902	-0.906746	0.918062
H	-4.603918	3.874444	-0.364741
H	-5.895118	-0.747111	-2.949424
H	-1.729925	1.827261	-2.125052
H	-3.912263	-1.886219	-2.044112
H	-3.710661	2.965312	-3.036693

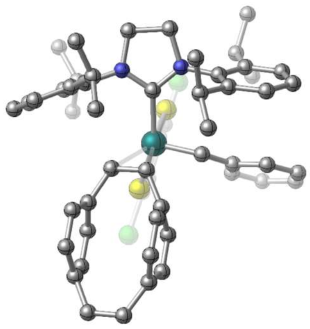

Zero-point correction= 0.995605 (Hartree/Particle)

Thermal correction to Energy= 1.056076

Thermal correction to Enthalpy= 1.057020

Thermal correction to Gibbs Free Energy= 0.901348

Sum of electronic and zero-point Energies= -4087.185905

Sum of electronic and thermal Energies= -4087.125434

Sum of electronic and thermal Enthalpies= -4087.124490

Sum of electronic and thermal Free Energies= -4087.280162

M06L-D3/def2tzvpp-SDD(Ru)-SMD(benzene).

E(scf) = -4090.74207844 a.u.

Int1

E(scf) = -4088.21894347 a.u.

ν_{min} = 17.16 cm^{-1}

S	-0.110801	-0.405708	2.296970	C	-3.812987	-3.565978	-0.280420
C	-1.935704	0.773187	0.303935	H	-4.419850	-2.334217	-3.384425
Ru	-0.083953	-0.166708	0.011883	H	-3.835901	-4.511702	0.265267
C	0.810166	1.468044	-0.140000	C	-0.072258	5.619637	0.780984
C	-0.353027	-0.992029	-2.496954	C	-1.526304	4.002157	-0.312314
S	1.551504	-1.886193	0.023019	C	-0.745111	3.614371	1.993370
N	-3.088118	0.045510	0.330424	C	-0.048987	4.831360	1.928339
N	-2.221420	1.973021	0.889537	C	-0.814901	5.209232	-0.325789
C	-3.369277	-1.190802	-0.343700	C	-2.357684	3.623825	-1.532004
C	-4.115356	0.644042	1.201207	C	-0.741167	2.826995	3.293963
C	-3.633874	2.086861	1.300876	H	0.518993	5.166566	2.798406
C	-1.458448	3.191155	0.849121	H	-0.847330	5.841628	-1.215812
H	-4.172642	2.763008	0.615724	H	-4.111426	0.140402	2.182088
H	-3.707375	2.498210	2.316448	H	-2.734292	2.605083	-1.382312
H	-5.118116	0.538950	0.766609	H	-1.198361	1.850275	3.091751
C	-4.189226	-3.536274	-1.618899	C	-2.959045	-2.490759	1.838818
C	-3.387064	-2.402163	0.381638	H	-2.571427	-1.508762	2.136376
C	-3.742555	-1.142969	-1.714794	C	-3.733365	0.167613	-2.502583
C	-4.139600	-2.336106	-2.330730	H	-2.885996	0.762120	-2.129086

C	1.149391	-1.564809	2.731634
C	1.416959	-1.865733	4.081455
C	1.892100	-2.221712	1.718470
C	2.400523	-2.783552	4.447389
C	2.890530	-3.139522	2.110740
C	3.145820	-3.421461	3.452768
H	2.581067	-2.994347	5.502279
H	3.924659	-4.138553	3.715524
H	0.316569	2.237304	-0.751262
C	0.379684	0.154927	-2.705366
H	-0.217904	1.068532	-2.724687
H	-1.413216	-0.834418	-2.311483
C	-0.028485	-2.404067	-2.795997
C	-0.681403	-3.423657	-2.078585
C	0.821844	-2.771671	-3.854173
C	-0.462449	-4.766208	-2.379544
H	-1.350754	-3.152505	-1.263474
C	1.041903	-4.117881	-4.158187
H	1.306433	-1.997583	-4.451377
C	0.408283	-5.120935	-3.417422
H	-0.972548	-5.539235	-1.799163
H	1.708453	-4.383200	-4.982897
H	0.584908	-6.173478	-3.653270
H	-4.508485	-4.453474	-2.119726
H	0.481528	6.561405	0.750988
Cl	0.481660	-1.095235	5.360003
Cl	3.853662	-3.968352	0.896410
C	-4.143700	-2.838091	2.755700
H	-4.548115	-3.838360	2.528854
H	-3.824453	-2.836858	3.810431
H	-4.967574	-2.114429	2.646851
C	-1.796401	-3.480438	2.024261
H	-0.954145	-3.228705	1.363112
H	-1.425955	-3.436999	3.060227

H	-2.105674	-4.517657	1.816615
C	-5.013122	0.986310	-2.254792
H	-5.901418	0.420055	-2.577859
H	-5.143808	1.242820	-1.195883
H	-4.983291	1.928857	-2.823536
C	-3.519357	-0.012611	-4.012663
H	-4.400872	-0.456981	-4.501378
H	-3.349927	0.969577	-4.481220
H	-2.650207	-0.649135	-4.235056
C	-3.577307	4.550776	-1.683333
H	-4.193551	4.243190	-2.543319
H	-4.213638	4.536737	-0.785084
H	-3.261284	5.593007	-1.850938
C	-1.526978	3.612792	-2.825280
H	-1.240766	4.630505	-3.134103
H	-0.600496	3.035866	-2.699592
H	-2.101876	3.163671	-3.650682
C	-1.594239	3.533444	4.363453
H	-1.629464	2.929646	5.284771
H	-1.173368	4.519299	4.621902
H	-2.629382	3.694779	4.021742
C	0.681736	2.563870	3.811324
H	0.653856	1.845662	4.644531
H	1.310583	2.127933	3.022496
H	1.161014	3.489089	4.171710
C	3.155914	1.040522	0.708728
C	2.152140	1.897718	0.195175
C	2.569923	3.143204	-0.339557
C	3.906852	3.382043	-0.626260
C	4.858082	2.367116	-0.411852
C	4.491354	1.268008	0.388998
C	6.050777	2.284723	-1.309926
H	7.037657	2.637833	-0.988814
C	3.531345	2.078121	-3.421406

C	4.537975	1.219637	-2.950002	H	2.870724	0.126360	1.223277
C	4.173719	-0.108081	-2.660414	H	1.810390	3.861076	-0.659379
C	2.843867	-0.518428	-2.691431	H	5.235640	0.504716	0.629825
C	1.808432	0.391673	-2.991355	H	3.781988	3.108949	-3.681338
C	2.194772	1.676596	-3.425838	H	2.608147	-1.530499	-2.373594
C	5.880653	1.742664	-2.533457	H	4.934395	-0.809955	-2.309522
H	6.736343	1.650425	-3.213647	H	1.426586	2.405192	-3.698003
H	4.191815	4.279000	-1.181206				

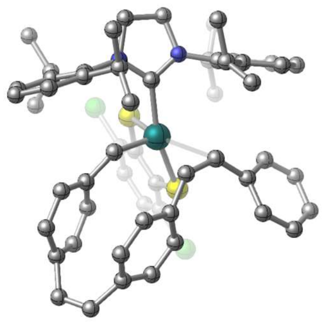

Zero-point correction= 0.995936 (Hartree/Particle)

Thermal correction to Energy= 1.056691

Thermal correction to Enthalpy= 1.057635

Thermal correction to Gibbs Free Energy= 0.901680

Sum of electronic and zero-point Energies= -4087.223007

Sum of electronic and thermal Energies= -4087.162252

Sum of electronic and thermal Enthalpies= -4087.161308

Sum of electronic and thermal Free Energies= -4087.317263

M06L-D3/def2tzvpp-SDD(Ru)-SMD(benzene).

E(scf) = -4090.77500924 a.u.

Int2

E(scf) = -4088.24922199 a.u.

ν_{min} = 11.58 cm^{-1}

S	-0.256745	-0.523903	1.044587	C	-3.162252	0.072477	0.537195

Ru	-1.668627	-0.538222	-0.750008
C	-1.264265	0.949592	-1.735353
S	-0.373755	-2.266868	-1.626001
N	-4.022897	-0.858103	0.996220
N	-3.422161	1.241270	1.155154
C	-4.166291	-2.161187	0.411378
C	-5.016714	-0.310296	1.931666
C	-4.457848	1.093746	2.202517
C	-2.819403	2.511372	0.866506
H	-5.210428	1.889811	2.122634
H	-3.996645	1.159847	3.198025
H	-6.011150	-0.293351	1.453718
C	-4.480648	-4.683769	-0.716093
C	-3.532816	-3.265657	1.022618
C	-4.949539	-2.296705	-0.759518
C	-5.090883	-3.577599	-1.308840
C	-3.710831	-4.526748	0.435651
H	-5.685062	-3.712268	-2.215720
H	-3.230826	-5.399161	0.883192
C	-1.740960	5.024225	0.334840
C	-3.204817	3.190445	-0.317765
C	-1.894789	3.068808	1.776768
C	-1.375363	4.340701	1.489872
C	-2.641414	4.448771	-0.562117
C	-4.214074	2.589034	-1.294256
C	-1.474538	2.360255	3.060758
H	-0.659470	4.794628	2.177537
H	-2.904763	4.993272	-1.469209
H	-5.082604	-0.926576	2.838954
H	-4.035825	1.506088	-1.317729
H	-1.774630	1.304712	2.975509
C	-2.680569	-3.113292	2.274757
H	-2.474429	-2.041359	2.404716
C	-5.623654	-1.105334	-1.430568

H	-5.361503	-0.200466	-0.867045
C	1.165848	-1.438541	0.522735
C	2.358010	-1.412721	1.272474
C	1.118803	-2.215058	-0.663301
C	3.481076	-2.139012	0.886354
C	2.268648	-2.940540	-1.037622
C	3.438673	-2.905995	-0.279558
H	4.390715	-2.084926	1.482129
H	4.319422	-3.458011	-0.607291
H	-4.601551	-5.675286	-1.159687
H	-1.312991	6.007000	0.122388
Cl	2.471682	-0.420942	2.721408
Cl	2.266686	-3.914459	-2.499854
C	-3.440344	-3.606840	3.518921
H	-3.664489	-4.683671	3.441643
H	-2.836994	-3.451171	4.428055
H	-4.397978	-3.077631	3.649506
C	-1.319215	-3.813505	2.139320
H	-0.806620	-3.501602	1.218367
H	-0.673609	-3.550735	2.992009
H	-1.423334	-4.910563	2.126613
C	-7.156368	-1.228327	-1.395670
H	-7.502382	-2.099160	-1.975470
H	-7.525309	-1.345337	-0.364316
H	-7.625085	-0.329043	-1.827295
C	-5.115822	-0.894592	-2.866624
H	-5.395165	-1.735248	-3.521394
H	-5.547380	0.023444	-3.296544
H	-4.019358	-0.801176	-2.897955
C	-5.661607	2.808505	-0.819091
H	-6.372362	2.326704	-1.510167
H	-5.835133	2.389703	0.181702
H	-5.899970	3.883809	-0.778117
C	-4.050093	3.085588	-2.736570

H	-4.360394	4.136746	-2.849810
H	-3.007943	3.002020	-3.078393
H	-4.681613	2.486054	-3.410847
C	-2.191805	2.981517	4.275844
H	-1.960030	2.419184	5.194858
H	-1.862462	4.023021	4.424562
H	-3.285277	3.000264	4.149984
C	0.045579	2.366802	3.288658
H	0.289431	1.779992	4.188208
H	0.586215	1.910382	2.449808
H	0.432031	3.386170	3.447165
C	-0.251198	1.990469	-1.753925
C	0.507425	2.336294	-0.613682
C	0.044067	2.660083	-2.964449
C	1.555869	3.235008	-0.695626
H	0.268956	1.859378	0.332401
C	1.101352	3.563587	-3.046108
H	-0.546489	2.438219	-3.857746
C	1.913769	3.828692	-1.925172
H	2.130189	3.470443	0.201564
H	1.331926	4.042739	-4.001560
C	3.091837	4.700805	-2.050268
H	2.973974	5.552197	-2.731136
C	4.293409	4.574257	-1.438671
H	5.006872	5.390673	-1.603024
C	4.808872	3.483989	-0.588685
C	4.468346	2.125923	-0.776628
C	5.720079	3.787929	0.441243
C	4.996843	1.128071	0.034777
C	6.236892	2.789886	1.265679
H	6.014270	4.828283	0.606441
C	5.904944	1.434957	1.070339
H	4.696478	0.092409	-0.129030
C	6.476324	0.424228	1.978275
H	6.599036	0.782110	3.007611
C	6.876043	-0.851207	1.762062
H	7.194996	-1.394920	2.659016
C	6.974078	-1.653016	0.529330
C	7.146633	-1.100679	-0.756711
C	6.938873	-3.058751	0.641841
C	7.233089	-1.920014	-1.882418
H	7.221286	-0.019392	-0.873611
C	7.021075	-3.879619	-0.485021
H	6.829249	-3.511479	1.631341
C	7.159942	-3.312789	-1.755846
H	7.362436	-1.467578	-2.868990
H	6.974913	-4.965527	-0.369999
H	7.219810	-3.950555	-2.641093
H	-1.853612	0.886308	-2.683758
H	3.777385	1.848225	-1.573403
H	6.926304	3.060786	2.070300

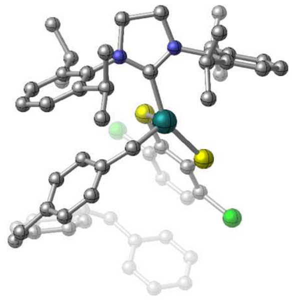

Zero-point correction= 0.995829 (Hartree/Particle)

Thermal correction to Energy= 1.057278

Thermal correction to Enthalpy= 1.058222

Thermal correction to Gibbs Free Energy= 0.897108

Sum of electronic and zero-point Energies= -4087.253393

Sum of electronic and thermal Energies= -4087.191944

Sum of electronic and thermal Enthalpies= -4087.191000

Sum of electronic and thermal Free Energies= -4087.352114

M06L-D3/def2tzvpp-SDD(Ru)-SMD(benzene).

E(scf) = -4090.81104685 a.u.

Int2-M0

E(scf) = -4704.69298800 a.u.

ν_{min} = 3.92 cm^{-1}

S	0.480037	-1.810867	1.583425
C	3.035129	-1.079675	0.477154
Ru	1.186451	-0.030071	0.255953
C	0.812458	-0.643410	-1.496579
S	-0.884570	0.950837	0.801310
N	3.911051	-0.871267	1.493776
N	3.346911	-2.281211	-0.079449
C	4.175570	0.357281	2.188281
C	4.755363	-2.042295	1.782936
C	4.495266	-2.941314	0.575038
C	2.881224	-2.821046	-1.328599
H	5.349197	-2.985655	-0.117896
H	4.238719	-3.968843	0.860609
H	5.808611	-1.748123	1.886470
C	4.717769	2.775064	3.467617
C	3.486777	0.672474	3.379051
C	5.181381	1.209045	1.663525
C	5.431401	2.419623	2.322625
C	3.765389	1.904228	3.992252
H	6.191579	3.098918	1.930800
H	3.230198	2.179028	4.903524
C	2.044775	-3.902736	-3.766383
C	3.358959	-2.258434	-2.542856
C	2.041714	-3.959855	-1.330253
C	1.627633	-4.473971	-2.568403
C	2.914460	-2.813159	-3.749610
C	4.370229	-1.115943	-2.575543
C	1.627222	-4.687726	-0.057447
H	0.964693	-5.340777	-2.590747
H	3.255162	-2.388545	-4.695220
H	4.432343	-2.500656	2.732184
H	4.299558	-0.581620	-1.619260
H	1.861816	-4.036957	0.795383
C	2.511619	-0.292528	4.033767
H	2.333651	-1.115656	3.331183
C	5.999675	0.846296	0.427919

H	5.569270	-0.065333	-0.007359
C	-1.163383	-1.461320	2.119165
C	-1.902325	-2.388578	2.884027
C	-1.776044	-0.238617	1.746566
C	-3.219335	-2.138815	3.263799
C	-3.108029	-0.001144	2.152387
C	-3.828816	-0.937304	2.889316
H	-3.764101	-2.879845	3.850181
H	-4.863576	-0.727420	3.162365
H	4.914492	3.731129	3.958972
H	1.700029	-4.312841	-4.718838
Cl	-1.162441	-3.898246	3.402423
Cl	-3.903948	1.508803	1.733381
C	3.126081	-0.893074	5.310993
H	3.311277	-0.116258	6.071302
H	2.443259	-1.639033	5.749148
H	4.086884	-1.390584	5.101027
C	1.144609	0.347504	4.318274
H	0.714930	0.790965	3.407974
H	0.438665	-0.417272	4.677329
H	1.212180	1.132245	5.089015
C	7.458200	0.530808	0.806210
H	7.960463	1.422467	1.215076
H	7.520070	-0.262151	1.567749
H	8.024090	0.199785	-0.079601
C	5.945526	1.930309	-0.661157
H	6.433370	2.861463	-0.332377
H	6.468833	1.586105	-1.567484
H	4.910651	2.173867	-0.936892
C	5.808575	-1.655542	-2.694509
H	6.536363	-0.828683	-2.657077
H	6.055217	-2.359286	-1.886817
H	5.943514	-2.186803	-3.650533
C	4.108981	-0.087738	-3.687411

H	4.321460	-0.501600	-4.685331
H	3.071157	0.274805	-3.685926
H	4.767769	0.783766	-3.551448
C	2.425252	-5.997799	0.094244
H	2.178663	-6.489946	1.049029
H	2.182563	-6.700104	-0.720166
H	3.513378	-5.829382	0.066363
C	0.119514	-4.975624	0.002225
H	-0.154155	-5.353432	0.998508
H	-0.463756	-4.064026	-0.175777
H	-0.181461	-5.731959	-0.740741
C	-0.360288	-1.291208	-2.049221
C	-1.430484	-1.821895	-1.292006
C	-0.468497	-1.369090	-3.462051
C	-2.547438	-2.365591	-1.901571
H	-1.368480	-1.825041	-0.211562
C	-1.604317	-1.887537	-4.073680
H	0.354055	-0.997365	-4.077414
C	-2.689448	-2.365900	-3.306710
H	-3.334169	-2.787908	-1.277636
H	-1.668382	-1.910419	-5.164960
C	-3.893628	-2.871929	-3.981036
H	-3.717682	-3.282273	-4.982200
C	-5.172079	-2.906979	-3.532686
H	-5.894671	-3.431299	-4.169787
C	-5.738629	-2.337671	-2.295684
C	-5.350357	-1.077280	-1.793586
C	-6.703534	-3.061221	-1.570318
C	-5.863932	-0.585623	-0.600290
C	-7.207669	-2.575517	-0.363926
H	-7.036874	-4.034172	-1.942148
C	-6.805652	-1.325699	0.147089
H	-5.511283	0.373438	-0.222378
C	-7.320453	-0.881724	1.454417

H	-7.536003	-1.704411	2.146678
C	-7.538208	0.363476	1.937280
H	-7.821090	0.420765	2.995132
C	-7.471814	1.672638	1.261840
C	-7.855149	1.856423	-0.081515
C	-7.058763	2.802500	1.994864
C	-7.791710	3.116527	-0.677434
H	-8.209041	1.000921	-0.659276
C	-6.986422	4.062238	1.397007
H	-6.768400	2.681330	3.041676
C	-7.348848	4.224273	0.055672
H	-8.094254	3.236544	-1.721038
H	-6.646432	4.921388	1.981056
H	-7.295792	5.209697	-0.414115
C	2.614122	4.467401	-0.018167
C	1.672707	3.494912	0.386826
C	0.355077	3.936669	0.564388
H	-0.393570	3.259275	0.969123
C	-0.062988	5.162400	0.030641
C	0.825535	5.968771	-0.695780
C	2.196372	5.679512	-0.565247
H	2.933011	6.323761	-1.052841
C	2.181247	2.081572	0.207879
C	0.349139	6.797861	-1.852828
H	0.093745	7.857322	-1.728237
C	-0.290850	3.788837	-2.758526
C	0.595025	4.772155	-3.229303
C	1.922134	4.375684	-3.478019
H	2.628998	5.091094	-3.907134
C	2.401364	3.163203	-2.976646
C	1.567619	2.329236	-2.203572
C	0.187577	2.578975	-2.249664
H	-0.499968	1.908249	-1.731860
C	0.245045	6.223501	-3.074457
H	-0.081338	6.824465	-3.932005
C	2.219388	1.555443	-1.103688
H	-1.132461	5.390095	0.020798
H	3.671013	4.194178	-0.084405
H	1.523997	-0.426781	-2.300392
H	3.106722	0.990551	-1.384677
H	3.024359	1.834880	0.850018
H	-7.930205	-3.175265	0.196720
H	-4.614555	-0.485758	-2.341058
H	-1.344713	4.040254	-2.615446
H	3.473933	2.956262	-3.025440

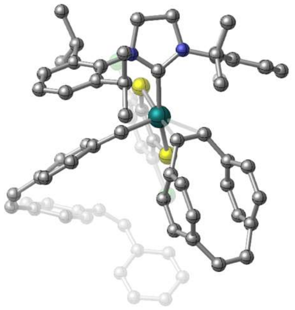

Zero-point correction= 1.224937 (Hartree/Particle)

Thermal correction to Energy= 1.298856

Thermal correction to Enthalpy= 1.299801

Thermal correction to Gibbs Free Energy= 1.112389

Sum of electronic and zero-point Energies= -4703.468051

Sum of electronic and thermal Energies= -4703.394132

Sum of electronic and thermal Enthalpies= -4703.393187

Sum of electronic and thermal Free Energies= -4703.580599

M06L-D3/def2tzvpp-SDD(Ru)-SMD(benzene).

E(scf) = -4707.80332538 a.u.

TS2

E(scf) = -4704.68505082 a.u.

ν_{min} = -193.11 cm^{-1}

S	-0.308997	-1.698201	-1.602242	C	-2.227051	-4.433653	3.440878
C	-2.958544	-1.269603	-0.608002	C	-3.481559	-2.756954	2.197855
Ru	-1.203935	-0.118428	-0.212939	C	-1.881435	-4.233624	1.035892
C	-1.065956	-0.592196	1.637876	C	-1.602394	-4.845937	2.269201
S	0.880887	1.031420	-0.503558	C	-3.161289	-3.398018	3.400688
N	-3.788820	-1.008882	-1.651537	C	-4.541794	-1.656996	2.188736
N	-3.180690	-2.559130	-0.233870	C	-1.189417	-4.760282	-0.213793
C	-4.089802	0.272580	-2.232325	H	-0.877221	-5.661162	2.306608
C	-4.571730	-2.176946	-2.091328	H	-3.648528	-3.087205	4.325579
C	-4.080813	-3.284674	-1.153506	H	-4.358988	-2.389945	-3.149596
C	-2.817415	-3.176281	1.012260	H	-4.284990	-0.956454	1.381367
H	-4.890582	-3.776906	-0.596451	H	-1.388632	-4.057189	-1.032045
H	-3.520504	-4.062807	-1.691694	C	-2.285290	-0.088898	-4.025670
H	-5.650132	-1.976705	-1.997626	H	-2.106269	-0.977140	-3.407900
C	-4.696481	2.780537	-3.295770	C	-6.073229	0.462215	-0.588267
C	-3.349190	0.749365	-3.334530	H	-5.651149	-0.492967	-0.253820
C	-5.178258	1.008780	-1.698603	C	1.369029	-1.260851	-1.945868
C	-5.456448	2.268063	-2.244430	C	2.198967	-2.105687	-2.710379
C	-3.664484	2.020862	-3.839927	C	1.896154	-0.050651	-1.432014
H	-6.280267	2.859843	-1.838999	C	3.533233	-1.789178	-2.955317
H	-3.092297	2.416407	-4.681711	C	3.247215	0.260150	-1.712966

C	4.060411	-0.596364	-2.449002
H	4.154808	-2.467560	-3.540792
H	5.101945	-0.330125	-2.628061
H	-4.918725	3.771736	-3.698576
H	-1.989960	-4.919735	4.390506
Cl	1.556765	-3.591628	-3.402323
Cl	3.941700	1.765454	-1.128052
C	-2.789023	-0.572335	-5.397808
H	-2.971206	0.274773	-6.079563
H	-2.041757	-1.231021	-5.869555
H	-3.732170	-1.135311	-5.306591
C	-0.941792	0.644766	-4.153246
H	-0.591358	1.008925	-3.176528
H	-0.173650	-0.039430	-4.546112
H	-1.009497	1.504288	-4.839421
C	-7.488585	0.176706	-1.122991
H	-7.995674	1.109342	-1.418378
H	-7.462695	-0.479767	-2.006751
H	-8.103439	-0.311908	-0.349796
C	-6.137886	1.373450	0.648483
H	-6.527035	2.372948	0.399110
H	-6.807430	0.939647	1.407513
H	-5.149289	1.503185	1.110938
C	-5.930839	-2.241633	1.872873
H	-6.694929	-1.449012	1.854760
H	-5.951297	-2.749881	0.898940
H	-6.220030	-2.977309	2.640388
C	-4.598360	-0.836531	3.486702
H	-5.034175	-1.411195	4.318840
H	-3.601162	-0.495106	3.804059
H	-5.230957	0.051822	3.338223
C	-1.752307	-6.136793	-0.612680
H	-1.290059	-6.483480	-1.551123
H	-1.543447	-6.890219	0.164634

H	-2.843812	-6.107221	-0.758723
C	0.337608	-4.820805	-0.050667
H	0.811568	-5.057709	-1.014554
H	0.734790	-3.852795	0.284571
H	0.642242	-5.590341	0.676739
C	0.190906	-0.712870	2.406329
C	1.335077	-1.306636	1.837261
C	0.246600	-0.338798	3.762786
C	2.492100	-1.494566	2.581094
H	1.299410	-1.648581	0.804672
C	1.426067	-0.479445	4.491056
H	-0.635426	0.094481	4.239580
C	2.586465	-1.038685	3.912786
H	3.338370	-1.998503	2.116088
H	1.454670	-0.149423	5.533218
C	3.795445	-1.174496	4.739297
H	3.590766	-1.170528	5.816322
C	5.099827	-1.325227	4.401607
H	5.779992	-1.511431	5.241721
C	5.775755	-1.291403	3.092938
C	5.392003	-0.438059	2.036007
C	6.891427	-2.125379	2.878976
C	6.061927	-0.449764	0.818930
C	7.554812	-2.146350	1.653438
H	7.227626	-2.785437	3.683631
C	7.162971	-1.303573	0.593654
H	5.713858	0.208968	0.026737
C	7.866097	-1.405275	-0.695771
H	8.333855	-2.383446	-0.859402
C	8.006914	-0.522511	-1.713358
H	8.496799	-0.915909	-2.612255
C	7.618835	0.896869	-1.820978
C	7.690918	1.793806	-0.736827
C	7.208221	1.401123	-3.071416

C	7.323237	3.130415	-0.889021
H	8.039791	1.434361	0.232716
C	6.830864	2.736892	-3.222212
H	7.171356	0.727823	-3.932633
C	6.881603	3.606713	-2.129331
H	7.382865	3.807557	-0.032971
H	6.495045	3.099413	-4.197084
H	6.585072	4.652361	-2.243271
C	-3.191270	3.951204	0.237590
C	-2.171462	3.086655	-0.217993
C	-0.975566	3.693526	-0.627569
H	-0.196150	3.088139	-1.087432
C	-0.687120	5.014346	-0.267760
C	-1.588320	5.758180	0.510534
C	-2.900643	5.262607	0.611301
H	-3.657693	5.844121	1.144341
C	-2.387631	1.631440	0.109408
C	-1.095936	6.772696	1.500669
H	-1.043235	7.838759	1.247893
C	0.205725	4.020102	2.571426
C	-0.785949	4.890867	3.053819
C	-1.986959	4.311348	3.499521
H	-2.760209	4.943121	3.944663
C	-2.292779	2.986615	3.178862
C	-1.407571	2.219285	2.396604
C	-0.099106	2.697389	2.245130
H	0.644109	2.091201	1.728307
C	-0.715901	6.355060	2.730664
H	-0.364654	7.084845	3.470261
C	-2.042934	1.192549	1.507677
H	0.322709	5.396696	-0.440014
H	-4.177187	3.543981	0.471145
H	-1.893153	-1.057339	2.180330
H	-2.915514	0.736244	1.978219
H	-3.411685	1.352686	-0.125345
H	4.547702	0.241322	2.164540
H	8.398686	-2.827632	1.511826
H	-3.299008	2.609907	3.385312
H	1.179424	4.421929	2.279914

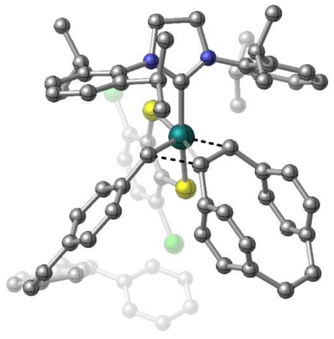

Zero-point correction= 1.225987 (Hartree/Particle)

Thermal correction to Energy= 1.298577

Thermal correction to Enthalpy= 1.299521

Thermal correction to Gibbs Free Energy= 1.118075

Sum of electronic and zero-point Energies= -4703.459064

Sum of electronic and thermal Energies= -4703.386474

Sum of electronic and thermal Enthalpies= -4703.385530

Sum of electronic and thermal Free Energies= -4703.566976

M06L-D3/def2tzvpp-SDD(Ru)-SMD(benzene).

E(scf) = -4707.79444143 a.u.

Product

E(scf) = -4704.77141076 a.u.

ν_{min} = 14.37 cm^{-1}

S	0.905242	-0.908506	-0.728337	C	-2.369127	-2.434643	-4.183134
C	-1.861162	-1.119960	0.303680	C	-3.505771	-4.008518	-2.747550
Ru	-0.368479	-2.535790	0.216122	C	-3.978428	-3.549694	-0.309856
C	5.504683	3.041176	-2.143147	C	-1.555937	-0.182284	-3.368184
S	0.790132	-4.135744	-1.039459	H	-1.969669	-2.177959	-5.166119
N	-2.191740	-0.194386	1.222399	H	-3.994188	-4.976142	-2.614449
N	-2.663889	-0.959771	-0.772260	H	-2.784933	1.724405	0.549876
C	-1.738167	-0.119255	2.577926	H	-3.656233	-2.817629	0.441325
C	-3.229834	0.734686	0.735975	H	-1.316463	0.242747	-2.382107
C	-3.706330	0.054982	-0.551099	C	0.012535	1.639514	1.900022
C	-2.756775	-1.888238	-1.864444	H	-0.334627	1.343977	0.898182
H	-4.686941	-0.435064	-0.432108	C	-3.655600	-1.716035	3.203246
H	-3.776892	0.746306	-1.398683	H	-3.836233	-1.619551	2.124532
H	-4.026213	0.855558	1.480180	C	2.067873	-1.762937	-1.751198
C	-0.950684	0.054417	5.242433	C	3.025296	-1.031460	-2.480498
C	-0.676210	0.743090	2.920716	C	2.043614	-3.176591	-1.864090
C	-2.439075	-0.866103	3.553229	C	3.980309	-1.658722	-3.275537
C	-2.015495	-0.773381	4.886022	C	3.027648	-3.793648	-2.666619
C	-0.295994	0.807352	4.269505	C	3.984734	-3.053086	-3.361956
H	-2.533995	-1.347633	5.657525	H	4.711320	-1.060665	-3.821281
H	0.530037	1.458617	4.561928	H	4.724642	-3.569674	-3.974573
C	-2.997826	-3.664744	-3.999483	H	-0.631353	0.115328	6.285963
C	-3.397040	-3.135336	-1.657096	H	-3.089220	-4.362101	-4.836019
C	-2.234664	-1.522946	-3.126405	Cl	3.002260	0.725279	-2.411882

Cl	3.069079	-5.541166	-2.830805
C	-0.389077	3.107847	2.137368
H	-0.117880	3.425669	3.157243
H	0.128438	3.777246	1.435139
H	-1.473426	3.258909	2.015154
C	1.539896	1.481524	1.911438
H	1.830691	0.434082	1.755297
H	1.985706	2.082750	1.103560
H	1.976802	1.830293	2.860956
C	-4.919708	-1.199472	3.912023
H	-4.841000	-1.302745	5.006246
H	-5.096874	-0.136749	3.684443
H	-5.803848	-1.770004	3.583970
C	-3.424046	-3.208544	3.484478
H	-3.211126	-3.390499	4.550029
H	-4.317786	-3.793875	3.215892
H	-2.578542	-3.598093	2.898483
C	-5.516612	-3.524256	-0.335347
H	-5.924211	-3.767712	0.659568
H	-5.897691	-2.532897	-0.627421
H	-5.913233	-4.259979	-1.053836
C	-3.453808	-4.918521	0.154370
H	-3.797591	-5.732069	-0.503551
H	-2.352609	-4.945621	0.164430
H	-3.812562	-5.140634	1.170832
C	-2.501014	0.780745	-4.109272
H	-2.023177	1.763614	-4.249244
H	-2.756449	0.382870	-5.105111
H	-3.442892	0.938665	-3.563396
C	-0.228543	-0.325563	-4.129879
H	0.337284	0.617784	-4.089233
H	0.401045	-1.110079	-3.690359
H	-0.394163	-0.570353	-5.191663
C	4.387694	3.802812	-1.555267
C	3.219888	4.050539	-2.306141
C	4.463671	4.365386	-0.265714
C	2.157500	4.784573	-1.780676
H	3.143890	3.649928	-3.320483
C	3.414294	5.114873	0.250556
H	5.367354	4.220812	0.328447
C	2.226699	5.331737	-0.483209
H	1.275592	4.965646	-2.397471
H	3.513063	5.558044	1.245363
C	1.208969	6.215072	0.101792
H	1.631382	6.920486	0.827155
C	-0.122972	6.357475	-0.098503
H	-0.568701	7.207252	0.431686
C	-1.124858	5.576955	-0.842140
C	-1.037443	4.192929	-1.098705
C	-2.317706	6.231341	-1.217672
C	-2.109696	3.496658	-1.657948
C	-3.376065	5.542408	-1.804665
H	-2.418416	7.303419	-1.027118
C	-3.304326	4.153903	-2.004031
H	-2.021055	2.420120	-1.811052
C	-4.474171	3.412073	-2.545794
H	-4.525981	3.332402	-3.638738
C	-5.467728	2.825790	-1.847221
H	-6.224126	2.300228	-2.441123
C	-5.684459	2.726195	-0.390265
C	-5.046496	3.538292	0.572647
C	-6.584875	1.743315	0.074668
C	-5.290670	3.359950	1.935496
H	-4.355966	4.317658	0.256835
C	-6.819452	1.556648	1.438306
H	-7.099180	1.107146	-0.651035
C	-6.170864	2.365645	2.377936
H	-4.782826	4.002001	2.659523

H	-7.510993	0.777830	1.769160
H	-6.353813	2.225933	3.446048
C	1.654297	-2.002815	3.899006
C	1.554856	-2.321978	2.526630
C	2.749038	-2.340604	1.767503
H	2.715511	-2.661594	0.728515
C	3.961672	-1.983755	2.336720
C	4.043637	-1.556425	3.678991
C	2.870632	-1.617246	4.458803
H	2.912335	-1.331618	5.513714
C	0.258356	-2.624458	1.936074
C	5.308272	-1.080508	4.267860
H	5.478125	-1.379015	5.309316
C	5.085388	0.808584	1.767738
C	6.269190	0.328345	2.367658
C	7.473333	0.512384	1.661221
H	8.412307	0.164792	2.101387
C	7.484965	1.105963	0.397816
C	6.298864	1.569933	-0.205321
C	5.094835	1.393618	0.511126
H	4.150855	1.699656	0.064389
C	6.274098	-0.327468	3.687588
H	7.184822	-0.164939	4.276262
C	6.344736	2.158264	-1.553443
H	4.866201	-2.013082	1.727700
H	0.753503	-2.016377	4.515650
H	5.671600	3.230902	-3.209948
H	7.169539	1.791600	-2.175719
H	-0.474779	-3.086263	2.634549
H	-4.291259	6.076785	-2.071553
H	-0.138109	3.641538	-0.823854
H	4.138256	0.706050	2.292957
H	8.434076	1.216712	-0.134422

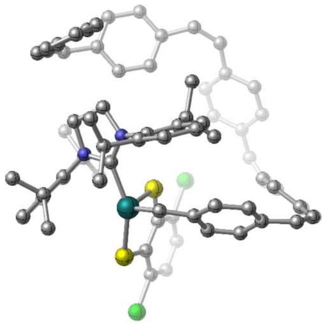

Zero-point correction= 1.226285 (Hartree/Particle)

Thermal correction to Energy= 1.300640

Thermal correction to Enthalpy= 1.301584

Thermal correction to Gibbs Free Energy= 1.114730

Sum of electronic and zero-point Energies= -4703.545126

Sum of electronic and thermal Energies= -4703.470771

Sum of electronic and thermal Enthalpies= -4703.469827

Sum of electronic and thermal Free Energies= -4703.656681

M06L-D3/def2tzvpp-SDD(Ru)-SMD(benzene).

E(scf) = -4707.87940694 a.u.

N0

E(scf) = -272.544582260 a.u.

ν_{min} = 260.81 cm^{-1}

C	-1.280154	0.671904	-0.504517	H	1.140265	1.211481	-1.530460
C	-0.086319	1.127105	0.323553	H	2.090234	1.180592	-0.029964
H	-0.116398	2.162310	0.690797	C	1.188524	-0.779946	-0.519899
H	-1.927296	1.334125	-1.084074	H	2.090011	-1.180980	-0.030133
C	-1.280327	-0.671673	-0.504471	H	1.139982	-1.211517	-1.530594
H	-1.927647	-1.333731	-1.084013	C	-0.036174	-0.000039	1.380885
C	-0.086517	-1.127112	0.323493	H	-0.908477	0.000018	2.052329
H	-0.116738	-2.162332	0.690679	H	0.889752	-0.000147	1.980029
C	1.188687	0.779792	-0.519810				

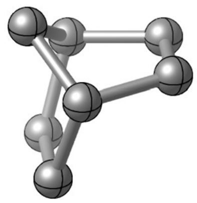

Zero-point correction= 0.152230 (Hartree/Particle)

Thermal correction to Energy= 0.157451

Thermal correction to Enthalpy= 0.158395

Thermal correction to Gibbs Free Energy= 0.123711

Sum of electronic and zero-point Energies= -272.392352

Sum of electronic and thermal Energies= -272.387131

Sum of electronic and thermal Enthalpies= -272.386187

Sum of electronic and thermal Free Energies= -272.420871

N0-Ru-1

E(scf) = -3744.31287663 a.u.

ν_{min} = 17.23 cm^{-1}

S	1.654508	0.063441	-1.050316
C	-1.220109	0.326499	-0.928973
Ru	0.039087	-0.387313	0.596762
C	-1.328019	-1.849541	1.766715
C	0.180311	1.215731	1.553345
S	1.639170	-1.904532	1.464217
N	-1.280780	1.577537	-1.458543
N	-1.840904	-0.513438	-1.793630
C	-1.123613	2.842964	-0.795097
C	-1.785726	1.575535	-2.847662
C	-2.350721	0.162702	-2.999840
C	-2.130031	-1.905960	-1.576463
H	-3.450469	0.143260	-3.012366
H	-1.988723	-0.347326	-3.902884
H	-2.537066	2.363936	-2.990551
C	-1.013170	5.414538	0.298703
C	-0.059285	3.701154	-1.149977
C	-2.142969	3.267488	0.098690
C	-2.058793	4.556110	0.640627
C	-0.028288	4.986260	-0.585148
H	-2.829954	4.901876	1.330970
H	0.790041	5.660713	-0.844560
C	-2.677680	-4.597960	-1.086097
C	-3.372084	-2.265577	-0.993807
C	-1.202126	-2.886591	-1.998003
C	-1.491022	-4.230810	-1.716608
C	-3.618307	-3.623914	-0.751853
C	-4.463182	-1.245260	-0.686837
C	0.030430	-2.539281	-2.819477
H	-0.778579	-5.003142	-2.012144
H	-4.561248	-3.926136	-0.292640
H	-0.954857	1.765813	-3.544000
H	-3.998711	-0.249671	-0.668501
H	0.205468	-1.460524	-2.723864
C	1.029120	3.307139	-2.138457
H	0.915212	2.237817	-2.356208
C	-3.344574	2.388486	0.423719
H	-3.030826	1.349372	0.280568
C	3.106036	-0.832210	-0.625502
C	4.281635	-0.721052	-1.398345
C	3.107713	-1.697713	0.498407
C	5.439037	-1.427683	-1.079928
C	4.292880	-2.399555	0.807529
C	5.446087	-2.270348	0.034416
H	6.328557	-1.319659	-1.702046
H	6.342896	-2.829706	0.303689
C	-0.992144	-2.358736	3.166112
H	-0.283544	-3.193780	3.220332
H	-1.600777	-2.550913	0.977096
C	-1.884356	-0.585595	1.935979
H	-2.672959	-0.176042	1.314305
H	-0.733973	1.678519	1.952069
C	-1.868721	-0.299025	3.436696
H	-1.956144	0.753156	3.735817
C	-2.396420	-2.652598	3.793431
H	-3.016466	-3.278749	3.133426
H	-2.280249	-3.190797	4.746710
C	-2.988721	-1.224193	4.010945
H	-3.138135	-1.010602	5.080846
H	-3.955617	-1.071216	3.509796
C	-0.618489	-1.061190	3.910761
H	0.328487	-0.618329	3.581301
H	-0.591917	-1.192814	5.003557
C	1.345488	2.034213	1.870018
C	2.683863	1.582550	1.814568
C	1.114761	3.378481	2.252703

C	3.740128	2.446421	2.098063
H	2.887498	0.545105	1.569981
C	2.172928	4.246692	2.511959
H	0.087122	3.742801	2.310728
C	3.492215	3.783583	2.432406
H	4.766445	2.073918	2.051501
H	1.970093	5.287445	2.776965
H	4.324833	4.460707	2.640261
Cl	4.306208	0.305188	-2.828132
Cl	4.350915	-3.476443	2.194075
H	-0.966684	6.418599	0.727908
H	-2.881720	-5.650410	-0.873472
C	-5.140736	-1.463751	0.675571
H	-5.768004	-0.594691	0.930600
H	-5.798126	-2.347186	0.664579
H	-4.404882	-1.604397	1.476901
C	-5.530567	-1.245791	-1.799801
H	-5.093407	-1.088480	-2.796925
H	-6.059692	-2.212248	-1.822613
H	-6.275650	-0.453337	-1.622074
C	-0.224664	-2.850555	-4.306777
H	0.641287	-2.544080	-4.915852
H	-0.384528	-3.930319	-4.463026
H	-1.114665	-2.326210	-4.690030
C	1.303326	-3.240429	-2.325225
H	2.184036	-2.824938	-2.838232
H	1.443836	-3.090863	-1.246215
H	1.279869	-4.323791	-2.526602
C	-4.506884	2.649907	-0.551116
H	-5.355221	1.981732	-0.330564
H	-4.859491	3.690544	-0.466501
H	-4.210474	2.481112	-1.596563
C	-3.823125	2.508471	1.876754
H	-4.324495	3.470283	2.068446
H	-4.549108	1.711003	2.100945
H	-2.987767	2.415090	2.585476
C	0.882652	4.084504	-3.459122
H	1.029605	5.165899	-3.302166
H	1.635813	3.745546	-4.188784
H	-0.114256	3.948430	-3.908031
C	2.437266	3.484926	-1.548367
H	2.530367	2.960302	-0.588371
H	3.187624	3.057124	-2.230446

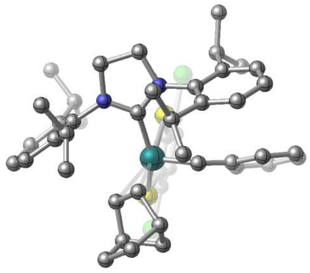

Zero-point correction= 0.922185 (Hartree/Particle)

Thermal correction to Energy= 0.976377

Thermal correction to Enthalpy= 0.977321

Thermal correction to Gibbs Free Energy= 0.835457

Sum of electronic and zero-point Energies= -3743.390692

Sum of electronic and thermal Energies= -3743.336500

Sum of electronic and thermal Enthalpies= -3743.335556

Sum of electronic and thermal Free Energies= -3743.477420

TS1-N0

E(scf) = -3744.29811071 a.u.

ν_{min} = -178.99 cm^{-1}

S	1.589597	-0.230245	-1.365029	C	-3.007639	-4.147543	0.717196
C	-1.266413	-0.250168	-0.994128	C	-4.150395	-2.013516	0.000004
Ru	0.249809	-0.168372	0.493032	C	0.415226	-3.473826	-1.852158
C	-0.867053	-1.102776	2.066565	H	-0.048549	-5.558685	-0.179526
C	-0.225517	1.604279	1.049283	H	-3.891164	-4.365466	1.321197
S	2.246855	-0.872920	1.658021	H	-1.391420	0.274693	-3.966773
N	-1.614547	0.702887	-1.903270	H	-3.865133	-1.092604	-0.522482
N	-1.698975	-1.441997	-1.487531	H	0.404640	-2.436192	-2.207331
C	-1.697482	2.126879	-1.722025	C	0.389769	2.459008	-3.194270
C	-2.132968	0.135252	-3.164837	H	0.420883	1.368251	-3.084263
C	-2.334147	-1.340759	-2.812436	C	-3.784921	1.753756	-0.253387
C	-1.781957	-2.687755	-0.773792	H	-3.278791	0.802783	-0.041288
H	-3.395898	-1.627582	-2.751289	C	3.264279	-0.457499	-0.865300
H	-1.839984	-2.019936	-3.521974	C	4.315585	-0.395335	-1.800982
H	-3.059073	0.642483	-3.469009	C	3.554373	-0.753168	0.490616
C	-2.001567	4.899233	-1.500240	C	5.639830	-0.611173	-1.426088
C	-0.776638	2.978898	-2.368419	C	4.904543	-0.974600	0.846706
C	-2.776618	2.654029	-0.961573	C	5.933456	-0.901660	-0.090644
C	-2.904654	4.045080	-0.865578	H	6.433271	-0.551988	-2.172132
C	-0.951069	4.367123	-2.239707	H	6.963630	-1.070853	0.225803
H	-3.722060	4.472903	-0.283348	C	-0.375500	-1.454401	3.474891
H	-0.243797	5.039659	-2.729516	H	0.303003	-2.314520	3.536924
C	-1.957542	-5.064494	0.681259	H	-1.530160	-1.829818	1.597512
C	-2.947408	-2.951296	-0.010594	C	-1.307159	0.288373	2.161889
C	-0.745582	-3.641550	-0.884391	H	-2.258551	0.554897	1.704529
C	-0.848435	-4.817478	-0.124992	H	-1.114288	2.066011	0.615001

C	-1.113467	0.677463	3.631345
H	-1.117322	1.756132	3.833860
C	-1.688988	-1.599760	4.304256
H	-2.406753	-2.284691	3.827823
H	-1.463107	-2.000921	5.304498
C	-2.210768	-0.129891	4.388127
H	-2.270928	0.218414	5.430576
H	-3.208949	0.003589	3.943160
C	0.145815	-0.107261	4.019522
H	1.057018	0.255286	3.535996
H	0.312728	-0.126325	5.107724
C	0.539340	2.649915	1.758767
C	1.859833	2.510526	2.233529
C	-0.099406	3.900861	1.929078
C	2.504257	3.573140	2.867130
H	2.375630	1.560515	2.091616
C	0.544703	4.962082	2.565375
H	-1.113619	4.036240	1.543925
C	1.851147	4.799658	3.039828
H	3.529003	3.444254	3.224655
H	0.030021	5.918678	2.686084
H	2.362412	5.629225	3.535173
Cl	3.976158	-0.060424	-3.496732
Cl	5.332021	-1.347619	2.509179
H	-2.118254	5.982228	-1.411512
H	-2.014895	-5.985558	1.266827
C	-4.590655	-1.606575	1.414332
H	-5.424899	-0.889234	1.361885
H	-4.939139	-2.472640	1.998503
H	-3.771209	-1.135560	1.972226
C	-5.327903	-2.636278	-0.773061
H	-5.037379	-2.922917	-1.795731
H	-5.694895	-3.543561	-0.266577
H	-6.166112	-1.923950	-0.841197
C	0.223741	-4.391846	-3.073971
H	1.029502	-4.225842	-3.807472
H	0.246045	-5.454766	-2.781573
H	-0.739536	-4.206103	-3.576165
C	1.783267	-3.706370	-1.194389
H	2.588474	-3.434343	-1.894382
H	1.900571	-3.087204	-0.293506
H	1.927890	-4.762130	-0.913159
C	-4.988185	1.435737	-1.158242
H	-5.695287	0.765319	-0.644138
H	-5.525552	2.359560	-1.426453
H	-4.679557	0.944341	-2.091936
C	-4.259463	2.311906	1.097270
H	-4.932285	3.174890	0.974554
H	-4.817686	1.538589	1.646675
H	-3.413908	2.630696	1.725791
C	0.207431	2.779269	-4.687905
H	0.203874	3.866937	-4.868385
H	1.032398	2.343974	-5.274659
H	-0.740495	2.374752	-5.077981
C	1.734328	2.990626	-2.670590
H	1.842421	2.787038	-1.595112
H	2.565279	2.487703	-3.187442
H	1.834200	4.076197	-2.833105

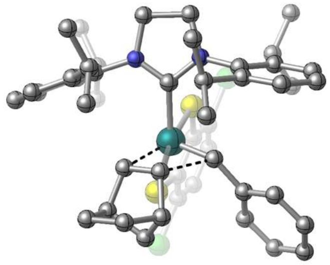

Zero-point correction= 0.922405 (Hartree/Particle)

Thermal correction to Energy= 0.975756

Thermal correction to Enthalpy= 0.976700

Thermal correction to Gibbs Free Energy= 0.836021

Sum of electronic and zero-point Energies= -3743.375705

Sum of electronic and thermal Energies= -3743.322355

Sum of electronic and thermal Enthalpies= -3743.321410

Sum of electronic and thermal Free Energies= -3743.462090

Int1-N0

E(scf) = -3744.30360138 a.u.

ν_{min} = 16.31 cm^{-1}

S	1.464072	-0.413773	-1.370115
C	-1.307108	0.023680	-0.962180
Ru	0.233854	-0.229689	0.518026
C	-1.000338	-1.155674	1.799241
C	-0.166469	1.465710	1.549315
S	2.249942	-0.964493	1.640008
N	-1.435555	1.078071	-1.810538
N	-1.956481	-1.025684	-1.531419
C	-1.223056	2.473125	-1.533270
C	-2.087597	0.725860	-3.089703
C	-2.490315	-0.736337	-2.874004
C	-2.321192	-2.271040	-0.911029
H	-3.579569	-0.893512	-2.897325
H	-2.034813	-1.417197	-3.609040
H	-2.942491	1.390234	-3.281464
C	-0.993679	5.239767	-1.178837
C	-0.184872	3.172881	-2.187698
C	-2.151285	3.151170	-0.696857
C	-2.011361	4.535424	-0.534795
C	-0.090836	4.560634	-1.990421
H	-2.709437	5.076234	0.105598
H	0.709393	5.114886	-2.485165
C	-3.059480	-4.644642	0.359980
C	-3.561415	-2.337055	-0.227991
C	-1.484592	-3.402014	-1.026404
C	-1.869853	-4.577006	-0.361773
C	-3.905901	-3.537161	0.407863
C	-4.536779	-1.164781	-0.181650
C	-0.229803	-3.405648	-1.884531
H	-1.227482	-5.458060	-0.422399

H	-4.850799	-3.605718	0.951635
H	-1.375771	0.845716	-3.919332
H	-4.061665	-0.308476	-0.675336
H	-0.047071	-2.375132	-2.211725
C	0.825761	2.494591	-3.100970
H	0.647207	1.413447	-3.057689
C	-3.304186	2.428966	-0.003389
H	-3.007086	1.379507	0.128207
C	3.113812	-0.892392	-0.970810
C	4.081838	-1.047934	-1.979612
C	3.456292	-1.131255	0.382589
C	5.386432	-1.434874	-1.682314
C	4.786904	-1.528113	0.660125
C	5.735226	-1.676088	-0.349452
H	6.119560	-1.545350	-2.482051
H	6.750631	-1.980195	-0.091041
C	-0.766693	-1.933464	3.096590
H	-0.351625	-2.936845	2.928637
H	-1.931091	-1.464972	1.323827
C	-1.069843	0.409527	2.294821
H	-2.087373	0.722732	2.038539
H	-0.814317	2.111338	0.959187
C	-0.924257	0.200399	3.830031
H	-0.660911	1.128705	4.353476
C	-2.133350	-1.924043	3.841150
H	-2.964995	-2.235206	3.191750
H	-2.103918	-2.619809	4.693912
C	-2.247846	-0.442459	4.323224
H	-2.299526	-0.382200	5.420736
H	-3.136551	0.075791	3.931088
C	0.042828	-0.983167	3.989422
H	1.061001	-0.803091	3.637508
H	0.103924	-1.329664	5.033401
C	0.877650	2.329064	2.169941
C	1.719317	2.002038	3.250362
C	1.049139	3.596730	1.571350
C	2.681129	2.903019	3.710513
H	1.651410	1.029439	3.725631
C	2.021204	4.492048	2.019741
H	0.413766	3.876334	0.729520
C	2.843433	4.150681	3.097788
H	3.320306	2.618904	4.550476
H	2.132893	5.460159	1.524348
H	3.605275	4.847377	3.456625
Cl	3.656894	-0.760137	-3.665265
Cl	5.285625	-1.848591	2.312947
H	-0.902543	6.319652	-1.038625
H	-3.337561	-5.568689	0.873092
C	-4.865213	-0.723924	1.253044
H	-5.552223	0.136489	1.243905
H	-5.350779	-1.528838	1.826518
H	-3.959732	-0.426922	1.798689
C	-5.823017	-1.487379	-0.962816
H	-5.601849	-1.792402	-1.997683
H	-6.382049	-2.308908	-0.486638
H	-6.483883	-0.606215	-0.998751
C	-0.441487	-4.262250	-3.146048
H	0.446625	-4.209404	-3.796525
H	-0.609830	-5.321322	-2.889767
H	-1.312136	-3.917361	-3.727267
C	1.017346	-3.856122	-1.108332
H	1.916329	-3.727917	-1.731040
H	1.159128	-3.257427	-0.196435
H	0.956704	-4.918087	-0.820337
C	-4.568848	2.445050	-0.881156
H	-5.395405	1.911872	-0.386037
H	-4.894281	3.481059	-1.067834
H	-4.398266	1.967545	-1.856494

C	-3.618253	2.979245	1.396743	H	1.355648	2.394012	-5.213325
H	-4.101831	3.967565	1.352247	H	-0.366772	2.762054	-4.929813
H	-4.311953	2.301300	1.917091	C	2.267639	2.721355	-2.615334
H	-2.710123	3.076097	2.010407	H	2.374436	2.442823	-1.556661
C	0.656284	2.943554	-4.562786	H	2.962685	2.096211	-3.196516
H	0.864337	4.019783	-4.679812	H	2.574238	3.773731	-2.730284

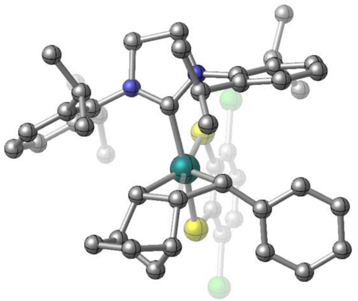

Zero-point correction= 0.924472 (Hartree/Particle)

Thermal correction to Energy= 0.977870

Thermal correction to Enthalpy= 0.978815

Thermal correction to Gibbs Free Energy= 0.838807

Sum of electronic and zero-point Energies= -3743.379129

Sum of electronic and thermal Energies= -3743.325731

Sum of electronic and thermal Enthalpies= -3743.324787

Sum of electronic and thermal Free Energies= -3743.464794

TS2-N0

E(scf) = -3744.29878191 a.u.

ν_{min} = -195.45 cm^{-1}

S	-1.204064	-1.291777	1.131171	S	-2.558668	0.136260	-1.331624
C	1.386900	-0.126765	0.853083	N	1.455754	0.640168	1.974412
Ru	-0.293775	0.031734	-0.483210	N	2.190824	-1.205480	1.064257
C	0.625863	-0.760292	-1.949643	C	1.026431	2.000999	2.151680
C	0.073467	2.042920	-1.215975	C	2.166169	-0.018120	3.088768

C	2.810978	-1.224484	2.402896
C	2.700397	-2.126694	0.082668
H	3.905619	-1.135509	2.317954
H	2.586467	-2.175430	2.907562
H	2.892740	0.666428	3.548144
C	0.398051	4.665493	2.726443
C	-0.092429	2.289919	2.963529
C	1.825161	3.041380	1.603138
C	1.487031	4.366231	1.906381
C	-0.385750	3.636343	3.237792
H	2.082418	5.182470	1.495483
H	-1.248912	3.876334	3.862240
C	3.696302	-3.857642	-1.873471
C	3.852647	-1.755467	-0.656214
C	2.102744	-3.395999	-0.085892
C	2.611238	-4.237960	-1.087211
C	4.323076	-2.633929	-1.641718
C	4.627573	-0.471793	-0.381698
C	0.973773	-3.894897	0.800318
H	2.149362	-5.214667	-1.245912
H	5.199160	-2.358622	-2.233069
H	1.444589	-0.316682	3.865139
H	4.064065	0.114121	0.353316
H	0.639547	-3.053886	1.418948
C	-0.988518	1.208093	3.546140
H	-0.628270	0.239497	3.180046
C	3.040687	2.752374	0.724862
H	2.860389	1.792747	0.220737
C	-2.859727	-1.704140	0.688981
C	-3.592603	-2.652306	1.425725
C	-3.463275	-1.052716	-0.416258
C	-4.913408	-2.962163	1.107349
C	-4.808812	-1.373749	-0.713463
C	-5.522662	-2.311834	0.029933

H	-5.456655	-3.704540	1.693134
H	-6.555747	-2.536823	-0.239149
C	0.243807	-1.065766	-3.389786
H	-0.298281	-2.023091	-3.447555
H	1.619311	-1.161358	-1.720133
C	0.852728	1.242949	-2.160190
H	1.913606	1.261300	-1.897776
H	0.692602	2.449871	-0.419733
C	0.614146	1.173844	-3.678880
H	0.422425	2.185060	-4.065817
C	1.552828	-1.030552	-4.225282
H	2.378036	-1.567277	-3.733115
H	1.387182	-1.506349	-5.203929
C	1.821499	0.498252	-4.381263
H	1.834687	0.790597	-5.441771
H	2.784699	0.812776	-3.953512
C	-0.502367	0.149418	-3.954351
H	-1.450762	0.344816	-3.447681
H	-0.698008	0.047546	-5.034159
C	-1.065153	2.964086	-1.471783
C	-1.804788	3.086660	-2.663971
C	-1.432946	3.795726	-0.390819
C	-2.864288	3.993142	-2.762881
H	-1.583484	2.463185	-3.524278
C	-2.493867	4.693043	-0.486001
H	-0.882041	3.718066	0.546379
C	-3.220462	4.798238	-1.678137
H	-3.422837	4.059800	-3.700154
H	-2.755141	5.312299	0.376262
H	-4.055204	5.498924	-1.759339
Cl	-2.845512	-3.483170	2.789070
Cl	-5.626828	-0.589937	-2.055924
H	0.153921	5.706436	2.952064
H	4.069573	-4.525464	-2.653932

C	4.786274	0.409149	-1.628363	C	4.313061	2.595438	1.576748
H	5.311513	1.343355	-1.376133	H	5.183122	2.368661	0.940673
H	5.367330	-0.095209	-2.415976	H	4.525487	3.526457	2.126364
H	3.809664	0.674163	-2.054409	H	4.217323	1.785998	2.314019
C	6.000675	-0.788395	0.239306	C	3.263390	3.795487	-0.380759
H	5.903523	-1.410070	1.142993	H	3.609729	4.759142	0.024412
H	6.640639	-1.334725	-0.472084	H	4.037739	3.439893	-1.077545
H	6.522294	0.141521	0.517640	H	2.344793	3.980019	-0.957870
C	1.472966	-5.001104	1.747154	C	-0.928774	1.185576	5.082631
H	0.663456	-5.310547	2.427964	H	-1.324350	2.118076	5.517565
H	1.804056	-5.891780	1.187686	H	-1.532283	0.351479	5.475457
H	2.322404	-4.657426	2.359626	H	0.104073	1.061064	5.446350
C	-0.245091	-4.366349	-0.007633	C	-2.434611	1.349068	3.040950
H	-1.082506	-4.583754	0.672385	H	-2.470204	1.359549	1.941392
H	-0.579142	-3.586074	-0.707141	H	-3.039202	0.494610	3.383703
H	-0.025445	-5.281325	-0.581718	H	-2.907543	2.272067	3.413616

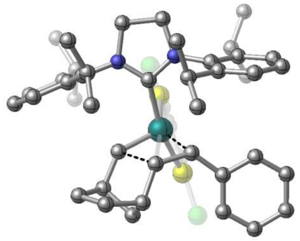

Zero-point correction= 0.922407 (Hartree/Particle)

Thermal correction to Energy= 0.975819

Thermal correction to Enthalpy= 0.976763

Thermal correction to Gibbs Free Energy= 0.835761

Sum of electronic and zero-point Energies= -3743.376375

Sum of electronic and thermal Energies= -3743.322963

Sum of electronic and thermal Enthalpies= -3743.322019

Sum of electronic and thermal Free Energies= -3743.463021

Int2-N0

E(scf) = -3744.32174041 a.u.

ν_{min} = 12.86 cm^{-1}

S	0.770324	1.969939	0.686447
C	-1.358715	-0.003395	0.875522
Ru	0.239957	0.016819	-0.501793
C	-0.404579	1.019571	-1.910345
C	0.265108	-2.382576	-1.100057
S	2.541604	-0.109091	-0.975267
N	-1.185842	-0.574038	2.099608
N	-2.430535	0.827463	0.966526
C	-0.347935	-1.692300	2.438251
C	-1.985309	0.089599	3.145751
C	-3.014874	0.862480	2.322372
C	-3.198474	1.364777	-0.124483
H	-4.006592	0.383922	2.323398
H	-3.136513	1.900603	2.658752
H	-2.428280	-0.645200	3.831190
C	1.146267	-3.903880	3.261979
C	0.865339	-1.486959	3.131323
C	-0.822106	-3.001287	2.146334
C	-0.053066	-4.091113	2.571742
C	1.599931	-2.616700	3.529466
H	-0.388228	-5.106290	2.357567
H	2.542125	-2.477718	4.063433
C	-4.718570	2.379155	-2.235992
C	-4.174552	0.538343	-0.737781
C	-3.057602	2.724985	-0.485739
C	-3.817278	3.202997	-1.564347
C	-4.910297	1.065914	-1.808215
C	-4.564236	-0.825170	-0.171091
C	-2.207628	3.694603	0.324269
H	-3.710571	4.245308	-1.871575
H	-5.659676	0.444220	-2.303071
H	-1.342360	0.763007	3.734054

H	-3.821153	-1.104591	0.584496
H	-1.553183	3.101087	0.974444
C	1.381098	-0.102326	3.492117
H	0.756139	0.634676	2.973459
C	-2.137965	-3.234996	1.405725
H	-2.259518	-2.407537	0.692635
C	2.429747	2.367665	0.258568
C	3.018687	3.577367	0.682996
C	3.216338	1.450785	-0.487396
C	4.342488	3.895163	0.386335
C	4.555975	1.789692	-0.771725
C	5.117386	2.994156	-0.348232
H	4.762265	4.842669	0.726939
H	6.154945	3.223644	-0.594234
C	-0.403911	0.757900	-3.390841
H	0.029424	1.658306	-3.862789
H	-0.738609	2.044955	-1.690776
C	-0.704513	-1.811781	-1.902018
H	-1.687406	-1.715141	-1.440438
H	-0.054316	-2.618836	-0.086107
C	-0.681502	-1.606388	-3.405379
H	-0.462324	-2.576727	-3.881563
C	-1.867996	0.519956	-3.877824
H	-2.601368	0.980153	-3.203646
H	-2.011800	0.976015	-4.868974
C	-2.012446	-1.025627	-3.945499
H	-2.143647	-1.348653	-4.989417
H	-2.887309	-1.389131	-3.390664
C	0.323627	-0.515665	-3.825925
H	1.317475	-0.608753	-3.373542
H	0.439663	-0.523579	-4.922660
C	1.558495	-3.025311	-1.412427

C	2.103663	-3.197373	-2.699376	H	-2.507324	5.208758	1.870511
C	2.292853	-3.529001	-0.316062	H	-3.775606	5.199396	0.617380
C	3.332348	-3.840293	-2.876916	H	-3.760643	3.939107	1.868811
H	1.578512	-2.833415	-3.578151	C	-1.297488	4.589820	-0.528864
C	3.518051	-4.166398	-0.492197	H	-0.615138	5.156918	0.122126
H	1.894635	-3.398454	0.691474	H	-0.668852	3.995093	-1.206472
C	4.047923	-4.323963	-1.778768	H	-1.875442	5.311288	-1.129356
H	3.734118	-3.958002	-3.886563	C	-3.338178	-3.194002	2.368748
H	4.065339	-4.536992	0.378344	H	-4.280871	-3.343902	1.819022
H	5.011713	-4.818701	-1.922880	H	-3.253218	-3.991358	3.124336
Cl	2.082848	4.728017	1.633583	H	-3.411124	-2.235439	2.899513
Cl	5.575609	0.681941	-1.679387	C	-2.168088	-4.532179	0.584722
H	1.731397	-4.769550	3.582074	H	-2.216600	-5.424997	1.227948
H	-5.294231	2.769411	-3.079013	H	-3.063940	-4.543870	-0.054910
C	-4.600527	-1.963223	-1.199457	H	-1.285907	-4.626929	-0.065460
H	-4.925538	-2.898028	-0.716504	C	1.271734	0.148273	5.006525
H	-5.303676	-1.755426	-2.020905	H	1.919637	-0.540986	5.572972
H	-3.612309	-2.145033	-1.640061	H	1.583504	1.177495	5.247326
C	-5.928686	-0.715864	0.540207	H	0.241363	0.008751	5.371321
H	-5.941027	0.099526	1.279630	C	2.814910	0.130092	2.990620
H	-6.732709	-0.513977	-0.185738	H	2.893266	-0.080460	1.914685
H	-6.169906	-1.656385	1.061345	H	3.101741	1.181494	3.147458
C	-3.115886	4.556519	1.223426	H	3.544208	-0.500878	3.524328

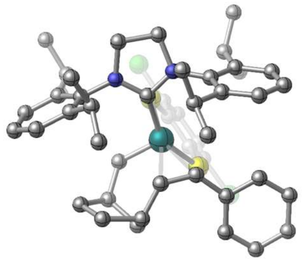

Zero-point correction= 0.923465 (Hartree/Particle)

Thermal correction to Energy= 0.977332

Thermal correction to Enthalpy= 0.978276

Thermal correction to Gibbs Free Energy= 0.837454

Sum of electronic and zero-point Energies= -3743.398275

Sum of electronic and thermal Energies= -3743.344408

Sum of electronic and thermal Enthalpies= -3743.343464

Sum of electronic and thermal Free Energies= -3743.484287

TS1-α1 (O^nOct)

E(scf) = -4867.11556036 a.u.

ν_{min} = -171.07 cm^{-1}

S	-1.611142	2.831157	0.294966
C	1.016194	2.278212	1.141010
Ru	-0.223163	1.045349	-0.076738
C	0.924173	-0.695572	0.484364
C	0.749170	1.328932	-1.701556
S	-2.246316	-0.075335	-0.758350
N	1.555632	3.484537	0.825807
N	0.922430	2.219931	2.495649
C	2.123020	3.872736	-0.435569
C	1.758270	4.359364	1.998518
C	1.263639	3.488746	3.160546
C	0.686281	1.040409	3.286946
H	2.025977	3.330844	3.936565
H	0.366485	3.903496	3.645991
H	2.817210	4.642997	2.088441
C	3.295417	4.670992	-2.845771
C	1.498628	4.873492	-1.211971
C	3.344343	3.274174	-0.850083
C	3.906219	3.689636	-2.063764
C	2.108245	5.255396	-2.418510
H	4.837973	3.240224	-2.409255
H	1.635169	6.023103	-3.034252
C	0.303991	-1.326125	4.714379
C	1.813341	0.278286	3.682318
C	-0.617737	0.691468	3.702860
C	-0.784253	-0.516008	4.399224
C	1.594456	-0.914749	4.382694
C	3.242180	0.761539	3.450811
C	-1.805421	1.624952	3.527899
H	-1.786275	-0.818034	4.710317
H	2.447314	-1.529613	4.677892
H	1.172290	5.282707	1.889034
H	3.204063	1.631463	2.783101
H	-1.513963	2.405052	2.814918
C	0.206113	5.560681	-0.795051
H	-0.183152	5.039713	0.088125
C	4.061237	2.219057	-0.010028
H	3.297984	1.665072	0.554977
C	-3.240849	2.433761	-0.243065
C	-4.275493	3.389004	-0.192727
C	-3.511248	1.137699	-0.744706
C	-5.563320	3.089825	-0.631059
C	-4.821613	0.861776	-1.198450
C	-5.834155	1.816444	-1.142717
H	-6.345899	3.847643	-0.578489
H	-6.832694	1.565929	-1.503480
H	1.693094	-0.357958	1.177891
C	1.348385	-0.457418	-0.936275

H	2.372254	-0.080050	-0.964591
H	1.752805	1.761126	-1.676274
C	0.242723	1.314003	-3.090062
C	-1.082780	1.643953	-3.430900
C	1.151147	1.018425	-4.129054
C	-1.489377	1.656616	-4.766628
H	-1.783995	1.918052	-2.646223
C	0.736698	1.003558	-5.460400
H	2.188480	0.780302	-3.880317
C	-0.588167	1.322414	-5.782945
H	-2.519130	1.927258	-5.012730
H	1.448529	0.749651	-6.249848
H	-0.913852	1.320118	-6.826311
H	3.749804	4.977679	-3.791190
H	0.149642	-2.269068	5.244813
Cl	-3.965000	4.998621	0.448051
Cl	-5.207143	-0.710549	-1.884220
C	0.457311	7.029306	-0.409339
H	0.818658	7.612613	-1.272265
H	-0.475342	7.496737	-0.054166
H	1.211158	7.119243	0.389261
C	-0.878350	5.453600	-1.880360
H	-1.013805	4.410371	-2.199660
H	-1.842320	5.807858	-1.484444
H	-0.629247	6.058446	-2.767338
C	5.009658	2.886250	1.003682
H	5.774618	3.479342	0.477103
H	4.475869	3.561810	1.685939
H	5.524688	2.129359	1.614307
C	4.834365	1.182994	-0.840257
H	5.741942	1.611020	-1.294002
H	5.153688	0.353755	-0.191909
H	4.222132	0.764121	-1.653563
C	3.869828	1.225591	4.778862

H	4.867243	1.660896	4.603724
H	3.248609	1.981639	5.284152
H	3.987090	0.377848	5.473257
C	4.130319	-0.283231	2.769058
H	4.238466	-1.188189	3.386910
H	3.715334	-0.600065	1.805176
H	5.141172	0.119731	2.600291
C	-2.130951	2.314622	4.866165
H	-2.947085	3.043585	4.733137
H	-2.450859	1.581392	5.624989
H	-1.255950	2.849863	5.269241
C	-3.045297	0.930647	2.947631
H	-3.827629	1.674320	2.730034
H	-2.807149	0.412099	2.008029
H	-3.471450	0.196540	3.650402
C	-0.831024	-2.573027	0.775182
C	0.488627	-2.106911	0.796552
C	1.517132	-3.081353	0.803634
C	1.205459	-4.405222	0.484742
C	-0.096884	-4.798188	0.149425
C	-1.143664	-3.905100	0.450741
C	-0.244171	-5.899123	-0.858109
H	-0.639245	-6.882741	-0.578613
C	1.879678	-3.821850	-2.452390
C	0.548512	-4.272335	-2.525775
C	-0.447303	-3.288089	-2.630185
H	-1.489599	-3.587518	-2.766818
C	-0.164546	-1.953786	-2.347158
C	1.112687	-1.570799	-1.918685
C	2.161414	-2.482806	-2.150213
H	3.191128	-2.193957	-1.924539
C	0.126810	-5.660894	-2.138324
H	0.058135	-6.461619	-2.884674
H	-0.983653	-1.240107	-2.314536

H	2.697278	-4.547851	-2.461658
H	2.007062	-5.111426	0.278092
H	-1.626030	-1.835074	0.825419
O	2.809312	-2.633366	0.884362
O	-2.411967	-4.343038	0.225089
C	3.876633	-3.542334	0.655267
H	3.719771	-4.075929	-0.298075
C	-3.505985	-3.482431	0.528481
H	-3.455048	-3.178336	1.592414
H	-3.456646	-2.562864	-0.078971
H	3.905540	-4.305101	1.456882
C	5.186041	-2.774738	0.588761
H	5.063308	-1.945261	-0.128387
H	5.399351	-2.315094	1.565544
C	6.362740	-3.655712	0.166152
H	6.151541	-4.107067	-0.820827
H	6.463964	-4.502318	0.870156
C	7.687103	-2.891611	0.099513
H	7.898736	-2.443015	1.087911
H	7.580198	-2.040808	-0.599012
C	8.875340	-3.754261	-0.330325
H	8.661913	-4.202841	-1.318462
H	8.980260	-4.604783	0.368806
C	10.198618	-2.988790	-0.397750
H	10.411345	-2.539338	0.590506
H	10.093144	-2.138068	-1.097092
C	11.389385	-3.850142	-0.827076
H	11.174123	-4.298706	-1.814178

H	11.492239	-4.699636	-0.127292
C	12.706127	-3.075177	-0.891177
H	13.543711	-3.719959	-1.202315
H	12.642004	-2.240229	-1.609405
H	12.962684	-2.644410	0.091537
C	-4.798603	-4.227905	0.247647
H	-4.839015	-4.468039	-0.829319
H	-4.782633	-5.192641	0.784255
C	-6.029134	-3.412202	0.650238
H	-6.013876	-3.238789	1.742296
H	-5.971030	-2.414710	0.182558
C	-7.355149	-4.066578	0.258915
H	-7.435192	-5.060107	0.738344
H	-7.357428	-4.256814	-0.830513
C	-8.577931	-3.221908	0.623589
H	-8.575121	-3.024865	1.712031
H	-8.488894	-2.231468	0.139322
C	-9.911129	-3.860347	0.228725
H	-10.000545	-4.850665	0.713807
H	-9.912537	-4.059044	-0.859596
C	-11.133183	-3.010586	0.587477
H	-11.040680	-2.021604	0.102314
H	-11.129636	-2.812605	1.675088
C	-12.460299	-3.656691	0.187504
H	-12.593848	-4.632275	0.685113
H	-13.320528	-3.023135	0.457731
H	-12.504322	-3.834373	-0.900460

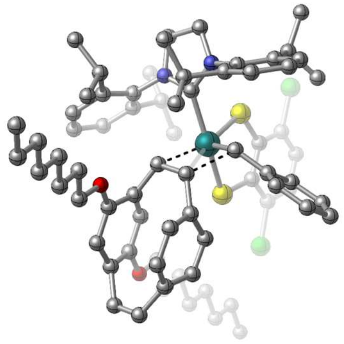

Zero-point correction= 1.458329 (Hartree/Particle)

Thermal correction to Energy= 1.542031

Thermal correction to Enthalpy= 1.542976

Thermal correction to Gibbs Free Energy= 1.331662

Sum of electronic and zero-point Energies= -4865.657231

Sum of electronic and thermal Energies= -4865.573529

Sum of electronic and thermal Enthalpies= -4865.572585

Sum of electronic and thermal Free Energies= -4865.783899

M06L-D3/def2tzvpp-SDD(Ru)-SMD(benzene).

E(scf) = -4870.37281174 a.u.

TS1-α2 (O^nOct)

E(scf) = -4867.10926537 a.u.

ν_{min} = -109.71 cm^{-1}

S	2.864875	1.357518	-0.670491
C	0.209160	2.382925	-1.075475
Ru	0.835323	0.693584	0.122661
C	-0.963219	-0.349407	-0.362222
C	0.214676	1.466414	1.763737
S	2.032287	-1.142069	1.084590
N	0.479170	3.685141	-0.779507
N	-0.085476	2.335837	-2.400928
C	0.488351	4.310531	0.514341
C	0.574573	4.543793	-1.977763
C	0.022115	3.639586	-3.080540
C	-0.649001	1.238133	-3.139321
H	-0.964677	3.960691	-3.450537
H	0.696707	3.556968	-3.944855
H	0.000728	5.470706	-1.841023
C	0.441241	5.643243	2.971900
C	1.694671	4.800658	1.058939
C	-0.752340	4.501133	1.182991
C	-0.746245	5.165768	2.414820
C	1.643339	5.463663	2.296581

H	-1.682472	5.313394	2.954504
H	2.568066	5.843408	2.735799
C	-1.776807	-0.908214	-4.518111
C	-2.060927	1.118869	-3.201482
C	0.196260	0.342634	-3.825251
C	-0.394947	-0.738408	-4.498359
C	-2.601268	0.025318	-3.889451
C	-3.000229	2.156757	-2.591846
C	1.700748	0.545549	-3.899443
H	0.243026	-1.456476	-5.017674
H	-3.685572	-0.099482	-3.933924
H	1.625190	4.819137	-2.156118
H	-2.389081	2.886484	-2.047516
H	1.962285	1.356345	-3.207960
C	3.034691	4.668946	0.349914
H	2.897274	3.994713	-0.504035
C	-2.073377	4.008359	0.592759
H	-1.850967	3.117602	-0.011725
C	4.107823	0.297585	0.003737
C	5.473486	0.529676	-0.249997
C	3.724509	-0.786859	0.834465
C	6.467483	-0.266907	0.315051
C	4.753737	-1.571202	1.408842
C	6.099595	-1.318301	1.160711
H	7.517190	-0.064913	0.099758
H	6.861821	-1.947961	1.621020
H	-1.430425	0.273400	-1.119892
C	-1.260860	0.181072	1.005078
H	-1.929246	1.043996	0.961856
H	-0.487860	2.299443	1.744218
C	0.710315	1.277605	3.141286
C	2.074409	1.096172	3.440993
C	-0.206299	1.373788	4.209965
C	2.503084	0.995116	4.765566

H	2.797783	1.062104	2.628710
C	0.218486	1.237432	5.531633
H	-1.265577	1.532294	3.994051
C	1.576953	1.048160	5.812936
H	3.567168	0.866562	4.978495
H	-0.509688	1.286876	6.345081
H	1.913721	0.952093	6.848374
H	0.424314	6.156714	3.936439
H	-2.216434	-1.763922	-5.036392
Cl	5.967303	1.846824	-1.308902
Cl	4.351636	-2.907020	2.479506
C	3.509993	6.031335	-0.186015
H	3.697658	6.739713	0.637734
H	4.449105	5.914734	-0.750886
H	2.763470	6.490415	-0.853692
C	4.106022	4.025178	1.244454
H	3.747209	3.075086	1.664646
H	5.008036	3.807341	0.652877
H	4.395545	4.685392	2.077865
C	-2.687176	5.060426	-0.348514
H	-2.902912	5.990011	0.202154
H	-2.012921	5.312002	-1.178881
H	-3.630818	4.693216	-0.782058
C	-3.102883	3.577193	1.648371
H	-3.521544	4.437754	2.193038
H	-3.942814	3.059929	1.160358
H	-2.667944	2.891009	2.390843
C	-3.749877	2.921418	-3.698267
H	-4.349952	3.738638	-3.266176
H	-3.053056	3.357419	-4.431050
H	-4.433481	2.253924	-4.247205
C	-3.991767	1.566834	-1.575933
H	-4.628597	0.790183	-2.027365
H	-3.473093	1.119416	-0.717360

H	-4.656288	2.355685	-1.190006
C	2.122541	0.983057	-5.313321
H	3.206781	1.178651	-5.345409
H	1.898774	0.200795	-6.057362
H	1.599829	1.901044	-5.627958
C	2.471564	-0.697780	-3.440630
H	3.544994	-0.470877	-3.345304
H	2.111306	-1.039352	-2.462765
H	2.364958	-1.531126	-4.153758
C	-0.548498	-2.970555	-0.618956
C	-1.336360	-1.796538	-0.619456
C	-2.728725	-1.983347	-0.597087
C	-3.316352	-3.187633	-0.207288
C	-2.481772	-4.238493	0.223058
C	-1.122578	-4.159321	-0.120355
C	-3.006472	-5.168261	1.278318
H	-3.300312	-6.195805	1.032756
C	-3.568404	-2.196008	2.695846
C	-2.714928	-3.305348	2.838025
C	-1.336248	-3.046729	2.906436
H	-0.644377	-3.870437	3.099331
C	-0.818797	-1.810780	2.529130
C	-1.669137	-0.802278	2.056888
C	-3.047773	-0.954877	2.307651
H	-3.738983	-0.151254	2.035443
C	-3.156122	-4.707316	2.540083
H	-3.579608	-5.354230	3.317572
H	0.259896	-1.684744	2.474255
H	-4.651435	-2.339026	2.711482
H	-3.357417	-1.101443	-0.695086
O	0.724281	-2.880188	-1.070595
O	-4.657602	-3.335071	-0.014557
H	-0.474420	-4.997905	0.135279
C	1.658907	-3.919603	-0.802934

H	1.289532	-4.881874	-1.205312
H	1.779287	-4.025313	0.290362
C	2.980451	-3.530209	-1.443774
H	2.886568	-3.570101	-2.542147
H	3.167105	-2.479630	-1.184456
C	4.168429	-4.365085	-0.967664
H	4.066101	-5.418072	-1.289501
H	4.168710	-4.377616	0.134947
C	5.505804	-3.793125	-1.448580
H	5.599562	-3.920284	-2.543120
H	5.505717	-2.703687	-1.268873
C	6.723284	-4.396660	-0.747230
H	6.764991	-5.486234	-0.933580
H	6.593496	-4.281545	0.344751
C	8.046370	-3.751634	-1.163778
H	8.195250	-3.883652	-2.252033
H	7.978358	-2.659888	-1.000437
C	9.264318	-4.296872	-0.414198
H	9.331980	-5.388623	-0.574207
H	9.107588	-4.162367	0.672017
C	-5.526213	-2.252474	-0.296755
H	-5.422656	-1.943504	-1.355683
H	-5.264124	-1.376004	0.325750
C	-6.949280	-2.696220	-0.005841
H	-7.175400	-3.584377	-0.620570
H	-7.005578	-3.023621	1.046874
C	-7.974837	-1.593033	-0.270774
H	-7.898111	-1.263393	-1.323641
H	-7.727707	-0.705651	0.341424
C	-9.415309	-2.020007	0.019903
H	-9.662401	-2.907071	-0.592132
H	-9.491531	-2.350629	1.072277
C	-10.446373	-0.920147	-0.241929
H	-10.367798	-0.589382	-1.294509

H	-10.197472	-0.033260	0.370474
C	-11.887410	-1.345765	0.047643
H	-12.136048	-2.232950	-0.564567
H	-11.965837	-1.676975	1.100203
C	-12.920775	-0.246671	-0.213774
H	-12.670021	0.638850	0.398653
H	-12.839922	0.083164	-1.265792
C	-14.356920	-0.683227	0.078589
H	-14.473897	-0.986175	1.132908
H	-15.077386	0.126918	-0.118307
H	-14.645213	-1.546317	-0.545112
C	10.578215	-3.633303	-0.828788
H	10.547991	-2.545370	-0.647434
H	11.436628	-4.041531	-0.271133
H	10.776711	-3.780904	-1.903965

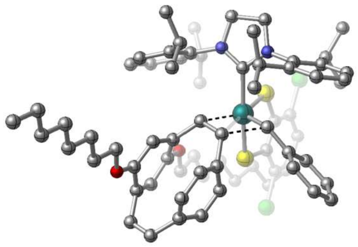

Zero-point correction= 1.458611 (Hartree/Particle)

Thermal correction to Energy= 1.541847

Thermal correction to Enthalpy= 1.542791

Thermal correction to Gibbs Free Energy= 1.334873

Sum of electronic and zero-point Energies= -4865.650655

Sum of electronic and thermal Energies= -4865.567419

Sum of electronic and thermal Enthalpies= -4865.566475

Sum of electronic and thermal Free Energies= -4865.774393

TS1-β2 (O^{n}Oct)

E(scf) = -4867.10897214 a.u.

ν_{min} = -232.79 cm^{-1}

S	-2.358178	-2.403496	-0.834533
C	0.376033	-2.663259	-0.018940
Ru	-0.774982	-0.864979	-0.269135
C	0.968254	0.252509	-0.776278
C	-0.737307	-0.422291	1.595549
S	-2.281305	0.740422	-1.173278
N	0.175310	-3.624970	0.923628
N	1.066143	-3.244077	-1.036039
C	-0.183245	-3.441344	2.304129
C	0.599932	-4.970444	0.485818
C	1.281371	-4.690907	-0.856882
C	1.733443	-2.598980	-2.135342

H	2.357301	-4.923294	-0.853543	H	1.734685	-0.502467	-0.932374
H	0.820403	-5.240249	-1.691533	C	0.804040	0.520933	0.700533
H	1.269758	-5.425702	1.229096	H	1.492660	-0.124195	1.243895
C	-0.798197	-3.181512	5.020991	H	-0.052027	-0.929494	2.276656
C	-1.433723	-3.894078	2.778210	C	-1.790173	0.242183	2.395931
C	0.777410	-2.878201	3.189010	C	-3.156632	0.079511	2.106336
C	0.440551	-2.753349	4.542102	C	-1.427033	0.918172	3.578150
C	-1.717914	-3.749073	4.146456	C	-4.130292	0.571515	2.977497
H	1.155389	-2.312909	5.238310	H	-3.450532	-0.455250	1.204757
H	-2.683357	-4.087198	4.528081	C	-2.399754	1.444788	4.427148
C	3.083766	-1.351962	-4.237386	H	-0.367837	1.040177	3.819616
C	3.089995	-2.220792	-1.963704	C	-3.757731	1.263920	4.133616
C	1.070809	-2.418522	-3.368332	H	-5.187257	0.425446	2.741348
C	1.766048	-1.770565	-4.401940	H	-2.100636	1.985324	5.328905
C	3.743235	-1.594120	-3.032638	H	-4.522064	1.660695	4.806774
C	3.867418	-2.499814	-0.679234	H	-1.042622	-3.071287	6.080391
C	-0.324130	-2.963232	-3.631434	H	3.604407	-0.845777	-5.053948
H	1.264982	-1.604150	-5.357822	Cl	-5.014658	-4.005307	-1.647815
H	4.784785	-1.284685	-2.918920	Cl	-4.882168	2.244011	-2.355962
H	-0.277449	-5.624684	0.378385	C	-2.577963	-6.061286	2.180038
H	3.173588	-2.940082	0.047077	H	-2.939449	-6.238517	3.206397
H	-0.726794	-3.322599	-2.677016	H	-3.287269	-6.540189	1.485696
C	-2.471169	-4.554833	1.881665	H	-1.605274	-6.569002	2.080104
H	-2.142894	-4.435028	0.841897	C	-3.845784	-3.875285	1.988881
C	2.156722	-2.425526	2.712998	H	-3.760158	-2.791391	1.830150
H	2.061922	-2.135699	1.657424	H	-4.523759	-4.272312	1.218657
C	-3.777564	-1.552776	-1.449734	H	-4.310498	-4.045585	2.973487
C	-4.948955	-2.252503	-1.799978	C	3.171521	-3.581035	2.783479
C	-3.731074	-0.144172	-1.601177	H	3.287164	-3.927830	3.822879
C	-6.073651	-1.592125	-2.289068	H	2.857041	-4.441923	2.177180
C	-4.882671	0.502521	-2.109692	H	4.159217	-3.256611	2.419711
C	-6.036012	-0.202257	-2.443515	C	2.699093	-1.198222	3.462460
H	-6.967559	-2.159129	-2.551337	H	2.997057	-1.443329	4.493629
H	-6.903779	0.335376	-2.828314	H	3.593604	-0.812975	2.949581

H	1.960131	-0.384281	3.511967
C	4.986468	-3.527504	-0.925917
H	5.480724	-3.795593	0.021876
H	4.595349	-4.450391	-1.381892
H	5.754825	-3.122653	-1.604029
C	4.436130	-1.226519	-0.030960
H	5.148304	-0.711867	-0.695205
H	3.641507	-0.512045	0.222701
H	4.974396	-1.475484	0.896833
C	-0.259155	-4.165755	-4.590669
H	-1.262641	-4.600858	-4.726454
H	0.116958	-3.868124	-5.583422
H	0.406535	-4.955132	-4.205191
C	-1.294262	-1.890526	-4.148930
H	-2.312076	-2.305036	-4.218549
H	-1.335441	-1.027007	-3.469140
H	-1.008879	-1.530671	-5.150612
C	0.388638	2.472695	-1.994226
C	1.268044	1.432333	-1.655788
C	2.645077	1.680966	-1.862687
C	3.123835	2.977736	-2.049760
C	2.232322	4.065445	-2.067982
C	0.867700	3.767789	-2.207804
C	2.664144	5.402797	-1.545064
H	3.063629	6.179054	-2.208751
C	2.917703	3.497207	1.099792
C	2.062496	4.532974	0.674085
C	0.682849	4.318541	0.775305
H	0.024817	5.121575	0.448404
C	0.134293	3.043664	1.011395
C	1.010236	1.947968	1.152851
C	2.375305	2.233546	1.351003
H	3.049665	1.392710	1.506853
C	2.591077	5.622576	-0.213625
H	2.939769	6.573785	0.205316
H	4.201154	3.160470	-2.014063
H	-0.686507	2.310786	-1.948716
O	-1.193611	2.792821	0.970815
O	4.258394	3.737855	1.048641
H	3.361612	0.871884	-1.713729
C	5.157991	2.640321	1.048681
H	5.138586	2.124390	2.028628
H	4.853169	1.902674	0.282941
C	6.554130	3.156835	0.750537
H	6.830523	3.904107	1.514166
H	6.530666	3.690335	-0.215461
C	7.589962	2.031889	0.708384
H	7.593110	1.497400	1.676533
H	7.287436	1.282688	-0.047220
C	9.006977	2.517604	0.396599
H	9.310025	3.265628	1.152307
H	9.004095	3.052271	-0.571206
C	10.043372	1.392734	0.351942
H	10.042698	0.857607	1.320001
H	9.737920	0.645141	-0.404003
C	11.462457	1.873884	0.041885
H	11.767494	2.622036	0.797475
H	11.463427	2.408792	-0.926399
C	12.500099	0.748895	-0.001834
H	12.496187	0.215330	0.966345
H	12.192967	0.002483	-0.757249
C	13.914618	1.240678	-0.311374
H	14.259794	1.962854	0.447799
H	14.638534	0.410328	-0.336325
H	13.954100	1.748594	-1.289887
C	-2.112426	3.836060	0.664507
H	-2.018661	4.101496	-0.405768
H	-1.879153	4.739089	1.258424

C	-3.512584	3.350511	0.983197	C	-8.530771	4.070671	0.482285
H	-3.654252	2.382762	0.484063	H	-8.592216	3.058608	0.039674
H	-3.583342	3.159634	2.065332	H	-8.685304	3.931956	1.569060
C	-4.605454	4.318610	0.536604	C	-9.659973	4.937101	-0.081536
H	-4.492114	4.520367	-0.543685	H	-9.596815	5.947879	0.361800
H	-4.493255	5.294504	1.045827	H	-9.501855	5.075185	-1.166868
C	-6.008482	3.762358	0.790200	C	-11.052403	4.354552	0.164427
H	-6.078182	2.760592	0.330305	H	-11.249834	4.237372	1.243583
H	-6.153068	3.610558	1.876143	H	-11.843985	4.998214	-0.252191
C	-7.131854	4.640143	0.237921	H	-11.153672	3.357899	-0.297761
H	-6.979512	4.773574	-0.849356	H	0.147445	4.584956	-2.298970
H	-7.064372	5.652123	0.679779				

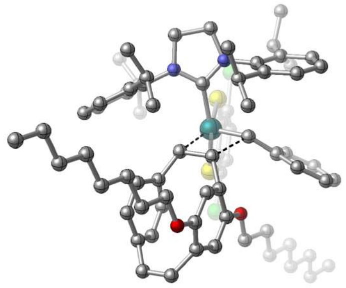

Zero-point correction= 1.458405 (Hartree/Particle)

Thermal correction to Energy= 1.541885

Thermal correction to Enthalpy= 1.542829

Thermal correction to Gibbs Free Energy= 1.333832

Sum of electronic and zero-point Energies= -4865.650567

Sum of electronic and thermal Energies= -4865.567087

Sum of electronic and thermal Enthalpies= -4865.566143

Sum of electronic and thermal Free Energies= -4865.775140

TS1-β1 (O^nOct)

E(scf) = -4867.11914049 a.u.

ν_{min} = -119.32 cm^{-1}

S	1.373327	-3.174846	0.344223	C	-1.365175	-2.512540	0.440702

Ru	0.286615	-1.175861	0.176774
C	-0.801716	0.426172	1.111683
C	-0.163783	-0.700263	-1.622358
S	2.475229	-0.205136	0.391139
N	-1.759580	-3.480486	-0.432384
N	-1.828871	-2.869896	1.667121
C	-1.812002	-3.393464	-1.866692
C	-2.367944	-4.649511	0.235115
C	-2.523139	-4.169465	1.681501
C	-1.871249	-2.060122	2.855973
H	-3.574149	-4.041042	1.984162
H	-2.042185	-4.841310	2.407701
H	-3.321240	-4.917874	-0.241364
C	-2.030847	-3.272588	-4.654303
C	-0.977035	-4.206804	-2.664152
C	-2.778262	-2.533790	-2.457471
C	-2.860162	-2.485913	-3.854269
C	-1.107244	-4.125137	-4.060845
H	-3.583918	-1.824146	-4.330808
H	-0.465289	-4.743328	-4.691571
C	-1.988944	-0.432604	5.121094
C	-3.014505	-1.249717	3.071473
C	-0.823651	-2.117943	3.799674
C	-0.899927	-1.276838	4.921430
C	-3.045118	-0.435152	4.210287
C	-4.218516	-1.267252	2.134336
C	0.332885	-3.097141	3.676637
H	-0.090809	-1.292369	5.654657
H	-3.908927	0.209735	4.386894
H	-1.695699	-5.516913	0.157491
H	-3.971920	-1.906995	1.278864
H	0.267829	-3.567894	2.688331
C	0.036805	-5.183667	-2.086150
H	0.106516	-5.000162	-1.007660
C	-3.733238	-1.690042	-1.616400
H	-3.209021	-1.418110	-0.688940
C	3.110094	-2.885664	0.386257
C	4.019518	-3.961411	0.428404
C	3.597417	-1.555994	0.364243
C	5.396130	-3.751944	0.427733
C	4.999319	-1.369798	0.350224
C	5.886271	-2.443329	0.380322
H	6.077159	-4.603164	0.460289
H	6.960612	-2.254498	0.366872
H	-1.713481	-0.025246	1.494585
C	-0.937166	0.739328	-0.341987
H	-1.930688	0.478536	-0.702935
H	-1.156860	-0.918180	-2.022000
C	0.731921	-0.265390	-2.712028
C	2.058669	-0.719500	-2.842135
C	0.214589	0.598101	-3.701307
C	2.849878	-0.299395	-3.912897
H	2.454166	-1.425013	-2.114048
C	1.015528	1.040769	-4.753665
H	-0.819480	0.942634	-3.621574
C	2.338467	0.594335	-4.860419
H	3.873996	-0.669761	-4.003446
H	0.607110	1.731421	-5.495639
H	2.965881	0.933803	-5.688606
H	-2.109838	-3.219024	-5.742992
H	-2.022657	0.221036	5.996182
Cl	3.430955	-5.619112	0.503162
Cl	5.669611	0.249542	0.280796
C	-0.417222	-6.639406	-2.294564
H	-0.471300	-6.891222	-3.366552
H	0.294981	-7.334283	-1.820706
H	-1.413562	-6.821912	-1.860964
C	1.445809	-4.957232	-2.658231

H	1.748001	-3.905552	-2.549250
H	2.176695	-5.572369	-2.112406
H	1.502862	-5.225984	-3.725335
C	-4.980104	-2.504017	-1.224011
H	-5.528861	-2.822258	-2.124922
H	-4.721361	-3.407095	-0.654276
H	-5.661751	-1.899886	-0.605051
C	-4.153848	-0.374870	-2.284068
H	-4.819953	-0.542670	-3.144878
H	-4.701508	0.244365	-1.562732
H	-3.288963	0.209936	-2.631256
C	-5.441761	-1.888338	2.833203
H	-6.280354	-1.991543	2.125509
H	-5.212183	-2.885221	3.241029
H	-5.782250	-1.258160	3.670628
C	-4.566553	0.115791	1.562980
H	-4.813387	0.837356	2.357755
H	-3.743888	0.539341	0.970160
H	-5.446756	0.039872	0.905753
C	0.209870	-4.212717	4.730285
H	1.014222	-4.954498	4.597460
H	0.287733	-3.809708	5.753553
H	-0.755935	-4.737936	4.651585
C	1.703130	-2.406915	3.753760
H	2.503904	-3.130734	3.535894
H	1.782276	-1.594018	3.017175
H	1.892484	-1.986781	4.754699
C	0.941367	1.989109	2.250479
C	-0.371487	1.563189	2.003726
C	-1.402994	2.465095	2.347345
C	-1.124446	3.814505	2.567451
C	0.194145	4.296092	2.478725
C	1.222002	3.339265	2.485180
C	0.466585	5.691477	1.998046

H	0.694397	6.503312	2.699513
C	-1.172807	4.427411	-0.495094
C	0.157589	4.797294	-0.267728
C	1.167071	3.870556	-0.598984
C	0.820994	2.531123	-0.826749
C	-0.513762	2.108647	-0.782591
C	-1.524641	3.100062	-0.772199
C	0.464872	5.931008	0.667465
H	0.698123	6.934019	0.291085
H	-1.953856	4.523630	2.638011
H	1.767178	1.295463	2.101471
H	-2.444743	2.147581	2.260684
H	2.264688	3.667848	2.488807
O	-2.826077	2.674841	-0.828891
O	2.440044	4.324147	-0.488875
H	-1.947260	5.152785	-0.251409
H	1.601268	1.774512	-0.874950
C	-3.865006	3.628719	-0.659560
H	-3.738582	4.460778	-1.377729
H	-3.815862	4.061245	0.358276
C	-5.204141	2.949753	-0.880578
H	-5.228914	2.535350	-1.902165
H	-5.285054	2.095848	-0.190840
C	-6.388194	3.894547	-0.669229
H	-6.308986	4.752462	-1.362038
H	-6.340958	4.322950	0.349053
C	-7.741958	3.207711	-0.864248
H	-7.789904	2.779598	-1.882659
H	-7.814462	2.346969	-0.173472
C	-8.939988	4.133955	-0.646149
H	-8.867824	4.993210	-1.338687
H	-8.888036	4.563838	0.371584
C	-10.292839	3.444172	-0.834887
H	-10.344918	3.014340	-1.852906

H	-10.363680	2.583965	-0.142779
C	-11.493582	4.368365	-0.615256
H	-11.438403	4.797601	0.401934
H	-11.420708	5.226895	-1.307722
C	-12.839836	3.668268	-0.804922
H	-12.953311	2.826392	-0.101010
H	-13.684046	4.357089	-0.640825
H	-12.935413	3.258965	-1.824926
C	3.550837	3.435791	-0.537045
H	3.538109	2.847238	-1.473658
H	3.502050	2.713182	0.296999
C	4.798356	4.303324	-0.460451
H	4.813352	4.966684	-1.343079
H	4.703739	4.963188	0.419487
C	6.097705	3.505423	-0.375463
H	6.134271	2.763099	-1.192907
H	6.101293	2.922015	0.561529
C	7.356040	4.373405	-0.424729
H	7.366547	4.953086	-1.366523
H	7.317158	5.121686	0.388784
C	8.650645	3.565289	-0.310482
H	8.685041	2.817031	-1.124267
H	8.633441	2.981704	0.628844
C	9.919200	4.419443	-0.353760
H	9.884395	5.167011	0.461046
H	9.935664	5.002277	-1.294009
C	11.213364	3.609283	-0.239856
H	11.245574	2.862690	-1.054462
H	11.194080	3.027277	0.699755
C	12.475210	4.472103	-0.283815
H	13.389441	3.862595	-0.199820
H	12.536804	5.039270	-1.228141
H	12.484929	5.205162	0.540639

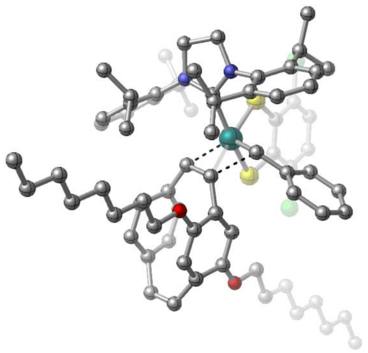

Zero-point correction= 1.458454 (Hartree/Particle)

Thermal correction to Energy= 1.542134

Thermal correction to Enthalpy= 1.543078

Thermal correction to Gibbs Free Energy= 1.332168

Sum of electronic and zero-point Energies= -4865.660686

Sum of electronic and thermal Energies= -4865.577007

Sum of electronic and thermal Enthalpies= -4865.576063

Sum of electronic and thermal Free Energies= -4865.786972

M06L-D3/def2tzvpp-SDD(Ru)-SMD(benzene)

E(scf) = -4870.37871650 a.u.

ent-M2 (O^nOct)

E(scf) = -1395.35626315 a.u.

ν_{min} = 5.96 cm^{-1}

C	-0.970557	-0.987576	-1.044743	H	-1.726797	-1.763918	-0.932029
C	0.373903	-1.358607	-0.898567	C	-3.696536	-0.174022	-0.837223
C	1.357585	-0.360852	-1.053443	H	-3.722397	-0.856074	-1.709036
C	0.970564	0.987352	-1.044939	H	-3.523958	-0.795423	0.062423
C	-0.373897	1.358413	-0.898841	C	3.696543	0.173841	-0.837262
C	-1.357579	0.360627	-1.053520	H	3.722402	0.855700	-1.709227
C	0.714517	-2.627609	-0.170992	H	3.523969	0.795444	0.062246
H	0.995010	-3.537427	-0.714495	C	5.007170	-0.584589	-0.714010
C	-0.714514	2.627564	-0.171529	H	5.133655	-1.216117	-1.610282
H	-0.995001	3.537271	-0.715222	H	4.932011	-1.274110	0.144729
C	0.986408	0.977319	2.073566	C	6.213588	0.339950	-0.544190
C	-0.354149	1.366551	1.908770	H	6.270697	1.033991	-1.403135
C	-1.338344	0.376049	2.073230	H	6.067825	0.976189	0.348508
H	-2.393772	0.651964	2.004533	C	7.539952	-0.412576	-0.415074
C	-0.986446	-0.976909	2.073736	H	7.686022	-1.048644	-1.307673
C	0.354114	-1.366173	1.909044	H	7.481998	-1.107789	0.442858
C	1.338305	-0.375638	2.073332	C	8.752188	0.505129	-0.242654
H	2.393735	-0.651567	2.004714	H	8.808386	1.200653	-1.100824
C	-0.691469	2.635613	1.181220	H	8.604123	1.141491	0.649827
H	-0.942517	3.554753	1.724139	C	10.079180	-0.245851	-0.113158
C	0.691452	-2.635381	1.181758	H	10.227202	-0.882421	-1.005718
H	0.942488	-3.554411	1.724870	H	10.022705	-0.941888	0.744838
H	-1.775956	-1.730712	2.010193	C	11.293406	0.670638	0.059837
H	1.775920	1.731109	2.009892	H	11.347346	1.365581	-0.798159
O	-2.653225	0.775306	-0.984077	H	11.142950	1.306058	0.951814
O	2.653231	-0.775517	-0.983904	C	12.613677	-0.090284	0.188160
H	1.726803	1.763716	-0.932377	H	13.468406	0.594162	0.311334

H	12.599457	-0.767893	1.058641
H	12.805582	-0.707773	-0.705693
C	-5.007164	0.584436	-0.714140
H	-5.133660	1.215748	-1.610562
H	-4.931995	1.274164	0.144432
C	-6.213578	-0.340063	-0.544081
H	-6.270688	-1.034324	-1.402848
H	-6.067812	-0.976074	0.348779
C	-7.539944	0.412494	-0.415155
H	-7.686023	1.048320	-1.307925
H	-7.481983	1.107939	0.442588
C	-8.752176	-0.505168	-0.242475
H	-8.808375	-1.200931	-1.100450
H	-8.604105	-1.141279	0.650183
C	-10.079169	0.245846	-0.113183
H	-10.227200	0.882158	-1.005925
H	-10.022689	0.942130	0.744612
C	-11.293391	-0.670597	0.060085
H	-11.142928	-1.305756	0.952247
H	-11.347334	-1.365790	-0.797707
C	-12.613664	0.090358	0.188193
H	-12.805576	0.707585	-0.705839
H	-13.468390	-0.594054	0.311573
H	-12.599441	0.768223	1.058476

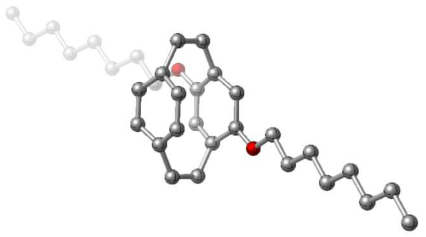

Zero-point correction= 0.687600 (Hartree/Particle)

Thermal correction to Energy= 0.723129

Thermal correction to Enthalpy= 0.724073

Thermal correction to Gibbs Free Energy= 0.614960

Sum of electronic and zero-point Energies= -1394.668663

Sum of electronic and thermal Energies= -1394.633135

Sum of electronic and thermal Enthalpies= -1394.632190

Sum of electronic and thermal Free Energies= -1394.741303

M06L-D3/def2tzvpp-SMD(benzene).

E(scf) = -1396.61457512 a.u.

M2´ (O^{n}Oct)

E(scf) = -1395.35626315 a.u.

ν_{min} = 5.96 cm^{-1}

O	2.653229	0.775344	-0.983978
O	-2.653226	-0.775482	-0.983861
C	1.357584	0.360666	-1.053445
C	0.373901	1.358447	-0.898731
C	-0.970559	0.987390	-1.044840
H	-1.726796	1.763752	-0.932249
C	-1.357582	-0.360814	-1.053391
C	-0.373898	-1.358574	-0.898554
C	0.970561	-0.987536	-1.044719
H	1.726799	-1.763884	-0.932034
C	0.714517	2.627569	-0.171367
H	0.995011	3.537294	-0.715025
C	-0.714510	-2.627602	-0.171025
H	-0.995001	-3.537400	-0.714563
C	3.696542	-0.173989	-0.837188
H	3.722384	-0.856003	-1.709032
H	3.523985	-0.795432	0.062433
C	5.007172	0.584463	-0.714098
H	4.932020	1.274154	0.144505
H	5.133648	1.215816	-1.610494
C	6.213590	-0.340042	-0.544107
H	6.067845	-0.976092	0.348729
H	6.270681	-1.034266	-1.402905
C	7.539958	0.412510	-0.415179
H	7.482017	1.107917	0.442596
H	7.686016	1.048377	-1.307923
C	8.752195	-0.505159	-0.242570
H	8.604145	-1.141312	0.650062
H	8.808374	-1.200883	-1.100579
C	10.079191	0.245849	-0.113274
H	10.227199	0.882205	-1.005988
H	10.022732	0.942091	0.744556
C	11.293417	-0.670601	0.059920
H	11.142976	-1.305807	0.952052
H	11.347340	-1.365752	-0.797909
C	12.613693	0.090347	0.188035
H	12.805584	0.707620	-0.705970
H	13.468423	-0.594071	0.311359
H	12.599491	0.768167	1.058353
C	-3.696539	0.173872	-0.837213
H	-3.722378	0.855762	-1.709155
H	-3.523987	0.795445	0.062320
C	-5.007171	-0.584561	-0.714020
H	-4.932030	-1.274117	0.144692
H	-5.133635	-1.216054	-1.610319
C	-6.213591	0.339971	-0.544190
H	-6.270679	1.034049	-1.403107
H	-6.067851	0.976172	0.348538
C	-7.539959	-0.412561	-0.415140
H	-7.686008	-1.048587	-1.307772
H	-7.482023	-1.107814	0.442760
C	-8.752198	0.505137	-0.242705
H	-8.808375	1.200702	-1.100842
H	-8.604154	1.141456	0.649810
C	-10.079194	-0.245849	-0.113276
H	-10.227194	-0.882376	-1.005870
H	-10.022739	-0.941929	0.744686
C	-11.293423	0.670631	0.059735
H	-11.347343	1.365617	-0.798227
H	-11.142988	1.306008	0.951747
C	-12.613698	-0.090296	0.187989
H	-12.805583	-0.707740	-0.705898
H	-13.468430	0.594144	0.311178
H	-12.599499	-0.767949	1.058437

C	-1.338324	-0.375717	2.073368	H	-1.775945	1.731032	2.009993
C	-0.354126	-1.366243	1.909062	C	-0.691447	-2.635426	1.181726
C	0.986430	-0.976978	2.073786	H	-0.942487	-3.554476	1.724802
H	1.775943	-1.730777	2.010231	C	0.691451	2.635571	1.181382
C	1.338322	0.375980	2.073327	H	0.942493	3.554693	1.724337
C	0.354125	1.366485	1.908886	H	2.393750	0.651904	2.004652
C	-0.986432	0.977241	2.073652	H	-2.393751	-0.651649	2.004723

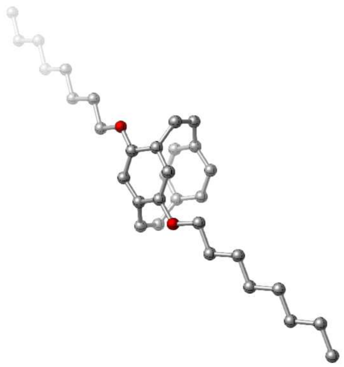

Zero-point correction= 0.687600 (Hartree/Particle)

Thermal correction to Energy= 0.723129

Thermal correction to Enthalpy= 0.724073

Thermal correction to Gibbs Free Energy= 0.614960

Sum of electronic and zero-point Energies= -1394.668663

Sum of electronic and thermal Energies= -1394.633135

Sum of electronic and thermal Enthalpies= -1394.632190

Sum of electronic and thermal Free Energies= -1394.741304

M06L-D3/def2tzvpp-SMD(benzene).

E(scf) = -1396.61457513 a.u.

Ru-1a-M (O^nOct)

E(scf) = -4867.13812014 a.u.

ν_{min} = 6.02 cm^{-1}

S	-2.487095	-2.313067	-0.366511	Ru	-0.620522	-0.971864	-0.313540
C	0.464532	-2.740231	-0.270989	C	1.334420	1.328079	-1.758987

C	-0.569176	-0.585283	1.494341
S	-1.944141	0.714835	-1.290814
N	0.596703	-3.653670	0.716399
N	0.932698	-3.286511	-1.417786
C	0.523326	-3.438920	2.134539
C	1.127856	-4.944666	0.231049
C	1.290847	-4.706670	-1.277983
C	1.217284	-2.550941	-2.620496
H	2.314353	-4.887378	-1.633907
H	0.605922	-5.325404	-1.878121
H	2.075852	-5.180982	0.736881
C	0.562334	-3.312230	4.922065
C	-0.538548	-4.013132	2.866402
C	1.587869	-2.758358	2.782190
C	1.584183	-2.715506	4.182560
C	-0.493635	-3.938809	4.267106
H	2.393722	-2.204607	4.706414
H	-1.305220	-4.377324	4.851350
C	1.790214	-1.071065	-4.911074
C	2.509106	-1.985825	-2.771830
C	0.235058	-2.441970	-3.631324
C	0.542401	-1.674736	-4.765270
C	2.768967	-1.243704	-3.932020
C	3.635035	-2.242839	-1.774134
C	-1.086936	-3.190623	-3.552100
H	-0.205419	-1.558877	-5.551894
H	3.752833	-0.791524	-4.071740
H	0.417128	-5.752507	0.453862
H	3.189828	-2.660029	-0.861119
H	-1.236140	-3.495020	-2.508021
C	-1.721262	-4.694648	2.192576
H	-1.628750	-4.529083	1.109935
C	2.727968	-2.098851	2.011045
H	2.355406	-1.869993	1.003664

C	-3.811558	-1.303784	-0.961235
C	-5.121067	-1.818231	-1.050592
C	-3.583158	0.047687	-1.331044
C	-6.193394	-1.032608	-1.469302
C	-4.685790	0.830710	-1.731309
C	-5.975777	0.305675	-1.805889
H	-7.193302	-1.464971	-1.524603
H	-6.802973	0.939689	-2.126819
H	1.291334	0.607456	-2.580920
C	1.394403	0.873867	-0.480288
H	1.495403	-0.219224	-0.312854
H	0.431290	-0.362285	1.902232
C	-1.608509	-0.401415	2.502686
C	-2.961202	-0.132385	2.196182
C	-1.233689	-0.447357	3.866313
C	-3.899854	0.052741	3.210378
H	-3.266998	-0.031862	1.160051
C	-2.178130	-0.288379	4.878621
H	-0.189549	-0.634743	4.122125
C	-3.517704	-0.038806	4.554154
H	-4.937078	0.274288	2.947312
H	-1.868786	-0.351663	5.924975
H	-4.258236	0.098108	5.346401
H	0.581949	-3.269294	6.013961
H	2.008169	-0.475339	-5.801030
Cl	-5.433012	-3.499743	-0.637197
Cl	-4.461874	2.524091	-2.155353
C	-1.720461	-6.212415	2.447077
H	-1.844227	-6.433951	3.519955
H	-2.552253	-6.693121	1.907225
H	-0.781181	-6.685940	2.119429
C	-3.055489	-4.066211	2.631016
H	-3.037217	-2.975150	2.505763
H	-3.879933	-4.454961	2.013641

H	-3.281570	-4.294581	3.685420
C	3.926706	-3.052542	1.858955
H	4.324987	-3.331935	2.847728
H	3.654878	-3.979947	1.334517
H	4.735476	-2.568751	1.289547
C	3.186771	-0.764951	2.620916
H	3.743549	-0.912637	3.559935
H	3.844745	-0.235144	1.917557
H	2.337665	-0.097478	2.831229
C	4.608480	-3.296226	-2.340145
H	5.368265	-3.564226	-1.588191
H	4.089469	-4.217125	-2.647667
H	5.131520	-2.902592	-3.226883
C	4.408005	-0.981124	-1.363926
H	4.927142	-0.525513	-2.222317
H	3.763644	-0.213713	-0.917666
H	5.181426	-1.238565	-0.624022
C	-1.023630	-4.465873	-4.413827
H	-1.957011	-5.043549	-4.314083
H	-0.891340	-4.215399	-5.479478
H	-0.184831	-5.117034	-4.120002
C	-2.294381	-2.324763	-3.937786
H	-3.228630	-2.853034	-3.693161
H	-2.292368	-1.375281	-3.385265
H	-2.310356	-2.099457	-5.016474
C	0.116667	3.524159	-1.913959
C	1.323213	2.807151	-2.003317
C	2.522239	3.537110	-1.932705
C	2.515730	4.858270	-1.473453
C	1.314984	5.466844	-1.069550
C	0.114594	4.841246	-1.448571
C	1.299003	6.462840	0.053183
H	1.252545	7.541156	-0.140778
C	2.566141	3.854843	1.439928

C	1.339226	4.528551	1.573771
C	0.158277	3.760105	1.524889
C	0.237659	2.438459	1.068766
C	1.440497	1.862446	0.650442
C	2.635177	2.531420	0.978198
C	1.288101	6.008466	1.327429
H	1.221118	6.720501	2.158296
H	3.468128	5.346150	-1.248979
H	-0.833917	2.998998	-2.021247
H	3.477058	3.018093	-2.048929
H	-0.840101	5.310314	-1.197244
O	3.798637	1.909719	0.627116
O	-1.037053	4.390730	1.698235
H	3.475677	4.452451	1.490117
H	-0.676125	1.913019	0.815747
C	5.037098	2.580705	0.792749
H	5.167314	2.883509	1.849460
H	5.057365	3.502378	0.180287
C	6.148444	1.636187	0.368456
H	6.066413	0.708286	0.960164
H	5.984904	1.347631	-0.683141
C	7.541850	2.242868	0.536869
H	7.690071	2.541440	1.591115
H	7.614051	3.174819	-0.053773
C	8.665505	1.291251	0.119559
H	8.586786	0.357140	0.706320
H	8.519273	0.995561	-0.935936
C	10.066859	1.880657	0.292326
H	10.208897	2.179334	1.347737
H	10.145787	2.813524	-0.296516
C	11.190628	0.926572	-0.118078
H	11.109407	-0.007263	0.469534
H	11.049906	0.628900	-1.174224
C	12.593917	1.512916	0.058462

H	12.673000	2.445577	-0.529594
H	12.731071	1.811074	1.114047
C	13.709038	0.550470	-0.352258
H	13.615616	0.262458	-1.413105
H	14.705912	0.999171	-0.214358
H	13.674146	-0.376869	0.244352
C	-2.210294	3.604976	1.496110
H	-2.255525	2.805498	2.260264
H	-2.168313	3.106232	0.513172
C	-3.468458	4.449609	1.535981
H	-3.508790	5.046270	2.462719
H	-3.442961	5.164504	0.695288
C	-4.685514	3.517993	1.412255
H	-4.916216	3.077156	2.398484
H	-4.410614	2.666147	0.765757
C	-5.937733	4.157662	0.815303
H	-6.270217	5.011822	1.433957
H	-5.678643	4.574420	-0.174579
C	-7.080027	3.153125	0.645423
H	-7.426028	2.813732	1.639729
H	-6.686003	2.252963	0.140969
C	-8.266839	3.685521	-0.158319
H	-7.907615	4.011235	-1.152699
H	-8.672300	4.592424	0.328616
C	-9.389537	2.660767	-0.342348
H	-9.751380	2.336084	0.650428
H	-8.973234	1.754298	-0.819443
C	-10.562092	3.185313	-1.171754
H	-11.352236	2.425991	-1.288340
H	-11.017634	4.072607	-0.700172
H	-10.232485	3.483295	-2.181578

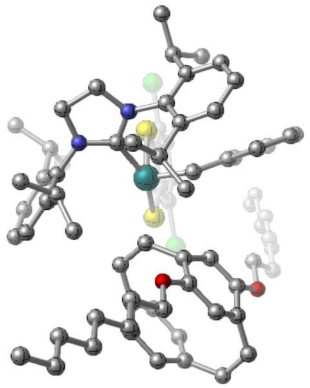

Zero-point correction= 1.456416 (Hartree/Particle)

Thermal correction to Energy= 1.541627

Thermal correction to Enthalpy= 1.542571

Thermal correction to Gibbs Free Energy= 1.327724

Sum of electronic and zero-point Energies= -4865.681704

Sum of electronic and thermal Energies= -4865.596493

Sum of electronic and thermal Enthalpies= -4865.595549

Sum of electronic and thermal Free Energies= -4865.810396

M06L-D3/def2tzvpp-SDD(Ru)-SMD(benzene).

E(scf) = -4870.39067194 a.u.

Ru-1a-ent-M (O^nOct)

E(scf) = -4867.13014688 a.u.

ν_{min} = 3.08 cm^{-1}

S	-2.232702	2.186730	0.369229
C	0.455000	2.008101	1.246714
Ru	-0.421932	0.899292	-0.329025
C	1.470932	-0.665009	-0.011326
C	0.107698	2.006652	-1.740270
S	-2.061612	-0.542985	-1.253260
N	0.720825	3.339973	1.272444
N	0.396400	1.581291	2.536762
C	1.125172	4.192159	0.189616
C	0.886493	3.858859	2.647076
C	0.361825	2.702830	3.490774
C	0.467291	0.228303	3.024076
H	0.976471	2.492618	4.374022
H	-0.677991	2.866163	3.820174
H	1.948340	4.083771	2.837707
C	2.038250	6.034610	-1.704426
C	0.303613	5.287012	-0.165965
C	2.401658	4.005630	-0.404543
C	2.832075	4.942513	-1.353557
C	0.786219	6.196248	-1.119501
H	3.810510	4.822889	-1.821662
H	0.162904	7.044388	-1.409851
C	0.696360	-2.388409	3.960078
C	1.750570	-0.283109	3.346918
C	-0.705976	-0.517593	3.273429
C	-0.560376	-1.838887	3.724045
C	1.838878	-1.606954	3.794457
C	3.000280	0.595591	3.342821
C	-2.091863	0.105534	3.198387
H	-1.451971	-2.442724	3.903056
H	2.817649	-2.031774	4.025808
H	0.313324	4.782774	2.787582
H	2.763438	1.523208	2.804041
H	-2.010293	1.035691	2.623321
C	-1.068399	5.527423	0.451644
H	-1.305775	4.666770	1.090810
C	3.334212	2.866799	-0.003192
H	2.715392	2.050439	0.393248
C	-3.708602	1.366752	-0.128234
C	-4.977009	1.886613	0.200179
C	-3.632818	0.170332	-0.881539
C	-6.152771	1.252010	-0.196911
C	-4.833395	-0.442735	-1.296756
C	-6.080425	0.080316	-0.955688
H	-7.118544	1.674193	0.083848
H	-6.988712	-0.425535	-1.285168
H	2.025228	-0.001409	0.649654
C	1.561839	-0.414672	-1.378418
H	2.138322	0.456173	-1.690830
H	1.185735	2.138772	-1.912489
C	-0.654220	2.777321	-2.715827
C	-2.036528	2.618358	-2.969850

C	0.047790	3.754718	-3.463865
C	-2.686165	3.421072	-3.905525
H	-2.593394	1.849218	-2.445631
C	-0.608183	4.571730	-4.381440
H	1.117850	3.883699	-3.291991
C	-1.981183	4.409115	-4.603766
H	-3.754025	3.277546	-4.087434
H	-0.048800	5.335879	-4.926830
H	-2.498626	5.044205	-5.327514
H	2.395633	6.754943	-2.444567
H	0.786877	-3.425309	4.292736
Cl	-5.103822	3.357637	1.157008
Cl	-4.790258	-1.907839	-2.269195
C	-1.076121	6.794407	1.326330
H	-0.893439	7.696801	0.720145
H	-2.054980	6.914172	1.818040
H	-0.301828	6.763855	2.109569
C	-2.171943	5.600117	-0.616961
H	-2.142603	4.722460	-1.274795
H	-3.161920	5.619532	-0.135078
H	-2.075862	6.505448	-1.238253
C	4.295579	3.321251	1.110990
H	4.917201	4.160871	0.760419
H	3.758636	3.658109	2.008936
H	4.964567	2.499006	1.404819
C	4.141038	2.287306	-1.177237
H	4.951484	2.963826	-1.491328
H	4.606516	1.335260	-0.877607
H	3.510384	2.096578	-2.058547
C	3.374774	0.976773	4.789380
H	4.207869	1.698430	4.796838
H	2.528974	1.423322	5.334261
H	3.692546	0.084757	5.353340
C	4.207047	-0.033405	2.637265
H	4.508483	-0.980434	3.111244
H	3.995772	-0.255114	1.585261
H	5.072336	0.645625	2.689622
C	-2.577029	0.471932	4.613664
H	-3.545733	0.995533	4.561944
H	-2.709242	-0.430157	5.234112
H	-1.860090	1.129095	5.131495
C	-3.121992	-0.773079	2.477242
H	-4.071985	-0.228994	2.363965
H	-2.776495	-1.044317	1.471471
H	-3.336535	-1.701519	3.030356
C	0.039068	-2.727470	0.477851
C	1.278065	-2.089712	0.431101
C	2.432075	-2.904628	0.419230
C	2.299744	-4.272294	0.167457
C	1.048316	-4.857136	-0.085648
C	-0.100211	-4.103517	0.225980
C	1.001648	-6.009147	-1.045719
H	0.808674	-7.032437	-0.702829
C	2.590810	-3.675200	-2.824127
C	1.355748	-4.350839	-2.832008
C	0.203692	-3.558010	-2.940176
H	-0.776543	-4.034656	-3.023224
C	0.262058	-2.182671	-2.710789
C	1.464154	-1.566448	-2.342722
C	2.647815	-2.297517	-2.583168
H	3.618672	-1.833045	-2.391762
C	1.204148	-5.769400	-2.362556
H	1.192104	-6.603234	-3.074998
H	-0.668625	-1.625141	-2.666932
H	3.520484	-4.250803	-2.828953
H	3.185652	-4.864006	-0.058248
H	-0.845479	-2.102766	0.520726
O	3.635348	-2.257949	0.420696

O	-1.316093	-4.700785	0.072245
C	4.831630	-2.974955	0.168927
H	4.755883	-3.516813	-0.791695
C	-2.465491	-3.937999	0.429453
H	-2.402231	-3.663104	1.499652
H	-2.495426	-3.000599	-0.148968
H	4.999299	-3.730570	0.960489
C	5.983536	-1.984602	0.117134
H	5.736996	-1.203155	-0.623426
H	6.066965	-1.475051	1.090638
C	7.315071	-2.646719	-0.239685
H	7.227751	-3.148349	-1.221091
H	7.535342	-3.447755	0.490082
C	8.486854	-1.663099	-0.276803
H	8.571293	-1.160802	0.704801
H	8.266356	-0.861599	-1.006120
C	9.825483	-2.315345	-0.628646
H	9.740491	-2.815786	-1.611254
H	10.041611	-3.119288	0.099536
C	10.999114	-1.334001	-0.660621
H	11.082716	-0.832590	0.321990
H	10.783657	-0.530213	-1.389562
C	12.339705	-1.986018	-1.009776
H	12.253858	-2.486165	-1.991809
H	12.551581	-2.789799	-0.281063
C	13.505869	-0.997195	-1.037040
H	14.455909	-1.494435	-1.290594
H	13.335613	-0.201343	-1.781829
H	13.635627	-0.507739	-0.056878
C	-3.739386	-4.711357	0.151014
H	-3.825349	-4.869202	-0.937748
H	-3.692146	-5.709824	0.618312
C	-4.946081	-3.916094	0.666516
H	-4.986121	-3.979659	1.769346
H	-4.791528	-2.847933	0.435274
C	-6.289401	-4.338327	0.073085
H	-6.499361	-5.396092	0.318139
H	-6.220695	-4.283433	-1.027942
C	-7.447469	-3.454258	0.540939
H	-7.553629	-3.533256	1.639125
H	-7.190251	-2.397951	0.340305
C	-8.785417	-3.777271	-0.125815
H	-9.055083	-4.830918	0.076779
H	-8.669913	-3.702789	-1.223589
C	-9.930688	-2.865060	0.321871
H	-9.653200	-1.813961	0.119961
H	-10.045578	-2.938967	1.418868
C	-11.263104	-3.183955	-0.357335
H	-11.580615	-4.218836	-0.144313
H	-12.066581	-2.511005	-0.016714
H	-11.184732	-3.083758	-1.453232

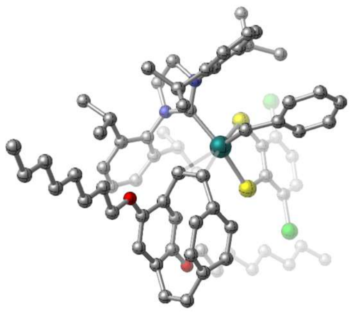

Zero-point correction= 1.458057 (Hartree/Particle)

Thermal correction to Energy= 1.542585

Thermal correction to Enthalpy= 1.543529

Thermal correction to Gibbs Free Energy= 1.330190

Sum of electronic and zero-point Energies= -4865.672090

Sum of electronic and thermal Energies= -4865.587562

Sum of electronic and thermal Enthalpies= -4865.586618

Sum of electronic and thermal Free Energies= -4865.799957

M06L-D3/def2tzvpp-SDD(Ru)-SMD(benzene).

E(scf) = -4870.38389977 a.u.

Int1-α1 (O^nOct)

E(scf) = -4867.19227002 a.u.

ν_{min} = 5.61 cm^{-1}

S	3.386890	-0.582964	0.422331	C	3.533479	-5.609452	-2.449203
C	1.845619	-3.103469	0.915098	C	5.540375	-4.635533	-1.521990
Ru	1.782525	-1.772061	-0.669642	H	3.037975	-6.147770	-3.260088
C	0.191902	-0.893692	-0.425067	H	6.605217	-4.413461	-1.613488
C	-8.330859	2.267644	-1.875771	C	-2.006317	-0.717060	3.317989
S	2.680016	-1.002370	-2.685599	C	-1.184777	-2.757913	2.282552
N	2.733370	-4.122481	0.873114	C	0.395080	-1.106850	3.227881
N	1.215489	-3.142771	2.105768	C	-0.700854	-0.317530	3.601382
C	3.465083	-4.515705	-0.298055	C	-2.244930	-1.931076	2.677547
C	2.631694	-5.011904	2.041873	C	-1.463455	-4.104440	1.618682
C	1.769000	-4.181401	2.997849	C	1.806252	-0.704882	3.637278
C	0.130993	-2.308903	2.533815	H	-0.531363	0.628084	4.119188
H	0.962640	-4.757037	3.473754	H	-3.272347	-2.236074	2.473020
H	2.367665	-3.705740	3.790345	H	3.624045	-5.242889	2.449940
H	2.150388	-5.959935	1.745348	H	-0.539662	-4.699789	1.668451
C	4.894757	-5.324951	-2.547296	H	2.510570	-1.261490	3.002117
C	4.844664	-4.215875	-0.379415	C	5.578477	-3.472971	0.728572
C	2.790695	-5.213878	-1.328602	H	4.816706	-2.990542	1.357531

C	1.306739	-5.556159	-1.255842	H	5.954640	-1.729211	-0.541395
H	0.883738	-5.067323	-0.367925	H	6.823981	-1.707377	1.006327
C	4.230103	0.351107	-0.820391	H	7.385510	-2.767809	-0.305053
C	5.250314	1.253294	-0.461991	C	1.092823	-7.070465	-1.085778
C	3.894127	0.197362	-2.189713	H	1.467411	-7.625613	-1.961114
C	5.925603	2.015642	-1.411085	H	1.619328	-7.454074	-0.197434
C	4.578094	0.991195	-3.134875	H	0.020350	-7.299139	-0.974188
C	5.579488	1.887872	-2.758511	C	0.531581	-5.022292	-2.471808
H	6.707497	2.708541	-1.099133	H	0.857091	-5.507350	-3.405435
H	6.086806	2.480845	-3.520499	H	-0.545829	-5.214479	-2.354676
H	-0.625084	-1.533994	-0.823833	H	0.674629	-3.937402	-2.596455
C	-6.995661	2.448254	-2.007273	C	-2.548074	-4.902442	2.360422
H	-6.551474	1.972702	-2.889451	H	-2.625319	-5.918548	1.941452
H	-8.789546	1.607815	-2.621714	H	-2.321375	-4.988963	3.434784
C	-9.290018	2.814018	-0.896132	H	-3.540054	-4.433304	2.261851
C	-10.347301	2.000942	-0.440300	C	-1.814289	-3.957948	0.129709
C	-9.235930	4.148371	-0.446170	H	-2.677910	-3.292385	-0.010308
C	-11.291081	2.489789	0.466214	H	-0.975160	-3.534607	-0.435535
H	-10.421312	0.969898	-0.794397	H	-2.054667	-4.939938	-0.308191
C	-10.184317	4.639667	0.452059	C	2.066418	-1.108111	5.102624
H	-8.443904	4.803987	-0.813406	H	3.108603	-0.887926	5.385871
C	-11.211896	3.810837	0.919224	H	1.403579	-0.545966	5.781003
H	-12.095385	1.837037	0.815464	H	1.881807	-2.179295	5.277512
H	-10.125650	5.679130	0.785298	C	2.100552	0.788823	3.439618
H	-11.952663	4.197005	1.623936	H	3.172371	0.985455	3.595932
H	5.455544	-5.639341	-3.431137	H	1.845383	1.111956	2.423496
H	-2.844575	-0.076905	3.603994	H	1.539512	1.413108	4.153853
Cl	5.698422	1.431899	1.233475	C	0.652570	1.491436	0.156241
Cl	4.193621	0.863344	-4.843143	C	-0.249430	0.417601	-0.004957
C	6.385919	-4.447795	1.605802	C	-1.627596	0.663642	0.252599
H	7.172173	-4.945172	1.014090	C	-2.022009	1.914050	0.727174
H	6.875108	-3.908674	2.433417	C	-1.124763	2.984638	0.876375
H	5.752799	-5.237282	2.040121	C	0.242178	2.754558	0.576885
C	6.487042	-2.358269	0.184592	C	-1.573571	4.300442	1.355537

H	-0.823908	4.872284	1.910532
C	-5.158262	4.002665	0.959434
C	-3.910287	4.310346	0.384664
C	-3.734111	4.036970	-0.983819
C	-4.753611	3.458096	-1.734652
C	-6.000925	3.128760	-1.161041
C	-6.173911	3.408723	0.211206
C	-2.798494	4.853232	1.202194
H	-2.999071	5.795142	1.727203
H	-3.058488	2.083253	0.994595
H	1.695580	1.297296	-0.068647
H	-5.318509	4.192649	2.024270
H	-4.580932	3.243768	-2.793055
H	-7.106353	3.137565	0.705865
H	-2.772605	4.260399	-1.451166
O	-2.512002	-0.346681	0.033547
O	1.088332	3.807535	0.722848
C	-3.897203	-0.059661	0.183707
H	-4.119709	0.164714	1.243616
H	-4.157067	0.840309	-0.402648
C	-4.737588	-1.232347	-0.287041
H	-4.431844	-1.503787	-1.312063
H	-4.542644	-2.113612	0.346785
C	-6.224225	-0.870129	-0.253485
H	-6.504114	-0.566715	0.772513
H	-6.392319	0.023629	-0.878702
C	-7.164397	-1.982766	-0.718726
H	-6.856766	-2.328803	-1.722907
H	-7.066383	-2.860021	-0.052528
C	-8.624166	-1.525383	-0.765529
H	-8.700286	-0.661189	-1.449356
H	-8.917597	-1.140522	0.228921
C	-9.616396	-2.602833	-1.202850
H	-9.317357	-2.998781	-2.191518
H	-9.560591	-3.460329	-0.506240
C	-11.061981	-2.101889	-1.275769
H	-11.113602	-1.251259	-1.980266
H	-11.352944	-1.693130	-0.290607
C	-12.060260	-3.179407	-1.700529
H	-13.088978	-2.787194	-1.745439
H	-11.811395	-3.581237	-2.697272
H	-12.055433	-4.026538	-0.993941
C	2.457587	3.656872	0.356677
H	2.935598	2.868022	0.965366
H	2.531514	3.340074	-0.700588
C	3.154035	4.990231	0.564085
H	2.616772	5.762479	-0.013558
H	3.067180	5.275148	1.627231
C	4.624138	4.939871	0.146971
H	5.136117	4.144803	0.715093
H	4.692124	4.636861	-0.913066
C	5.371841	6.258656	0.343865
H	4.871039	7.058560	-0.232700
H	5.304887	6.564487	1.404604
C	6.843722	6.178520	-0.068338
H	7.337810	5.373354	0.507212
H	6.907875	5.870145	-1.128723
C	7.614450	7.485721	0.125832
H	7.549882	7.793580	1.186479
H	7.119941	8.290503	-0.450062
C	9.086653	7.401816	-0.286205
H	9.148633	7.093966	-1.346122
H	9.577888	6.595979	0.289449
C	9.848095	8.712945	-0.086904
H	9.830924	9.026228	0.970805
H	10.903225	8.622351	-0.391549
H	9.397925	9.528332	-0.678204

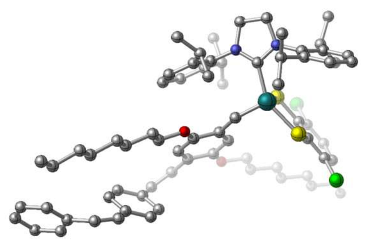

Zero-point correction= 1.457731 (Hartree/Particle)

Thermal correction to Energy= 1.543613

Thermal correction to Enthalpy= 1.544557

Thermal correction to Gibbs Free Energy= 1.323993

Sum of electronic and zero-point Energies= -4865.734539

Sum of electronic and thermal Energies= -4865.648657

Sum of electronic and thermal Enthalpies= -4865.647713

Sum of electronic and thermal Free Energies= -4865.868277

M06L-D3/def2tzvpp-SDD(Ru)-SMD(benzene).

E(scf) = -4870.45388672 a.u.

Int1-β1 (O^nOct)

E(scf) = -4867.19160325 a.u.

ν_{min} = 6.07 cm^{-1}

S	3.915484	-0.951745	1.934129
C	4.125918	-2.014995	-0.910349
Ru	3.749956	-0.128471	-0.180849
C	1.953564	0.064550	-0.440164
C	-7.994560	1.772221	-3.039894
S	4.605375	1.894636	0.585671
N	5.413314	-2.395596	-1.068967
N	3.328075	-3.031844	-1.281534
C	6.508041	-1.464079	-1.019516
C	5.542971	-3.730618	-1.675383
C	4.091892	-4.237104	-1.662791
C	1.897787	-3.035719	-1.369960
H	3.755073	-4.613149	-2.639496
H	3.927970	-5.029281	-0.915642
H	5.953999	-3.639721	-2.694597
C	8.565797	0.406276	-0.902710
C	7.347663	-1.440349	0.117421
C	6.698669	-0.579936	-2.110139
C	7.740092	0.353352	-2.024689
C	8.374710	-0.486589	0.151113
H	7.903485	1.054868	-2.845596
H	9.031300	-0.434709	1.021148
C	-0.867627	-3.208646	-1.635160
C	1.305123	-2.714097	-2.612726

C	1.127617	-3.432146	-0.254996
C	-0.262879	-3.512814	-0.416420
C	-0.090014	-2.804885	-2.720184
C	2.133093	-2.310248	-3.828026
C	1.763901	-3.797437	1.079217
H	-0.883736	-3.814162	0.429317
H	-0.573838	-2.561500	-3.669070
H	6.220136	-4.365315	-1.088504
H	3.188475	-2.287974	-3.523923
H	2.809080	-3.457763	1.055973
C	7.172226	-2.424451	1.265185
H	6.136613	-2.791716	1.220130
C	5.837412	-0.629250	-3.368088
H	5.007828	-1.325809	-3.188545
C	4.444154	0.413731	2.930665
C	4.605992	0.261458	4.322025
C	4.725130	1.671753	2.340707
C	5.014095	1.318004	5.133165
C	5.123861	2.731973	3.180935
C	5.270824	2.565379	4.558026
H	5.128045	1.164577	6.207122
H	5.586810	3.407646	5.174766
H	1.735073	0.194927	-1.524972
C	-6.706996	1.548932	-2.686220
H	-6.001882	1.456828	-3.521616
H	-8.194958	1.747307	-4.117571
C	-9.181205	2.072222	-2.224588
C	-10.457196	1.785635	-2.750454
C	-9.114054	2.671305	-0.949730
C	-11.618165	2.040057	-2.015974
H	-10.535102	1.339936	-3.746169
C	-10.271837	2.933164	-0.218229
H	-8.142294	2.937793	-0.532055
C	-11.530665	2.609583	-0.741522

H	-12.594636	1.791912	-2.439896
H	-10.193133	3.395193	0.769407
H	-12.436130	2.809028	-0.162974
H	9.365459	1.149253	-0.849234
H	-1.953822	-3.274789	-1.736276
Cl	4.302408	-1.302701	5.068256
Cl	5.465449	4.318064	2.501653
C	8.121537	-3.626605	1.098466
H	9.173436	-3.300661	1.152773
H	7.953729	-4.366066	1.898402
H	7.984703	-4.132932	0.130408
C	7.365524	-1.775989	2.644538
H	6.800906	-0.838093	2.727552
H	7.007471	-2.452123	3.436050
H	8.426986	-1.560190	2.848468
C	6.641141	-1.178449	-4.560416
H	7.474911	-0.505316	-4.817749
H	7.069538	-2.168343	-4.337015
H	5.997337	-1.275186	-5.449878
C	5.207240	0.729526	-3.711832
H	5.969210	1.479323	-3.975498
H	4.527377	0.628346	-4.571988
H	4.630116	1.138338	-2.869493
C	2.005364	-3.344510	-4.960132
H	2.672505	-3.084956	-5.797982
H	2.268421	-4.356418	-4.613277
H	0.975822	-3.384410	-5.351071
C	1.785797	-0.898628	-4.328938
H	0.731909	-0.827598	-4.641797
H	1.959125	-0.141576	-3.549955
H	2.410781	-0.635611	-5.197128
C	1.770231	-5.324113	1.280849
H	2.279096	-5.586816	2.222371
H	0.741030	-5.716294	1.330969

H	2.283214	-5.845862	0.457395
C	1.087053	-3.092055	2.264719
H	1.665293	-3.265368	3.185492
H	1.035167	-2.007395	2.103681
H	0.065670	-3.469927	2.433588
C	0.872743	0.705702	1.742418
C	0.790690	0.224060	0.417093
C	-0.490476	-0.084623	-0.094932
C	-1.633177	0.082167	0.673424
C	-1.560878	0.617543	1.976889
C	-0.278872	0.892112	2.499911
C	-2.740795	0.902645	2.798470
H	-2.541511	0.911475	3.876474
C	-5.931001	0.687536	0.944693
C	-4.666760	1.282145	1.121023
C	-4.113084	1.998942	0.030964
C	-4.799453	2.035690	-1.187478
C	-6.062029	1.440068	-1.364312
C	-6.617329	0.728074	-0.272816
C	-4.024492	1.151071	2.442860
H	-4.730841	1.239716	3.277522
H	-2.602989	-0.186454	0.253589
H	1.846075	0.965906	2.155453
H	-0.574855	-0.476607	-1.110117
H	-0.190879	1.292962	3.513407
O	-2.934930	2.639355	0.238379
H	-6.356208	0.139386	1.785701
H	-4.371918	2.581695	-2.028716
C	-2.101888	2.967522	-0.868937
H	-2.582057	3.734645	-1.505082
H	-1.951184	2.064943	-1.490126
C	-0.772334	3.473875	-0.332687
H	-0.911712	4.481253	0.096581
H	-0.464033	2.818821	0.495068
C	0.330039	3.492154	-1.393501
H	-0.013156	4.030846	-2.296782
H	0.528297	2.452832	-1.713321
C	1.630787	4.120688	-0.888905
H	1.464743	5.198154	-0.704000
H	1.894187	3.679636	0.089138
C	2.821613	3.935793	-1.831579
H	2.536740	4.214708	-2.863774
H	3.073084	2.859561	-1.864595
C	4.057078	4.731657	-1.400557
H	4.204364	4.599316	-0.315857
H	3.871072	5.811036	-1.557992
C	5.344925	4.321288	-2.117849
H	5.230488	4.472465	-3.207800
H	5.499304	3.238058	-1.974041
C	6.577494	5.069549	-1.609176
H	7.494098	4.751573	-2.132768
H	6.472946	6.159268	-1.748134
H	6.723471	4.884909	-0.531766
O	-7.796916	0.094933	-0.487201
C	-8.586066	-0.338867	0.611245
H	-8.725563	0.497479	1.322355
H	-8.077946	-1.158661	1.154658
C	-9.925652	-0.803463	0.067339
H	-10.353800	0.012986	-0.533026
H	-9.755220	-1.647390	-0.623526
C	-10.908527	-1.206631	1.166421
H	-10.483639	-2.028858	1.771914
H	-11.046452	-0.357651	1.861581
C	-12.273372	-1.630245	0.618713
H	-12.689135	-0.804715	0.011900
H	-12.139485	-2.477811	-0.078802
C	-13.279217	-2.019971	1.703581
H	-12.861117	-2.844643	2.310696

H	-13.410715	-1.171428	2.400853	H	-15.783357	-1.979596	2.934214
C	-14.644810	-2.438701	1.154574	C	-17.014564	-3.244966	1.677685
H	-15.062380	-1.613756	0.547098	H	-17.718981	-3.519617	2.479311
H	-14.513328	-3.287022	0.456763	H	-17.472449	-2.427275	1.095579
C	-15.653982	-2.828455	2.237979	H	-16.918634	-4.113668	1.004422
H	-15.234184	-3.652235	2.843942				

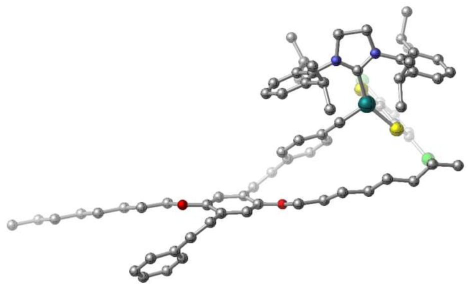

Zero-point correction= 1.457500 (Hartree/Particle)

Thermal correction to Energy= 1.542998

Thermal correction to Enthalpy= 1.543943

Thermal correction to Gibbs Free Energy= 1.326283

Sum of electronic and zero-point Energies= -4865.734104

Sum of electronic and thermal Energies= -4865.648605

Sum of electronic and thermal Enthalpies= -4865.647661

Sum of electronic and thermal Free Energies= -4865.865320

M06L-D3/def2tzvpp-SDD(Ru)-SMD(benzene).

E(scf) = -4870.45287975 a.u.

Int1-α1-ent-M (O^nOct)

E(scf) = -6262.58924570 a.u.

ν_{min} = 6.36 cm^{-1}

S	0.960209	-1.503305	1.210757	C	-0.179966	0.387747	-0.987816
C	0.191367	1.120760	1.915352	S	3.152969	-0.441725	-0.840923
Ru	1.272402	0.574062	0.183824	N	-1.102174	0.838815	2.211387
C	1.882220	2.919183	0.087193	N	0.830463	1.384430	3.088128

C	-2.227529	0.732774	1.325552
C	-1.388128	0.954316	3.657858
C	0.013338	0.997667	4.251871
C	2.089494	2.057265	3.283850
H	0.121114	1.717468	5.071277
H	0.339862	0.006598	4.608682
H	-1.967307	1.871899	3.852284
C	-4.625019	0.613073	-0.103305
C	-2.942468	-0.486267	1.271975
C	-2.693197	1.891471	0.648855
C	-3.892651	1.799522	-0.069162
C	-4.148708	-0.516898	0.555118
H	-4.274240	2.676884	-0.593848
H	-4.723255	-1.441703	0.522386
C	4.480667	3.444011	3.664607
C	2.057158	3.465724	3.452998
C	3.293956	1.329539	3.416081
C	4.483688	2.054432	3.586052
C	3.273554	4.138985	3.621535
C	0.747280	4.240205	3.582920
C	3.316901	-0.188807	3.511960
H	5.429382	1.516200	3.668913
H	3.276133	5.225172	3.730624
H	-1.976435	0.098511	4.009793
H	-0.067215	3.594650	3.227280
H	2.384422	-0.566305	3.074840
C	-2.472605	-1.758866	1.967455
H	-1.509745	-1.546298	2.450701
C	-1.981846	3.237712	0.735818
H	-0.926474	3.046928	0.971205
C	2.291770	-2.544112	0.734906
C	2.439599	-3.835027	1.282341
C	3.255686	-2.081002	-0.192777
C	3.539299	-4.634305	0.983296
C	4.320251	-2.937016	-0.539530
C	4.487771	-4.181617	0.063433
H	3.642291	-5.614482	1.449563
H	5.336723	-4.807107	-0.207620
H	1.161760	3.277050	0.820771
C	1.395509	2.686608	-1.203838
H	0.327624	2.835707	-1.362970
H	-0.808786	1.265898	-1.196437
C	-0.677375	-0.781319	-1.701876
C	0.097666	-1.923961	-2.008379
C	-2.028900	-0.778059	-2.119630
C	-0.465108	-3.009849	-2.667400
H	1.153125	-1.939285	-1.749348
C	-2.604959	-1.895145	-2.718776
C	-1.838330	-3.043093	-2.988670
H	-3.656777	-1.867444	-3.003313
H	-5.568420	0.574899	-0.651868
H	5.421407	3.988041	3.779052
Cl	1.227310	-4.470841	2.389634
Cl	5.480749	-2.448447	-1.768650
C	-3.468815	-2.206501	3.052660
H	-4.438299	-2.492057	2.614598
H	-3.077546	-3.086501	3.588475
H	-3.659605	-1.412617	3.793073
C	-2.223860	-2.896081	0.961651
H	-1.522828	-2.587932	0.177373
H	-1.782784	-3.763641	1.476725
H	-3.156630	-3.222126	0.483747
C	-2.577656	4.089740	1.871997
H	-3.646608	4.281445	1.685331
H	-2.495506	3.591984	2.848697
H	-2.062297	5.059049	1.940544
C	-2.011221	4.035281	-0.578830
H	-3.004851	4.469149	-0.772907

H	-1.293804	4.868863	-0.526967	H	3.289437	1.030185	-2.540942
H	-1.748463	3.411841	-1.446613	H	3.610175	5.851209	-3.597566
C	0.475144	4.564867	5.065726	H	4.741682	6.258559	-0.995478
H	-0.516290	5.031689	5.184254	H	4.297827	1.575977	0.460061
H	0.509913	3.668169	5.702885	C	-2.384244	-4.273272	-3.611455
H	1.230809	5.268886	5.450533	H	-1.889567	-4.577753	-4.542360
C	0.695871	5.524770	2.747863	C	-3.288727	-5.129126	-3.100005
H	1.477580	6.240009	3.048910	H	-3.453067	-6.063558	-3.648996
H	0.837810	5.321015	1.680985	C	-4.000352	-5.026220	-1.804329
H	-0.273090	6.028553	2.888170	C	-3.759175	-5.990161	-0.803649
C	3.345898	-0.617934	4.991636	C	-4.940455	-4.018421	-1.541033
H	3.281308	-1.715431	5.072772	C	-4.417230	-5.899958	0.424595
H	4.281058	-0.293480	5.477948	C	-5.601304	-3.933085	-0.310361
H	2.508814	-0.183298	5.560281	H	-5.149241	-3.289754	-2.319779
C	4.474232	-0.837197	2.740577	C	-5.361402	-4.896898	0.700585
H	4.361504	-1.931860	2.736871	H	-4.204485	-6.658131	1.181996
H	4.490917	-0.503866	1.695380	C	-5.985201	-4.862083	2.036606
H	5.453518	-0.608529	3.190888	H	-5.291654	-5.138451	2.839531
C	4.427668	2.625302	0.219763	C	-7.244400	-4.568363	2.437654
C	3.289882	3.430993	0.220044	H	-7.398670	-4.561082	3.523112
C	3.461084	4.806418	-0.055456	C	-8.461614	-4.279517	1.664797
C	4.674424	5.253415	-0.581689	C	-8.673581	-4.750923	0.352947
C	5.727037	4.366038	-0.857773	C	-9.489412	-3.528235	2.269081
C	5.652812	3.071366	-0.307368	C	-9.846551	-4.447961	-0.337243
C	6.601098	4.678977	-2.036504	H	-7.909968	-5.365318	-0.126259
H	7.638056	5.011061	-1.908674	C	-10.662215	-3.216704	1.576562
C	3.541147	4.779148	-3.393379	H	-9.355676	-3.170112	3.293786
C	4.703153	3.985600	-3.445256	C	-10.843877	-3.670183	0.265962
C	4.528910	2.602870	-3.288040	H	-9.986646	-4.823020	-1.354492
C	3.337339	2.089077	-2.775231	H	-11.437119	-2.617867	2.062046
C	2.297396	2.939944	-2.381830	H	-11.759772	-3.428856	-0.279281
C	2.348978	4.264130	-2.870208	O	-6.450027	-2.921343	0.001240
C	6.085531	4.549991	-3.281687	O	-2.933586	-7.048222	-1.073076
H	6.702451	4.798273	-4.153939	H	-2.641574	0.104856	-1.930416

H	0.162044	-3.870573	-2.907222
C	-1.647824	-7.115623	-0.443162
H	-1.714142	-6.772877	0.605887
H	-1.391568	-8.187275	-0.427312
C	-0.592130	-6.334883	-1.220648
H	-0.721848	-5.254352	-1.044619
H	-0.796265	-6.500053	-2.291146
C	0.846747	-6.761002	-0.918387
H	0.922765	-7.858190	-1.036862
H	1.098984	-6.545131	0.135150
C	1.871325	-6.089408	-1.841569
H	1.980331	-5.027110	-1.559005
H	1.482475	-6.093196	-2.877047
C	3.248083	-6.757951	-1.835054
H	3.141662	-7.820287	-2.123942
H	3.642401	-6.766504	-0.804494
C	4.257445	-6.076218	-2.761356
H	3.913993	-6.169725	-3.808622
H	4.272052	-4.993625	-2.551098
C	5.682872	-6.623247	-2.645512
H	5.686622	-7.700382	-2.893735
H	6.011652	-6.562653	-1.591617
C	6.684017	-5.877998	-3.529133
H	6.399239	-5.938439	-4.593077
H	6.723099	-4.810948	-3.255794
H	7.701449	-6.288779	-3.430388
C	-6.987955	-2.092867	-1.020827
H	-6.179635	-1.532679	-1.526406
H	-7.487595	-2.720183	-1.782160
C	-7.974906	-1.141701	-0.369647
H	-8.749549	-1.737305	0.136161
H	-7.449161	-0.575888	0.418651
C	-8.619217	-0.180624	-1.367981
H	-7.837664	0.418543	-1.872469

H	-9.117222	-0.759968	-2.167249
C	-9.636991	0.759047	-0.717985
H	-10.414271	0.155307	-0.214297
H	-9.138935	1.336143	0.083089
C	-10.300161	1.724444	-1.702104
H	-9.520223	2.325992	-2.205501
H	-10.795567	1.145151	-2.503548
C	-11.320082	2.661555	-1.051676
H	-12.099478	2.059346	-0.547994
H	-10.824636	3.240574	-0.249703
C	-11.985868	3.628418	-2.034538
H	-12.479409	3.047568	-2.835157
H	-11.205008	4.228405	-2.536858
C	-13.003299	4.559392	-1.373739
H	-13.465471	5.242574	-2.104368
H	-13.813740	3.985848	-0.892798
H	-12.528990	5.176601	-0.591946
H	1.520645	4.947949	-2.667259
H	5.386852	1.933667	-3.393004
O	2.334030	5.577591	-0.017569
O	6.719096	2.241388	-0.491569
C	2.357417	6.912130	-0.493350
H	3.041134	7.527314	0.122849
H	2.734693	6.940503	-1.531927
C	0.939324	7.456988	-0.440174
H	0.584867	7.441519	0.603478
H	0.284150	6.767456	-1.001263
C	0.820842	8.870861	-1.010074
H	1.493194	9.550891	-0.455031
H	1.178726	8.879345	-2.056144
C	-0.607151	9.419140	-0.959788
H	-0.964159	9.408510	0.086842
H	-1.278078	8.735414	-1.512363
C	-0.745438	10.833110	-1.527673

H	-0.074485	11.516067	-0.973939
H	-0.386625	10.842941	-2.573781
C	-2.174595	11.377810	-1.478189
H	-2.533653	11.366607	-0.431823
H	-2.845188	10.694137	-2.032065
C	-2.316978	12.792890	-2.045104
H	-1.645813	13.474043	-1.490565
H	-1.957053	12.801861	-3.090230
C	-3.749190	13.326112	-1.990563
H	-4.122129	13.359796	-0.952822
H	-3.820882	14.344696	-2.404629
H	-4.435801	12.682063	-2.565769
C	6.652119	0.949318	0.102915
H	6.533450	1.055759	1.198239
H	5.770086	0.403166	-0.270720
C	7.891880	0.138052	-0.224528
H	7.916138	-0.038232	-1.313545
H	8.800915	0.710058	0.026869
C	7.841512	-1.199650	0.529859
H	8.175646	-1.054382	1.573070
H	6.787699	-1.520233	0.599404
C	8.626097	-2.339945	-0.121154
H	9.708383	-2.114563	-0.126606
H	8.321596	-2.410289	-1.179741
C	8.367005	-3.690900	0.552274
H	8.817749	-3.701013	1.562049
H	7.279312	-3.798256	0.711496
C	8.862018	-4.896219	-0.249018
H	9.955453	-4.824109	-0.399715
H	8.414524	-4.862452	-1.259293
C	8.522936	-6.241183	0.399935
H	8.969423	-6.282216	1.410224
H	7.429206	-6.298364	0.554378
C	8.985169	-7.446739	-0.418969
H	10.077262	-7.428135	-0.573338
H	8.733111	-8.397787	0.077284
H	8.511269	-7.454009	-1.414476

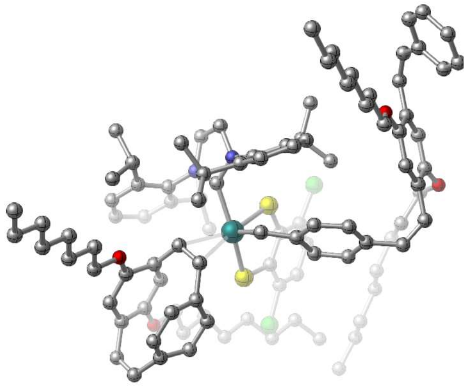

Zero-point correction= 2.152150 (Hartree/Particle)

Thermal correction to Energy= 2.272662

Thermal correction to Enthalpy= 2.273607

Thermal correction to Gibbs Free Energy= 1.984075

Sum of electronic and zero-point Energies= -6260.437096

Sum of electronic and thermal Energies= -6260.316583

Sum of electronic and thermal Enthalpies= -6260.315639

Sum of electronic and thermal Free Energies= -6260.605171

M06L-D3/def2tzvpp-SDD(Ru)-SMD(benzene).

E(scf) = -6267.08579103 a.u.

Int1-β1-M (O^nOct)

E(scf) = -6262.59421631 a.u.

ν_{min} = 4.62 cm^{-1}

S	2.087522	-1.080914	-2.035868	C	-2.419173	-4.083035	-3.365632
C	-0.839080	-1.253194	-2.665716	C	-0.014855	-4.283504	-3.908958
Ru	0.218534	-1.949440	-1.019365	C	-0.133113	-5.676103	-3.782851
C	-1.473069	-4.600250	0.142540	C	-2.485641	-5.479626	-3.268254
C	-0.202002	-0.571900	0.138805	C	-3.698365	-3.258183	-3.254192
S	1.675082	-3.341732	0.205025	C	1.292973	-3.669034	-4.385110
N	-1.151802	0.011674	-3.024430	H	0.741748	-6.305508	-3.955969
N	-1.079142	-2.063224	-3.724406	H	-3.440166	-5.957841	-3.039754
C	-1.333073	1.154400	-2.172239	H	-0.964894	0.832795	-4.973951
C	-1.592120	0.112120	-4.431625	H	-3.413715	-2.201714	-3.158014
C	-1.421434	-1.323621	-4.949155	H	1.255753	-2.593842	-4.167305
C	-1.160972	-3.496689	-3.655280	C	0.876254	2.132913	-3.052534
H	-2.333572	-1.726906	-5.409073	H	0.966500	1.106192	-3.434777
H	-0.601248	-1.412979	-5.679084	C	-3.506651	0.093886	-1.264226
H	-2.632413	0.467533	-4.479588	H	-2.975119	-0.834464	-1.513564
C	-1.846160	3.505034	-0.757607	C	3.482739	-1.845541	-1.263423
C	-0.406298	2.216764	-2.237814	C	4.796303	-1.501000	-1.640245
C	-2.503890	1.240094	-1.373189	C	3.302086	-2.803350	-0.230312
C	-2.737801	2.434338	-0.678060	C	5.912259	-2.049451	-1.011465
C	-0.687795	3.388806	-1.519339	C	4.443954	-3.314465	0.420777
H	-3.632355	2.529282	-0.060034	C	5.735574	-2.952458	0.039094
H	0.010063	4.225029	-1.553107	H	6.912534	-1.768462	-1.336425
C	-1.351022	-6.269562	-3.455313	H	6.596557	-3.375300	0.557736

H	-1.263220	-4.966126	-0.867017
C	-1.775544	-3.288384	0.323427
H	-1.891807	-2.645729	-0.573510
H	-1.272447	-0.487577	0.388611
C	0.591955	0.367634	0.917191
C	1.977560	0.231827	1.159031
C	-0.080543	1.444365	1.540564
C	2.650304	1.129255	1.983678
H	2.518910	-0.605593	0.731285
C	0.601825	2.361043	2.331027
C	1.981220	2.218765	2.570628
H	0.062227	3.191306	2.789085
H	-2.038899	4.432383	-0.215604
H	-1.421041	-7.356007	-3.359572
Cl	5.061457	-0.358847	-2.956288
Cl	4.267784	-4.446747	1.755816
C	0.840166	3.092390	-4.255540
H	0.764516	4.139931	-3.921411
H	1.759910	2.994586	-4.854804
H	-0.020356	2.895857	-4.914852
C	2.110246	2.403396	-2.176202
H	2.113063	1.759931	-1.286110
H	3.033195	2.192566	-2.738796
H	2.141271	3.450359	-1.842661
C	-4.656967	0.256746	-2.274322
H	-5.214830	1.186246	-2.076243
H	-4.295174	0.300262	-3.311717
H	-5.360905	-0.586684	-2.198205
C	-4.072869	-0.091782	0.152805
H	-4.773066	0.714537	0.423197
H	-4.613992	-1.046479	0.221696
H	-3.277253	-0.118433	0.912265
C	-4.531747	-3.399315	-4.543651
H	-5.408086	-2.731769	-4.512574
H	-3.946573	-3.156186	-5.443885
H	-4.895815	-4.433320	-4.658112
C	-4.559052	-3.606753	-2.031502
H	-4.931054	-4.642669	-2.079063
H	-4.019097	-3.489522	-1.083726
H	-5.443413	-2.952159	-1.995167
C	1.430609	-3.837566	-5.909961
H	2.347954	-3.343646	-6.269707
H	1.488052	-4.903603	-6.185906
H	0.573447	-3.401309	-6.447519
C	2.522487	-4.226799	-3.654560
H	3.413291	-3.633549	-3.912051
H	2.391201	-4.176930	-2.564772
H	2.726061	-5.273452	-3.933135
C	-0.276253	-5.556591	2.137070
C	-1.431609	-5.505337	1.337099
C	-2.628852	-5.992454	1.889530
C	-2.729733	-6.213011	3.267196
C	-1.641494	-5.941893	4.114448
C	-0.382626	-5.770314	3.513254
C	-1.859824	-5.434898	5.510327
H	-1.791186	-6.102933	6.377277
C	-3.339294	-3.225275	3.713811
C	-2.172056	-3.214277	4.498228
C	-0.997383	-2.690806	3.921050
C	-0.968616	-2.482803	2.535944
C	-2.055833	-2.781423	1.710343
C	-3.299927	-3.015234	2.327138
C	-2.091288	-4.114946	5.696227
H	-2.196916	-3.719091	6.713067
H	-0.016353	-2.301177	2.051363
H	-4.247404	-3.617341	4.170440
H	-3.713154	-6.412119	3.701622
H	0.682360	-5.238897	1.723737

C	2.709049	3.186060	3.427918
H	3.152717	2.794759	4.351877
C	2.894940	4.477432	3.115926
H	3.474710	5.108270	3.801171
C	2.462804	5.082228	1.825737
C	3.334054	5.014963	0.718118
C	1.197841	5.654922	1.654130
C	2.941300	5.568346	-0.503311
C	0.776358	6.143935	0.409734
H	0.516756	5.663313	2.503879
C	1.675994	6.160362	-0.683386
H	3.623840	5.543833	-1.353476
C	1.332770	6.658601	-2.027591
H	1.779205	6.060928	-2.831536
C	0.592771	7.713406	-2.445034
H	0.458735	7.786758	-3.530757
C	-0.044603	8.805815	-1.695769
C	0.413555	9.250377	-0.438522
C	-1.142470	9.476303	-2.271499
C	-0.224841	10.294555	0.229377
H	1.283091	8.772368	0.014845
C	-1.789393	10.516797	-1.600009
H	-1.502131	9.162212	-3.255451
C	-1.336860	10.927439	-0.341852
H	0.147671	10.619633	1.204386
H	-2.649884	11.008792	-2.060734
H	-1.840236	11.740700	0.186925
H	0.496563	-5.606611	4.141593
H	-3.534791	-6.014698	1.278435
O	-4.363173	-3.223855	1.496926
O	0.131766	-2.623266	4.682084
C	-5.608923	-3.639205	2.033132
H	-5.974946	-2.895310	2.766628
H	-5.492535	-4.600910	2.568185
C	-6.592394	-3.787412	0.885312
H	-6.648790	-2.828157	0.342776
H	-6.189336	-4.523477	0.170182
C	-7.986854	-4.213281	1.345785
H	-7.917628	-5.166528	1.901853
H	-8.382243	-3.472320	2.064908
C	-8.976819	-4.374215	0.189690
H	-8.579368	-5.115173	-0.528589
H	-9.042649	-3.421518	-0.368022
C	-10.378016	-4.799448	0.633349
H	-10.310315	-5.750949	1.193074
H	-10.774568	-4.057011	1.350829
C	-11.366373	-4.962701	-0.523434
H	-10.969447	-5.705507	-1.240761
H	-11.433107	-4.011241	-1.083964
C	-12.769504	-5.387523	-0.081912
H	-12.700107	-6.337575	0.478899
H	-13.163868	-4.643994	0.634741
C	-13.748310	-5.548472	-1.245771
H	-13.395009	-6.311101	-1.960372
H	-14.748183	-5.854145	-0.898037
H	-13.862497	-4.603440	-1.803438
C	1.312644	-2.170675	4.021905
H	1.502287	-2.780632	3.123004
H	1.162585	-1.129651	3.677778
C	2.542012	-2.260145	4.905895
H	2.752025	-3.321376	5.124724
H	2.367512	-1.759497	5.872969
C	3.714740	-1.622382	4.141682
H	3.674716	-0.525144	4.257858
H	3.565026	-1.806342	3.063195
C	5.109536	-2.134865	4.494248
H	5.343948	-1.937379	5.556571
H	5.122233	-3.232150	4.370309

C	6.182210	-1.520895	3.589389
H	5.840304	-1.593765	2.541048
H	6.271426	-0.438656	3.800221
C	7.556974	-2.181734	3.693803
H	7.928759	-2.121307	4.733899
H	7.450835	-3.260644	3.472586
C	8.588381	-1.574140	2.739813
H	8.181858	-1.602574	1.714237
H	8.717114	-0.501710	2.974545
C	9.944920	-2.277255	2.768182
H	10.654150	-1.818311	2.060987
H	10.396970	-2.236330	3.773647
H	9.845402	-3.341548	2.494870
O	-0.490589	6.565394	0.166909
O	4.521796	4.385752	0.919046
H	-1.153797	1.561875	1.383197
H	3.717065	0.988090	2.171869
C	5.229189	3.840369	-0.188602
H	4.519687	3.389183	-0.905366
H	5.781695	4.637783	-0.722155
C	6.181784	2.781551	0.340187
H	5.582805	2.005396	0.846820
H	6.826273	3.235242	1.112964
C	7.032122	2.149102	-0.761179
H	7.658365	2.925680	-1.238472
H	6.371401	1.755502	-1.552880
C	7.924274	1.014422	-0.254631
H	7.288627	0.236720	0.205303
H	8.576078	1.387432	0.557036
C	8.786104	0.381262	-1.349287
H	9.432806	1.155980	-1.801513
H	8.130590	0.027779	-2.166580
C	9.655132	-0.774491	-0.850335
H	10.306634	-0.414236	-0.032859
H	9.011680	-1.546360	-0.390422
C	10.514345	-1.427728	-1.934608
H	11.167617	-0.662756	-2.392760
H	9.857603	-1.788393	-2.747419
C	11.364170	-2.584858	-1.407532
H	12.052355	-2.243711	-0.615630
H	10.730532	-3.375538	-0.971115
H	11.971272	-3.043511	-2.204530
C	-1.375624	6.843428	1.242328
H	-1.597031	5.918266	1.808563
H	-0.900979	7.558510	1.940292
C	-2.645550	7.432959	0.654496
H	-2.367320	8.298202	0.034599
H	-3.100850	6.692885	-0.026735
C	-3.654280	7.860102	1.720329
H	-3.937135	6.991078	2.343227
H	-3.176032	8.582928	2.407055
C	-4.914781	8.492969	1.126409
H	-4.623142	9.358366	0.503063
H	-5.393539	7.773673	0.436256
C	-5.932310	8.942725	2.176544
H	-6.222519	8.075864	2.799264
H	-5.450741	9.660011	2.867194
C	-7.189220	9.581012	1.581272
H	-6.898248	10.447830	0.958501
H	-7.670483	8.864041	0.889726
C	-8.209268	10.032770	2.629854
H	-7.725895	10.748293	3.320051
H	-8.498098	9.165138	3.250986
C	-9.460439	10.669563	2.023496
H	-10.175353	10.984247	2.800708
H	-9.203500	11.559974	1.424960
H	-9.982094	9.963779	1.355011

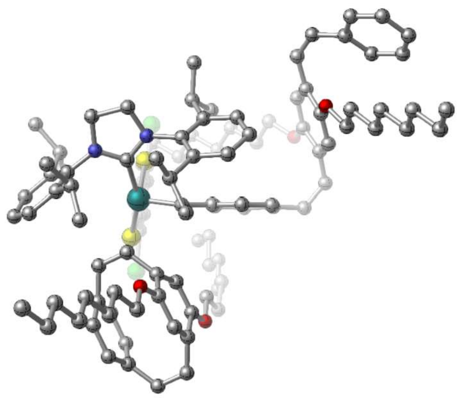

Zero-point correction= 2.148548 (Hartree/Particle)

Thermal correction to Energy= 2.270981

Thermal correction to Enthalpy= 2.271926

Thermal correction to Gibbs Free Energy= 1.972727

Sum of electronic and zero-point Energies= -6260.445669

Sum of electronic and thermal Energies= -6260.323235

Sum of electronic and thermal Enthalpies= -6260.322291

Sum of electronic and thermal Free Energies= -6260.621490

M06L-D3/def2tzvpp-SDD(Ru)-SMD(benzene).

E(scf) = -6267.09251365 a.u.

TS2-α1 (O^nOct)

E(scf) = -6262.56726629 a.u.

ν_{min} = -65.73 cm^{-1}

S	-3.406491	-1.423299	-1.842419	C	-2.495019	1.096997	-5.032072
C	-2.597819	1.136031	-2.665739	C	-3.896077	1.520524	-4.599328
Ru	-2.563599	0.363584	-0.663644	C	-4.654902	2.560703	-2.441304
C	-2.643606	2.419307	0.016137	H	-4.241662	2.442290	-5.084882
C	-0.649018	0.268692	-0.490003	H	-4.646849	0.732809	-4.779268
S	-3.417782	-0.823578	1.257050	H	-1.935153	1.906279	-5.526346
N	-1.859605	0.756950	-3.741355	C	2.170686	-0.525186	-3.769469
N	-3.732379	1.713430	-3.148854	C	-0.193465	-1.014103	-4.097504
C	-0.492195	0.319690	-3.740386	C	0.536471	1.253741	-3.443270

C	1.862668	0.801454	-3.464420
C	1.151748	-1.417574	-4.090109
H	2.672511	1.493786	-3.229717
H	1.401231	-2.450172	-4.343297
C	-6.333333	4.272515	-1.019504
C	-4.392678	3.953116	-2.450200
C	-5.810684	2.032890	-1.826423
C	-6.626108	2.913471	-1.098582
C	-5.239783	4.789893	-1.713354
C	-3.279995	4.564782	-3.299007
C	-6.252564	0.592169	-2.033711
H	-7.512892	2.525102	-0.593845
H	-5.043211	5.863809	-1.684079
H	-2.501758	0.231080	-5.704892
H	-2.666480	3.747792	-3.701930
H	-5.402744	0.040252	-2.452465
C	-1.265200	-2.005710	-4.530216
H	-2.243877	-1.563418	-4.305480
C	0.242666	2.722109	-3.135858
H	-0.767311	2.780568	-2.704931
C	-4.157940	-2.566602	-0.732474
C	-4.816978	-3.718587	-1.203721
C	-4.100327	-2.322575	0.661167
C	-5.389930	-4.640973	-0.330850
C	-4.657219	-3.288025	1.530696
C	-5.297004	-4.427209	1.048121
H	-5.893075	-5.525505	-0.720954
H	-5.719811	-5.148267	1.749065
H	-2.592907	3.057240	-0.863979
C	-1.280754	1.998455	0.446306
H	-0.514082	2.481179	-0.163521
H	-0.088673	0.744033	-1.296799
C	0.292422	-0.469788	0.361262
C	-0.042783	-1.359930	1.402349

C	1.668337	-0.202622	0.150920
C	0.943478	-1.904837	2.219316
H	-1.090383	-1.609406	1.568985
C	2.649631	-0.753362	0.960994
C	2.309554	-1.618670	2.020979
H	3.695929	-0.501797	0.783892
H	3.211026	-0.860008	-3.759954
H	-6.972748	4.937026	-0.433107
Cl	-4.942524	-4.018776	-2.936461
Cl	-4.541413	-3.082325	3.274238
C	-1.188075	-2.264575	-6.046808
H	-0.233655	-2.746293	-6.315901
H	-2.004297	-2.933897	-6.363487
H	-1.261714	-1.333776	-6.631310
C	-1.190750	-3.328196	-3.756435
H	-1.142711	-3.151880	-2.673365
H	-2.088573	-3.933552	-3.951486
H	-0.312682	-3.921555	-4.054487
C	0.253589	3.558345	-4.429583
H	1.246732	3.513295	-4.904884
H	-0.481675	3.197501	-5.161828
H	0.025721	4.612953	-4.212570
C	1.201396	3.349722	-2.110830
H	2.202416	3.523279	-2.535711
H	0.810496	4.326494	-1.787323
H	1.325757	2.721730	-1.216280
C	-3.879207	5.325711	-4.496775
H	-3.079952	5.691900	-5.161677
H	-4.555246	4.690327	-5.090239
H	-4.461373	6.197037	-4.155405
C	-2.337307	5.469628	-2.499882
H	-2.874756	6.322189	-2.056975
H	-1.861394	4.926555	-1.674859
H	-1.550871	5.879268	-3.152957

C	-7.394490	0.539167	-3.065879	C	6.678537	-1.967317	1.395474
H	-7.670732	-0.506774	-3.277834	C	5.612479	-2.989570	-0.949199
H	-8.292487	1.059968	-2.693915	C	7.450917	-1.941065	0.228460
H	-7.102658	1.015535	-4.015848	H	7.081449	-1.555954	2.321170
C	-6.648812	-0.120757	-0.734018	C	6.929928	-2.491704	-0.968062
H	-6.868474	-1.180619	-0.935511	H	5.210692	-3.404437	-1.873878
H	-5.833004	-0.086751	0.000856	C	7.666164	-2.521599	-2.245762
H	-7.546834	0.323648	-0.275398	H	7.036528	-2.289190	-3.113617
C	-4.409560	2.331115	1.886428	C	8.956858	-2.812637	-2.532363
C	-3.500722	3.032654	1.091701	H	9.239780	-2.696451	-3.585277
C	-3.120771	4.325345	1.525702	C	10.052909	-3.288340	-1.675182
C	-3.421785	4.719991	2.830462	C	9.845712	-4.004022	-0.477937
C	-4.071670	3.864463	3.733465	C	11.382500	-3.055731	-2.081259
C	-4.699832	2.718663	3.207276	C	10.922666	-4.431035	0.297932
C	-3.683065	3.995018	5.176764	H	8.828157	-4.230464	-0.156779
H	-4.381617	4.386655	5.925423	C	12.462894	-3.475721	-1.301326
C	-0.634058	3.685782	3.706049	H	11.567707	-2.520820	-3.017071
C	-1.533738	2.986166	4.533122	C	12.237768	-4.160450	-0.102705
C	-1.853787	1.676285	4.147700	H	10.736073	-4.981259	1.223952
C	-1.568646	1.221667	2.863606	H	13.484166	-3.266872	-1.630177
C	-0.970370	2.060049	1.916099	H	13.079882	-4.489583	0.511180
C	-0.351926	3.227419	2.411375	O	8.688828	-1.391310	0.164293
C	-2.428730	3.641335	5.545787	O	3.587494	-3.517159	0.280652
H	-2.106452	3.775673	6.585468	H	1.961450	0.466597	-0.660517
H	-1.963233	0.256709	2.568294	H	0.652172	-2.579118	3.029179
H	-0.279966	4.675579	4.007165	C	2.959592	-4.088187	-0.854756
H	-2.961130	5.617134	3.239702	H	2.902414	-3.349226	-1.677179
H	-4.735011	1.359948	1.529893	H	3.550883	-4.950698	-1.219742
C	3.318890	-2.176959	2.930583	C	1.566727	-4.525726	-0.430912
H	2.965191	-2.346255	3.954223	H	0.965131	-3.628443	-0.209073
C	4.617337	-2.471492	2.686845	H	1.656804	-5.080944	0.518295
H	5.218490	-2.760050	3.557762	C	0.871125	-5.391113	-1.479441
C	5.364725	-2.467303	1.415362	H	1.448301	-6.322103	-1.630945
C	4.836048	-3.002714	0.213148	H	0.879249	-4.869025	-2.451731

C	-0.571889	-5.741912	-1.111932
H	-1.153493	-4.810331	-0.988538
H	-0.588304	-6.237009	-0.123441
C	-1.263003	-6.636326	-2.142906
H	-0.711993	-7.591553	-2.230712
H	-1.196369	-6.158313	-3.136619
C	-2.732072	-6.913761	-1.823897
H	-2.810245	-7.418015	-0.842425
H	-3.258461	-5.952590	-1.709722
C	-3.446312	-7.752022	-2.886804
H	-2.945790	-8.733919	-2.975699
H	-3.325391	-7.261391	-3.870119
C	-4.936024	-7.950741	-2.602763
H	-5.096849	-8.410679	-1.612807
H	-5.464750	-6.983965	-2.613554
H	-5.412107	-8.600481	-3.354963
C	9.424001	-1.140937	1.353588
H	8.937426	-0.346058	1.951038
H	9.451082	-2.056784	1.974167
C	10.829295	-0.728265	0.952481
H	11.240727	-1.507701	0.294114
H	10.769706	0.194678	0.349847
C	11.757062	-0.521815	2.149611
H	11.350244	0.263453	2.813702
H	11.783768	-1.447318	2.754402
C	13.184226	-0.152477	1.738378
H	13.581797	-0.940900	1.072851
H	13.161011	0.770570	1.129879
C	14.136367	0.042779	2.919807
H	13.736854	0.830922	3.585143
H	14.157279	-0.880803	3.528133
C	15.563637	0.406901	2.505139
H	15.962635	-0.381685	1.839657
H	15.542487	1.329955	1.895708

C	16.519790	0.603091	3.684695
H	16.538989	-0.320141	4.292409
H	16.118719	1.390932	4.348367
C	17.942552	0.966524	3.257760
H	18.607125	1.101259	4.126328
H	18.380148	0.179357	2.620568
H	17.956423	1.904521	2.677158
H	0.211196	3.872216	1.731701
H	-2.493049	1.065954	4.790423
O	-2.266467	5.029890	0.720544
O	-5.403256	1.929797	4.068233
C	-1.636111	6.199721	1.221423
H	-2.383934	7.002660	1.368007
H	-1.182853	5.991773	2.205615
C	-0.549076	6.638841	0.253799
H	-1.000173	6.954864	-0.698817
H	0.083269	5.763337	0.025502
C	0.312847	7.770252	0.816120
H	-0.330279	8.630997	1.077372
H	0.779523	7.443438	1.763664
C	1.404732	8.230376	-0.152503
H	0.936846	8.561458	-1.098258
H	2.041095	7.366025	-0.419230
C	2.282773	9.355117	0.399542
H	1.644452	10.217565	0.667800
H	2.749990	9.022263	1.345105
C	3.374350	9.815796	-0.568900
H	2.906879	10.148289	-1.514866
H	4.012466	8.952872	-0.837370
C	4.253746	10.941400	-0.017781
H	3.613808	11.801833	0.250687
H	4.719146	10.606957	0.927449
C	5.340749	11.394370	-0.993372
H	4.901166	11.765718	-1.934642

H	5.956657	12.204003	-0.569978
H	6.015725	10.560985	-1.252176
C	-6.054343	0.780027	3.535349
H	-6.821906	1.098798	2.802484
H	-5.332665	0.142945	2.998490
C	-6.677935	-0.035287	4.652096
H	-5.867640	-0.414503	5.298612
H	-7.314481	0.607509	5.283839
C	-7.479969	-1.205997	4.068843
H	-8.441928	-0.834801	3.670877
H	-6.932511	-1.615218	3.202593
C	-7.731514	-2.349210	5.051737
H	-8.322518	-1.985619	5.912970
H	-6.761491	-2.681473	5.464262
C	-8.431096	-3.548740	4.409770
H	-9.403975	-3.229320	3.991380
H	-7.828385	-3.890043	3.547591
C	-8.648990	-4.722824	5.365690
H	-9.250822	-4.386376	6.230919
H	-7.673618	-5.036937	5.782661
C	-9.330063	-5.929605	4.714644
H	-10.304373	-5.614563	4.297989
H	-8.726947	-6.260670	3.849236
C	-9.536785	-7.101571	5.674997
H	-10.164663	-6.806855	6.532982
H	-10.027706	-7.954409	5.178895
H	-8.574530	-7.458486	6.079775

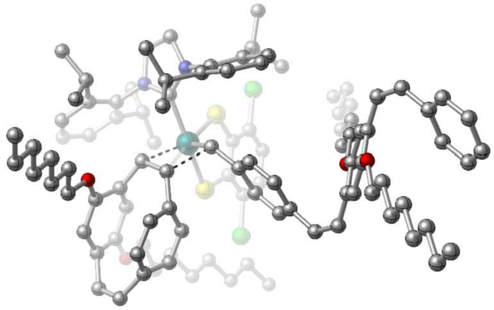

Zero-point correction= 2.150847 (Hartree/Particle)

Thermal correction to Energy= 2.271612

Thermal correction to Enthalpy= 2.272556

Thermal correction to Gibbs Free Energy= 1.976275

Sum of electronic and zero-point Energies= -6260.416419

Sum of electronic and thermal Energies= -6260.295654

Sum of electronic and thermal Enthalpies= -6260.294710

Sum of electronic and thermal Free Energies= -6260.590991

M06L-D3/def2tzvpp-SDD(Ru)-SMD(benzene).

E(scf) = -6267.07633336 a.u.

TS2-β1 (O^{n}Oct)

E(scf) = -6262.57389305 a.u.

ν_{min} = -128.11 cm^{-1}

S	-2.692687	-0.956482	-2.535067
C	-1.894053	1.779097	-2.635438
Ru	-2.451943	0.630195	-0.899462
C	-3.093979	2.434118	0.082973
C	-0.689144	0.633525	-0.131118
S	-3.703993	-0.897156	0.465021
N	-0.823048	1.582889	-3.453280
N	-2.816393	2.472857	-3.353060
C	0.509396	1.199891	-3.073031
C	-1.045095	2.055295	-4.836127
C	-2.440348	2.680285	-4.761727
C	-4.000726	3.132460	-2.872144
H	-2.449695	3.752076	-5.010279
H	-3.167759	2.175566	-5.415851
H	-0.262072	2.769568	-5.128655
C	3.134148	0.512893	-2.387761
C	1.038368	-0.035745	-3.506538
C	1.304067	2.121507	-2.337940
C	2.613216	1.749778	-2.006641
C	2.354740	-0.360622	-3.138219
H	3.238753	2.432289	-1.430297
H	2.772782	-1.319866	-3.449749
C	-6.253698	4.462309	-1.904463
C	-3.884946	4.484187	-2.459926
C	-5.244286	2.466770	-2.874784
C	-6.359056	3.153408	-2.367464
C	-5.028235	5.125715	-1.967911
C	-2.577381	5.265290	-2.570496
C	-5.423422	1.079985	-3.472629
H	-7.328554	2.651504	-2.343680
H	-4.959157	6.161169	-1.626742
H	-1.005779	1.210183	-5.537059
H	-1.798636	4.576058	-2.920168
H	-4.425439	0.672543	-3.674497
C	0.262233	-1.002635	-4.390599
H	-0.778566	-0.658463	-4.437191
C	0.782178	3.497333	-1.926666
H	-0.305013	3.413580	-1.785154
C	-3.610234	-2.317677	-1.889756
C	-3.950354	-3.418373	-2.700841
C	-4.060109	-2.282548	-0.546674
C	-4.746312	-4.457100	-2.226848
C	-4.854627	-3.356418	-0.081891
C	-5.199276	-4.424778	-0.905066
H	-5.008724	-5.286380	-2.882591
H	-5.823159	-5.229638	-0.514466
H	-3.013200	3.228762	-0.652924
C	-1.761195	2.162827	0.711655
H	-1.009644	2.850731	0.326772
H	0.039011	1.294616	-0.599868
C	0.032569	-0.274797	0.771746
C	-0.488615	-1.425440	1.402189
C	1.396101	0.029007	0.995874
C	0.312201	-2.221390	2.215215
H	-1.530390	-1.696898	1.233176
C	2.200651	-0.779915	1.786723
C	1.678657	-1.935686	2.397292
H	3.251076	-0.523531	1.930696
H	4.148911	0.232985	-2.096553
H	-7.132078	4.973975	-1.503478

Cl	-3.386418	-3.507061	-4.367065
Cl	-5.482841	-3.349231	1.559866
C	0.833887	-1.011576	-5.820218
H	1.868476	-1.392258	-5.829635
H	0.229168	-1.662752	-6.471965
H	0.850032	-0.002811	-6.262508
C	0.222547	-2.420515	-3.807440
H	-0.157229	-2.399144	-2.777474
H	-0.456214	-3.056462	-4.394790
H	1.216286	-2.896146	-3.806961
C	1.026058	4.531077	-3.041940
H	2.105355	4.637611	-3.236422
H	0.543549	4.243591	-3.986039
H	0.632777	5.517407	-2.750318
C	1.361088	4.013988	-0.602540
H	2.425127	4.282384	-0.694553
H	0.820966	4.917955	-0.292850
H	1.264735	3.280021	0.210928
C	-2.700467	6.386382	-3.618478
H	-1.728669	6.884339	-3.767817
H	-3.039298	5.996448	-4.591093
H	-3.425817	7.151324	-3.297550
C	-2.091581	5.832493	-1.227191
H	-2.826924	6.523046	-0.784867
H	-1.891559	5.041193	-0.491521
H	-1.157361	6.397336	-1.371346
C	-6.172538	1.166306	-4.814860
H	-6.244593	0.168527	-5.277658
H	-7.196946	1.550250	-4.677458
H	-5.658569	1.835796	-5.523592
C	-6.117182	0.102983	-2.512091
H	-6.111751	-0.913317	-2.935468
H	-5.598657	0.061720	-1.543690
H	-7.166718	0.384689	-2.329533

C	-5.058758	1.770231	1.654419
C	-4.236723	2.716997	1.026914
C	-4.267825	4.025537	1.558440
C	-4.786274	4.265002	2.832512
C	-5.307720	3.209244	3.601827
C	-5.583711	2.009208	2.927680
C	-5.193385	3.211620	5.098246
H	-6.041485	3.488779	5.736211
C	-2.119399	3.449814	4.166439
C	-2.888297	2.437270	4.759918
C	-2.818917	1.148188	4.197079
C	-2.266193	0.996000	2.917307
C	-1.733626	2.073138	2.208303
C	-1.521756	3.282862	2.911262
C	-4.029707	2.812772	5.659557
H	-3.937687	2.758343	6.750748
H	-2.417184	0.061322	2.389893
H	-2.162310	4.440850	4.615589
H	-4.624215	5.241858	3.296420
H	-5.160872	0.777727	1.221241
C	2.555063	-2.836418	3.169598
H	2.172366	-3.194961	4.132053
C	3.772451	-3.251617	2.764898
H	4.369740	-3.858409	3.456080
C	4.386389	-2.961641	1.447815
C	3.702711	-3.235953	0.237225
C	5.664116	-2.386883	1.379877
C	4.299943	-2.890363	-0.978891
C	6.256756	-2.032210	0.160610
H	6.177277	-2.169702	2.316885
C	5.582609	-2.313413	-1.052382
H	3.779579	-3.090335	-1.914772
C	6.117270	-1.983083	-2.386895
H	5.353923	-1.609296	-3.080432

C	7.360817	-2.089552	-2.911726	H	2.721285	10.845076	0.974919
H	7.471480	-1.704656	-3.932385	C	1.797707	12.686010	1.639925
C	8.595985	-2.665221	-2.359631	H	0.777931	13.084782	1.791579
C	8.606008	-3.664728	-1.364986	H	2.270472	12.682848	2.639117
C	9.836109	-2.233844	-2.872095	C	2.577604	13.611940	0.705619
C	9.806612	-4.179765	-0.877826	H	2.106973	13.660003	-0.291038
H	7.660201	-4.043446	-0.975187	H	2.629817	14.639338	1.100369
C	11.040502	-2.742438	-2.379367	H	3.611825	13.255066	0.563805
H	9.852263	-1.474374	-3.658954	C	-3.329710	-1.182427	4.338196
C	11.031728	-3.714540	-1.373775	H	-3.777414	-1.268131	3.333083
H	9.788272	-4.952111	-0.104377	H	-2.253886	-1.424519	4.238620
H	11.989293	-2.378070	-2.781601	C	-4.005931	-2.151030	5.291283
H	11.971542	-4.114198	-0.984711	H	-5.091018	-1.947300	5.296401
H	-6.052738	1.182169	3.467093	H	-3.644703	-1.963355	6.317215
H	-3.720944	4.824484	1.052898	C	-3.742024	-3.603451	4.884938
O	-0.913966	4.303091	2.229034	H	-2.666030	-3.827369	5.007777
O	-3.471489	0.144225	4.840186	H	-3.952171	-3.721749	3.808454
C	-0.841544	5.587027	2.830368	C	-4.566973	-4.628728	5.663836
H	-0.357425	5.517068	3.822861	H	-4.340598	-4.547503	6.743373
H	-1.861716	5.986934	2.988253	H	-5.640296	-4.382874	5.561302
C	-0.046072	6.508651	1.923602	C	-4.335313	-6.067219	5.195722
H	0.965391	6.089592	1.790542	H	-4.566933	-6.134435	4.116249
H	-0.517836	6.516346	0.928735	H	-3.260988	-6.313888	5.288195
C	0.045179	7.937479	2.461118	C	-5.160355	-7.107209	5.956144
H	-0.973350	8.341048	2.610756	H	-4.927862	-7.041188	7.035780
H	0.517288	7.930082	3.460782	H	-6.234463	-6.858275	5.865444
C	0.827598	8.873896	1.537333	C	-4.933808	-8.543904	5.477754
H	0.355149	8.874555	0.537266	H	-5.165310	-8.605972	4.398618
H	1.846053	8.470129	1.387433	H	-3.860024	-8.790525	5.568760
C	0.922635	10.311060	2.053349	C	-5.764298	-9.576141	6.241525
H	-0.096791	10.712419	2.204884	H	-5.580583	-10.599258	5.875635
H	1.395894	10.309257	3.052933	H	-5.527948	-9.558651	7.319051
C	1.701736	11.246797	1.126517	H	-6.843801	-9.372926	6.138882
H	1.228482	11.247381	0.126503	O	7.450537	-1.395619	0.068465

O	2.492547	-3.838397	0.338896
H	1.829386	0.912302	0.523757
H	-0.115332	-3.113298	2.680427
C	1.724701	-4.101080	-0.826677
H	1.487488	-3.153579	-1.340584
H	2.307153	-4.726720	-1.530603
C	0.458214	-4.831997	-0.407896
H	-0.174980	-4.149511	0.181834
H	0.752588	-5.653591	0.268016
C	-0.325266	-5.394574	-1.595303
H	0.343655	-6.034643	-2.200569
H	-0.643467	-4.574366	-2.260196
C	-1.555163	-6.206475	-1.184795
H	-2.270830	-5.548351	-0.659063
H	-1.256378	-6.981527	-0.454462
C	-2.257717	-6.870421	-2.370508
H	-1.534263	-7.503811	-2.917499
H	-2.572483	-6.089886	-3.085320
C	-3.466647	-7.718450	-1.972459
H	-3.136469	-8.543525	-1.313855
H	-4.153721	-7.107037	-1.360994
C	-4.231782	-8.297244	-3.165081
H	-3.548125	-8.923856	-3.766552
H	-4.540347	-7.471515	-3.832859
C	-5.459164	-9.113603	-2.758912
H	-5.176339	-9.964630	-2.116434
H	-6.174664	-8.496266	-2.189399
H	-5.990298	-9.518049	-3.635640
C	8.341090	-1.379514	1.174814
H	7.915584	-0.787163	2.007614
H	8.495113	-2.411576	1.543295
C	9.654954	-0.780027	0.705082
H	9.992325	-1.343243	-0.177554
H	9.472792	0.255227	0.367972
C	10.742035	-0.805663	1.779348
H	10.410278	-0.238156	2.668756
H	10.891521	-1.846585	2.121610
C	12.078352	-0.245510	1.286509
H	12.399221	-0.814474	0.394379
H	11.933125	0.795398	0.942652
C	13.188496	-0.282249	2.338504
H	12.865970	0.286218	3.230843
H	13.330896	-1.323952	2.682105
C	14.525182	0.272630	1.841450
H	14.846417	-0.295421	0.948110
H	14.383016	1.314615	1.498052
C	15.639368	0.234879	2.890849
H	15.779216	-0.807025	3.232765
H	15.315905	0.802488	3.782589
C	16.969981	0.791462	2.382605
H	17.751684	0.751445	3.158206
H	17.333441	0.220366	1.511467
H	16.866447	1.843166	2.065867

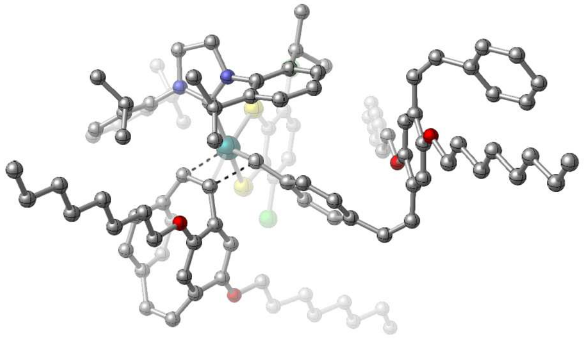

Zero-point correction= 2.150887 (Hartree/Particle)

Thermal correction to Energy= 2.271795

Thermal correction to Enthalpy= 2.272739

Thermal correction to Gibbs Free Energy= 1.976145

Sum of electronic and zero-point Energies= -6260.423006

Sum of electronic and thermal Energies= -6260.302099

Sum of electronic and thermal Enthalpies= -6260.301154

Sum of electronic and thermal Free Energies= -6260.597748

M06L-D3/def2tzvpp-SDD(Ru)-SMD(benzene).

E(scf) = -6267.08202412 a.u.

Int2-α1 (O^nOct)

E(scf) = -6262.66149486 a.u.

ν_{min} = 8.89 cm^{-1}

S	-2.983921	-0.129458	1.195156	C	-0.680983	-0.076075	5.305802
C	-1.192173	-1.198290	3.302645	C	-1.954687	-0.914367	5.502359
Ru	-1.139505	-1.462851	1.250246	C	-2.914454	-2.803950	4.077567
C	-1.685189	-3.203554	1.027418	H	-1.907150	-1.590134	6.367007
C	3.563257	-2.704208	-3.385437	H	-2.844144	-0.277600	5.616585
S	-0.748415	-0.894345	-1.007127	H	0.172953	-0.444261	5.899484
N	-0.410373	-0.256454	3.870342	C	3.092786	1.335987	2.112531
N	-2.040575	-1.675354	4.237191	C	0.710660	1.532374	2.593861
C	0.772151	0.288370	3.257173	C	1.981172	-0.440558	3.353870

C	3.132604	0.106028	2.770553	C	2.921662	-0.604024	-2.121611
C	1.889769	2.034292	2.023692	C	1.950789	0.540862	-4.457138
H	4.076292	-0.441819	2.827713	H	2.537676	-1.288831	-5.431581
H	1.864133	2.987770	1.497378	C	2.346886	0.667984	-2.085273
C	-4.590250	-5.017430	3.816075	C	1.858357	1.279351	-3.258031
C	-2.345472	-4.105394	4.071799	H	2.237540	1.169160	-1.123077
C	-4.305426	-2.602665	3.944542	H	3.996975	1.759276	1.669989
C	-5.127630	-3.734417	3.823909	H	-5.245900	-5.885514	3.711071
C	-3.211206	-5.197251	3.932747	Cl	-5.136304	2.092788	0.481610
C	-0.840614	-4.328360	4.218476	Cl	-1.332946	0.009272	-4.058914
C	-4.942285	-1.217950	3.944745	C	-0.541029	3.592668	3.335175
H	-6.206817	-3.604195	3.723991	H	0.284979	4.257610	3.033695
H	-2.807866	-6.209537	3.911648	H	-1.482738	4.157256	3.238173
H	-0.828179	0.987605	5.541527	H	-0.397938	3.346733	4.399918
H	-0.339789	-3.534713	3.646844	C	-0.865972	2.664267	0.991067
H	-4.143783	-0.481949	3.771456	H	-0.805698	1.767601	0.362631
C	-0.578310	2.326792	2.461880	H	-1.876242	3.085401	0.882267
H	-1.403447	1.693184	2.815342	H	-0.145341	3.389099	0.591544
C	2.055443	-1.804476	4.027163	C	3.055882	-1.828641	5.193355
H	1.068952	-2.037686	4.447052	H	4.086724	-1.651671	4.847170
C	-3.045622	0.561808	-0.431684	H	2.816044	-1.056605	5.941620
C	-4.020939	1.521387	-0.759655	H	3.036262	-2.809832	5.694903
C	-2.127362	0.137556	-1.427823	C	2.371691	-2.894534	2.992970
C	-4.156957	2.025575	-2.050839	H	3.370687	-2.746722	2.554917
C	-2.330390	0.605258	-2.744955	H	2.348828	-3.895639	3.450984
C	-3.318012	1.541289	-3.056457	H	1.645639	-2.880906	2.165870
H	-4.927545	2.763826	-2.273816	C	-0.385628	-4.193713	5.682781
H	-3.427393	1.888747	-4.084360	H	0.705921	-4.323417	5.760588
H	-0.924169	-3.884071	1.454872	H	-0.633611	-3.211374	6.106264
C	2.943683	-3.832984	-3.795605	H	-0.865994	-4.961432	6.310702
H	3.572440	-4.726949	-3.881659	C	-0.354373	-5.664144	3.638627
H	4.618388	-2.793638	-3.099998	H	-0.681634	-6.518498	4.252771
C	2.991632	-1.338884	-3.320083	H	-0.715602	-5.822097	2.612084
C	2.498959	-0.735100	-4.490738	H	0.746494	-5.683035	3.622467

C	-5.594701	-0.916426	5.307555	C	3.278610	3.608645	-1.172490
H	-5.999741	0.108460	5.326080	C	2.240406	5.524862	0.541963
H	-6.427335	-1.612838	5.500256	C	4.010717	3.985921	-0.045064
H	-4.882103	-1.019687	6.140493	H	3.685319	2.865617	-1.852171
C	-5.966674	-1.038438	2.811395	C	3.513716	4.988934	0.825765
H	-6.262699	0.019134	2.733614	H	1.836470	6.288897	1.208709
H	-5.541674	-1.333708	1.842301	C	4.201393	5.434329	2.048718
H	-6.880156	-1.627964	2.990617	H	3.511328	5.642968	2.875463
C	-3.561425	-3.124934	-0.623280	C	5.510705	5.644130	2.327694
C	-2.532509	-3.833211	0.041154	H	5.727623	5.911181	3.368796
C	-2.111294	-5.074681	-0.507166	C	6.702454	5.603531	1.467990
C	-2.555379	-5.444219	-1.776718	C	6.661675	5.841671	0.078780
C	-3.444602	-4.648624	-2.508254	C	7.958079	5.351093	2.056310
C	-4.029237	-3.522271	-1.872829	C	7.821539	5.786930	-0.692827
C	-3.662733	-4.923215	-3.942921	H	5.708137	6.074385	-0.397542
H	-4.692776	-5.054674	-4.291361	C	9.120475	5.287106	1.283739
C	-0.198729	-5.459765	-5.144396	H	8.016887	5.184610	3.135700
C	-1.229610	-4.680918	-4.584839	C	9.056690	5.498205	-0.097462
C	-0.852863	-3.573920	-3.795282	H	7.763396	5.971150	-1.768798
C	0.482528	-3.272239	-3.558147	H	10.079503	5.071447	1.761857
C	1.518516	-4.067675	-4.091963	H	9.963309	5.447961	-0.705649
C	1.143047	-5.172664	-4.884592	O	5.179086	3.387532	0.303714
C	-2.652969	-5.002595	-4.837806	O	0.273838	5.631627	-0.874737
H	-2.899119	-5.307062	-5.862285	H	3.274609	-1.054676	-1.190077
H	0.710722	-2.409933	-2.934338	H	1.556466	0.975920	-5.379123
H	-0.453912	-6.318646	-5.771820	C	-0.622342	6.099902	0.129593
H	-2.155799	-6.345460	-2.243198	H	-0.470488	5.534687	1.064421
H	-3.891622	-2.193804	-0.171415	H	-0.430193	7.166468	0.350101
C	1.155913	2.571711	-3.325756	C	-2.030016	5.874794	-0.405440
H	0.446983	2.603328	-4.160920	H	-2.056351	4.853188	-0.819983
C	1.178487	3.705722	-2.582160	H	-2.209490	6.556781	-1.254523
H	0.441506	4.456982	-2.875527	C	-3.139005	6.014360	0.636665
C	2.001320	4.124589	-1.443404	H	-3.124303	7.022321	1.090453
C	1.494199	5.120308	-0.566433	H	-2.946341	5.304846	1.462377

C	-4.520982	5.725501	0.043407
H	-4.468519	4.780977	-0.526293
H	-4.777894	6.510292	-0.691933
C	-5.637795	5.599990	1.079550
H	-5.708407	6.528819	1.675953
H	-5.369290	4.798365	1.791515
C	-6.998471	5.278286	0.457906
H	-7.313645	6.114865	-0.193313
H	-6.887661	4.403090	-0.206201
C	-8.097627	4.984537	1.481277
H	-8.217983	5.855606	2.151423
H	-7.768253	4.150506	2.128046
C	-9.441822	4.633209	0.841850
H	-9.813690	5.459710	0.213710
H	-9.351110	3.747878	0.190764
H	-10.210077	4.416091	1.601481
C	5.873878	2.583307	-0.641760
H	5.259383	1.710961	-0.933812
H	6.069045	3.176949	-1.554472
C	7.177577	2.132024	-0.008621
H	7.689888	3.015715	0.401081
H	6.951271	1.470721	0.846275
C	8.092276	1.417071	-1.003252
H	7.547507	0.582604	-1.483336
H	8.355524	2.114466	-1.819646
C	9.373369	0.880475	-0.362498
H	9.890131	1.704015	0.163847
H	9.105266	0.144947	0.417700
C	10.334043	0.231078	-1.360458
H	9.791841	-0.535869	-1.942241
H	10.664899	0.987873	-2.095802
C	11.556706	-0.412389	-0.703965
H	12.105395	0.350765	-0.120552
H	11.217137	-1.165653	0.031550
C	12.513224	-1.076748	-1.697395
H	12.849852	-0.325521	-2.435127
H	11.959112	-1.838507	-2.276170
C	13.728010	-1.722664	-1.029943
H	14.396860	-2.193830	-1.768106
H	14.318620	-0.976846	-0.471317
H	13.419838	-2.501882	-0.312266
H	1.920328	-5.815629	-5.308012
H	-1.613571	-2.924966	-3.361310
O	-1.247777	-5.867272	0.196984
O	-4.935832	-2.822447	-2.601467
C	0.022360	-6.197200	-0.383376
H	0.326639	-7.144176	0.089149
H	-0.077981	-6.379892	-1.467138
C	1.052001	-5.109261	-0.116977
H	1.085839	-4.926233	0.969739
H	0.713982	-4.170038	-0.588938
C	2.452579	-5.465935	-0.615841
H	2.762338	-6.439213	-0.191416
H	2.432680	-5.597761	-1.711299
C	3.480240	-4.397527	-0.244272
H	3.480735	-4.266957	0.850934
H	3.152957	-3.430807	-0.665589
C	4.906402	-4.689231	-0.707243
H	5.253898	-5.640007	-0.261955
H	4.913588	-4.847690	-1.799987
C	5.885349	-3.569404	-0.348039
H	5.882261	-3.420280	0.748350
H	5.519748	-2.617334	-0.777099
C	7.319742	-3.813269	-0.821919
H	7.685190	-4.768359	-0.402501
H	7.318740	-3.947183	-1.919257
C	8.274925	-2.681464	-0.443999
H	8.332001	-2.554468	0.650291

H	9.297223	-2.862287	-0.812350	C	-8.243431	2.496652	-2.687990
H	7.935046	-1.721951	-0.865453	H	-9.160701	2.155857	-2.172679
C	-5.559553	-1.691400	-2.000936	H	-7.578454	2.874171	-1.889488
H	-6.195312	-2.024821	-1.157746	C	-8.588206	3.644043	-3.639273
H	-4.796979	-1.011750	-1.589895	H	-9.265053	3.273727	-4.432149
C	-6.369935	-0.943440	-3.043054	H	-7.668525	3.971337	-4.160229
H	-5.669549	-0.546998	-3.797834	C	-9.227656	4.849356	-2.945480
H	-7.043515	-1.642013	-3.567627	H	-10.143831	4.522827	-2.420987
C	-7.159020	0.203197	-2.397926	H	-8.547813	5.210987	-2.154698
H	-8.054738	-0.199862	-1.891743	C	-9.561059	5.994553	-3.901810
H	-6.549971	0.657574	-1.599046	H	-10.268114	5.669131	-4.683754
C	-7.568004	1.307091	-3.373563	H	-10.016938	6.847092	-3.372791
H	-8.233478	0.895567	-4.154833	H	-8.654882	6.363569	-4.411697
H	-6.665503	1.663474	-3.903760				

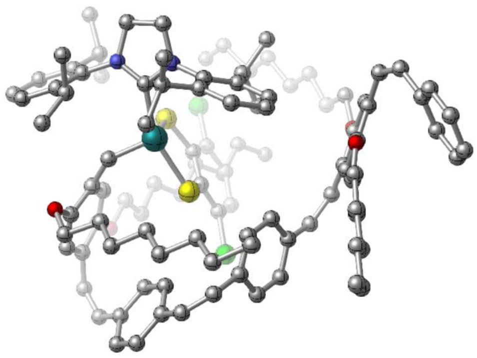

Zero-point correction= 2.152669 (Hartree/Particle)

Thermal correction to Energy= 2.274043

Thermal correction to Enthalpy= 2.274987

Thermal correction to Gibbs Free Energy= 1.982943

Sum of electronic and zero-point Energies= -6260.508826

Sum of electronic and thermal Energies= -6260.387452

Sum of electronic and thermal Enthalpies= -6260.386508

Sum of electronic and thermal Free Energies= -6260.678552

M06L-D3/def2tzvpp-SDD(Ru)-SMD(benzene).

E(scf) = -6267.16116298 a.u.

Int2-β1 (O^nOct)

E(scf) = -6262.62835673 a.u.

ν_{min} = 6.41 cm^{-1}

S	-1.415806	0.389482	2.872093
C	-1.891368	-2.193255	1.634195
Ru	-1.773301	-0.312354	0.713799
C	-3.627103	-0.062680	0.598902
C	-1.196056	-1.040953	-1.787583
S	-0.732555	1.748366	0.067073
N	-0.763071	-2.920693	1.875257
N	-2.851531	-2.669629	2.479785
C	0.459497	-2.966857	1.118746
C	-0.866379	-3.739686	3.097165
C	-2.373542	-3.761566	3.346571
C	-4.269565	-2.486107	2.345436
H	-2.840021	-4.716331	3.052166
H	-2.637946	-3.562937	4.394595
H	-0.429413	-4.735638	2.943463
C	2.894098	-3.339893	-0.207024
C	1.627611	-2.346992	1.619908
C	0.498772	-3.760926	-0.060242
C	1.729342	-3.919749	-0.709635
C	2.839579	-2.561287	0.942416
H	1.786317	-4.513688	-1.622045
H	3.756084	-2.102791	1.313981
C	-7.020074	-2.131912	2.019669
C	-4.964968	-3.279903	1.399986
C	-4.953582	-1.585210	3.192164
C	-6.331875	-1.410747	2.993444
C	-6.343113	-3.076630	1.248143
C	-4.280748	-4.384445	0.603445
C	-4.262189	-0.870580	4.342844
H	-6.875209	-0.701010	3.620431
H	-6.897837	-3.678811	0.526151
H	-0.319300	-3.250413	3.919265
H	-3.207930	-4.337970	0.817459
H	-3.180833	-0.999564	4.209772
C	1.628104	-1.438952	2.840389
H	0.595496	-1.344857	3.197912
C	-0.742724	-4.464982	-0.604572
H	-1.607821	-3.822200	-0.385777
C	-0.763061	2.026759	2.815740
C	-0.533107	2.740458	4.008355
C	-0.454993	2.633372	1.569734
C	-0.014043	4.033986	4.003061
C	0.059216	3.947770	1.593018
C	0.280127	4.643637	2.782150
H	0.150615	4.559253	4.944591
H	0.671148	5.660784	2.750573
H	-4.235724	-0.924834	0.283540
C	-2.512001	-0.712440	-2.030256
H	-3.229114	-1.485567	-1.755292
H	-1.051811	-2.012006	-1.314549
C	0.058153	-0.443192	-2.291497
C	0.191327	0.167871	-3.551388
C	1.212503	-0.538401	-1.494958
C	1.412892	0.707658	-3.957121
H	-0.668623	0.223462	-4.221081
C	2.423656	0.003560	-1.890516
C	2.559935	0.658008	-3.134681
H	3.264004	-0.081733	-1.209946

H	3.848094	-3.479667	-0.715638
H	-8.092638	-1.976492	1.877248
Cl	-0.894013	1.996971	5.563302
Cl	0.448775	4.773229	0.089350
C	2.483422	-2.017378	3.979782
H	3.543742	-2.093576	3.688689
H	2.424776	-1.368690	4.868780
H	2.144883	-3.025471	4.269250
C	2.081643	-0.017424	2.462567
H	1.449312	0.398931	1.665261
H	2.000670	0.652359	3.332974
H	3.124993	-0.001776	2.111429
C	-0.974848	-5.810556	0.106587
H	-0.117197	-6.483206	-0.054634
H	-1.106885	-5.688720	1.189902
H	-1.876249	-6.306775	-0.286101
C	-0.714700	-4.677771	-2.125814
H	0.014850	-5.449459	-2.417731
H	-1.701858	-5.018385	-2.473512
H	-0.464450	-3.753314	-2.667100
C	-4.779376	-5.769232	1.055297
H	-4.230133	-6.567903	0.531348
H	-4.645693	-5.915633	2.138555
H	-5.851059	-5.896053	0.832091
C	-4.436658	-4.223294	-0.914116
H	-5.476825	-4.381395	-1.233491
H	-4.133948	-3.221483	-1.249805
H	-3.816078	-4.963826	-1.441355
C	-4.652111	-1.522480	5.682893
H	-4.104003	-1.048552	6.513397
H	-5.731007	-1.408950	5.880867
H	-4.424908	-2.600705	5.694245
C	-4.534849	0.639962	4.367783
H	-3.890630	1.124312	5.117465
H	-4.308017	1.095174	3.394664
H	-5.583815	0.862782	4.624010
C	-3.888938	2.448168	0.778082
C	-4.428751	1.147780	0.623064
C	-5.767991	1.059915	0.163472
C	-6.407574	2.164296	-0.390739
C	-5.710239	3.378868	-0.535658
C	-4.509730	3.539575	0.182167
C	-6.016320	4.324565	-1.654855
H	-6.621301	5.223693	-1.489017
C	-5.249279	1.610992	-3.113972
C	-4.626393	2.862219	-3.065108
C	-3.218008	2.900716	-2.968849
C	-2.510848	1.716487	-2.754635
C	-3.153199	0.470499	-2.613126
C	-4.548315	0.424759	-2.869058
C	-5.455494	4.090312	-2.859233
H	-5.580835	4.813055	-3.673806
H	-1.446322	1.773885	-2.565035
H	-6.331049	1.592391	-3.219180
H	-7.383906	2.043996	-0.866284
H	-2.920824	2.575862	1.250140
C	3.798935	1.297110	-3.608531
H	3.700096	1.786785	-4.584123
C	5.005703	1.400323	-3.011981
H	5.772769	1.989968	-3.529098
C	5.386976	0.818378	-1.698321
C	5.293677	1.597721	-0.525447
C	5.738001	-0.532122	-1.586082
C	5.530574	1.001656	0.717223
C	5.952044	-1.135252	-0.339766
H	5.762240	-1.131531	-2.495480
C	5.884149	-0.357040	0.840253
H	5.444835	1.589479	1.630888

C	6.064115	-0.900914	2.199271
H	5.381135	-0.456219	2.932831
C	6.917175	-1.824478	2.702630
H	6.760798	-2.074644	3.758628
C	8.047112	-2.535179	2.087203
C	8.776137	-2.026012	0.992967
C	8.456981	-3.766832	2.636300
C	9.843246	-2.739027	0.448116
H	8.502471	-1.058433	0.569926
C	9.520559	-4.486970	2.086593
H	7.918952	-4.170904	3.498503
C	10.215667	-3.978848	0.984289
H	10.391142	-2.324850	-0.402373
H	9.807398	-5.448534	2.520045
H	11.047535	-4.539432	0.550584
H	-3.986439	4.498355	0.147778
H	-6.248562	0.078579	0.129387
O	-5.168123	-0.783623	-2.757446
O	-2.636290	4.127997	-2.985493
C	-6.582884	-0.861413	-2.880905
H	-6.885148	-0.611866	-3.915762
H	-7.065568	-0.130059	-2.209063
C	-7.014609	-2.263787	-2.501787
H	-6.501329	-2.989289	-3.155435
H	-6.659039	-2.456014	-1.478610
C	-8.528514	-2.464254	-2.569004
H	-9.027289	-1.715214	-1.926283
H	-8.887749	-2.269416	-3.596196
C	-8.963566	-3.866447	-2.136111
H	-8.600966	-4.058235	-1.109120
H	-8.463047	-4.617353	-2.774957
C	-10.476694	-4.086005	-2.182846
H	-10.974356	-3.329774	-1.547468
H	-10.840847	-3.901180	-3.210586

C	-10.907835	-5.484518	-1.735929
H	-10.540692	-5.668503	-0.708586
H	-10.410031	-6.240776	-2.371547
C	-12.421955	-5.707491	-1.777720
H	-12.916793	-4.949772	-1.142943
H	-12.786530	-5.523156	-2.804842
C	-12.840704	-7.107588	-1.327359
H	-12.517981	-7.305430	-0.291108
H	-13.933994	-7.238575	-1.367620
H	-12.386950	-7.883684	-1.966766
C	-1.213821	4.208337	-3.057631
H	-0.757473	3.688146	-2.199974
H	-0.863043	3.708727	-3.981756
C	-0.816352	5.672065	-3.040402
H	-1.138220	6.102480	-2.076811
H	-1.368739	6.209919	-3.830095
C	0.691683	5.865831	-3.218288
H	0.989525	5.553211	-4.235868
H	1.233967	5.192304	-2.530879
C	1.148610	7.303115	-2.964974
H	0.627313	7.987136	-3.659978
H	0.826822	7.605056	-1.951039
C	2.660669	7.499336	-3.085055
H	3.169305	6.785112	-2.413315
H	2.990322	7.236860	-4.107405
C	3.116675	8.918884	-2.744276
H	2.654358	9.635500	-3.448718
H	2.729939	9.189686	-1.745931
C	4.635990	9.101893	-2.755441
H	5.092292	8.360530	-2.074012
H	5.026753	8.863573	-3.761509
C	5.074993	10.508586	-2.347026
H	6.171580	10.616128	-2.361622
H	4.654164	11.270425	-3.024922

H	4.728117	10.749062	-1.327468
O	6.159359	-2.468850	-0.187551
O	4.928379	2.894632	-0.693706
H	1.148846	-1.018525	-0.522606
H	1.481447	1.185942	-4.938435
C	4.570608	3.678008	0.436680
H	3.768462	3.171176	1.006868
H	5.438641	3.796482	1.113602
C	4.094119	5.030250	-0.060432
H	3.245449	4.866389	-0.743896
H	4.898449	5.495122	-0.656611
C	3.671099	5.957327	1.079042
H	4.525943	6.127696	1.759690
H	2.893021	5.457164	1.681448
C	3.136575	7.300414	0.583347
H	2.295663	7.115360	-0.105852
H	3.915273	7.804769	-0.015983
C	2.668171	8.236295	1.697411
H	3.496892	8.412056	2.408449
H	1.872133	7.737215	2.281217
C	2.147372	9.579077	1.180948
H	2.946992	10.082765	0.606012
H	1.330175	9.397709	0.457993
C	1.649052	10.518984	2.281234
H	2.466896	10.697280	3.003295
H	0.848353	10.014675	2.852629
C	1.134940	11.855976	1.745769
H	1.926120	12.395315	1.197741
H	0.294656	11.707163	1.046595
H	0.782630	12.512907	2.557301
C	6.578281	-3.260844	-1.290838
H	5.792794	-3.289956	-2.070077
H	7.482211	-2.813852	-1.745045
C	6.867366	-4.657994	-0.771796
H	7.585556	-4.575160	0.057225
H	5.939347	-5.076020	-0.343911
C	7.422330	-5.590991	-1.847604
H	6.702561	-5.675952	-2.683031
H	8.337777	-5.145821	-2.279523
C	7.749206	-6.987255	-1.313152
H	8.465836	-6.893257	-0.476408
H	6.836131	-7.434114	-0.877860
C	8.322926	-7.933069	-2.369909
H	7.605711	-8.024259	-3.207025
H	9.235314	-7.483658	-2.804676
C	8.653250	-9.327479	-1.833195
H	9.369954	-9.235685	-0.995454
H	7.740770	-9.776975	-1.398189
C	9.229060	-10.275848	-2.888258
H	10.140376	-9.824500	-3.321624
H	8.511639	-10.365162	-3.724539
C	9.555055	-11.665977	-2.340665
H	9.967308	-12.325428	-3.121302
H	10.296078	-11.608799	-1.525354
H	8.654024	-12.154036	-1.931915

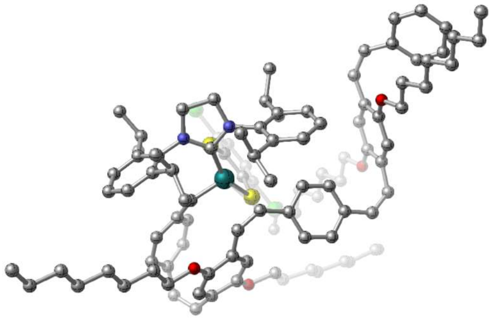

Zero-point correction= 2.152267 (Hartree/Particle)

Thermal correction to Energy= 2.273656

Thermal correction to Enthalpy= 2.274600

Thermal correction to Gibbs Free Energy= 1.981422

Sum of electronic and zero-point Energies= -6260.476090

Sum of electronic and thermal Energies= -6260.354701

Sum of electronic and thermal Enthalpies= -6260.353757

Sum of electronic and thermal Free Energies= -6260.646935

M06L-D3/def2tzvpp-SDD(Ru)-SMD(benzene).

E(scf) = -6267.13031927 a.u.

Ru-1a-ent-M (^{n}Oct)

E(scf) = -4716.81528952 a.u.

ν_{min} = 7.13 cm^{-1}

S	-2.119714	-2.255276	-1.171999	C	1.490669	-3.647029	-2.771832
C	0.710221	-2.504425	-0.858937	C	1.804754	-1.159543	-2.665311
Ru	-0.545500	-1.022038	0.006878	H	2.472787	-3.675273	-3.262299
C	1.222734	0.769982	0.077716	H	0.714233	-3.781253	-3.543738
C	-0.667076	-1.837010	1.693822	H	2.328361	-5.027238	-1.274732
S	-2.243072	0.622363	0.184490	C	0.101072	-5.866017	3.093595
N	0.721715	-3.834190	-0.567509	C	-0.647136	-5.326359	0.838041
N	1.278192	-2.362047	-2.086002	C	1.433972	-4.347182	1.734927
C	0.474096	-4.474768	0.695915	C	1.218040	-5.048806	2.927990
C	1.356266	-4.646573	-1.626730	C	-0.814048	-6.004386	2.054966

H	1.940335	-4.960605	3.741437
H	-1.680735	-6.654797	2.187485
C	2.898255	1.172051	-3.732603
C	3.172996	-0.868963	-2.435474
C	1.000133	-0.337628	-3.484014
C	1.567881	0.843645	-3.986645
C	3.698184	0.308987	-2.983304
C	4.088288	-1.801362	-1.647212
C	-0.409581	-0.735391	-3.890256
H	0.958696	1.508002	-4.602896
H	4.747380	0.559980	-2.813908
H	0.727770	-5.507527	-1.884333
H	3.481184	-2.630208	-1.260446
H	-0.709258	-1.585372	-3.265576
C	-1.647144	-5.579965	-0.282772
H	-1.443722	-4.859552	-1.085661
C	2.718439	-3.539646	1.576615
H	2.571269	-2.831092	0.751077
C	-3.599292	-1.302743	-1.262386
C	-4.738181	-1.789027	-1.936960
C	-3.659009	-0.030649	-0.640917
C	-5.916396	-1.048691	-2.010857
C	-4.866926	0.696475	-0.715905
C	-5.981639	0.202435	-1.391267
H	-6.775942	-1.448949	-2.550053
H	-6.895240	0.796849	-1.433736
H	1.695253	0.397864	-0.831343
C	1.329503	-0.021205	1.223016
H	1.934835	-0.924166	1.159026
H	0.264184	-2.007635	2.248675
C	-1.796830	-2.280228	2.498742
C	-3.158806	-2.147359	2.141661
C	-1.499143	-2.874761	3.751781
C	-4.167171	-2.601320	2.989513
H	-3.425033	-1.688447	1.197282
C	-2.507520	-3.331850	4.596192
H	-0.454230	-2.983914	4.048259
C	-3.848502	-3.198344	4.215289
H	-5.211924	-2.489013	2.689665
H	-2.250500	-3.793827	5.552609
H	-4.643996	-3.554356	4.875168
H	-0.052601	-6.399069	4.035123
H	3.319814	2.097278	-4.133165
Cl	-4.688051	-3.353491	-2.741064
Cl	-4.998697	2.272777	0.050859
C	-1.479877	-7.000889	-0.854114
H	-1.717691	-7.762991	-0.093894
H	-2.160413	-7.153260	-1.707445
H	-0.451206	-7.192736	-1.198800
C	-3.099070	-5.349030	0.165615
H	-3.213531	-4.363112	0.633771
H	-3.772813	-5.382286	-0.703890
H	-3.430374	-6.117013	0.883503
C	3.891213	-4.462655	1.193776
H	4.078424	-5.198348	1.992488
H	3.689370	-5.022151	0.268818
H	4.813902	-3.880574	1.043956
C	3.084589	-2.714413	2.821306
H	3.416544	-3.354348	3.653480
H	3.914736	-2.030623	2.585639
H	2.242007	-2.106121	3.181034
C	5.170364	-2.411316	-2.555712
H	5.773762	-3.144505	-1.996557
H	4.729582	-2.922509	-3.426025
H	5.852865	-1.634820	-2.937155
C	4.720943	-1.110734	-0.430728
H	5.359574	-0.266856	-0.729099
H	3.953187	-0.719512	0.249682

H	5.347917	-1.820078	0.131019
C	-0.426656	-1.207446	-5.355535
H	-1.434280	-1.558891	-5.631095
H	-0.152193	-0.390360	-6.043343
H	0.281270	-2.035838	-5.520843
C	-1.441233	0.375578	-3.649495
H	-2.455764	-0.004390	-3.845211
H	-1.415815	0.725343	-2.607204
H	-1.274659	1.241902	-4.310005
C	-0.061262	2.978115	0.448853
C	1.121006	2.268360	0.248412
C	2.343195	2.931220	0.560499
C	2.270873	4.064258	1.373685
C	1.052439	4.571782	1.863612
C	-0.132909	4.131212	1.251118
C	1.062506	5.225643	3.216842
H	1.041707	6.316930	3.325654
C	2.321776	2.232768	3.994679
C	1.126284	2.955704	4.160401
C	-0.067583	2.310561	3.798718
H	-1.022934	2.805412	3.987239
C	-0.046559	1.166977	2.998987
C	1.174342	0.630155	2.562719
C	2.343308	1.071044	3.216264
H	3.305274	0.612445	2.972149
C	1.126390	4.447640	4.323035
H	1.173471	4.911681	5.315613
H	-0.981942	0.795729	2.578952
H	3.268342	2.665874	4.329423
H	3.194959	4.479336	1.780254
H	-0.994730	2.529404	0.121127
C	4.894159	3.231226	0.233439
H	5.076871	3.585384	1.262740
C	-2.353095	5.051016	0.378030
H	-1.845102	5.838338	-0.207596
H	-2.431083	4.173602	-0.285659
H	4.688396	4.135425	-0.366920
C	6.164032	2.550950	-0.283443
H	6.371175	1.652979	0.327244
H	5.988274	2.181017	-1.310609
C	7.396766	3.456543	-0.280996
H	7.577304	3.825705	0.745707
H	7.190104	4.355579	-0.890792
C	8.661029	2.769885	-0.802684
H	8.476308	2.397572	-1.827900
H	8.866810	1.871572	-0.190839
C	9.897312	3.671450	-0.808455
H	10.082399	4.044145	0.216531
H	9.691386	4.569455	-1.420738
C	11.161197	2.983277	-1.331232
H	10.972974	2.610415	-2.354799
H	11.364884	2.086416	-0.717811
C	12.390173	3.893309	-1.334315
H	12.621129	4.253538	-0.317484
H	12.226057	4.781318	-1.967991
C	-3.767390	5.500674	0.747180
H	-4.240861	4.710297	1.357961
H	-3.722241	6.395020	1.396415
C	-4.650327	5.789283	-0.468984
H	-4.231833	6.641586	-1.036088
H	-4.613264	4.921015	-1.150780
C	-6.110796	6.074579	-0.114146
H	-6.163424	6.938202	0.575183
H	-6.517020	5.213368	0.448546
C	-6.998614	6.339324	-1.331674
H	-6.593859	7.196604	-1.902063
H	-6.943071	5.471210	-2.015158
C	-8.463843	6.612103	-0.981624

H	-8.517734	7.479821	-0.298712	H	-2.049454	3.931290	2.190143
H	-8.864107	5.754951	-0.409674	C	3.674558	2.307775	0.198879
C	-9.343095	6.867290	-2.206585	H	3.594831	1.864212	-0.805736
H	-9.334180	6.001032	-2.889763	H	3.858725	1.451604	0.871107
H	-8.984969	7.740021	-2.778751	H	13.282983	3.371529	-1.714904
C	-1.498040	4.686458	1.599986	H	-10.390891	7.059726	-1.924439
H	-1.392270	5.565588	2.255771				

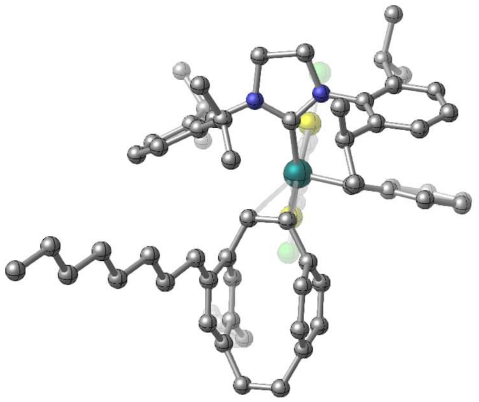

Zero-point correction= 1.448504 (Hartree/Particle)

Thermal correction to Energy= 1.531087

Thermal correction to Enthalpy= 1.532031

Thermal correction to Gibbs Free Energy= 1.324395

Sum of electronic and zero-point Energies= -4715.366786

Sum of electronic and thermal Energies= -4715.284203

Sum of electronic and thermal Enthalpies= -4715.283259

Sum of electronic and thermal Free Energies= -4715.490895

M06L-D3/def2tzvpp-SDD(Ru)-SMD(benzene).

E(scf) = -4719.91568965 a.u.

Ru-1a-M (^{n}Oct)

E(scf) = -4716.82095515 a.u.

ν_{min} = 8.48 cm^{-1}

S	-2.840706	-1.662005	-0.546641	C	-0.129888	-2.808983	-0.307503

Ru	-0.716606	-0.814935	-0.320199
C	1.906663	0.938739	-1.538142
C	-0.702958	-0.452605	1.491478
S	-1.490275	1.116269	-1.391894
N	-0.317202	-3.757057	0.638181
N	0.245347	-3.425765	-1.453431
C	-0.409730	-3.569550	2.058735
C	-0.067891	-5.122558	0.130557
C	0.165209	-4.892485	-1.368123
C	0.795930	-2.768606	-2.607311
H	1.089413	-5.356602	-1.738847
H	-0.672721	-5.255443	-1.984088
H	0.804573	-5.560773	0.638693
C	-0.498660	-3.498068	4.846406
C	-1.624739	-3.857569	2.716921
C	0.761144	-3.222092	2.780430
C	0.688585	-3.195114	4.179358
C	-1.642468	-3.815307	4.119319
H	1.577622	-2.939308	4.758986
H	-2.572832	-4.031998	4.648137
C	1.902110	-1.443265	-4.794596
C	2.200155	-2.584832	-2.665015
C	-0.049130	-2.371315	-3.668484
C	0.530239	-1.686014	-4.747004
C	2.730093	-1.910334	-3.773637
C	3.144832	-3.168814	-1.618541
C	-1.522842	-2.746869	-3.703924
H	-0.103459	-1.348670	-5.569203
H	3.807768	-1.746805	-3.839421
H	-0.934547	-5.766968	0.330049
H	2.538963	-3.513605	-0.770156
H	-1.823027	-3.017616	-2.683365
C	-2.896234	-4.219268	1.962221
H	-2.702774	-4.064998	0.891300

C	2.092060	-2.942752	2.090735
H	1.882007	-2.710632	1.038805
C	-3.830916	-0.346104	-1.190498
C	-5.223006	-0.500322	-1.350514
C	-3.240773	0.898986	-1.526002
C	-6.028846	0.539895	-1.810220
C	-4.076082	1.944043	-1.971376
C	-5.451852	1.774548	-2.118831
H	-7.103822	0.387148	-1.915034
H	-6.068735	2.605799	-2.461567
H	1.795747	0.167045	-2.305739
C	1.769323	0.598306	-0.229231
H	1.619629	-0.469858	0.019081
H	0.301612	-0.397071	1.949302
C	-1.750779	-0.122485	2.453565
C	-3.008673	0.406177	2.089477
C	-1.484332	-0.313950	3.829261
C	-3.973321	0.683915	3.056569
H	-3.213340	0.631443	1.047629
C	-2.457445	-0.055818	4.793226
H	-0.509249	-0.699947	4.131545
C	-3.710169	0.439082	4.409605
H	-4.935763	1.099353	2.749404
H	-2.237959	-0.238000	5.848358
H	-4.472475	0.649079	5.164296
H	-0.533219	-3.473168	5.938460
H	2.332381	-0.904206	-5.642305
Cl	-5.983916	-2.040493	-0.972068
Cl	-3.391640	3.519275	-2.350694
C	-3.270362	-5.697567	2.172233
H	-3.506945	-5.899193	3.229986
H	-4.158444	-5.958161	1.574038
H	-2.452013	-6.376224	1.883051
C	-4.066459	-3.298621	2.346458

H	-3.785782	-2.241885	2.247195	H	2.812610	7.187046	-0.398890
H	-4.924170	-3.476384	1.679646	C	3.247161	3.490130	1.613588
H	-4.397880	-3.477298	3.382423	C	2.155935	4.369104	1.520720
C	3.001624	-4.185348	2.123084	C	0.858953	3.819101	1.410338
H	3.260605	-4.448243	3.161478	C	0.771636	2.464546	1.070089
H	2.517248	-5.062602	1.669967	C	1.896640	1.661233	0.832408
H	3.938642	-3.996243	1.575570	C	3.155438	2.131345	1.274437
C	2.838006	-1.728599	2.660456	C	2.412124	5.805520	1.163102
H	3.172610	-1.894738	3.696301	H	2.392317	6.593579	1.925950
H	3.734447	-1.532232	2.055225	H	4.693171	4.525637	-1.036547
H	2.219109	-0.818912	2.646153	H	0.145626	2.953165	-2.237796
C	3.881177	-4.396353	-2.187982	H	4.347331	2.175583	-1.676229
H	4.501374	-4.870272	-1.409970	H	0.497686	5.299842	-1.635356
H	3.179735	-5.151662	-2.575210	H	4.235271	3.924884	1.773851
H	4.543350	-4.104066	-3.019022	H	-0.197932	2.062462	0.793125
C	4.150176	-2.147543	-1.066650	C	5.745921	1.920867	1.078521
H	4.845185	-1.794748	-1.844966	H	5.967407	2.527255	1.974013
H	3.642643	-1.268305	-0.651929	H	5.725406	2.627388	0.231436
H	4.757530	-2.601962	-0.268250	C	6.874595	0.908588	0.868914
C	-1.727446	-3.980975	-4.603310	H	6.884274	0.191161	1.710766
H	-2.782606	-4.298978	-4.584506	H	6.658558	0.306136	-0.033532
H	-1.459146	-3.755012	-5.648682	C	8.257750	1.547018	0.730023
H	-1.107312	-4.831858	-4.278993	H	8.476690	2.144877	1.634255
C	-2.433394	-1.591716	-4.141444	H	8.243412	2.267357	-0.109099
H	-3.487991	-1.863236	-3.980667	C	9.383048	0.533988	0.508032
H	-2.227355	-0.684535	-3.557952	H	9.396059	-0.186156	1.347544
H	-2.309828	-1.353383	-5.210367	H	9.161870	-0.063999	-0.395938
C	1.161084	3.297432	-2.037898	C	10.767571	1.169983	0.366402
C	2.203313	2.364191	-1.896967	H	10.988839	1.768197	1.270266
C	3.502914	2.868003	-1.720094	H	10.754426	1.890175	-0.473205
C	3.699776	4.204335	-1.360238	C	11.893408	0.156414	0.144110
C	2.601414	5.059956	-1.173779	H	11.904070	-0.562412	0.983915
C	1.362971	4.633899	-1.685109	H	11.669673	-0.440511	-0.759083
C	2.640719	6.137964	-0.129487	C	13.272269	0.802669	0.003819

H	13.299359	1.501095	-0.849717	H	-6.419312	2.619756	0.360118
H	13.535842	1.377999	0.907470	C	-7.946630	4.111495	0.060839
C	-1.704288	3.927984	1.365136	H	-7.690623	4.305642	-0.997242
H	-1.793143	3.293640	2.265464	H	-8.243303	5.089624	0.482010
H	-1.691188	3.231949	0.513231	C	-9.123661	3.137578	0.130315
C	-2.964231	4.778975	1.206642	H	-9.421372	2.945942	1.175219
H	-3.020378	5.560580	1.986966	H	-8.856897	2.165065	-0.317619
H	-2.916840	5.309076	0.238034	C	-0.382751	4.695266	1.410320
C	-4.218344	3.898362	1.245255	H	-0.325617	5.374736	0.540713
H	-4.387094	3.552880	2.281022	H	-0.358495	5.359640	2.292871
H	-4.020445	2.983499	0.660159	C	4.381112	1.242982	1.222867
C	-5.492509	4.538092	0.697605	H	4.254481	0.525028	0.397088
H	-5.710515	5.482663	1.230458	H	4.398903	0.624834	2.138240
H	-5.321246	4.812334	-0.359246	H	14.061176	0.049943	-0.155085
C	-6.699809	3.600353	0.785203	H	-10.007199	3.522289	-0.404363
H	-6.942612	3.409848	1.847737				

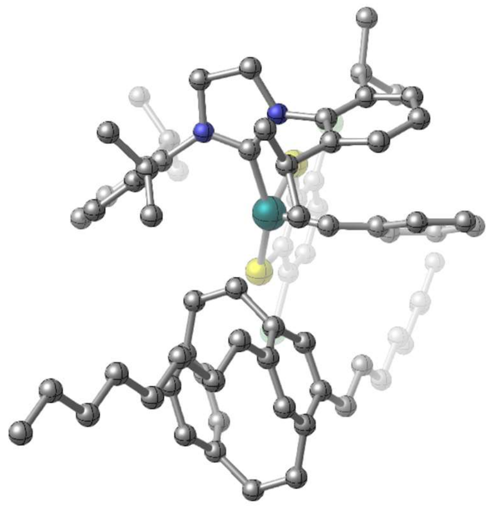

Zero-point correction= 1.447105 (Hartree/Particle)

Thermal correction to Energy= 1.530327

Thermal correction to Enthalpy= 1.531271

Thermal correction to Gibbs Free Energy= 1.321810

Sum of electronic and zero-point Energies= -4715.373850

Sum of electronic and thermal Energies= -4715.290628

Sum of electronic and thermal Enthalpies= -4715.289684

Sum of electronic and thermal Free Energies= -4715.499145

M06L-D3/def2tzvpp-SDD(Ru)-SMD(benzene).

E(scf) = -4719.92137858 a.u.

TS1-α1 (^{n}Oct)

E(scf) = -4716.80036051 a.u.

ν_{min} = -172.19 cm^{-1}

S	2.234589	1.874518	-0.969221	C	-0.635501	0.095427	-3.560711
C	-0.532606	2.348626	-0.962753	C	-1.244000	-1.036092	-4.126230
Ru	0.522169	0.837512	0.130951	C	-3.429413	-0.255351	-3.467268
C	-1.139519	-0.523261	-0.009553	C	-3.791706	1.928071	-2.248373
C	0.013498	1.533028	1.842785	C	0.852260	0.328928	-3.767817
S	2.229498	-0.733330	0.810635	H	-0.622431	-1.777298	-4.633109
N	-0.462366	3.688479	-0.728822	H	-4.513344	-0.390308	-3.456609
N	-0.905657	2.192397	-2.261460	H	0.037714	5.226348	-2.109746
C	-0.382803	4.360309	0.541658	H	-3.168434	2.723322	-1.820362
C	-0.768684	4.505612	-1.920775	H	1.135608	1.206743	-3.176113
C	-0.890579	3.453337	-3.023363	C	2.010359	5.158128	-0.011102
C	-1.459145	1.020626	-2.884086	H	1.861108	4.468867	-0.850997
H	-1.802877	3.559352	-3.626455	C	-2.842949	3.702025	0.998486
H	-0.025978	3.454414	-3.707447	H	-2.614119	2.908030	0.275029
H	-1.698607	5.073041	-1.762389	C	3.747975	1.026629	-0.651315
C	-0.282873	5.732779	2.979098	C	4.963835	1.479196	-1.199900
C	0.791145	5.063347	0.895457	C	3.740038	-0.117621	0.182384
C	-1.527560	4.375890	1.384701	C	6.166907	0.825905	-0.941104
C	-1.446915	5.063372	2.602173	C	4.977564	-0.747926	0.454709
C	0.816820	5.736458	2.127875	C	6.170582	-0.293029	-0.100973
H	-2.309078	5.077866	3.270564	H	7.092645	1.192829	-1.385729
H	1.720622	6.274194	2.421357	H	7.103647	-0.809799	0.128219
C	-2.623626	-1.219898	-4.072439	H	-1.878291	-0.039440	-0.647774
C	-2.869493	0.878879	-2.864753	C	-1.241861	-0.020035	1.405966

H	-2.087329	0.666010	1.505710
H	-0.803042	2.252986	1.907910
C	0.747800	1.503635	3.126179
C	2.153015	1.545184	3.200483
C	0.009279	1.524392	4.328003
C	2.797122	1.586966	4.438615
H	2.733844	1.567375	2.280258
C	0.654188	1.534586	5.564636
H	-1.082501	1.508479	4.285794
C	2.052784	1.566135	5.622869
H	3.888364	1.630769	4.476232
H	0.065708	1.524687	6.485459
H	2.560659	1.583942	6.590565
H	-0.238740	6.257371	3.936748
H	-3.076084	-2.109016	-4.518451
Cl	4.990844	2.891678	-2.250354
Cl	5.043244	-2.128675	1.539618
C	2.162605	6.579932	-0.582108
H	2.355513	7.312939	0.218400
H	3.009136	6.620186	-1.286601
H	1.256520	6.907206	-1.116882
C	3.299929	4.715716	0.699904
H	3.173961	3.728588	1.166197
H	4.122510	4.638654	-0.027203
H	3.601035	5.431717	1.481621
C	-3.784389	4.710025	0.312218
H	-4.025886	5.536531	0.999768
H	-3.332580	5.149007	-0.588424
H	-4.728234	4.227262	0.015720
C	-3.565992	3.034140	2.180142
H	-4.004627	3.775140	2.866293
H	-4.390309	2.406704	1.808581
H	-2.891879	2.395875	2.770701
C	-4.686262	2.578692	-3.318179
H	-5.280782	3.395311	-2.877604
H	-4.090199	2.996042	-4.145011
H	-5.388420	1.847990	-3.750715
C	-4.637785	1.362965	-1.098723
H	-5.291039	0.547032	-1.438139
H	-4.006541	0.966402	-0.291607
H	-5.281408	2.147156	-0.672186
C	1.144025	0.654948	-5.243741
H	2.211104	0.896753	-5.376738
H	0.906439	-0.198357	-5.900304
H	0.552306	1.518077	-5.589618
C	1.718912	-0.838333	-3.273505
H	2.785890	-0.578734	-3.356675
H	1.518099	-1.066859	-2.218040
H	1.547977	-1.755015	-3.860387
C	0.012892	-2.811882	-0.198673
C	-1.141128	-2.026057	-0.185466
C	-2.385563	-2.702864	-0.048883
C	-2.350505	-4.015260	0.430345
C	-1.154183	-4.677117	0.754585
C	0.050841	-4.135553	0.268369
C	-1.195485	-5.671163	1.878213
H	-1.134510	-6.750771	1.693179
C	-2.478114	-2.955241	3.333466
C	-1.309011	-3.734258	3.388871
C	-0.085683	-3.047165	3.320675
H	0.850265	-3.594867	3.455826
C	-0.032295	-1.725397	2.882265
C	-1.200839	-1.056443	2.495437
C	-2.424174	-1.624916	2.901148
H	-3.360496	-1.090618	2.718491
C	-1.314718	-5.216475	3.147751
H	-1.370759	-5.926949	3.981404
H	0.939325	-1.268668	2.710855

H	-3.454169	-3.430919	3.460988	C	3.852147	-5.032642	-0.237017
H	-3.284010	-4.493429	0.728634	H	4.126427	-4.851699	0.817544
H	0.951506	-2.319323	-0.423448	H	3.803366	-6.130711	-0.359649
C	-4.975036	-2.809169	-0.096276	C	4.943294	-4.446629	-1.138262
H	-5.050965	-3.210983	0.929643	H	4.755208	-4.739241	-2.188218
C	2.487745	-4.395882	-0.500858	H	4.862846	-3.345446	-1.113415
H	2.171169	-4.591078	-1.541794	C	6.367850	-4.833724	-0.740487
H	2.620478	-3.306629	-0.418303	H	6.491091	-5.931624	-0.796894
H	-4.921095	-3.687153	-0.764191	H	6.525696	-4.560284	0.318581
C	-6.243768	-2.014593	-0.413052	C	7.439031	-4.149869	-1.592918
H	-6.281615	-1.112619	0.225272	H	7.289288	-4.413985	-2.656857
H	-6.191732	-1.643735	-1.453400	H	7.296806	-3.054155	-1.533632
C	-7.533550	-2.816502	-0.230574	C	8.873159	-4.493009	-1.182805
H	-7.588836	-3.186044	0.810313	H	9.017313	-5.587332	-1.244919
H	-7.495036	-3.719876	-0.867253	H	9.015984	-4.231548	-0.118171
C	-8.800915	-2.022034	-0.553620	C	9.931184	-3.785849	-2.030983
H	-8.744838	-1.653775	-1.595227	H	9.829510	-2.689513	-1.959353
H	-8.835575	-1.117347	0.082030	H	9.832440	-4.055083	-3.096361
C	-10.093777	-2.819258	-0.368433	C	1.382508	-4.837246	0.463516
H	-10.148873	-3.188419	0.672944	H	1.237332	-5.929365	0.398579
H	-10.059577	-3.723411	-1.004881	H	1.723964	-4.649794	1.499464
C	-11.362028	-2.023826	-0.689394	C	-3.700960	-1.975480	-0.232563
H	-11.304553	-1.655614	-1.730181	H	-3.691448	-1.494202	-1.222901
H	-11.393360	-1.120599	-0.052734	H	-3.756723	-1.141576	0.486832
C	-12.647556	-2.830194	-0.500222	H	-13.542427	-2.232802	-0.737986
H	-12.746950	-3.182053	0.540632	H	10.953225	-4.048395	-1.713072
H	-12.657599	-3.721294	-1.150513				

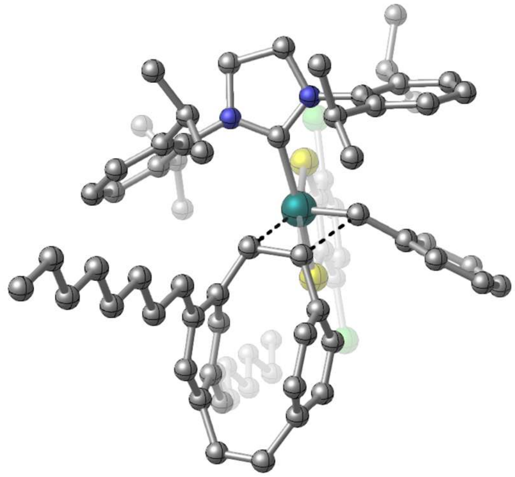

Zero-point correction= 1.449452 (Hartree/Particle)

Thermal correction to Energy= 1.530777

Thermal correction to Enthalpy= 1.531721

Thermal correction to Gibbs Free Energy= 1.327874

Sum of electronic and zero-point Energies= -4715.350909

Sum of electronic and thermal Energies= -4715.269584

Sum of electronic and thermal Enthalpies= -4715.268639

Sum of electronic and thermal Free Energies= -4715.472487

M06L-D3/def2tzvpp-SDD(Ru)-SMD(benzene).

E(scf) = -4719.90621517 a.u.

TS1-β1 (^{n}Oct)

E(scf) = -4716.80382468 a.u.

ν_{min} = -213.01 cm^{-1}

S	-2.064143	-2.594718	-0.306611	N	1.247603	-3.319365	-1.197104
C	0.770462	-2.679596	-0.097025	C	0.758166	-3.280578	2.339489
Ru	-0.482165	-0.952311	-0.244195	C	1.152218	-4.939717	0.498256
C	1.116837	0.154599	-1.136461	C	1.575493	-4.736441	-0.960026
C	-0.132429	-0.325491	1.537039	C	1.612866	-2.731054	-2.459311
S	-2.269917	0.494099	-0.956220	H	2.648608	-4.917034	-1.127967
N	0.813177	-3.568738	0.932243	H	1.012472	-5.371666	-1.659483

H	1.945453	-5.363666	1.129680	H	1.949246	0.210574	0.833499
C	0.753591	-2.818408	5.099231	H	0.676791	-0.763833	2.126489
C	-0.336094	-3.721878	3.114936	C	-1.004059	0.491040	2.411423
C	1.864814	-2.620099	2.940691	C	-2.410021	0.456855	2.346972
C	1.832960	-2.395574	4.322409	C	-0.395199	1.315182	3.381184
C	-0.313176	-3.476284	4.498166	C	-3.179295	1.253063	3.197822
H	2.664401	-1.882662	4.806739	H	-2.896173	-0.210549	1.638814
H	-1.154053	-3.807069	5.111073	C	-1.163511	2.127248	4.215081
C	2.358479	-1.529856	-4.866570	H	0.695132	1.338052	3.454219
C	2.943727	-2.268630	-2.622985	C	-2.560410	2.103239	4.120272
C	0.679361	-2.670136	-3.516153	H	-4.269315	1.214865	3.131524
C	1.074655	-2.045113	-4.709739	H	-0.673222	2.781095	4.940634
C	3.289237	-1.660277	-3.836078	H	-3.165298	2.738945	4.771838
C	4.015530	-2.457134	-1.551914	H	0.748896	-2.632402	6.176112
C	-0.691624	-3.322675	-3.432488	H	2.641662	-1.038079	-5.800421
H	0.361674	-1.970779	-5.533439	Cl	-4.709862	-4.398840	-0.410802
H	4.303982	-1.281714	-3.977853	Cl	-5.168484	1.763502	-1.586138
H	0.267737	-5.588370	0.586060	C	-1.534523	-5.939175	2.964511
H	3.530509	-2.867585	-0.658606	H	-1.655117	-6.032153	4.056434
H	-0.852369	-3.629723	-2.392057	H	-2.370051	-6.475832	2.486573
C	-1.527483	-4.463778	2.527198	H	-0.597751	-6.450117	2.689701
H	-1.438343	-4.434763	1.434885	C	-2.858056	-3.776076	2.875263
C	3.080991	-2.172538	2.131656	H	-2.828979	-2.709851	2.607418
H	2.729274	-1.904018	1.124685	H	-3.680520	-4.239363	2.309964
C	-3.632929	-1.877701	-0.675533	H	-3.092250	-3.858233	3.948912
C	-4.800734	-2.665653	-0.705314	C	4.094647	-3.321741	1.981508
C	-3.719701	-0.489504	-0.943030	H	4.448859	-3.650309	2.971670
C	-6.047411	-2.109132	-0.980585	H	3.658370	-4.194201	1.476403
C	-4.995935	0.052473	-1.225051	H	4.969474	-2.997575	1.396704
C	-6.142322	-0.737744	-1.239592	C	3.777739	-0.929982	2.703986
H	-6.935221	-2.742381	-0.994848	H	4.330590	-1.157242	3.628803
H	-7.108882	-0.281313	-1.457515	H	4.504617	-0.537211	1.977846
H	1.935671	-0.543835	-1.293717	H	3.059319	-0.128779	2.932862
C	1.112438	0.648671	0.291690	C	5.068972	-3.480217	-2.014966

H	5.786036	-3.691107	-1.205055
H	4.604133	-4.431152	-2.318662
H	5.636974	-3.098585	-2.878660
C	4.690406	-1.144720	-1.123855
H	5.191836	-0.645164	-1.967238
H	3.963774	-0.438024	-0.701604
H	5.453171	-1.337152	-0.353301
C	-0.733719	-4.592106	-4.302890
H	-1.705710	-5.099890	-4.192260
H	-0.597658	-4.350965	-5.370072
H	0.058415	-5.303920	-4.018916
C	-1.833030	-2.361606	-3.796824
H	-2.805796	-2.841003	-3.605986
H	-1.791445	-1.444581	-3.191296
H	-1.802799	-2.076145	-4.860650
C	-0.008113	1.816824	-2.790049
C	1.117720	1.209278	-2.216084
C	2.356794	1.843567	-2.451424
C	2.407624	3.173611	-2.873757
C	1.222822	3.892404	-3.110746
C	0.043251	3.140205	-3.235620
C	1.144085	5.367977	-2.846052
H	1.196868	6.097304	-3.663643
C	2.092611	4.225105	0.010304
C	0.892256	4.777238	-0.470561
C	-0.314061	4.115598	-0.166443
C	-0.223354	2.795901	0.294749
C	0.992367	2.137530	0.471326
C	2.180505	2.913780	0.490480
C	0.962582	5.794242	-1.574278
H	0.865975	6.868027	-1.371509
H	3.364725	3.701404	-2.848644
H	-0.970324	1.310943	-2.751722
H	3.281647	1.358049	-2.132007

H	-0.887754	3.636242	-3.522381
H	3.011182	4.788642	-0.158997
H	-1.136552	2.214939	0.378866
C	4.772204	3.130151	0.665844
H	4.707330	4.039635	1.288775
H	4.846955	3.478030	-0.379700
C	6.040287	2.351876	1.027318
H	5.967523	1.998707	2.072825
H	6.093437	1.437308	0.407012
C	7.330472	3.154490	0.852409
H	7.280662	4.066611	1.475607
H	7.400433	3.508976	-0.192812
C	8.594351	2.367300	1.205768
H	8.522244	2.012108	2.250844
H	8.641161	1.454788	0.582130
C	9.888402	3.165030	1.031538
H	9.841865	4.077147	1.655706
H	9.960192	3.520898	-0.013444
C	11.152502	2.376322	1.384037
H	11.077786	2.020955	2.428176
H	11.196450	1.465291	0.759338
C	12.439628	3.182874	1.207048
H	12.556375	3.522123	0.163816
H	12.436885	4.081876	1.846437
C	-2.801936	4.251376	0.420399
H	-2.553233	4.532362	1.459819
H	-2.875340	3.152761	0.410347
C	-4.174679	4.805567	0.042121
H	-4.166555	5.911368	0.068247
H	-4.393021	4.527895	-1.005193
C	-5.290553	4.270279	0.943132
H	-5.133281	4.628286	1.977818
H	-5.210288	3.169715	0.986800
C	-6.698642	4.641213	0.476927

H	-6.801012	5.742021	0.437088	H	-10.255616	2.709714	1.784193
H	-6.836234	4.285193	-0.560856	C	-1.670768	4.694434	-0.515364
C	-7.806541	4.055478	1.354464	H	-1.928269	4.391967	-1.547576
H	-7.668512	4.396280	2.398057	H	-1.609514	5.795403	-0.537224
H	-7.699338	2.954638	1.381264	C	3.513866	2.281363	0.835921
C	-9.220597	4.412350	0.889116	H	3.645285	1.374074	0.225064
H	-9.353443	4.073169	-0.154602	H	3.470988	1.917135	1.877452
H	-9.326800	5.512454	0.862895	H	13.331287	2.590197	1.467574
C	-10.318377	3.811095	1.767829	H	-11.324183	4.083334	1.408983
H	-10.229337	4.160247	2.810659				

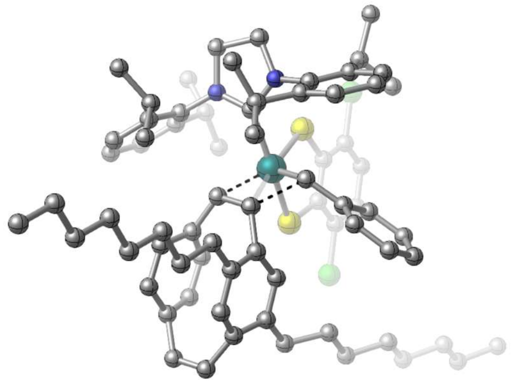

Zero-point correction= 1.449344 (Hartree/Particle)

Thermal correction to Energy= 1.530811

Thermal correction to Enthalpy= 1.531755

Thermal correction to Gibbs Free Energy= 1.326717

Sum of electronic and zero-point Energies= -4715.354481

Sum of electronic and thermal Energies= -4715.273014

Sum of electronic and thermal Enthalpies= -4715.272070

Sum of electronic and thermal Free Energies= -4715.477108

M06L-D3/def2tzvpp-SDD(Ru)-SMD(benzene).

E(scf) = -4719.91168544 a.u.

Int1-α1 (^{n}Oct)

E(scf) = -4716.87849069 a.u.

ν_{min} = 9.66 cm^{-1}

S	-3.084863	-0.060165	0.712335	H	-4.253132	-2.300824	2.368211
C	-1.584444	-2.669357	0.908729	C	-0.277825	-0.040845	2.969532
Ru	-2.015862	-1.472710	-0.721973	H	-1.217407	-0.531805	2.681388
C	-0.450409	-0.674630	-1.215001	C	1.610622	-4.175127	0.579565
S	-3.611325	-0.956163	-2.333731	H	0.602471	-4.531441	0.832665
N	-0.569854	-2.715992	1.792570	C	-4.410643	0.653263	-0.216700
N	-2.512348	-3.581414	1.278895	C	-5.290074	1.570213	0.390326
C	0.698584	-2.055089	1.717091	C	-4.626984	0.285485	-1.568080
C	-0.850471	-3.629871	2.919107	C	-6.358102	2.133345	-0.301520
C	-2.077308	-4.400569	2.422881	C	-5.697585	0.887872	-2.261321
C	-3.620480	-3.988031	0.459219	C	-6.557722	1.795634	-1.641918
H	-1.832232	-5.419277	2.076927	H	-7.018933	2.841719	0.198378
H	-2.869444	-4.475516	3.178239	H	-7.380764	2.232993	-2.208352
H	0.016814	-4.272933	3.124426	H	0.087828	-1.454383	-1.807180
C	3.218639	-0.881564	1.666423	C	0.151608	0.648604	-1.233355
C	0.859205	-0.762140	2.259636	C	-0.636756	1.798643	-0.997245
C	1.783897	-2.782438	1.176649	C	1.538922	0.817792	-1.514030
C	3.046077	-2.175095	1.176785	C	-0.114190	3.091815	-0.970735
C	2.135426	-0.183628	2.197354	H	-1.697140	1.643126	-0.817930
H	3.904188	-2.712448	0.770102	C	2.051475	2.111957	-1.478584
H	2.283964	0.826464	2.581751	C	1.278638	3.249738	-1.185937
C	-5.771145	-4.813032	-1.105495	H	3.104640	2.266351	-1.693350
C	-3.373123	-4.780971	-0.688714	H	4.203589	-0.414595	1.627010
C	-4.929090	-3.609943	0.836974	H	-6.615004	-5.131230	-1.722806
C	-5.993582	-4.037321	0.030989	Cl	-5.052143	2.016248	2.077145
C	-4.473540	-5.181489	-1.457807	Cl	-5.999827	0.487054	-3.943807
C	-1.973119	-5.224730	-1.101066	C	-0.123052	-0.184983	4.496103
C	-5.201834	-2.778293	2.082502	H	0.796101	0.315474	4.843226
H	-7.013965	-3.753062	0.294345	H	-0.978514	0.276286	5.015663
H	-4.312688	-5.790028	-2.350154	H	-0.061950	-1.239927	4.806413
H	-1.063565	-3.039189	3.824318	C	-0.400928	1.437202	2.575899
H	-1.244239	-4.684231	-0.482895	H	-0.463494	1.554089	1.487211

H	-1.316256	1.866215	3.012177	H	2.859447	3.399933	1.505926
H	0.453232	2.030798	2.941227	C	6.319151	2.821796	-0.156137
C	2.605926	-5.191613	1.159861	H	5.682153	4.244770	-1.639018
H	3.644048	-4.952482	0.879033	C	6.014202	2.129652	1.034416
H	2.553782	-5.221579	2.259823	H	4.484728	1.848562	2.539600
H	2.386389	-6.201438	0.777842	H	7.266983	2.635266	-0.661142
C	1.688491	-4.131607	-0.955942	C	6.897907	1.124584	1.649572
H	2.672775	-3.771703	-1.294718	H	6.360374	0.378862	2.247272
H	1.527643	-5.135338	-1.380510	C	8.237184	0.941713	1.577510
H	0.923515	-3.460390	-1.371709	H	8.613976	0.026774	2.049775
C	-1.767578	-6.726590	-0.835757	C	9.291799	1.780307	0.975642
H	-0.735038	-7.025162	-1.080603	C	9.267122	3.188460	1.033259
H	-1.955879	-6.979801	0.219519	C	10.407205	1.160044	0.378056
H	-2.451144	-7.333825	-1.451169	C	10.301515	3.945444	0.482006
C	-1.655754	-4.876470	-2.564990	H	8.429058	3.688903	1.521838
H	-2.280010	-5.453229	-3.265378	C	11.438045	1.917680	-0.182943
H	-1.826973	-3.808809	-2.773167	H	10.459213	0.069936	0.343201
H	-0.603555	-5.107159	-2.792152	C	11.388093	3.314655	-0.136986
C	-5.673134	-3.669061	3.247651	H	10.263326	5.036337	0.540857
H	-5.817130	-3.067893	4.160176	H	12.286437	1.413195	-0.652793
H	-6.634920	-4.149730	3.003607	H	12.195867	3.909752	-0.570370
H	-4.955449	-4.471846	3.477218	C	3.946673	-0.054974	-1.747142
C	-6.217545	-1.653478	1.826797	H	4.224912	0.709727	-2.492070
H	-6.213706	-0.937949	2.663368	H	4.164004	0.395290	-0.763871
H	-5.975545	-1.098289	0.911734	C	4.853395	-1.271096	-1.943307
H	-7.242964	-2.045635	1.729667	H	4.501744	-2.103978	-1.310098
C	1.921189	4.577118	-1.161913	H	4.781323	-1.634768	-2.984955
H	1.320588	5.411069	-1.539548	C	6.308839	-0.956217	-1.591718
C	3.181450	4.870171	-0.770955	H	6.351793	-0.598756	-0.547316
H	3.538060	5.893663	-0.939848	H	6.657479	-0.103266	-2.203212
C	4.152331	3.947402	-0.137015	C	7.278775	-2.126485	-1.757135
C	3.837776	3.250900	1.043335	H	6.895831	-3.003005	-1.200848
C	5.418429	3.726703	-0.712987	H	7.313884	-2.435537	-2.818483
C	4.751430	2.368976	1.615982	C	8.690642	-1.796573	-1.267501

H	8.640618	-1.524498	-0.197191
H	9.053550	-0.888247	-1.783481
C	9.706267	-2.924883	-1.457036
H	9.335199	-3.837134	-0.954762
H	9.770942	-3.178388	-2.530957
C	11.098280	-2.577439	-0.926210
H	11.071091	-2.373090	0.157744
H	11.499242	-1.676441	-1.420707
C	-2.476533	4.059952	-0.480083
H	-2.899737	3.534348	-1.354144
H	-2.636992	3.386272	0.379222
C	-3.246395	5.365614	-0.257290
H	-3.071890	6.043701	-1.113946
H	-2.834824	5.887663	0.627013
C	-4.747901	5.154424	-0.072109
H	-5.146427	4.613022	-0.948065
H	-4.911126	4.484048	0.787762
C	-5.551383	6.438236	0.135042
H	-5.409162	7.110637	-0.731857
H	-5.153571	6.986515	1.009746
C	-7.046574	6.179411	0.337755
H	-7.440673	5.624490	-0.534531
H	-7.183034	5.506463	1.205465
C	-7.878037	7.447390	0.546629
H	-7.740294	8.117645	-0.321604
H	-7.482375	8.000176	1.418389
C	-9.367979	7.167918	0.748585
H	-9.796245	6.645311	-0.123598
H	-9.536216	6.526611	1.630435
C	-0.982425	4.302518	-0.691694
H	-0.861995	5.019459	-1.525287
H	-0.574932	4.828749	0.191226
C	2.448809	-0.357821	-1.824113
H	2.200078	-0.758297	-2.825572
H	2.226959	-1.171112	-1.119051
H	-9.941823	8.097030	0.896452
H	11.816092	-3.396886	-1.091391

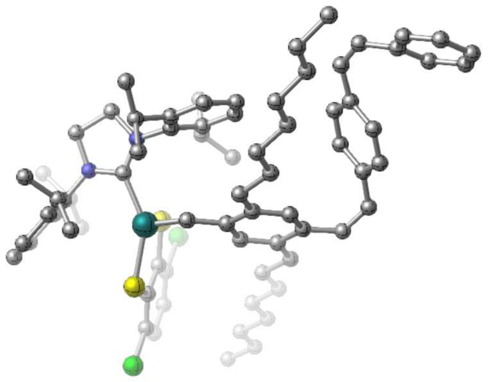

Zero-point correction= 1.448642 (Hartree/Particle)

Thermal correction to Energy= 1.532309

Thermal correction to Enthalpy= 1.533253

Thermal correction to Gibbs Free Energy= 1.320746

Sum of electronic and zero-point Energies= -4715.429849

Sum of electronic and thermal Energies= -4715.346182

Sum of electronic and thermal Enthalpies= -4715.345238

Sum of electronic and thermal Free Energies= -4715.557744

M06L-D3/def2tzvpp-SDD(Ru)-SMD(benzene).

E(scf) = -4719.98799260 a.u.

Int1-β1 (^{n}Oct)

E(scf) = -4716.87411274 a.u.

ν_{min} = 6.21 cm^{-1}

S	-3.831405	0.187180	2.037320	C	-1.193517	3.429539	-1.523859
C	-4.031582	2.064915	-0.337161	C	-1.168019	3.360881	0.945570
Ru	-3.566190	0.062609	-0.219246	C	0.217114	3.576163	0.906445
C	-1.754417	0.066650	-0.439610	C	0.192699	3.639661	-1.508018
C	8.080940	-0.758502	-3.205063	C	-1.934891	3.389126	-2.855729
S	-4.316390	-2.139805	-0.138235	C	-1.880455	3.237113	2.285240
N	-5.332301	2.411294	-0.455443	H	0.777464	3.627533	1.841809
N	-3.289795	3.185946	-0.290794	H	0.731471	3.747231	-2.452311
C	-6.359855	1.464676	-0.796354	H	-6.270299	4.223692	0.146702
C	-5.529475	3.864263	-0.580186	H	-2.991103	3.172521	-2.646281
C	-4.117539	4.409634	-0.310507	H	-2.896638	2.865939	2.090992
C	-1.860278	3.296753	-0.283409	C	-7.182034	1.537774	1.633185
H	-3.763751	5.099971	-1.089493	H	-6.176767	1.958740	1.778591
H	-4.041771	4.921943	0.661134	C	-5.533724	1.529818	-3.239452
H	-5.895176	4.106670	-1.591851	H	-4.783000	2.186940	-2.781402
C	-8.287150	-0.430779	-1.458290	C	-4.274883	-1.447085	2.552043
C	-7.243015	1.007248	0.208014	C	-4.462026	-1.738950	3.917689
C	-6.443482	1.004891	-2.133737	C	-4.457670	-2.478940	1.597476
C	-7.419751	0.047718	-2.439512	C	-4.790824	-3.019742	4.355397
C	-8.202846	0.050910	-0.152574	C	-4.773482	-3.772613	2.062391
H	-7.499090	-0.333729	-3.459824	C	-4.940826	-4.046877	3.419819
H	-8.890610	-0.331213	0.603638	H	-4.925519	-3.211223	5.420825
C	0.892169	3.720890	-0.304346	H	-5.190725	-5.058934	3.740651

H	-1.493376	0.298646	-1.497542
C	6.793074	-0.558177	-2.863766
H	6.124094	-0.181279	-3.647775
H	8.386597	-0.456603	-4.213904
C	9.151961	-1.332874	-2.365966
C	10.453426	-0.799958	-2.432678
C	8.919915	-2.423725	-1.505049
C	11.480459	-1.311176	-1.636035
H	10.653751	0.038856	-3.105073
C	9.950674	-2.946021	-0.721488
H	7.921881	-2.861784	-1.454265
C	11.232460	-2.386118	-0.775518
H	12.478365	-0.868059	-1.685643
H	9.751325	-3.793608	-0.060780
H	12.035827	-2.788877	-0.153673
H	-9.035437	-1.185283	-1.713504
H	1.972547	3.885631	-0.310373
Cl	-4.292788	-0.458957	5.113359
Cl	-4.982316	-5.094945	0.923126
C	-8.213197	2.664007	1.837779
H	-9.238876	2.279329	1.712451
H	-8.130279	3.084553	2.853229
H	-8.079737	3.484567	1.115762
C	-7.370038	0.437321	2.689127
H	-6.739894	-0.435653	2.473222
H	-7.087663	0.813910	3.684418
H	-8.418342	0.101727	2.746371
C	-6.327757	2.382915	-4.244690
H	-7.081639	1.775461	-4.771098
H	-6.856491	3.208831	-3.742922
H	-5.653920	2.815701	-5.002028
C	-4.763715	0.410319	-3.956874
H	-5.440138	-0.272177	-4.493811
H	-4.066286	0.838095	-4.693682
H	-4.179451	-0.201671	-3.253814
C	-1.878380	4.752873	-3.566749
H	-2.485248	4.735141	-4.486467
H	-2.257341	5.561533	-2.921954
H	-0.845366	5.011384	-3.850466
C	-1.424874	2.268683	-3.776795
H	-0.363028	2.407180	-4.035069
H	-1.531887	1.280283	-3.305968
H	-1.997589	2.256455	-4.717604
C	-2.004983	4.611999	2.967454
H	-2.566988	4.524657	3.911354
H	-1.009766	5.024080	3.202593
H	-2.522928	5.343633	2.327175
C	-1.199235	2.223322	3.218129
H	-1.825974	2.048444	4.106200
H	-1.057026	1.256766	2.717194
H	-0.217949	2.585967	3.564892
C	-0.700293	-1.187361	1.470492
C	-0.609535	-0.288898	0.384283
C	0.658022	0.251817	0.075885
C	1.786188	-0.088512	0.811631
C	1.704634	-1.017537	1.868767
C	0.433409	-1.535736	2.195688
C	2.880909	-1.471871	2.624559
H	2.674485	-1.768152	3.659660
C	5.829141	-0.443353	0.814189
C	4.757567	-1.352708	0.866798
C	4.365568	-2.008281	-0.327826
C	5.075141	-1.701363	-1.495843
C	6.148733	-0.796767	-1.544983
C	6.529417	-0.128626	-0.356363
C	4.157908	-1.599691	2.205118
H	4.884829	-1.907657	2.967290
H	2.749969	0.352554	0.554750

H	-1.663233	-1.633657	1.714127
H	0.746113	0.957135	-0.752288
H	0.343305	-2.250243	3.018444
H	6.115262	0.063931	1.740684
H	4.793253	-2.207070	-2.423527
C	2.170392	-2.669439	-1.463031
H	2.580402	-2.823328	-2.476686
H	1.937496	-1.596449	-1.390161
C	0.869301	-3.461674	-1.320050
H	1.067161	-4.540380	-1.464545
H	0.492164	-3.352164	-0.287696
C	-0.218650	-2.997753	-2.292128
H	0.170704	-3.029677	-3.327311
H	-0.443358	-1.935792	-2.085494
C	-1.515008	-3.805971	-2.212921
H	-1.314507	-4.851389	-2.514008
H	-1.859553	-3.848561	-1.164101
C	-2.646526	-3.232075	-3.067784
H	-2.291852	-3.071422	-4.103958
H	-2.904000	-2.230319	-2.677684
C	-3.904427	-4.105417	-3.088329
H	-4.159275	-4.387601	-2.053239
H	-3.680353	-5.047743	-3.621646
C	-5.109228	-3.415636	-3.728857
H	-4.884264	-3.078537	-4.755776
H	-5.405357	-2.528972	-3.144696
C	8.827517	0.525880	0.581144
H	9.174457	-0.477753	0.295226
H	8.509935	0.451139	1.636623
C	9.994821	1.506401	0.464205
H	10.291895	1.576322	-0.598138
H	9.665730	2.521428	0.756448
C	11.214025	1.100505	1.294008
H	10.937047	1.064232	2.364201
H	11.502857	0.069664	1.020389
C	12.418732	2.025237	1.109884
H	12.690812	2.054640	0.037912
H	12.132730	3.060494	1.374622
C	13.643780	1.612892	1.928671
H	13.374374	1.585090	3.001288
H	13.926041	0.576196	1.664542
C	14.854436	2.530054	1.735835
H	15.121007	2.556300	0.663245
H	14.569688	3.565202	1.999377
C	16.073124	2.107738	2.557451
H	15.844281	2.106207	3.636684
H	16.399739	1.088516	2.289681
C	7.626620	0.915370	-0.297119
H	7.197757	1.858003	0.089353
H	7.984844	1.136088	-1.312034
C	3.235626	-3.018127	-0.406218
H	3.657816	-4.013656	-0.636305
H	2.753065	-3.116471	0.574038
H	16.927512	2.785164	2.397952
H	-5.982579	-4.086084	-3.779964

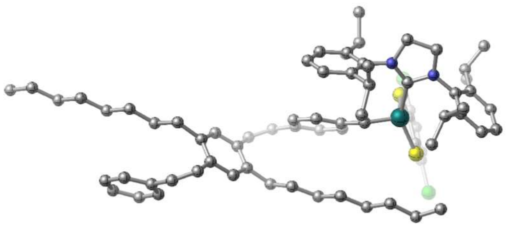

Zero-point correction= 1.447972 (Hartree/Particle)

Thermal correction to Energy= 1.531548

Thermal correction to Enthalpy= 1.532492

Thermal correction to Gibbs Free Energy= 1.317958

Sum of electronic and zero-point Energies= -4715.426141

Sum of electronic and thermal Energies= -4715.342564

Sum of electronic and thermal Enthalpies= -4715.341620

Sum of electronic and thermal Free Energies= -4715.556154

M06L-D3/def2tzvpp-SDD(Ru)-SMD(benzene).

E(scf) = -4719.97946551 a.u.

Int1-α1-ent-M (^{n}Oct)

E(scf) = -5961.95079800 a.u.

ν_{min} = 5.28 cm^{-1}

S	-1.538326	-2.104169	-1.959316	C	1.698064	1.736821	-2.038146
C	-1.355522	0.687962	-2.559680	C	3.007278	1.672434	-1.544086
Ru	-1.784328	-0.194857	-0.669727	C	3.246287	-0.594198	-2.328024
C	-3.232822	1.680352	0.110978	H	3.438917	2.542954	-1.046887
C	-0.057347	0.044122	0.024968	H	3.860935	-1.490599	-2.432763
S	-2.654234	-1.659381	0.987295	C	-6.153323	2.586865	-2.782317
N	-0.165952	0.639510	-3.221720	C	-3.781818	2.909268	-3.224893
N	-2.309676	0.984443	-3.482773	C	-4.730890	0.632014	-3.097487
C	1.160532	0.582351	-2.667047	C	-5.986933	1.204664	-2.842490
C	-0.297525	0.959753	-4.658140	C	-5.061663	3.429728	-2.992841
C	-1.807729	0.913767	-4.865288	C	-2.618441	3.865970	-3.470496
C	-3.627049	1.499904	-3.242768	C	-4.605580	-0.872759	-3.269276
H	-2.190955	1.746915	-5.468927	H	-6.851884	0.552554	-2.704469
H	-2.143006	-0.031011	-5.326046	H	-5.205323	4.511913	-2.965384
H	0.131603	1.954118	-4.861029	H	0.239406	0.226800	-5.271724
C	3.772442	0.513700	-1.670615	H	-1.703462	3.270155	-3.583272
C	1.945025	-0.580566	-2.854288	H	-3.537403	-1.114601	-3.320917

C	1.460999	-1.782285	-3.654697
H	0.383523	-1.662661	-3.826871
C	0.928112	3.050647	-1.939390
H	-0.139810	2.823877	-2.057944
C	-2.117472	-3.478980	-1.020622
C	-2.129928	-4.779945	-1.563727
C	-2.563538	-3.284264	0.310936
C	-2.536868	-5.882807	-0.815176
C	-2.952436	-4.418061	1.057890
C	-2.939894	-5.699631	0.510559
H	-2.541627	-6.876890	-1.261864
H	-3.253307	-6.550010	1.117476
H	-3.582299	1.900198	-0.897483
C	-1.905438	2.002309	0.407524
H	-1.321847	2.502511	-0.364799
H	0.335950	1.065805	0.062320
C	0.908283	-0.857321	0.626563
C	0.785329	-2.262555	0.738739
C	2.061455	-0.257793	1.189070
C	1.771582	-3.016058	1.366883
H	-0.083830	-2.761590	0.323724
C	3.046498	-1.012324	1.810678
C	2.932537	-2.412368	1.896102
H	3.906056	-0.512976	2.252276
H	4.781247	0.471090	-1.256988
H	-7.140156	3.011445	-2.582037
Cl	-1.660048	-5.037292	-3.241886
Cl	-3.471606	-4.240429	2.727735
C	2.168600	-1.839006	-5.022747
H	3.251633	-2.002117	-4.896839
H	1.770438	-2.670439	-5.626832
H	2.043228	-0.907435	-5.597265
C	1.652854	-3.107167	-2.905675
H	1.232427	-3.057095	-1.893389
H	1.140820	-3.924019	-3.435354
H	2.716082	-3.373516	-2.823364
C	1.330958	3.999124	-3.084986
H	2.397517	4.265104	-3.006909
H	1.175885	3.540797	-4.072532
H	0.743603	4.930226	-3.046010
C	1.091157	3.765703	-0.587923
H	2.104499	4.177665	-0.462693
H	0.386003	4.608957	-0.523624
H	0.892166	3.099668	0.264288
C	-2.814822	4.656235	-4.776195
H	-1.931791	5.281896	-4.983767
H	-2.970687	3.987135	-5.637031
H	-3.690441	5.321954	-4.710141
C	-2.382312	4.815392	-2.286917
H	-3.250498	5.466813	-2.109323
H	-2.192615	4.257545	-1.360359
H	-1.512871	5.462413	-2.481252
C	-5.244487	-1.317056	-4.597177
H	-5.093654	-2.398149	-4.749558
H	-6.329714	-1.121569	-4.607048
H	-4.800617	-0.785870	-5.454819
C	-5.180776	-1.658604	-2.081670
H	-4.952245	-2.729819	-2.193288
H	-4.741912	-1.327180	-1.129010
H	-6.275610	-1.553473	-2.011930
C	-4.545498	0.705199	2.106070
C	-4.249169	1.744975	1.227031
C	-4.667418	3.055944	1.596149
C	-4.998767	3.270882	2.935432
C	-4.951882	2.249493	3.902997
C	-4.897868	0.919916	3.450899
C	-4.579927	2.637575	5.306418
H	-5.335882	2.721578	6.096832

C	-1.936412	3.819687	3.638500
C	-2.256574	2.762301	4.509633
C	-1.874039	1.470111	4.114475
C	-1.521391	1.203643	2.790862
C	-1.521887	2.232644	1.836759
C	-1.555874	3.557562	2.318234
C	-3.285646	2.914633	5.591578
H	-3.005411	3.236678	6.601801
H	-1.410066	0.167371	2.470610
H	-2.147841	4.850533	3.935668
H	-5.140873	4.295046	3.284720
H	-4.323786	-0.311183	1.793033
C	3.977528	-3.245966	2.528691
H	3.617958	-3.975953	3.264190
C	5.293136	-3.252963	2.239402
H	5.916899	-3.979274	2.776267
C	5.968789	-2.469134	1.166510
C	6.275122	-3.096461	-0.064079
C	6.292718	-1.118512	1.333428
C	6.839090	-2.319073	-1.078738
C	6.860043	-0.332346	0.318579
H	6.073636	-0.646234	2.294059
C	7.143748	-0.954114	-0.920103
H	7.054363	-2.791897	-2.042183
C	7.679054	-0.223527	-2.099437
H	7.030029	-0.236923	-2.984207
C	8.846375	0.440173	-2.206050
H	9.032604	0.983253	-3.140186
C	9.915587	0.539066	-1.192205
C	10.295500	-0.562547	-0.400545
C	10.597222	1.757748	-1.012475
C	11.307258	-0.440027	0.553723
H	9.791046	-1.520071	-0.539549
C	11.597129	1.885179	-0.045673

H	10.323047	2.619479	-1.627349
C	11.955342	0.786218	0.742671
H	11.589308	-1.306517	1.157274
H	12.098247	2.846358	0.094299
H	12.739589	0.883817	1.497445
H	2.173455	0.826547	1.134190
H	1.653632	-4.100795	1.434105
C	4.488141	-4.680002	-0.896065
H	3.799641	-4.030362	-0.331501
H	4.490414	-4.287868	-1.927969
C	3.950005	-6.110766	-0.882252
H	3.875700	-6.454748	0.166523
H	4.673935	-6.788833	-1.371769
C	2.586567	-6.242429	-1.562524
H	2.687011	-5.962943	-2.626643
H	1.889027	-5.501760	-1.131407
C	1.960619	-7.634960	-1.464222
H	1.756194	-7.870512	-0.402887
H	2.687357	-8.395313	-1.807273
C	0.670620	-7.757237	-2.276130
H	0.900858	-7.588566	-3.344898
H	-0.013255	-6.939663	-1.993906
C	-0.057520	-9.093708	-2.120539
H	0.646454	-9.923736	-2.315365
H	-0.376133	-9.210178	-1.068252
C	-1.269211	-9.223354	-3.046010
H	-0.961549	-9.198242	-4.105048
H	-1.974957	-8.388972	-2.900018
C	8.117998	1.445916	1.674967
H	7.765190	1.056656	2.646774
H	9.046140	0.901900	1.446886
C	8.433860	2.936277	1.803209
H	8.772960	3.311822	0.820516
H	7.511328	3.497861	2.043108

C	9.508728	3.238947	2.848595
H	9.164112	2.895631	3.841891
H	10.408376	2.640754	2.616542
C	9.894349	4.717295	2.925871
H	10.240238	5.053239	1.930114
H	8.995357	5.321513	3.150264
C	10.978420	5.017449	3.963164
H	10.633378	4.683177	4.959669
H	11.875466	4.410034	3.738356
C	11.374521	6.494548	4.035568
H	11.718459	6.826121	3.038584
H	10.476647	7.099308	4.259493
C	12.459221	6.781668	5.074607
H	13.380186	6.215162	4.855328
H	12.128094	6.491189	6.086076
H	-1.463269	4.391023	1.616643
H	-2.027446	0.632513	4.798585
C	-5.279681	5.498965	0.989362
H	-6.337545	5.274919	1.215540
H	-4.854658	5.924330	1.915100
C	-5.204828	6.556191	-0.114835
H	-5.610639	6.135070	-1.053297
H	-4.144765	6.789331	-0.324753
C	-5.947972	7.851019	0.218052
H	-7.008487	7.619072	0.428666
H	-5.542006	8.274392	1.155588
C	-5.869966	8.904471	-0.889233
H	-6.274233	8.478197	-1.826591
H	-4.808601	9.135222	-1.098875
C	-6.613912	10.200947	-0.562139
H	-7.675137	9.969787	-0.352161
H	-6.209520	10.627344	0.375100
C	-6.536780	11.254525	-1.670404
H	-6.940857	10.825857	-2.605872

H	-5.475500	11.483309	-1.878940
C	-7.283625	12.545539	-1.332644
H	-8.354431	12.350324	-1.152899
H	-6.877242	13.013043	-0.419769
C	-5.858976	-1.411047	3.898867
H	-6.895289	-1.044442	3.786550
H	-5.531428	-1.727886	2.894712
C	-5.831151	-2.633233	4.817590
H	-4.781884	-2.957966	4.941032
H	-6.180579	-2.354549	5.829372
C	-6.658212	-3.807562	4.289886
H	-7.726323	-3.522595	4.256037
H	-6.361813	-4.010395	3.245511
C	-6.492941	-5.091082	5.105402
H	-6.778166	-4.902464	6.157508
H	-5.421622	-5.365053	5.126476
C	-7.299403	-6.272314	4.562343
H	-8.371932	-6.002360	4.532329
H	-7.006924	-6.456628	3.511460
C	-7.125634	-7.562842	5.367423
H	-7.418389	-7.377267	6.417221
H	-6.052849	-7.827977	5.397595
C	-7.929436	-8.738563	4.810340
H	-9.009392	-8.513023	4.800063
H	-7.632199	-8.967180	3.772747
C	5.906431	-4.540094	-0.317793
H	6.631013	-4.996551	-1.013029
H	5.968636	-5.116199	0.619130
C	7.076066	1.143689	0.585753
H	6.109876	1.589841	0.886492
H	7.371427	1.652199	-0.342175
C	-4.958871	-0.268437	4.388452
H	-5.284214	0.058468	5.389337
H	-3.936995	-0.670475	4.519469

C	-4.550919	4.206351	0.618989	H	-7.784569	-9.652620	5.408713
H	-4.905500	3.867596	-0.367237	H	-1.816846	-10.164565	-2.877665
H	-3.481739	4.434780	0.468592	H	12.723598	7.851035	5.103766
H	-7.210659	13.283965	-2.147270				

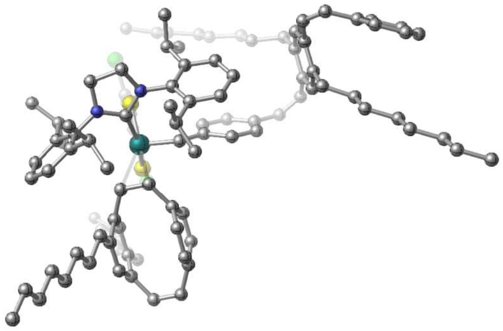

Zero-point correction= 2.131415 (Hartree/Particle)

Thermal correction to Energy= 2.249094

Thermal correction to Enthalpy= 2.250038

Thermal correction to Gibbs Free Energy= 1.960742

Sum of electronic and zero-point Energies= -5959.819383

Sum of electronic and thermal Energies= -5959.701704

Sum of electronic and thermal Enthalpies= -5959.700760

Sum of electronic and thermal Free Energies= -5959.990056

M06L-D3/def2tzvpp-SDD(Ru)-SMD(benzene).

E(scf) = -5966.14681722 a.u.

Int1-β1-M (^{n}Oct)

E(scf) = -5961.96216698 a.u.

ν_{min} = 6.16 cm^{-1}

S	-0.935842	-2.410555	-1.845936	S	-3.243464	-2.102096	0.359849
C	-1.629366	0.311009	-2.817075	N	-0.444692	0.756701	-3.292800
Ru	-2.073942	-0.599680	-0.999340	N	-2.502469	0.251720	-3.851960
C	-4.926651	0.836419	0.111068	C	0.659785	1.279689	-2.538005
C	-0.753058	0.136472	0.060136	C	-0.479310	1.021339	-4.746053

C	-1.864771	0.504257	-5.153803
C	-3.926926	0.098994	-3.728185
H	-2.444001	1.231790	-5.738818
H	-1.809659	-0.433737	-5.728694
H	-0.349324	2.097660	-4.936395
C	2.887680	2.438508	-1.323997
C	1.876632	0.566323	-2.505433
C	0.537844	2.564321	-1.949331
C	1.673514	3.124409	-1.349998
C	2.981051	1.167212	-1.882656
H	1.610105	4.117366	-0.900571
H	3.927880	0.628820	-1.825768
C	-6.684246	-0.152629	-3.408784
C	-4.715798	1.268375	-3.590132
C	-4.511203	-1.186606	-3.791099
C	-5.898348	-1.286964	-3.605579
C	-6.099137	1.113515	-3.425121
C	-4.126460	2.671063	-3.704058
C	-3.695114	-2.422161	-4.138553
H	-6.371473	-2.270290	-3.627631
H	-6.729134	1.997685	-3.307430
H	0.337990	0.487380	-5.249582
H	-3.032567	2.582110	-3.675482
H	-2.638890	-2.187210	-3.953918
C	2.030277	-0.811453	-3.130457
H	1.033495	-1.149539	-3.447875
C	-0.755976	3.368076	-2.002110
H	-1.572086	2.675112	-2.243162
C	-1.523251	-3.822645	-0.957457
C	-1.040827	-5.114423	-1.248103
C	-2.499672	-3.677406	0.061414
C	-1.480420	-6.238555	-0.551456
C	-2.891821	-4.818568	0.790247
C	-2.399554	-6.087817	0.489521
H	-1.104790	-7.225460	-0.815625
H	-2.737417	-6.952633	1.061485
H	-5.217517	0.546347	-0.903281
C	-3.688707	1.356564	0.323146
H	-3.040396	1.534120	-0.556488
H	-0.872016	1.215368	0.269229
C	0.384629	-0.423839	0.778466
C	0.495572	-1.779874	1.160763
C	1.426371	0.450637	1.160786
C	1.609631	-2.233198	1.860462
H	-0.315806	-2.468507	0.941141
C	2.556575	-0.014355	1.823894
C	2.679236	-1.371238	2.179020
H	3.342926	0.687584	2.095468
H	3.762528	2.891645	-0.855819
H	-7.762445	-0.253679	-3.261734
Cl	0.125322	-5.339360	-2.549251
Cl	-4.045020	-4.671267	2.111005
C	2.937575	-0.759545	-4.373149
H	3.962554	-0.459087	-4.098994
H	2.995924	-1.751569	-4.849403
H	2.571991	-0.039991	-5.123179
C	2.560908	-1.833796	-2.115279
H	1.941590	-1.856100	-1.209027
H	2.545305	-2.844887	-2.550966
H	3.596644	-1.605865	-1.819874
C	-0.702102	4.431525	-3.114889
H	0.089234	5.169757	-2.906897
H	-0.491017	3.987709	-4.098949
H	-1.660419	4.970390	-3.185300
C	-1.123577	4.022300	-0.663711
H	-0.401725	4.800228	-0.369956
H	-2.107571	4.504471	-0.748485
H	-1.179900	3.287687	0.153572

C	-4.501087	3.300980	-5.059020
H	-4.009964	4.280272	-5.178789
H	-4.202205	2.661437	-5.904145
H	-5.589851	3.455863	-5.130832
C	-4.532140	3.597801	-2.548795
H	-5.616184	3.792815	-2.541233
H	-4.265899	3.166800	-1.576071
H	-4.025871	4.571196	-2.643402
C	-3.854431	-2.752895	-5.634868
H	-3.216843	-3.609317	-5.908547
H	-4.898719	-3.017381	-5.870274
H	-3.576517	-1.900543	-6.275052
C	-4.037633	-3.639444	-3.269037
H	-3.298695	-4.438378	-3.433530
H	-4.019701	-3.380352	-2.201947
H	-5.031709	-4.049803	-3.510014
C	-5.798278	-0.477063	2.082172
C	-5.860917	0.671153	1.272837
C	-6.526568	1.793458	1.793923
C	-6.805241	1.878229	3.161274
C	-6.424867	0.842024	4.030239
C	-6.083382	-0.390573	3.446890
C	-5.987809	1.141926	5.434997
H	-6.659237	0.983063	6.287438
C	-3.945209	2.930266	3.698529
C	-3.803163	1.770360	4.476367
C	-3.071819	0.687135	3.938615
C	-2.822604	0.710514	2.561639
C	-3.272688	1.740010	1.720757
C	-3.693784	2.951429	2.318187
C	-4.721997	1.572842	5.648468
H	-4.379309	1.748571	6.675616
H	-2.433812	-0.187650	2.091362
H	-4.447883	3.785747	4.152801

H	-7.141616	2.831992	3.576034
H	-5.346891	-1.391510	1.695450
C	3.860239	-1.905358	2.893734
H	3.636102	-2.527538	3.769063
C	5.153442	-1.783172	2.538197
H	5.895055	-2.300222	3.160812
C	5.668592	-1.115410	1.309758
C	6.019996	-1.893469	0.181089
C	5.814237	0.274068	1.243480
C	6.403284	-1.223620	-0.984381
C	6.241524	0.948888	0.089639
H	5.578696	0.864466	2.132923
C	6.522894	0.176916	-1.061318
H	6.636912	-1.814992	-1.875157
C	6.872609	0.775673	-2.378239
H	6.134900	0.605251	-3.173101
C	7.962998	1.495419	-2.703715
H	8.001711	1.919624	-3.714046
C	9.124143	1.801215	-1.844643
C	9.669726	0.848859	-0.961401
C	9.726426	3.072016	-1.912475
C	10.765101	1.167956	-0.156583
H	9.227549	-0.147234	-0.909308
C	10.811443	3.396600	-1.095251
H	9.323183	3.818655	-2.602210
C	11.334296	2.445515	-0.212216
H	11.175189	0.415468	0.521822
H	11.250078	4.396405	-1.146080
H	12.184936	2.697448	0.425915
H	-5.857247	-1.248545	4.085255
H	-6.656756	2.680477	1.168902
C	-5.029448	5.171928	2.033881
H	-4.627425	5.652806	2.942623
H	-5.945945	4.645350	2.349604

C	-5.391002	6.254390	1.014106
H	-4.469283	6.768692	0.683263
H	-5.806120	5.774625	0.107736
C	-6.388388	7.288422	1.539583
H	-7.305866	6.770780	1.876507
H	-5.970311	7.773959	2.440958
C	-6.760404	8.360695	0.513200
H	-7.179321	7.873393	-0.387186
H	-5.841816	8.875731	0.174817
C	-7.757163	9.397178	1.036413
H	-8.675291	8.881949	1.376099
H	-7.337758	9.885345	1.936183
C	-8.130845	10.468881	0.008825
H	-8.549685	9.978663	-0.889157
H	-7.211762	10.981262	-0.330073
C	-9.126409	11.499767	0.542457
H	-10.067711	11.018089	0.857109
H	-8.718873	12.029072	1.420421
C	-1.979461	-1.638613	4.029400
H	-2.476195	-1.886954	3.079368
H	-0.988496	-1.238108	3.750580
C	-1.807734	-2.949119	4.796484
H	-2.805320	-3.387245	4.984998
H	-1.357592	-2.768761	5.790715
C	-0.955929	-3.950025	4.009652
H	0.079636	-3.571518	3.947804
H	-1.321841	-3.985595	2.969182
C	-0.948776	-5.369086	4.574172
H	-0.556748	-5.359026	5.608788
H	-1.990424	-5.733089	4.645481
C	-0.136955	-6.351781	3.726400
H	-0.570140	-6.392566	2.709828
H	0.892334	-5.966384	3.596142
C	-0.075094	-7.764361	4.312629
H	0.409690	-7.722902	5.305281
H	-1.103954	-8.124691	4.496899
C	0.660426	-8.760903	3.417327
H	0.140877	-8.881286	2.454037
H	1.684907	-8.418808	3.192856
H	1.350355	1.509526	0.907399
H	1.662196	-3.284551	2.153423
C	4.553658	-3.977125	-0.064241
H	3.811976	-3.455493	0.560572
H	4.276791	-3.754547	-1.106686
C	4.457427	-5.482733	0.177947
H	4.764505	-5.708756	1.216506
H	5.183989	-6.007271	-0.470919
C	3.056131	-6.041283	-0.069971
H	2.717614	-5.753889	-1.081106
H	2.341989	-5.558278	0.623206
C	2.959846	-7.561062	0.079377
H	3.279375	-7.855388	1.096972
H	3.676885	-8.041852	-0.612445
C	1.554586	-8.097078	-0.189210
H	1.227665	-7.759188	-1.188670
H	0.854521	-7.629581	0.525500
C	1.424698	-9.618177	-0.103586
H	2.120694	-10.084774	-0.824456
H	1.751540	-9.958496	0.895244
C	0.000852	-10.107659	-0.374461
H	-0.337920	-9.805886	-1.380204
H	-0.711633	-9.681023	0.352056
C	7.517318	2.956134	1.030654
H	7.318458	2.678324	2.081347
H	8.444034	2.436259	0.748771
C	7.742905	4.464631	0.928833
H	7.938841	4.721932	-0.128177
H	6.818444	5.004401	1.208266

C	8.909087	4.959962	1.785881	H	6.282851	-3.759513	1.208343
H	8.701920	4.743650	2.850553	H	6.665891	-3.820567	-0.511856
H	9.811558	4.377167	1.528111	C	6.363886	2.457490	0.143210
C	9.209180	6.450439	1.617297	H	5.421217	2.878172	0.535680
H	9.417646	6.658690	0.550902	H	6.487805	2.861381	-0.871596
H	8.306586	7.039282	1.866744	C	-4.018211	4.167499	1.475474
C	10.384827	6.940747	2.465096	H	-4.370028	3.823519	0.490003
H	10.177582	6.733922	3.531912	H	-3.076602	4.706946	1.270507
H	11.285294	6.348279	2.215432	C	-2.783228	-0.560077	4.756355
C	10.696263	8.429559	2.290868	H	-3.747876	-0.986022	5.086426
H	10.902361	8.633586	1.224050	H	-2.267441	-0.269080	5.689627
H	9.795142	9.019300	2.540342	H	-0.076778	-11.204957	-0.310616
C	11.874007	8.906653	3.141715	H	-9.376389	12.257010	-0.218014
H	12.796239	8.356473	2.888771	H	12.074645	9.980285	2.995598
H	11.679888	8.745690	4.215732	H	0.733497	-9.756813	3.883309
C	5.951127	-3.404370	0.218131				

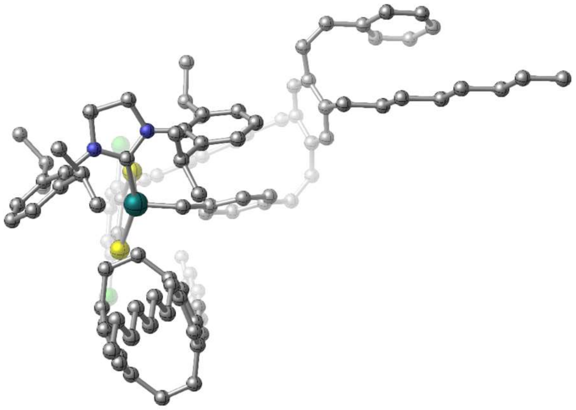

Zero-point correction= 2.130532 (Hartree/Particle)

Thermal correction to Energy= 2.248623

Thermal correction to Enthalpy= 2.249567

Thermal correction to Gibbs Free Energy= 1.961666

Sum of electronic and zero-point Energies= -5959.831635

Sum of electronic and thermal Energies= -5959.713544

Sum of electronic and thermal Enthalpies= -5959.712600

Sum of electronic and thermal Free Energies= -5960.000501

M06L-D3/def2tzvpp-SDD(Ru)-SMD(benzene).

E(scf) = -5966.15525706 a.u.

TS2-α1 (^{n}Oct)

E(scf) = -5961.93692686 a.u.

ν_{min} = -95.92 cm^{-1}

S	1.841709	-1.827281	1.873317	C	5.002177	3.816411	2.780471
C	1.430905	0.899268	2.441686	C	2.591145	4.077389	3.497025
Ru	1.860623	0.026582	0.520117	C	4.852054	-0.505712	3.011061
C	2.848660	1.856225	-0.084414	H	6.945534	1.070014	2.294989
C	0.163070	0.523423	-0.231360	H	5.080092	4.905994	2.795973
S	2.758639	-1.489444	-1.130774	H	0.031298	0.206249	5.209296
N	0.288965	0.773120	3.173059	H	1.721819	3.423743	3.641911
N	2.428770	1.191660	3.322045	H	3.810239	-0.824639	3.130188
C	-1.068135	0.723596	2.697507	C	-1.236734	-1.721569	3.481687
C	0.497972	1.003902	4.617759	H	-0.166566	-1.553082	3.655561
C	2.019167	1.013805	4.725864	C	-0.947124	3.251248	2.166721
C	3.704000	1.792263	3.039072	H	0.131767	3.050262	2.206103
H	2.406854	1.822994	5.358324	C	2.544510	-3.194784	1.010873
H	2.420307	0.057562	5.102234	C	2.777076	-4.425503	1.653544
H	0.040247	1.961041	4.914518	C	2.895341	-3.049217	-0.352990
C	-3.715620	0.640991	1.798551	C	3.352274	-5.501897	0.982969
C	-1.801491	-0.478813	2.809448	C	3.407990	-4.177696	-1.034094
C	-1.674841	1.909554	2.204563	C	3.652753	-5.380401	-0.377031
C	-3.004176	1.840178	1.767333	H	3.540804	-6.436222	1.510189
C	-3.119640	-0.500872	2.324864	H	4.069268	-6.223981	-0.928839
H	-3.489267	2.736045	1.375782	H	2.818590	2.522848	0.776533
H	-3.685963	-1.433661	2.360909	C	1.566260	1.908210	-0.854557
C	6.125467	3.051852	2.463986	H	0.892719	2.662759	-0.440068
C	3.775832	3.208101	3.078104	H	-0.412352	1.236829	0.358239
C	4.851740	1.002401	2.813680	C	-0.719800	-0.022487	-1.273215
C	6.050514	1.661518	2.498932	C	-0.458536	-1.132977	-2.103666

C	-1.972528	0.618500	-1.417267
C	-1.396704	-1.564169	-3.038514
H	0.485920	-1.665495	-1.994768
C	-2.917274	0.172272	-2.330241
C	-2.651030	-0.936025	-3.155125
H	-3.878801	0.680703	-2.407819
H	-4.735166	0.598394	1.409894
H	7.068308	3.543588	2.212135
Cl	2.329436	-4.644602	3.342764
Cl	3.749364	-4.091462	-2.755845
C	-1.912456	-1.954022	4.845981
H	-2.988362	-2.161310	4.723784
H	-1.458164	-2.819073	5.355751
H	-1.819230	-1.078283	5.508123
C	-1.351356	-2.968739	2.597502
H	-0.963364	-2.770600	1.590805
H	-0.761621	-3.794989	3.022869
H	-2.394304	-3.309750	2.503167
C	-1.317125	4.097520	3.399893
H	-2.396463	4.319767	3.402020
H	-1.084567	3.577637	4.340523
H	-0.772844	5.054573	3.396636
C	-1.205319	4.058115	0.883028
H	-2.232967	4.451520	0.844534
H	-0.523817	4.921441	0.839179
H	-1.048028	3.456455	-0.024342
C	2.872475	4.767501	4.844208
H	1.981798	5.317028	5.189615
H	3.150636	4.040400	5.623210
H	3.700151	5.489456	4.754760
C	2.189392	5.108761	2.434202
H	3.006403	5.811438	2.217060
H	1.906990	4.626169	1.489022
H	1.329454	5.701000	2.782407
C	5.596276	-0.868520	4.309264
H	5.527519	-1.952510	4.496908
H	6.664386	-0.601438	4.249008
H	5.168559	-0.343057	5.178586
C	5.417730	-1.278630	1.811757
H	5.329931	-2.362478	1.984256
H	4.862971	-1.047977	0.892753
H	6.481321	-1.051278	1.636103
C	4.835273	0.976790	-1.455197
C	4.115416	2.032023	-0.892001
C	4.431509	3.345924	-1.337357
C	5.144996	3.461642	-2.532279
C	5.565923	2.349800	-3.281217
C	5.567344	1.091935	-2.648583
C	5.618459	2.516443	-4.771971
H	6.571986	2.575246	-5.311277
C	2.470983	3.490588	-4.100677
C	3.161256	2.431428	-4.717597
C	2.808138	1.132066	-4.321525
C	2.077250	0.914781	-3.155906
C	1.672306	1.987320	-2.352552
C	1.723257	3.269628	-2.937249
C	4.455072	2.613887	-5.458258
H	4.470342	2.774167	-6.543131
H	1.960634	-0.105173	-2.805993
H	2.651346	4.518950	-4.425228
H	5.236031	4.442287	-3.000086
H	4.682757	-0.011393	-1.037911
C	-3.682895	-1.431058	-4.091628
H	-3.373418	-1.604372	-5.128879
C	-4.952277	-1.701030	-3.735360
H	-5.655920	-2.021334	-4.513732
C	-5.474998	-1.628271	-2.341012
C	-5.042745	-2.521573	-1.330739

C	-6.406590	-0.631918	-2.014583
C	-5.566176	-2.345136	-0.044001
C	-6.926192	-0.452181	-0.725456
H	-6.714385	0.063358	-2.801512
C	-6.501455	-1.349361	0.284770
H	-5.251201	-3.029793	0.748080
C	-6.955877	-1.269405	1.698936
H	-6.161504	-1.108307	2.438299
C	-8.211826	-1.380480	2.173463
H	-8.358221	-1.222925	3.248629
C	-9.436892	-1.681636	1.406050
C	-9.449031	-2.631294	0.365473
C	-10.640630	-1.025183	1.725677
C	-10.621507	-2.896164	-0.344708
H	-8.529562	-3.163433	0.116444
C	-11.809329	-1.277173	1.003615
H	-10.651403	-0.294339	2.539048
C	-11.803892	-2.213403	-0.036076
H	-10.611649	-3.637769	-1.147556
H	-12.728105	-0.739953	1.252256
H	-12.718384	-2.413683	-0.599867
H	-2.209473	1.474915	-0.785421
H	-1.169139	-2.430841	-3.664676
C	-2.909642	-3.805612	-0.635405
H	-2.349033	-2.856453	-0.600632
H	-3.283538	-3.977219	0.387551
C	-1.976732	-4.959657	-1.021186
H	-1.408086	-4.687267	-1.929431
H	-2.592499	-5.834150	-1.304244
C	-1.017971	-5.388024	0.091597
H	-1.613721	-5.603115	0.997744
H	-0.352726	-4.551683	0.365132
C	-0.164462	-6.615976	-0.245758
H	0.659154	-6.319918	-0.920754
H	-0.770936	-7.350808	-0.807813
C	0.409624	-7.294536	1.000233
H	-0.424593	-7.686535	1.612531
H	0.907157	-6.538485	1.630135
C	1.391233	-8.429080	0.703446
H	0.889427	-9.198568	0.088465
H	2.214495	-8.037516	0.079958
C	1.962712	-9.070302	1.968987
H	1.163214	-9.515802	2.584892
H	2.473840	-8.320741	2.597220
C	-9.209092	0.596112	-1.239010
H	-9.021251	0.659570	-2.325777
H	-9.648989	-0.396350	-1.063257
C	-10.220767	1.666882	-0.829893
H	-10.389362	1.596263	0.260213
H	-9.798289	2.673572	-1.008991
C	-11.564059	1.535416	-1.549812
H	-11.409782	1.638784	-2.640151
H	-11.951716	0.512428	-1.394292
C	-12.614173	2.546339	-1.085650
H	-12.765227	2.435804	0.004744
H	-12.228194	3.572599	-1.232054
C	-13.961301	2.405190	-1.797442
H	-13.811983	2.516457	-2.887935
H	-14.343974	1.377329	-1.651696
C	-15.018326	3.408276	-1.327906
H	-15.165290	3.295341	-0.238029
H	-14.633525	4.434275	-1.473880
C	-16.360207	3.256192	-2.045444
H	-16.783836	2.249759	-1.887911
H	-16.247805	3.398379	-3.133679
H	1.327313	4.132038	-2.393861
H	3.249832	0.273023	-4.832422
C	4.214340	5.930125	-1.245327

H	5.310589	6.031348	-1.332306
H	3.818706	5.999130	-2.274262
C	3.668558	7.097803	-0.420432
H	4.108149	7.064763	0.593295
H	2.579281	6.970661	-0.277551
C	3.941127	8.469505	-1.039982
H	5.030041	8.592909	-1.188489
H	3.494853	8.508483	-2.051124
C	3.408037	9.636329	-0.205642
H	3.855390	9.595773	0.805260
H	2.319279	9.509841	-0.056171
C	3.677029	11.010698	-0.822138
H	4.765814	11.136926	-0.971694
H	3.229538	11.051379	-1.833017
C	3.143773	12.177871	0.013057
H	3.591607	12.134453	1.022855
H	2.055844	12.049253	0.161425
C	3.418522	13.546194	-0.612067
H	4.501188	13.714895	-0.740219
H	2.952987	13.628974	-1.608802
C	6.479822	-1.288353	-2.365604
H	7.225924	-0.931199	-1.632350
H	5.586024	-1.562400	-1.784959
C	6.979767	-2.562843	-3.044830
H	6.228322	-2.884404	-3.787539
H	7.909782	-2.367189	-3.610407
C	7.200127	-3.697390	-2.039119
H	8.056965	-3.451448	-1.384323
H	6.320135	-3.752068	-1.374013

C	7.410150	-5.073216	-2.671800
H	8.302467	-5.057292	-3.325428
H	6.550332	-5.293164	-3.330363
C	7.545892	-6.196427	-1.641327
H	8.413256	-5.993492	-0.985187
H	6.660422	-6.182200	-0.978292
C	7.689806	-7.590815	-2.255749
H	8.577397	-7.608546	-2.914551
H	6.823432	-7.785372	-2.914371
C	7.800468	-8.703491	-1.212400
H	8.677217	-8.551878	-0.560052
H	6.908955	-8.728226	-0.562722
C	-4.095871	-3.675013	-1.601546
H	-4.679428	-4.613991	-1.556431
H	-3.714523	-3.613745	-2.629100
C	-7.878216	0.700427	-0.475717
H	-7.377233	1.642276	-0.765444
H	-8.088641	0.784207	0.599373
C	6.119077	-0.149837	-3.324910
H	6.996649	0.122623	-3.936294
H	5.365058	-0.521074	-4.045218
C	3.861102	4.567865	-0.649429
H	4.179153	4.542466	0.404472
H	2.762186	4.484651	-0.617192
H	3.023942	14.365514	0.010217
H	7.900063	-9.695839	-1.681273
H	2.688719	-9.865809	1.735556
H	-17.100080	3.990038	-1.687190

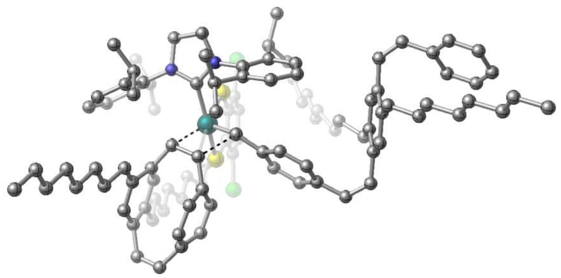

Zero-point correction= 2.132400 (Hartree/Particle)

Thermal correction to Energy= 2.248808

Thermal correction to Enthalpy= 2.249752

Thermal correction to Gibbs Free Energy= 1.965772

Sum of electronic and zero-point Energies= -5959.804527

Sum of electronic and thermal Energies= -5959.688119

Sum of electronic and thermal Enthalpies= -5959.687175

Sum of electronic and thermal Free Energies= -5959.971155

M06L-D3/def2tzvpp-SDD(Ru)-SMD(benzene).

E(scf) = -5966.13658906 a.u.

TS2-β1 (^{n}Oct)

E(scf) = -5961.94209708 a.u.

ν_{min} = -189.03 cm^{-1}

S	-2.532959	-1.793758	-2.077782	C	0.935183	1.891566	-2.573555
C	-2.161077	0.957914	-2.685454	C	2.279715	1.815742	-2.189415
Ru	-2.520540	0.070439	-0.754131	C	2.371544	-0.516893	-2.783417
C	-3.434020	1.889654	-0.085175	H	2.781445	2.700639	-1.796769
C	-0.777674	0.521696	-0.076182	H	2.932958	-1.450788	-2.850001
S	-3.459715	-1.383958	0.921090	C	-6.895577	2.997280	-2.326708
N	-1.067080	0.785223	-3.478389	C	-4.561932	3.293076	-2.949915
N	-3.180972	1.344432	-3.495820	C	-5.577380	1.044484	-2.953634
C	0.308400	0.712564	-3.063822	C	-6.787809	1.626347	-2.543895
C	-1.372675	0.883994	-4.920689	C	-5.793584	3.823529	-2.546424
C	-2.827118	1.363305	-4.926094	C	-3.401548	4.242655	-3.239987
C	-4.456590	1.887547	-3.108693	C	-5.534363	-0.439450	-3.282920
H	-2.948119	2.376338	-5.339550	H	-7.663253	0.989547	-2.400810
H	-3.495377	0.689541	-5.482715	H	-5.892892	4.901546	-2.400665
H	-0.680571	1.577833	-5.418120	H	-1.260598	-0.101304	-5.395965
C	2.991053	0.618131	-2.273466	H	-2.518870	3.636300	-3.476023
C	1.031113	-0.492002	-3.205468	H	-4.485958	-0.714075	-3.449998

C	0.434482	-1.744885	-3.830171
H	-0.647477	-1.593476	-3.924976
C	0.187205	3.219379	-2.457088
H	-0.863979	2.987878	-2.230591
C	-3.213831	-3.146741	-1.177278
C	-3.407148	-4.401382	-1.786988
C	-3.618177	-2.957721	0.166719
C	-4.008169	-5.457666	-1.108589
C	-4.204441	-4.052401	0.843664
C	-4.402731	-5.281260	0.220449
H	-4.164412	-6.411256	-1.610906
H	-4.869314	-6.098441	0.771951
H	-3.496343	2.549409	-0.946943
C	-2.061233	1.954422	0.534307
H	-1.441229	2.669253	-0.005813
H	-0.180832	1.193508	-0.691402
C	0.109612	-0.069131	0.939182
C	-0.156869	-1.208168	1.725732
C	1.364584	0.560388	1.103064
C	0.774966	-1.673869	2.650629
H	-1.105247	-1.729556	1.599793
C	2.302540	0.082247	2.007046
C	2.027770	-1.050400	2.795575
H	3.266683	0.582358	2.105139
H	4.027450	0.572445	-1.932144
H	-7.844848	3.427411	-1.998074
Cl	-2.893971	-4.670107	-3.450286
Cl	-4.755352	-3.874897	2.501923
C	1.011626	-1.975439	-5.238809
H	2.097403	-2.161859	-5.195050
H	0.537085	-2.851992	-5.708996
H	0.851458	-1.105288	-5.895324
C	0.625059	-2.985115	-2.947541
H	0.286447	-2.789034	-1.922027

H	0.030409	-3.824864	-3.337291
H	1.678253	-3.304852	-2.906704
C	0.216245	3.991022	-3.789017
H	1.255007	4.220759	-4.075470
H	-0.237757	3.418204	-4.608793
H	-0.331700	4.942142	-3.700603
C	0.696325	4.117742	-1.321026
H	1.681605	4.552928	-1.549597
H	-0.000363	4.953805	-1.161128
H	0.785261	3.567521	-0.372763
C	-3.703746	5.112744	-4.473525
H	-2.827416	5.726639	-4.737650
H	-3.968350	4.497811	-5.347918
H	-4.547543	5.794500	-4.280323
C	-3.023502	5.121745	-2.037493
H	-3.862302	5.757380	-1.713695
H	-2.714307	4.513704	-1.176984
H	-2.184214	5.786762	-2.294388
C	-6.303042	-0.724675	-4.585865
H	-6.215511	-1.789797	-4.855552
H	-7.374998	-0.489792	-4.479156
H	-5.912848	-0.126292	-5.425189
C	-6.047790	-1.317078	-2.131615
H	-5.888779	-2.381045	-2.365316
H	-5.512580	-1.099967	-1.195994
H	-7.125253	-1.169845	-1.954419
C	-5.284725	1.187514	1.608518
C	-4.596912	2.141158	0.844667
C	-4.803386	3.492371	1.198183
C	-5.325391	3.830323	2.447715
C	-5.695699	2.827736	3.361261
C	-5.832391	1.526892	2.849728
C	-5.563733	3.051571	4.839948
H	-6.436753	3.292794	5.458753

C	-2.546547	3.633864	3.823004	H	9.859064	-0.498172	-3.088891
C	-3.164657	2.594874	4.547210	C	11.045988	-2.470496	-0.570297
C	-2.909054	1.270163	4.151046	H	9.857329	-3.883337	0.559592
C	-2.350957	1.079672	2.878932	H	11.962622	-1.001023	-1.868552
C	-2.011651	2.124024	2.027131	H	11.972144	-2.695989	-0.035822
C	-1.956401	3.436741	2.570434	H	-6.212887	0.729623	3.493574
C	-4.345358	2.926178	5.416103	H	-4.387188	4.283710	0.570387
H	-4.240525	3.068131	6.498743	C	-1.506885	5.993024	2.348209
H	-2.333079	0.069190	2.484154	H	-0.936190	6.013294	3.293467
H	-2.683987	4.653106	4.187316	H	-2.548836	6.245791	2.612886
H	-5.282314	4.872122	2.776406	C	-0.959495	7.060085	1.396374
H	-5.257026	0.141093	1.311785	H	0.084880	6.811909	1.130336
C	3.059720	-1.578453	3.715408	H	-1.525523	7.024147	0.446354
H	2.763113	-1.754003	4.756052	C	-1.014300	8.479838	1.962844
C	4.315230	-1.873472	3.331631	H	-2.059210	8.726394	2.228254
H	5.028215	-2.219099	4.090375	H	-0.447605	8.516161	2.911729
C	4.804450	-1.794735	1.924974	C	-0.470394	9.543802	1.006840
C	4.325146	-2.666366	0.916535	H	-1.037146	9.504715	0.057608
C	5.743409	-0.810999	1.583183	H	0.574565	9.295914	0.741959
C	4.809519	-2.480519	-0.383924	C	-0.525254	10.965602	1.569759
C	6.226844	-0.623616	0.280981	H	-1.570349	11.213359	1.834701
H	6.087079	-0.131362	2.368933	H	0.041248	11.004415	2.519124
C	5.753750	-1.497877	-0.727576	C	0.018892	12.030461	0.613646
H	4.455203	-3.145926	-1.175678	H	-0.548228	11.989279	-0.334425
C	6.169590	-1.409206	-2.152999	H	1.062939	11.780385	0.349981
H	5.358787	-1.225303	-2.868556	C	-0.040779	13.447077	1.186553
C	7.409229	-1.539053	-2.664426	H	-1.078009	13.735514	1.427438
H	7.528021	-1.371701	-3.741517	H	0.546326	13.524863	2.117305
C	8.649503	-1.873573	-1.936287	C	-2.332431	-1.063300	4.978135
C	8.672284	-2.835564	-0.907257	H	-1.859072	-1.148835	3.989828
C	9.856796	-1.238117	-2.283715	H	-1.520891	-0.758643	5.662253
C	9.859190	-3.132649	-0.234830	C	-2.861943	-2.455989	5.348861
H	7.749590	-3.351663	-0.637290	H	-3.888934	-2.567486	4.959337
C	11.040555	-1.522349	-1.599113	H	-2.935135	-2.576962	6.444892

C	-2.004087	-3.568907	4.737724
H	-0.954761	-3.463186	5.071727
H	-1.992671	-3.413811	3.644003
C	-2.493441	-4.991094	5.018140
H	-2.301788	-5.257858	6.074133
H	-3.589274	-5.028229	4.887526
C	-1.858476	-6.030363	4.091070
H	-2.090522	-5.757032	3.045076
H	-0.756063	-5.982477	4.175729
C	-2.327740	-7.464046	4.348047
H	-2.080262	-7.748423	5.387366
H	-3.430896	-7.496493	4.282091
C	-1.727999	-8.481088	3.376022
H	-2.007324	-8.249147	2.335972
H	-0.625746	-8.477087	3.424912
H	1.605818	1.435297	0.497285
H	0.537230	-2.557196	3.247783
C	2.170489	-3.937344	0.253725
H	1.644523	-2.971128	0.178205
H	2.527769	-4.171841	-0.762983
C	1.200038	-5.035004	0.706169
H	0.617019	-4.678023	1.575176
H	1.785175	-5.898456	1.074828
C	0.255932	-5.533253	-0.389474
H	0.866667	-5.871046	-1.247544
H	-0.360933	-4.702230	-0.771924
C	-0.659583	-6.677500	0.058174
H	-1.476955	-6.270873	0.680152
H	-0.099033	-7.369323	0.713578
C	-1.249214	-7.468107	-1.111140
H	-0.426945	-7.954702	-1.669267
H	-1.715512	-6.769151	-1.825627
C	-2.269258	-8.529278	-0.694141
H	-1.785568	-9.255202	-0.015810
H	-3.064748	-8.049194	-0.096745
C	-2.890007	-9.266312	-1.881560
H	-2.118473	-9.785687	-2.474778
H	-3.402588	-8.564169	-2.561486
C	8.543059	0.376146	0.739740
H	8.388715	0.432459	1.832160
H	8.957519	-0.623065	0.541772
C	9.563942	1.430279	0.310727
H	9.697956	1.367581	-0.784650
H	9.168307	2.443560	0.512540
C	10.925435	1.263263	0.987838
H	10.807088	1.360305	2.083236
H	11.285305	0.233460	0.812023
C	11.983145	2.254712	0.499568
H	12.096904	2.151381	-0.596053
H	11.625766	3.288008	0.667499
C	13.348651	2.075831	1.166456
H	13.236876	2.179820	2.262146
H	13.702408	1.040911	0.999185
C	14.413272	3.058797	0.672193
H	14.522181	2.953545	-0.422911
H	14.057738	4.091946	0.840275
C	15.773736	2.868178	1.344098
H	16.168162	1.853828	1.163243
H	15.700045	3.001732	2.436727
C	3.366868	-3.805123	1.206586
H	2.993377	-3.725150	2.235486
H	3.938611	-4.752009	1.173179
C	7.192650	0.514368	0.017212
H	6.718002	1.461609	0.332635
H	7.373216	0.607709	-1.062514
C	-1.455449	4.596126	1.733079
H	-2.029407	4.627145	0.792046
H	-0.416845	4.383949	1.425537

C	-3.395733	0.046448	4.902630	H	0.356872	14.190853	0.477416
H	-4.275077	-0.356425	4.368188	H	16.518735	3.588241	0.968891
H	-3.749445	0.323037	5.908375	H	-2.069214	-9.505982	3.594701
H	-3.627272	-10.018825	-1.558243				

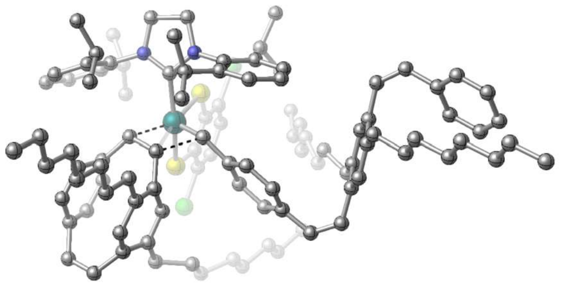

Zero-point correction= 2.132594 (Hartree/Particle)

Thermal correction to Energy= 2.249011

Thermal correction to Enthalpy= 2.249956

Thermal correction to Gibbs Free Energy= 1.967108

Sum of electronic and zero-point Energies= -5959.809503

Sum of electronic and thermal Energies= -5959.693086

Sum of electronic and thermal Enthalpies= -5959.692141

Sum of electronic and thermal Free Energies= -5959.974989

M06L-D3/def2tzvpp-SDD(Ru)-SMD(benzene).

E(scf) = -5966.14382996 a.u.

Int2-α1 (^{n}Oct)

E(scf) = -5962.02716578 a.u.

ν_{min} = 7.68 cm^{-1}

S	-2.648411	-0.033510	0.908496	N	-0.509048	-0.704977	3.857949
C	-1.413751	-1.442711	3.178518	N	-2.496641	-1.619683	3.966698
Ru	-1.138178	-1.712530	1.132357	C	0.846858	-0.490511	3.426016
C	-1.978731	-3.317206	0.764030	C	-0.954259	-0.351868	5.215551
C	4.112770	-2.768648	-3.130399	C	-2.422738	-0.806051	5.199636
S	-0.393579	-1.222357	-1.057872	C	-3.570416	-2.537617	3.709550

H	-2.711325	-1.394398	6.081028	H	-1.562429	-4.119291	1.400064
H	-3.108267	0.050809	5.122525	C	3.540940	-3.911893	-3.567304
H	-0.344584	-0.884059	5.965473	H	4.158671	-4.817647	-3.540623
C	3.477797	-0.110645	2.583582	H	5.122842	-2.843179	-2.709030
C	1.182559	0.692446	2.731544	C	3.542226	-1.398888	-3.211681
C	1.817197	-1.474681	3.730373	C	3.277115	-0.833873	-4.472112
C	3.130896	-1.263011	3.290947	C	3.257949	-0.619908	-2.074159
C	2.513439	0.859280	2.314921	C	2.754848	0.449967	-4.584093
H	3.894784	-2.013681	3.505204	H	3.480577	-1.420401	-5.371049
H	2.794781	1.759488	1.764261	C	2.702310	0.654762	-2.187766
C	-5.638980	-4.353350	3.254730	C	2.452117	1.231921	-3.448796
C	-3.310731	-3.929268	3.833465	H	2.426826	1.190666	-1.280088
C	-4.852052	-2.051464	3.371611	H	4.505106	0.032164	2.238344
C	-5.876924	-2.986750	3.154455	H	-6.447694	-5.065371	3.071652
C	-4.365631	-4.817264	3.589644	Cl	-4.125384	2.658160	0.099069
C	-1.937271	-4.455301	4.250249	Cl	-0.524312	-0.212422	-4.159291
C	-5.169800	-0.564746	3.265073	C	0.356469	3.007678	3.299466
H	-6.876456	-2.634139	2.893564	H	1.343971	3.458479	3.117872
H	-4.200198	-5.891965	3.665297	H	-0.406842	3.769396	3.071717
H	-0.844126	0.727939	5.389067	H	0.289260	2.762657	4.371923
H	-1.184869	-3.856460	3.716746	C	0.183205	2.126864	0.927168
H	-4.216411	-0.022605	3.194910	H	0.119302	1.228083	0.302727
C	0.153278	1.764997	2.416754	H	-0.663588	2.778385	0.669231
H	-0.840878	1.352708	2.635222	H	1.106151	2.656530	0.663985
C	1.477536	-2.733705	4.518598	C	2.263167	-2.806304	5.838338
H	0.413417	-2.692980	4.781127	H	3.346303	-2.898833	5.658803
C	-2.361528	0.698307	-0.675352	H	2.100395	-1.905078	6.450552
C	-3.053364	1.862283	-1.053608	H	1.945627	-3.682542	6.426570
C	-1.461179	0.092220	-1.588773	C	1.675180	-4.002707	3.677661
C	-2.937135	2.405375	-2.330906	H	2.725750	-4.129728	3.373052
C	-1.433360	0.603955	-2.904228	H	1.380756	-4.896185	4.249811
C	-2.139011	1.751786	-3.269583	H	1.064670	-3.966811	2.763976
H	-3.494820	3.301863	-2.600865	C	-1.694720	-4.270937	5.760122
H	-2.066872	2.125330	-4.291403	H	-0.692207	-4.634279	6.037003

H	-1.763350	-3.220870	6.070064
H	-2.436398	-4.841722	6.341775
C	-1.687044	-5.920583	3.873863
H	-2.304291	-6.609369	4.472035
H	-1.897882	-6.116136	2.813460
H	-0.634210	-6.180185	4.060933
C	-5.913442	-0.077476	4.523361
H	-6.090027	1.009289	4.472859
H	-6.892834	-0.575743	4.612466
H	-5.351029	-0.291681	5.445481
C	-5.971696	-0.217449	1.999535
H	-6.021405	0.874935	1.872845
H	-5.493371	-0.630622	1.100619
H	-7.005585	-0.594627	2.054690
C	-3.342874	-2.867279	-1.274283
C	-2.639816	-3.776015	-0.444325
C	-2.329681	-5.065717	-0.967416
C	-2.572833	-5.290286	-2.323486
C	-3.127772	-4.324642	-3.173493
C	-3.609391	-3.110714	-2.619674
C	-3.079132	-4.577224	-4.633629
H	-4.029270	-4.641210	-5.177763
C	0.518147	-5.310602	-5.398103
C	-0.547621	-4.564665	-4.852758
C	-0.224685	-3.591522	-3.885903
C	1.088014	-3.364542	-3.495423
C	2.153398	-4.114220	-4.028815
C	1.835070	-5.109176	-4.976224
C	-1.934104	-4.757791	-5.330650
H	-2.026765	-5.045320	-6.384969
H	1.276072	-2.591427	-2.753487
H	0.308064	-6.069912	-6.157167
H	-2.252161	-6.238090	-2.764049
H	-3.600673	-1.905057	-0.838108
C	1.807996	2.534960	-3.684563
H	1.366235	2.596994	-4.685638
C	1.623311	3.646320	-2.930041
H	1.010267	4.414608	-3.412664
C	2.116275	4.023973	-1.594183
C	1.324793	4.841785	-0.745775
C	3.379054	3.607678	-1.143093
C	1.863406	5.214775	0.488238
C	3.900848	3.936105	0.111910
H	3.967829	2.984296	-1.812753
C	3.133672	4.802019	0.932882
H	1.272619	5.836333	1.163308
C	3.566865	5.253756	2.276270
H	2.837013	5.076883	3.076019
C	4.708736	5.873142	2.644737
H	4.840360	6.057753	3.717469
C	5.815879	6.353416	1.799748
C	5.622870	6.801661	0.476600
C	7.120472	6.391135	2.330561
C	6.701528	7.237169	-0.293643
H	4.617524	6.806859	0.053196
C	8.201440	6.820158	1.556825
H	7.286768	6.064442	3.361018
C	7.996882	7.241166	0.238648
H	6.530243	7.579847	-1.317435
H	9.206986	6.828844	1.985485
H	8.840256	7.579883	-0.368405
H	3.427055	-1.032206	-1.077812
H	2.547270	0.855042	-5.578279
C	-1.010591	5.741506	-0.046854
H	-1.000376	5.036856	0.802971
H	-0.633272	6.701175	0.347870
C	-2.452831	5.916054	-0.530299
H	-2.763043	4.998037	-1.056750

H	-2.500644	6.727648	-1.279861	C	11.342453	-2.983100	-1.184309
C	-3.453267	6.183125	0.594517	H	11.634456	-2.555538	-2.158449
H	-3.172583	7.102405	1.141775	H	10.611006	-3.784687	-1.383551
H	-3.383861	5.357805	1.326263	H	2.635644	-5.720998	-5.402265
C	-4.900435	6.290681	0.108531	H	-1.001400	-2.976405	-3.437532
H	-5.135548	5.404402	-0.507074	C	-0.108730	-6.257162	-0.517588
H	-5.002018	7.162920	-0.563734	H	0.300733	-7.131838	0.019482
C	-5.923205	6.393227	1.241659	H	0.022503	-6.465774	-1.594227
H	-5.696099	7.277803	1.866314	C	0.700788	-5.014408	-0.148158
H	-5.808030	5.517979	1.908740	H	0.494811	-4.748014	0.902104
C	-7.375350	6.468873	0.763058	H	0.363283	-4.156028	-0.752575
H	-7.495516	7.346291	0.102165	C	2.207974	-5.197249	-0.309775
H	-7.593332	5.589501	0.130753	H	2.537239	-6.040042	0.327227
C	-8.387327	6.544125	1.906820	H	2.437648	-5.491784	-1.349606
H	-8.212929	7.433733	2.535713	C	3.013017	-3.947078	0.048921
H	-8.313483	5.659386	2.561889	H	2.651654	-3.533299	1.006161
C	5.709616	2.116216	-0.186888	H	2.820936	-3.168753	-0.706917
H	4.924076	1.340022	-0.202452	C	4.513930	-4.215811	0.157237
H	5.889580	2.378529	-1.243563	H	4.679533	-4.998795	0.921717
C	7.000769	1.530414	0.386945	H	4.879044	-4.643670	-0.794355
H	7.751724	2.335392	0.491962	C	5.354363	-2.989653	0.517723
H	6.817110	1.156763	1.412162	H	4.920799	-2.503271	1.408192
C	7.582244	0.407255	-0.473856	H	5.282068	-2.241999	-0.291549
H	6.836461	-0.402964	-0.570553	C	6.821580	-3.327392	0.779570
H	7.743733	0.786878	-1.499720	H	6.919694	-4.047740	1.609296
C	8.894005	-0.171017	0.058909	H	7.290453	-3.784162	-0.108096
H	9.626836	0.645638	0.196736	C	-4.975342	-0.928983	-2.810692
H	8.725410	-0.590589	1.068350	H	-5.824814	-1.356723	-2.247512
C	9.498640	-1.246245	-0.846462	H	-4.322794	-0.453900	-2.063739
H	8.737849	-2.020981	-1.049710	C	-5.465830	0.169162	-3.755300
H	9.741214	-0.799876	-1.828881	H	-4.589667	0.584715	-4.284192
C	10.748598	-1.913040	-0.267462	H	-6.122120	-0.256196	-4.537049
H	11.509335	-1.139479	-0.055385	C	-6.187358	1.302970	-3.019671
H	10.495751	-2.362380	0.710505	H	-7.172866	0.951006	-2.662739

H	-5.617927	1.555666	-2.110282
C	-6.361301	2.574352	-3.852451
H	-7.017891	2.372228	-4.719250
H	-5.380819	2.855623	-4.280222
C	-6.907016	3.760708	-3.054419
H	-7.889169	3.496161	-2.619626
H	-6.240819	3.948969	-2.191756
C	-7.043541	5.044306	-3.877503
H	-7.730932	4.861243	-4.723718
H	-6.065149	5.284501	-4.333221
C	-7.532337	6.241418	-3.061825
H	-8.513355	6.037153	-2.600452
H	-6.831575	6.474863	-2.244725
H	12.237465	-3.449502	-0.741887
C	-1.615580	-6.159854	-0.203541
C	-4.211969	-2.048457	-3.518708
H	-4.871659	-2.529097	-4.261898
H	-3.387307	-1.605198	-4.108951
C	-0.091730	5.206424	-1.147244
C	5.220865	3.349527	0.576031
H	-9.422469	6.597135	1.532546
H	6.005261	4.123220	0.525202
H	5.136270	3.107953	1.649719
H	-0.064550	5.933295	-1.981770
H	-0.563900	4.303549	-1.571705
H	-7.636432	7.144550	-3.684721
H	-1.755730	-6.039315	0.878972
H	-2.087809	-7.121574	-0.463799
H	7.406047	-2.433299	1.041842

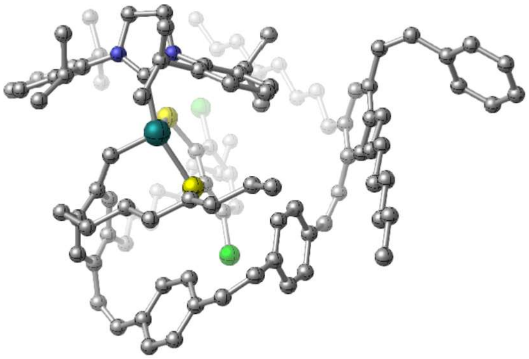

Zero-point correction= 2.134562 (Hartree/Particle)

Thermal correction to Energy= 2.251719

Thermal correction to Enthalpy= 2.252663

Thermal correction to Gibbs Free Energy= 1.969585

Sum of electronic and zero-point Energies= -5959.892604

Sum of electronic and thermal Energies= -5959.775447

Sum of electronic and thermal Enthalpies= -5959.774503

Sum of electronic and thermal Free Energies= -5960.057581

M06L-D3/def2tzvpp-SDD(Ru)-SMD(benzene).

E(scf) = -5966.22235542 a.u.

Int2-β1 (^{n}Oct)

E(scf) = -5961.99607563 a.u.

ν_{min} = 5.64 cm^{-1}

S	-1.551222	0.433060	2.807384
C	-2.083818	-2.218928	1.721139
Ru	-1.984834	-0.378266	0.705059
C	-3.841014	-0.121818	0.626466
C	-1.417455	-1.245957	-1.751558
S	-1.026896	1.661314	-0.088791
N	-0.948368	-2.926565	1.990810
N	-3.037037	-2.668238	2.587060
C	0.258287	-3.008142	1.213013
C	-1.042081	-3.687081	3.250721
C	-2.550167	-3.722773	3.494284
C	-4.453666	-2.469434	2.465352
H	-3.002927	-4.693564	3.230383
H	-2.822296	-3.491523	4.533397
H	-0.586739	-4.681268	3.148936
C	2.650853	-3.398181	-0.182536
C	1.432050	-2.349425	1.646198
C	0.271787	-3.858679	0.072851
C	1.481909	-4.027906	-0.610940
C	2.622416	-2.568482	0.931656
H	1.518821	-4.662815	-1.496305
H	3.536864	-2.059991	1.240203
C	-7.192844	-2.047397	2.133573
C	-5.174732	-3.278779	1.553166
C	-5.107567	-1.517368	3.279330
C	-6.481780	-1.314344	3.081784
C	-6.546058	-3.036550	1.392493
C	-4.532050	-4.443112	0.807296
C	-4.385612	-0.769114	4.389279
H	-7.002564	-0.566957	3.683520
H	-7.120300	-3.641801	0.687566
H	-0.509131	-3.147430	4.050592
H	-3.459418	-4.437422	1.030116
H	-3.308662	-0.921101	4.243833
C	1.458973	-1.399491	2.833750
H	0.432135	-1.285514	3.201331
C	-0.978640	-4.601641	-0.395917
H	-1.841336	-3.951878	-0.189178
C	-0.973579	2.092287	2.640017
C	-0.736562	2.884957	3.779808
C	-0.757272	2.645134	1.350979
C	-0.320297	4.211723	3.678916
C	-0.359848	3.997084	1.276543
C	-0.143361	4.775027	2.413885
H	-0.149509	4.800073	4.581271
H	0.156162	5.817284	2.308700
H	-4.455322	-1.004991	0.394769
C	-2.748605	-0.967478	-1.973986
H	-3.412087	-1.749061	-1.608571
H	-1.238044	-2.178460	-1.217674
C	-0.182320	-0.659332	-2.313022
C	-0.070901	-0.118953	-3.606845
C	0.982955	-0.702374	-1.528280
C	1.139725	0.409775	-4.056223

H	-0.938908	-0.106647	-4.267949
C	2.187496	-0.183012	-1.971878
C	2.298718	0.408548	-3.249252
H	3.043789	-0.238013	-1.306887
H	3.582588	-3.540214	-0.733362
H	-8.260102	-1.863546	1.986020
Cl	-0.967433	2.205985	5.387959
Cl	-0.150623	4.765110	-0.290176
C	2.320159	-1.954673	3.980214
H	3.373671	-2.066643	3.675701
H	2.290944	-1.274032	4.846315
H	1.966024	-2.944584	4.310383
C	1.920544	0.006247	2.409989
H	1.289389	0.401294	1.600413
H	1.843076	0.702887	3.259240
H	2.964715	0.008770	2.059758
C	-1.184650	-5.906051	0.395704
H	-0.331282	-6.586537	0.244939
H	-1.283527	-5.725528	1.474118
H	-2.097181	-6.423095	0.059051
C	-0.993957	-4.900849	-1.902519
H	-0.264409	-5.680420	-2.172683
H	-1.987416	-5.271251	-2.197814
H	-0.772529	-4.006581	-2.503741
C	-5.095631	-5.783428	1.313904
H	-4.565789	-6.627393	0.843439
H	-4.989891	-5.878136	2.406056
H	-6.166348	-5.881613	1.072730
C	-4.665501	-4.336024	-0.716896
H	-5.716816	-4.310202	-1.040128
H	-4.177686	-3.427993	-1.094189
H	-4.187282	-5.199128	-1.205054
C	-4.764124	-1.359916	5.760567
H	-4.196872	-0.860874	6.562989

H	-5.838172	-1.221889	5.968921
H	-4.552967	-2.440174	5.812990
C	-4.633485	0.745740	4.357540
H	-3.964079	1.249763	5.071315
H	-4.422621	1.156570	3.361306
H	-5.672323	0.996671	4.628377
C	-4.125998	2.397232	0.661055
C	-4.649145	1.082725	0.580622
C	-5.985152	0.953388	0.120473
C	-6.628785	2.011216	-0.512316
C	-5.943026	3.221458	-0.728197
C	-4.755092	3.443016	-0.004764
C	-6.256264	4.082078	-1.911981
H	-6.855492	4.994156	-1.806667
C	-5.516260	1.258293	-3.192222
C	-4.885553	2.511918	-3.232941
C	-3.477179	2.565657	-3.153985
C	-2.801283	1.381036	-2.858110
C	-3.446072	0.154820	-2.620820
C	-4.844649	0.076310	-2.865793
C	-5.723841	3.747181	-3.106171
H	-5.877739	4.402367	-3.972440
H	-1.732375	1.434804	-2.699510
H	-6.598905	1.241301	-3.308328
H	-7.596826	1.847301	-0.990941
H	-3.169204	2.567838	1.142147
C	3.532421	1.013339	-3.775933
H	3.426223	1.440278	-4.779642
C	4.749307	1.130083	-3.202244
H	5.523652	1.649258	-3.781744
C	5.156668	0.635878	-1.856501
C	4.961650	1.431966	-0.701529
C	5.684217	-0.651606	-1.719414
C	5.288443	0.873460	0.535553

C	6.026187	-1.212118	-0.477265
H	5.798614	-1.262959	-2.619213
C	5.827860	-0.421306	0.677005
H	5.126928	1.454268	1.445033
C	6.088089	-0.900908	2.060083
H	5.215217	-0.893334	2.724953
C	7.251784	-1.319423	2.594359
H	7.223839	-1.703993	3.620819
C	8.581215	-1.323993	1.951702
C	9.016193	-0.256314	1.142003
C	9.459884	-2.403083	2.164067
C	10.279510	-0.280247	0.548184
H	8.354514	0.596518	0.981664
C	10.716709	-2.435351	1.555591
H	9.142145	-3.234269	2.799657
C	11.131233	-1.373736	0.743978
H	10.600075	0.558296	-0.075296
H	11.375318	-3.293081	1.714197
H	12.115698	-1.396470	0.270054
H	-4.249050	4.409045	-0.078777
H	-6.459162	-0.030972	0.144365
C	-7.114191	-1.198550	-2.890524
H	-7.326610	-0.940107	-3.942973
H	-7.579736	-0.406309	-2.279471
C	-7.773093	-2.534556	-2.541097
H	-7.306712	-3.344240	-3.132261
H	-7.555646	-2.771221	-1.484174
C	-9.287161	-2.554316	-2.754122
H	-9.746689	-1.731819	-2.174836
H	-9.512650	-2.337005	-3.814581
C	-9.940684	-3.879397	-2.355403
H	-9.709411	-4.093679	-1.294801
H	-9.480125	-4.701951	-2.933932
C	-11.457165	-3.906683	-2.556719
H	-11.917010	-3.083229	-1.978553
H	-11.688415	-3.693231	-3.617202
C	-12.110124	-5.231797	-2.154422
H	-11.876556	-5.443166	-1.094726
H	-11.648579	-6.052888	-2.733063
C	-13.625486	-5.247794	-2.359244
H	-14.115665	-4.457957	-1.765156
H	-13.885862	-5.072726	-3.416873
C	-1.220624	3.723881	-3.499902
H	-0.783537	3.169996	-2.655407
H	-1.052064	3.100192	-4.396364
C	-0.447632	5.035369	-3.636762
H	-0.698608	5.694721	-2.786860
H	-0.764321	5.583543	-4.543322
C	1.066177	4.796233	-3.664122
H	1.343285	4.292162	-4.608741
H	1.323618	4.082085	-2.861272
C	1.916047	6.052832	-3.476594
H	1.661708	6.804922	-4.246648
H	1.656699	6.513449	-2.505006
C	3.418493	5.765737	-3.507161
H	3.636754	4.940536	-2.807537
H	3.702808	5.389553	-4.507848
C	4.292114	6.968774	-3.146280
H	4.100583	7.790256	-3.860558
H	3.982167	7.352104	-2.156922
C	5.785246	6.639788	-3.121019
H	6.002162	5.850163	-2.381276
H	6.129823	6.272575	-4.102585
H	0.932528	-1.126932	-0.529932
H	1.190851	0.838526	-5.060984
C	4.141036	3.611397	0.443023
H	3.448389	3.060297	1.104383
H	5.090590	3.706419	0.999988

C	3.571253	5.003870	0.169711
H	2.641045	4.902994	-0.413525
H	4.272251	5.567993	-0.470972
C	3.280415	5.810721	1.435063
H	4.221115	5.981582	1.991477
H	2.636570	5.213041	2.104948
C	2.599014	7.151188	1.152442
H	1.651687	6.962034	0.616034
H	3.223732	7.743046	0.457265
C	2.316646	7.977617	2.408504
H	3.269251	8.198673	2.925457
H	1.728297	7.370165	3.122229
C	1.572549	9.286363	2.131590
H	2.159707	9.892410	1.417485
H	0.620577	9.057354	1.618460
C	1.292508	10.104179	3.392951
H	2.229766	10.375420	3.907912
H	0.678280	9.532084	4.109009
C	7.908699	-2.827006	-1.126997
H	7.817324	-2.602589	-2.204817
H	8.623203	-2.095614	-0.722869
C	8.477512	-4.233767	-0.941016
H	8.549736	-4.445947	0.141293
H	7.774094	-4.982159	-1.352398
C	9.856557	-4.418309	-1.576716
H	9.787525	-4.239153	-2.665963
H	10.535775	-3.640294	-1.184033
C	10.471507	-5.796126	-1.324777
H	10.539177	-5.967709	-0.233915
H	9.791474	-6.579225	-1.709448
C	11.856609	-5.976312	-1.949648
H	11.790573	-5.805588	-3.040684
H	12.534448	-5.191023	-1.564913
C	12.478762	-7.350995	-1.690763
H	12.542952	-7.519324	-0.600022
H	11.799517	-8.133731	-2.075500
C	13.863167	-7.518647	-2.318487
H	14.571432	-6.769371	-1.925878
H	13.821925	-7.389265	-3.413340
H	14.285305	-8.516106	-2.115510
H	0.754791	11.038227	3.163186
H	6.394999	7.519511	-2.858999
H	-14.066952	-6.212510	-2.061609
C	-2.723149	3.878253	-3.255512
H	-2.876124	4.441656	-2.315855
H	-3.177213	4.499971	-4.047143
C	-5.605643	-1.217604	-2.644844
H	-5.447491	-1.531641	-1.599203
H	-5.153118	-2.020392	-3.253655
C	4.361412	2.815060	-0.841698
H	3.396596	2.719440	-1.370748
H	4.998360	3.401011	-1.529275
C	6.545122	-2.635240	-0.443003
H	5.811212	-3.292765	-0.943197
H	6.611105	-2.985688	0.596212

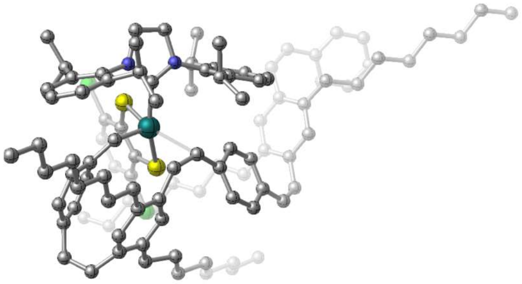

Zero-point correction= 2.133514 (Hartree/Particle)

Thermal correction to Energy= 2.250799

Thermal correction to Enthalpy= 2.251743

Thermal correction to Gibbs Free Energy= 1.967293

Sum of electronic and zero-point Energies= -5959.862562

Sum of electronic and thermal Energies= -5959.745277

Sum of electronic and thermal Enthalpies= -5959.744332

Sum of electronic and thermal Free Energies= -5960.028783

M06L-D3/def2tzvpp-SDD(Ru)-SMD(benzene).

E(scf) = -5966.19425979 a.u.

M2

E(scf) = -1395.35626315 a.u.

ν_{min} = 5.9677 cm^{-1}

O	2.653229	0.775344	-0.983978	C	0.714517	2.627569	-0.171367
O	-2.653226	-0.775482	-0.983861	H	0.995011	3.537294	-0.715025
C	1.357584	0.360666	-1.053445	C	-0.714510	-2.627602	-0.171025
C	0.373901	1.358447	-0.898731	H	-0.995001	-3.537400	-0.714563
C	-0.970559	0.987390	-1.044840	C	3.696542	-0.173989	-0.837188
H	-1.726796	1.763752	-0.932249	H	3.722384	-0.856003	-1.709032
C	-1.357582	-0.360814	-1.053391	H	3.523985	-0.795432	0.062433
C	-0.373898	-1.358574	-0.898554	C	5.007172	0.584463	-0.714098
C	0.970561	-0.987536	-1.044719	H	4.932020	1.274154	0.144505
H	1.726799	-1.763884	-0.932034	H	5.133648	1.215816	-1.610494

C	6.213590	-0.340042	-0.544107	H	-7.686008	-1.048587	-1.307772
H	6.067845	-0.976092	0.348729	H	-7.482023	-1.107814	0.442760
H	6.270681	-1.034266	-1.402905	C	-8.752198	0.505137	-0.242705
C	7.539958	0.412510	-0.415179	H	-8.808375	1.200702	-1.100842
H	7.482017	1.107917	0.442596	H	-8.604154	1.141456	0.649810
H	7.686016	1.048377	-1.307923	C	-10.079194	-0.245849	-0.113276
C	8.752195	-0.505159	-0.242570	H	-10.227194	-0.882376	-1.005870
H	8.604145	-1.141312	0.650062	H	-10.022739	-0.941929	0.744686
H	8.808374	-1.200883	-1.100579	C	-11.293423	0.670631	0.059735
C	10.079191	0.245849	-0.113274	H	-11.347343	1.365617	-0.798227
H	10.227199	0.882205	-1.005988	H	-11.142988	1.306008	0.951747
H	10.022732	0.942091	0.744556	C	-12.613698	-0.090296	0.187989
C	11.293417	-0.670601	0.059920	H	-12.805583	-0.707740	-0.705898
H	11.142976	-1.305807	0.952052	H	-13.468430	0.594144	0.311178
H	11.347340	-1.365752	-0.797909	H	-12.599499	-0.767949	1.058437
C	12.613693	0.090347	0.188035	C	-1.338324	-0.375717	2.073368
H	12.805584	0.707620	-0.705970	C	-0.354126	-1.366243	1.909062
H	13.468423	-0.594071	0.311359	C	0.986430	-0.976978	2.073786
H	12.599491	0.768167	1.058353	H	1.775943	-1.730777	2.010231
C	-3.696539	0.173872	-0.837213	C	1.338322	0.375980	2.073327
H	-3.722378	0.855762	-1.709155	C	0.354125	1.366485	1.908886
H	-3.523987	0.795445	0.062320	C	-0.986432	0.977241	2.073652
C	-5.007171	-0.584561	-0.714020	H	-1.775945	1.731032	2.009993
H	-4.932030	-1.274117	0.144692	C	-0.691447	-2.635426	1.181726
H	-5.133635	-1.216054	-1.610319	H	-0.942487	-3.554476	1.724802
C	-6.213591	0.339971	-0.544190	C	0.691451	2.635571	1.181382
H	-6.270679	1.034049	-1.403107	H	0.942493	3.554693	1.724337
H	-6.067851	0.976172	0.348538	H	2.393750	0.651904	2.004652
C	-7.539959	-0.412561	-0.415140	H	-2.393751	-0.651649	2.004723

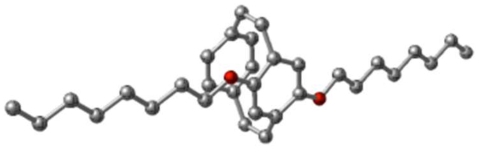

Zero-point correction= 0.687600 (Hartree/Particle)

Thermal correction to Energy= 0.723129

Thermal correction to Enthalpy= 0.724073

Thermal correction to Gibbs Free Energy= 0.614960

Sum of electronic and zero-point Energies= -1394.668663

Sum of electronic and thermal Energies= -1394.633135

Sum of electronic and thermal Enthalpies= -1394.632190

Sum of electronic and thermal Free Energies= -1394.741304

M06L-D3/def2tzvpp-SDD(Ru)-SMD(benzene).

E(scf) = -1396.61457512 a.u.

ent-M2

E(scf) = -1395.35626315 a.u.

ν_{min} = 5.9683 cm^{-1}

C	-0.970557	-0.987576	-1.044743	H	2.393735	-0.651567	2.004714
C	0.373903	-1.358607	-0.898567	C	-0.691469	2.635613	1.181220
C	1.357585	-0.360852	-1.053443	H	-0.942517	3.554753	1.724139
C	0.970564	0.987352	-1.044939	C	0.691452	-2.635381	1.181758
C	-0.373897	1.358413	-0.898841	H	0.942488	-3.554411	1.724870
C	-1.357579	0.360627	-1.053520	H	-1.775956	-1.730712	2.010193
C	0.714517	-2.627609	-0.170992	H	1.775920	1.731109	2.009892
H	0.995010	-3.537427	-0.714495	O	-2.653225	0.775306	-0.984077
C	-0.714514	2.627564	-0.171529	O	2.653231	-0.775517	-0.983904
H	-0.995001	3.537271	-0.715222	H	1.726803	1.763716	-0.932377
C	0.986408	0.977319	2.073566	H	-1.726797	-1.763918	-0.932029
C	-0.354149	1.366551	1.908770	C	-3.696536	-0.174022	-0.837223
C	-1.338344	0.376049	2.073230	H	-3.722397	-0.856074	-1.709036
H	-2.393772	0.651964	2.004533	H	-3.523958	-0.795423	0.062423
C	-0.986446	-0.976909	2.073736	C	3.696543	0.173841	-0.837262
C	0.354114	-1.366173	1.909044	H	3.722402	0.855700	-1.709227
C	1.338305	-0.375638	2.073332	H	3.523969	0.795444	0.062246

C	5.007170	-0.584589	-0.714010	C	-5.007164	0.584436	-0.714140
H	5.133655	-1.216117	-1.610282	H	-5.133660	1.215748	-1.610562
H	4.932011	-1.274110	0.144729	H	-4.931995	1.274164	0.144432
C	6.213588	0.339950	-0.544190	C	-6.213578	-0.340063	-0.544081
H	6.270697	1.033991	-1.403135	H	-6.270688	-1.034324	-1.402848
H	6.067825	0.976189	0.348508	H	-6.067812	-0.976074	0.348779
C	7.539952	-0.412576	-0.415074	C	-7.539944	0.412494	-0.415155
H	7.686022	-1.048644	-1.307673	H	-7.686023	1.048320	-1.307925
H	7.481998	-1.107789	0.442858	H	-7.481983	1.107939	0.442588
C	8.752188	0.505129	-0.242654	C	-8.752176	-0.505168	-0.242475
H	8.808386	1.200653	-1.100824	H	-8.808375	-1.200931	-1.100450
H	8.604123	1.141491	0.649827	H	-8.604105	-1.141279	0.650183
C	10.079180	-0.245851	-0.113158	C	-10.079169	0.245846	-0.113183
H	10.227202	-0.882421	-1.005718	H	-10.227200	0.882158	-1.005925
H	10.022705	-0.941888	0.744838	H	-10.022689	0.942130	0.744612
C	11.293406	0.670638	0.059837	C	-11.293391	-0.670597	0.060085
H	11.347346	1.365581	-0.798159	H	-11.142928	-1.305756	0.952247
H	11.142950	1.306058	0.951814	H	-11.347334	-1.365790	-0.797707
C	12.613677	-0.090284	0.188160	C	-12.613664	0.090358	0.188193
H	13.468406	0.594162	0.311334	H	-12.805576	0.707585	-0.705839
H	12.599457	-0.767893	1.058641	H	-13.468390	-0.594054	0.311573
H	12.805582	-0.707773	-0.705693	H	-12.599441	0.768223	1.058476

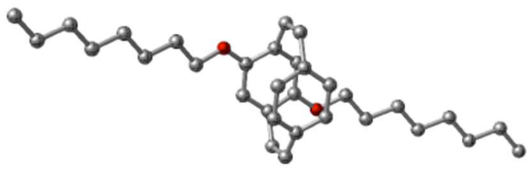

Zero-point correction= 0.687600 (Hartree/Particle)

Thermal correction to Energy= 0.723129

Thermal correction to Enthalpy= 0.724073

Thermal correction to Gibbs Free Energy= 0.614960

Sum of electronic and zero-point Energies= -1394.668663

Sum of electronic and thermal Energies= -1394.633135

Sum of electronic and thermal Enthalpies= -1394.632190

Sum of electronic and thermal Free Energies= -1394.741303

M06L-D3/def2tzvpp-SDD(Ru)-SMD(benzene).

E(scf) = -1396.61457512 a.u.

M4

E(scf) = -1245.04162691 a.u.

ν_{min} = 6.3661 cm^{-1}

C	0.317681	2.703088	1.137274	H	1.916191	-1.543009	-1.038517
H	0.411358	3.653382	1.676608	H	-1.916198	1.542862	-1.038634
C	0.352789	2.692653	-0.216356	C	3.871958	0.101962	-0.817898
H	0.483542	3.637466	-0.758010	H	3.902416	-0.683721	-1.593286
C	-1.111557	0.832707	2.035493	H	3.673905	-0.418343	0.136287
C	0.162698	1.401846	1.870193	C	5.240079	0.783912	-0.746650
C	1.272733	0.555801	2.038116	H	5.437007	1.311558	-1.698425
C	1.111595	-0.832497	2.035579	H	5.215958	1.569555	0.031483
C	-0.162661	-1.401655	1.870358	C	6.390956	-0.181062	-0.454265
C	-1.272695	-0.555591	2.038194	H	6.416521	-0.965675	-1.233336
C	-0.317651	-2.702983	1.137593	H	6.190305	-0.709652	0.496386
H	-0.411322	-3.653214	1.677039	C	7.759895	0.498379	-0.375585
C	1.073578	-0.853439	-1.109103	H	7.960325	1.026051	-1.326797
C	-0.215486	-1.383961	-0.944407	H	7.732761	1.283977	0.402597
C	-1.325575	-0.525575	-1.116497	C	8.911080	-0.465510	-0.080137
C	-1.073587	0.853283	-1.109168	H	8.938399	-1.251430	-0.858266
C	0.215482	1.383825	-0.944571	H	8.710427	-0.993315	0.871218
C	1.325566	0.525419	-1.116596	C	10.281004	0.213658	-0.000865
C	-0.352776	-2.692705	-0.216038	H	10.479274	0.740371	-0.952336
H	-0.483540	-3.637581	-0.757580	H	10.250878	0.999208	0.776344
H	1.998116	-1.468864	1.968872	C	11.423638	-0.758470	0.295695
H	-1.998078	1.469066	1.968720	H	11.267775	-1.273923	1.258576
H	2.281728	0.972006	1.977246	H	11.497957	-1.535123	-0.484318
H	-2.281691	-0.971803	1.977383	C	-3.871965	-0.102071	-0.817851

H	-3.902415	0.683485	-1.593370	C	-10.281025	-0.213603	-0.000907
H	-3.673918	0.418392	0.136249	H	-10.250917	-0.999000	0.776457
C	-5.240087	-0.784006	-0.746501	H	-10.479275	-0.740504	-0.952278
H	-5.437003	-1.311821	-1.698185	C	-11.423664	0.758585	0.295435
H	-5.215980	-1.569511	0.031772	H	-11.497965	1.535083	-0.484734
C	-6.390968	0.181022	-0.454306	H	-11.267821	1.274231	1.258217
H	-6.416520	0.965495	-1.233519	C	-2.735403	-1.084229	-1.106640
H	-6.190329	0.709784	0.496253	H	-2.777407	-1.902216	-0.367824
C	-7.759909	-0.498401	-0.375523	H	-2.925503	-1.572158	-2.081533
H	-7.960320	-1.026254	-1.326638	C	2.735396	1.084072	-1.106855
H	-7.732793	-1.283850	0.402810	H	2.777409	1.902192	-0.368187
C	-8.911099	0.465547	-0.080284	H	2.925490	1.571822	-2.081838
H	-8.938400	1.251315	-0.858566	H	12.395673	-0.241941	0.346657
H	-8.710463	0.993536	0.870973	H	-12.395701	0.242068	0.346479

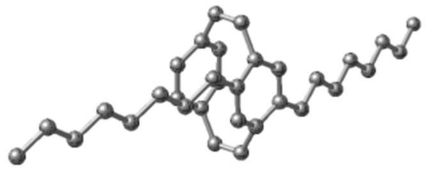

Zero-point correction= 0.678414 (Hartree/Particle)

Thermal correction to Energy= 0.711821

Thermal correction to Enthalpy= 0.712765

Thermal correction to Gibbs Free Energy= 0.609085

Sum of electronic and zero-point Energies= -1244.363213

Sum of electronic and thermal Energies= -1244.329806

Sum of electronic and thermal Enthalpies= -1244.328862

Sum of electronic and thermal Free Energies= -1244.432542

M06L-D3/def2tzvpp-SDD(Ru)-SMD(benzene).

E(scf) = -1246.14665515 a.u.

ent-M4

E(scf) = -1245.04162691 a.u.

ν_{min} = 6.3567 cm^{-1}

C	0.352705	-2.692719	-0.215829	H	3.902371	0.683380	-1.593377
H	0.483435	-3.637602	-0.757366	C	5.240094	-0.784048	-0.746483
C	0.317548	-2.702988	1.137802	H	5.216037	-1.569507	0.031841
H	0.411172	-3.653219	1.677257	H	5.436956	-1.311936	-1.698140
C	-1.073596	-0.853427	-1.108920	C	6.390991	0.180994	-0.454424
C	0.215462	-1.383977	-0.944210	H	6.190409	0.709828	0.496108
C	1.325569	-0.525610	-1.116301	H	6.416498	0.965417	-1.233691
C	1.073611	0.853253	-1.108992	C	7.759936	-0.498418	-0.375673
C	-0.215454	1.383824	-0.944397	H	7.960289	-1.026351	-1.326757
C	-1.325555	0.525434	-1.116416	H	7.732868	-1.283811	0.402721
C	-0.352719	2.692661	-0.216191	C	8.911145	0.465546	-0.080580
H	-0.483433	3.637474	-0.757855	H	8.710566	0.993618	0.870644
C	1.111580	0.832685	2.035673	H	8.938402	1.251255	-0.858927
C	-0.162663	1.401866	1.870370	C	10.281074	-0.213595	-0.001228
C	-1.272727	0.555855	2.038302	H	10.479264	-0.740582	-0.952565
H	-2.281704	0.972105	1.977430	H	10.251011	-0.998928	0.776204
C	-1.111634	-0.832446	2.035766	C	11.423732	0.758610	0.294961
C	0.162611	-1.401649	1.870558	H	11.267951	1.274344	1.257707
C	1.272673	-0.555616	2.038395	H	11.497994	1.535043	-0.485278
H	2.281651	-0.971874	1.977593	C	-5.240080	0.783942	-0.746663
C	-0.317589	2.703108	1.137440	H	-5.216012	1.569556	0.031504
H	-0.411222	3.653410	1.676768	H	-5.436953	1.311641	-1.698422
H	-1.998165	-1.468799	1.969061	C	-6.390976	-0.181039	-0.454396
H	1.998112	1.469029	1.968896	H	-6.416493	-0.965620	-1.233503
H	1.916233	1.542821	-1.038477	H	-6.190383	-0.709681	0.496241
H	-1.916221	-1.542987	-1.038347	C	-7.759918	0.498392	-0.375766
C	3.871971	-0.102124	-0.817801	H	-7.960288	1.026122	-1.326960
H	3.673982	0.418417	0.136270	H	-7.732835	1.283951	0.402459
C	-3.871959	0.102002	-0.817860	C	-8.911123	-0.465507	-0.080446
H	-3.902365	-0.683647	-1.593290	H	-8.938395	-1.251384	-0.858621
H	-3.673966	-0.418362	0.136306	H	-8.710529	-0.993373	0.870890

C	-10.281050	0.213654	-0.001217	H	-2.925415	1.571962	-2.081634
H	-10.479257	0.740431	-0.952667	H	-2.777400	1.902172	-0.367948
H	-10.250970	0.999159	0.776040	C	2.735387	-1.084296	-1.106441
C	-11.423703	-0.758483	0.295210	H	2.925438	-1.572339	-2.081286
H	-11.267906	-1.274003	1.258068	H	2.777402	-1.902200	-0.367535
H	-11.497981	-1.535090	-0.484855	H	12.395773	0.242097	0.345994
C	-2.735374	1.084119	-1.106687	H	-12.395743	-0.241958	0.346145

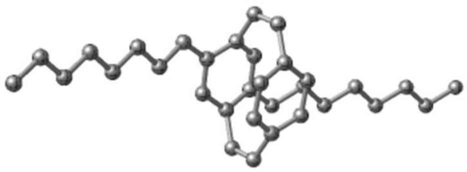

Zero-point correction= 0.678414 (Hartree/Particle)

Thermal correction to Energy= 0.711820

Thermal correction to Enthalpy= 0.712765

Thermal correction to Gibbs Free Energy= 0.609083

Sum of electronic and zero-point Energies= -1244.363213

Sum of electronic and thermal Energies= -1244.329807

Sum of electronic and thermal Enthalpies= -1244.328862

Sum of electronic and thermal Free Energies= -1244.432544

M06L-D3/def2tzvpp-SDD(Ru)-SMD(benzene).

E(scf) = -1246.14665515 a.u.

M (OMe)

E(scf) = -845.309882745 a.u.

ν_{min} = 48.2083 cm^{-1}

C	-1.516638	-2.263577	1.009743	H	-2.098214	-3.017026	-0.886765
H	-2.054330	-3.050041	1.552858	C	0.611681	-1.246631	1.901519
C	-1.535835	-2.248998	-0.343018	C	-0.782309	-1.174703	1.736932

C	-1.387609	0.083668	1.900578	H	1.110388	-2.217629	1.838088
C	-0.611641	1.246509	1.901645	H	-2.474898	0.169031	1.828659
C	0.782343	1.174592	1.736985	H	2.474934	-0.169150	1.828521
C	1.387650	-0.083789	1.900504	O	-2.762336	0.135592	-1.169793
C	1.516633	2.263527	1.009847	O	2.762318	-0.135522	-1.169796
H	2.054321	3.049969	1.552998	H	-1.054143	2.230861	-1.098086
C	-0.593724	1.250084	-1.214673	H	1.054120	-2.230801	-1.098183
C	0.798339	1.161545	-1.070474	C	-3.423927	1.380852	-1.118727
C	1.400663	-0.102972	-1.229139	H	-3.206723	2.000208	-2.007954
C	0.593701	-1.250020	-1.214729	H	-3.153351	1.954349	-0.213419
C	-0.798359	-1.161488	-1.070526	C	3.423930	-1.380774	-1.118815
C	-1.400683	0.103040	-1.229135	H	3.206726	-2.000081	-2.008076
C	1.535805	2.249029	-0.342915	H	3.153380	-1.954332	-0.213538
H	2.098161	3.017099	-0.886627	H	-4.500242	1.163049	-1.091797
H	-1.110349	2.217513	1.838316	H	4.500242	-1.162954	-1.091887

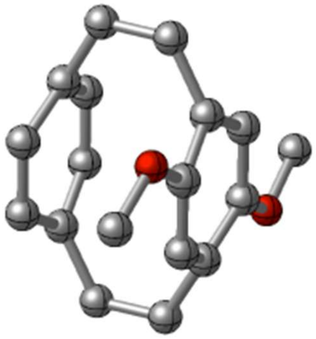

Zero-point correction= 0.290626 (Hartree/Particle)

Thermal correction to Energy= 0.307083

Thermal correction to Enthalpy= 0.308027

Thermal correction to Gibbs Free Energy= 0.247891

Sum of electronic and zero-point Energies= -845.019257

Sum of electronic and thermal Energies= -845.002800

Sum of electronic and thermal Enthalpies= -845.001856

Sum of electronic and thermal Free Energies= -845.061992

M' (OMe)

E(scf) = -845.309882745 a.u.

ν_{min} = 48.2041 cm^{-1}

C	-1.535813	2.249047	-0.342808	C	-1.387718	-0.083898	1.900483
H	-2.098169	3.017138	-0.886488	H	-2.475003	-0.169241	1.828481
C	-1.516666	2.263471	1.009955	C	1.516557	-2.263667	1.009641
H	-2.054382	3.049871	1.553139	H	2.054208	-3.050184	1.552720
C	0.593757	1.250176	-1.214537	H	1.110298	2.217384	1.838466
C	-0.798309	1.161623	-1.070411	H	-1.110469	-2.217736	1.837950
C	-1.400626	-0.102884	-1.229199	H	-1.054077	-2.230719	-1.098400
C	-0.593658	-1.249924	-1.214844	H	1.054159	2.230948	-1.097854
C	0.798396	-1.161400	-1.070569	O	-2.762280	-0.135441	-1.169915
C	1.400731	0.103136	-1.229043	O	2.762385	0.135662	-1.169646
C	1.535822	-2.248979	-0.343116	C	-3.423873	-1.380716	-1.119097
H	2.098211	-3.016973	-0.886898	H	-3.153361	-1.954348	-0.213856
C	-0.611758	-1.246743	1.901436	C	3.424034	1.380897	-1.118531
C	0.782238	-1.174819	1.736878	H	3.206866	2.000286	-2.007742
C	1.387541	0.083533	1.900607	H	3.153477	1.954367	-0.213202
H	2.474831	0.168895	1.828701	H	-3.206592	-1.999920	-2.008410
C	0.611579	1.246380	1.901727	H	4.500337	1.163031	-1.091600
C	-0.782400	1.174489	1.737045	H	-4.500189	-1.162908	-1.092217

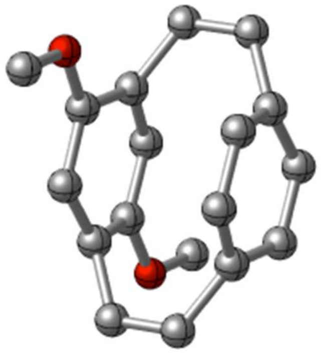

Zero-point correction= 0.290626 (Hartree/Particle)

Thermal correction to Energy= 0.307083

Thermal correction to Enthalpy= 0.308027

Thermal correction to Gibbs Free Energy= 0.247891

Sum of electronic and zero-point Energies= -845.019257

Sum of electronic and thermal Energies= -845.002800

Sum of electronic and thermal Enthalpies= -845.001856

Sum of electronic and thermal Free Energies= -845.061992

Ru-1b-M' (OMe)

E(scf) = -4081.34585484 a.u.

ν_{min} = 12.1014 cm^{-1}

S	-2.188670	1.152621	-1.162241	C	3.692585	-0.789794	-3.332795
C	-1.087340	-1.457224	-1.371422	C	1.892316	-2.350297	-2.784134
Ru	-0.582732	-0.004869	0.086817	C	1.377025	0.011780	-3.206641
C	1.347056	-1.275188	0.277571	C	2.744091	0.229126	-3.438396
C	-1.759624	-0.573167	1.442598	C	3.238541	-2.081746	-3.038454
S	0.220016	2.124265	0.694996	C	1.461000	-3.758863	-2.457067
N	-2.265370	-2.115075	-1.507762	C	0.425758	1.167988	-3.372232
N	-0.408686	-1.591112	-2.540414	H	3.072505	1.240076	-3.697439
C	-3.192604	-2.470907	-0.475638	H	3.961414	-2.898402	-2.961294
C	-2.384317	-2.813627	-2.802662	H	-3.368145	-2.628634	-3.255286
C	-1.230565	-2.208410	-3.597696	H	1.019766	-4.261186	-3.335403
C	0.966247	-1.279680	-2.825868	H	-0.612150	0.843657	-3.505981
H	-0.654087	-2.950128	-4.165616	C	-4.932606	-0.845810	-1.424747
H	-1.567209	-1.424654	-4.297463	H	-4.240679	-0.712807	-2.264132
H	-2.276574	-3.901617	-2.650785	C	-1.465277	-4.132736	0.367813
C	-5.047493	-3.345485	1.451291	H	-0.666189	-3.391371	0.513703
C	-4.482167	-1.908285	-0.454890	C	-1.845134	2.869758	-0.990653
C	-2.829726	-3.494660	0.430435	C	-2.628699	3.839033	-1.653407
C	-3.763617	-3.907485	1.384597	C	-0.779249	3.302711	-0.163303
C	-5.383997	-2.358524	0.521485	C	-2.367176	5.201436	-1.528703
H	-3.483979	-4.692076	2.093683	C	-0.532423	4.688271	-0.048038
H	-6.382749	-1.913258	0.552772	C	-1.308520	5.629192	-0.722201

H	-2.987052	5.923233	-2.062216	C	2.711972	-1.640523	3.160866
H	-1.084593	6.691437	-0.616237	H	2.460375	-2.700573	3.062587
H	1.063522	-2.182366	-0.248882	C	5.878715	0.366897	3.160635
C	0.877123	-1.180015	1.605559	H	6.576284	0.635383	3.963502
H	0.228868	-1.993085	1.942208	H	1.458640	1.463631	2.618689
H	-1.629189	-1.578949	1.865869	H	4.734350	-2.079916	3.745485
C	-2.853285	0.108136	2.125382	H	5.739222	-2.117701	0.933013
C	-3.154701	1.485928	2.006862	H	2.363545	1.282925	-0.238676
C	-3.656067	-0.668912	2.999227	O	3.358365	-3.081463	0.103142
C	-4.229975	2.043261	2.696518	O	4.784519	2.247629	0.447471
H	-2.525861	2.122379	1.393141	C	4.300545	-4.099005	0.352632
C	-4.737220	-0.112470	3.678240	H	4.674209	-4.070230	1.392315
H	-3.424978	-1.728137	3.124899	C	3.828271	3.259131	0.185379
C	-5.032864	1.247414	3.523015	H	3.466388	3.221729	-0.858348
H	-4.440540	3.110386	2.591585	H	2.955522	3.190855	0.853849
H	-5.348507	-0.736373	4.335131	H	5.163726	-4.036289	-0.335050
H	-5.877557	1.689606	4.057704	H	4.336185	4.218871	0.350507
Cl	-3.969913	3.338280	-2.673797	H	3.782768	-5.054115	0.188299
Cl	0.786357	5.274977	0.955917	H	2.317569	-4.358195	-2.122807
C	3.133843	0.535941	-0.069220	H	0.704348	-3.784367	-1.661090
C	2.741212	-0.801150	-0.011197	H	0.437390	1.815655	-2.480995
C	3.755869	-1.773121	0.158626	H	0.723152	1.785145	-4.233463
C	5.029217	-1.372938	0.574006	H	-5.932560	-1.084300	-1.819589
C	5.330720	-0.018340	0.796894	H	-4.993768	0.130535	-0.919298
C	4.421526	0.941801	0.314715	H	-1.353773	-4.909849	1.136198
C	6.305618	0.350830	1.877459	H	-1.283423	-4.600996	-0.612997
H	7.344444	0.614790	1.645947	C	5.166449	-0.500804	-3.450571
C	4.007221	-1.288506	3.543703	H	5.715749	-1.345702	-3.894249
C	4.441705	0.046902	3.455159	H	5.362003	0.398507	-4.053968
C	3.451409	1.027147	3.286227	H	5.587485	-0.324914	-2.444759
H	3.734330	2.082842	3.276471	C	-6.017993	-3.760680	2.527616
C	2.154731	0.674087	2.894907	H	-5.855344	-4.802115	2.844403
C	1.821433	-0.666262	2.658730	H	-5.895569	-3.123079	3.420933

H	-7.062177	-3.656540	2.196114

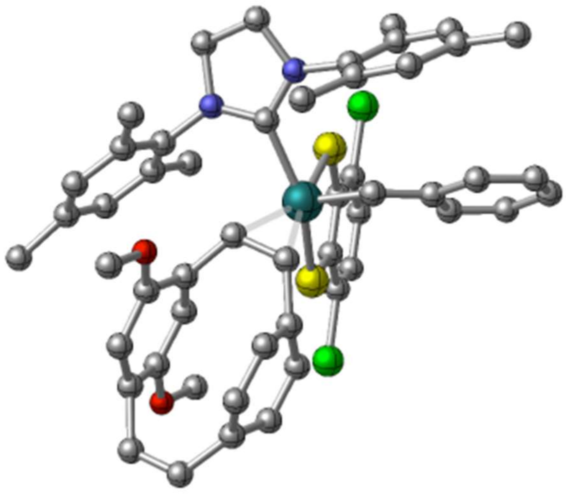

Zero-point correction= 0.887285 (Hartree/Particle)

Thermal correction to Energy= 0.946313

Thermal correction to Enthalpy= 0.947257

Thermal correction to Gibbs Free Energy= 0.792814

Sum of electronic and zero-point Energies= -4080.458570

Sum of electronic and thermal Energies= -4080.399542

Sum of electronic and thermal Enthalpies= -4080.398598

Sum of electronic and thermal Free Energies= -4080.553041

Ru-1b-M (OMe)

E(scf) = -4081.34289732 a.u.

ν_{min} = 13.7990 cm^{-1}

S	2.276483	1.167400	1.068573	N	0.654816	-1.518592	2.754154
C	1.178520	-1.426846	1.503627	C	3.132133	-2.502861	0.370012
Ru	0.503661	-0.004259	0.089504	C	2.627879	-2.775215	2.790233
C	-1.461782	-1.251794	0.178155	C	1.587295	-2.142092	3.711241
C	1.494794	-0.609619	-1.397424	C	-0.663159	-1.162166	3.204744
S	-0.368776	2.120375	-0.440081	H	1.069919	-2.870092	4.350010
N	2.351215	-2.106786	1.503704	H	2.017460	-1.360262	4.359732

H	2.497117	-3.865534	2.680027	C	-1.088874	-1.233445	-1.184122
C	4.708670	-3.452360	-1.758171	H	-0.493045	-2.084344	-1.515013
C	4.413029	-1.957953	0.164868	H	1.316109	-1.625656	-1.774324
C	2.643279	-3.545743	-0.450922	C	2.489348	0.052765	-2.232954
C	3.439229	-3.995367	-1.508053	C	2.784140	1.436567	-2.207295
C	5.174346	-2.446044	-0.908271	C	3.192565	-0.753314	-3.164427
H	3.060924	-4.794231	-2.152371	C	3.762981	1.973743	-3.041332
H	6.164708	-2.015318	-1.082486	H	2.222280	2.093571	-1.552053
C	-3.288308	-0.581559	4.048524	C	4.180368	-0.217177	-3.986850
C	-1.615598	-2.204879	3.310390	H	2.960135	-1.818128	-3.220652
C	-0.988888	0.149475	3.599019	C	4.474457	1.150438	-3.922869
C	-2.309483	0.412201	3.997089	H	3.969789	3.046158	-3.005642
C	-2.909978	-1.892418	3.730238	H	4.718728	-0.863164	-4.684936
C	-1.270673	-3.635064	2.969135	H	5.245028	1.576688	-4.570539
C	0.008485	1.278237	3.625028	Cl	4.310105	3.364602	2.193531
H	-2.573768	1.437513	4.272449	Cl	-0.864171	5.278157	-0.825076
H	-3.656880	-2.689928	3.776086	C	-3.231771	0.584192	0.550397
H	3.662113	-2.587963	3.110355	C	-2.828784	-0.755797	0.546879
H	-0.833529	-4.160630	3.835906	C	-3.848068	-1.731118	0.520652
H	1.044907	0.923159	3.623267	C	-5.151675	-1.384993	0.161247
C	4.997438	-0.876540	1.037521	C	-5.472122	-0.052622	-0.162535
H	4.424856	-0.714456	1.957912	C	-4.539471	0.932646	0.197592
C	1.295073	-4.167935	-0.188911	C	-6.520180	0.263116	-1.189638
H	0.489493	-3.420971	-0.231437	H	-7.552100	0.496740	-0.900274
C	1.947625	2.883259	0.868568	C	-4.352744	-1.397965	-2.874036
C	2.846446	3.857910	1.354003	C	-4.751915	-0.052454	-2.870100
C	0.780949	3.308946	0.185334	C	-3.744379	0.929535	-2.822089
C	2.608759	5.219801	1.186907	C	-2.434287	0.556209	-2.475271
C	0.564134	4.694422	0.017864	C	-2.103602	-0.772437	-2.192738
C	1.458247	5.641237	0.513761	C	-3.042775	-1.769774	-2.557086
H	3.320737	5.946562	1.580337	C	-6.165588	0.287016	-2.494644
H	1.254660	6.703507	0.372938	H	-6.907521	0.556526	-3.255760
H	-1.139041	-2.131425	0.732589	H	-5.877637	-2.178465	-0.037223

H	-2.484318	1.368198	0.662255	H	-0.154314	1.907376	4.513201
H	-3.581693	-2.788495	0.601853	H	-2.169374	-4.194172	2.671325
H	-4.773776	1.984780	0.016571	H	-0.540130	-3.706425	2.151981
O	-2.659253	-3.067534	-2.351034	H	6.039082	-1.116796	1.301944
O	-4.155253	2.223281	-2.920533	H	4.998579	0.086456	0.503547
H	-5.131878	-2.157940	-2.925025	H	1.069319	-4.952278	-0.924094
H	-1.705088	1.326016	-2.240817	H	1.249895	-4.624117	0.813480
C	-3.604820	-4.101428	-2.511051	C	-4.722837	-0.252578	4.371609
H	-4.019957	-4.126153	-3.534998	H	-5.213184	-1.066193	4.928378
H	-4.439547	-4.008426	-1.792725	H	-4.808593	0.673577	4.959603
C	-3.242733	3.264062	-2.621117	H	-5.291132	-0.105941	3.436231
H	-2.371360	3.258045	-3.299933	C	5.524675	-3.907689	-2.941265
H	-2.870292	3.203607	-1.585934	H	5.315544	-4.956283	-3.202209
H	-3.074771	-5.044507	-2.319743	H	5.287511	-3.295519	-3.829341
H	-3.791041	4.206283	-2.756027	H	6.604290	-3.803655	-2.755113
H	-0.102849	1.921305	2.737706				

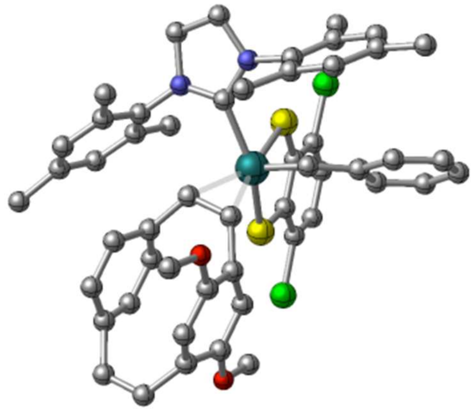

Zero-point correction= 0.887344 (Hartree/Particle)

Thermal correction to Energy= 0.946420

Thermal correction to Enthalpy= 0.947364

Thermal correction to Gibbs Free Energy= 0.792585

Sum of electronic and zero-point Energies= -4080.455554

Sum of electronic and thermal Energies= -4080.396477

Sum of electronic and thermal Enthalpies= -4080.395533

Sum of electronic and thermal Free Energies= -4080.550312

TS1-α (Ru-1b/OMe)

E(scf) = -4081.33690156 a.u.

ν_{min} = -93.9383 cm^{-1}

S	-2.351252	1.325604	-1.000461	C	-0.268727	1.468338	-3.716317
C	-1.364575	-1.308204	-1.649952	H	2.357600	1.788489	-4.075081
Ru	-0.597650	0.051757	-0.221561	H	3.603506	-2.297897	-3.597856
C	1.212846	-1.138442	-0.265611	H	-3.852379	-2.187547	-3.369373
C	-1.299646	-0.823672	1.333553	H	1.084001	-3.934312	-4.057692
S	0.394653	2.162887	0.348969	H	-1.285786	1.061347	-3.701279
N	-2.551171	-1.962182	-1.703642	C	-5.110810	-0.639945	-1.016943
N	-0.827960	-1.349061	-2.897620	H	-4.545975	-0.422681	-1.930389
C	-3.312737	-2.409357	-0.576579	C	-1.579040	-4.245787	-0.280459
C	-2.858132	-2.518332	-3.034971	H	-0.708727	-3.572354	-0.263443
C	-1.718269	-1.962974	-3.896720	C	-2.038819	3.018048	-0.614880
C	0.495079	-0.942392	-3.283686	C	-2.994737	4.015253	-0.899829
H	-1.187267	-2.740110	-4.465677	C	-0.809747	3.389949	-0.016848
H	-2.059990	-1.193699	-4.608482	C	-2.753732	5.357724	-0.617303
H	-2.866535	-3.619953	-2.991552	C	-0.583561	4.758244	0.255490
C	-4.720597	-3.300491	1.689466	C	-1.536438	5.731024	-0.038621
C	-4.513474	-1.771871	-0.219817	H	-3.512900	6.105962	-0.848721
C	-2.843745	-3.535950	0.138623	H	-1.328642	6.777561	0.188419
C	-3.556119	-3.959440	1.264122	H	1.018026	-2.002688	-0.894821
C	-5.187989	-2.228418	0.924597	C	0.676869	-1.376987	1.106209
H	-3.186831	-4.817394	1.833361	H	0.274860	-2.389955	1.194030
H	-6.109956	-1.720207	1.222719	H	-1.689129	-1.839699	1.243658
C	3.154294	-0.199386	-3.829896	C	-1.672099	-0.280927	2.649352
C	1.501864	-1.934764	-3.340605	C	-1.527095	1.067672	3.037438
C	0.785359	0.396136	-3.611223	C	-2.256312	-1.181482	3.571380
C	2.122449	0.741406	-3.861415	C	-1.943494	1.492409	4.298594
C	2.814924	-1.540092	-3.606706	H	-1.087510	1.776653	2.337402
C	1.207809	-3.396874	-3.101508	C	-2.669155	-0.755984	4.833487

H	-2.388955	-2.226295	3.279964	H	2.487747	1.369525	-0.483535
C	-2.512786	0.585540	5.201122	O	3.096512	-3.080380	-0.323264
H	-1.825360	2.541952	4.579176	O	4.888140	2.080332	0.530998
H	-3.115367	-1.469658	5.530721	C	3.912163	-4.184190	-0.000105
H	-2.837702	0.924931	6.188093	H	4.156230	-4.212679	1.077063
Cl	-4.535823	3.579803	-1.627598	C	4.047642	3.186824	0.253240
Cl	0.928189	5.279247	0.987784	H	3.797148	3.251363	-0.821195
C	3.169954	0.551175	-0.273481	H	3.105644	3.148188	0.823853
C	2.664664	-0.749829	-0.340927	H	4.854713	-4.183081	-0.577124
C	3.582264	-1.811358	-0.148009	H	4.611220	4.084731	0.540804
C	4.835010	-1.545947	0.410496	H	3.337729	-5.084375	-0.258817
C	5.218784	-0.241311	0.758052	H	2.035227	-3.865863	-2.552167
C	4.438498	0.822519	0.265551	H	0.291754	-3.551121	-2.517432
C	6.117102	-0.053492	1.945426	H	-0.196440	2.173333	-2.873631
H	7.173106	0.216983	1.827857	H	-0.128364	2.043361	-4.644967
C	3.635148	-1.829352	3.280339	H	-6.151023	-0.879692	-1.290783
C	4.133845	-0.514473	3.323908	H	-5.126416	0.290218	-0.429236
C	3.209561	0.525454	3.133776	H	-1.362285	-5.094652	0.382360
H	3.544148	1.562431	3.218592	H	-1.651100	-4.632892	-1.309552
C	1.940037	0.269127	2.612416	C	4.596274	0.216822	-3.961417
C	1.567205	-1.035230	2.262679	H	5.200264	-0.551242	-4.468888
C	2.363445	-2.087655	2.760840	H	4.702101	1.164098	-4.511529
H	2.067824	-3.124267	2.575192	H	5.030094	0.366387	-2.956607
C	5.599359	-0.220134	3.184018	C	-5.423319	-3.714459	2.957974
H	6.242232	-0.106423	4.065374	H	-5.275456	-4.783011	3.176393
H	1.310730	1.108671	2.324133	H	-5.026992	-3.144712	3.817068
H	4.303130	-2.668504	3.493393	H	-6.504592	-3.515852	2.907833
H	5.447556	-2.368109	0.778810				

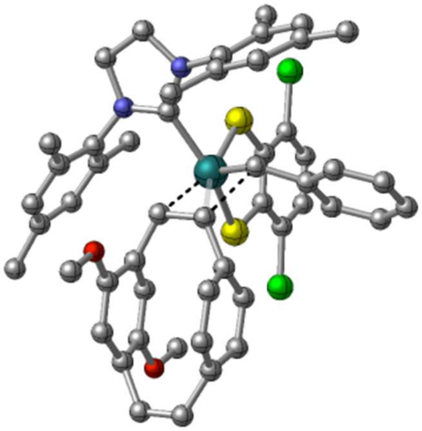

Zero-point correction= 0.887295 (Hartree/Particle)

Thermal correction to Energy= 0.945645

Thermal correction to Enthalpy= 0.946589

Thermal correction to Gibbs Free Energy= 0.792950

Sum of electronic and zero-point Energies= -4080.449606

Sum of electronic and thermal Energies= -4080.391256

Sum of electronic and thermal Enthalpies= -4080.390312

Sum of electronic and thermal Free Energies= -4080.543951

TS1-β (Ru-1b/OMe)

E(scf) = -4081.33536713 a.u.

ν_{min} = -71.9490 cm^{-1}

S	2.501193	1.092037	0.972325	C	-0.032550	-1.384630	3.439765
C	1.473219	-1.588252	1.449642	H	1.681755	-3.439947	4.007435
Ru	0.560489	-0.001679	0.384586	H	2.669148	-1.973773	4.223764
C	-1.319407	-1.091726	0.585078	H	3.024584	-4.141969	2.146515
C	0.952000	-0.653744	-1.373681	C	4.095019	-3.139164	-2.686565
S	-0.375768	2.204038	0.232331	C	4.301035	-1.932593	-0.559156
N	2.604066	-2.292300	1.194420	C	2.496433	-3.565598	-0.885047
N	1.158267	-1.802374	2.754619	C	2.984057	-3.824310	-2.169005
C	3.141371	-2.580960	-0.100858	C	4.745917	-2.219140	-1.860333
C	3.096714	-3.061376	2.352496	H	2.475537	-4.568036	-2.789264
C	2.160422	-2.604365	3.476305	H	5.632407	-1.697755	-2.233788

C	-2.460141	-0.593014	4.625380	C	1.636978	-0.152582	-5.033932
C	-1.095372	-2.312768	3.547679	H	1.507457	-1.817425	-3.668611
C	-0.134892	-0.104406	4.016043	C	1.552699	1.237283	-5.178046
C	-1.362783	0.269904	4.584761	H	1.202446	3.121870	-4.170946
C	-2.292892	-1.894070	4.131067	H	1.866042	-0.782746	-5.896989
C	-0.971523	-3.731295	3.042636	H	1.717284	1.697981	-6.155552
C	1.011894	0.872411	4.063985	Cl	4.933180	3.144990	1.307565
H	-1.456571	1.276719	5.002710	Cl	-0.747501	5.383099	-0.208203
H	-3.131896	-2.594347	4.174951	C	-3.161658	0.689614	0.989943
H	4.153215	-2.825974	2.548019	C	-2.717922	-0.637309	0.908584
H	-0.614136	-4.404143	3.841586	C	-3.714235	-1.630772	0.797478
H	1.968399	0.412391	3.791622	C	-5.016893	-1.295477	0.426726
C	5.093524	-0.970180	0.288976	C	-5.365728	0.044533	0.177909
H	4.681104	-0.859469	1.298051	C	-4.468938	1.028796	0.624591
C	1.282409	-4.295779	-0.363275	C	-6.406754	0.382227	-0.849162
H	0.466725	-3.600560	-0.113863	H	-7.433645	0.636841	-0.559625
C	2.256128	2.824976	0.751804	C	-4.245446	-1.284478	-2.596781
C	3.326010	3.732183	0.898837	C	-4.635200	0.061323	-2.520719
C	0.972274	3.319874	0.413512	C	-3.623198	1.034345	-2.397074
C	3.150138	5.102257	0.719275	C	-2.326192	0.633573	-2.042415
C	0.817925	4.712509	0.230765	C	-2.008299	-0.712654	-1.844941
C	1.885648	5.594840	0.382055	C	-2.942114	-1.683749	-2.280512
H	3.998014	5.778362	0.836537	C	-6.049980	0.404463	-2.153122
H	1.729433	6.663740	0.230494	H	-6.785648	0.683905	-2.916655
H	-1.092671	-2.026573	1.095592	H	-5.716801	-2.092427	0.160739
C	-0.989477	-1.200335	-0.860368	H	-2.445423	1.484189	1.190591
H	-0.642855	-2.202940	-1.106799	H	-3.423348	-2.685164	0.815149
H	1.277878	-1.687689	-1.497655	H	-4.728459	2.084812	0.513477
C	1.125891	0.056474	-2.649216	O	-2.564747	-2.994799	-2.159677
C	1.050372	1.455574	-2.815061	O	-4.020971	2.333291	-2.437218
C	1.430850	-0.733492	-3.783313	H	-5.027926	-2.034329	-2.709563
C	1.262301	2.035857	-4.065064	H	-1.600928	1.380546	-1.731975
H	0.821709	2.081020	-1.955101	C	-3.511109	-4.012535	-2.400526

H	-3.913805	-3.965678	-3.428547	H	5.104265	0.035005	-0.158080
H	-4.354104	-3.965926	-1.687360	H	0.899905	-5.010271	-1.104764
C	-3.084119	3.352562	-2.132573	H	1.508224	-4.855085	0.559031
H	-2.220627	3.334535	-2.821606	C	-3.800935	-0.131742	5.136300
H	-2.701575	3.271359	-1.103167	H	-4.344843	-0.942419	5.645475
H	-2.985475	-4.967975	-2.267705	H	-3.704879	0.714817	5.832712
H	-3.614520	4.306925	-2.251084	H	-4.428634	0.203492	4.291828
H	0.849196	1.702145	3.358958	C	4.546582	-3.362406	-4.107933
H	1.097555	1.302819	5.073912	H	4.312585	-4.380212	-4.455406
H	-1.946363	-4.115070	2.707963	H	4.033738	-2.656996	-4.785488
H	-0.265894	-3.821178	2.206149	H	5.628238	-3.195323	-4.222798
H	6.138442	-1.312195	0.368103				

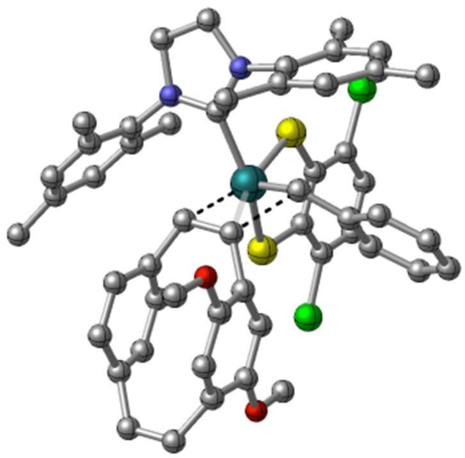

Zero-point correction= 0.887024 (Hartree/Particle)

Thermal correction to Energy= 0.945549

Thermal correction to Enthalpy= 0.946493

Thermal correction to Gibbs Free Energy= 0.791996

Sum of electronic and zero-point Energies= -4080.448344

Sum of electronic and thermal Energies= -4080.389818

Sum of electronic and thermal Enthalpies= -4080.388874

Sum of electronic and thermal Free Energies= -4080.543371

Int1-β (Ru-1b/OMe)

E(scf) = -4081.40840076 a.u.

ν_{min} = 10.3854 cm^{-1}

S	1.468519	0.445348	1.358747	H	6.467666	0.316490	0.986007
C	3.877206	-0.590511	-0.392696	H	5.172190	-2.841539	-2.728501
Ru	1.922836	-0.137329	-0.801816	H	4.763784	-2.728540	2.556849
C	1.111215	-1.761306	-0.989742	C	4.362051	2.035332	2.113739
C	-5.991841	1.710324	-2.006584	H	5.337714	2.382017	2.498122
S	0.630159	1.665110	-1.486596	C	4.602047	1.497240	-2.942026
N	4.775491	0.410510	-0.288349	H	3.780160	0.788552	-3.145826
N	4.518229	-1.762247	-0.223685	C	0.546127	1.951328	1.265911
C	4.418290	1.792731	-0.426573	C	0.187250	2.644850	2.438175
C	6.136749	-0.060764	0.005305	C	0.171298	2.495078	0.011614
C	5.969914	-1.593822	-0.019280	C	-0.507190	3.851816	2.397955
C	3.895198	-3.047821	-0.207194	C	-0.520546	3.721658	-0.008843
H	6.525698	-2.069420	-0.843136	C	-0.858636	4.399445	1.161945
H	6.284048	-2.072606	0.919874	H	-0.761800	4.362794	3.327435
H	6.842891	0.305061	-0.756001	H	-1.386924	5.351198	1.107540
C	3.425299	4.395222	-0.741334	H	1.182458	-2.014799	-2.078165
C	4.132318	2.560104	0.719437	C	-6.138662	0.366768	-2.049355
C	4.292934	2.331741	-1.724346	H	-6.618157	-0.050612	-2.940319
C	3.800008	3.632162	-1.856447	H	-6.271042	2.261698	-2.912501
C	3.624217	3.852150	0.533142	C	-5.506943	2.555600	-0.894390
H	3.666990	4.048663	-2.859021	C	-4.667998	3.653473	-1.162923
H	3.349051	4.438626	1.414281	C	-5.886083	2.320113	0.442965
C	2.567773	-5.513229	-0.177745	C	-4.194363	4.466136	-0.129695
C	3.675057	-3.720422	-1.421933	H	-4.361340	3.856507	-2.192505
C	3.483841	-3.588268	1.024112	C	-5.416978	3.133956	1.474339
C	2.828800	-4.824639	1.014527	H	-6.550745	1.483995	0.668150
C	3.007691	-4.950935	-1.384000	C	-4.562661	4.207867	1.193573
C	4.100067	-3.095670	-2.726300	H	-3.522004	5.293346	-0.364263
C	3.694086	-2.821470	2.305330	H	-5.720672	2.930614	2.504676
H	2.480668	-5.245606	1.961872	H	-4.186410	4.838757	2.002643
H	2.812591	-5.476551	-2.323253	Cl	0.643963	1.993498	4.008386

Cl	-0.950486	4.465414	-1.545398	C	-8.326772	-3.143959	-1.604894
C	0.189538	-2.723500	1.149427	H	-8.744090	-3.308860	-0.594692
C	0.145370	-2.561027	-0.251630	H	-7.732159	-4.031250	-1.890104
C	-0.942369	-3.129993	-0.950119	C	-2.229513	-0.092197	1.279922
C	-2.003336	-3.712944	-0.264889	H	-2.780028	0.847993	1.459327
C	-1.994387	-3.790642	1.139837	H	-1.780409	-0.062598	0.272443
C	-0.845768	-3.353536	1.826940	H	-9.155933	-3.027664	-2.315927
C	-3.194032	-4.222417	1.879505	H	-1.417061	-0.183230	2.009767
H	-3.056102	-4.976450	2.663502	H	3.296111	-1.797239	2.223523
C	-6.011291	-2.804346	0.003842	H	3.195361	-3.315851	3.150405
C	-4.842067	-2.639404	0.769421	H	3.907373	-3.767888	-3.573991
C	-4.126222	-1.419451	0.643121	H	3.554096	-2.154540	-2.906872
C	-4.551732	-0.465971	-0.285259	H	3.581645	2.392591	2.800453
C	-5.710302	-0.635720	-1.060536	H	4.347114	0.939079	2.148540
C	-6.447198	-1.834027	-0.905028	H	4.723307	2.127685	-3.834152
C	-4.435570	-3.728737	1.683399	H	5.515596	0.896882	-2.813729
H	-5.253958	-4.186948	2.252409	C	1.772560	-6.794762	-0.161118
H	-2.872014	-4.081114	-0.814249	H	1.949006	-7.398194	-1.064151
H	1.031328	-2.314454	1.702147	H	2.012392	-7.409929	0.719947
H	-0.977484	-3.053732	-2.040502	H	0.692320	-6.570637	-0.117181
H	-0.809786	-3.434299	2.916531	C	2.770024	5.740854	-0.922525
O	-7.567913	-1.956866	-1.672514	H	3.243886	6.317224	-1.732719
O	-3.057036	-1.230460	1.452618	H	1.708767	5.606898	-1.194148
H	-6.568054	-3.733957	0.123744	H	2.804901	6.340913	-0.001133
H	-3.978953	0.448591	-0.415527				

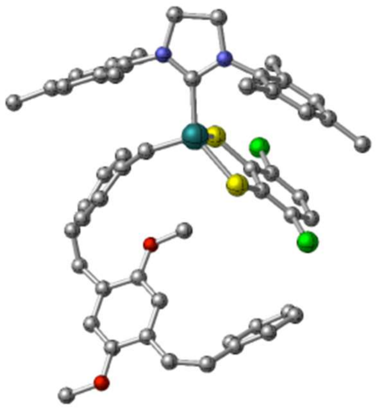

Zero-point correction= 0.885762 (Hartree/Particle)

Thermal correction to Energy= 0.946257

Thermal correction to Enthalpy= 0.947201

Thermal correction to Gibbs Free Energy= 0.784747

Sum of electronic and zero-point Energies= -4080.522639

Sum of electronic and thermal Energies= -4080.462144

Sum of electronic and thermal Enthalpies= -4080.461200

Sum of electronic and thermal Free Energies= -4080.623654

Ru-1b

E(scf) = -3236.00924284 a.u.

ν_{min} = 5.3476 cm^{-1}

S	-0.317820	1.006139	1.140633	H	-0.380370	-3.963621	2.638929
C	0.425583	-1.896133	0.431699	H	2.323827	-4.149378	1.462729
Ru	-0.241894	-0.311499	-0.718873	C	5.276316	-0.124986	0.399053
C	1.252160	0.154365	-1.656577	C	3.238706	-0.519441	1.702970
S	-2.108769	0.829673	-1.501525	C	3.654842	-1.792032	-0.357165
N	1.630358	-2.209101	0.936761	C	4.857749	-1.102240	-0.517932
N	-0.467446	-2.813827	0.849918	C	4.456843	0.146460	1.497952
C	2.848358	-1.481524	0.757654	H	5.484461	-1.323803	-1.386437
C	1.592055	-3.402265	1.805287	H	4.764240	0.908653	2.220035
C	0.133767	-3.878285	1.670427	C	-4.490488	-2.800341	-0.475534
C	-1.833593	-2.836949	0.417111	C	-2.141491	-3.373995	-0.849401
H	0.046627	-4.847997	1.154025	C	-2.831486	-2.308291	1.258846

C	-4.150504	-2.299941	0.788400	H	1.849234	2.066460	0.217601
C	-3.474187	-3.343944	-1.274091	C	4.287298	2.049482	-2.816125
C	-1.055523	-3.917449	-1.745047	H	3.100166	0.412002	-3.565237
C	-2.480209	-1.724998	2.602195	C	4.462091	2.981168	-1.785959
H	-4.933439	-1.877921	1.424782	H	3.719153	3.704742	0.114160
H	-3.724919	-3.747281	-2.259438	H	4.970468	2.039344	-3.669237
H	1.853555	-3.119191	2.837254	H	5.280864	3.703789	-1.832767
H	-0.354698	-4.568324	-1.200269	Cl	-0.783068	3.404705	3.179092
H	-1.969697	-2.457605	3.248014	Cl	-4.305590	3.138119	-2.022474
C	2.408581	-0.192847	2.919409	H	-1.479819	-4.491257	-2.580699
H	1.417582	-0.662064	2.892335	H	-0.453388	-3.098837	-2.175539
C	3.207587	-2.829309	-1.355833	H	-1.799705	-0.865414	2.481481
H	3.015907	-3.802724	-0.875339	H	-3.379638	-1.379690	3.130848
C	-1.542148	2.244054	0.808443	H	2.921887	-0.520207	3.839208
C	-1.751294	3.303545	1.713388	H	2.248344	0.892894	2.999162
C	-2.329599	2.177528	-0.366652	H	2.266351	-2.526925	-1.841205
C	-2.712849	4.284303	1.480823	H	3.963737	-2.980366	-2.138863
C	-3.298304	3.178816	-0.584232	C	-5.909464	-2.719764	-0.980735
C	-3.492287	4.220811	0.322659	H	-6.131467	-3.522611	-1.700144
H	-2.847809	5.093062	2.200358	H	-6.637657	-2.779231	-0.157592
H	-4.249119	4.979612	0.119725	H	-6.077937	-1.759210	-1.497893
H	1.207881	-0.465121	-2.590977	C	6.558522	0.634530	0.171935
C	2.336255	1.126519	-1.664638	H	7.362625	-0.028037	-0.185314
C	2.535529	2.067047	-0.629651	H	6.406674	1.410599	-0.596768
C	3.235782	1.135901	-2.756911	H	6.904006	1.135914	1.088272
C	3.583339	2.982737	-0.695063				

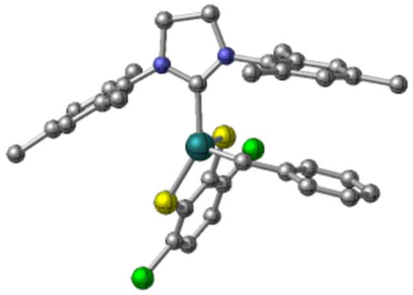

Zero-point correction= 0.591781 (Hartree/Particle)

Thermal correction to Energy= 0.632982

Thermal correction to Enthalpy= 0.633926

Thermal correction to Gibbs Free Energy= 0.515360

Sum of electronic and zero-point Energies= -3235.417461

Sum of electronic and thermal Energies= -3235.376261

Sum of electronic and thermal Enthalpies= -3235.375317

Sum of electronic and thermal Free Energies= -3235.493882

Ru-1a-M' (OMe)

E(scf) = -4317.07705877 a.u.

ν_{min} = 13.0523 cm^{-1}

S	-2.400879	0.885299	-0.878656	H	-1.584596	-3.648940	4.355666
C	-1.169431	-1.687427	-0.629673	H	-5.118730	-1.395294	3.444843
Ru	-0.314772	0.192819	-0.102341	C	2.330675	-1.945737	-4.378036
C	1.962557	-0.443264	-0.552095	C	1.091064	-3.097691	-2.631333
C	-0.687401	0.059718	1.737133	C	0.136053	-1.102605	-3.733420
S	0.305181	2.459563	-0.320629	C	1.292962	-1.026675	-4.524353
N	-2.211316	-2.316381	-0.020998	C	2.219381	-2.983896	-3.453170
N	-1.055555	-2.217025	-1.874980	C	0.977999	-4.269799	-1.662070
C	-2.575625	-2.318396	1.370620	C	-1.026610	-0.157165	-3.991233
C	-2.836714	-3.346945	-0.877109	H	1.376047	-0.237338	-5.274268
C	-2.178108	-3.099023	-2.231820	H	3.028772	-3.710523	-3.359750
C	0.073434	-2.118526	-2.755887	H	-3.925735	-3.217848	-0.903061
H	-1.818067	-4.016776	-2.714972	H	0.112698	-4.087984	-1.011618
H	-2.844532	-2.575725	-2.937672	H	-1.737868	-0.270401	-3.163804
H	-2.622578	-4.348915	-0.473371	C	-4.754937	-1.054179	0.797648
C	-3.386719	-2.519292	4.040710	H	-4.234804	-0.945423	-0.162803
C	-3.803987	-1.740320	1.769652	C	-0.497125	-3.769511	1.884421
C	-1.766721	-3.031162	2.295601	H	-0.161956	-3.352994	0.926071
C	-2.193124	-3.109258	3.627898	C	-2.328163	2.623640	-1.141509
C	-4.180857	-1.847625	3.116894	C	-3.450310	3.339572	-1.609048

C	-1.126427	3.327372	-0.879001	C	-0.784221	-5.266760	1.662750
C	-3.403298	4.715674	-1.820424	H	-1.121985	-5.737967	2.599872
C	-1.103864	4.723402	-1.087773	H	-1.567856	-5.429157	0.908566
C	-2.221682	5.413237	-1.553772	H	0.123663	-5.791496	1.325520
H	-4.285906	5.237825	-2.192138	C	0.661794	-3.597955	2.879425
H	-2.166072	6.491446	-1.708935	H	0.473837	-4.133018	3.823199
H	1.714361	-1.103839	-1.380159	H	1.586967	-4.011482	2.449241
C	1.626732	-0.889174	0.736745	H	0.848762	-2.541936	3.121500
H	1.198214	-1.885566	0.828471	C	0.713532	-5.583850	-2.419673
H	-0.270755	-0.790978	2.290313	H	0.567921	-6.415569	-1.711313
C	-1.437658	0.910345	2.650381	H	-0.181528	-5.517186	-3.057540
C	-2.007403	2.162475	2.322189	H	1.565511	-5.838089	-3.071026
C	-1.591746	0.444788	3.981514	C	2.204346	-4.411321	-0.748257
C	-2.708785	2.899828	3.273987	H	3.088066	-4.737016	-1.318745
H	-1.896931	2.557512	1.319781	H	2.459033	-3.461858	-0.259388
C	-2.301745	1.177849	4.928753	H	2.017424	-5.173327	0.024486
H	-1.156073	-0.517398	4.257118	C	-1.753039	-0.550315	-5.290407
C	-2.865693	2.410346	4.576460	H	-2.634421	0.092990	-5.445949
H	-3.140369	3.864183	2.995054	H	-1.093088	-0.439220	-6.166833
H	-2.416907	0.789955	5.943874	H	-2.094915	-1.597676	-5.260777
H	-3.421661	2.990859	5.317328	C	-0.606613	1.319204	-4.010724
H	-3.698367	-2.587161	5.085976	H	-1.496611	1.963446	-4.081645
H	3.226684	-1.860854	-4.997943	H	-0.072822	1.591682	-3.088764
Cl	-4.950284	2.494693	-1.971680	H	0.043720	1.550464	-4.869852
Cl	0.355988	5.647875	-0.764255	C	3.419900	1.721340	-0.595540
C	-6.016564	-1.907997	0.569016	C	3.249638	0.337931	-0.705382
H	-6.600130	-2.009163	1.498840	C	4.416106	-0.460294	-0.591779
H	-6.666295	-1.436255	-0.185839	C	5.581074	0.096476	-0.055955
H	-5.773466	-2.925277	0.222855	C	5.640474	1.440468	0.340685
C	-5.138944	0.360716	1.259267	C	4.600113	2.286575	-0.083188
H	-4.245515	0.957795	1.482070	C	6.514259	1.803028	1.505680
H	-5.693066	0.880429	0.463042	H	7.457915	2.343096	1.365305
H	-5.775784	0.336290	2.158604	C	4.657307	-0.624913	2.859583

C	4.791021	0.774729	2.923209	H	2.549482	2.353358	-0.732201
C	3.623274	1.539616	2.768433	O	4.268560	-1.801844	-0.812709
H	3.680013	2.626605	2.865110	O	4.718622	3.606858	0.229529
C	2.456763	0.966711	2.258774	C	5.371517	-2.655406	-0.602185
C	2.433686	-0.387871	1.892270	H	5.680267	-2.674309	0.458440
C	3.487827	-1.202134	2.354517	C	3.608279	4.462291	0.027139
H	3.470278	-2.274984	2.146130	H	3.307432	4.506065	-1.034259
C	6.120247	1.450245	2.751135	H	2.729371	4.149504	0.615716
H	6.757486	1.682449	3.612949	H	6.239422	-2.362699	-1.219904
H	1.622977	1.613778	1.983192	H	3.923731	5.463761	0.349749
H	5.529157	-1.259947	3.040105	H	5.049021	-3.662496	-0.894598
H	6.394983	-0.557697	0.253811				

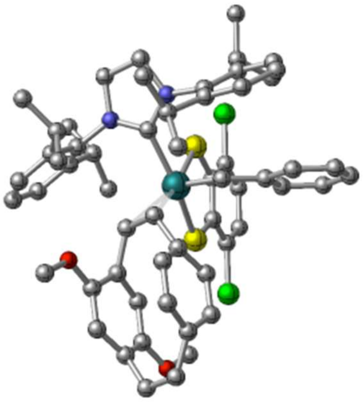

Zero-point correction= 1.060598 (Hartree/Particle)

Thermal correction to Energy= 1.126195

Thermal correction to Enthalpy= 1.127139

Thermal correction to Gibbs Free Energy= 0.961516

Sum of electronic and zero-point Energies= -4316.016460

Sum of electronic and thermal Energies= -4315.950864

Sum of electronic and thermal Enthalpies= -4315.949920

Sum of electronic and thermal Free Energies= -4316.115543

Ru-1a-M (OMe)

E(scf) = -4317.07783951 a.u.

ν_{min} = 11.1103 cm^{-1}

S	-2.516760	0.656659	-0.788549	H	0.512326	-4.086785	-0.885173
C	-1.049548	-1.806434	-0.519808	H	-2.055612	-0.691923	-3.040248
Ru	-0.318397	0.179760	-0.195477	C	-4.534235	-1.331469	1.226097
C	1.897151	-0.340764	-0.868949	H	-4.118144	-1.236422	0.214959
C	-0.478203	0.124311	1.683001	C	0.048238	-3.582769	2.029018
S	0.092579	2.458977	-0.611532	H	0.284382	-3.125187	1.059645
N	-1.982410	-2.457716	0.226257	C	-2.617812	2.379589	-1.134217
N	-0.989130	-2.434686	-1.721376	C	-3.837214	2.985242	-1.504383
C	-2.213088	-2.390079	1.644546	C	-1.457175	3.185805	-1.033448
C	-2.640785	-3.563011	-0.502142	C	-3.925056	4.350762	-1.765034
C	-2.011045	-3.479921	-1.893234	C	-1.569396	4.569987	-1.288312
C	0.008378	-2.283599	-2.742827	C	-2.783300	5.150166	-1.650262
H	-1.549022	-4.423259	-2.217218	H	-4.881715	4.786488	-2.056229
H	-2.728819	-3.168266	-2.668643	H	-2.834132	6.222249	-1.844737
H	-2.444944	-4.519653	0.004701	H	1.616981	-1.132949	-1.558785
C	-2.757744	-2.508294	4.386348	C	1.773428	-0.623196	0.504762
C	-3.445198	-1.893294	2.130650	H	1.464518	-1.628039	0.779888
C	-1.257866	-2.968641	2.523149	H	0.056559	-0.659609	2.232213
C	-1.554576	-3.009645	3.891529	C	-1.193727	0.974137	2.623974
C	-3.688173	-1.957902	3.511412	C	-1.884187	2.162631	2.291040
H	-0.833537	-3.445693	4.584610	C	-1.180340	0.578736	3.986375
H	-4.629354	-1.568750	3.904619	C	-2.538816	2.906791	3.270361
C	1.996801	-2.013034	-4.677681	H	-1.903374	2.503027	1.263043
C	1.148302	-3.125800	-2.686125	C	-1.844227	1.317528	4.962138
C	-0.178047	-1.358356	-3.792362	H	-0.650609	-0.333990	4.265437
C	0.847415	-1.228310	-4.742134	C	-2.528528	2.486400	4.606007
C	2.134649	-2.966182	-3.668365	H	-3.065677	3.821369	2.987476
C	1.324048	-4.199635	-1.615907	H	-1.829541	0.983783	6.002599
C	-1.463787	-0.562543	-3.954806	H	-3.048702	3.071286	5.369087
H	0.735544	-0.505050	-5.552501	H	-2.967518	-2.546888	5.458204
H	3.028887	-3.592451	-3.639295	H	2.784780	-1.891156	-5.424976
H	-3.727780	-3.410821	-0.523317	Cl	-5.291955	2.010982	-1.675938

Cl	-0.158435	5.608952	-1.156535	C	3.168989	1.892194	-1.378166
C	-5.744489	-2.282056	1.163508	C	3.073554	0.495325	-1.318816
H	-6.229116	-2.372158	2.149571	C	4.290454	-0.221843	-1.346416
H	-6.495777	-1.899249	0.453891	C	5.499942	0.420174	-1.075092
H	-5.458762	-3.297166	0.844748	C	5.530414	1.797691	-0.791716
C	-4.975152	0.076763	1.657120	C	4.383257	2.536760	-1.117615
H	-4.110767	0.745929	1.756732	C	6.534922	2.363040	0.169342
H	-5.644150	0.511872	0.899343	H	7.431268	2.887798	-0.182995
H	-5.514132	0.057957	2.618378	C	5.067318	0.131244	1.999892
C	-0.101749	-5.097270	1.795138	C	5.060501	1.534043	1.953167
H	-0.354800	-5.611207	2.736546	C	3.811827	2.189069	1.943088
H	-0.892350	-5.327083	1.066527	C	2.648778	1.446404	1.687945
H	0.839999	-5.524130	1.415192	C	2.701296	0.067431	1.454240
C	1.241560	-3.306606	2.956169	C	3.904844	-0.609099	1.756957
H	1.169229	-3.875596	3.896330	C	6.295144	2.256789	1.495897
H	2.178740	-3.591644	2.459519	H	6.995212	2.698474	2.214969
H	1.325454	-2.239267	3.209517	H	6.395646	-0.184734	-0.908974
C	1.197918	-5.605996	-2.230099	H	2.267002	2.492727	-1.466427
H	1.240759	-6.375998	-1.442870	H	4.267182	-1.314198	-1.386212
H	0.250921	-5.729407	-2.778585	H	4.382715	3.621211	-0.981456
H	2.018716	-5.801296	-2.939141	O	3.902089	-1.964064	1.562897
C	2.644689	-4.062517	-0.840752	O	3.835200	3.547949	1.983659
H	3.516956	-4.200250	-1.499578	H	6.034317	-0.370946	2.014734
H	2.740768	-3.084478	-0.352223	H	1.715792	1.962583	1.472637
H	2.703783	-4.833679	-0.055911	C	5.116601	-2.678981	1.609296
C	-2.290032	-1.115505	-5.130305	H	5.604476	-2.599314	2.597367
H	-3.250763	-0.580908	-5.207194	H	5.824377	-2.334437	0.833995
H	-1.756605	-0.992446	-6.087432	C	2.628822	4.266595	1.792693
H	-2.504970	-2.189090	-5.004436	H	1.878441	4.016651	2.564403
C	-1.215567	0.945066	-4.109203	H	2.183370	4.078641	0.801565
H	-2.173801	1.487097	-4.103068	H	4.864922	-3.731273	1.419254
H	-0.609448	1.335134	-3.278782	H	2.886174	5.330848	1.877374
H	-0.701902	1.181402	-5.055089				

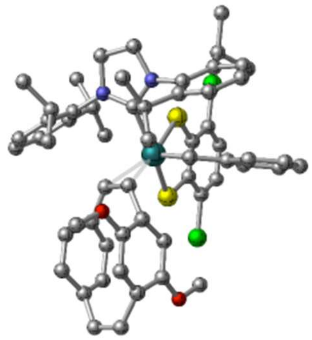

Zero-point correction= 1.060452 (Hartree/Particle)

Thermal correction to Energy= 1.126278

Thermal correction to Enthalpy= 1.127222

Thermal correction to Gibbs Free Energy= 0.959816

Sum of electronic and zero-point Energies= -4316.017387

Sum of electronic and thermal Energies= -4315.951561

Sum of electronic and thermal Enthalpies= -4315.950617

Sum of electronic and thermal Free Energies= -4316.118023

TS1-α (Ru-1a/OMe)

E(scf) = -4317.06734726 a.u.

ν_{min} = -179.8563 cm^{-1}

S	-2.230085	1.133836	-1.012426	C	-0.247188	-2.276708	-2.587871
C	-1.486675	-1.552985	-0.536545	H	-2.446393	-3.838188	-2.511247
Ru	-0.372507	0.210895	-0.056888	H	-3.138563	-2.266422	-2.980518
C	1.495382	-0.827223	-0.256535	H	-3.631066	-3.785342	-0.424156
C	-0.507890	-0.039495	1.836920	C	-3.858436	-1.777434	4.087448
S	0.531292	2.433061	-0.190192	C	-4.110790	-1.005594	1.788983
N	-2.660669	-1.948227	0.026860	C	-2.402487	-2.691168	2.361503
N	-1.417209	-2.132538	-1.763565	C	-2.823774	-2.626921	3.695521
C	-3.047881	-1.855398	1.409305	C	-4.494321	-0.982718	3.140083
C	-3.483642	-2.788929	-0.867050	H	-2.335994	-3.251373	4.445302
C	-2.658569	-2.819616	-2.157201	H	-5.308319	-0.324706	3.450857

C	2.082282	-2.589259	-4.089195	C	-0.101003	1.545802	5.239765
C	0.556897	-3.426835	-2.389758	H	0.337555	-0.371765	4.353666
C	0.056637	-1.328732	-3.588138	C	-0.632517	2.824905	5.034787
C	1.243980	-1.500063	-4.316717	H	-1.566943	4.165733	3.614494
C	1.727683	-3.554510	-3.147443	H	0.283770	1.261462	6.222350
C	0.162951	-4.541738	-1.424928	H	-0.666716	3.542521	5.858552
C	-0.890712	-0.196080	-3.950321	H	-4.168712	-1.739697	5.134634
H	1.509768	-0.768136	-5.082385	H	3.008189	-2.695907	-4.659898
H	2.375312	-4.420310	-2.994277	Cl	-4.545253	2.994994	-2.213288
H	-4.474002	-2.336003	-1.011194	Cl	0.908970	5.622747	-0.502203
H	-0.718579	-4.209983	-0.862181	C	-6.306058	-0.658063	0.591620
H	-1.676134	-0.162350	-3.186099	H	-6.892238	-0.599472	1.523478
C	-4.873372	-0.135026	0.800515	H	-6.825656	-0.055668	-0.171045
H	-4.350556	-0.181254	-0.162632	H	-6.315159	-1.709362	0.261866
C	-1.291001	-3.666138	1.978535	C	-4.886792	1.342634	1.226034
H	-0.803817	-3.280751	1.072437	H	-3.868698	1.701091	1.433977
C	-1.992389	2.872321	-1.187433	H	-5.299956	1.964000	0.417329
C	-3.011364	3.694847	-1.707324	H	-5.500887	1.501758	2.127182
C	-0.757371	3.448507	-0.802804	C	-1.876339	-5.051335	1.645506
C	-2.833944	5.068620	-1.853088	H	-2.401368	-5.464901	2.521553
C	-0.599129	4.845350	-0.961072	H	-2.596570	-5.005779	0.816681
C	-1.617011	5.645161	-1.474428	H	-1.080020	-5.755993	1.361212
H	-3.638954	5.681398	-2.260388	C	-0.195119	-3.803556	3.048546
H	-1.457966	6.719378	-1.578476	H	-0.542520	-4.375944	3.922753
H	1.260185	-1.813044	-0.650335	H	0.669935	-4.339579	2.629497
C	1.288234	-0.792700	1.232316	H	0.153702	-2.824801	3.410986
H	0.995116	-1.778918	1.596940	C	-0.238413	-5.811875	-2.197261
H	-0.832345	-0.999477	2.242407	H	-0.602274	-6.587485	-1.503776
C	-0.531410	0.983574	2.904150	H	-1.033282	-5.608634	-2.931966
C	-1.073390	2.269400	2.716095	H	0.621721	-6.226580	-2.747488
C	-0.069819	0.628817	4.189397	C	1.255515	-4.857985	-0.394582
C	-1.131575	3.176510	3.776056	H	2.162213	-5.244714	-0.884182
H	-1.472848	2.541686	1.740991	H	1.545230	-3.965600	0.175779

H	0.906670	-5.630906	0.307336	C	2.683039	1.190867	2.030009
C	-1.571491	-0.480999	-5.301495	C	2.389003	-0.178948	2.046199
H	-2.307776	0.305952	-5.533035	C	3.374881	-1.046838	2.554555
H	-0.836642	-0.509365	-6.123038	H	3.156824	-2.113589	2.655752
H	-2.097555	-1.449453	-5.290944	C	6.407100	1.068957	1.846712
C	-0.209416	1.180291	-3.949672	H	7.205552	1.408984	2.517298
H	-0.958223	1.972556	-4.105889	H	1.915609	1.907101	1.741261
H	0.287834	1.380376	-2.989482	H	5.475378	-1.330986	2.940812
H	0.541309	1.265599	-4.751758	H	5.965642	-1.529397	0.071784
C	3.236898	0.920287	-1.079440	H	2.447396	1.631498	-1.303813
C	2.858134	-0.383584	-0.737153	O	3.540341	-2.609098	-0.255868
C	3.903548	-1.314500	-0.512298	O	4.917962	2.686405	-1.037544
C	5.210005	-0.854632	-0.327137	C	4.534348	-3.541540	0.106119
C	5.522440	0.510484	-0.373992	H	5.024815	-3.270774	1.057848
C	4.553138	1.381277	-0.906743	C	3.924737	3.656831	-1.319729
C	6.624235	1.011894	0.512360	H	3.432533	3.471749	-2.291614
H	7.589865	1.329798	0.102129	H	3.145664	3.690647	-0.541947
C	4.692226	-0.602225	2.714216	H	5.307627	-3.640749	-0.676755
C	5.046031	0.715848	2.373211	H	4.439915	4.626004	-1.362629
C	3.996942	1.631055	2.184739	H	4.028575	-4.507021	0.232252
H	4.229516	2.679324	1.981173				

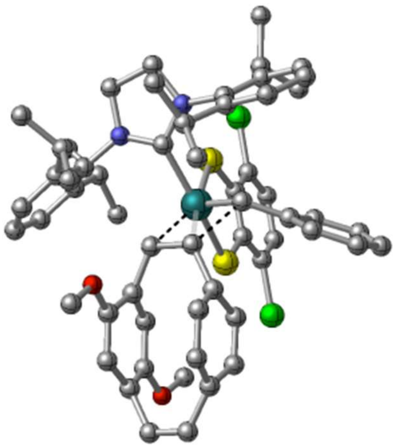

Zero-point correction= 1.060971 (Hartree/Particle)

Thermal correction to Energy= 1.125631

Thermal correction to Enthalpy= 1.126575

Thermal correction to Gibbs Free Energy= 0.962969

Sum of electronic and zero-point Energies= -4316.006376

Sum of electronic and thermal Energies= -4315.941716

Sum of electronic and thermal Enthalpies= -4315.940772

Sum of electronic and thermal Free Energies= -4316.104378

TS1-β (Ru-1a/OMe)

E(scf) = -4317.07019433 a.u.

ν_{min} = -163.2626 cm^{-1}

S	-2.515071	0.858098	-0.841461	C	-0.577926	-1.736786	-3.606888
C	-1.411082	-1.732291	-0.287481	C	0.428666	-1.841000	-4.580339
Ru	-0.436730	0.170356	-0.196169	C	1.517994	-3.599451	-3.340071
C	1.447486	-0.724762	-0.689079	C	0.605918	-4.514219	-1.169777
C	-0.189722	0.124223	1.704367	C	-1.751298	-0.798679	-3.841298
S	0.236784	2.414441	-0.691551	H	0.385130	-1.198985	-5.462596
N	-2.409087	-2.168512	0.528940	H	2.328285	-4.326826	-3.253289
N	-1.472866	-2.475575	-1.423471	H	-4.300466	-2.651764	-0.298657
C	-2.541509	-1.958045	1.945294	H	-0.189217	-4.249432	-0.463097
C	-3.321056	-3.124701	-0.131515	H	-2.347727	-0.773985	-2.921329
C	-2.595835	-3.429681	-1.445573	C	-4.611564	-0.468289	1.559234
C	-0.485591	-2.562794	-2.466815	H	-4.262937	-0.551718	0.522729
H	-2.221270	-4.463561	-1.502450	C	-0.505629	-3.508079	2.299876
H	-3.222515	-3.248131	-2.331181	H	-0.198679	-3.111592	1.321828
H	-3.477712	-4.011701	0.498047	C	-2.468739	2.597380	-1.143628
C	-2.848220	-1.684425	4.712787	C	-3.638922	3.309200	-1.474674
C	-3.599842	-1.172622	2.451304	C	-1.237948	3.291519	-1.042817
C	-1.638219	-2.624064	2.819196	C	-3.617708	4.685318	-1.690026
C	-1.811243	-2.464345	4.199288	C	-1.240747	4.689253	-1.258403
C	-3.730757	-1.051441	3.845009	C	-2.408422	5.379020	-1.574556
H	-1.124598	-2.955216	4.889855	H	-4.539432	5.210346	-1.943527
H	-4.541151	-0.443601	4.252412	H	-2.372498	6.458038	-1.731044
C	1.473195	-2.751763	-4.446803	H	1.224517	-1.751789	-0.963881
C	0.543994	-3.530265	-2.336114	C	1.506360	-0.584542	0.802888

H	1.344117	-1.547590	1.281113	C	0.327939	-5.948353	-1.656737
H	-0.324847	-0.806282	2.258536	H	0.271568	-6.642833	-0.802853
C	-0.087244	1.253340	2.651531	H	-0.620197	-6.011282	-2.213348
C	-0.745503	2.482469	2.454932	H	1.128893	-6.300856	-2.326425
C	0.650709	1.067158	3.840074	C	1.929502	-4.457405	-0.387831
C	-0.642139	3.502267	3.403011	H	2.792033	-4.691063	-1.031781
H	-1.361439	2.621696	1.568990	H	2.105576	-3.472537	0.066062
C	0.777282	2.096414	4.772414	H	1.919133	-5.199761	0.425816
H	1.149796	0.110758	4.014307	C	-2.655252	-1.336189	-4.965566
C	0.132487	3.320171	4.553885	H	-3.537349	-0.687319	-5.090851
H	-1.167717	4.446014	3.237784	H	-2.120706	-1.369323	-5.929260
H	1.375428	1.943814	5.674210	H	-3.009696	-2.356445	-4.745926
H	0.223901	4.126021	5.286700	C	-1.307831	0.644474	-4.125966
H	-2.964177	-1.571914	5.793638	H	-2.185546	1.308453	-4.163381
H	2.250793	-2.810272	-5.212348	H	-0.641963	1.020944	-3.335656
Cl	-5.174103	2.464588	-1.643420	H	-0.782067	0.726882	-5.090848
Cl	0.253346	5.605873	-1.125547	C	2.916109	1.073521	-1.874172
C	-5.994498	-1.137051	1.653243	C	2.649190	-0.240157	-1.461782
H	-6.410092	-1.052918	2.670962	C	3.740021	-1.138066	-1.483861
H	-6.701747	-0.653866	0.959773	C	5.051620	-0.665322	-1.547859
H	-5.947367	-2.208902	1.402490	C	5.310872	0.715116	-1.618329
C	-4.702775	1.034744	1.870518	C	4.229535	1.547111	-1.949849
H	-3.706603	1.500043	1.852672	C	6.540334	1.299272	-0.986538
H	-5.319345	1.539983	1.112112	H	7.423086	1.557657	-1.584030
H	-5.155093	1.220917	2.857975	C	5.106097	-0.148774	1.522556
C	-0.992755	-4.952111	2.081603	C	5.305069	1.184640	1.132994
H	-1.341593	-5.386838	3.032046	C	4.190553	2.049136	1.138696
H	-1.824929	-5.002693	1.365642	C	2.901551	1.506302	1.237734
H	-0.178287	-5.584738	1.695412	C	2.702029	0.128623	1.354874
C	0.745813	-3.500460	3.190558	C	3.819867	-0.685938	1.644454
H	0.582943	-4.046946	4.132682	C	6.530141	1.545185	0.342817
H	1.580038	-3.986702	2.664844	H	7.400914	2.009191	0.820932
H	1.068372	-2.479312	3.440964	H	5.876354	-1.360625	-1.368420

H	2.094565	1.778092	-1.986969	H	5.315874	-2.707097	2.746224
H	3.570087	-2.194776	-1.268092	H	5.244245	-2.929044	0.964483
H	4.395917	2.618601	-2.087625	C	3.329973	4.232359	0.664824
O	3.570017	-2.021532	1.822740	H	2.697493	4.290074	1.568873
O	4.428989	3.355061	0.849026	H	2.693745	3.929674	-0.182500
H	5.971069	-0.811496	1.527072	H	4.207425	-3.924666	2.035885
H	2.035448	2.134138	1.043995	H	3.756421	5.224119	0.463405
C	4.647842	-2.928275	1.894481				

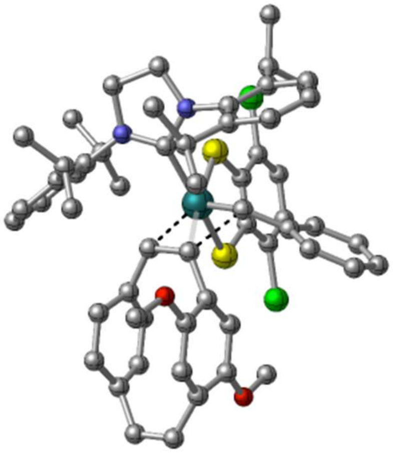

Zero-point correction= 1.061083 (Hartree/Particle)

Thermal correction to Energy= 1.125782

Thermal correction to Enthalpy= 1.126727

Thermal correction to Gibbs Free Energy= 0.963295

Sum of electronic and zero-point Energies= -4316.009111

Sum of electronic and thermal Energies= -4315.944412

Sum of electronic and thermal Enthalpies= -4315.943468

Sum of electronic and thermal Free Energies= -4316.106899

Int1-α (Ru-1a/OMe)

E(scf) = -4317.11684534 a.u.

ν_{min} = 22.1523 cm^{-1}

S	-0.256431	-0.513455	2.278873	Ru	-0.304468	-0.176172	-0.002188
C	-1.880420	1.118034	0.428725	C	0.985803	1.194429	-0.072940

C	-0.828073	-0.605861	-2.546111	H	-3.298380	1.727177	-1.747333
S	0.720218	-2.316840	-0.167120	C	0.587502	-2.035319	2.575528
N	-3.164231	0.652804	0.468501	C	0.833765	-2.475470	3.891589
N	-1.876029	2.303444	1.098037	C	0.998919	-2.850186	1.489833
C	-3.749078	-0.436058	-0.263252	C	1.463368	-3.691252	4.154320
C	-4.010290	1.400094	1.413874	C	1.627342	-4.079889	1.779445
C	-3.206406	2.677663	1.620092	C	1.861262	-4.501298	3.088038
C	-0.900265	3.358479	1.109957	H	1.635908	-4.001451	5.185738
H	-3.605853	3.533720	1.052379	H	2.352677	-5.458084	3.268947
H	-3.134720	2.974174	2.674975	H	0.706896	2.106032	-0.612446
H	-5.012137	1.571993	0.999756	C	0.190839	0.313117	-2.667308
C	-5.118527	-2.452736	-1.630978	H	-0.120489	1.355839	-2.583604
C	-3.965993	-1.679953	0.372159	H	-1.806511	-0.187744	-2.312346
C	-4.233510	-0.184083	-1.574988	C	-0.886128	-2.007938	-3.021031
C	-4.900412	-1.218579	-2.244000	C	-1.752377	-2.918865	-2.390849
C	-4.660064	-2.674840	-0.335809	C	-0.183562	-2.436516	-4.162145
H	-5.271050	-1.053758	-3.257027	C	-1.889512	-4.223511	-2.861128
H	-4.835635	-3.642449	0.138949	H	-2.303389	-2.599924	-1.508810
C	0.610292	5.702709	1.288224	C	-0.318274	-3.744573	-4.634759
C	-0.773347	4.193319	-0.029620	H	0.468058	-1.737417	-4.688876
C	-0.234233	3.663755	2.317341	C	-1.166250	-4.646212	-3.982776
C	0.528727	4.840542	2.377247	H	-2.562330	-4.913688	-2.345752
C	-0.030941	5.372858	0.094810	H	0.238592	-4.058279	-5.521544
C	-1.394922	3.836447	-1.375964	H	-1.268465	-5.670128	-4.351405
C	-0.310372	2.763881	3.541593	H	-5.645568	-3.244061	-2.169561
H	1.054535	5.088615	3.301984	H	1.189169	6.627057	1.361482
H	0.055667	6.043905	-0.761994	Cl	0.318940	-1.495078	5.260774
H	-4.119893	0.825802	2.348425	Cl	2.148517	-5.136352	0.473039
H	-1.602292	2.757225	-1.361769	C	-4.698141	-2.086122	2.753742
H	-0.922337	1.891553	3.276866	H	-5.332091	-2.950697	2.496229
C	-3.502745	-1.964755	1.792833	H	-4.344380	-2.225034	3.788202
H	-2.883234	-1.120573	2.119780	H	-5.336231	-1.188232	2.728112
C	-4.128688	1.193259	-2.226601	C	-2.604420	-3.209665	1.862330

H	-1.765614	-3.129026	1.156955	C	4.504957	2.169233	-0.530140
H	-2.180102	-3.314689	2.872644	C	5.123037	0.922374	-0.393187
H	-3.163939	-4.131173	1.633382	C	4.462351	-0.094016	0.338810
C	-5.411849	2.006080	-1.965759	C	6.240508	0.575556	-1.323101
H	-6.283006	1.504377	-2.417488	H	7.283910	0.563067	-0.989392
H	-5.614687	2.122529	-0.891716	C	3.751182	1.299814	-3.406780
H	-5.330478	3.012926	-2.404470	C	4.476704	0.168131	-2.999299
C	-3.836135	1.154400	-3.734984	C	3.751528	-1.010865	-2.748539
H	-4.695815	0.775529	-4.309891	H	4.286975	-1.912404	-2.440764
H	-3.627699	2.173005	-4.099316	C	2.359963	-1.022056	-2.759194
H	-2.967080	0.524555	-3.973521	C	1.624927	0.158821	-2.989367
C	-2.725750	4.567326	-1.618759	C	2.354737	1.300187	-3.381536
H	-3.144173	4.293536	-2.600355	H	1.821314	2.228437	-3.600299
H	-3.477264	4.320382	-0.855534	C	5.917650	0.249023	-2.590770
H	-2.576146	5.659144	-1.604619	H	6.705394	-0.018892	-3.305482
C	-0.427232	4.085199	-2.545563	H	1.848155	-1.937877	-2.475514
H	-0.298189	5.159879	-2.749670	H	4.283905	2.224869	-3.639568
H	0.565469	3.663207	-2.334478	H	5.044290	2.946345	-1.068802
H	-0.817460	3.623434	-3.466303	H	2.541061	-0.768769	1.036916
C	-0.980386	3.465390	4.735259	O	2.483603	3.489936	-0.462106
H	-1.076653	2.767567	5.582696	O	5.167852	-1.236748	0.538050
H	-0.388573	4.329252	5.080035	C	3.154064	4.574117	-1.073448
H	-1.987471	3.834616	4.482408	H	3.527562	4.307077	-2.077288
C	1.083398	2.235010	3.921513	C	4.487096	-2.387092	1.013609
H	0.999977	1.475219	4.713628	H	4.049271	-2.222766	2.013285
H	1.567189	1.757488	3.057459	H	3.686011	-2.694879	0.323856
H	1.735254	3.046289	4.285942	H	3.997116	4.929037	-0.455844
C	3.112848	0.064735	0.644240	H	5.235288	-3.187749	1.079094
C	2.393784	1.221233	0.244829	H	2.415121	5.378068	-1.165250
C	3.155745	2.342191	-0.211253				

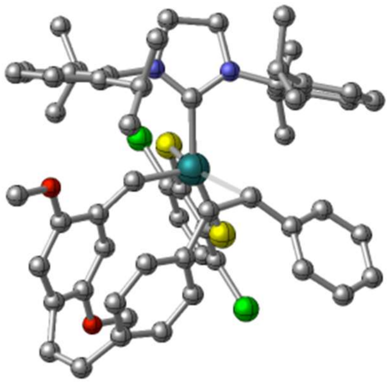

Zero-point correction= 1.061539 (Hartree/Particle)

Thermal correction to Energy= 1.127290

Thermal correction to Enthalpy= 1.128234

Thermal correction to Gibbs Free Energy= 0.963032

Sum of electronic and zero-point Energies= -4316.055306

Sum of electronic and thermal Energies= -4315.989555

Sum of electronic and thermal Enthalpies= -4315.988611

Sum of electronic and thermal Free Energies= -4316.153814

Int1-β(Ru-1a/OMe)

E(scf) = -4317.11657394 a.u.

ν_{min} = 15.6738 cm^{-1}

S	-0.681898	1.033610	-2.205535	C	-4.165122	-1.440200	-1.184164
C	-2.209256	-0.519811	-0.213721	C	-2.060628	-2.764233	-1.327286
Ru	-0.265817	0.247999	-0.081331	H	-4.669580	-2.242314	-0.620928
C	0.528832	-1.387651	-0.528888	H	-4.421449	-1.555507	-2.246207
C	0.011288	0.345358	2.530890	H	-5.383117	-0.016010	-0.010779
S	1.391805	1.953946	0.030645	C	-3.687155	3.302570	3.042123
N	-3.272727	0.256892	0.141501	C	-3.352605	2.655034	0.717969
N	-2.705015	-1.523781	-0.994470	C	-3.450903	0.940051	2.493790
C	-3.317025	1.302963	1.125323	C	-3.626113	1.963820	3.432896
C	-4.483800	-0.050383	-0.640050	C	-3.546545	3.640492	1.700582

H	-3.720425	1.717332	4.490699	C	0.173586	3.991289	3.512362
H	-3.578744	4.690677	1.402890	H	-1.031081	2.791415	2.190968
C	-0.819084	-5.190016	-1.941802	C	1.934605	2.824729	4.688077
C	-2.049026	-3.804022	-0.363289	H	2.084435	0.706522	4.313273
C	-1.523825	-2.957101	-2.619865	C	1.251246	4.012349	4.406223
C	-0.896496	-4.181013	-2.899097	H	-0.376984	4.909003	3.290210
C	-1.403994	-5.004735	-0.688969	H	2.764350	2.824967	5.399782
C	-2.768813	-3.686555	0.975054	H	1.550792	4.947051	4.887192
C	-1.655393	-1.914811	-3.719195	H	-3.829768	4.083354	3.793164
H	-0.460906	-4.343439	-3.886927	H	-0.315046	-6.130874	-2.177278
H	-1.370236	-5.812468	0.045590	Cl	-0.566708	2.385548	-5.092089
H	-4.602883	0.689013	-1.448561	Cl	3.443608	4.355748	-0.668104
H	-3.096452	-2.647412	1.091962	C	-4.497335	3.671410	-1.294664
H	-2.009264	-0.986360	-3.253565	H	-4.774339	4.600396	-0.769433
C	-3.193352	3.079059	-0.734222	H	-4.379562	3.912730	-2.363483
H	-2.940826	2.186550	-1.319493	H	-5.340636	2.969438	-1.193364
C	-3.434056	-0.520776	2.944712	C	-2.015203	4.051105	-0.911342
H	-2.731187	-1.055789	2.288690	H	-1.090946	3.629813	-0.490399
C	0.518093	2.277514	-2.565160	H	-1.836708	4.238023	-1.981528
C	0.559776	2.887234	-3.834658	H	-2.208791	5.021096	-0.425274
C	1.426010	2.706508	-1.563586	C	-4.815325	-1.178239	2.772187
C	1.463212	3.906835	-4.130187	H	-5.569431	-0.656307	3.382844
C	2.325373	3.746613	-1.882053	H	-5.156101	-1.160873	1.728919
C	2.351552	4.341139	-3.143709	H	-4.784467	-2.230989	3.094284
H	1.467155	4.358234	-5.123171	C	-2.940153	-0.721645	4.385174
H	3.062909	5.141993	-3.349463	H	-3.674161	-0.362610	5.123812
H	0.092574	-2.293391	-0.083065	H	-2.785358	-1.794632	4.577150
C	0.677237	-0.833910	2.268805	H	-1.988133	-0.203189	4.572031
H	0.028492	-1.700920	2.153878	C	-4.027514	-4.574513	0.990137
H	-1.073653	0.269047	2.522173	H	-4.587165	-4.437302	1.929341
C	0.493806	1.601341	3.148195	H	-4.703066	-4.342284	0.152329
C	-0.201509	2.799080	2.896348	H	-3.756274	-5.639736	0.911229
C	1.556235	1.629579	4.070007	C	-1.871212	-4.009805	2.177206

H	-1.601074	-5.077596	2.201682	C	3.123677	-0.226977	1.958020
H	-0.938246	-3.431646	2.161818	C	2.108235	-1.172742	2.203244
H	-2.399153	-3.786864	3.117894	C	2.474639	-2.542342	2.188495
C	-2.709187	-2.353851	-4.752848	C	5.944556	-2.389367	0.667325
H	-2.845360	-1.571471	-5.517036	H	6.916022	-2.479311	1.167561
H	-2.399820	-3.278830	-5.267365	H	3.837942	-4.446891	-1.042384
H	-3.687270	-2.547248	-4.283562	H	2.437848	0.280418	-1.719004
C	-0.316134	-1.597448	-4.400769	H	1.439448	-3.843366	-0.943567
H	-0.428544	-0.727056	-5.064777	H	4.832795	-0.306957	-1.731290
H	0.451588	-1.345840	-3.656535	O	1.487480	-3.448372	2.450446
H	0.044812	-2.445396	-5.006047	O	5.390496	0.270283	1.231483
C	2.735938	-0.757417	-1.592642	H	4.025072	-3.971886	1.710640
C	1.772624	-1.716941	-1.198379	H	2.856923	0.823838	1.939081
C	2.184700	-3.074045	-1.159829	C	1.739849	-4.827167	2.271757
C	3.529513	-3.414405	-1.224600	H	2.524809	-5.191552	2.957428
C	4.503278	-2.400084	-1.290420	H	2.034226	-5.056144	1.232758
C	4.085394	-1.095746	-1.618257	C	5.088455	1.655382	1.186288
C	5.842429	-2.611120	-0.658340	H	4.805937	2.043689	2.181628
H	6.718923	-2.904419	-1.247548	H	4.272549	1.873225	0.480329
C	3.773927	-2.919193	1.825437	H	0.799767	-5.345889	2.497684
C	4.733747	-1.974570	1.447651	H	6.003355	2.162155	0.851778
C	4.415618	-0.605475	1.588178				

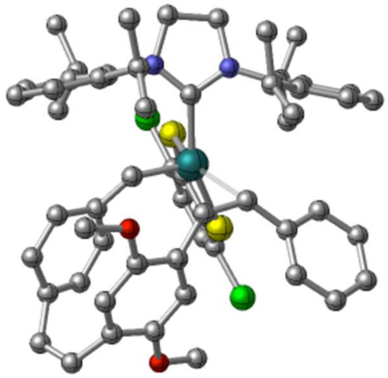

Zero-point correction= 1.061433 (Hartree/Particle)

Thermal correction to Energy= 1.127148

Thermal correction to Enthalpy= 1.128092

Thermal correction to Gibbs Free Energy= 0.962981

Sum of electronic and zero-point Energies= -4316.055141

Sum of electronic and thermal Energies= -4315.989426

Sum of electronic and thermal Enthalpies= -4315.988482

Sum of electronic and thermal Free Energies= -4316.153593

Int1-β′(Ru-1a/OMe)

E(scf) = -4317.13818672 a.u.

ν_{min} = 6.9862 cm^{-1}

c	-0.976032	-0.518987	1.463467	C	-2.791923	4.453501	0.690542
C	-3.704800	-0.023975	0.272688	C	-3.977680	4.230170	-1.406661
Ru	-1.862452	-0.303079	-0.616352	C	-5.342456	2.166633	-1.978306
C	-1.405144	1.368547	-1.210071	C	-2.836794	2.638076	2.471457
S	-0.131714	-1.662246	-1.436274	H	-2.162201	5.050344	1.352719
N	-4.431790	-1.081363	0.699515	H	-4.271269	4.651972	-2.370834
N	-4.397663	1.100258	0.540936	H	-5.998434	-1.166240	2.140568
C	-4.118030	-2.446650	0.383816	H	-5.649197	1.249620	-1.455233
C	-5.769745	-0.692158	1.177443	H	-2.967777	1.545995	2.447920
C	-5.647211	0.834173	1.277392	C	-3.418963	-2.804265	2.831021
C	-3.998683	2.430534	0.196141	H	-3.394948	-1.704962	2.800061
H	-6.490948	1.370762	0.819877	C	-4.919046	-2.032426	-2.036593
H	-5.545603	1.177495	2.318823	H	-4.946007	-0.996530	-1.671657
H	-6.532318	-1.010890	0.446906	C	0.596434	-1.303447	1.225016
C	-3.518727	-5.095367	-0.232206	C	1.496228	-1.425391	2.302023
C	-3.613311	-3.290378	1.400625	C	0.981214	-1.776381	-0.056192
C	-4.335020	-2.913041	-0.935739	C	2.763351	-1.980548	2.139972
C	-4.023817	-4.250260	-1.218772	C	2.271626	-2.330458	-0.196673
C	-3.319670	-4.618739	1.063136	C	3.153310	-2.433236	0.878436
H	-4.178816	-4.636587	-2.228343	H	3.445709	-2.042828	2.988481
H	-2.923711	-5.291615	1.825850	H	4.147204	-2.849119	0.723287
C	-3.163455	4.971305	-0.550676	H	-2.016277	1.606914	-2.110236
C	-4.421398	2.949695	-1.046664	H	-3.277700	-6.133514	-0.474376
C	-3.201376	3.175316	1.092952	H	-2.816624	5.963023	-0.851553

Cl	1.031374	-0.844341	3.897069	H	-1.187996	3.974512	3.038987
Cl	2.834920	-2.890791	-1.765441	C	-0.307038	2.295533	-1.013114
C	-4.602608	-3.239394	3.716661	C	0.701755	2.101878	-0.039569
H	-4.647177	-4.338478	3.791715	C	-0.161131	3.395688	-1.891693
H	-4.493907	-2.834335	4.735946	C	1.819396	2.915872	0.005638
H	-5.569819	-2.899610	3.314992	H	0.605356	1.277845	0.663124
C	-2.091753	-3.272112	3.448680	C	0.968227	4.208323	-1.847020
H	-1.242836	-3.046006	2.789763	H	-0.940975	3.591977	-2.632022
H	-1.917626	-2.755479	4.405506	C	2.013028	3.948526	-0.936492
H	-2.097353	-4.354613	3.655576	H	2.585168	2.724863	0.758111
C	-6.368418	-2.441236	-2.355647	H	1.072359	5.029136	-2.562135
H	-6.409316	-3.466779	-2.757307	C	3.260080	4.715890	-1.010312
H	-7.004831	-2.410759	-1.457196	H	3.152085	5.743352	-1.377436
H	-6.804999	-1.764821	-3.108590	C	4.519703	4.319175	-0.703294
C	-4.057788	-2.031846	-3.310365	H	5.288375	5.100102	-0.756407
H	-4.052196	-3.019142	-3.798487	C	5.036757	3.007408	-0.269022
H	-4.453540	-1.305599	-4.037123	C	4.608915	1.764509	-0.805367
H	-3.011876	-1.766433	-3.091100	C	6.036717	2.988386	0.722277
C	-6.624341	2.956103	-2.293756	C	5.105406	0.570737	-0.272564
H	-7.320807	2.338195	-2.883220	C	6.556866	1.794127	1.228718
H	-7.138581	3.268560	-1.371089	H	6.382939	3.945762	1.111762
H	-6.408167	3.863320	-2.880498	C	6.087503	0.551585	0.733016
C	-4.633367	1.722369	-3.268074	H	4.727638	-0.380907	-0.638841
H	-4.230261	2.585174	-3.822436	C	6.597085	-0.690159	1.333725
H	-3.800213	1.038241	-3.053418	H	6.857498	-0.586255	2.391621
H	-5.335856	1.191803	-3.930596	C	6.782662	-1.922505	0.803496
C	-3.792882	3.212876	3.535136	H	7.078071	-2.698613	1.519601
H	-3.582286	2.773466	4.523654	C	6.655989	-2.416402	-0.579912
H	-3.668520	4.305430	3.615314	C	6.889073	-1.615373	-1.717229
H	-4.849098	3.019998	3.291221	C	6.327251	-3.771677	-0.785817
C	-1.377611	2.902930	2.865118	C	6.750768	-2.139411	-3.002237
H	-1.136395	2.367809	3.796768	H	7.184781	-0.573611	-1.588546
H	-0.688361	2.550284	2.089026	C	6.181118	-4.295180	-2.071721

H	6.166677	-4.419023	0.081141
C	6.384298	-3.478799	-3.187436
H	6.934064	-1.498025	-3.868364
H	5.902859	-5.344028	-2.201925
H	6.268159	-3.883525	-4.195959
O	3.723357	1.808111	-1.828868
O	7.515085	1.734450	2.196063
C	3.058876	0.622189	-2.229960
H	3.757117	-0.126887	-2.639582
H	2.501127	0.172519	-1.393676
H	2.344102	0.916473	-3.008942
C	7.981773	2.928137	2.786415
H	7.166454	3.493582	3.273514
H	8.716455	2.634475	3.548257
H	8.476767	3.585663	2.048736

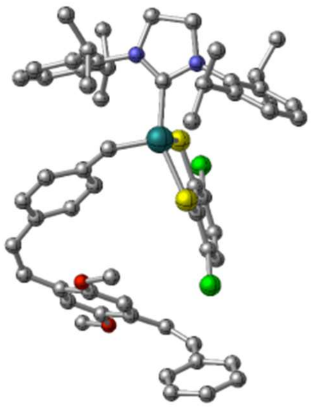

Zero-point correction= 1.059786 (Hartree/Particle)

Thermal correction to Energy= 1.126811

Thermal correction to Enthalpy= 1.127756

Thermal correction to Gibbs Free Energy= 0.952929

Sum of electronic and zero-point Energies= -4316.078401

Sum of electronic and thermal Energies= -4316.011375

Sum of electronic and thermal Enthalpies= -4316.010431

Sum of electronic and thermal Free Energies= -4316.185258

GII/III

E(scf) = -2210.18378060 a.u.

ν_{min} = 8.6550 cm^{-1}

Ru	0.275488	-0.827073	-0.237812	C	-2.318526	2.368184	-1.001455
Cl	0.632061	-0.799392	-2.580467	C	-2.355356	1.857366	1.406315
Cl	0.650224	-1.602811	1.962666	C	-3.720036	2.317835	-1.008399
C	-1.561964	-0.885573	-0.261215	C	-3.752449	1.814234	1.349683
H	-2.146097	0.016285	-0.463518	C	-4.452019	2.037240	0.153164
C	0.601768	1.079368	0.085914	H	-4.250347	2.482436	-1.950534
N	-0.229112	2.139446	0.232218	H	-4.312204	1.583406	2.260781
N	1.875186	1.540782	0.200504	C	3.060059	0.740868	0.128071
C	0.482263	3.400129	0.502737	C	3.609284	0.222493	1.321518
C	1.958556	2.992582	0.391907	C	3.688212	0.537782	-1.118060
C	-1.655505	2.134084	0.215608	C	4.738992	-0.597190	1.225937

C	4.816177	-0.293142	-1.160103	H	3.224307	-0.198907	3.406383
C	5.342302	-0.887117	-0.006373	H	3.570654	1.507427	3.045428
H	5.161825	-1.019671	2.142189	C	6.522299	-1.824117	-0.083804
H	5.299509	-0.473230	-2.124876	H	6.181309	-2.872438	-0.143000
C	-1.608593	1.530888	2.673789	H	7.138922	-1.629393	-0.974275
H	-2.297085	1.418036	3.522948	H	7.163507	-1.741940	0.807438
H	-1.044039	0.590699	2.557966	H	2.536850	3.227107	1.298634
H	-0.875615	2.311968	2.931563	H	2.466011	3.458631	-0.468555
C	-1.531940	2.557579	-2.273230	H	0.217274	3.775252	1.504440
H	-0.956296	1.645683	-2.507185	H	0.192641	4.167866	-0.230568
H	-2.194423	2.775732	-3.122526	C	-2.390187	-2.082042	-0.168321
H	-0.803732	3.380040	-2.189349	C	-1.902580	-3.360053	0.199295
C	-5.958240	1.947188	0.128759	C	-3.770431	-1.951803	-0.461521
H	-6.409669	2.613767	0.881608	C	-2.759323	-4.457565	0.253560
H	-6.368241	2.215746	-0.855746	H	-0.852082	-3.481640	0.468132
H	-6.292296	0.923104	0.366813	C	-4.623303	-3.052694	-0.407084
C	3.238420	1.255050	-2.365461	H	-4.161310	-0.967324	-0.733144
H	2.194219	1.581337	-2.305659	C	-4.119075	-4.309772	-0.051275
H	3.873445	2.143081	-2.532187	H	-2.367783	-5.436180	0.541852
H	3.330648	0.607719	-3.248445	H	-5.684539	-2.932911	-0.638988
C	3.058825	0.602106	2.672720	H	-4.786519	-5.174095	-0.005104
H	1.982550	0.806026	2.638723				

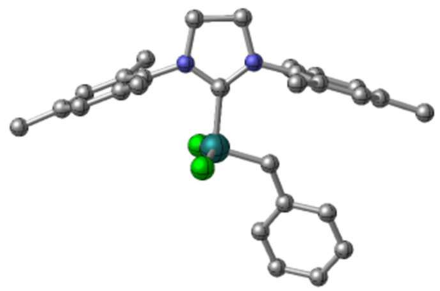

Zero-point correction= 0.531546 (Hartree/Particle)

Thermal correction to Energy= 0.567161

Thermal correction to Enthalpy= 0.568105

Thermal correction to Gibbs Free Energy= 0.459090

Sum of electronic and zero-point Energies= -2209.652234

Sum of electronic and thermal Energies= -2209.616620

Sum of electronic and thermal Enthalpies= -2209.615676

Sum of electronic and thermal Free Energies= -2209.724691

TS1-α-E

E(scf) = -3055.49856331 a.u.

ν_{min} = -267.9794 cm^{-1}

Ru	-0.564673	0.242609	0.138897	C	2.127920	4.961440	0.204962
Cl	0.063874	0.920152	-2.193892	H	2.267609	4.331985	2.262059
Cl	-1.420278	-0.147668	2.491874	H	1.658212	5.474252	-1.839385
C	-0.855628	-1.525464	-0.587596	C	-4.727527	0.687462	2.357733
H	-0.518715	-1.558854	-1.634043	H	-4.825077	-0.005696	3.205706
C	-1.965304	1.779332	0.093603	H	-3.799066	1.253495	2.496927
N	-3.300284	1.630574	0.017000	H	-5.580099	1.388277	2.408116
N	-1.678381	3.090418	0.216506	C	-3.473865	0.307142	-2.561464
C	-3.997444	2.931294	-0.025367	H	-2.383050	0.166394	-2.531316
C	-2.897125	3.900494	0.408581	H	-3.858876	-0.180127	-3.468411
C	-4.046741	0.409583	-0.085541	H	-3.635597	1.392130	-2.661651
C	-4.147589	-0.241243	-1.330980	C	-6.409933	-3.187085	-0.397151
C	-4.735204	-0.063254	1.050077	H	-6.373235	-3.774831	0.532366
C	-4.905692	-1.416896	-1.401534	H	-7.468506	-2.938179	-0.589087
C	-5.481657	-1.240425	0.924134	H	-6.062945	-3.830580	-1.218480
C	-5.575092	-1.935743	-0.287713	C	-0.795449	4.363269	-2.191581
H	-4.974359	-1.940451	-2.358910	H	-0.242823	4.790467	-3.040422
H	-6.001929	-1.628608	1.804908	H	-1.094616	3.338666	-2.456545
C	-0.382160	3.706303	0.229016	H	-1.711786	4.963130	-2.060276
C	0.393448	3.713544	1.403306	C	-0.073734	3.069300	2.682844
C	0.054211	4.354893	-0.946817	H	-1.168180	3.023577	2.755586
C	1.649486	4.336063	1.359353	H	0.273786	2.027394	2.754099
C	1.305244	4.978168	-0.930595	H	0.315735	3.613800	3.555553

C	3.509381	5.565249	0.168002	C	5.897143	-2.654216	-1.492368
H	4.252525	4.802363	-0.123926	H	6.589485	-3.278877	-2.069232
H	3.577661	6.385249	-0.563049	C	4.904938	-1.374410	1.264611
H	3.811727	5.951737	1.153327	C	5.396615	-0.821711	0.064168
H	-2.980102	4.189771	1.470165	C	4.698310	0.260325	-0.487159
H	-2.858253	4.815813	-0.195609	H	5.097188	0.717393	-1.392172
H	-4.862096	2.927743	0.650652	C	3.400103	0.599544	-0.060262
H	-4.357055	3.124495	-1.050387	C	2.782651	-0.191428	0.929626
C	-1.768572	-2.659978	-0.324191	C	3.610561	-1.048455	1.681034
C	-2.040330	-3.514026	-1.415843	H	3.147700	-1.626266	2.481344
C	-2.369804	-2.950913	0.915551	C	6.367122	-1.623218	-0.755291
C	-2.875042	-4.623106	-1.274012	H	7.441822	-1.407504	-0.736208
H	-1.585356	-3.299763	-2.386585	C	1.324705	-0.541587	1.083932
C	-3.196241	-4.064985	1.056992	H	1.697772	-0.984059	-2.123402
H	-2.194211	-2.279888	1.755527	H	4.475029	-4.506327	0.015259
C	-3.451696	-4.906232	-0.031980	H	0.364080	-2.464030	1.120995
H	-3.072018	-5.269273	-2.133223	H	1.011026	-0.520976	2.132306
H	-3.658644	-4.271258	2.025673	O	2.663023	1.579910	-0.627109
H	-4.106258	-5.773683	0.084733	O	5.660738	-2.350604	1.840752
C	2.273175	-1.804699	-1.691513	C	3.096541	2.163911	-1.836971
C	1.810969	-2.443975	-0.532519	H	2.284642	2.819109	-2.165397
C	2.551067	-3.536205	-0.040689	H	4.018359	2.756552	-1.692150
H	2.178555	-4.099495	0.819918	H	3.271919	1.400327	-2.614049
C	3.850498	-3.773418	-0.501616	C	5.142497	-3.070035	2.938566
C	4.422781	-2.929643	-1.471105	H	5.918683	-3.787511	3.237336
C	3.561032	-2.060932	-2.160858	H	4.917635	-2.409204	3.795512
H	3.963186	-1.437083	-2.962828	H	4.226392	-3.626153	2.669459
C	0.837628	-1.791341	0.399685				

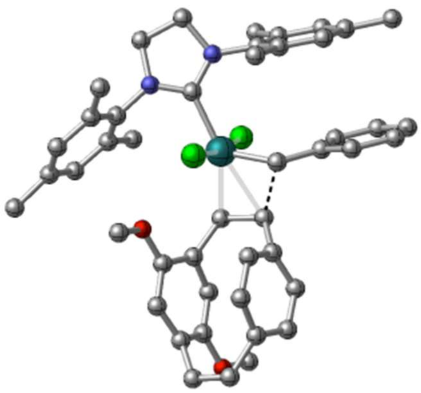

Zero-point correction= 0.827602 (Hartree/Particle)

Thermal correction to Energy= 0.879551

Thermal correction to Enthalpy= 0.880495

Thermal correction to Gibbs Free Energy= 0.741310

Sum of electronic and zero-point Energies= -3054.670961

Sum of electronic and thermal Energies= -3054.619012

Sum of electronic and thermal Enthalpies= -3054.618068

Sum of electronic and thermal Free Energies= -3054.757253

TS1-β-E

E(scf) = -3055.51154849 a.u.

ν_{min} = -225.6708 cm^{-1}

Ru	0.608896	0.343805	-0.250732	C	4.009000	-0.134007	1.554188
Cl	-0.323004	0.921205	2.077687	C	4.806757	0.343580	-0.723388
Cl	1.675165	0.064348	-2.483524	C	4.775360	-1.307177	1.546792
C	0.797954	-1.451157	0.431577	C	5.555464	-0.837424	-0.678459
H	0.356377	-1.531192	1.434939	C	5.546996	-1.680510	0.441116
C	1.910612	1.938106	0.080285	H	4.760747	-1.949812	2.430947
N	3.220956	1.862901	0.359679	H	6.156309	-1.112643	-1.550385
N	1.537398	3.230603	0.031828	C	0.189720	3.684621	-0.169961
C	3.809382	3.191564	0.622207	C	-0.347909	3.709284	-1.473593
C	2.676181	4.148373	0.216342	C	-0.573837	4.081150	0.947834
C	4.015082	0.667942	0.397426	C	-1.708489	4.013519	-1.618952

C	-1.925125	4.381839	0.746819	C	1.925556	-3.455675	1.289937
C	-2.520820	4.315780	-0.520203	C	2.397774	-2.850133	-1.006591
H	-2.144446	4.005349	-2.622060	C	2.787866	-4.547721	1.187041
H	-2.536869	4.654104	1.611833	H	1.403860	-3.265141	2.231620
C	4.876720	1.248131	-1.927518	C	3.250128	-3.948072	-1.110390
H	5.196919	0.689505	-2.818412	H	2.265811	-2.171410	-1.847920
H	3.901422	1.700273	-2.148570	C	3.451696	-4.799604	-0.017532
H	5.609266	2.059340	-1.768380	H	2.938531	-5.204958	2.047160
C	3.203024	0.248815	2.768208	H	3.773917	-4.134868	-2.051330
H	2.121515	0.151410	2.590161	H	4.128902	-5.653178	-0.103353
H	3.468208	-0.380346	3.629552	C	-2.342726	-1.848821	1.306053
H	3.372110	1.300668	3.049145	C	-1.940148	-2.277528	0.038347
C	6.377914	-2.939222	0.459022	C	-2.836397	-3.071116	-0.715693
H	6.031518	-3.639936	1.232115	C	-4.171555	-3.170228	-0.305352
H	6.334470	-3.457607	-0.510960	C	-4.631617	-2.518705	0.849233
H	7.438446	-2.708381	0.661284	C	-3.672610	-1.985638	1.732587
C	0.038758	4.214990	2.318366	C	-0.852063	-1.610586	-0.755454
H	-0.721491	4.086580	3.101329	C	-6.053989	-2.040553	0.918521
H	0.820378	3.464631	2.488818	H	-6.822685	-2.619957	1.443394
H	0.481486	5.218922	2.446500	C	-4.897319	-0.404553	-1.656547
C	0.498724	3.418567	-2.687030	C	-5.292619	-0.082967	-0.346361
H	1.520133	3.811921	-2.574263	C	-4.407406	0.695609	0.420177
H	0.603485	2.337467	-2.864854	H	-4.706603	1.021143	1.419536
H	0.053840	3.870584	-3.585176	C	-3.072063	0.841300	0.035611
C	-4.004430	4.522134	-0.686933	C	-2.605464	0.231002	-1.138520
H	-4.255400	4.895182	-1.691529	C	-3.569999	-0.235571	-2.056214
H	-4.530638	3.560787	-0.549729	H	-3.246941	-0.644823	-3.017687
H	-4.405824	5.226720	0.057602	C	-6.378479	-0.867359	0.330235
H	2.883636	4.681432	-0.725974	H	-7.411533	-0.501473	0.369947
H	2.447924	4.893968	0.990126	C	-1.218396	-0.288812	-1.357568
H	4.721883	3.331139	0.027155	H	-1.669261	-1.209468	1.871953
H	4.076964	3.274171	1.688250	H	-4.913978	-3.591386	-0.982714
C	1.715861	-2.584411	0.197469	H	-0.335751	-2.248477	-1.474938

H	-0.882804	-0.207514	-2.397017	H	-2.686050	-4.446118	-3.691048
H	-2.359839	1.290913	0.726044	H	-3.713536	-5.069699	-2.360436
H	-5.584653	-0.949290	-2.309499	C	-3.256658	-0.733095	3.729941
O	-2.379895	-3.520281	-1.921821	H	-3.852272	-0.386159	4.585083
O	-4.147908	-1.432896	2.880586	H	-2.447158	-1.389251	4.097854
C	-3.272743	-4.160548	-2.807680	H	-2.796173	0.132356	3.225065
H	-4.090368	-3.487113	-3.122778				

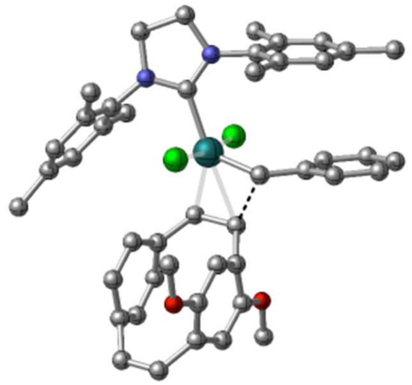

Zero-point correction= 0.827121 (Hartree/Particle)

Thermal correction to Energy= 0.879474

Thermal correction to Enthalpy= 0.880419

Thermal correction to Gibbs Free Energy= 0.739330

Sum of electronic and zero-point Energies= -3054.684427

Sum of electronic and thermal Energies= -3054.632074

Sum of electronic and thermal Enthalpies= -3054.631130

Sum of electronic and thermal Free Energies= -3054.772219

TS1-α-E'

E(scf) = -3055.51472098 a.u.

ν_{min} = -104.2218 cm^{-1}

Ru	-0.563519	0.250204	0.246251	C	-1.189983	-1.357533	-0.528739
Cl	0.314774	0.822192	-1.983118	H	-0.780397	-1.494494	-1.540252
Cl	-1.512063	0.034391	2.489603	C	-1.775172	1.910316	0.046719

N	-3.103147	1.976878	-0.133935	H	0.718324	4.124879	-3.041781
N	-1.285355	3.158461	0.175509	H	-0.755492	3.429497	-2.339311
C	-3.580492	3.371729	-0.235592	H	-0.421197	5.175248	-2.173998
C	-2.347723	4.178431	0.196912	C	0.081783	3.046485	2.796155
C	-4.005712	0.880338	-0.326806	H	-1.008188	3.013783	2.685836
C	-4.088369	0.269273	-1.594993	H	0.383852	2.064538	3.188458
C	-4.844642	0.490333	0.734370	H	0.340195	3.804029	3.553094
C	-4.995083	-0.783500	-1.761943	C	4.393811	3.952900	0.355297
C	-5.740309	-0.564478	0.512918	H	4.792710	4.172944	1.356903
C	-5.824341	-1.220537	-0.720474	H	4.814213	2.981481	0.039580
H	-5.059525	-1.272903	-2.738031	H	4.766920	4.715675	-0.345441
H	-6.385884	-0.886348	1.335123	H	-2.444740	4.591603	1.214922
C	0.109102	3.483180	0.272368	H	-2.110732	5.003353	-0.488649
C	0.787653	3.355782	1.499974	H	-4.448564	3.531765	0.417596
C	0.781611	3.912780	-0.894816	H	-3.886526	3.583514	-1.273554
C	2.180058	3.537878	1.507443	C	-2.155996	-2.416457	-0.224487
C	2.163143	4.102788	-0.827403	C	-2.288612	-3.438364	-1.196607
C	2.888589	3.879319	0.353617	C	-2.950783	-2.495734	0.937707
H	2.719989	3.397429	2.448431	C	-3.165597	-4.504864	-1.007804
H	2.696131	4.409418	-1.732077	H	-1.684421	-3.388588	-2.106240
C	-4.815549	1.200540	2.063705	C	-3.825898	-3.564454	1.124530
H	-5.247313	0.569700	2.854099	H	-2.870215	-1.707258	1.682826
H	-3.788890	1.456028	2.356204	C	-3.934952	-4.572957	0.159890
H	-5.409734	2.130777	2.028543	H	-3.248762	-5.284566	-1.768850
C	-3.240516	0.745362	-2.745733	H	-4.438303	-3.605855	2.028635
H	-2.171162	0.549769	-2.577009	H	-4.623544	-5.407872	0.314210
H	-3.538359	0.254563	-3.682999	C	1.926279	-2.180721	-1.479269
H	-3.333919	1.834280	-2.889229	C	1.522481	-2.589782	-0.201059
C	-6.758917	-2.386074	-0.922914	C	2.278899	-3.599961	0.428431
H	-7.565791	-2.397298	-0.174786	C	3.546202	-3.938661	-0.058697
H	-7.215135	-2.369232	-1.925145	C	4.074858	-3.288687	-1.189689
H	-6.206131	-3.336057	-0.830587	C	3.183303	-2.535703	-1.969635
C	0.037876	4.170838	-2.179972	C	0.651830	-1.753612	0.689158

C	5.547020	-3.051872	-1.365074	H	2.471017	0.928472	-0.872478
H	6.179772	-3.773315	-1.896135	H	5.433005	-2.075027	1.688990
C	4.827685	-1.327197	1.177875	H	3.546742	-2.056601	-2.882104
C	5.203538	-0.960538	-0.123418	H	1.958983	-3.990402	1.398500
C	4.431594	0.019340	-0.777279	O	3.097309	-1.454906	2.883278
C	3.166680	0.354604	-0.266786	O	4.875061	0.430393	-1.999917
C	2.673964	-0.243567	0.895456	C	4.031875	1.246858	-2.792305
C	3.585617	-0.964801	1.705365	H	3.813659	2.208875	-2.296541
C	6.083934	-1.903923	-0.891027	H	4.574927	1.439427	-3.727496
H	7.145214	-1.687107	-1.060292	H	3.070602	0.754407	-3.017174
C	1.230841	-0.527128	1.204934	C	3.891560	-2.331668	3.652080
H	1.349938	-1.427274	-2.014348	H	4.846663	-1.864398	3.953243
H	4.188265	-4.589682	0.541170	H	3.311321	-2.570498	4.553512
H	0.021494	-2.292943	1.402348	H	4.110407	-3.268288	3.108253
H	0.959065	-0.339429	2.246560				

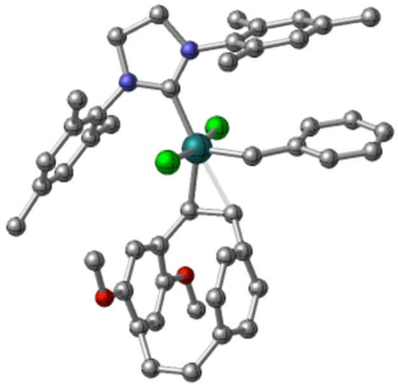

Zero-point correction= 0.825292 (Hartree/Particle)

Thermal correction to Energy= 0.877340

Thermal correction to Enthalpy= 0.878284

Thermal correction to Gibbs Free Energy= 0.739010

Sum of electronic and zero-point Energies= -3054.689429

Sum of electronic and thermal Energies= -3054.637381

Sum of electronic and thermal Enthalpies= -3054.636437

Sum of electronic and thermal Free Energies= -3054.775711

TS1-β-E'

E(scf) = -3055.50200999 a.u.

ν_{min} = -246.1137 cm^{-1}

Ru	0.673622	0.379106	-0.183094	H	3.887199	1.175952	-2.396826
Cl	0.050623	0.953605	2.184659	H	5.633791	1.448007	-2.139937
Cl	1.428611	0.005520	-2.574243	C	3.523052	0.096838	2.663754
C	0.687392	-1.474415	0.381324	H	2.425139	0.109239	2.586323
H	0.291630	-1.551514	1.398976	H	3.801781	-0.512288	3.535251
C	2.177802	1.797966	0.012941	H	3.828319	1.136681	2.863042
N	3.491581	1.582204	0.178456	C	6.078657	-3.548867	0.284499
N	1.943206	3.123339	-0.030591	H	5.305127	-4.333807	0.242053
C	4.245623	2.841722	0.341282	H	6.752654	-3.698265	-0.572233
C	3.180078	3.917580	0.061267	H	6.654629	-3.706586	1.210171
C	4.146038	0.305123	0.208674	C	0.695830	4.204442	2.366616
C	4.164692	-0.434700	1.408910	H	-0.007527	4.142276	3.208422
C	4.795967	-0.155285	-0.952500	H	1.427126	3.395313	2.484483
C	4.806954	-1.678079	1.405211	H	1.225945	5.170460	2.444868
C	5.428431	-1.404855	-0.901385	C	0.752445	3.481036	-2.660321
C	5.436077	-2.185125	0.260473	H	1.756112	3.933645	-2.642953
H	4.818570	-2.267013	2.326954	H	0.895074	2.401118	-2.819381
H	5.922360	-1.780622	-1.802043	H	0.211956	3.885628	-3.528107
C	0.626629	3.690768	-0.125713	C	-3.562454	4.659640	-0.310811
C	-0.001476	3.766266	-1.386289	H	-3.882731	5.059548	-1.284958
C	-0.029964	4.109983	1.049484	H	-4.092261	3.702923	-0.155181
C	-1.356935	4.119836	-1.424715	H	-3.894803	5.352170	0.477719
C	-1.380259	4.464479	0.955006	H	3.355149	4.455492	-0.884328
C	-2.074410	4.425845	-0.261987	H	3.099912	4.658960	0.868782
H	-1.867004	4.143587	-2.391997	H	5.085438	2.878384	-0.366157
H	-1.913958	4.747618	1.866803	H	4.654385	2.900615	1.362836
C	4.846435	0.676668	-2.208545	C	1.400767	-2.715919	0.007251
H	5.077644	0.051770	-3.082895	C	1.206648	-3.822477	0.866324

C	2.252357	-2.872767	-1.102592	C	-3.674959	0.601358	-1.608672
C	1.828050	-5.046260	0.615413	C	-6.476441	-0.380152	0.672756
H	0.546472	-3.710308	1.730530	H	-7.474518	0.056620	0.797129
C	2.874098	-4.097297	-1.349481	C	-1.337081	0.035522	-1.083892
H	2.427926	-2.021947	-1.757460	H	-0.602952	-1.929794	-1.520830
C	2.664243	-5.188251	-0.498059	H	-1.110152	0.210098	-2.140971
H	1.659835	-5.890603	1.288897	H	-2.194962	1.037421	1.415455
H	3.542288	-4.196107	-2.208820	H	-5.775723	0.249340	-1.937435
H	3.156102	-6.144119	-0.697234	H	-4.214911	-1.948709	2.716470
C	-2.543820	-2.093717	1.359260	H	-2.862274	-2.562772	-1.980388
C	-2.173352	-2.113325	-0.000589	H	-4.547423	0.870723	2.143804
C	-3.150734	-2.517779	-0.930121	H	-3.433586	0.483720	-2.669233
C	-4.505171	-2.600590	-0.592574	O	-1.573250	-1.880997	2.284801
C	-4.906338	-2.272808	0.719445	O	-5.499464	-2.794072	-1.501450
C	-3.908112	-2.168886	1.694573	C	-1.912623	-1.482190	3.597166
C	-1.034634	-1.381117	-0.678004	H	-2.435416	-2.287080	4.144195
C	-6.272132	-1.688886	0.942293	H	-0.965750	-1.248430	4.098818
H	-7.105194	-2.314214	1.284026	H	-2.538737	-0.574211	3.593853
C	-5.005382	0.479885	-1.197161	C	-5.173226	-2.923427	-2.869001
C	-5.318425	0.431083	0.173669	H	-4.520375	-3.795161	-3.056207
C	-4.314826	0.821300	1.076928	H	-6.121244	-3.067955	-3.404365
C	-2.983158	0.908606	0.673705	H	-4.676441	-2.016572	-3.258491
C	-2.631147	0.640689	-0.658182				

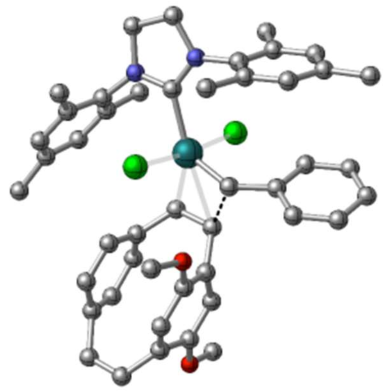

Zero-point correction= 0.827022 (Hartree/Particle)

Thermal correction to Energy= 0.879398

Thermal correction to Enthalpy= 0.880342

Thermal correction to Gibbs Free Energy= 0.739156

Sum of electronic and zero-point Energies= -3054.674988

Sum of electronic and thermal Energies= -3054.622612

Sum of electronic and thermal Enthalpies= -3054.621668

Sum of electronic and thermal Free Energies= -3054.762854

TS1-α-Z

E(scf) = -3055.51291045 a.u.

ν_{min} = -113.4068 cm^{-1}

Ru	0.644526	0.285897	0.457298	C	-0.667425	3.654264	0.929221
Cl	1.448890	0.522844	2.771173	C	-1.722539	4.009289	-1.646206
Cl	-0.045006	0.453964	-1.882681	C	-2.035464	3.903830	0.733546
C	1.402415	-1.453221	0.519950	C	-2.586623	4.055496	-0.540389
H	1.856775	-1.660183	1.499434	H	-2.130505	4.158391	-2.650129
C	1.936567	1.844000	0.051612	H	-2.684804	3.968340	1.611473
N	3.276247	1.802269	-0.015530	C	3.529963	0.267661	-2.438791
N	1.523250	3.118210	-0.077087	H	3.754962	-0.436589	-3.251585
C	3.864994	3.132858	-0.262531	H	2.437138	0.339353	-2.343294
C	2.649880	4.067286	-0.127659	H	3.890486	1.264342	-2.743910
C	4.076242	0.612727	0.017218	C	4.790043	1.180737	2.394300
C	4.759236	0.265790	1.197096	H	3.853691	1.741377	2.499915
C	4.176114	-0.172715	-1.151268	H	4.946064	0.611102	3.321637
C	5.467393	-0.945452	1.214207	H	5.624650	1.899143	2.305244
C	4.898387	-1.367648	-1.084033	C	6.238078	-3.115148	0.149029
C	5.530782	-1.784298	0.095689	H	6.876713	-3.267236	-0.735840
H	5.981314	-1.238874	2.134291	H	6.864495	-3.211954	1.048146
H	4.954216	-2.000462	-1.974078	H	5.502434	-3.937534	0.158043
C	0.150608	3.520851	-0.208770	C	-0.135444	3.540677	2.334928
C	-0.358593	3.744005	-1.508614	H	-0.529050	2.643125	2.834054

H	0.956769	3.456993	2.366938	C	-1.204582	-0.200298	1.445850
H	-0.445312	4.416402	2.926914	C	-5.972477	-1.766575	-0.728917
C	0.537071	3.697413	-2.720005	H	-7.005038	-1.544284	-1.022661
H	1.257218	2.871396	-2.652868	C	-3.607408	-3.724327	0.661169
H	-0.051739	3.557162	-3.637363	C	-4.023762	-3.261974	-0.601819
H	1.102530	4.639141	-2.831681	C	-3.043946	-2.690153	-1.428354
C	-4.073779	4.207593	-0.733693	H	-3.319250	-2.364375	-2.434511
H	-4.531814	3.218965	-0.914632	C	-1.808979	-2.300920	-0.910442
H	-4.560638	4.635742	0.155282	C	-1.513836	-2.495777	0.444789
H	-4.310225	4.842185	-1.601725	C	-2.360389	-3.354356	1.177473
H	2.541786	4.756442	-0.976384	H	-2.127473	-3.583693	2.221126
H	2.673383	4.663229	0.799586	C	-5.465251	-3.004449	-0.933627
H	4.317049	3.161390	-1.267491	H	-6.096794	-3.788696	-1.368431
H	4.651257	3.346664	0.474772	C	-0.670262	-1.546922	1.235148
C	1.727961	-2.523968	-0.428407	H	-1.160485	-1.674066	-1.516359
C	1.485688	-2.502444	-1.817187	H	-4.321093	-4.236016	1.312929
C	2.387069	-3.653130	0.113745	H	-2.249191	0.915664	-0.943731
C	1.889190	-3.564012	-2.625669	H	-5.496375	-1.504512	1.876584
H	0.998174	-1.633654	-2.253217	H	-1.013682	0.168810	2.455903
C	2.782454	-4.717878	-0.694236	H	-0.207018	-1.965735	2.133695
H	2.595688	-3.682355	1.186174	O	-3.215447	-0.771066	3.107532
C	2.536678	-4.674689	-2.071303	O	-4.595423	0.307391	-2.135679
H	1.698667	-3.523668	-3.701096	C	-4.102775	-1.460497	3.960804
H	3.287388	-5.579989	-0.251530	H	-3.593837	-1.556944	4.929285
H	2.850354	-5.503850	-2.710795	H	-5.047885	-0.906386	4.105452
C	-3.001890	0.470007	-0.300344	H	-4.337520	-2.469651	3.577172
C	-2.610055	0.062785	0.977711	C	-3.640061	0.869159	-3.017756
C	-3.604375	-0.474131	1.832460	H	-4.114765	0.905689	-4.007690
C	-4.826211	-0.878137	1.288992	H	-3.355807	1.892459	-2.717175
C	-5.101389	-0.730954	-0.078856	H	-2.720730	0.261682	-3.069792
C	-4.245438	0.093699	-0.834074				

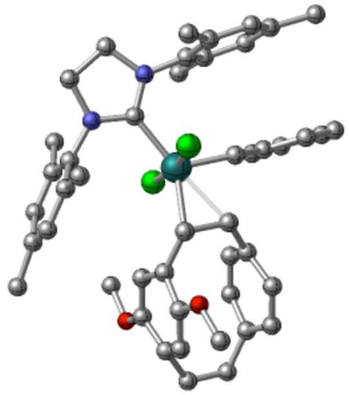

Zero-point correction= 0.825624 (Hartree/Particle)

Thermal correction to Energy= 0.877559

Thermal correction to Enthalpy= 0.878503

Thermal correction to Gibbs Free Energy= 0.739959

Sum of electronic and zero-point Energies= -3054.687286

Sum of electronic and thermal Energies= -3054.635351

Sum of electronic and thermal Enthalpies= -3054.634407

Sum of electronic and thermal Free Energies= -3054.772951

TS1-β-Z

E(scf) = -3055.49805972 a.u.

ν_{min} = -125.2554 cm^{-1}

Ru	0.653804	0.399161	0.369652	C	4.589689	-0.301739	1.472624
Cl	1.163151	0.644850	2.785952	C	4.254749	-0.647687	-0.938978
Cl	0.297663	0.464664	-2.021888	C	5.087620	-1.610200	1.559043
C	1.004462	-1.447259	0.673454	C	4.767758	-1.941138	-0.801464
H	1.222731	-1.665102	1.727743	C	5.164888	-2.450752	0.442133
C	2.228700	1.710878	0.077552	H	5.424444	-1.979460	2.532185
N	3.543193	1.448957	0.098760	H	4.841167	-2.575918	-1.688363
N	2.038509	3.032839	-0.086335	C	0.741021	3.619227	-0.282138
C	4.362916	2.668765	-0.039261	C	0.238215	3.761774	-1.591683
C	3.304114	3.778081	-0.202595	C	-0.022115	4.001106	0.841933
C	4.129570	0.146005	0.220299	C	-1.101095	4.139413	-1.744091

C	-1.358972	4.364706	0.635682	C	1.474818	-2.608215	-1.557929
C	-1.929715	4.391969	-0.643284	C	1.608923	-3.814310	0.535689
H	-1.516498	4.206001	-2.753683	C	1.866734	-3.758477	-2.237029
H	-1.973945	4.621304	1.502957	H	1.274545	-1.685828	-2.096021
C	3.857166	-0.109833	-2.288957	C	1.988715	-4.971396	-0.145728
H	4.050174	-0.849106	-3.078919	H	1.524024	-3.833632	1.625469
H	2.790222	0.156017	-2.320567	C	2.121046	-4.945771	-1.537991
H	4.424249	0.803888	-2.534792	H	1.977915	-3.730904	-3.324192
C	4.611993	0.602193	2.678440	H	2.189467	-5.890472	0.410393
H	3.756490	1.288089	2.688603	H	2.428097	-5.846256	-2.076486
H	4.583306	0.018255	3.609553	C	-2.874855	0.791137	-1.013243
H	5.542244	1.197788	2.692735	C	-2.618105	0.872848	0.362550
C	5.637047	-3.877779	0.565850	C	-3.730177	1.043155	1.216226
H	6.293471	-4.159577	-0.272690	C	-5.023824	0.782746	0.759636
H	6.182765	-4.049264	1.505732	C	-5.237621	0.385692	-0.572862
H	4.774233	-4.565151	0.545620	C	-4.174854	0.567893	-1.473241
C	0.584831	4.040991	2.220011	C	-1.367104	0.431733	1.058886
H	0.852545	3.035146	2.575344	C	-6.355177	-0.557301	-0.908037
H	1.506805	4.645463	2.225983	H	-7.336286	-0.192473	-1.235290
H	-0.114856	4.486672	2.941519	C	-4.508437	-2.368069	1.097160
C	1.113360	3.553900	-2.801047	C	-4.801784	-2.359219	-0.281958
H	1.842739	2.749680	-2.645752	C	-3.726871	-2.435550	-1.174284
H	0.511472	3.290147	-3.681524	H	-3.953274	-2.448575	-2.239399
H	1.662914	4.482507	-3.037538	C	-2.399461	-2.227423	-0.759945
C	-3.405003	4.638432	-0.825284	C	-2.142511	-1.940762	0.594095
H	-3.945363	3.675477	-0.796852	C	-3.187170	-2.167935	1.513589
H	-3.816619	5.271662	-0.024548	H	-2.986608	-1.971163	2.567128
H	-3.625442	5.112077	-1.794188	C	-6.144788	-1.884240	-0.757032
H	3.360671	4.278842	-1.181104	H	-6.958465	-2.592450	-0.952927
H	3.368300	4.547775	0.581348	C	-1.095669	-1.011186	1.151094
H	5.030787	2.580194	-0.909635	H	-2.036693	0.744438	-1.706563
H	4.985164	2.806845	0.857741	H	-5.842814	0.720090	1.480998
C	1.333828	-2.611893	-0.156200	H	-1.231207	0.940549	2.018928

H	-0.788080	-1.287797	2.163149	H	-2.041386	-3.143980	-3.323480
H	-3.567787	1.201664	2.286429	H	-2.257810	-1.356445	-3.337458
H	-4.326646	0.340879	-2.531811	C	-5.369777	-2.165047	3.322440
O	-5.576495	-2.382964	1.943631	H	-6.358750	-2.208282	3.798502
O	-1.363088	-2.140676	-1.623815	H	-4.724007	-2.941669	3.771354
C	-1.598979	-2.180303	-3.012194	H	-4.920924	-1.174183	3.518351
H	-0.622555	-2.056974	-3.494843				

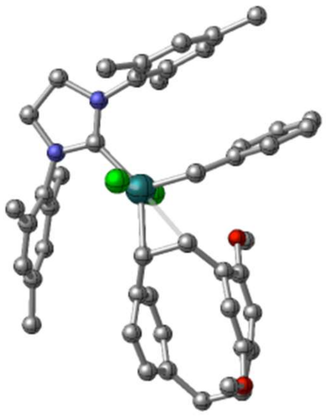

Zero-point correction= 0.825273 (Hartree/Particle)

Thermal correction to Energy= 0.877205

Thermal correction to Enthalpy= 0.878150

Thermal correction to Gibbs Free Energy= 0.738630

Sum of electronic and zero-point Energies= -3054.672787

Sum of electronic and thermal Energies= -3054.620854

Sum of electronic and thermal Enthalpies= -3054.619910

Sum of electronic and thermal Free Energies= -3054.759429

TS1-α-Z'

E(scf) = -3055.49696531 a.u.

ν_{min} = -286.0644 cm^{-1}

Ru	0.627359	0.253142	0.318405	C	0.942054	-1.655922	0.460105
Cl	1.336285	0.247092	2.867735	H	1.368168	-1.900546	1.441956
Cl	0.178910	0.529811	-2.058265	C	2.213106	1.593746	0.140867

N	3.515521	1.255266	0.149570	H	0.597968	2.613190	2.604216
N	2.106662	2.933868	0.078864	H	1.584658	4.072577	2.627502
C	4.396474	2.430730	0.008796	H	-0.105713	4.149106	3.176529
C	3.419239	3.600401	0.180432	C	1.416658	3.777595	-2.574424
C	4.062461	-0.070337	0.209801	H	1.660133	2.706358	-2.602127
C	4.592163	-0.541964	1.425609	H	0.914078	4.037391	-3.516874
C	4.114858	-0.847037	-0.967681	H	2.364218	4.341744	-2.539329
C	5.087177	-1.853902	1.461973	C	-2.817043	5.790646	-0.684776
C	4.622903	-2.146657	-0.878700	H	-3.610728	5.034019	-0.815845
C	5.095448	-2.676219	0.329830	H	-3.102017	6.414309	0.176310
H	5.482206	-2.238160	2.407064	H	-2.813872	6.422067	-1.586328
H	4.642230	-2.766821	-1.779060	H	3.524573	4.369840	-0.596097
C	0.895315	3.686288	-0.089603	H	3.510962	4.088780	1.164347
C	0.539848	4.096249	-1.390642	H	4.878574	2.417224	-0.982786
C	0.121679	4.039343	1.033570	H	5.183262	2.413365	0.774895
C	-0.651303	4.812299	-1.552902	C	1.220082	-2.756645	-0.491413
C	-1.073021	4.737827	0.813505	C	1.010352	-2.716182	-1.882869
C	-1.486996	5.113701	-0.469766	C	1.756416	-3.938983	0.064734
H	-0.944883	5.125351	-2.558990	C	1.330816	-3.813346	-2.683271
H	-1.695422	4.997427	1.674685	H	0.620091	-1.806540	-2.332951
C	3.652203	-0.293648	-2.289479	C	2.066027	-5.039810	-0.734067
H	3.929896	-0.965797	-3.113244	H	1.938297	-3.985935	1.141519
H	2.561607	-0.157978	-2.313892	C	1.857711	-4.979247	-2.116122
H	4.097064	0.694670	-2.489509	H	1.168346	-3.756056	-3.762705
C	4.705232	0.338248	2.643992	H	2.475487	-5.944850	-0.278201
H	3.920247	1.102285	2.671610	H	2.105518	-5.836169	-2.747902
H	4.622111	-0.254910	3.565779	C	-3.236638	0.911437	-0.229884
H	5.690492	0.838023	2.661761	C	-2.745324	0.282019	0.931338
C	5.583932	-4.101013	0.397978	C	-3.683627	-0.366302	1.757195
H	6.225546	-4.347712	-0.462921	C	-4.967569	-0.686011	1.306458
H	6.151363	-4.296844	1.319918	C	-5.335130	-0.343497	-0.011264
H	4.730378	-4.799278	0.370847	C	-4.522773	0.572055	-0.693291
C	0.565961	3.699206	2.431823	C	-1.328204	-0.112558	1.275737

C	-6.310791	-1.226503	-0.735652	H	-3.320592	-0.809235	2.684738
H	-7.359073	-0.931086	-0.861322	H	-4.825175	0.876091	-1.695019
C	-4.018813	-3.475129	0.167202	H	-1.544104	-1.183362	-1.739575
C	-4.448252	-2.795600	-0.987642	H	-4.735736	-4.055097	0.753608
C	-3.470573	-2.131203	-1.745773	O	-2.397744	1.734809	-0.899886
H	-3.760720	-1.645154	-2.680357	O	-5.831458	-1.484033	1.993545
C	-2.207231	-1.872032	-1.218135	C	-5.445350	-2.004192	3.247272
C	-1.882807	-2.304183	0.074742	H	-5.231046	-1.202813	3.977600
C	-2.742387	-3.236203	0.688648	H	-6.291931	-2.601120	3.612615
H	-2.481967	-3.649795	1.667616	H	-4.557363	-2.656353	3.164941
C	-5.886579	-2.425148	-1.194859	C	-2.702450	2.107436	-2.226280
H	-6.590579	-3.106863	-1.686694	H	-3.593122	2.760817	-2.269369
C	-0.939274	-1.545700	0.950358	H	-1.829677	2.654019	-2.596558
H	-1.086036	0.129219	2.315126	H	-2.865854	1.224215	-2.867372
H	-0.681504	-2.063697	1.879901				

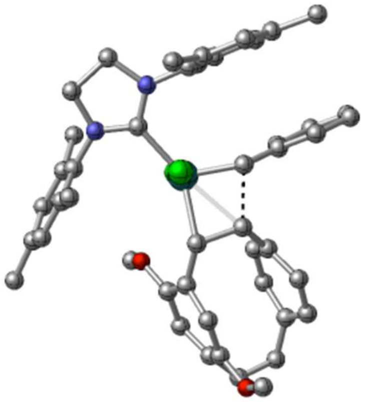

Zero-point correction= 0.827455 (Hartree/Particle)

Thermal correction to Energy= 0.879444

Thermal correction to Enthalpy= 0.880388

Thermal correction to Gibbs Free Energy= 0.740779

Sum of electronic and zero-point Energies= -3054.669510

Sum of electronic and thermal Energies= -3054.617521

Sum of electronic and thermal Enthalpies= -3054.616577

Sum of electronic and thermal Free Energies= -3054.756187

TS1-β-Z'

E(scf) = -3055.50942308 a.u.

ν_{min} = -272.3739 cm^{-1}

Ru	0.710567	0.355806	0.387331	H	2.365405	-0.036107	-2.414947
Cl	1.653202	0.374386	2.840764	H	3.966235	0.587736	-2.831813
Cl	-0.012401	0.630704	-1.947968	C	4.990710	0.517340	2.260110
C	0.868564	-1.570273	0.570988	H	4.207889	1.279299	2.355301
H	1.331907	-1.834972	1.530586	H	5.040305	-0.027759	3.213156
C	2.278583	1.674864	-0.014147	H	5.962497	1.023059	2.117054
N	3.573427	1.347327	-0.144362	C	5.315313	-4.108889	0.236839
N	2.134526	3.005058	-0.147726	H	5.885863	-4.451530	-0.641339
C	4.420217	2.520448	-0.433697	H	5.913498	-4.310044	1.137826
C	3.426810	3.693126	-0.321648	H	4.403829	-4.728615	0.288108
C	4.097986	0.013080	-0.070687	C	0.861465	3.826413	2.331592
C	4.717183	-0.423266	1.114829	H	0.951538	2.764668	2.607943
C	3.977375	-0.829808	-1.196209	H	1.884638	4.235090	2.327875
C	5.122099	-1.764236	1.187338	H	0.292631	4.340497	3.119316
C	4.395474	-2.158061	-1.071878	C	1.060571	3.712376	-2.744798
C	4.946219	-2.651504	0.119099	H	1.677058	2.806104	-2.688342
H	5.580055	-2.123788	2.113388	H	0.376606	3.598602	-3.597320
H	4.273661	-2.830977	-1.925112	H	1.722996	4.568533	-2.963408
C	0.864309	3.674282	-0.206928	C	-3.182272	5.134468	-0.403104
C	0.294679	3.944840	-1.467538	H	-3.834545	4.243089	-0.403391
C	0.197715	4.004459	0.990693	H	-3.467599	5.749705	0.463832
C	-1.007775	4.455535	-1.504094	H	-3.398457	5.702517	-1.320792
C	-1.106900	4.507203	0.898732	H	3.406989	4.323633	-1.221651
C	-1.738503	4.706791	-0.335158	H	3.627112	4.339689	0.547435
H	-1.474381	4.636850	-2.476646	H	4.859488	2.425393	-1.439544
H	-1.647076	4.738736	1.821058	H	5.240484	2.586527	0.294854
C	3.425483	-0.313996	-2.499884	C	0.991164	-2.703865	-0.374070
H	3.514225	-1.072869	-3.289534	C	0.785016	-2.643099	-1.766015

C	1.397640	-3.937493	0.182104	C	-3.088272	-2.537981	1.316905
C	0.974975	-3.771137	-2.564687	C	-6.070803	-1.524284	-0.731291
H	0.501798	-1.695944	-2.219932	H	-6.869301	-2.123432	-1.184707
C	1.579903	-5.067404	-0.615661	C	-0.976491	-1.254870	1.249389
H	1.577760	-4.002608	1.258316	H	-0.892615	0.515300	2.499916
C	1.370492	-4.987578	-1.996700	H	-0.679729	-1.731320	2.185084
H	0.817419	-3.697460	-3.643882	H	-3.314620	0.308402	3.017155
H	1.892247	-6.010177	-0.159428	H	-4.398093	1.318762	-1.715157
H	1.518992	-5.867911	-2.627351	H	-1.579184	-1.265312	-1.437959
C	-2.857500	1.248151	-0.216245	H	-5.221396	-2.820915	1.500054
C	-2.503107	0.808253	1.068454	H	-2.077466	1.527842	-0.921798
C	-3.551087	0.576661	1.983527	H	-5.628544	0.071404	2.219812
C	-4.864967	0.444666	1.532171	O	-2.755242	-2.825742	2.610067
C	-5.166543	0.571851	0.165450	O	-3.991683	-1.465807	-2.604093
C	-4.179467	1.139826	-0.659435	C	-3.763239	-3.219171	3.516024
C	-1.179637	0.231024	1.481958	H	-4.271496	-4.144160	3.188842
C	-6.291156	-0.225559	-0.427585	H	-3.265064	-3.406871	4.476646
H	-7.269109	0.226752	-0.631886	H	-4.521708	-2.427307	3.654476
C	-4.398662	-2.589765	0.823916	C	-2.971800	-1.049206	-3.493048
C	-4.715911	-2.111976	-0.457272	H	-2.213570	-1.839862	-3.639007
C	-3.653230	-1.832390	-1.338727	H	-3.462783	-0.841361	-4.453336
C	-2.350529	-1.729335	-0.832085	H	-2.459177	-0.140341	-3.137316
C	-2.073097	-1.960981	0.516523				

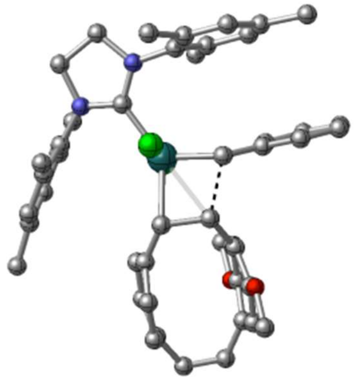

Zero-point correction= 0.827487 (Hartree/Particle)

Thermal correction to Energy= 0.879667

Thermal correction to Enthalpy= 0.880611

Thermal correction to Gibbs Free Energy= 0.740620

Sum of electronic and zero-point Energies= -3054.681936

Sum of electronic and thermal Energies= -3054.629756

Sum of electronic and thermal Enthalpies= -3054.628812

Sum of electronic and thermal Free Energies= -3054.768803

G-α-E’

E(scf) = -3055.52493197 a.u.

ν_{min} = 10.9260 cm^{-1}

Ru	-0.652505	0.343453	0.326154	C	0.450317	3.753590	-0.858583
Cl	0.107096	0.659511	-1.985411	C	1.686727	3.713586	1.660107
Cl	-1.202287	0.311238	2.699049	C	1.826331	3.955903	-0.727263
C	-1.656699	-1.087131	-0.308422	C	2.472625	3.887952	0.516520
H	-1.791824	-1.060582	-1.401369	H	2.161423	3.722146	2.645678
C	-2.032957	1.760695	0.039526	H	2.418005	4.148147	-1.626909
N	-3.357474	1.715168	-0.205052	C	-4.746084	0.403145	1.956881
N	-1.663717	3.055361	0.169256	H	-5.392964	-0.183599	2.624208
C	-3.962479	3.058998	-0.288850	H	-3.710628	0.338043	2.325226
C	-2.788805	3.991790	0.064628	H	-5.046545	1.460752	2.037039
C	-4.139351	0.553269	-0.499560	C	-3.487878	0.830227	-2.934457
C	-4.229610	0.114246	-1.835267	H	-2.405044	0.859312	-2.733305
C	-4.839966	-0.092319	0.539072	H	-3.645276	0.336859	-3.903902
C	-5.007267	-1.019260	-2.106728	H	-3.823410	1.875942	-3.034098
C	-5.605167	-1.217884	0.216589	C	-6.487714	-2.949655	-1.393555
C	-5.694370	-1.702864	-1.095603	H	-7.460323	-2.941355	-0.876528
H	-5.072390	-1.379249	-3.137443	H	-6.669868	-3.071816	-2.471591
H	-6.139141	-1.738869	1.016347	H	-5.941164	-3.841170	-1.041534
C	-0.294274	3.458894	0.307040	C	-0.216192	3.901133	-2.202230
C	0.297284	3.528249	1.585569	H	0.486035	3.676406	-3.016506

H	-1.075073	3.228546	-2.311075	C	1.127824	-1.535721	1.073756
H	-0.568162	4.939508	-2.337501	C	5.540578	-2.928803	-1.791834
C	-0.520146	3.506477	2.850966	H	6.084263	-3.667704	-2.392996
H	-1.526372	3.104164	2.692128	C	5.287911	-0.957864	0.700431
H	-0.042225	2.891762	3.625815	C	5.357340	-0.741337	-0.683845
H	-0.608290	4.535369	3.241504	C	4.404668	0.118922	-1.265616
C	3.974790	3.964848	0.604699	C	3.251409	0.459999	-0.539226
H	4.311281	4.290682	1.600383	C	3.058809	-0.007296	0.764700
H	4.406764	2.966382	0.415521	C	4.159908	-0.582037	1.437729
H	4.392867	4.651059	-0.148078	C	6.108915	-1.729434	-1.528220
H	-2.936078	4.516644	1.022544	H	7.101051	-1.497080	-1.932702
H	-2.592663	4.744860	-0.713288	C	1.710395	-0.321186	1.345991
H	-4.801650	3.143646	0.417181	H	1.174428	-1.608381	-1.698981
H	-4.354771	3.234547	-1.302939	H	4.743187	-4.238930	0.526420
C	-2.224516	-2.314578	0.239483	H	0.303864	-1.877303	1.705742
C	-2.704784	-3.263615	-0.696819	H	1.327273	0.261000	2.189275
C	-2.337546	-2.623112	1.614802	H	2.401943	0.907051	-1.050772
C	-3.257814	-4.473571	-0.281206	H	6.034149	-1.611508	1.151142
H	-2.638473	-3.034931	-1.762770	H	3.179903	-2.252025	-2.961630
C	-2.908315	-3.824721	2.027981	H	2.725943	-3.607494	1.788526
H	-1.990549	-1.894490	2.346506	O	3.953178	-0.933222	2.740667
C	-3.365894	-4.755453	1.085235	O	4.564693	0.392558	-2.590087
H	-3.610876	-5.195439	-1.021609	C	3.555536	1.119299	-3.266415
H	-2.995352	-4.042284	3.095499	H	3.398341	2.114382	-2.812383
H	-3.805803	-5.699848	1.416312	H	3.904475	1.247394	-4.300109
C	1.915791	-2.251198	-1.223474	H	2.590357	0.586692	-3.264343
C	1.841890	-2.482712	0.157559	C	4.924843	-1.705832	3.411663
C	2.788450	-3.358812	0.725568	H	5.900609	-1.189054	3.458731
C	3.935735	-3.713308	0.009031	H	4.553038	-1.858226	4.433866
C	4.154498	-3.206773	-1.285694	H	5.067691	-2.690845	2.931779
C	3.060559	-2.615111	-1.937607				

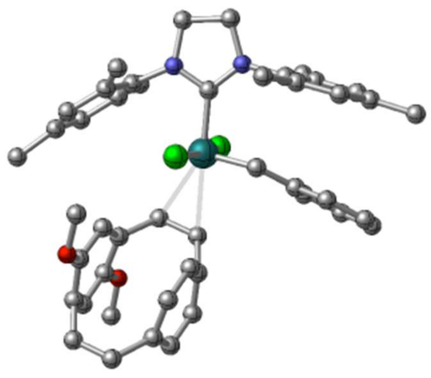

Zero-point correction= 0.825043 (Hartree/Particle)

Thermal correction to Energy= 0.877997

Thermal correction to Enthalpy= 0.878941

Thermal correction to Gibbs Free Energy= 0.737107

Sum of electronic and zero-point Energies= -3054.699889

Sum of electronic and thermal Energies= -3054.646935

Sum of electronic and thermal Enthalpies= -3054.645991

Sum of electronic and thermal Free Energies= -3054.787825

Int1-G-α-E'

E(scf) = -3055.58792095 a.u.

ν_{min} = 7.6323 cm^{-1}

Ru	2.940729	0.487614	0.141032	C	6.161887	-0.897232	-1.333379
Cl	2.805232	1.205635	-2.120150	C	5.941681	-1.849188	0.924348
Cl	3.937877	0.560122	2.287309	C	7.436439	-0.428443	-0.976962
C	-7.526884	-0.578837	-0.035381	C	7.218785	-1.372236	1.227309
H	-8.038584	0.059706	0.692335	C	7.974025	-0.640327	0.297198
C	2.927046	-1.406978	-0.340866	H	8.025692	0.109630	-1.725319
N	4.090505	-2.043444	-0.648232	H	7.636874	-1.571767	2.218185
N	1.908219	-2.261383	-0.623122	C	0.522804	-2.140015	-0.287679
C	3.881446	-3.332853	-1.316273	C	0.104307	-2.502411	1.006854
C	2.385619	-3.575178	-1.091000	C	-0.393853	-1.714969	-1.267652
C	5.407724	-1.566873	-0.352056	C	-1.263948	-2.444270	1.301709

C	-1.749654	-1.661273	-0.921514	C	-9.263275	-2.247610	0.551985
C	-2.206223	-2.028727	0.352807	C	-7.567742	-2.906324	-1.039384
H	-1.598552	-2.716384	2.306680	C	-9.854159	-3.508916	0.449946
H	-2.472284	-1.325580	-1.671316	H	-9.700532	-1.498648	1.218398
C	5.150217	-2.656967	1.919856	C	-8.156611	-4.165521	-1.140356
H	5.727168	-2.826514	2.839710	H	-6.673615	-2.691227	-1.628252
H	4.221619	-2.136042	2.191226	C	-9.303763	-4.475427	-0.397163
H	4.870294	-3.640136	1.506247	H	-10.748368	-3.737864	1.035394
C	5.666585	-0.749936	-2.749353	H	-7.717788	-4.914680	-1.804533
H	4.575140	-0.655470	-2.797525	H	-9.762550	-5.463851	-0.479120
H	6.103232	0.136492	-3.232073	C	-6.379799	2.256971	0.283495
H	5.963968	-1.629221	-3.347857	C	-5.821059	1.221779	-0.497388
C	9.327331	-0.091321	0.676438	C	-4.585617	1.482500	-1.130117
H	9.908256	-0.822059	1.261240	C	-3.919001	2.689548	-0.955069
H	9.916942	0.190055	-0.208797	C	-4.457630	3.704521	-0.139502
H	9.218861	0.810065	1.304141	C	-5.718942	3.471808	0.445951
C	0.083408	-1.306612	-2.636374	C	-6.440431	-0.092643	-0.676378
H	-0.759138	-1.021450	-3.282295	C	-3.743006	4.975188	0.093327
H	0.780788	-0.457234	-2.570142	H	-4.364229	5.878631	0.087173
H	0.630456	-2.124646	-3.133617	C	-1.738588	2.936556	1.285410
C	1.115817	-2.849282	2.070598	C	-1.437680	4.047438	0.481303
H	1.808481	-3.642445	1.749373	C	-0.176578	4.102899	-0.163564
H	1.738400	-1.970399	2.310798	C	0.696805	3.022528	-0.064710
H	0.623928	-3.180494	2.995586	C	0.368700	1.861664	0.678303
C	-3.677035	-1.961659	0.680830	C	-0.866729	1.853909	1.397164
H	-3.879912	-2.257593	1.720321	C	-2.415309	5.132367	0.297873
H	-4.068702	-0.942904	0.535829	H	-2.013437	6.150119	0.328388
H	-4.265685	-2.621508	0.022085	C	1.184748	0.666988	0.683354
H	2.190946	-4.343769	-0.323897	H	-7.348085	2.113788	0.767833
H	1.854437	-3.870797	-2.007124	H	-2.961538	2.852868	-1.452323
H	4.518252	-4.110833	-0.870349	H	-5.923355	-0.738433	-1.392847
H	4.137271	-3.249843	-2.386021	H	0.686314	-0.182040	1.156400
C	-8.109406	-1.916374	-0.189096	H	1.629093	3.021354	-0.624005

H	-2.692604	2.931575	1.807284	H	1.292011	6.299384	-2.095461
H	-6.175732	4.253822	1.058924	H	2.157920	5.314658	-0.870498
H	-4.138176	0.712761	-1.764805	C	-2.384810	0.642589	2.795690
O	-1.127561	0.766933	2.160340	H	-3.215216	0.700299	2.071757
O	0.068134	5.217517	-0.902219	H	-2.393051	-0.346721	3.269945
C	1.307830	5.332234	-1.576033	H	-2.526276	1.417288	3.569493
H	1.447669	4.524887	-2.316159				

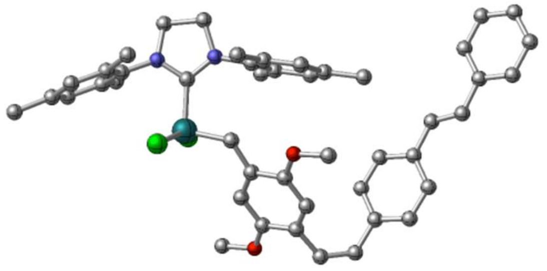

Zero-point correction= 0.825923 (Hartree/Particle)

Thermal correction to Energy= 0.880039

Thermal correction to Enthalpy= 0.880983

Thermal correction to Gibbs Free Energy= 0.731220

Sum of electronic and zero-point Energies= -3054.761998

Sum of electronic and thermal Energies= -3054.707882

Sum of electronic and thermal Enthalpies= -3054.706937

Sum of electronic and thermal Free Energies= -3054.856701

Int1-G-α-E'-Oru

E(scf) = -3055.60117917 a.u.

ν_{min} = 5.2968 cm^{-1}

Ru	1.570957	-0.644920	0.554145	H	-7.781502	1.129808	0.599376
Cl	2.328641	-1.012511	2.781600	C	3.006269	-1.783218	-0.234907
Cl	0.076561	-0.770587	-1.304943	N	2.882624	-3.137382	-0.272646
C	-7.332714	0.532614	-0.201245	N	4.219478	-1.481841	-0.759004

C	4.065465	-3.826674	-0.800528	H	6.325660	-1.248806	1.090023
C	4.975904	-2.660398	-1.211476	C	3.433972	-0.171869	-3.182726
C	1.715066	-3.875948	0.102218	H	3.773407	-1.178342	-3.474721
C	1.583773	-4.364384	1.417427	H	2.435016	-0.291491	-2.730500
C	0.738178	-4.148618	-0.881964	H	3.329877	0.429865	-4.096619
C	0.394155	-5.023780	1.761613	C	6.083430	3.862998	-1.673042
C	-0.426043	-4.816704	-0.490456	H	6.403572	4.030852	-2.713431
C	-0.629749	-5.238211	0.832265	H	5.250020	4.560208	-1.476912
H	0.274455	-5.387513	2.786568	H	6.912630	4.144149	-1.007116
H	-1.197581	-5.014782	-1.240410	H	5.141510	-2.610820	-2.300009
C	4.736015	-0.176591	-1.011394	H	5.962227	-2.694680	-0.724109
C	4.376997	0.480169	-2.204697	H	3.788884	-4.472631	-1.647948
C	5.550802	0.434635	-0.042506	H	4.513508	-4.462177	-0.019159
C	4.845687	1.784100	-2.403996	C	-8.132687	-0.625985	-0.612570
C	5.996737	1.741635	-0.285643	C	-9.366490	-0.864143	0.029499
C	5.647126	2.434542	-1.452970	C	-7.733632	-1.527892	-1.624095
H	4.561730	2.314628	-3.317664	C	-10.168576	-1.952664	-0.319655
H	6.616788	2.237024	0.466914	H	-9.697401	-0.179948	0.816091
C	0.970192	-3.806798	-2.331970	C	-8.533979	-2.614389	-1.972534
H	0.018070	-3.690972	-2.867901	H	-6.785603	-1.379515	-2.145251
H	1.534669	-2.874728	-2.449535	C	-9.756438	-2.834515	-1.323254
H	1.534996	-4.618011	-2.825572	H	-11.119692	-2.112707	0.194755
C	2.707789	-4.283583	2.418442	H	-8.202715	-3.298173	-2.758480
H	3.442599	-3.514498	2.157611	H	-10.380918	-3.687745	-1.599500
H	2.328105	-4.045975	3.422001	C	-5.735976	2.986884	0.705963
H	3.221313	-5.259556	2.476115	C	-5.330764	2.063219	-0.282266
C	-1.924781	-5.898998	1.236452	C	-4.077114	2.281764	-0.896162
H	-2.309428	-6.554969	0.439877	C	-3.257314	3.341012	-0.522444
H	-1.808488	-6.497941	2.152241	C	-3.644507	4.235643	0.495776
H	-2.702787	-5.141150	1.434466	C	-4.917570	4.049506	1.075536
C	5.847486	-0.269909	1.255961	C	-6.134220	0.912462	-0.699871
H	6.514343	0.329674	1.891499	C	-2.791969	5.349525	0.947349
H	4.913548	-0.461588	1.810457	H	-3.335695	6.262919	1.216590

C	-0.797908	2.981356	1.333135
C	-0.483384	4.292463	0.922417
C	0.805183	4.552578	0.378891
C	1.714236	3.507437	0.197797
C	1.367393	2.186108	0.545792
C	0.106606	1.950035	1.137623
C	-1.443702	5.394355	1.071211
H	-0.998031	6.362845	1.317476
C	2.220206	1.054218	0.266738
H	-6.705752	2.874587	1.195096
H	-2.296770	3.476794	-1.021497
H	-5.682477	0.313232	-1.497077
H	3.208955	1.304171	-0.124212
H	2.693706	3.676013	-0.249372
H	-1.768051	2.794055	1.787123
H	-5.258569	4.745420	1.847213
H	-3.741540	1.600084	-1.682739
O	-0.106843	0.643055	1.474375
O	1.054718	5.843388	0.031861
C	2.318677	6.181763	-0.501932
H	2.511857	5.660692	-1.457047
H	2.301625	7.264436	-0.683944
H	3.137650	5.947861	0.202079
C	-1.416526	0.198060	1.824005
H	-2.131015	0.437051	1.021036
H	-1.345000	-0.888302	1.950820
H	-1.737765	0.659941	2.770594

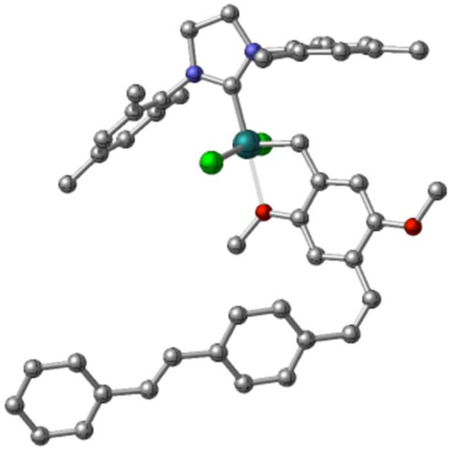

Zero-point correction= 0.825900 (Hartree/Particle)

Thermal correction to Energy= 0.880134

Thermal correction to Enthalpy= 0.881078

Thermal correction to Gibbs Free Energy= 0.728285

Sum of electronic and zero-point Energies= -3054.775279

Sum of electronic and thermal Energies= -3054.721045

Sum of electronic and thermal Enthalpies= -3054.720101

Sum of electronic and thermal Free Energies= -3054.872895

References

1. Schneider, R.V.; Waibel, K.A.; Arndt, A.P.; Lang, M.; Seim, R.; Busko, D.; Bräse, S.; Lemmer, U.; Meier, M.A.R. Sequence-definition in stiff conjugated oligomers. *Scientific Reports* **2018**, *8*, 17483.
2. Lidster, B. J.; Kumar, D. R.; Spring, A. M.; Yu, C.-Y.; Helliwell, M.; Raftery, J.; Turner, M. L. Alkyl substituted [2.2]paracyclophane-1,9-dienes. *Org. Biomol. Chem.* **2016**, *14*, 6079–6087.
3. Hsu, T.-W.; Kim, C.; Michaudel, Q. Stereoretentive Ring-Opening Metathesis Polymerization to Access All-cis Poly(p-phenylenevinylene)s with Living Characteristics. *J. Am. Chem. Soc.* **2020**, *142*, 11983–11987.
4. Mandal, H.; Ogunyemi, O.; Nicholson, J. L.; Orr, M.; Lalisse, R. F.; Rentería-Gómez, Á.; Gogoi, A. R.; Gutierrez, O.; Michaudel, Q.; Goodson III, T. Linear and Nonlinear Optical Properties of All-*cis* and All-*trans* Poly(*p*-phenylenevinylene). *J. Phys. Chem. C.* **2024**, *128*, 2518–2528.
5. Hsu, T.-W.; Kempel, S. J.; Michaudel, Q. All-cis poly(p-phenylene vinylene)s with high molar masses and fast photoisomerization rates obtained through stereoretentive ring-opening metathesis polymerization of [2,2]paracyclophane dienes with various aryl substituents. *J. Polym. Sci.* **2022**, *60*, 569–578.
6. (a) Lee, C.; Yang, W.; Parr, R. G. Development of the Colle-Salvetti Correlation-Energy Formula into a Functional of the Electron Density. *Phys. Rev. B* **1988**, *37*, 785–789. (b) Becke, A. D. Density Functional Thermochemistry. III. The Role of Exact Exchange. *J. Chem. Phys.* **1993**, *98*, 5648–5652.
7. (a) Grimme, S. Accurate description of van der Waals complexes by density functional theory including empirical corrections. *J. Comput. Chem.* **2004**, *25*, 1463–1473. (b) Grimme, S.; Antony, J.; Ehrlich, S.; Krieg, H. A consistent and accurate ab initio parametrization of density functional dispersion correction (DFT-D) for the 94 elements H-Pu. *J. Chem. Phys.* **2010**, *132*, 154104. (c) Grimme, S. Density functional theory with London dispersion corrections. *WIRES Comput. Mol. Sci.* **2011**, *1*, 211–228. (d) Ehrlich, S.; Moellmann, J.; Grimme, S. Dispersion-Corrected Density Functional Theory for Aromatic Interactions in Complex Systems. *Acc. Chem. Res.* **2012**, *46*, 916–926.
8. (a) Weigend, F.; Ahlrichs, R. Balanced basis sets of split valence, triple zeta valence and quadruple zeta valence quality for H to Rn: Design and assessment of accuracy. *Phys. Chem. Chem. Phys.* **2005**, *7*, 3297–3305. (b) Weigend, F. Accurate Coulomb-fitting basis sets for H to Rn. *Phys. Chem. Chem. Phys.* **2006**, *8*, 1057–1065.
9. Andrae, D.; Haeussermann, U.; Dolg, M.; Stoll, H.; Preuss, H. Energy-adjusted ab initio pseudopotentials for the 2nd and 3rd row transition-elements. *Theor. Chem. Acc.* **1990**, *77*, 123–41.
10. Gaussian 16, Revision C.01, Frisch, M. J.; Trucks, G. W.; Schlegel, H. B.; Scuseria, G. E.; Robb, M. A.; Cheeseman, J. R.; Scalmani, G.; Barone, V.; Petersson, G. A.; Nakatsuji, H.; Li, X.; Caricato, M.; Marenich, A. V.; Bloino, J.; Janesko, B. G.; Gomperts, R.; Mennucci, B.; Hratchian, H. P.; Ortiz, J. V.; Izmaylov, A. F.; Sonnenberg, J. L.; Williams-Young, D.; Ding, F.; Lipparini, F.; Egidi, F.; Goings, J.; Peng, B.; Petrone, A.; Henderson, T.; Ranasinghe, D.; Zakrzewski, V. G.; Gao, J.; Rega, N.; Zheng, G.; Liang, W.; Hada, M.; Ehara, M.; Toyota, K.; Fukuda, R.; Hasegawa, J.; Ishida, M.; Nakajima, T.; Honda, Y.; Kitao, O.; Nakai, H.; Vreven, T.; Throssell, K.; Montgomery, J. A., Jr.; Peralta, J. E.; Ogliaro, F.; Bearpark, M. J.; Heyd, J. J.; Brothers, E. N.; Kudin, K. N.; Staroverov, V. N.; Keith, T. A.; Kobayashi, R.; Normand, J.; Raghavachari, K.; Rendell, A. P.; Burant, J. C.; Iyengar, S. S.; Tomasi, J.; Cossi, M.; Millam, J. M.; Klene, M.; Adamo, C.; Cammi, R.; Ochterski, J. W.; Martin, R. L.; Morokuma, K.; Farkas, O.; Foresman, J. B.; Fox, D. J. Gaussian, Inc., Wallingford CT, 2016.
11. Tomasi, J.; Mennucci, B.; Cammi, R. Quantum mechanical continuum solvation models. *Chem. Rev.* **2005**, *105*, 2999–3093.

12. Zhao, Y.; Truhlar, D. G. A new local density functional for main-group thermochemistry, transition metal bonding, thermochemical kinetics, and noncovalent interactions. *J. Chem. Phys.* **2006**, *125*, 194101: 1–18.
13. Marenich, A. V.; Cramer, C. J.; Truhlar, D. G. Universal Solvation Model Based on Solute Electron Density and on a Continuum Model of the Solvent Defined by the Bulk Dielectric Constant and Atomic Surface Tensions. *J. Phys. Chem. B* **2009**, *113*, 6378–6396.
14. Legault, C. Y. (2009) CYLview, 1.0b, Universite de Sherbrooke: Sherbrooke, Canada, http://www.cylview.org.
15. Lu, T.; Chen, F., Multiwfn: A multifunctional wavefunction analyzer. *J. Comp. Chem.* **2012**, *33*, 580–592.
16. Humphrey, W.; Dalke, A.; Schulten, K., VMD – Visual Molecular Dynamics. *J. Mol. Graphics* **1996**, *14*, 33–38.
17. Vogiatzis, K. D.; Polynski, M. V.; Kirkland, J. K.; Townsend, J.; Hashemi, A.; Liu, C.; Pidko, E. A. Computational Approach to Molecular Catalysis by 3d Transition Metals: Challenges and Opportunities. *Chem. Rev.* **2019**, *119*, 2453–2523.
18. Bickelhaupt, F. M.; Houk, K. N. Analyzing Reaction Rates with the Distortion/Interaction-Activation Strain Model. *Angew. Chem. Int. Ed.* **2017**, *56*, 10070–10086.
